# Whole-genome sequencing reveals high complexity of copy number variation at insecticide resistance loci in malaria mosquitoes

**DOI:** 10.1101/gr.245795.118

**Published:** 2019-08

**Authors:** Eric R. Lucas, Alistair Miles, Nicholas J. Harding, Chris S. Clarkson, Mara K.N. Lawniczak, Dominic P. Kwiatkowski, David Weetman, Martin J. Donnelly, The Anopheles gambiae 1000 Genomes Consortium

**Affiliations:** 1 Liverpool School of Tropical Medicine, Liverpool L3 5QA, United Kingdom;; 2 Wellcome Sanger Institute, Hinxton, Cambridge CB10 1SA, United Kingdom;; 3 Big Data Institute, University of Oxford, Li Ka Shing Centre for Health Information and Discovery, Oxford OX3 7LF, United Kingdom

## Abstract

Polymorphisms in genetic copy number can influence gene expression, coding sequence, and zygosity, making them powerful actors in the evolutionary process. Copy number variants (CNVs) are however understudied, being more difficult to detect than single-nucleotide polymorphisms. We take advantage of the intense selective pressures on the major malaria vector *Anopheles gambiae*, caused by the widespread use of insecticides for malaria control, to investigate the role of CNVs in the evolution of insecticide resistance. Using the whole-genome sequencing data from 1142 samples in the *An. gambiae* 1000 genomes project, we identified 250 gene-containing CNVs, encompassing a total of 267 genes of which 28 were in gene families linked to metabolic insecticide resistance, representing significant enrichment of these families. The five major gene clusters for metabolic resistance all contained CNVs, with 44 different CNVs being found across these clusters and multiple CNVs frequently covering the same genes. These 44 CNVs are widespread (45% of individuals carry at least one of them) and have been spreading through positive selection, indicated by their high local frequencies and extended haplotype homozygosity. Our results demonstrate the importance of CNVs in the response to selection, highlighting the urgent need to identify the contribution of each CNV to insecticide resistance and to track their spread as the use of insecticides in malaria endemic countries intensifies and as the operational deployment of next-generation bed nets targeting metabolic resistance gathers pace. Our detailed descriptions of CNVs found across the species range provide the tools to do so.

Copy number variants (CNVs) are a form of genetic variation that occur when a genomic sequence is deleted or duplicated, potentially affecting both the structure and expression levels of coding sequences and playing a crucial role in evolution and adaptation (Redon et al. 2006; Chen et al. 2008; Emerson et al. 2008). Part of the importance of CNVs lies in the wide range of effects that they can have on the transcriptome. Increases in copy number (amplifications) encompassing the entire sequence of a gene can lead to elevated expression levels if new gene copies are associated with *cis*-regulatory sequences required for transcription (Handsaker et al. 2015). Alternatively, duplication or deletion of only part of a gene's sequence can lead to major changes in protein sequence. For example, in humans, a CNV spanning parts of two glycophorin genes creates a novel hybrid glycophorin associated with resistance to malaria (Leffler et al. 2017). CNVs can also allow alternative variants of a gene to appear in tandem on the same chromosome through heterogeneous gene duplication, creating constitutive heterozygotes. This can be seen in the mosquitoes *Anopheles gambiae* and *Culex pipiens,* where mutations in Acetylcholinesterase 1 (*Ace1*) cause resistance to carbamate and organophosphate insecticides, but carry a fitness cost in the absence of insecticide. This cost is mitigated in heterozygotes, leading to the spread of heterogeneous *Ace1* duplications in which the mutant and wild-type alleles co-occur (Labbé et al. 2007; Assogba et al. 2015).

Although the importance of CNVs is widely recognized, they typically receive less attention than single-nucleotide polymorphisms (SNPs) in investigations of genetic variation, likely because they are harder to identify. Population-level genome-wide analyses of CNVs are thus rare, and the extent of their impact on evolution is poorly understood (although a worldwide study has been conducted in humans [Sudmant et al. 2015]).

The malaria mosquito *An. gambiae* and its close sister species *An. coluzzii* are excellent organisms in which to study the evolution of CNVs at a population level for three reasons. First, these species are the major vectors of malaria in Sub-Saharan Africa (SSA) and are highly anthropophilic (Kiszewski et al. 2004). Consequently, they are heavily targeted by insecticides used in malaria control programs, creating intense selection pressure that drives rapid contemporary evolution. CNVs thus have an opportunity to contribute to the selective response to these pressures, providing a context in which their importance can be assessed. Second, CNVs can play a key role in the evolution of insecticide resistance through their ability to affect gene expression and allow coexpression of wild-type and mutant alleles (Bass and Field 2011; Weetman et al. 2018). Despite nearly two decades of genetic research into insecticide resistance, known resistance-associated SNPs are still unable to explain much of the variance in insecticide resistance (Donnelly et al. 2016). CNVs potentially represent a crucial source of missing variation that can potentially fill this gap. Third, the *Anopheles gambiae* 1000 Genomes Project (Ag1000G) has produced whole-genome sequencing data from 1142 individual *An. gambiae* and *An. coluzzii* from multiple locations in SSA, providing a unique opportunity to conduct genome-wide searches for CNVs from across the species’ distributions (https://www.malariagen.net/data/ag1000g-phase-2-ar1).

The two major mechanisms of insecticide resistance are target site resistance and metabolic resistance (Liu 2015). CNVs have been found to affect all three major insecticide target site genes in insects: *Ace1* (Assogba et al. 2015; Weetman et al. 2015), the *para* voltage-gated sodium channel (*Vgsc*) (Martins et al. 2013, 2017), and the gamma-aminobutyric acid (GABA) receptor Rdl (Remnant et al. 2013), usually combining resistant and wild-type alleles to provide resistance while mitigating its cost. However, duplications in *Ace1* are the only CNVs so far shown to play a role in *Anopheles* insecticide resistance, being associated with a resistance-conferring mutation and either increasing the resistance which it confers (Assogba et al. 2016) or diminishing the fitness cost of the mutation (Assogba et al. 2015).

Metabolic genes whose expression levels are associated with insecticide resistance have been reported in a wide range of species (Li et al. 2007) and are particularly concerning because up-regulation of a single gene can provide cross-resistance to a range of insecticides with different targets. For example, in *An. gambiae*, up-regulation of the cytochrome P450s *Cyp6m2* and *Cyp6p3* is associated with resistance to pyrethroids and bendiocarb (Edi et al. 2014). Despite the importance of metabolic insecticide resistance, the causative genetic variants often remain unidentified, although focused studies have identified cases in which CNVs play a critical role in metabolic resistance in a range of species. In *Drosophila*, duplication of the detoxification gene *Cyp6g1* has been implicated in resistance to DDT (Schmidt et al. 2010), whereas in *Cx. quinquefasciatus* resistance to permethrin is associated with increased expression of *Cyp9m10*, due in part to a duplication (Itokawa et al. 2011). Similarly, amplification of esterase genes leading to elevated expression provides increased resistance to organophosphates in the mosquitoes *Cx. pipiens* (Raymond et al. 2001) and *Aedes albopictus* (Grigoraki et al. 2015), and to several insecticides in the peach-potato aphid *Myzus persicae* (Devonshire et al. 1998; Field et al. 1999). In brown planthoppers, neofunctionalization of a duplicated copy of *Cyp6er1* has even created a novel gene variant providing resistance to the neonicotinoid imidacloprid (Zimmer et al. 2018). In *An. gambiae* and *An. coluzzii,* the most important metabolic genes that have been identified as major insecticide resistance candidates to date are *Gste2* (Mitchell et al. 2014), *Cyp6p3* (Müller et al. 2008; Edi et al. 2014), *Cyp6m2* (Stevenson et al. 2011; Mitchell et al. 2012; Edi et al. 2014), *Cyp6z1* (Chiu et al. 2008), and *Cyp9k1* (Main et al. 2015; Vontas et al. 2018). If CNVs play an important role in the evolution of insecticide resistance in *An. gambiae* and *An. coluzzii*, we would expect to find them among such genes.

Here, we perform an agnostic genome-wide scan of CNVs in *An. gambiae* and *An. coluzzii* using the whole-genome sequencing from Phase 2 of Ag1000G, which consists of 1142 wild-caught mosquitoes collected from 16 populations between 2000 and 2012 and sequenced with a mean coverage of 32×. We then focus on five regions of particular interest for metabolic insecticide resistance to investigate whether CNVs in these regions are under positive selection.

## Results

### Genome-wide CNV discovery

**Figure 1.**
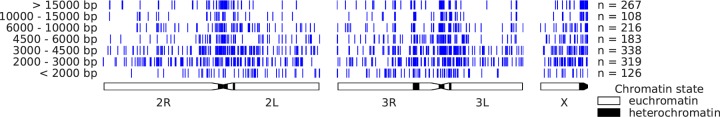
Distribution of CNVs in different size ranges across the genome. Blue bars represent regions of the genome in which a CNV was found. Numbers on the *left* indicate the CNV size range. Numbers on the *right* show the number of CNVs in that size range. Simulations showed a significant enrichment of CNVs in heterochromatic regions (0 out of 10,000 simulations had as many CNVs in the heterochromatin).

We first used short-read whole-genome sequencing data from the Ag1000G Phase 2 data set to find genomic regions with increased read coverage relative to the genomic average, controlling for GC richness and excluding 27 individual mosquitoes with unusually high coverage variance. Briefly, we filtered 300-bp genomic windows for mapping quality and rare levels of GC content (which prevented normalization) and identified regions covering at least five contiguous 300-bp windows of at least 1.5-fold increased coverage determined by a Hidden Markov Model (HMM). We then filtered this initial CNV set based on the relative likelihood of the observed read coverage in the absence of a CNV. After grouping identical CNVs between individuals according to their genomic locations and retaining only CNVs present in at least 5% of individuals in a population (or at least three individuals in small populations), we identified 1557 candidate CNVs (Fig. 1; Supplemental Data S1). The largest of these (203,100 bp) was the well-documented duplication in the insecticide target site *Ace1*. No CNVs were found in the other common insecticide target sites *Vgsc* or *Rdl*.

To estimate the accuracy of the CNV detection method, we performed simulations in which the coverage values obtained at each 300-bp window were randomly shuffled. These simulations indicated a low rate of CNVs being the result of random chance (false discovery rate of 0.003 at the sample level; 0.013 at the population level) and good rates of recovery for CNVs spanning at least 10 windows (≥85% at the sample level; ≥83% after population-level filtering), with a lower rate of recovery for CNVs spanning only five windows (31.4% at an individual level; 42.2% after population-level filtering).

To determine the genomic factors associated with the presence of CNVs, we investigated how CNVs are distributed relative to chromatin type and gene content. We found that CNVs were particularly abundant in heterochromatic regions. Of the 1557 CNVs, 534 (34.3%) were found in the heterochromatin, which covers only 9.3% of the genome (*P* < 0.0002; from 10,000 simulations, the number of heterochromatic CNVs ranged from 44 to 112, with a mean of 77). CNVs in heterochromatin were also significantly larger than in euchromatin (median for heterochromatin: 7200 bp, median for euchromatin: 3300 bp; Wilcoxon test, *n*_1_ = 534, *n*_2_ = 1023, *W* = 377,190, *P* < 0.0001) (Supplemental Fig. S1).

CNVs were also more abundant in gene-containing regions. Of the 1557 CNVs, 250 contained at least one gene, significantly more than expected by chance (*P* < 0.0002; across 10,000 simulations, the number of CNVs that contained genes ranged from 107 to 187, with a mean of 145). Because euchromatin typically contains more genes than heterochromatin, we repeated the simulation, focusing on euchromatic CNVs and randomizing their position within the euchromatin. Of the 1023 euchromatic CNVs, 226 contained at least one gene, again representing a significant enrichment for genic regions (*P* < 0.0002; across 10,000 simulations, the number of CNVs that contained genes ranged from 59 to 127, with a mean of 92). This enrichment was consistent for all sizes of euchromatic CNVs (Supplemental Table S1).

### CNVs are enriched in metabolic resistance gene families

We next explored whether CNVs were enriched for genes potentially involved in metabolic insecticide resistance. Overall, out of 10,939 genes included in the analysis, 267 (2.4%) were within at least one CNV (Supplemental Data S2). Of these 267 genes, 28 were candidate metabolic resistance genes (defined as a cytochrome P450, glutathione S-transferase or carboxylesterase, and referred to as “metabolic detox genes” from here on). Because many related genes occur in clusters, and are therefore not independently included in CNV events, we counted the number of CNVs that included at least one metabolic detox gene. Of the 250 CNVs that contained any genes, 27 contained at least one metabolic detox gene, significantly more than expected by chance (*P* < 0.0002; from 10,000 simulations, the number of gene-containing CNVs that contained detox genes ranged from 0 to 14, with a mean of 4). The same result was found when only euchromatic CNVs were considered, for all CNV sizes (Supplemental Table S2). Although there was some variation between populations in the number of metabolic detox genes found in CNVs (Table 1), this was not significant (Fisher's exact test: *P* = 0.08).

**Table 1.**
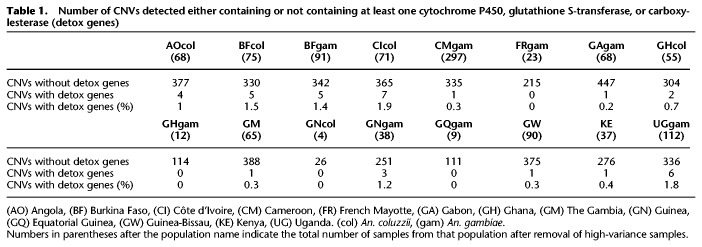
Number of CNVs detected either containing or not containing at least one cytochrome P450, glutathione S-transferase, or carboxylesterase (detox genes)

Genes found in CNVs were enriched for 13 molecular function Gene Ontology (GO) terms after multiple correction to a *Q*-value threshold of 0.05 (Supplemental Data S3), primarily reflecting an enrichment for two classes of genes: cytochrome P450s (significant GO terms included monooxygenase activity, heme binding, iron ion binding, oxidoreductase activity) and proteases (significant GO terms included several forms of peptidase activity). This result also held when heterochromatic CNVs were excluded, with 12 of the 13 GO terms remaining significant (Supplemental Data S3). No GO terms from biological process or cellular compartment ontologies were significantly enriched.

**Figure 2.**
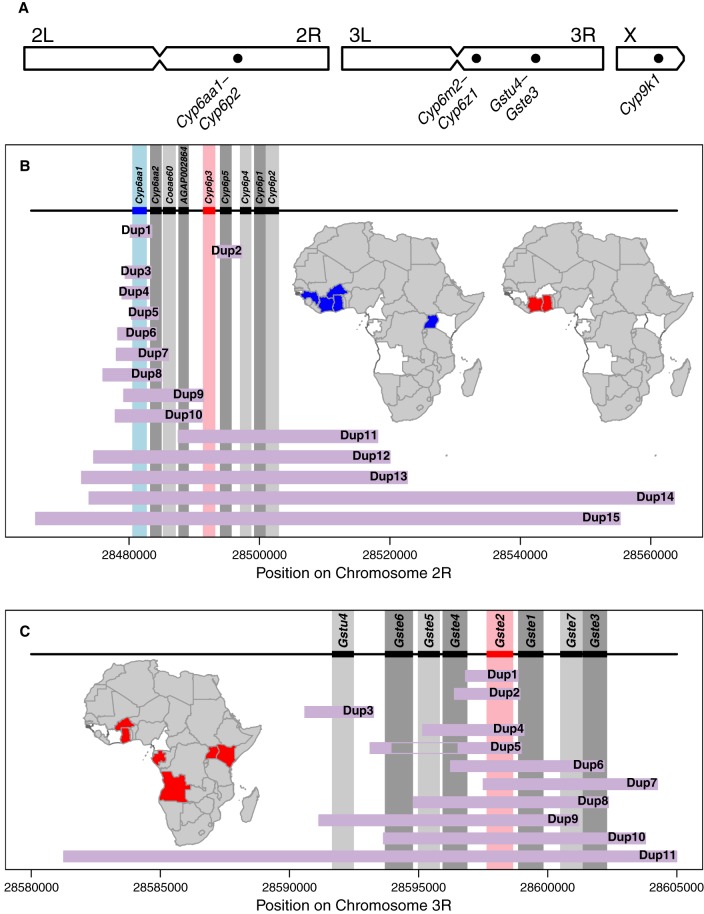
(*A*) CNVs in gene clusters known to be associated with metabolic insecticide resistance were found on all three chromosomes. (*B*) Of the 15 CNVs in *Cyp6aa1*–*Cyp6p2*, 13 include *Cyp6aa1* and five include *Cyp6p3*. *Inset* maps show countries in which at least 5% of individuals carried a CNV in *Cyp6aa1* (blue) and *Cyp6p3* (red), and the countries absent from the data set are shown in gray. (*C*) Of the 11 duplications in *Gstu4*–*Gste3*, 10 include *Gste2*. *Inset* map shows countries in which at least 5% of individuals carried a CNV in *Gste2* (red). Black rectangles and vertical gray bars show the positions of the genes in the cluster, with *Cyp6aa1*, *Cyp6p3*, and *Gste2* highlighted in color. Purple horizontal bars show the extent of each CNV, with the gap in Gstue_Dup5 showing the deletion within this amplification. CNV names are abbreviated to Dup# and refer to Cyp6aap_Dup# and Gstue_Dup# in panels *B* and *C*, respectively. Further details on each of these CNVs, and of those from the other gene clusters, are presented in Supplemental Data S5–S8.

The 28 metabolic detox genes found inside CNVs were predominantly from gene clusters that have previously been implicated in insecticide resistance (Supplemental Data S2), with 16 of the 28 genes being members of the glutathione S-transferase epsilon cluster (*Gste* on Chromosome 3R) or one of four cytochrome P450 clusters (*Cyp6p* on Chromosome 2R, *Cyp6m* on Chromosome 3R, *Cyp6z* on Chromosome 3R, and *Cyp9k1* on Chromosome X). This again indicates that genes involved in metabolic insecticide resistance have been the focus of amplification events. We therefore performed a detailed analysis of the CNVs around these five gene clusters (Fig. 2A). Because the *Cyp6aa1/Cyp6aa2* genes, which are adjacent to the *Cyp6p* cluster, were also highly represented in the list of amplified genes (Supplemental Data S2), we extended the study region around *Cyp6p3* to include these genes.

To more accurately determine the number of CNVs in each of these five gene clusters, we used discordant read pairs and reads aligning to CNV breakpoints to distinguish different CNVs. Three of the five gene clusters showed high numbers of different CNVs. We identified 16 CNV alleles in *Cyp9k1* (named Cyp9k1_Dup1–16) (Supplemental Data S7; Supplemental Fig. S4), 15 in the *Cyp6aa1*–*Cyp6p2* cluster (Cyp6aap_Dup1–15) (Fig. 2B; Supplemental Data S4; Supplemental Fig. S1), 11 in the *Gstu4*–*Gste3* cluster (Gstue_Dup1–11) (Fig. 2C; Supplemental Data S5; Supplemental Fig. S2), one in *Cyp6m2* (Cyp6m_Dup1) (Supplemental Data S6; Supplemental Fig. S3), and one in *Cyp6z3–Cyp6z1* (Cyp6z_Dup1) (Supplemental Data S6; Supplemental Fig. S3).

Several CNV alleles were found across different populations (for example, Cyp6aap_Dup7 was found in *An. coluzzii* from Burkina Faso, Côte d'Ivoire, Ghana, and Guinea) (Supplemental Data S4), although none were found in all populations of either species (Supplemental Data S8). Furthermore, multiple CNV alleles covering the same genes could be found in the same population (for example, Cyp9k1_Dup4, Dup11, and Dup15 in *An. gambiae* from Burkina Faso) (Supplemental Data S7). For the *Cyp6aa/p* cluster, CNVs were primarily found in *An. coluzzii* from Burkina Faso, Côte d'Ivoire, and Ghana (Table 2). In *Cyp9k1*, CNVs were primarily found in *An. gambiae* from Burkina Faso, Ghana, and Guinea. Some genes were amplified at very high frequency in certain populations (Table 2). For example, >92% of *An. gambiae* from Burkina Faso had a CNV in *Cyp9k1*, and >90% of *An. coluzzii* from Côte d'Ivoire had a CNV covering genes in the *Cyp6aa/p* cluster. Overall, 511 of the 1142 samples in this study (45%) carried at least one of the CNVs described in these five gene clusters.

**Table 2.**
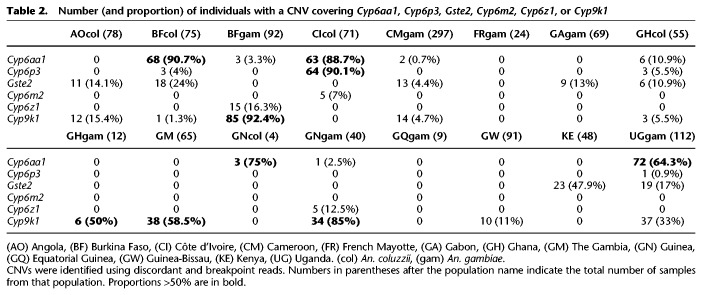
Number (and proportion) of individuals with a CNV covering *Cyp6aa1*, *Cyp6p3*, *Gste2*, *Cyp6m2*, *Cyp6z1*, or *Cyp9k1*

**Figure 3.**
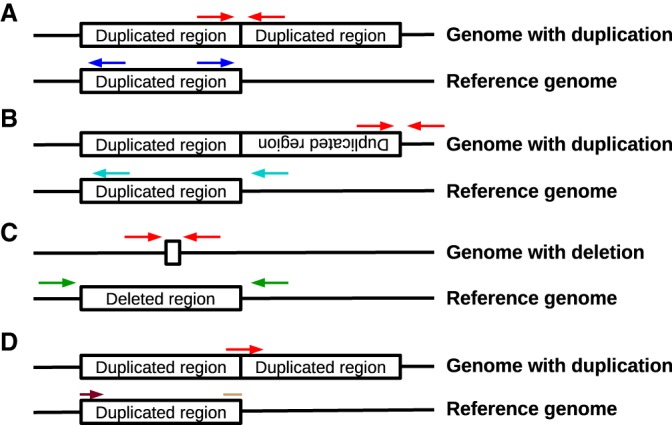
Three types of discordant read pairs (*A*–*C*) and breakpoint reads (*D*) were used to identify different CNV alleles. (*A*) In tandem duplications, read pairs derived from segments spanning the CNV breakpoint (red arrows) align facing away from each other around the breakpoint on the reference genome (dark blue arrows). (*B*) In tandem inversions, read pairs derived from segments spanning the end of the inverted segment (red arrows) align facing in the same direction as each other around the breakpoint on the reference genome (cyan arrows). (*C*) In deletions, read pairs derived from segments spanning the deleted sequence (red arrows) align in the correct orientation around the breakpoint, but farther apart than expected given the insert size of the sequencing library (green arrows). (*D*) In any of the aforementioned types of CNV (tandem duplication shown here as an example), reads crossing the breakpoint (red arrow) will only partially align on either side of the breakpoint. For the tandem duplication shown here, the start of the read (light brown start of an arrow) aligns at the end of the duplicated region, whereas the end of the read (dark brown end of an arrow) aligns at the start of the duplication.

We used the patterns of discordant reads supporting each of the CNVs to putatively determine the type of duplication event that caused them (Fig. 3). Of the 44 CNVs, 33 are supported by read pairs mapping facing away from each other in the genome and spanning the region of increased coverage, indicating a tandem duplication (Supplemental Data S4–S7), and two CNVs were supported by read pairs mapping in the same orientation, indicating a tandem inversion (Supplemental Data S4, S7). The remaining CNVs were supported by reads whose mate or soft-clipped bases mapped elsewhere on the genome, often in multiple genomic locations, suggesting a role for transposable elements (TEs) in the duplication event (Supplemental Data S4–S7). Two of these CNVs had flanking sequences that matched known transposable elements in *An. gambiae*. The soft-clipped bases of reads mapping to the breakpoints of Gstue_Dup7 and Gstue_Dup8, respectively, returned significant BLAST hits to a hAT DNA transposon (HATN1_AG; *E*-value = 2 × 10^−26^) and a transposon bearing similarity to mariner/Tc1-like elements (IKIRARA1; *E*-value = 10^−34^) (Leung and Romans 1998), in the Repbase database (Bao et al. 2015).

The CNVs were differentiated based on their patterns of discordant read pairs, which are influenced by the position of the CNV breakpoints and the orientation of the duplication (Fig. 3). It is possible that independent duplication events with very similar breakpoints could appear to be the same CNV (for example, Cyp6aap_Dup1 may in fact represent two different CNVs) (Supplemental Data S4), making the real number of CNV events even greater than reported. Equally, a mutation that disrupts the breakpoint of a CNV could affect its pattern of discordant reads and lead to a single CNV event appearing as two different CNV alleles. Although we cannot exclude this possibility, analysis of the haplotype backgrounds of the different CNVs suggested that this is not the case for at least most of the CNV alleles (Supplemental Data S8).

### CNVs in metabolic resistance genes are under positive selection

Several CNV alleles were found at high local frequencies (Supplemental Data S9), suggesting that they are likely to be under positive selection. To investigate this possibility, we phased the CNV genotype calls onto the Ag1000G Phase 2 haplotype scaffold and calculated extended haplotype homozygosity (EHH) for the CNV alleles present in at least 5% of individuals in a population.

**Figure 4.**
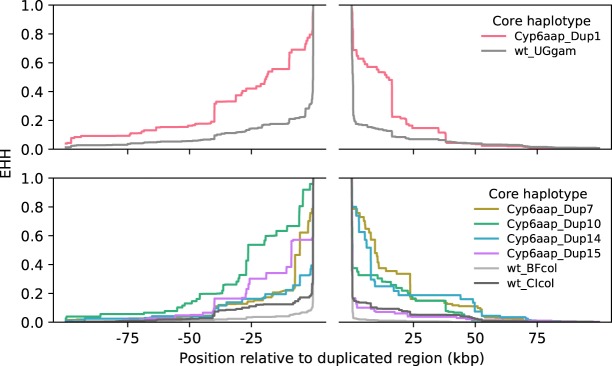
Evidence for prolonged linkage disequilibrium around CNVs in the *Cyp6aa1–Cyp6p2* gene cluster. Extended haplotype homozygosity (EHH) decay was calculated around CNV and non-CNV (WT) haplotypes using SNPs from outside the region containing CNVs (break in the *x*-axis): (BF) Burkina Faso; (CI) Côte d'Ivoire; (UG) Uganda; (col) *An. coluzzii*; (gam) *An. gambiae*.

**Figure 5.**
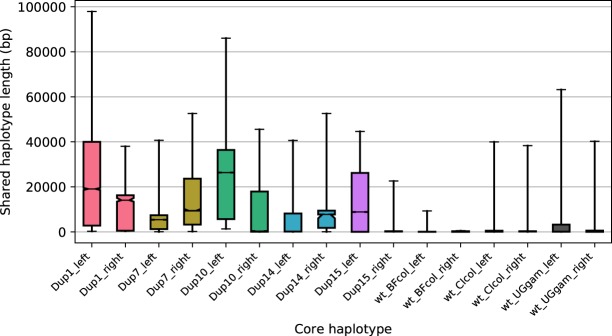
Lengths of pairwise shared haplotypes are greater between samples sharing a CNV allele than between wild-type samples. Shared haplotype lengths were calculated on either side of the CNV-containing region of the *Cyp6aa/p* gene cluster. Non-CNV (WT) samples were taken from the same populations as the focal CNV alleles. Bars show the distribution of shared haplotype lengths between all haplotype pairs with the same core haplotype. Bar limits show the interquartile range, fliers show the 5th and 95th percentiles, horizontal black lines show the median, and notches in the bars show the bootstrapped 95% confidence interval for the median. The names of the CNVs (Cyp6aap_Dup#) are abbreviated as Dup#: (BF) Burkina Faso; (CI) Côte d'Ivoire; (UG) Uganda; (col) *An. coluzzii*; (gam) *An. gambiae*.

Rates of EHH decay around CNV alleles were consistently lower than for wild-type (WT) haplotypes (Fig. 4; Supplemental Figs. S6, S8, S10), supporting our contention that these alleles are reaching high frequency through positive selection. Furthermore, the median length of shared haplotypes was significantly higher between pairs of haplotypes carrying the same CNV allele than between wild-type haplotypes from the same population (bootstrapped 95% confidence intervals for the medians did not overlap) (Fig. 5; Supplemental Figs. S7, S9, S11).

**Figure 6.**
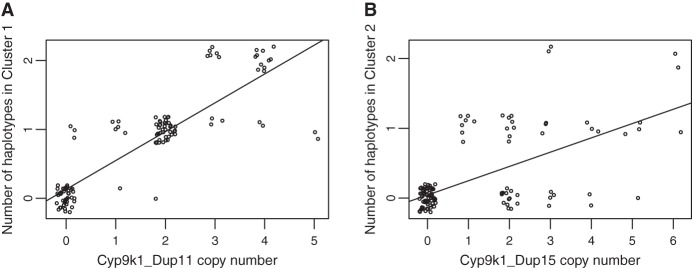
The two main haplotype clusters around *Cyp9k1* in Burkina Faso, Ghana, and Guinea are associated with respective CNV alleles. Points are jittered to show overlapping data. Lines show least-squares regression through the data. (*A*) Strong correlation between Cyp9k1_Dup11 and haplotype Cluster 1. Most of the points lie on a line of slope 0.5, indicating that Cyp9k1_Dup11 is found most frequently as a triplication (two extra copies per chromosome), although both lower and higher copy number versions of this CNV exist. (*B*) Weaker correlation between Cyp9k1_Dup15 and haplotype Cluster 2.

Phasing of CNV genotype calls was only possible for simple duplications, where the zygosity of the CNV alleles could be determined from the copy number estimates. For CNV alleles with higher copy numbers (triplications and above), this was not possible; thus the EHH decay could not be calculated. In the case of *Cyp9k1*, the CNV with the highest frequency (Cyp9k1_Dup11, found in *An. gambiae* from Burkina Faso, Ghana, and Guinea) could not be phased. We therefore investigated whether this CNV was associated, at the sample level, with haplotypes under selection. Hierarchical clustering of the haplotypes in these three populations revealed two large cross-population haplotype clusters around *Cyp9k1*, indicating selective sweeps (Supplemental Fig. S12). Cluster 1 was very strongly associated with Cyp9k1_Dup11 in both males (Fisher's exact test, *P* < 0.0001) (Supplemental Table S3) and females (Spearman's rank correlation: ρ = 0.9, *P* < 0.0001) (Fig. 6A). Cluster 2 was associated with the presence of Cyp9k1_Dup15, but the correlation was not as strong as between Cluster 1 and Cyp9k1_Dup11 (Spearman's rank correlation: ρ = 0.65, *P* < 0.0001) (Fig. 6B; Supplemental Table S4).

### A *Gste2* duplication in Burkina Faso is associated with the resistance-conferring I114T mutation

Ten of the 11 CNV alleles found in the *Gstu4*–*Gste3* cluster included *Gste2* (Fig. 2), perhaps reflecting the known importance of this gene in insecticide resistance. The well-characterized I114T mutation in *Gste2* is known to confer DDT resistance (Mitchell et al. 2014) and could be associated with gene duplications in a similar fashion to other mutations such as *Ace1* G119S. We therefore investigated whether any of the CNV alleles in *Gste2* were associated with this mutation. *Gste2*-114T is present across Africa and in both *An. gambiae* and *An. coluzzii* (The *Anopheles gambiae* 1000 Genomes Consortium 2017) but was only associated with Gstue_Dup1 in our data. Gstue_Dup1 was found in 16 *An. coluzzii* samples from Burkina Faso, all of which were at least heterozygote for 114T (Supplemental Table S5). The presence of 114T homozygotes, together with the ratio of reads supporting the I114 and 114T alleles in heterozygotes (roughly 1:2), indicate that both copies of *Gste2* in the Gstue_Dup1 CNV carry the 114T mutation.

### *Cyp6aa1* is more strongly associated with CNVs than *Cyp6p3*

Of the 15 CNV alleles found in the *Cyp6aa1*–*Cyp6p2* cluster, five included *Cyp6p3* but 13 included *Cyp6aa1* (Fig. 2B). *Cyp6p3* CNVs were found at high (>50%) frequency in one population (Côte d'Ivoire *An. coluzzii*: 90%), whereas *Cyp6aa1* CNVs were found at high frequency in *An. coluzzii* from Burkina Faso (91%), Côte d'Ivoire (89%) and Guinea (75%), and in *An. gambiae* from Uganda (64%).

## Discussion

Our study detected 1557 CNVs in 16 populations of *An. gambiae* and *An. coluzzii*, with CNVs being more abundant and larger in heterochromatin than in euchromatin. This parallels results in humans and rats, in which CNVs have been found to be particularly abundant in centromeres and telomeres (Nguyen et al. 2006; Guryev et al. 2008), typically composed of heterochromatin. The size distribution of CNVs was strongly right-skewed, with small CNVs being the most abundant in both euchromatin and heterochromatin, although we did not look for CNVs smaller than 1500 bp to avoid false discoveries. CNVs were biased toward gene-containing regions both overall and after excluding heterochromatic regions. This bias for genic regions has similarly been found in duplications in humans (Nguyen et al. 2006) and rats (Guryev et al. 2008), but not *Drosophila* (Schrider et al. 2016). Our results therefore indicate that abundance of CNVs around genes is not restricted to mammals.

The genes found in CNVs were themselves enriched for families involved in metabolic insecticide resistance. These results mirror findings in *Drosophila*, where cytochrome P450s were disproportionately represented in CNVs (Schrider et al. 2016). Similarly, in *Ae. aegypti*, cytochrome P450s were enriched among genes showing evidence of higher copy number in populations resistant to deltamethrin compared to susceptible populations (Faucon et al. 2015). In our study, the five metabolic genes most strongly associated with insecticide resistance in the literature for *An. gambiae* and *An. coluzzii*, and which have been shown to metabolize insecticides in vitro (*Gste2*, *Cyp6p3*, *Cyp6m2*, *Cyp6z1*, and *Cyp9k1*), were all found to be amplified in at least one population. Furthermore, three of these genes showed evidence of repeated CNV events within and between populations, with a total of 44 different CNVs across the five clusters, and as many as 16 in *Cyp9k1*. Of 44 CNVs, the majority (33) were tandem duplications, two were tandem inversions, and two (both in the *Gstu4*–*Gste3* cluster) showed evidence of association with known DNA transposons. The remaining seven could not be classified, but the presence of unmappable sequences around the breakpoints suggests that they may also be associated with repetitive elements.

Evidence for the importance of *Gste2* in insecticide resistance comes from studies showing its up-regulation in DDT resistant *An. gambiae* (Ding et al. 2003; David et al. 2005) and *An. funestus* (Riveron et al. 2014a) compared to susceptible mosquitoes, and from transgenic expression of *An. gambiae*/*An. funestus Gste2* in *Drosophila* providing resistance to DDT (Mitchell et al. 2014; Riveron et al. 2014a). Nonsynonymous SNPs in *Gste2* have also been shown to be associated with resistance to DDT in *An. funestus* (Riveron et al. 2014a) and to both DDT and deltamethrin in *An. gambiae* (Mitchell et al. 2014; Opondo et al. 2016). In our study, *Gste2* was amplified in Kenya, in *An. coluzzii* from Angola, Burkina Faso, and Ghana, and in *An. gambiae* from Gabon and Uganda.

*Cyp6p3* is up-regulated in mosquitoes resistant to pyrethroids, DDT, and bendiocarb (Djouaka et al. 2008; Müller et al. 2008; Fossog Tene et al. 2013; Kwiatkowska et al. 2013; Edi et al. 2014; Ngufor et al. 2015), metabolizes permethrin and deltamethrin (Müller et al. 2008), and provides resistance to pyrethroids when expressed in *Drosophila* (Edi et al. 2014). *Cyp6m2* is also up-regulated in mosquitoes with resistance to permethrin, DDT, and bendiocarb (Müller et al. 2007; Djouaka et al. 2008; Mitchell et al. 2012; Edi et al. 2014), metabolizes pyrethroids and DDT (Stevenson et al. 2011; Mitchell et al. 2012), and provides resistance to pyrethroids, DDT, and bendiocarb when expressed in *Drosophila* (Edi et al. 2014). In our study, *Cyp6p3* and *Cyp6m2* were found amplified primarily in *An. coluzzii* from Côte d'Ivoire, a population with known up-regulation of both *Cyp6p3* and *Cyp6m2* compared to susceptible populations (Edi et al. 2014). Particularly in the case of *Cyp6m2*, this up-regulation may not be driven solely by CNVs because the CNV frequency and copy number are not sufficient to explain the expression levels, but the selective pressure to up-regulate these genes may have played a part in maintaining these CNVs in the population.

*Cyp6z1* was amplified in *An gambiae* from Burkina Faso and Guinea. *Cyp6z1* is up-regulated in mosquitoes with resistance to pyrethroids and DDT (Nikou et al. 2003; David et al. 2005) and metabolizes DDT and carbaryl (Chiu et al. 2008). Finally, *Cyp9k1* was the most widely amplified gene of the five clusters that we studied, with CNVs found in more than half the populations in our data set. *Cyp9k1* is up-regulated in mosquitoes resistant to pyrethroids and DDT (Fossog Tene et al. 2013; Thomsen et al. 2014; Ngufor et al. 2015) and metabolizes deltamethrin (Vontas et al. 2018). Furthermore, a selective sweep in the *Cyp9k1* region has been associated with insecticide resistance in *An. coluzzii* (Main et al. 2015).

In-depth investigation of the CNVs around these five genes revealed strong evidence that they provide a selective advantage. First, some of the CNV alleles were found at high frequencies and across several populations. Second, the CNV alleles consistently showed evidence of being under positive selection as haplotype homozygosity was extended further for the CNVs than for wild-type haplotypes. Evidence for positive selection was also found in a CNV where the EHH score could not be calculated. Cyp9k1_Dup11, which exists as both duplications and triplications and thus could not be phased onto a haplotype scaffold for homozygosity calculation, was consistently found in the same samples as the haplotype of a large selective sweep around *Cyp9k1* in *An. gambiae* from Burkina Faso, Guinea, and Ghana, raising the strong possibility that this CNV is the focus of the selective sweep. Although Cyp9k1_Dup11 may be in linkage disequilibrium with other mutations, the high frequency of the triplicated version of Cyp9k1_Dup11 compared to the duplicated version, both of which are associated with the swept haplotype cluster, suggests that higher-order amplifications of *Cyp9k1* provide a selective advantage. The changes in allele frequencies in the different amplification levels in this CNV will need to be monitored to determine whether the triplication eventually replaces the duplication entirely.

A major current consideration for malaria control programs is the value of investing in next-generation bed nets that incorporate piperonyl butoxide (PBO), which interfere with cytochrome P450-mediated insecticide resistance, and which have been shown to be effective at reducing mosquito abundance and malaria incidence in at least some regions (Protopopoff et al. 2018). However, in most cases, it is not clear whether local insecticide resistance is cytochrome P450-mediated, partly due to the lack of molecular markers for identifying metabolic resistance. Our results highlight areas where mosquitoes are likely to exhibit cytochrome P450-based resistance. For example, CNVs in the *Cyp6aa/p* cluster were primarily found in *An. coluzzii* from Burkina Faso, Côte d'Ivoire, and Ghana, whereas CNVs in *Cyp9k1* were primarily found in *An. gambiae* from Burkina Faso, Ghana, and Guinea. Although the presence of cytochrome P450 CNVs indicates the likely presence of cytochrome P450-mediated resistance, we emphasize that their absence does not necessarily indicate that such resistance does not exist. Other forms of genetic variation that can increase gene expression, such as mutations in the regulatory region (Schmidt et al. 2010), need to be investigated to obtain a complete set of molecular markers for detecting metabolic resistance.

All but one of the 11 CNV alleles in the *Gstu/e* cluster included *Gste2*, indicating that this is the major target of gene amplification in this cluster. Given the body of evidence linking *Gste2* to DDT and pyrethroid resistance across multiple species—*An. gambiae* (Mitchell et al. 2014), *An. funestus* (Riveron et al. 2014a), *Aedes aegypti* (Lumjuan et al. 2011)—the focus of amplifications on this gene is likely to be linked to its importance in resistance.

The Gstue_Dup1 duplication in Burkina Faso occurs on the background of the *Gste2*_114T SNP, associated with DDT resistance in *An. gambiae* (Mitchell et al. 2014). The duplication may therefore serve to increase the dosage of *Gste2*, whose detoxifying activity has already been elevated by the 114T mutation. Alternatively, the role of Gstue_Dup1 may be to compensate for any negative fitness effects of 114T. Although impaired *Gste2* activity may be compensated by increasing the expression of the gene, Gstue_Dup1 is homogeneous for 114T, excluding the possibility of compensation by pairing of mutant and wild-type alleles as found in heterogeneous *Ace1* duplications (Assogba et al. 2015).

In the *Cyp6aa/p* cluster, only five of the 15 CNVs included *Cyp6p3*, and these were only found at appreciable frequency in *An. coluzzii* from Côte d'Ivoire. In contrast, 13 of the 15 CNVs included *Cyp6aa1*, with high CNV frequencies found in *An. coluzzii* from Burkina Faso, Côte d'Ivoire, and Guinea, and in *An. gambiae* from Uganda. Furthermore, the five high-frequency CNVs that include *Cyp6aa1* (Cyp6aap_Dup1, Cyp6aap_Dup7, Cyp6aap_ Dup10, Cyp6aap_Dup14, Cyp6aap_Dup15) all show evidence of positive selection. Although *Cyp6aa1* has received substantially less attention than *Cyp6p3*, it has previously been implicated in insecticide resistance. Expression of *Cyp6aa1* is higher in populations of *An. gambiae* and *An. coluzzii* that are resistant to pyrethroids and DDT compared to susceptible laboratory colonies (Kwiatkowska et al. 2013; Thomsen et al. 2014). There is also strong evidence for a link between *Cyp6aa1* and insecticide resistance in two congeneric species. In *An. funestus,* expression of *Cyp6aa1* is higher in mosquitoes that have survived permethrin exposure compared to a susceptible strain (Riveron et al. 2014b; Ibrahim et al. 2018), and the protein has been shown to metabolize pyrethroids and drive resistance when expressed in *Drosophila* (Ibrahim et al. 2018). In *An. minimus*, the ortholog of *Cyp6aa1* is up-regulated as a result of selection for resistance to deltamethrin (Rodpradit et al. 2005), and the protein has been shown to metabolize pyrethroids (Duangkaew et al. 2011). The ability of *An. gambiae Cyp6aa1* to metabolize insecticides has not been tested empirically, although theoretical modeling suggests that it should effectively bind to permethrin and deltamethrin (Ibrahim et al. 2018). The high frequency of amplifications in *Cyp6aa1* and the signals of selection associated with them suggest that the importance of this gene for insecticide resistance in *An. gambiae* and *An. coluzzii* has been underappreciated.

In conclusion, our results show a key role for CNVs in the adaptive response to strong and recent selective pressure. In populations of *Anopheles* mosquitoes across Africa, genes involved in metabolic resistance to insecticides have been duplicated, and these duplications have been driven to high frequencies by positive selection. These results highlight CNVs as a form of variation that can act as a rapid response to selective pressures requiring changes in expression levels. The widespread emergence of CNVs as a means of metabolic insecticide resistance reinforces the need to develop novel insecticidal compounds to tackle cross-resistance and highlights the potential value of PBO-treated bed nets. Our findings also highlight *Cyp6aa1* as a gene that should be more closely investigated for its importance in *An. gambiae*, having been so far overlooked in preference to its genomic neighbor *Cyp6p3*. More broadly, the focus on SNPs in *An. gambiae* insecticide resistance research has allowed the emergence and selective spread of copy number mutations in key insecticide resistance genes to go unnoticed. Our findings demonstrate the importance of surveillance and investigation of CNVs in these genes. To this end, the breakpoint descriptions provided in our study will allow these CNVs to be screened and monitored in mosquito populations, allowing the spread of these mutations to be tracked and providing the groundwork for future studies investigating their resistance profile.

## Methods

### Population sampling and whole-genome sequencing

We analyzed data from 1142 individual wild-caught specimens of *An. gambiae* and *An. coluzzii* collected and sequenced in Phase 2 of Ag1000G (https://www.malariagen.net/data/ag1000g-phase-2-ar1) (metadata provided in Supplemental Data S10). The specimens were collected from sites in 13 African countries (Angola *An. coluzzii n* = 78, Burkina Faso *An. coluzzii n* = 75, Burkina Faso *An. gambiae n* = 92, Cameroon *An. gambiae n* = 297, Côte d'Ivoire *An. coluzzii n* = 71, Equatorial Guinea (Bioko) *An. gambiae n* = 9, Gabon *An. gambiae n* = 69, Ghana *An. coluzzii n* = 55, Ghana *An. gambiae n* = 12, Guinea *An. coluzzii n* = 4, Guinea *An. gambiae n* = 40, Guinea-Bissau (mixed ancestry) *n* = 91, Kenya (undetermined ancestry) *n* = 48, Mayotte *An. gambiae n* = 24, The Gambia (mixed ancestry) *n* = 65, Uganda *An. gambiae n* = 112). Individual specimens were sequenced using the Illumina HiSeq platform to obtain 100-bp paired-end reads with a target coverage of 30×. Further details of population sampling, sample preparation, sequencing, alignment, species identification, and data production are reported elsewhere (The *Anopheles gambiae* 1000 Genomes Consortium 2017). Metadata for all samples, as well as detailed collection methods for populations not included in the aforementioned publication, are provided in Supplemental Methods SM1 and Supplemental Data S10.

### Calculation and normalization of coverage

For each individual, we used the *pysam* software package (https://github.com/pysam-developers/pysam) to count the number of aligned reads (coverage) in nonoverlapping 300-bp windows over the nuclear genome. The position of each read was considered to be its alignment start point; thus, each read was only counted once. Sequencing coverage can be biased by variation in local nucleotide composition. To account for this, we computed a normalized coverage from the read counts based on the expected coverage of each window given its GC content (Abyzov et al. 2011). For each 300-bp window we computed the percentage of (G + C) nucleotides to the nearest percentage point within the reference sequence and then divided the read counts in each window by the mean read count over all autosomal windows with the same (G + C) percentage. To minimize the impact of copy number variation when calculating these normalizing constants, we excluded windows from the calculation of mean read counts for which previous analyses of genome accessibility have found evidence for excessively high or low coverage or ambiguous alignment (windows with <90% accessible bases according to the Ag1000G Phase 2 genome accessibility map, referred to as “accessible windows”) (https://www.malariagen.net/data/ag1000g-phase-2-ar1). The normalized coverage values were then multiplied by a factor of 2, so that genome regions with a normal diploid copy number should have an expected normalized coverage of 2.

Before examining the normalized coverage data for evidence of copy number variation, we applied two filters to exclude windows for which coverage may be an unreliable indicator of copy number. The first filter removed windows in which >2% of reads were aligned with mapping quality 0 (Supplemental Fig. S13), which indicates that a read is mapped ambiguously and could be mapped equally well to a different genomic location. This filter removed 159,587 (20.8%) of 768,225 windows. The second filter removed windows for which the percentage (G + C) content was extreme and rarely represented within the accessible reference sequence, that is, fewer than 100 accessible windows with the same (G + C) percentage, because the small number of windows makes the calculation of a (G + C) normalizing constant unreliable. This filter removed 13,484 (2.2%) of the 608,638 remaining windows. Windows retained for analysis were referred to as “filtered windows.”

### Genome-wide copy number variation discovery

To detect the most likely copy number state (CNS) at each window in each individual, we applied a Gaussian HMM to the individual's normalized windowed coverage data, following a similar approach to Miles et al. (2016) and Leffler et al. (2017) (for details, see Supplemental Methods SM2). Because we are primarily interested in amplifications rather than deletions, we obtained a raw set of CNV calls for each sample by locating contiguous runs of at least five windows with amplified CNS (CNS > 2, or CNS > 1 for Chromosome X in males).

### CNV filtering

From the raw CNV call set, we created a quality-filtered list of CNV calls. We first removed samples with very high coverage variance, because high variance could lead to erratic CNV calls. We therefore removed 27 samples for which the variance in normalized coverage was greater than 0.2 (Supplemental Fig. S14), retaining 1115 samples for further analysis.

We then applied two filters to the raw CNV calls from these 1115 samples. For the first filter, we computed likelihoods for each raw CNV call for both the copy number state predicted by the HMM and for a null model of copy number = 2, and removed CNV calls for which the likelihood ratio was <1000 (Supplemental Methods SM3). For the second filter, we removed CNVs with low population frequencies. To do this, the raw CNV calls needed to be matched so that the same CNV in different individuals could be identified. We classed any two CNVs as identical if the breakpoints predicted by their copy number state transitions were within one window of each other. We then removed CNVs that were not found in at least 5% of individuals in at least one population (or at least three individuals for populations smaller than 40).

We determined the sensitivity and specificity of the CNV detection method using simulations in which coverage values were randomly shuffled across genomic windows. The details of these simulations are described in Supplemental Data S11.

### CNVs in chromatin types

Regions of heterochromatin and euchromatin were taken from Sharakhova et al. (2010). A CNV was considered to be heterochromatic if any part of it overlapped the heterochromatin regions. We performed simulations to determine whether the CNVs that we detected were evenly distributed between heterochromatin and euchromatin. For each run of the simulation, we randomly reallocated the start positions of every detected CNV, keeping the number of filtered windows covered by the CNVs unchanged, and calculated the number of CNVs that overlapped the heterochromatin. This simulation was run 10,000 times to obtain the distribution of the null model. Two-tailed *P*-values were obtained by calculating the proportion of simulations that produced at least as many heterochromatic CNVs as observed in the real data and multiplying this by two.

### Discovery of gene duplications and gene enrichment analysis

To determine the genes contained within each CNV, we compared the start and end points of the CNVs to the start and end points of all genes listed in the AgamP4.2 gene annotations (*Anopheles-gambiae-PEST_BASEFEATURES_AgamP4.2.gff3*). The start/end points of each CNV were calculated as the median of the start/end points of all the raw CNV calls that were matched to it. To keep only genes for which good coverage data were available, we retained only genes containing at least 50% filtered windows. We classed a retained gene as copied by a CNV if all the filtered windows within the gene were inside the CNV. To determine whether the CNVs that we detected contained more genes than expected by chance, we performed simulations as described for investigating enrichment for chromatin types, this time counting the number of CNVs that contained at least one gene. We also repeated these simulations after excluding the heterochromatic regions by only randomizing the euchromatic CNVs and preventing their randomized positions from being in the heterochromatin.

We identified genes that could potentially be involved in metabolic resistance through detoxification (“metabolic detox genes”) by finding genes whose annotations contained the terms “P450,” “glutathione S-transferase,” or “carboxylesterase” in the AgamP4 transcript annotations (*Anopheles-gambiae-PEST_ TRANSCRIPTS_AgamP4.2.fa*). We performed simulations to determine whether genes copied by CNVs were enriched for metabolic detox genes. For each run of the simulation, we randomized each genic CNV by reallocating the genes encompassed by the CNV, keeping the number of consecutive genes covered by each CNV unchanged, and calculated the number of CNVs that included at least one metabolic detox gene. This simulation was run 10,000 times to obtain the distribution of the null model. Two-tailed *P*-values were obtained by calculating the proportion of simulations that produced at least as many genic CNVs containing detox genes as observed in the real data and multiplying this by two.

GO term analysis of genes included in CNVs was performed using the *topGO* package in R (https://bioconductor.org/packages/release/bioc/html/topGO.html; R Core Team 2015). False discovery rates were calculated from the *P*-values using the R package *fdrtool* (https://cran.r-project.org/web/packages/fdrtool/index.html).

### Identifying CNV alleles at candidate metabolic insecticide resistance loci

We characterized in detail the different duplication events (CNV alleles) at five gene clusters of particular interest (*Cyp6aa1*–*Cyp6p2*, *Gstu4*–*Gste3*, *Cyp6m2*–*Cyp6m4*, *Cyp6z3*–*Cyp6z1*, *Cyp9k1*) using their unique patterns of discordant read pairs and reads crossing the CNV breakpoint (breakpoint reads, see Fig. 3; Supplemental Methods SM4). We manually inspected the five regions of interest in all 1142 samples to identify patterns of discordant and breakpoint reads (“diagnostic reads”) consistently associated with changes in coverage (Supplemental Figs. S2–S5). The start and end point of each CNV allele could usually be precisely determined by the breakpoint reads and was otherwise determined by discordant read pairs or the point of change in coverage (Supplemental Data S4–S7). Once the diagnostic reads were identified for a CNV allele, we recorded the presence of that allele in all samples with at least two supporting diagnostic reads.

To identify TEs that may be implicated in CNVs that did not appear to be tandem inversions or tandem duplications, we took the soft-clipped bases from the reads mapping to the breakpoints of these CNVs and used discontiguous megablast to compare them against the Repbase database (version 24.10) of *An. gambiae* transposable and repeat elements (www.girinst.org/repbase/ [Bao et al. 2015]).

### Detecting signals of selection on CNV alleles

We used the phased haplotypes to calculate the pairwise shared haplotype length and the EHH for each CNV allele (Sabeti et al. 2002), using only SNPs from outside the region in which CNVs were found. EHH calculations were performed using the *scikit-allel* Python package (https://zenodo.org/record/3238280).

Haplotype clusters in the *Cyp9k1* region were obtained with *scikit-allel*, using the first 1000 SNPs on the centromeric side of *Cyp9k1* (the telomeric side of this gene has low levels of accessibility). A distance matrix between haplotypes was computed using the proportion of accessible SNPs that differed between pairwise haplotype combinations. This was used to perform hierarchical clustering, with haplotype clusters defined using a cutoff of 0.001.

### Statistics

Statistical analysis was performed in R (R Core Team 2015). Contingency tables were analyzed with Fisher's exact test. Where the sample size was too large for the Fisher's exact test, *P*-values were obtained using the “simulated.p.value” option with 10^6^ replicates.

### Estimating allele-specific copy numbers and phasing CNV genotypes onto the Ag1000G haplotype scaffolds

To determine the allele-specific copy number in a sample, we estimated the change in coverage associated with each CNV allele (Supplemental Fig. S15; Supplemental Methods SM5). Thus, even when overlapping CNV alleles were present in a single sample, we were usually able to determine the number of copies of each allele.

For single-copy CNVs, it is possible to determine the genotype of a sample from the copy numbers (copy numbers of 1 indicate a heterozygote, copy numbers of 2 indicate a homozygote for the CNV). For higher-order CNVs, this is not possible because a heterozygote triplication cannot be distinguished from a homozygote duplication. We therefore applied two filters to retain only single-copy CNV alleles. The first filter removed CNV alleles for which the allele-specific copy number was found to rise above 2 in the data (if only a single sample rose as high as 2.5, we assumed that this could be an error and classed it as 2). This filter removed five CNV alleles (Cyp6aap_Dup11, Gstue_Dup2, Gstue_Dup8, Cyp9k1_Dup11, Cyp9k1_Dup15). For the second filter, we classed each sample as homozygote wild-type, heterozygote or homozygote CNV based on their copy numbers, and then removed CNV alleles that were inconsistent with Hardy-Weinberg expectations within the populations in which they were found. This filter removed four CNV alleles (Cyp6aap_Dup4, Gstue_Dup5, Gstue_Dup7, Cyp9k1_Dup10). Three CNV alleles (Cyp9k1_Dup7, Cyp9k1_Dup13, and Cyp9k1_Dup14) were also excluded because of difficulties in calling allele-specific copy number (Supplemental Data S7). In one case (Cyp6m_Dup1), all individuals were found to have a copy number of 2, indicating that the CNV is a triplication, with no duplications present in the population. This CNV was therefore retained, with all samples carrying the CNV classed as heterozygotic.

CNV alleles that passed both filters were phased onto the Ag1000G Phase 2 haplotype scaffolds using the MVNcall software v1.0 (Menelaou and Marchini 2013), using default parameters apart from setting *λ* = 0.1 to ensure that none of the input CNV genotype calls were changed during phasing. For each of the five gene clusters, phasing was performed using the 200 nonsingleton SNPs either side of the region in which CNVs were found, thus avoiding the inclusion of SNPs found inside any of the CNVs. Haplotypes that contained more than one CNV allele were rare and therefore excluded from subsequent calculations of haplotype homozygosity.

## Supplementary Material

Supplemental Material
