# Supplementary material for: Whole-genome sequencing reveals high complexity of copy number variation at insecticide resistance loci in malaria mosquitoes
Source: Genome Res. 2019 Aug;29(8):1250–61. doi: 10.1101/gr.245795.118 (PMC6673711; doi:10.1101/gr.245795.118)
Supplement: Supplemental Material [file supp_29_8_1250__index.html]

Whole-genome sequencing reveals high complexity of copy number variation at insecticide resistance loci in malaria mosquitoes — Supplemental Material 

# Whole-genome sequencing reveals high complexity of copy number variation at insecticide resistance loci in malaria mosquitoes

## Supplemental Material

- Supplementary\_Data\_S1.xls
- Supplementary\_Data\_S2.xls
- Supplementary\_Data\_S3.xls
- Supplementary\_Data\_S4.pdf
- Supplementary\_Data\_S5.pdf
- Supplementary\_Data\_S6.pdf
- Supplementary\_Data\_S7.pdf
- Supplementary\_Data\_S8.pdf
- Supplementary\_Data\_S9.xls
- Supplementary\_Data\_S10.csv
- Supplementary\_Data\_S11.pdf
- Supplementary\_Figures\_and\_tables.pdf
- Supplementary\_Methods.pdf
