# Supplementary material for: Whole-genome sequencing reveals high complexity of copy number variation at insecticide resistance loci in malaria mosquitoes
Source: Genome Res. 2019 Aug;29(8):1250–61. doi: 10.1101/gr.245795.118 (PMC6673711; doi:10.1101/gr.245795.118)
Supplement: Supplemental Material [file supp_gr.245795.118_Supplementary_Data_S4.pdf]

Electronic Supplementary Material S4

Description of CNV alleles detected in the CYP6AA - CYP6P region in Ag1000G phase 2.

Overview of all duplications in the CYP6AA - CYP6P region.

Fifteen CNVs were found that could be categorised according to their footprint of discordant reads or reads mapping to the CNV breakpoints. The regions covered by these CNVs are shown in Fig. **AA_S1**.

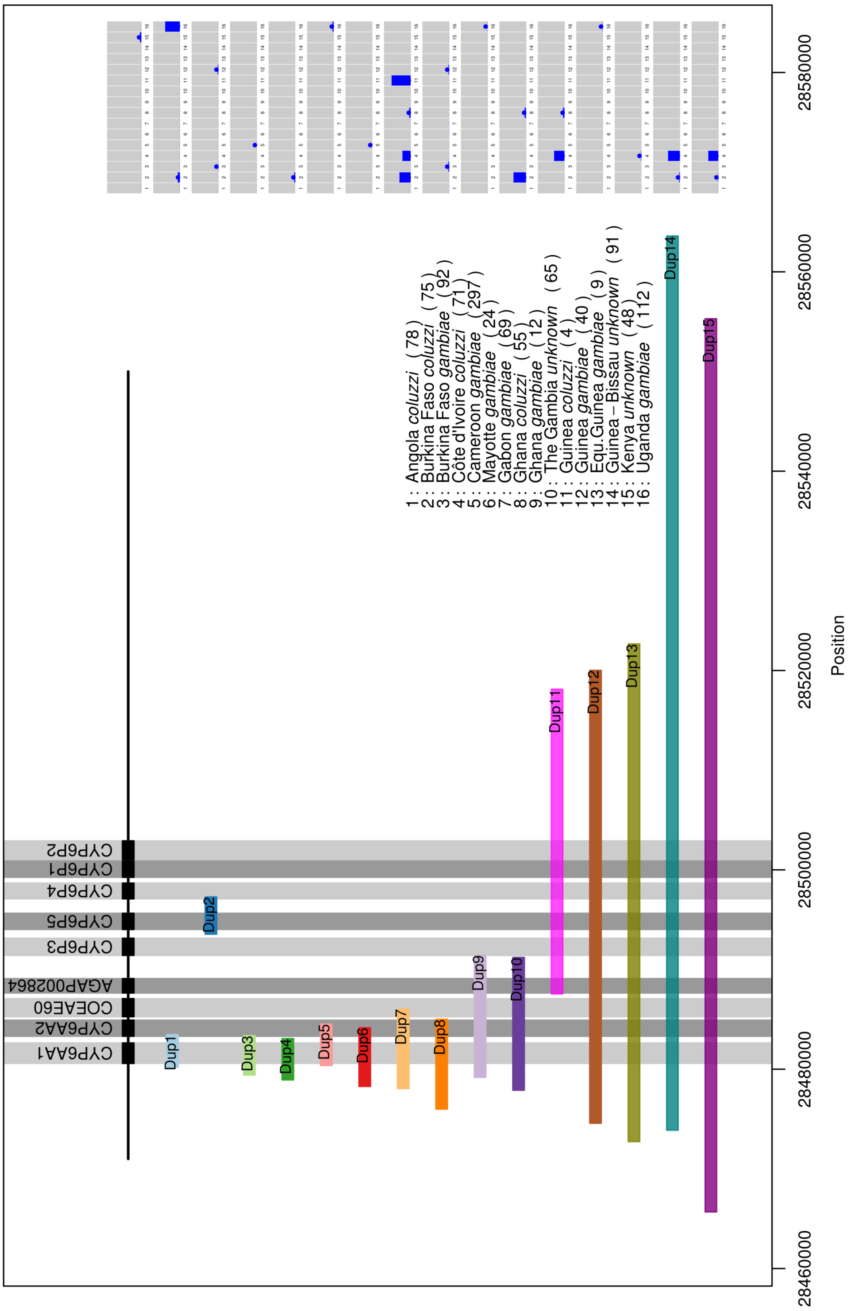

Fig. AA_S1: Overview of all the CNV alleles in the region ranging from CYP6AA1 to CYP6P2 present in Phase 2 of the Ag1000G dataset (alleles are named Cyp6aap_Dup*, abbreviated to Dup* in the plot). Position on chromosome 2R is shown on the X axis. Barplots on the right show the proportion of samples that carry a given CNV in each of the Phase 2 populations. Numbers below the barplots are numeric population IDs detailed to the left of the barplots (numbers in brackets indicate the total number of samples from that population). Blue points on the barplots indicate that at least one sample in this population carried the CNV. The top barplot (Cyp6aap_Dup0) shows CNVs that could not be categorised by discordant or breakpoint reads, each subsequent barplot represents the duplication shown to its left (Cyp6aap_Dup1 - Dup15).

Notes

Although 2 samples may carry CNVs that don't fit any of the these descriptions (Cyp6aap_Dup0 in Fig. **AA_S1**), these are probably not true CNVs since these two samples have very variable coverage.

It is worth nothing that the majority of duplications cover the CYP6AA1 gene, and such duplications are present in both *gambiae* and *coluzzi*. The duplications extending to the CYP6P3 are predominantly found in Côte d'Ivoire and almost exclusively present in *coluzzi* (the exception being Cyp6aap_Dup12, an exclusively Ugandan duplication present in only a single sample).

Cyp6aap_Dup1

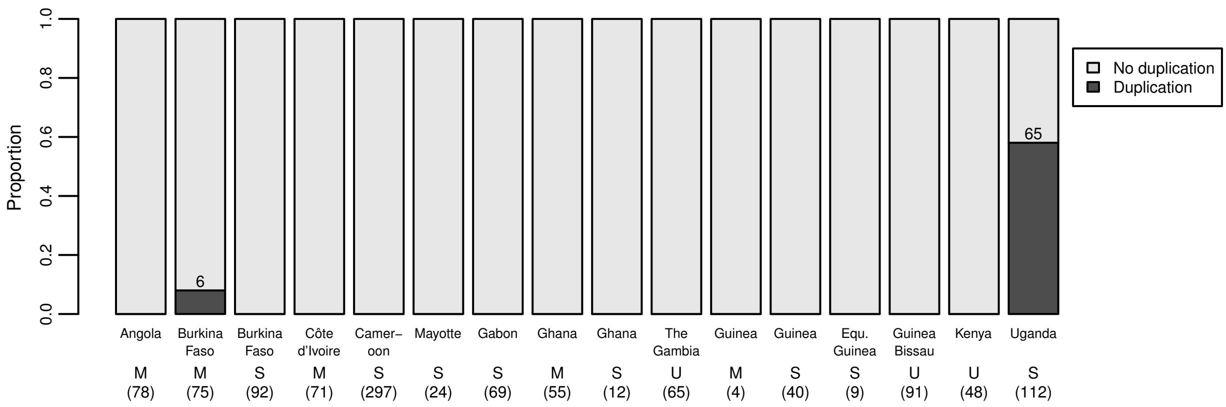

Fig. AA_S2: Barplot showing the proportion of samples that carry the Cyp6aap_Dup1 duplication in each of the Phase 2 populations. Numbers above the dark grey bars indicate the absolute number of samples carrying the duplication. S = *Anopheles gambiae*, M = *Anopheles coluzzi*, U = species undetermined. Numbers in brackets indicate the total number of samples from that population.

Cyp6aap_Dup1 was supported by face-away read pairs whose forward-facing read mapped in the interval 28480150 - 28480450 and whose reverse-facing read mapped in the interval 28483300 - 28483600 (Fig. **AA_S3**). Cyp6aap_Dup1 was also supported by reads soft-clipped at the breakpoints (positions 28480189 and 28483475), with the clipped bases at each breakpoint aligning at the other breakpoint. However, none of the samples from Burkina Faso were supported by these soft-clipped reads, raising the possibility that these samples may in fact carry a highly similar, but not identical duplication.

Cyp6aap_Dup1 breakpoint:

```
GAACCGCATGCCGATGC AATT AATTGTAATTTATTGCC
 end of the dup ^       ^ start of the dup
position 28483470       position 28480194
```

The `AATT` could sit on either side of the breakpoint.

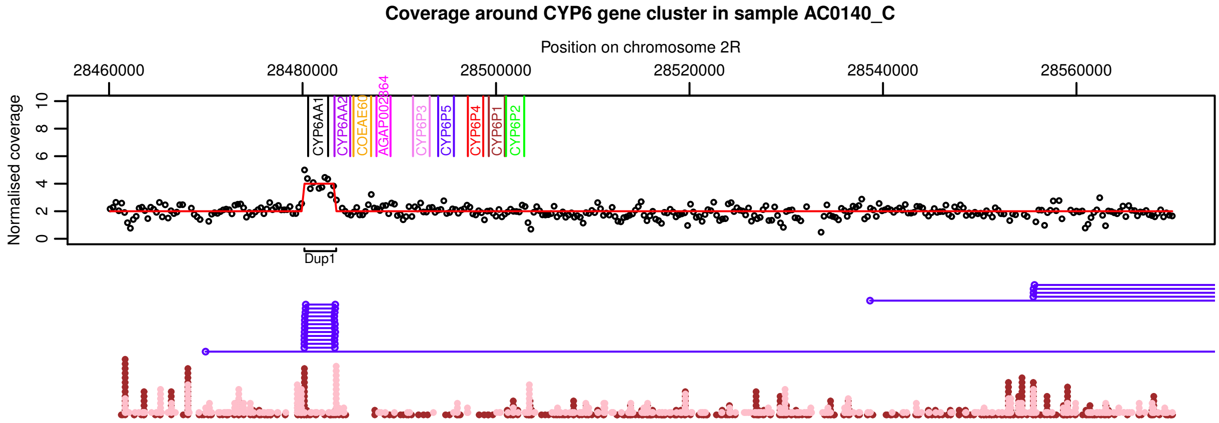

Fig. AA_S3: Example of coverage in an individual carrying the Cyp6aap_Dup1 duplication. Open black circles indicate coverage at each position. The red line shows the HMM estimation of the coverage state at each position. Vertical lines represent the positions of the various gene. Pairs of blue points connected by lines indicate pairs of face-away reads. Reads soft-clipped before the alignment start point (dark brown points) and after the alignment end point (light brown points) are present at the start and end points of the duplication (clipped at positions 28480189 and 28483475 respectively). In each case, the clipped bases align to the other end of the duplication, as expected.

The majority of samples with Cyp6aap_Dup1 were An. gambiae from Uganda (Table AA_S1.1). Estimates of copy number for Cyp6aap_Dup1 in this population indicated that only one sample had a copy number higher than 2. Assuming that samples a copy number of 1 are heterozygotes, and that samples with a copy number of 2 are homozygotes for Cyp6aap_Dup1 (Table AA_S1.2), the allele distribution is consistent with Hardy-Weinberg (HW) expectations ($P = 0.52$). The sample with an apparent copy number of 3 may represent a mis-calling of coverage, or a case of a rare triplication.

Table AA_S1.1: Copy number calls for Cyp6aap_Dup1. NAs were produced if coverage was too variable or if the duplication completely overlapped with another duplication whose coverage could also not be called.

copy number	AO col	BF col	BF gam	CI col	CM gam	FR gam	GA gam	GH col	GH gam	GM	GN col	GN gam	GQ gam	GW	KE	UG gam
NA	0	0	0	0	0	0	0	0	0	0	0	0	0	0	0	1
0	78	69	92	71	297	24	69	55	12	65	4	40	9	91	48	48
1	0	6	0	0	0	0	0	0	0	0	0	0	0	0	0	52
2	0	0	0	0	0	0	0	0	0	0	0	0	0	0	0	10
3	0	0	0	0	0	0	0	0	0	0	0	0	0	0	0	1

Table AA_S1.2: Copy number calls for all duplications in individuals that carry Cyp6aap_Dup1.

	Dup 0	Dup 1	Dup 2	Dup 3	Dup 4	Dup 5	Dup 6	Dup 7	Dup 8	Dup 9	Dup 10	Dup 11	Dup 12	Dup 13	Dup 14	Dup 15
AB0110_C	0	1	0	0	0	0	0	0	0	0	0	0	0	0	0	0
AB0188_C	0	1	0	0	0	0	0	0	0	0	0	0	0	0	0	0
AB0229_C	0	1	0	0	0	0	0	0	0	0	1	0	0	0	0	0
AB0242_C	0	1	0	0	0	0	0	0	0	0	0	0	0	0	0	0
AB0246_C	0	1	0	0	0	0	0	0	0	0	1	0	0	0	0	0
AB0282_C	0	1	0	0	0	0	0	0	0	0	0	0	0	0	0	0
AC0089_C	0	1	0	0	0	0	0	0	0	0	0	0	0	0	0	0
AC0092_C	0	2	0	0	0	0	0	0	0	0	0	0	0	0	0	0
AC0093_C	0	1	0	0	0	0	0	0	0	0	0	0	0	0	0	0
AC0095_C	0	2	0	0	0	0	0	0	0	0	0	0	0	0	0	0
AC0097_C	0	1	0	0	0	0	0	0	0	0	0	0	0	0	0	0
AC0098_C	0	1	0	0	0	0	0	0	0	0	0	0	0	0	0	0
AC0100_C	0	1	0	0	0	0	0	0	0	0	0	0	0	0	0	0
AC0102_C	0	1	0	0	0	0	0	0	0	0	0	0	0	0	0	0
AC0103_C	0	1	0	0	0	0	0	0	0	0	0	0	0	0	0	0
AC0104_C	0	1	0	0	0	0	0	0	0	0	0	0	0	0	0	0
AC0105_C	0	2	0	0	0	0	0	0	0	0	0	0	0	0	0	0
AC0108_C	0	1	0	0	0	0	0	0	0	0	0	0	0	0	0	0
AC0110_C	0	1	0	0	0	0	0	0	0	0	0	0	0	0	0	0
AC0111_C	0	1	0	0	0	0	0	0	0	0	0	0	0	0	0	0
AC0113_C	0	1	0	0	0	0	0	0	0	0	0	0	0	0	0	0
AC0114_C	0	1	0	0	0	0	0	0	0	0	0	0	0	0	0	0
AC0118_C	0	1	0	0	0	0	0	0	0	0	0	0	0	0	0	0
AC0120_C	0	1	0	0	0	0	0	0	0	0	0	0	0	0	0	0
AC0124_C	0	1	0	0	0	0	0	0	0	0	0	0	0	0	0	0
AC0125_C	0	2	0	0	0	0	0	0	0	0	0	0	0	0	0	0
AC0126_C	0	1	0	0	0	0	0	0	0	0	0	0	0	0	0	0
AC0129_C	0	1	0	0	0	0	0	0	0	0	0	0	0	0	0	0
AC0132_C	0	1	0	0	0	0	0	0	0	0	0	0	0	0	0	0
AC0135_C	0	1	0	0	0	0	0	0	0	0	0	0	0	0	0	0
AC0136_C	0	1	0	0	0	0	0	0	0	0	0	0	0	0	0	0
AC0138_C	0	1	0	0	0	0	0	0	0	0	0	0	0	0	0	0
AC0139_C	0	1	0	0	0	0	0	0	0	0	0	0	0	0	0	0
AC0140_C	0	2	0	0	0	0	0	0	0	0	0	0	0	0	0	0
AC0142_C	0	1	0	0	0	0	0	0	0	0	0	0	0	0	0	0
AC0143_C	0	1	0	0	0	0	0	0	0	0	0	0	0	0	0	0
AC0144_C	0	1	0	0	0	0	0	0	0	0	0	0	0	0	0	0
AC0145_C	0	1	0	0	0	0	0	0	0	0	0	0	0	0	0	0
AC0148_C	0	1	0	0	0	0	0	0	0	0	0	0	0	0	0	0
AC0149_C	0	1	0	0	0	0	0	0	0	0	0	0	0	0	0	0
AC0150_C	0	1	0	0	0	0	0	0	0	0	0	0	0	0	0	0
AC0151_C	0	1	0	0	0	0	0	0	0	0	0	0	0	0	0	0
AC0156_C	0	1	0	0	0	0	0	0	0	0	0	0	0	0	0	0
AC0160_C	0	1	0	0	0	0	0	0	0	0	0	0	0	0	0	0
AC0162_C	0	1	0	0	0	0	0	0	0	0	0	0	0	0	0	0
AC0163_C	0	1	0	0	0	0	0	0	0	0	0	0	0	0	0	0
AC0164_C	0	1	0	0	0	0	0	0	0	0	0	0	0	0	0	0
AC0166_C	0	2	0	0	0	0	0	0	0	0	0	0	0	0	0	0
AC0168_C	0	2	0	0	0	0	0	0	0	0	0	0	0	0	0	0
AC0169_C	0	1	0	0	0	0	0	0	0	0	0	0	0	0	0	0
AC0171_C	0	2	0	0	0	0	0	0	0	0	0	0	0	0	0	0
AC0172_C	0	1	0	0	0	0	0	0	0	0	0	0	0	0	0	0
AC0173_C	0	1	0	0	0	0	0	0	0	0	0	0	0	0	0	0
AC0174_C	0	1	0	0	0	0	0	0	0	0	0	0	0	0	0	0
AC0177_C	0	2	0	0	0	0	0	0	0	0	0	0	0	0	0	0
AC0180_C	0	0	0	0	0	0	0	0	0	0	0	0	0	0	0	0
AC0181_C	0	1	0	0	0	0	0	0	0	0	0	0	0	0	0	0
AC0183_C	0	1	0	0	0	0	0	0	0	0	0	0	0	0	0	0
AC0184_C	0	1	0	0	0	0	0	0	0	0	0	0	0	0	0	0
AC0187_C	0	2	0	0	0	0	0	0	0	0	0	0	0	0	0	0
AC0188_C	0	1	0	0	0	0	0	0	0	0	0	0	0	0	0	0
AC0189_C	0	1	0	0	0	0	0	0	0	0	0	0	0	0	0	0
AC0190_C	0	3	0	0	0	0	0	0	0	0	0	0	0	0	0	0
AC0191_C	0	1	0	0	0	0	0	0	0	0	0	0	0	0	0	0
AC0193_C	0	NA	0	0	0	0	0	0	0	0	0	0	0	0	0	0
AC0194_C	0	1	0	0	0	0	0	0	0	0	0	0	0	0	0	0
AC0195_C	0	1	0	0	0	0	0	0	0	0	0	0	0	0	0	0
AC0196_C	0	1	0	0	0	0	0	0	0	0	0	0	0	0	0	0
AC0197_C	0	1	0	0	0	0	0	0	0	0	0	0	0	0	0	0
AC0199_C	0	1	0	0	0	0	0	0	0	0	0	0	0	0	0	0
AC0203_C	0	1	0	0	0	0	0	0	0	0	0	0	0	0	0	0

Duplication type 2

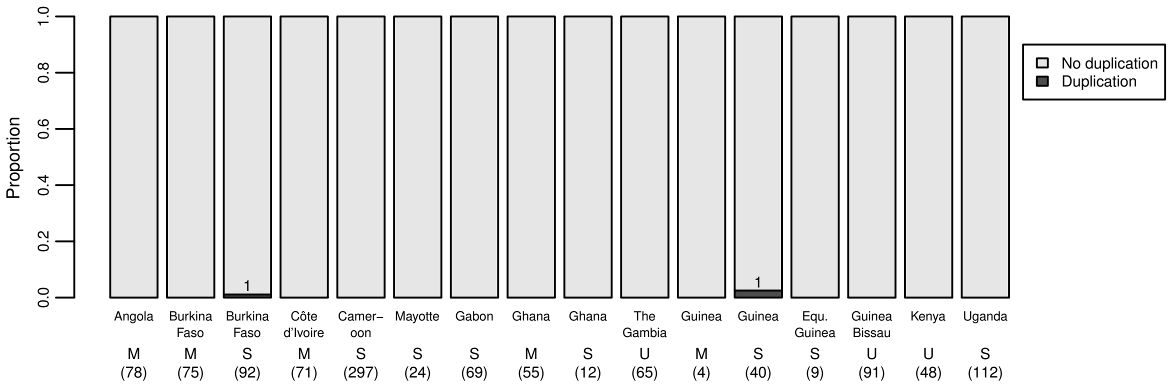

Fig. AA_S4: Barplot showing the proportion of samples that carry the Cyp6aap_Dup2 duplication in each of the Phase 2 populations. Numbers above the dark grey bars indicate the absolute number of samples carrying the duplication. S = *Anopheles gambiae*, M = *Anopheles coluzzi*, U = species undetermined. Numbers in brackets indicate the total number of samples from that population.

Cyp6aap_Dup2 was supported by face-away read pairs whose forward-facing read mapped in the interval 28493450 - 28493750 and whose reverse-facing read mapped in the interval 28497000 - 28497300 (Fig. **AA_S5**).Cyp6aap_Dup2 was also supported by reads soft-clipped at the breakpoints (positions 28493547 and 28497279), with the clipped bases at each breakpoint aligning at the other breakpoint.

Cyp6aap_Dup2 breakpoint:

```
ATGCGTGGCCCCTCGCCG AA TTTAATTATCCGCGACG
  end of the dup ^     ^ start of the dup
 position 28497276     position 28493550
```

The `AA` could sit on either side of the breakpoint.

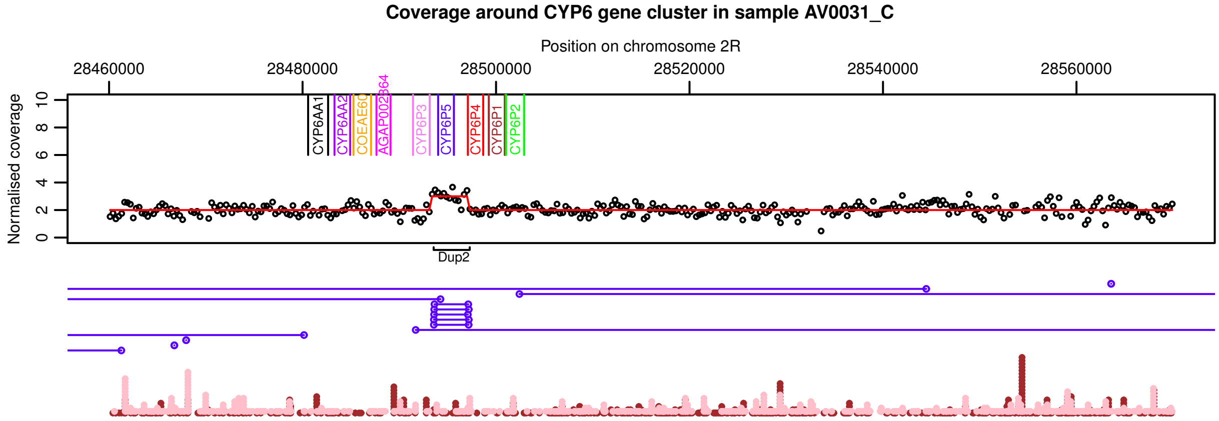

Fig. AA_S5: Coverage in the individual carrying the Cyp6aap_Dup2 duplication. Open black circles indicate coverage at each position. The red line shows the HMM estimation of the coverage state at each position. Vertical lines represent the positions of the various gene. Pairs of blue points connected by lines indicate pairs of face-away reads. Reads soft-clipped before the alignment start point (dark brown points) and after the alignment end point (light brown points) are present at the start and end points of the duplication (clipped at positions 28493547 and 28497279 respectively). In each case, the clipped bases align to the other end of the duplication, as expected.

The two samples that carry Cyp6aap_Dup2 both have a copy number of 1 (Tables AA_S2.1 & 2.2). Cyp6aap_Dup2 is therefore a single copy duplication and both samples are heterozygous.

Table AA_S2.1: Copy number calls for Cyp6aap_Dup2. NAs were produced if coverage was too variable or if the duplication completely overlapped with another duplication whose coverage could also not be called.

copy number	AO col	BF col	BF gam	CI col	CM gam	FR gam	GA gam	GH col	GH gam	GM	GN col	GN gam	GQ gam	GW	KE	UG gam
0	78	75	91	71	297	24	69	55	12	65	4	39	9	91	48	112
1	0	0	1	0	0	0	0	0	0	0	0	1	0	0	0	0

Table AA_S2.2: Copy number calls for all duplications in individuals that carry Cyp6aap_Dup2.

	Dup 0	Dup 1	Dup 2	Dup 3	Dup 4	Dup 5	Dup 6	Dup 7	Dup 8	Dup 9	Dup 10	Dup 11	Dup 12	Dup 13	Dup 14	Dup 15
AB0252_C	0	0	1	0	0	0	0	0	0	0	0	0	0	0	0	0
AV0031_C	0	0	1	0	0	0	0	0	0	0	0	0	0	0	0	0

Duplication type 3

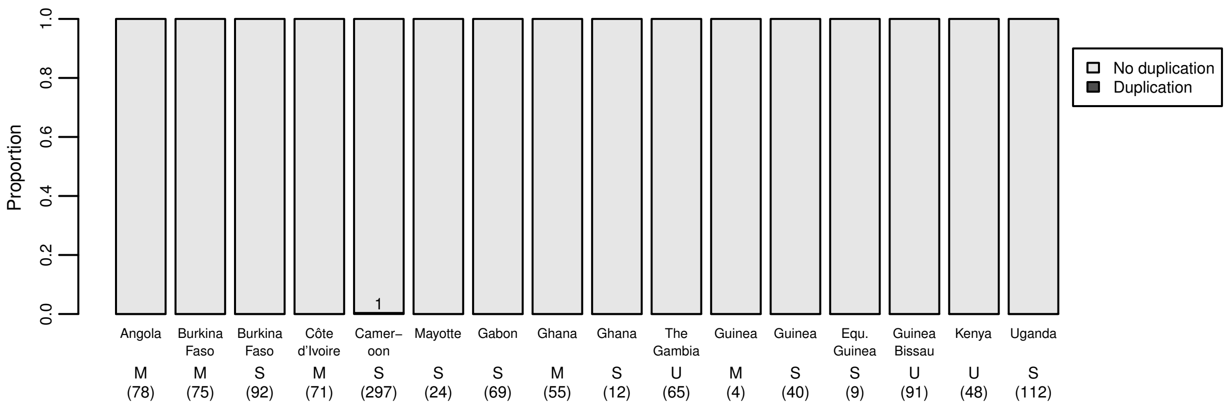

Fig. AA_S6: Barplot showing the proportion of samples that carry the Cyp6aap_Dup3 duplication in each of the Phase 2 populations. Numbers above the dark grey bars indicate the absolute number of samples carrying the duplication. S = *Anopheles gambiae*, M = *Anopheles coluzzi*, U = species undetermined. Numbers in brackets indicate the total number of samples from that population.

Cyp6aap_Dup3 was supported by face-away read pairs whose forward-facing read mapped in the interval 28479350 - 28479650 and whose reverse-facing read mapped in the interval 28483100 - 28483400 (Fig. AA_S7).Cyp6aap_Dup3 was also supported by reads soft-clipped at the breakpoints (positions 28479407 and 28483372), with the clipped bases at each breakpoint aligning at the other breakpoint.

Cyp6aap_Dup3 breakpoint:

```
GATCGCTTGGACATTCG CA CAAAGCGCGGGTGAAT
 end of the dup ^    ^ start of the dup
position 28483369    position 28479410
```

The `CA` could sit on either side of the breakpoint.

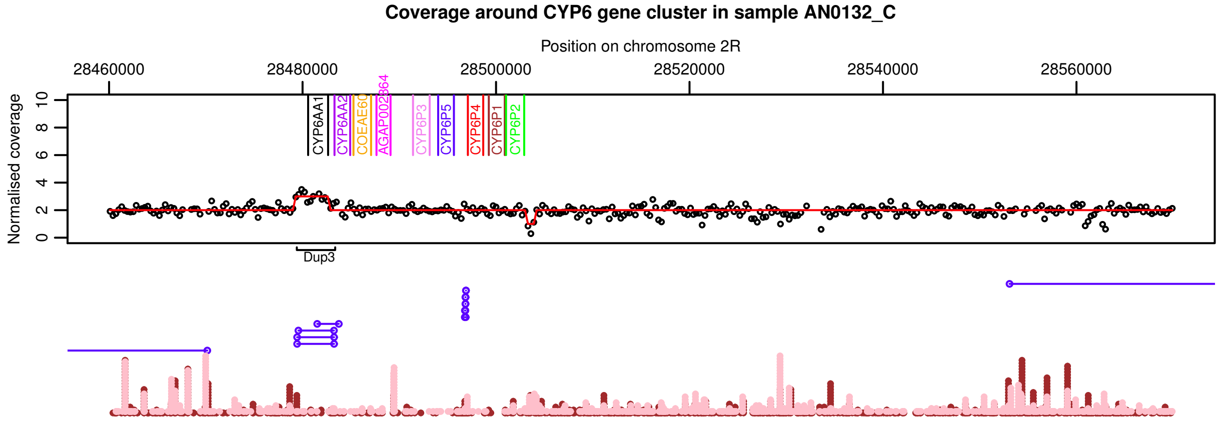

Fig. AA_S7: Coverage in the individual carrying the Cyp6aap_Dup3 duplication. Open black circles indicate coverage at each position. The red line shows the HMM estimation of the coverage state at each position. Vertical lines represent the positions of the various gene. Pairs of blue points connected by lines indicate pairs of face-away reads. Reads soft-clipped before the alignment start point (dark brown points) and after the alignment end point (light brown points) are present at the start and end points of the duplication (clipped at positions 28479407 and 28483372 respectively). In each case, the clipped bases align to the other end of the duplication, as expected.

The only sample that carries Cyp6aap_Dup3 has a copy number of 1 (Tables AA_S3.1 & 3.2). Cyp6aap_Dup3 is therefore a single copy duplication and the sample is heterozygous.

Table AA_S3.1: Copy number calls for Cyp6aap_Dup3. NAs were produced if coverage was too variable or if the duplication completely overlapped with another duplication whose coverage could also not be called.

copy number	AO col	BF col	BF gam	CI col	CM gam	FR gam	GA gam	GH col	GH gam	GM	GN col	GN gam	GQ gam	GW	KE	UG gam
0	78	75	92	71	296	24	69	55	12	65	4	40	9	91	48	112
1	0	0	0	0	1	0	0	0	0	0	0	0	0	0	0	0

Table AA_S3.2: Copy number calls for all duplications in individuals that carry Cyp6aap_Dup3.

	Dup 0	Dup 1	Dup 2	Dup 3	Dup 4	Dup 5	Dup 6	Dup 7	Dup 8	Dup 9	Dup 10	Dup 11	Dup 12	Dup 13	Dup 14	Dup 15
AN0132_C	0	0	0	1	0	0	0	0	0	0	0	0	0	0	0	0

Duplication type 4

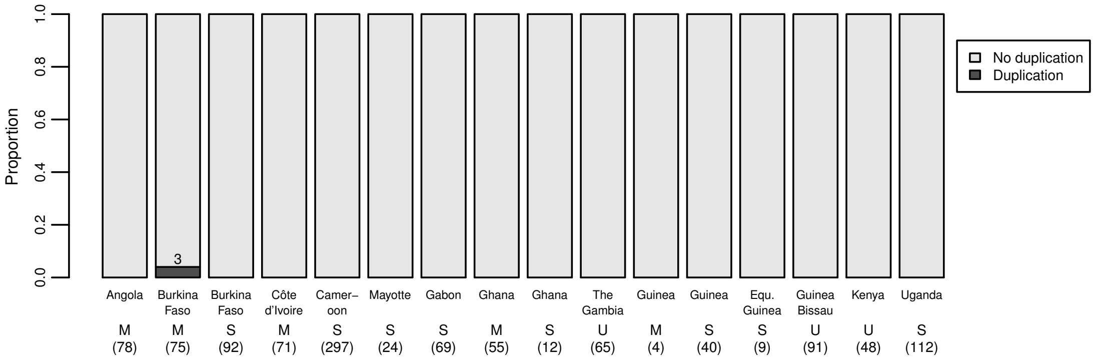

Fig. AA_S8: Barplot showing the proportion of samples that carry the Cyp6aap_Dup4 duplication in each of the Phase 2 populations. Numbers above the dark grey bars indicate the absolute number of samples carrying the duplication. S = *Anopheles gambiae*, M = *Anopheles coluzzi*, U = species undetermined. Numbers in brackets indicate the total number of samples from that population.

Cyp6aap_Dup4 was supported by face-away read pairs whose forward-facing read mapped in the interval 28478850 - 28479150 and whose reverse-facing read mapped in the interval 28482750 - 28483050 (Fig. **AA_S9**).Cyp6aap_Dup4 was also supported by reads soft-clipped at the breakpoints (positions 28478925 and 28483069), with the clipped bases at each breakpoint aligning at the other breakpoint.

Cyp6aap_Dup4 breakpoint:

```
GGCCACACCCAATTTCATGTT CATGTAATTCATGTTCATGTTTCAT GTAATTGATCAATTGTGCGTAATTA
    end of the dup ^                              ^ start of the dup
    position 28483068                             position 28478922
```

The sequence CATGTAATTCATGTTCATGTTTCAT is inserted between the sequences on either side of the breakpoint.

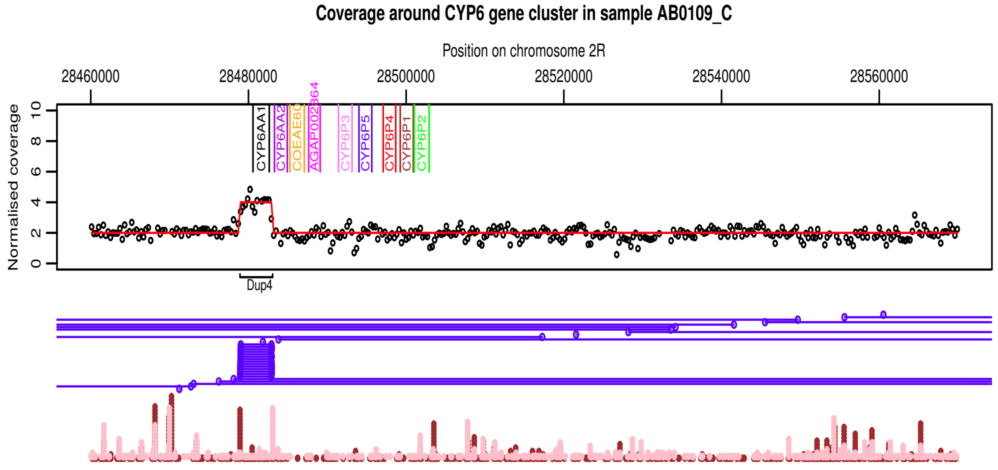

Fig. AA_S9: Example of coverage in an individual carrying the Cyp6aap_Dup4 duplication. Open black circles indicate coverage at each position. The red line shows the HMM estimation of the coverage state at each position. Vertical lines represent the positions of the various genes. Pairs of blue points connected by lines indicate pairs of face-away reads. Reads soft-clipped before the alignment start point (dark brown points) and after the alignment end point (light brown points) are present at the start and end points of the duplication (clipped at positions 28478925 and 28483069 respectively). In each case, the clipped bases align to the other end of the duplication, as expected.

Three samples carry Cyp6aap_Dup4, with copy numbers of 1, 1 and 2 respectively (Tables AA_S4.1 & 4.2). While this is just significantly different from HW expectations ($P = 0.04$), it is difficult to draw any firm conclusions from so few data.

Table AA_S4.1: Copy number calls for Cyp6aap_Dup4. NAs were produced if coverage was too variable or if the duplication completely overlapped with another duplication whose coverage could also not be called.

copy number	AO col	BF col	BF gam	CI col	CM gam	FR gam	GA gam	GH col	GH gam	GM	GN col	GN gam	GQ gam	GW	KE	UG gam
0	78	72	92	71	297	24	69	55	12	65	4	40	9	91	48	112
1	0	2	0	0	0	0	0	0	0	0	0	0	0	0	0	0
2	0	1	0	0	0	0	0	0	0	0	0	0	0	0	0	0

Table AA_S4.2: Copy number calls for all duplications individuals that carry Cyp6aap_Dup4.

	Dup 0	Dup 1	Dup 2	Dup 3	Dup 4	Dup 5	Dup 6	Dup 7	Dup 8	Dup 9	Dup 10	Dup 11	Dup 12	Dup 13	Dup 14	Dup 15
AB0109_C	0	0	0	0	2	0	0	0	0	0	0	0	0	0	0	0
AB0183_C	0	0	0	0	1	0	0	1	0	0	0	0	0	0	0	0
AB0257_C	0	0	0	0	1	0	0	0	0	0	0	0	0	0	0	0

Duplication type 5

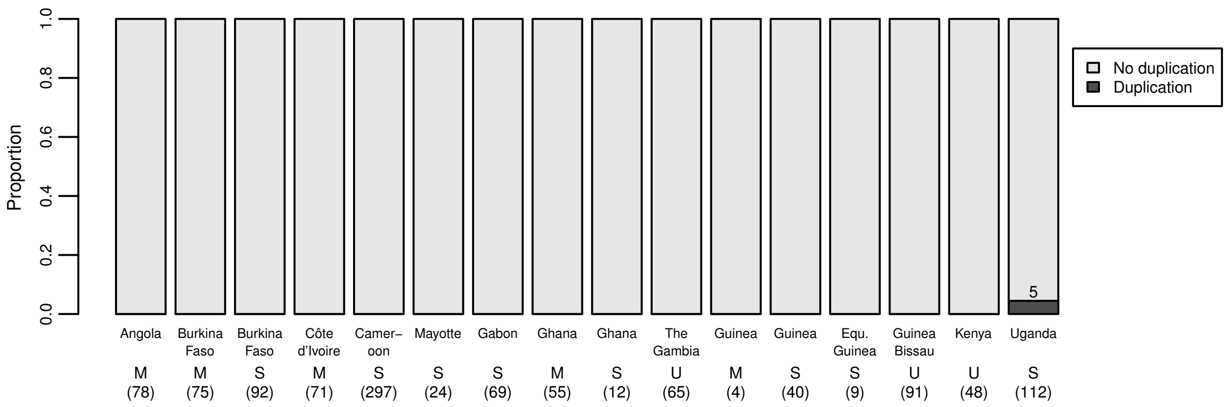

Fig. AA_S10: Barplot showing the proportion of samples that carry the Cyp6aap_Dup5 duplication in each of the Phase 2 populations. Numbers above the dark grey bars indicate the absolute number of samples carrying the duplication. S = *Anopheles gambiae*, M = *Anopheles coluzzi*, U = species undetermined. Numbers in brackets indicate the total number of samples from that population.

Cyp6aap_Dup5 was supported by face-away read pairs whose forward-facing read mapped in the interval 28480300 - 28480600 and whose reverse-facing read mapped in the interval 28484200 - 28484500 (Fig. AA_S11).Cyp6aap_Dup5 was also supported by reads soft-clipped at the breakpoints (positions 28480372 and 28484518), with the clipped bases at each breakpoint aligning at the other breakpoint.

Cyp6aap_Dup5 breakpoint:

```
GTTCGCCGCCGATCGAGAA  ACAAACGCTAAACGCTGAC
     end of the dup ^  ^ start of the dup
   position 28484517  position 28480373
```

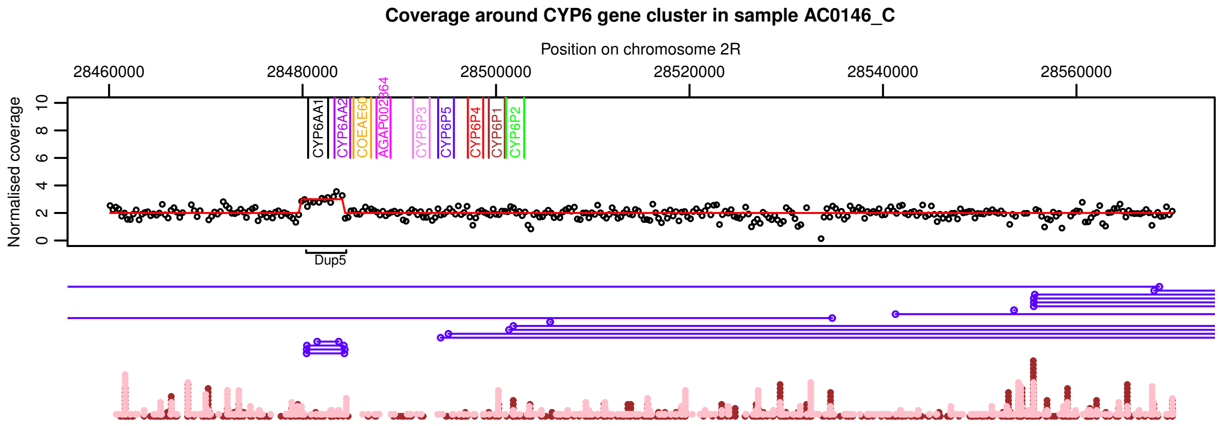

Fig. AA_S11: Example of coverage in an individual carrying the Cyp6aap_Dup5 duplication. Open black circles indicate coverage at each position. The red line shows the HMM estimation of the coverage state at each position. Vertical lines represent the positions of the various gene. Pairs of blue points connected by lines indicate pairs of face-away reads. Reads soft-clipped before the alignment start point (dark brown points) and after the alignment end point (light brown points) are present at the start and end points of the duplication (clipped at positions 28480372 and 28484518 respectively). In each case, the clipped bases align to the other end of the duplication, as expected.

The five samples that carry Cyp6aap_Dup5 all have a copy number of 1 (Tables AA_S5.1 & 5.2). Cyp6aap_Dup5 is therefore a single copy duplication and all samples are heterozygous.

Table AA_S5.1: Copy number calls for Cyp6aap_Dup5. NAs were produced if coverage was too variable or if the duplication completely overlapped with another duplication whose coverage could also not be called.

copy number	AO col	BF col	BF gam	CI col	CM gam	FR gam	GA gam	GH col	GH gam	GM	GN col	GN gam	GQ gam	GW	KE	UG gam
0	78	75	92	71	297	24	69	55	12	65	4	40	9	91	48	107
1	0	0	0	0	0	0	0	0	0	0	0	0	0	0	0	5

Table AA_S5.2: Copy number calls for all duplications in individuals that carry Cyp6aap_Dup5.

	Dup 0	Dup 1	Dup 2	Dup 3	Dup 4	Dup 5	Dup 6	Dup 7	Dup 8	Dup 9	Dup 10	Dup 11	Dup 12	Dup 13	Dup 14	Dup 15
AC0109_C	0	0	0	0	0	1	0	0	0	0	0	0	0	0	0	0
AC0146_C	0	0	0	0	0	1	0	0	0	0	0	0	0	0	0	0
AC0185_C	0	0	0	0	0	1	0	0	0	0	0	0	0	0	0	0
AC0186_C	0	0	0	0	0	1	0	0	0	0	0	0	0	0	0	0
AC0192_C	0	0	0	0	0	1	0	0	0	0	0	0	0	0	0	0

Duplication type 6

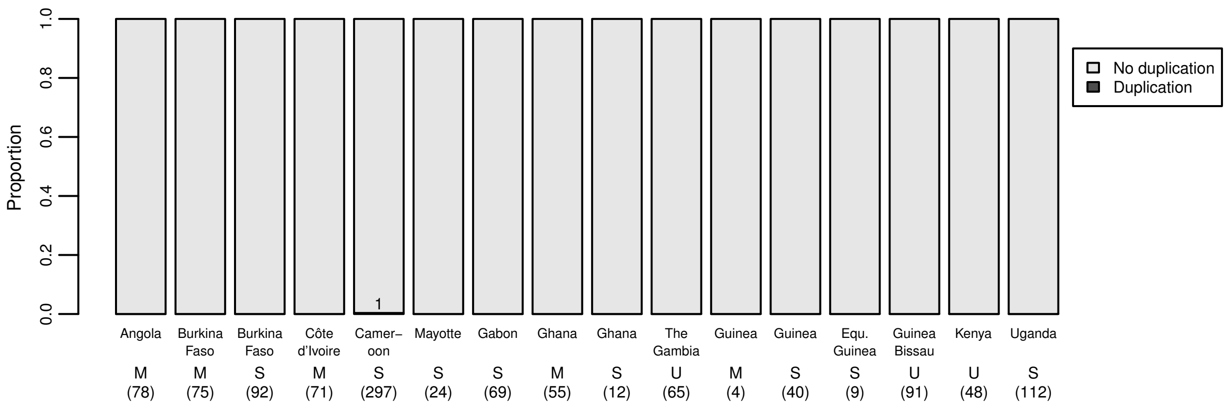

Fig. AA_S12: Barplot showing the proportion of samples that carry the Cyp6aap_Dup6 duplication in each of the Phase 2 populations. Numbers above the dark grey bars indicate the absolute number of samples carrying the duplication. S = *Anopheles gambiae*, M = *Anopheles coluzzi*, U = species undetermined. Numbers in brackets indicate the total number of samples from that population.

Cyp6aap_Dup6 was supported by face-away read pairs whose forward-facing read mapped in the interval 28478150 - 28478450 and whose reverse-facing read mapped in the interval 28483850 - 28484150 (**Fig. AA_S13**).Cyp6aap_Dup6 was also supported by reads soft-clipped at the breakpoints (positions 28478272 and 28484157), with the clipped bases at each breakpoint aligning at the other breakpoint.

Cyp6aap_Dup6 breakpoint:

```
GCCGACACATCACCGGGCA CTA GAAACCGTTACTACGCGATG
     end of the dup ^     ^ start of the dup
   position 28484156      position 28478273
```

The sequence `CTA` is inserted between the sequences on either side of the breakpoint.

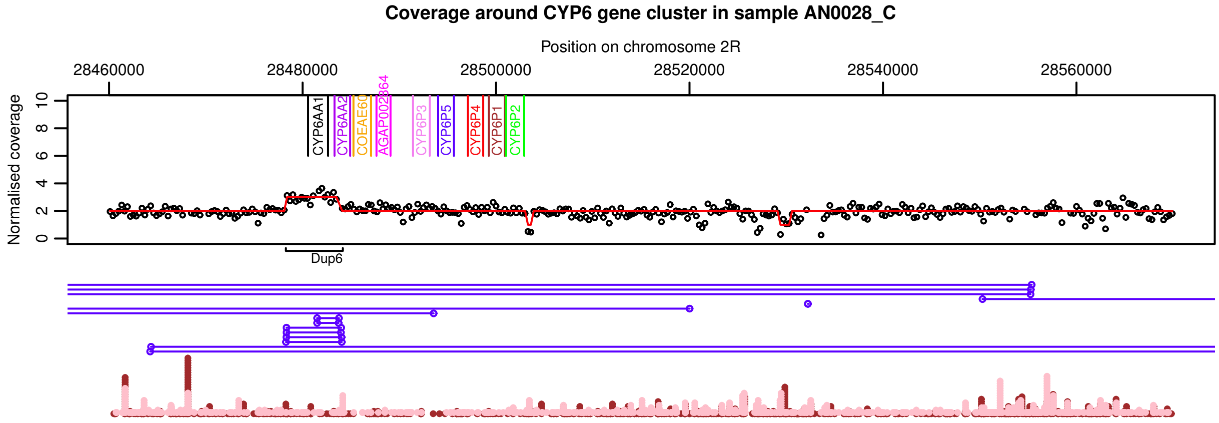

Fig. AA_S13: Coverage in the individual carrying the Cyp6aap_Dup6 duplication. Open black circles indicate coverage at each position. The red line shows the HMM estimation of the coverage state at each position. Vertical lines represent the positions of the various gene. Pairs of blue points connected by lines indicate pairs of face-away reads. Reads soft-clipped before the alignment start point (dark brown points) and after the alignment end point (light brown points) are present at the start and end points of the duplication (clipped at positions 28478272 and 28484157 respectively). In each case, the clipped bases align to the other end of the duplication, as expected.

The only sample that carries Cyp6aap_Dup6 has a copy number of 1 (Tables AA_S6.1 & 6.2). Cyp6aap_Dup6 is therefore a single copy duplication and the sample is heterozygous.

Table AA_S6.1: Copy number calls for Cyp6aap_Dup6. NAs were produced if coverage was too variable or if the duplication completely overlapped with another duplication whose coverage could also not be called.

copy number	AO col	BF col	BF gam	CI col	CM gam	FR gam	GA gam	GH col	GH gam	GM	GN col	GN gam	GQ gam	GW	KE	UG gam
0	78	75	92	71	296	24	69	55	12	65	4	40	9	91	48	112
1	0	0	0	0	1	0	0	0	0	0	0	0	0	0	0	0

Table AA_S6.2: Copy number calls for all duplications in individuals that carry Cyp6aap_Dup6.

	Dup 0	Dup 1	Dup 2	Dup 3	Dup 4	Dup 5	Dup 6	Dup 7	Dup 8	Dup 9	Dup 10	Dup 11	Dup 12	Dup 13	Dup 14	Dup 15
AN0028_C	0	0	0	0	0	0	1	0	0	0	0	0	0	0	0	0

Duplication type 7

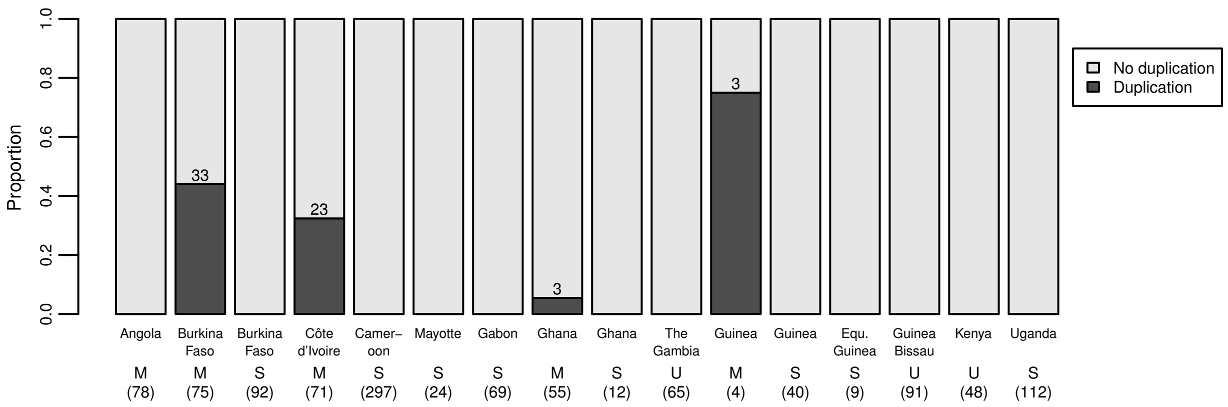

Fig. AA_S14: Barplot showing the proportion of samples that carry the Cyp6aap_Dup7 duplication in each of the Phase 2 populations. Numbers above the dark grey bars indicate the absolute number of samples carrying the duplication. S = *Anopheles gambiae*, M = *Anopheles coluzzi*, U = species undetermined. Numbers in brackets indicate the total number of samples from that population.

Cyp6aap_Dup7 was supported by same-strand read pairs mapping in the interval 28478000 - 28478300 and 28486000 - 28486300 respectively (Fig. **AA_S15**). Since this is an inverted repeat, the left-hand-side of the sequence below is the reverse complement of the sequence that is the beginning of the repeat (according to aligned genome position) but sits at the "end" of the repeat because it is inverted. Cyp6aap_Dup7 was also supported by reads soft-clipped at the breakpoints (positions 28478057 and 28486036), with the clipped bases at each breakpoint aligning at the other breakpoint.

Cyp6aap_Dup7 breakpoint:

```
GTTTGATTTTGCTTATCCTTTTT ATCGAC TCTCGATCAGTTCGATCGG
       end of the dup ^        ^ seq just after dup
      position 28478064        position 28486043
```

The ATCGAC could sit on either side of the breakpoint, but there is a mismatch if it sits left of position 28486043 (rather than left of 28476064).

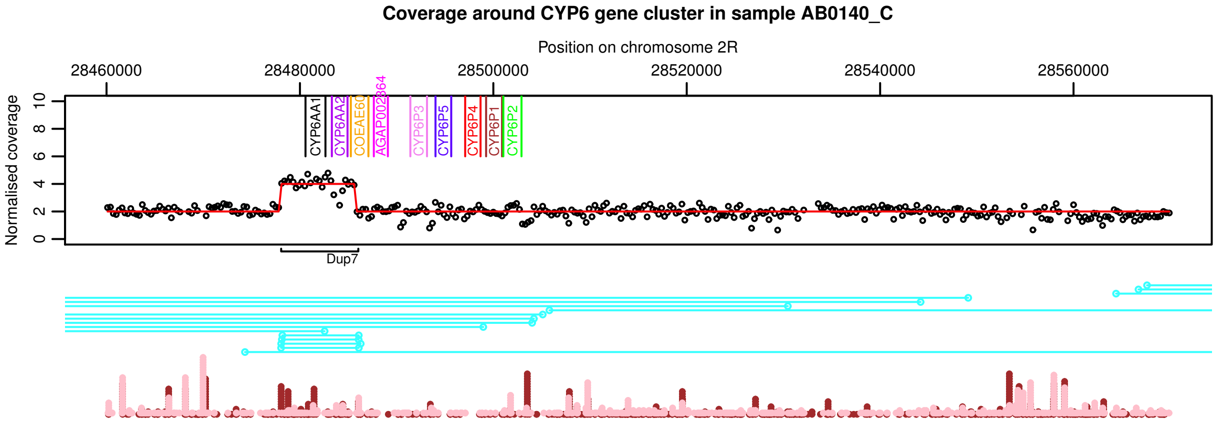

Fig. AA_S15: Example of coverage in an individual carrying the Cyp6aap_Dup7 duplication. Open black circles indicate coverage at each position. The red line shows the HMM estimation of the coverage state at each position. Vertical lines represent the positions of the various genes. Pairs of light blue points connected by lines indicate pairs of same-strand reads. Reads soft-clipped at the start of an the alignment (dark brown points) are present at the start and end points of the duplication (clipped at positions 28478057 and 28486036 respectively). In each case, the clipped bases align to the reverse complement of the other end of the duplication, as expected.

The majority of samples with Cyp6aap_Dup7 are in *An. coluzzii* from Burkina Faso and Côte d'ivoire (Table AA_S7.1). Estimates of copy number for Cyp6aap_Dup7 in these populations indicated that no sample had a copy number higher than 2. Assuming that samples with a copy number of 1 are heterozygotes and that samples with a copy number of 2 are homozygote for Cyp6aap_Dup7 (Table AA_S7.2), the allele distribution is consistent with HW expectations ($P = 0.09$ and $P = 0.19$ in Burkina Faso and Côte d'ivoire respectively).

Table AA_S7.1: Copy number calls for Cyp6aap_Dup7. NAs were produced if coverage was too variable or if the duplication completely overlapped with another duplication whose coverage could also not be called.

copy number	AO col	BF col	BF gam	CI col	CM gam	FR gam	GA gam	GH col	GH gam	GM	GN col	GN gam	GQ gam	GW	KE	UG gam
0	78	42	92	48	297	24	69	52	12	65	2	40	9	91	48	112
1	0	24	0	23	0	0	0	3	0	0	2	0	0	0	0	0
2	0	9	0	0	0	0	0	0	0	0	0	0	0	0	0	0

Table AA_S7.2: Copy number calls for all duplications in individuals that carry Cyp6aap_Dup7.

	Dup 0	Dup 1	Dup 2	Dup 3	Dup 4	Dup 5	Dup 6	Dup 7	Dup 8	Dup 9	Dup 10	Dup 11	Dup 12	Dup 13	Dup 14	Dup 15
AA0052_C	0	0	0	0	0	0	0	1	0	0	0	0	0	0	0	0
AA0066_C	0	0	0	0	0	0	0	1	0	0	0	0	0	0	0	0
AA0135_C	0	0	0	0	0	0	0	1	0	0	0	0	0	0	0	0
AB0088_C	0	0	0	0	0	0	0	2	0	0	0	0	0	0	0	0
AB0091_C	0	0	0	0	0	0	0	1	0	0	1	0	0	0	0	0
AB0098_C	0	0	0	0	0	0	0	1	0	0	0	0	0	0	0	0
AB0099_C	0	0	0	0	0	0	0	1	0	0	1	0	0	0	0	0
AB0100_C	0	0	0	0	0	0	0	1	0	0	1	0	0	0	0	0
AB0112_C	0	0	0	0	0	0	0	1	0	0	0	0	0	0	0	0
AB0114_C	0	0	0	0	0	0	0	1	0	0	0	0	0	0	0	0
AB0123_C	0	0	0	0	0	0	0	1	0	0	1	0	0	0	0	0
AB0124_C	0	0	0	0	0	0	0	1	0	0	0	0	0	0	0	0
AB0137_C	0	0	0	0	0	0	0	1	0	0	0	0	0	0	0	0
AB0140_C	0	0	0	0	0	0	0	2	0	0	0	0	0	0	0	0
AB0142_C	0	0	0	0	0	0	0	2	0	0	0	0	0	0	0	0
AB0181_C	0	0	0	0	0	0	0	1	0	0	1	0	0	0	0	0
AB0182_C	0	0	0	0	0	0	0	1	0	0	0	0	0	0	0	0
AB0183_C	0	0	0	0	1	0	0	1	0	0	0	0	0	0	0	0
AB0184_C	0	0	0	0	0	0	0	1	0	0	0	0	0	0	0	0
AB0185_C	0	0	0	0	0	0	0	2	0	0	0	0	0	0	0	0
AB0186_C	0	0	0	0	0	0	0	1	0	0	0	0	0	0	0	0
AB0189_C	0	0	0	0	0	0	0	2	0	0	0	0	0	0	0	0
AB0195_C	0	0	0	0	0	0	0	1	0	0	0	0	0	0	0	0
AB0204_C	0	0	0	0	0	0	0	1	0	0	1	0	0	0	0	0
AB0209_C	0	0	0	0	0	0	0	1	0	0	1	0	0	0	1	0
AB0212_C	0	0	0	0	0	0	0	2	0	0	0	0	0	0	0	0
AB0221_C	0	0	0	0	0	0	0	1	0	0	0	0	0	0	0	0
AB0224_C	0	0	0	0	0	0	0	1	0	0	0	0	0	0	0	0
AB0226_C	0	0	0	0	0	0	0	1	0	0	0	0	0	0	0	0
AB0237_C	0	0	0	0	0	0	0	1	0	0	1	0	0	0	0	0
AB0240_C	0	0	0	0	0	0	0	2	0	0	0	0	0	0	0	0
AB0248_C	0	0	0	0	0	0	0	1	0	0	0	0	0	0	0	0
AB0249_C	0	0	0	0	0	0	0	2	0	0	0	0	0	0	0	0
AB0263_C	0	0	0	0	0	0	0	1	0	0	0	0	0	0	0	0
AB0267_C	0	0	0	0	0	0	0	2	0	0	0	0	0	0	0	0
AB0276_C	0	0	0	0	0	0	0	1	0	0	1	0	0	0	0	0
AV0038_C	0	0	0	0	0	0	0	1	0	0	0	0	0	0	0	0
AV0040_C	0	0	0	0	0	0	0	1	0	0	0	0	0	0	0	0
AV0046_C	0	0	0	0	0	0	0	0	0	0	0	0	0	0	0	0
AY0006_C	0	0	0	0	0	0	0	1	0	0	0	2	0	0	0	0
AY0013_C	0	0	0	0	0	0	0	1	0	0	0	2	0	0	1	0
AY0018_C	0	0	0	0	0	0	0	1	0	0	0	0	0	0	0	1
AY0020_C	0	0	0	0	0	0	0	1	0	0	0	1	0	0	1	0
AY0023_C	0	0	0	0	0	0	0	1	0	0	0	3	0	0	0	1
AY0029_C	0	0	0	0	0	0	0	1	0	0	0	0	0	0	1	1
AY0031_C	0	0	0	0	0	0	0	1	0	0	0	0	0	0	0	0
AY0038_C	0	0	0	0	0	0	0	1	0	0	0	0	0	0	1	0
AY0039_C	0	0	0	0	0	0	0	1	0	0	0	2	0	1	0	1
AY0041_C	0	0	0	0	0	0	0	1	0	0	0	0	0	0	0	0
AY0042_C	0	0	0	0	0	0	0	1	0	0	0	0	0	0	0	0
AY0045_C	0	0	0	0	0	0	0	1	0	0	0	1	0	0	1	0
AY0047_C	0	0	0	0	0	0	0	1	0	0	0	0	0	0	0	0
AY0048_C	0	0	0	0	0	0	0	1	0	0	0	0	0	0	0	1
AY0055_C	0	0	0	0	0	0	0	1	0	0	0	2	0	0	0	0
AY0060_C	0	0	0	0	0	0	0	1	0	0	0	2	0	0	0	0
AY0062_C	0	0	0	0	0	0	0	1	0	0	0	0	0	0	0	1
AY0074_C	0	0	0	0	0	0	0	1	0	0	0	0	0	0	0	0
AY0076_C	0	0	0	0	0	0	0	1	0	0	0	2	0	0	2	0
AY0077_C	0	0	0	0	0	0	0	1	0	0	0	2	0	0	0	0
AY0079_C	0	0	0	0	0	0	0	1	0	0	0	0	0	0	0	1
AY0085_C	0	0	0	0	0	0	0	1	0	0	0	2	0	0	0	0
AY0088_C	0	0	0	0	0	0	0	1	0	0	0	2	0	0	0	0

Duplication type 8

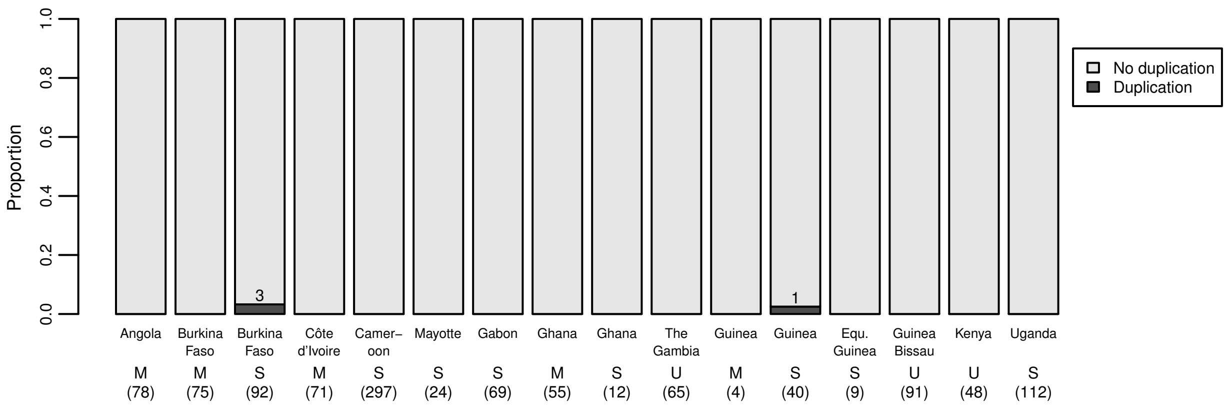

Fig. AA_S16: Barplot showing the proportion of samples that carry the Cyp6aap_Dup8 duplication in each of the Phase 2 populations. Numbers above the dark grey bars indicate the absolute number of samples carrying the duplication. S = *Anopheles gambiae*, M = *Anopheles coluzzi*, U = species undetermined. Numbers in brackets indicate the total number of samples from that population.

Cyp6aap_Dup8 was supported by face-away read pairs whose forward-facing read mapped in the interval 28475900 - 28476200 and whose reverse-facing read mapped in the interval 28484700 - 28485000 (**Fig. AA_S17**).Cyp6aap_Dup8 was also supported by reads soft-clipped at the breakpoints (positions 28475996 and 28485005), with the clipped bases at each breakpoint aligning at the other breakpoint.

Cyp6aap_Dup8 breakpoint:

```
AGTGCCCACCGTGAGCGA G CAAATTAACATTCAAC
  end of the dup ^   ^ start of the dup
 position 28485003   position 28475998
```

The G could sit on either side of the breakpoint.

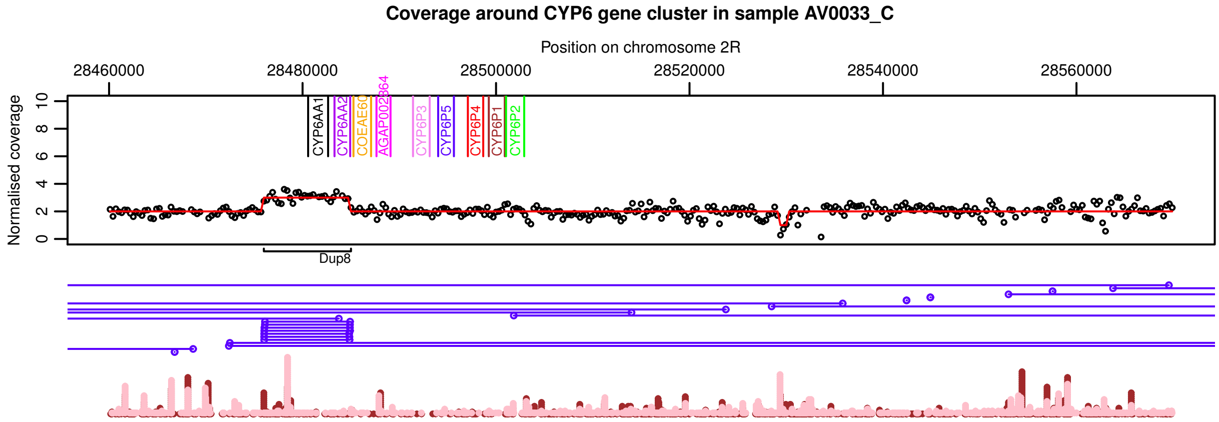

Fig. AA_S17: Example of coverage in an individual carrying the Cyp6aap_Dup8 duplication. Open black circles indicate coverage at each position. The red line shows the HMM estimation of the coverage state at each position. Vertical lines represent the positions of the various genes. Pairs of blue points connected by lines indicate pairs of face-away reads. Reads soft-clipped before the alignment start point (dark brown points) and after the alignment end point (light brown points) are present at the start and end points of the duplication (clipped at positions 28475996 and 28485005 respectively). In each case, the clipped bases align to the other end of the duplication, as expected.

The four samples that carry Cyp6aap_Dup8 all have a copy number of 1 (Tables AA_S8.1 & 8.2). Cyp6aap_Dup8 is therefore a single copy duplication and all samples are heterozygous.

Table AA_S8.1: Copy number calls for Cyp6aap_Dup8. NAs were produced if coverage was too variable or if the duplication completely overlapped with another duplication whose coverage could also not be called.

copy number	AO col	BF col	BF gam	CI col	CM gam	FR gam	GA gam	GH col	GH gam	GM	GN col	GN gam	GQ gam	GW	KE	UG gam
0	78	75	89	71	297	24	69	55	12	65	4	39	9	91	48	112
1	0	0	3	0	0	0	0	0	0	0	0	1	0	0	0	0

Table AA_S8.2: Copy number calls for all duplications in individuals that carry Cyp6aap_Dup8.

	Dup 0	Dup 1	Dup 2	Dup 3	Dup 4	Dup 5	Dup 6	Dup 7	Dup 8	Dup 9	Dup 10	Dup 11	Dup 12	Dup 13	Dup 14	Dup 15
AB0135_C	0	0	0	0	0	0	0	0	1	0	0	0	0	0	0	0
AB0203_C	0	0	0	0	0	0	0	0	1	0	0	0	0	0	0	0
AB0228_C	0	0	0	0	0	0	0	0	1	0	0	0	0	0	0	0
AV0033_C	0	0	0	0	0	0	0	0	1	0	0	0	0	0	0	0

Duplication type 9

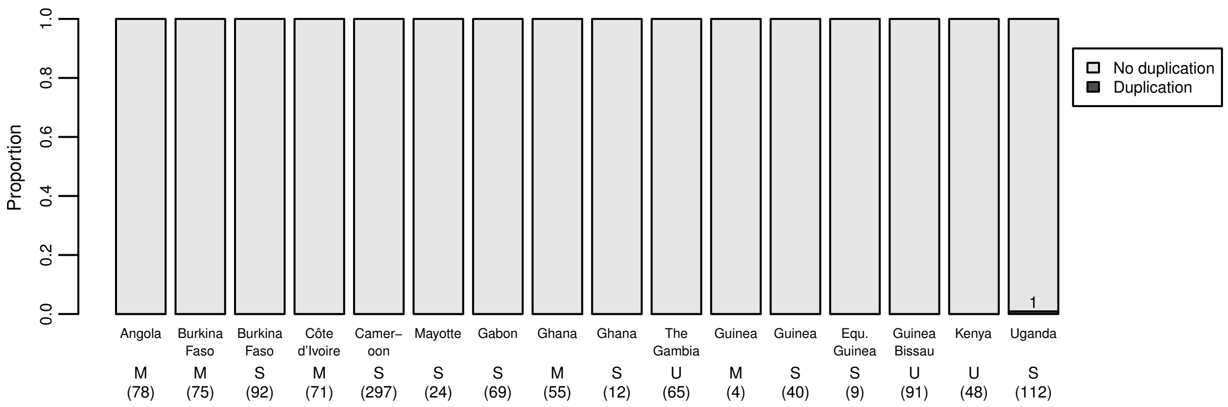

Fig. AA_S18: Barplot showing the proportion of samples that carry the Cyp6aap_Dup9 duplication in each of the Phase 2 populations. Numbers above the dark grey bars indicate the absolute number of samples carrying the duplication. S = *Anopheles gambiae*, M = *Anopheles coluzzi*, U = species undetermined. Numbers in brackets indicate the total number of samples from that population.

Cyp6aap_Dup9 was supported by face-away read pairs whose forward-facing read mapped in the interval 28479100 - 28479400 and whose reverse-facing read mapped in the interval 28491200 - 28491500 (Fig. **AA_S19**).Cyp6aap_Dup9 was also supported by reads soft-clipped at the breakpoints (positions 28479181 and 28491431), with the clipped bases at each breakpoint aligning at the other breakpoint.

Cyp6aap_Dup9 breakpoint:

```
CGTATCTTCTTTGTGCCT TGT GGGATGTTGAAGGAGAG
  end of the dup ^      ^ start of the dup
 position 28491430      position 28479182
```

The sequence TGT is inserted between the sequences on either side of the breakpoint.

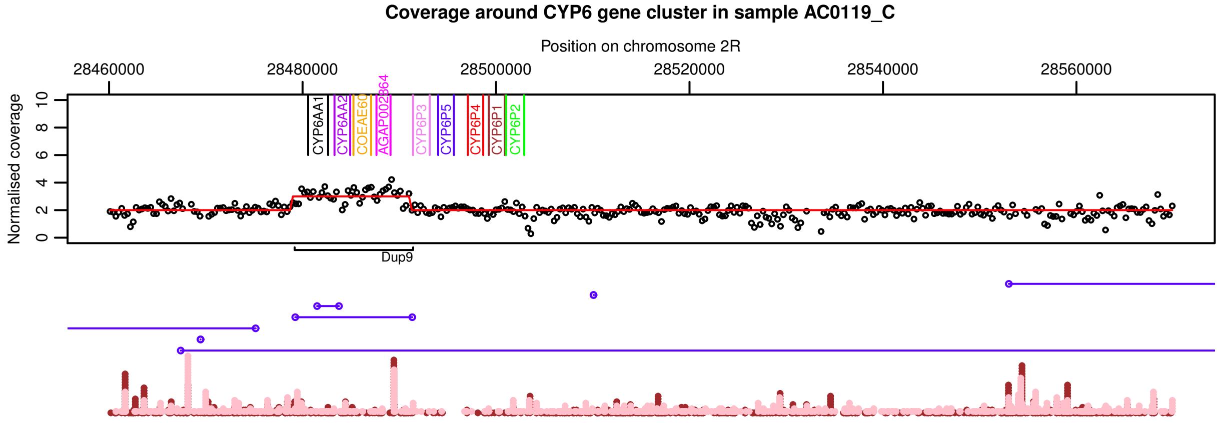

Fig. AA_S19: Coverage in the individual carrying the Cyp6aap_Dup9 duplication. Open black circles indicate coverage at each position. The red line shows the HMM estimation of the coverage state at each position. Vertical lines represent the positions of the various gene. Pairs of blue points connected by lines indicate pairs of face-away reads. Reads soft-clipped before the alignment start point (dark brown points) and after the alignment end point (light brown points) are present at the start and end points of the duplication (clipped at positions 28479181 and 28491431 respectively). In each case, the clipped bases align to the other end of the duplication, as expected.

The only sample that carries Cyp6aap_Dup9 has a copy number of 1 (Tables AA_S9.1 & 9.2). Cyp6aap_Dup9 is therefore a single copy duplication and the sample is heterozygous.

Table AA_S9.1: Copy number calls for Cyp6aap_Dup9. NAs were produced if coverage was too variable or if the duplication completely overlapped with another duplication whose coverage could also not be called.

copy number	AO col	BF col	BF gam	CI col	CM gam	FR gam	GA gam	GH col	GH gam	GM	GN col	GN gam	GQ gam	GW	KE	UG gam
0	78	75	92	71	297	24	69	55	12	65	4	40	9	91	48	111
1	0	0	0	0	0	0	0	0	0	0	0	0	0	0	0	1

Table AA_S9.2: Copy number calls for all duplications in individuals that carry Cyp6aap_Dup9.

	Dup 0	Dup 1	Dup 2	Dup 3	Dup 4	Dup 5	Dup 6	Dup 7	Dup 8	Dup 9	Dup 10	Dup 11	Dup 12	Dup 13	Dup 14	Dup 15
AC0119_C	0	0	0	0	0	0	0	0	0	1	0	0	0	0	0	0

Duplication type 10

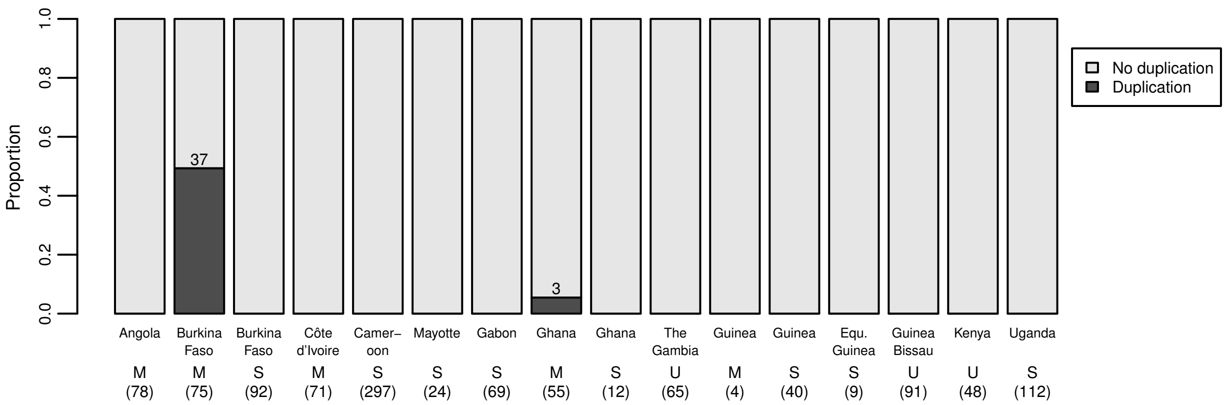

Fig. AA_S20: Barplot showing the proportion of samples that carry the Cyp6aap_Dup10 duplication in each of the Phase 2 populations. Numbers above the dark grey bars indicate the absolute number of samples carrying the duplication. S = *Anopheles gambiae*, M = *Anopheles coluzzi*, U = species undetermined. Numbers in brackets indicate the total number of samples from that population.

Cyp6aap_Dup10 was supported by face-away read pairs whose forward-facing read mapped in the interval 28477800 - 28478100 and whose reverse-facing read mapped in the interval 28490850 - 28491150 (Fig. **AA_S21**).Cyp6aap_Dup10 was also supported by reads soft-clipped at the breakpoints (positions 28477889 and 28491215), with the clipped bases at each breakpoint aligning at the other breakpoint.

Cyp6aap_Dup10 breakpoint:

```
GCAGATTCGCGCAGATGT G AACTTTATGATACTTCCG
  end of the dup ^    ^ start of the dup
 position 28491213    position 28477891
```

The G could sit on either side of the breakpoint.

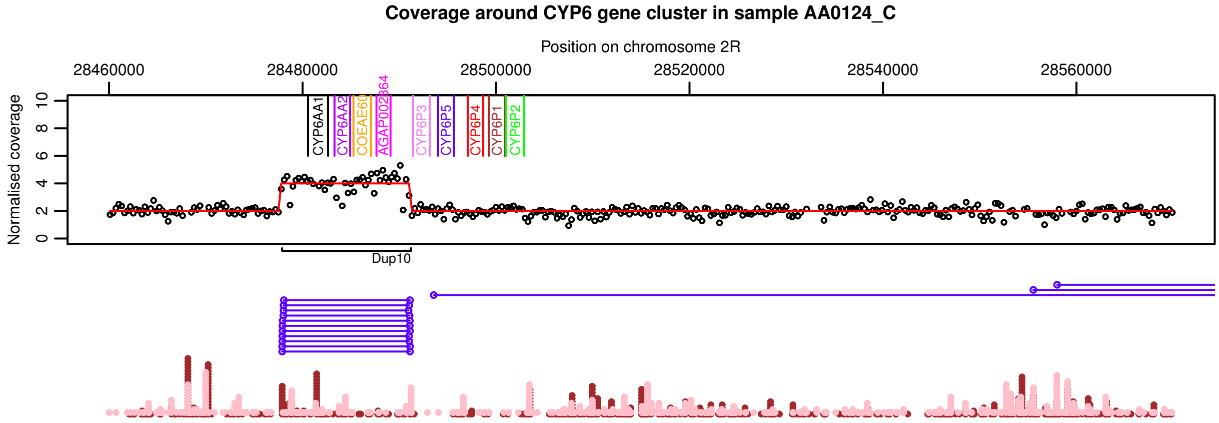

Fig. AA_S21: Example of coverage in an individual carrying the Cyp6aap_Dup10 duplication. Open black circles indicate coverage at each position. The red line shows the HMM estimation of the coverage state at each position. Vertical lines represent the positions of the various genes. Pairs of blue points connected by lines indicate pairs of face-away reads. Reads soft-clipped before the alignment start point (dark brown points) and after the alignment end point (light brown points) are present at the start and end points of the duplication (clipped at positions 28477889 and 28491215 respectively). In each case, the clipped bases align to the other end of the duplication, as expected.

The majority of samples with Cyp6aap_Dup10 are in *An. coluzzii* from Burkina Faso (Table AA_S10.1) Estimates of copy number for Cyp6aap_Dup10 in this population indicated that no sample has a copy number higher than 4. Assuming that samples with a copy number of 1 are heterozygotes and that samples with a copy number of 2 are homozygotes for Cyp6aap_Dup10 (Table AA_S10.2), the allele distribution is consistent with HW expectations ($P = 0.58$).

Table AA_S10.1: Copy number calls for Cyp6aap_Dup10. NAs were produced if coverage was too variable or if the duplication completely overlapped with another duplication whose coverage could also not be called.

copy number	AO col	BF col	BF gam	CI col	CM gam	FR gam	GA gam	GH col	GH gam	GM	GN col	GN gam	GQ gam	GW	KE	UG gam
0	78	38	92	71	297	24	69	52	12	65	4	40	9	91	48	112
1	0	29	0	0	0	0	0	2	0	0	0	0	0	0	0	0
2	0	8	0	0	0	0	0	1	0	0	0	0	0	0	0	0

Table AA_S10.2: Copy number calls for all duplications in individuals that carry Cyp6aap_Dup10.

	Dup 0	Dup 1	Dup 2	Dup 3	Dup 4	Dup 5	Dup 6	Dup 7	Dup 8	Dup 9	Dup 10	Dup 11	Dup 12	Dup 13	Dup 14	Dup 15
AA0043_C	0	0	0	0	0	0	0	0	0	0	1	0	0	0	0	0
AA0088_C	0	0	0	0	0	0	0	0	0	0	1	0	0	0	0	0
AA0124_C	0	0	0	0	0	0	0	0	0	0	2	0	0	0	0	0
AB0087_C	0	0	0	0	0	0	0	0	0	0	2	0	0	0	0	0
AB0090_C	0	0	0	0	0	0	0	0	0	0	1	0	0	0	1	0
AB0091_C	0	0	0	0	0	0	0	1	0	0	1	0	0	0	0	0
AB0094_C	0	0	0	0	0	0	0	0	0	0	1	0	0	0	0	0
AB0097_C	0	0	0	0	0	0	0	0	0	0	2	0	0	0	0	0
AB0099_C	0	0	0	0	0	0	0	1	0	0	1	0	0	0	0	0
AB0100_C	0	0	0	0	0	0	0	1	0	0	1	0	0	0	0	0
AB0111_C	0	0	0	0	0	0	0	0	0	0	2	0	0	0	0	0
AB0115_C	0	0	0	0	0	0	0	0	0	0	2	0	0	0	0	0
AB0122_C	0	0	0	0	0	0	0	0	0	0	2	0	0	0	0	0
AB0123_C	0	0	0	0	0	0	0	1	0	0	1	0	0	0	0	0
AB0138_C	0	0	0	0	0	0	0	0	0	0	1	0	0	0	0	0
AB0139_C	0	0	0	0	0	0	0	0	0	0	1	0	0	0	0	0
AB0181_C	0	0	0	0	0	0	0	1	0	0	1	0	0	0	0	0
AB0187_C	0	0	0	0	0	0	0	0	0	0	1	0	0	0	0	0
AB0190_C	0	0	0	0	0	0	0	0	0	0	2	0	0	0	0	0
AB0191_C	0	0	0	0	0	0	0	0	0	0	1	0	0	0	0	0
AB0192_C	0	0	0	0	0	0	0	0	0	0	1	0	0	0	0	0
AB0196_C	0	0	0	0	0	0	0	0	0	0	1	0	0	0	0	0
AB0204_C	0	0	0	0	0	0	0	1	0	0	1	0	0	0	0	0
AB0209_C	0	0	0	0	0	0	0	1	0	0	1	0	0	0	1	0
AB0213_C	0	0	0	0	0	0	0	0	0	0	2	0	0	0	0	0
AB0219_C	0	0	0	0	0	0	0	0	0	0	1	0	0	0	0	0
AB0222_C	0	0	0	0	0	0	0	0	0	0	1	0	0	0	0	0
AB0223_C	0	0	0	0	0	0	0	0	0	0	1	0	0	0	0	0
AB0229_C	0	1	0	0	0	0	0	0	0	0	1	0	0	0	0	0
AB0234_C	0	0	0	0	0	0	0	0	0	0	2	0	0	0	0	0
AB0237_C	0	0	0	0	0	0	0	1	0	0	1	0	0	0	0	0
AB0243_C	0	0	0	0	0	0	0	0	0	0	1	0	0	0	0	0
AB0246_C	0	1	0	0	0	0	0	0	0	0	1	0	0	0	0	0
AB0247_C	0	0	0	0	0	0	0	0	0	0	1	0	0	0	0	0
AB0250_C	0	0	0	0	0	0	0	0	0	0	1	0	0	0	0	0
AB0258_C	0	0	0	0	0	0	0	0	0	0	1	0	0	0	0	0
AB0262_C	0	0	0	0	0	0	0	0	0	0	1	0	0	0	0	0
AB0266_C	0	0	0	0	0	0	0	0	0	0	1	0	0	0	0	0
AB0276_C	0	0	0	0	0	0	0	1	0	0	1	0	0	0	0	0
AB0279_C	0	0	0	0	0	0	0	0	0	0	1	0	0	0	0	0

Duplication type 11

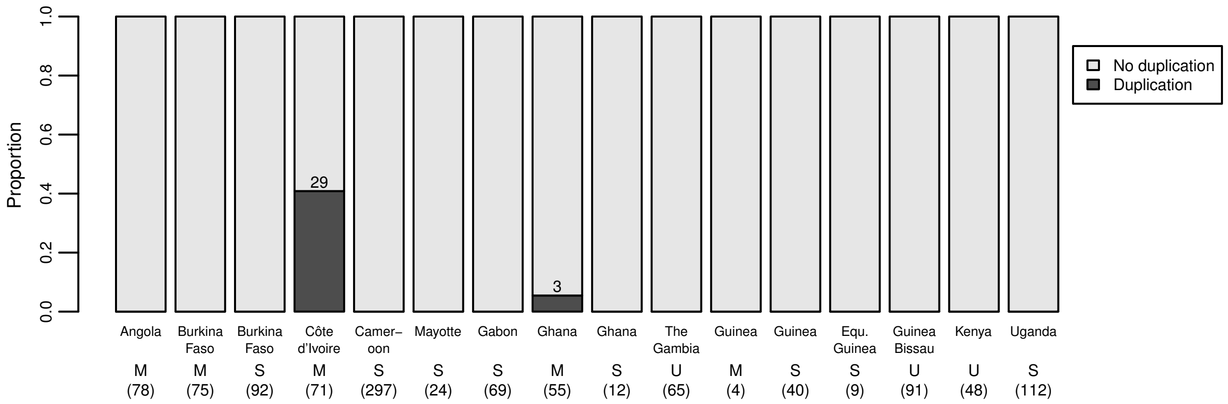

Fig. AA_S22: Barplot showing the proportion of samples that carry the Cyp6aap_Dup11 duplication in each of the Phase 2 populations. Numbers above the dark grey bars indicate the absolute number of samples carrying the duplication. S = *Anopheles gambiae*, M = *Anopheles coluzzi*, U = species undetermined. Numbers in brackets indicate the total number of samples from that population.

Cyp6aap_Dup11 was supported by face-away read pairs whose forward-facing read mapped in the interval 28487450 - 28487750 and whose reverse-facing read mapped in the interval 28517800 - 28518100 (Fig. Fig. **AA_S23**). One sample Cyp6aap_Dup11 was also supported by reads soft-clipped at the breakpoints (positions 28487546 and 28518123), with the clipped bases at each breakpoint aligning at the other breakpoint.did not have the face-away reads, but Cyp6aap_Dup11 could nevertheless be identified by the soft-clipped reads at the breakpoints.

Cyp6aap_Dup11 breakpoint:

```
GTTTACACTACACACAA  TTGCACG  TTATCTATTCCACTGCCAA
 end of the dup ^            ^ start of the dup
position 28518115           position 28487554
```

The TTGCACG could sit on either side of the breakpoint.

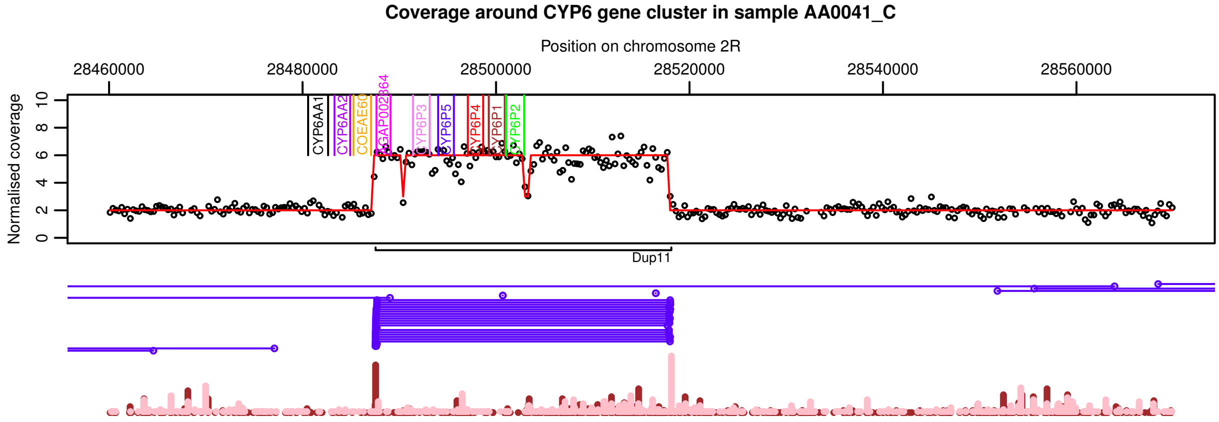

Fig. AA_S23: Example of coverage in an individual carrying the Cyp6aap_Dup11 duplication. Open black circles indicate coverage at each position. The red line shows the HMM estimation of the coverage state at each position. Vertical lines represent the positions of the various gene. Pairs of blue points connected by lines indicate pairs of face-away reads. Reads soft-clipped before the alignment start point (dark brown points) and after the alignment end point (light brown points) are present at the start and end points of the duplication (clipped at positions 28487546 and 28518123 respectively). In each case, the clipped bases align to the other end of the duplication, as expected.

The majority of samples with Cyp6aap_Dup11 are in *An. coluzzii* from Côte d'ivoire (Table AA_S11.1). Estimates of copy number for Cyp6aap_Dup11 in this population indicated that copy number ranged from 1 to 4 (Tables AA_S11.1 & 11.2), suggesting that both duplications and triplications exist. It is therefore not possible to call alleles based on coverage for Cyp6aap_Dup11.

Table AA_S11.1: Copy number calls for Cyp6aap_Dup11. NAs were produced if coverage was too variable or if the duplication completely overlapped with another duplication whose coverage could also not be called.

copy number	AO col	BF col	BF gam	CI col	CM gam	FR gam	GA gam	GH col	GH gam	GM	GN col	GN gam	GQ gam	GW	KE	UG gam
0	78	75	92	42	297	24	69	52	12	65	4	40	9	91	48	112
1	0	0	0	7	0	0	0	1	0	0	0	0	0	0	0	0
2	0	0	0	17	0	0	0	1	0	0	0	0	0	0	0	0
3	0	0	0	4	0	0	0	0	0	0	0	0	0	0	0	0
4	0	0	0	1	0	0	0	1	0	0	0	0	0	0	0	0

Table AA_S11.2: Copy number calls for all duplications in individuals that carry Cyp6aap_Dup11.

	Dup 0	Dup 1	Dup 2	Dup 3	Dup 4	Dup 5	Dup 6	Dup 7	Dup 8	Dup 9	Dup 10	Dup 11	Dup 12	Dup 13	Dup 14	Dup 15
AA0041_C	0	0	0	0	0	0	0	0	0	0	0	4	0	0	0	0
AA0087_C	0	0	0	0	0	0	0	0	0	0	0	2	0	0	0	0
AA0100_C	0	0	0	0	0	0	0	0	0	0	0	1	0	0	0	0
AY0006_C	0	0	0	0	0	0	0	1	0	0	0	2	0	0	0	0
AY0007_C	0	0	0	0	0	0	0	0	0	0	0	1	0	0	1	1
AY0011_C	0	0	0	0	0	0	0	0	0	0	0	1	0	0	1	0
AY0012_C	0	0	0	0	0	0	0	0	0	0	0	1	0	0	1	0
AY0013_C	0	0	0	0	0	0	0	1	0	0	0	2	0	0	1	0
AY0015_C	0	0	0	0	0	0	0	0	0	0	0	4	0	0	0	0
AY0016_C	0	0	0	0	0	0	0	0	0	0	0	3	0	0	1	1
AY0020_C	0	0	0	0	0	0	0	1	0	0	0	1	0	0	1	0
AY0023_C	0	0	0	0	0	0	0	1	0	0	0	3	0	0	0	1
AY0026_C	0	0	0	0	0	0	0	0	0	0	0	3	0	0	0	0
AY0033_C	0	0	0	0	0	0	0	0	0	0	0	2	0	0	1	0
AY0039_C	0	0	0	0	0	0	0	1	0	0	0	2	0	1	0	1
AY0043_C	0	0	0	0	0	0	0	0	0	0	0	1	0	0	1	1
AY0045_C	0	0	0	0	0	0	0	1	0	0	0	1	0	0	1	0
AY0052_C	0	0	0	0	0	0	0	0	0	0	0	2	0	0	1	0
AY0055_C	0	0	0	0	0	0	0	1	0	0	0	2	0	0	0	0
AY0057_C	0	0	0	0	0	0	0	0	0	0	0	2	0	0	0	1
AY0058_C	0	0	0	0	0	0	0	0	0	0	0	2	0	0	0	0
AY0060_C	0	0	0	0	0	0	0	1	0	0	0	2	0	0	0	0
AY0064_C	0	0	0	0	0	0	0	0	0	0	0	2	0	0	0	0
AY0068_C	0	0	0	0	0	0	0	0	0	0	0	2	0	0	1	0
AY0076_C	0	0	0	0	0	0	0	1	0	0	0	2	0	0	2	0
AY0077_C	0	0	0	0	0	0	0	1	0	0	0	2	0	0	0	0
AY0078_C	0	0	0	0	0	0	0	0	0	0	0	2	0	0	0	1
AY0080_C	0	0	0	0	0	0	0	0	0	0	0	2	0	0	0	0
AY0085_C	0	0	0	0	0	0	0	1	0	0	0	2	0	0	0	0
AY0088_C	0	0	0	0	0	0	0	1	0	0	0	2	0	0	0	0
AY0089_C	0	0	0	0	0	0	0	0	0	0	0	1	0	0	1	1
AY0090_C	0	0	0	0	0	0	0	0	0	0	0	3	0	0	0	0

Duplication type 12

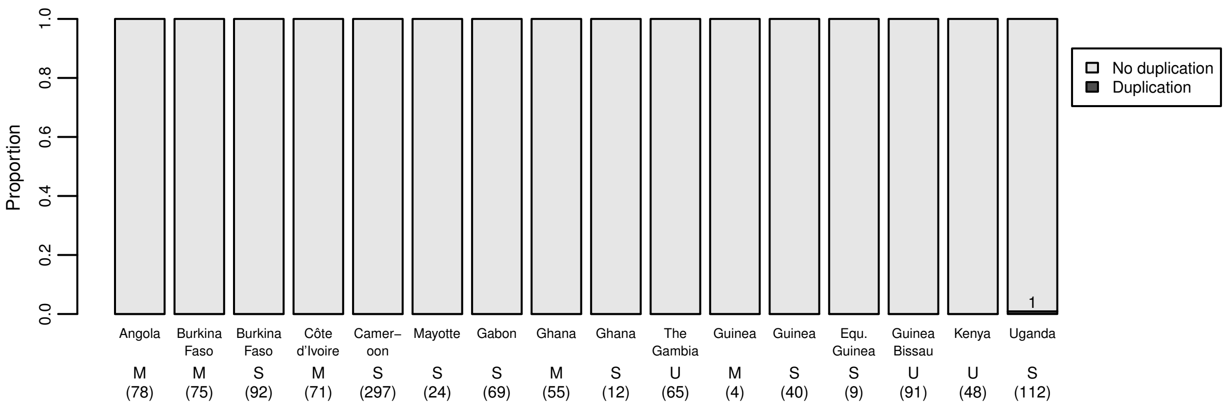

Fig. AA_S24: Barplot showing the proportion of samples that carry the Cyp6aap_Dup12 duplication in each of the Phase 2 populations. Numbers above the dark grey bars indicate the absolute number of samples carrying the duplication. S = *Anopheles gambiae*, M = *Anopheles coluzzi*, U = species undetermined. Numbers in brackets indicate the total number of samples from that population.

Cyp6aap_Dup12 was supported by face-away read pairs whose forward-facing read mapped in the interval 28474450 - 28474750 and whose reverse-facing read mapped in the interval 28519650 - 28519950 (Fig. **AA_S25**).Cyp6aap_Dup12 was also supported by reads soft-clipped at the breakpoints (positions 28474576 and 28520016), with the clipped bases at each breakpoint aligning at the other breakpoint.

Cyp6aap_Dup12 breakpoint:

```
AATCATACGGGACCAGCC A ACGGTAAGCCAGCAAAA
  end of the dup ^   ^ start of the dup
 position 28520014   position 28474578
```

The A could sit on either side of the breakpoint.

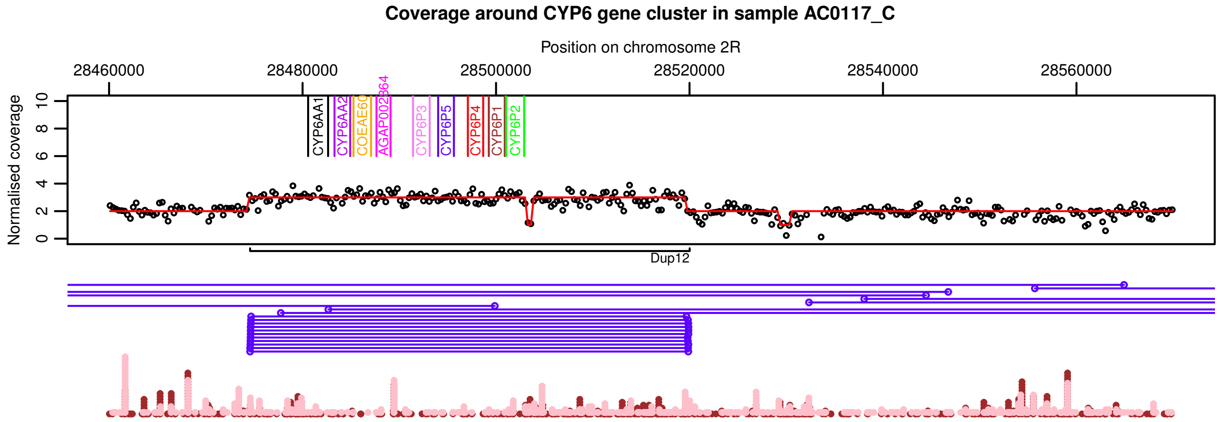

Fig. AA_S25: Coverage in the individual carrying the Cyp6aap_Dup12 duplication. Open black circles indicate coverage at each position. The red line shows the HMM estimation of the coverage state at each position. Vertical lines represent the positions of the various gene. Pairs of blue points connected by lines indicate pairs of face-away reads. Reads soft-clipped before the alignment start point (dark brown points) and after the alignment end point (light brown points) are present at the start and end points of the duplication (clipped at positions 28474576 and 28520016 respectively). In each case, the clipped bases align to the other end of the duplication, as expected.

The only sample that carries Cyp6aap_Dup12 has a copy number of 1 (Tables AA_S12.1 & 12.2). Cyp6aap_Dup12 is therefore a single copy duplication and the sample is heterozygous.

Table AA_S12.1: Copy number calls for Cyp6aap_Dup12. NAs were produced if coverage was too variable or if the duplication completely overlapped with another duplication whose coverage could also not be called.

copy number	AO col	BF col	BF gam	CI col	CM gam	FR gam	GA gam	GH col	GH gam	GM	GN col	GN gam	GQ gam	GW	KE	UG gam
0	78	75	92	71	297	24	69	55	12	65	4	40	9	91	48	111
1	0	0	0	0	0	0	0	0	0	0	0	0	0	0	0	1

Table AA_S12.2: Copy number calls for all duplications in individuals that carry Cyp6aap_Dup12.

	Dup 0	Dup 1	Dup 2	Dup 3	Dup 4	Dup 5	Dup 6	Dup 7	Dup 8	Dup 9	Dup 10	Dup 11	Dup 12	Dup 13	Dup 14	Dup 15
AC0117_C	0	0	0	0	0	0	0	0	0	0	0	0	1	0	0	0

Duplication type 13

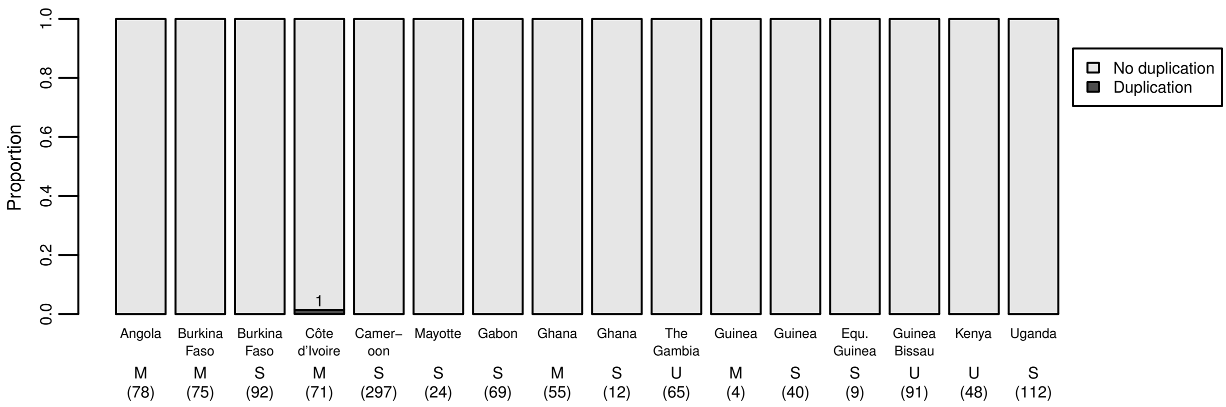

Fig. AA_S26: Barplot showing the proportion of samples that carry the Cyp6aap_Dup13 duplication in each of the Phase 2 populations. Numbers above the dark grey bars indicate the absolute number of samples carrying the duplication. S = *Anopheles gambiae*, M = *Anopheles coluzzi*, U = species undetermined. Numbers in brackets indicate the total number of samples from that population.

Cyp6aap_Dup13 was supported by face-away read pairs whose forward-facing read mapped in the interval 28472650 - 28472950 and whose reverse-facing read mapped in the interval 28522350 - 28522650 (Fig. Fig. **AA_S27**).Cyp6aap_Dup13 was also supported by reads soft-clipped at the breakpoints (positions 28472728 and 28522671), with the clipped bases at each breakpoint aligning at the other breakpoint.

Cyp6aap_Dup13 breakpoint:

```
   CGTACAGCATCGCCA TCCCG AAGAGCTGACGGAAGAAG
  end of the dup ^       ^ start of the dup
 position 28522662       position 28472734
```

The TCCCG could sit on either side of the breakpoint.

Coverage around CYP6 gene cluster in sample AY0039_C

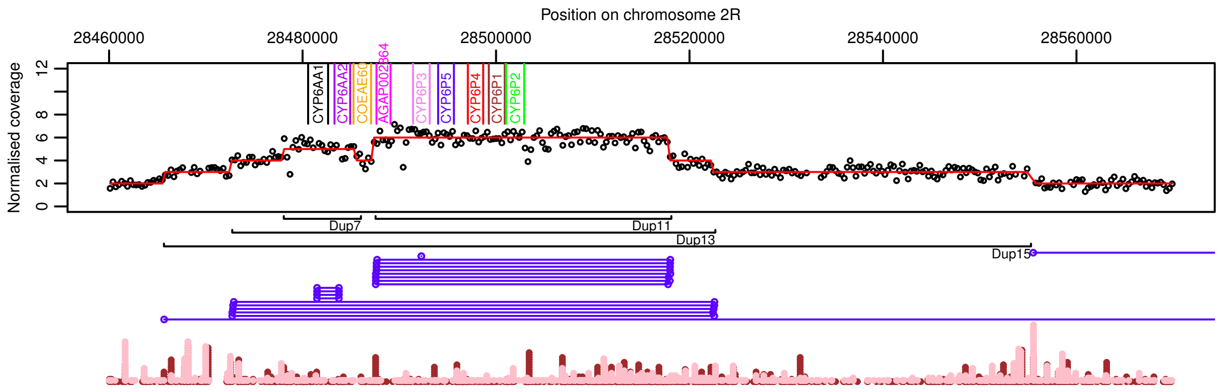

Fig. AA_S27: Coverage in the individual carrying the Cyp6aap_Dup13 duplication. Open black circles indicate coverage at each position. The red line shows the HMM estimation of the coverage state at each position. Vertical lines represent the positions of the various gene. Pairs of blue points connected by lines indicate pairs of face-away reads. Reads soft-clipped before the alignment start point (dark brown points) and after the alignment end point (light brown points) are present at the start and end points of the duplication (clipped at positions 28472728 and 28522671 respectively). In each case, the clipped bases align to the other end of the duplication, as expected.

The only sample that carries Cyp6aap_Dup13 has a copy number of 1 (Tables AA_S13.1 & 13.2). Cyp6aap_Dup13 is therefore a single copy duplication and the sample is heterozygous.

Table AA_S13.1: Copy number calls for Cyp6aap_Dup13. NAs were produced if coverage was too variable or if the duplication completely overlapped with another duplication whose coverage could also not be called.

copy number	AO col	BF col	BF gam	CI col	CM gam	FR gam	GA gam	GH col	GH gam	GM	GN col	GN gam	GQ gam	GW	KE	UG gam
0	78	75	92	70	297	24	69	55	12	65	4	40	9	91	48	112
1	0	0	0	1	0	0	0	0	0	0	0	0	0	0	0	0

Table AA_S13.2: Copy number calls for all duplications in individuals that carry Cyp6aap_Dup13.

	Dup 0	Dup 1	Dup 2	Dup 3	Dup 4	Dup 5	Dup 6	Dup 7	Dup 8	Dup 9	Dup 10	Dup 11	Dup 12	Dup 13	Dup 14	Dup 15
AY0039_C	0	0	0	0	0	0	0	1	0	0	0	2	0	1	0	1

Duplication type 14

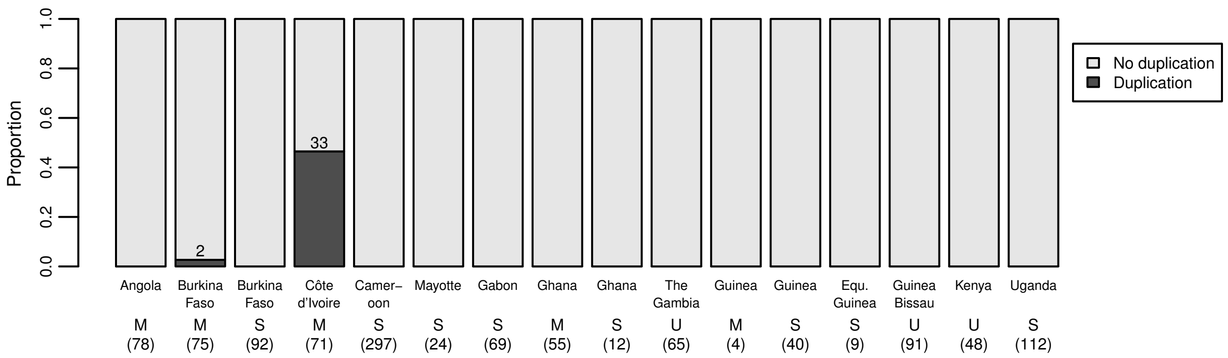

Fig. AA_S28: Barplot showing the proportion of samples that carry the Cyp6aap_Dup14 duplication in each of the Phase 2 populations. Numbers above the dark grey bars indicate the absolute number of samples carrying the duplication. S = *Anopheles gambiae*, M = *Anopheles coluzzi*, U = species undetermined. Numbers in brackets indicate the total number of samples from that population.

Cyp6aap_Dup14 was supported by face-away read pairs whose forward-facing read mapped in the interval 28473800 - 28474100 and whose reverse-facing read mapped in the interval 28563200 - 28563500 (Fig. Fig. **AA_S29**). One sample Cyp6aap_Dup14 was also supported by reads soft-clipped at the breakpoints (positions 28473874 and 28563596), with the clipped bases at each breakpoint aligning at the other breakpoint.did not have the face-away reads, but Cyp6aap_Dup14 could nevertheless be identified by the soft-clipped reads at the breakpoints.

Cyp6aap_Dup14 breakpoint:

```
CAACAACTACTATTCCACCC GC AGTTGGGCTGAGGAAGCTGC
      end of the dup ^    ^ start of the dup
     position 28563593    position 28473877
```

The GC could sit on either side of the breakpoint.

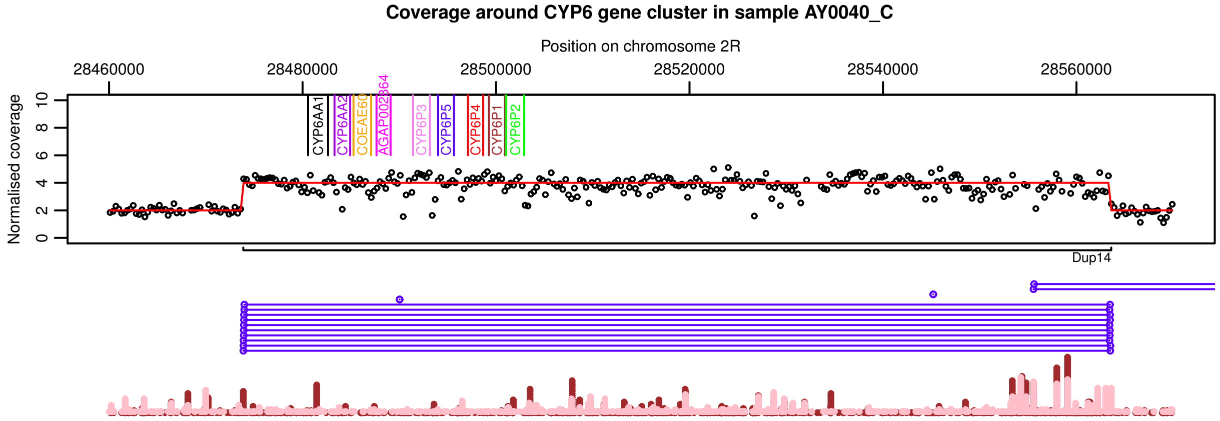

Fig. AA_S29: Example of coverage in an individual carrying the Cyp6aap_Dup14 duplication. Open black circles indicate coverage at each position. The red line shows the HMM estimation of the coverage state at each position. Vertical lines represent the positions of the various genes. Pairs of blue points connected by lines indicate pairs of face-away reads. Reads soft-clipped before the alignment start point (dark brown points) and after the alignment end point (light brown points) are present at the start and end points of the duplication (clipped at positions 28473874 and 28563596 respectively). In each case, the clipped bases align to the other end of the duplication, as expected.

The majority of samples with Cyp6aap_Dup14 are in *An. coluzzii* from Côte d'ivoire (Table AA_S14.1). Estimates of copy number for Cyp6aap_Dup14 in this population indicated that only one sample had a copy number higer than 2. Assuming that samples a copy number of 1 are heterozygotes, and that samples with a copy number of 2 are homozygotes for Cyp6aap_Dup14 (Table AA_S14.2), the allele distribution is consistent with Hardy-Weinberg (HW) expectations ($P = 0.32$). The sample with an apparent copy number of 3 may represent a mis-calling of coverage, or a case of a rare triplication.

Table AA_S14.1: Copy number calls for Cyp6aap_Dup14. NAs were produced if coverage was too variable or if the duplication completely overlapped with another duplication whose coverage could also not be called.

copy number	AO col	BF col	BF gam	CI col	CM gam	FR gam	GA gam	GH col	GH gam	GM	GN col	GN gam	GQ gam	GW	KE	UG gam
NA	0	0	0	1	0	0	0	0	0	0	0	0	0	0	0	0
0	78	73	92	38	297	24	69	55	12	65	4	40	9	91	48	112
1	0	2	0	29	0	0	0	0	0	0	0	0	0	0	0	0
2	0	0	0	2	0	0	0	0	0	0	0	0	0	0	0	0
3	0	0	0	1	0	0	0	0	0	0	0	0	0	0	0	0

Table AA_S14.2: Copy number calls for all duplications in individuals that carry Cyp6aap_Dup14.

	Dup 0	Dup 1	Dup 2	Dup 3	Dup 4	Dup 5	Dup 6	Dup 7	Dup 8	Dup 9	Dup 10	Dup 11	Dup 12	Dup 13	Dup 14	Dup 15
AB0090_C	0	0	0	0	0	0	0	0	0	0	1	0	0	0	1	0
AB0209_C	0	0	0	0	0	0	0	1	0	0	1	0	0	0	1	0
AY0007_C	0	0	0	0	0	0	0	0	0	0	0	1	0	0	1	1
AY0010_C	0	0	0	0	0	0	0	0	0	0	0	0	0	0	3	0
AY0011_C	0	0	0	0	0	0	0	0	0	0	0	1	0	0	1	0
AY0012_C	0	0	0	0	0	0	0	0	0	0	0	1	0	0	1	0
AY0013_C	0	0	0	0	0	0	0	1	0	0	0	2	0	0	1	0
AY0016_C	0	0	0	0	0	0	0	0	0	0	0	3	0	0	1	1
AY0017_C	0	0	0	0	0	0	0	0	0	0	0	0	0	0	1	0
AY0019_C	0	0	0	0	0	0	0	0	0	0	0	0	0	0	1	0
AY0020_C	0	0	0	0	0	0	0	1	0	0	0	1	0	0	1	0
AY0025_C	0	0	0	0	0	0	0	0	0	0	0	0	0	0	1	0
AY0029_C	0	0	0	0	0	0	0	1	0	0	0	0	0	0	1	1
AY0032_C	0	0	0	0	0	0	0	0	0	0	0	0	0	0	1	1
AY0033_C	0	0	0	0	0	0	0	0	0	0	0	2	0	0	1	0
AY0036_C	0	0	0	0	0	0	0	0	0	0	0	0	0	0	1	0
AY0038_C	0	0	0	0	0	0	0	1	0	0	0	0	0	0	1	0
AY0040_C	0	0	0	0	0	0	0	0	0	0	0	0	0	0	2	0
AY0043_C	0	0	0	0	0	0	0	0	0	0	0	1	0	0	1	1
AY0045_C	0	0	0	0	0	0	0	1	0	0	0	1	0	0	1	0
AY0049_C	0	0	0	0	0	0	0	0	0	0	0	0	0	0	1	0
AY0052_C	0	0	0	0	0	0	0	0	0	0	0	2	0	0	1	0
AY0054_C	0	0	0	0	0	0	0	0	0	0	0	0	0	0	1	0
AY0056_C	0	0	0	0	0	0	0	0	0	0	0	0	0	0	1	0
AY0061_C	0	0	0	0	0	0	0	0	0	0	0	0	0	0	1	0
AY0063_C	0	0	0	0	0	0	0	0	0	0	0	0	0	0	1	0
AY0065_C	0	0	0	0	0	0	0	0	0	0	0	0	0	0	1	0
AY0067_C	0	0	0	0	0	0	0	0	0	0	0	0	0	0	1	0
AY0068_C	0	0	0	0	0	0	0	0	0	0	0	2	0	0	1	0
AY0069_C	0	0	0	0	0	0	0	0	0	0	0	0	0	0	NA	2
AY0070_C	0	0	0	0	0	0	0	0	0	0	0	0	0	0	1	1
AY0072_C	0	0	0	0	0	0	0	0	0	0	0	0	0	0	1	0
AY0076_C	0	0	0	0	0	0	0	1	0	0	0	2	0	0	2	0
AY0083_C	0	0	0	0	0	0	0	0	0	0	0	0	0	0	1	1
AY0089_C	0	0	0	0	0	0	0	0	0	0	0	1	0	0	1	1

Duplication type 15

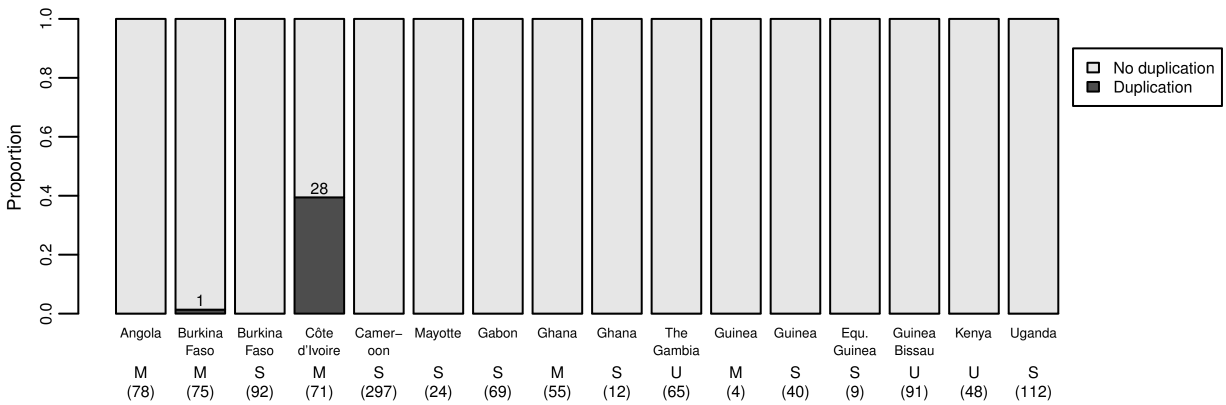

Fig. AA_S30: Barplot showing the proportion of samples that carry the Cyp6aap_Dup15 duplication in each of the Phase 2 populations. Numbers above the dark grey bars indicate the absolute number of samples carrying the duplication. S = *Anopheles gambiae*, M = *Anopheles coluzzi*, U = species undetermined. Numbers in brackets indicate the total number of samples from that population.

Cyp6aap_Dup15 was supported in some samples by face-away read pairs whose forward-facing read mapped in the interval 28465600 - 28465900 and whose reverse-facing read mapped in the interval 55958800 - 55959100 (Fig. Fig. **AA_S31**). However, while the coverage increase starts around that former interval, it does not extend as far as the latter interval, stopping around 28555000. Furthermore, some sample do not have these face-away reads but instead have reads that map to the same first interval, and whose mates map to a different chromosome. One side of the breakpoint can therefore be accurately mapped, while the other cannot.Accordingly, Cyp6aap_Dup15 was only supported by reads soft-clipped at one side of the breakpoint (position 28465673), with the clipped bases aligning at varying places in the genome.

Cyp6aap_Dup15 breakpoint:

```
ACTCCGAACGACTCCGAC  TCGCTACGGATTCAGAA
  end of the dup ^  ^ start of the dup
  position unknown  position 28465674
```

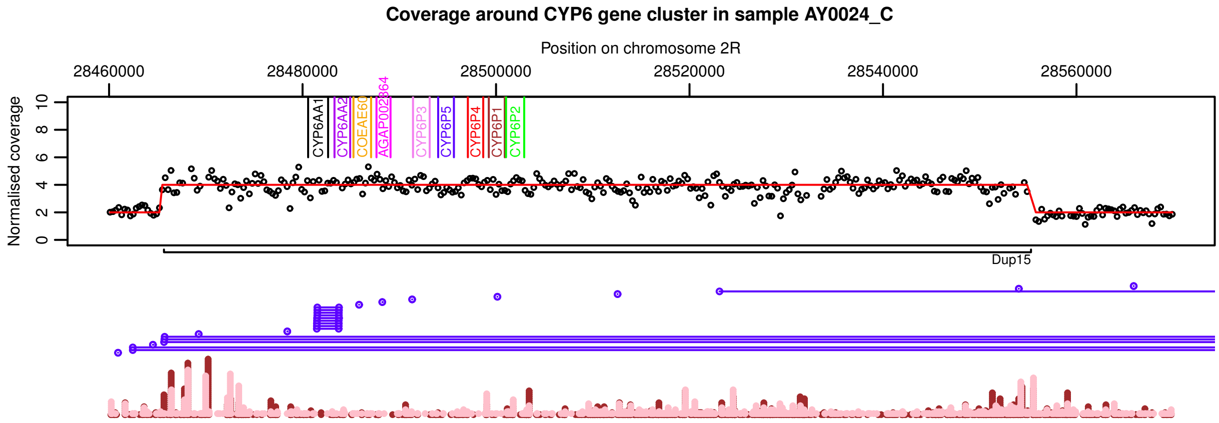

Fig. AA_S31: Example of coverage in an individual carrying the Cyp6aap_Dup15 duplication. Open black circles indicate coverage at each position. The red line shows the HMM estimation of the coverage state at each position. Vertical lines represent the positions of the various gene. Pairs of blue points connected by lines indicate pairs of face-away reads. Reads soft-clipped before the alignment start point (dark brown points) are present at the start point of the duplication (clipped at positions 28465673). In each case, the clipped bases align to the other end of the duplication, as expected.

The majority of samples with Cyp6aap_Dup15 are in *An. coluzzii* from Côte d'ivoire (Table AA_S15.1). Estimates of copy number for Cyp6aap_Dup11 in this population indicated that no sample has a copy number higher than 2. Assuming that samples with a copy number of 1 are heterozygotes and that samples with a copy number of 2 are homozygotes for Cyp6aap_Dup15 (Table AA_S15.2), the allele distribution is consistent with HW expectations ($P = 1$).

Table AA_S15.1: Copy number calls for Cyp6aap_Dup15. NAs were produced if coverage was too variable or if the duplication completely overlapped with another duplication whose coverage could also not be called.

copy number	AO col	BF col	BF gam	CI col	CM gam	FR gam	GA gam	GH col	GH gam	GM	GN col	GN gam	GQ gam	GW	KE	UG gam
0	78	74	92	43	297	24	69	55	12	65	4	40	9	91	48	112
1	0	1	0	25	0	0	0	0	0	0	0	0	0	0	0	0
2	0	0	0	3	0	0	0	0	0	0	0	0	0	0	0	0

Table AA_S15.2: Copy number calls for all duplications in individuals that carry Cyp6aap_Dup15.

	Dup 0	Dup 1	Dup 2	Dup 3	Dup 4	Dup 5	Dup 6	Dup 7	Dup 8	Dup 9	Dup 10	Dup 11	Dup 12	Dup 13	Dup 14	Dup 15
AB0095_C	0	0	0	0	0	0	0	0	0	0	0	0	0	0	0	1
AY0007_C	0	0	0	0	0	0	0	0	0	0	0	1	0	0	1	1
AY0016_C	0	0	0	0	0	0	0	0	0	0	0	3	0	0	1	1
AY0018_C	0	0	0	0	0	0	0	1	0	0	0	0	0	0	0	1
AY0023_C	0	0	0	0	0	0	0	1	0	0	0	3	0	0	0	1
AY0024_C	0	0	0	0	0	0	0	0	0	0	0	0	0	0	0	2
AY0027_C	0	0	0	0	0	0	0	0	0	0	0	0	0	0	0	1
AY0029_C	0	0	0	0	0	0	0	1	0	0	0	0	0	0	1	1
AY0032_C	0	0	0	0	0	0	0	0	0	0	0	0	0	0	1	1
AY0034_C	0	0	0	0	0	0	0	0	0	0	0	0	0	0	0	1
AY0035_C	0	0	0	0	0	0	0	0	0	0	0	0	0	0	0	1
AY0039_C	0	0	0	0	0	0	0	1	0	0	0	2	0	1	0	1
AY0043_C	0	0	0	0	0	0	0	0	0	0	0	1	0	0	1	1
AY0048_C	0	0	0	0	0	0	0	1	0	0	0	0	0	0	0	1
AY0050_C	0	0	0	0	0	0	0	0	0	0	0	0	0	0	0	1
AY0053_C	0	0	0	0	0	0	0	0	0	0	0	0	0	0	0	1
AY0057_C	0	0	0	0	0	0	0	0	0	0	0	2	0	0	0	1
AY0059_C	0	0	0	0	0	0	0	0	0	0	0	0	0	0	0	1
AY0062_C	0	0	0	0	0	0	0	1	0	0	0	0	0	0	0	1
AY0066_C	0	0	0	0	0	0	0	0	0	0	0	0	0	0	0	1
AY0069_C	0	0	0	0	0	0	0	0	0	0	0	0	0	0	NA	2
AY0070_C	0	0	0	0	0	0	0	0	0	0	0	0	0	0	1	1
AY0078_C	0	0	0	0	0	0	0	0	0	0	0	2	0	0	0	1
AY0079_C	0	0	0	0	0	0	0	1	0	0	0	0	0	0	0	1
AY0082_C	0	0	0	0	0	0	0	0	0	0	0	0	0	0	0	2
AY0083_C	0	0	0	0	0	0	0	0	0	0	0	0	0	0	1	1
AY0087_C	0	0	0	0	0	0	0	0	0	0	0	0	0	0	0	1
AY0089_C	0	0	0	0	0	0	0	0	0	0	0	1	0	0	1	1
AY0091_C	0	0	0	0	0	0	0	0	0	0	0	0	0	0	0	1

Table AA_S16: Coverage calls for all duplications in the Cyp6aa-Cyp6p cluster in all individuals.

	Dup 0	Dup 1	Dup 2	Dup 3	Dup 4	Dup 5	Dup 6	Dup 7	Dup 8	Dup 9	Dup 10	Dup 11	Dup 12	Dup 13	Dup 14	Dup 15
AA0040_C	0	0	0	0	0	0	0	0	0	0	0	0	0	0	0	0
AA0041_C	0	0	0	0	0	0	0	0	0	0	0	4	0	0	0	0
AA0042_C	0	0	0	0	0	0	0	0	0	0	0	0	0	0	0	0
AA0043_C	0	0	0	0	0	0	0	0	0	0	1	0	0	0	0	0
AA0044_C	0	0	0	0	0	0	0	0	0	0	0	0	0	0	0	0
AA0048_C	0	0	0	0	0	0	0	0	0	0	0	0	0	0	0	0
AA0049_C	0	0	0	0	0	0	0	0	0	0	0	0	0	0	0	0
AA0050_C	0	0	0	0	0	0	0	0	0	0	0	0	0	0	0	0
AA0051_C	0	0	0	0	0	0	0	0	0	0	0	0	0	0	0	0
AA0052_C	0	0	0	0	0	0	0	1	0	0	0	0	0	0	0	0
AA0053_C	0	0	0	0	0	0	0	0	0	0	0	0	0	0	0	0
AA0054_C	0	0	0	0	0	0	0	0	0	0	0	0	0	0	0	0
AA0055_C	0	0	0	0	0	0	0	0	0	0	0	0	0	0	0	0
AA0056_C	0	0	0	0	0	0	0	0	0	0	0	0	0	0	0	0
AA0060_C	0	0	0	0	0	0	0	0	0	0	0	0	0	0	0	0
AA0061_C	0	0	0	0	0	0	0	0	0	0	0	0	0	0	0	0
AA0063_C	0	0	0	0	0	0	0	0	0	0	0	0	0	0	0	0
AA0064_C	0	0	0	0	0	0	0	0	0	0	0	0	0	0	0	0
AA0066_C	0	0	0	0	0	0	0	1	0	0	0	0	0	0	0	0
AA0067_C	0	0	0	0	0	0	0	0	0	0	0	0	0	0	0	0
AA0068_C	0	0	0	0	0	0	0	0	0	0	0	0	0	0	0	0
AA0072_C	0	0	0	0	0	0	0	0	0	0	0	0	0	0	0	0
AA0073_C	0	0	0	0	0	0	0	0	0	0	0	0	0	0	0	0
AA0074_C	0	0	0	0	0	0	0	0	0	0	0	0	0	0	0	0
AA0075_C	0	0	0	0	0	0	0	0	0	0	0	0	0	0	0	0
AA0076_C	0	0	0	0	0	0	0	0	0	0	0	0	0	0	0	0
AA0077_C	0	0	0	0	0	0	0	0	0	0	0	0	0	0	0	0
AA0080_C	0	0	0	0	0	0	0	0	0	0	0	0	0	0	0	0
AA0084_C	0	0	0	0	0	0	0	0	0	0	0	0	0	0	0	0
AA0085_C	0	0	0	0	0	0	0	0	0	0	0	0	0	0	0	0
AA0086_C	0	0	0	0	0	0	0	0	0	0	0	0	0	0	0	0
AA0087_C	0	0	0	0	0	0	0	0	0	0	0	2	0	0	0	0
AA0088_C	0	0	0	0	0	0	0	0	0	0	1	0	0	0	0	0
AA0089_C	0	0	0	0	0	0	0	0	0	0	0	0	0	0	0	0
AA0090_C	0	0	0	0	0	0	0	0	0	0	0	0	0	0	0	0
AA0091_C	0	0	0	0	0	0	0	0	0	0	0	0	0	0	0	0
AA0096_C	0	0	0	0	0	0	0	0	0	0	0	0	0	0	0	0
AA0097_C	0	0	0	0	0	0	0	0	0	0	0	0	0	0	0	0
AA0098_C	0	0	0	0	0	0	0	0	0	0	0	0	0	0	0	0
AA0099_C	0	0	0	0	0	0	0	0	0	0	0	0	0	0	0	0
AA0100_C	0	0	0	0	0	0	0	0	0	0	0	1	0	0	0	0
AA0101_C	0	0	0	0	0	0	0	0	0	0	0	0	0	0	0	0
AA0102_C	0	0	0	0	0	0	0	0	0	0	0	0	0	0	0	0
AA0103_C	0	0	0	0	0	0	0	0	0	0	0	0	0	0	0	0
AA0104_C	0	0	0	0	0	0	0	0	0	0	0	0	0	0	0	0
AA0107_C	0	0	0	0	0	0	0	0	0	0	0	0	0	0	0	0
AA0108_C	0	0	0	0	0	0	0	0	0	0	0	0	0	0	0	0
AA0109_C	0	0	0	0	0	0	0	0	0	0	0	0	0	0	0	0
AA0110_C	0	0	0	0	0	0	0	0	0	0	0	0	0	0	0	0
AA0111_C	0	0	0	0	0	0	0	0	0	0	0	0	0	0	0	0
AA0113_C	0	0	0	0	0	0	0	0	0	0	0	0	0	0	0	0
AA0114_C	0	0	0	0	0	0	0	0	0	0	0	0	0	0	0	0
AA0115_C	0	0	0	0	0	0	0	0	0	0	0	0	0	0	0	0
AA0116_C	0	0	0	0	0	0	0	0	0	0	0	0	0	0	0	0
AA0122_C	0	0	0	0	0	0	0	0	0	0	0	0	0	0	0	0
AA0123_C	0	0	0	0	0	0	0	0	0	0	0	0	0	0	0	0
AA0124_C	0	0	0	0	0	0	0	0	0	0	2	0	0	0	0	0
AA0125_C	0	0	0	0	0	0	0	0	0	0	0	0	0	0	0	0
AA0127_C	0	0	0	0	0	0	0	0	0	0	0	0	0	0	0	0
AA0132_C	0	0	0	0	0	0	0	0	0	0	0	0	0	0	0	0
AA0133_C	0	0	0	0	0	0	0	0	0	0	0	0	0	0	0	0
AA0134_C	0	0	0	0	0	0	0	0	0	0	0	0	0	0	0	0
AA0135_C	0	0	0	0	0	0	0	1	0	0	0	0	0	0	0	0
AA0136_C	0	0	0	0	0	0	0	0	0	0	0	0	0	0	0	0
AA0139_C	0	0	0	0	0	0	0	0	0	0	0	0	0	0	0	0
AA0140_C	0	0	0	0	0	0	0	0	0	0	0	0	0	0	0	0

	Dup 0	Dup 1	Dup 2	Dup 3	Dup 4	Dup 5	Dup 6	Dup 7	Dup 8	Dup 9	Dup 10	Dup 11	Dup 12	Dup 13	Dup 14	Dup 15
AA0141_C	0	0	0	0	0	0	0	0	0	0	0	0	0	0	0	0
AB0085_C	0	0	0	0	0	0	0	0	0	0	0	0	0	0	0	0
AB0087_C	0	0	0	0	0	0	0	0	0	0	2	0	0	0	0	0
AB0088_C	0	0	0	0	0	0	0	2	0	0	0	0	0	0	0	0
AB0089_C	0	0	0	0	0	0	0	0	0	0	0	0	0	0	0	0
AB0090_C	0	0	0	0	0	0	0	0	0	0	1	0	0	0	1	0
AB0091_C	0	0	0	0	0	0	0	1	0	0	1	0	0	0	0	0
AB0092_C	0	0	0	0	0	0	0	0	0	0	0	0	0	0	0	0
AB0094_C	0	0	0	0	0	0	0	0	0	0	1	0	0	0	0	0
AB0095_C	0	0	0	0	0	0	0	0	0	0	0	0	0	0	0	1
AB0097_C	0	0	0	0	0	0	0	0	0	0	2	0	0	0	0	0
AB0098_C	0	0	0	0	0	0	0	1	0	0	0	0	0	0	0	0
AB0099_C	0	0	0	0	0	0	0	1	0	0	1	0	0	0	0	0
AB0100_C	0	0	0	0	0	0	0	1	0	0	1	0	0	0	0	0
AB0101_C	0	0	0	0	0	0	0	0	0	0	0	0	0	0	0	0
AB0103_C	0	0	0	0	0	0	0	0	0	0	0	0	0	0	0	0
AB0104_C	0	0	0	0	0	0	0	0	0	0	0	0	0	0	0	0
AB0108_C	0	0	0	0	0	0	0	0	0	0	0	0	0	0	0	0
AB0109_C	0	0	0	0	2	0	0	0	0	0	0	0	0	0	0	0
AB0110_C	0	1	0	0	0	0	0	0	0	0	0	0	0	0	0	0
AB0111_C	0	0	0	0	0	0	0	0	0	0	2	0	0	0	0	0
AB0112_C	0	0	0	0	0	0	0	1	0	0	0	0	0	0	0	0
AB0113_C	0	0	0	0	0	0	0	0	0	0	0	0	0	0	0	0
AB0114_C	0	0	0	0	0	0	0	1	0	0	0	0	0	0	0	0
AB0115_C	0	0	0	0	0	0	0	0	0	0	2	0	0	0	0	0
AB0117_C	0	0	0	0	0	0	0	0	0	0	0	0	0	0	0	0
AB0118_C	0	0	0	0	0	0	0	0	0	0	0	0	0	0	0	0
AB0119_C	0	0	0	0	0	0	0	0	0	0	0	0	0	0	0	0
AB0122_C	0	0	0	0	0	0	0	0	0	0	2	0	0	0	0	0
AB0123_C	0	0	0	0	0	0	0	1	0	0	1	0	0	0	0	0
AB0124_C	0	0	0	0	0	0	0	1	0	0	0	0	0	0	0	0
AB0126_C	0	0	0	0	0	0	0	0	0	0	0	0	0	0	0	0
AB0127_C	0	0	0	0	0	0	0	0	0	0	0	0	0	0	0	0
AB0128_C	0	0	0	0	0	0	0	0	0	0	0	0	0	0	0	0
AB0129_C	0	0	0	0	0	0	0	0	0	0	0	0	0	0	0	0
AB0130_C	0	0	0	0	0	0	0	0	0	0	0	0	0	0	0	0
AB0133_C	0	0	0	0	0	0	0	0	0	0	0	0	0	0	0	0
AB0134_C	0	0	0	0	0	0	0	0	0	0	0	0	0	0	0	0
AB0135_C	0	0	0	0	0	0	0	0	1	0	0	0	0	0	0	0
AB0136_C	0	0	0	0	0	0	0	0	0	0	0	0	0	0	0	0
AB0137_C	0	0	0	0	0	0	0	1	0	0	0	0	0	0	0	0
AB0138_C	0	0	0	0	0	0	0	0	0	0	1	0	0	0	0	0
AB0139_C	0	0	0	0	0	0	0	0	0	0	1	0	0	0	0	0
AB0140_C	0	0	0	0	0	0	0	2	0	0	0	0	0	0	0	0
AB0142_C	0	0	0	0	0	0	0	2	0	0	0	0	0	0	0	0
AB0143_C	0	0	0	0	0	0	0	0	0	0	0	0	0	0	0	0
AB0145_C	0	0	0	0	0	0	0	0	0	0	0	0	0	0	0	0
AB0146_C	0	0	0	0	0	0	0	0	0	0	0	0	0	0	0	0
AB0147_C	0	0	0	0	0	0	0	0	0	0	0	0	0	0	0	0
AB0148_C	0	0	0	0	0	0	0	0	0	0	0	0	0	0	0	0
AB0150_C	0	0	0	0	0	0	0	0	0	0	0	0	0	0	0	0
AB0151_C	0	0	0	0	0	0	0	0	0	0	0	0	0	0	0	0
AB0153_C	0	0	0	0	0	0	0	0	0	0	0	0	0	0	0	0
AB0155_C	0	0	0	0	0	0	0	0	0	0	0	0	0	0	0	0
AB0157_C	0	0	0	0	0	0	0	0	0	0	0	0	0	0	0	0
AB0158_C	0	0	0	0	0	0	0	0	0	0	0	0	0	0	0	0
AB0159_C	0	0	0	0	0	0	0	0	0	0	0	0	0	0	0	0
AB0160_C	0	0	0	0	0	0	0	0	0	0	0	0	0	0	0	0
AB0161_C	0	0	0	0	0	0	0	0	0	0	0	0	0	0	0	0
AB0162_C	0	0	0	0	0	0	0	0	0	0	0	0	0	0	0	0
AB0164_C	0	0	0	0	0	0	0	0	0	0	0	0	0	0	0	0
AB0165_C	0	0	0	0	0	0	0	0	0	0	0	0	0	0	0	0
AB0166_C	0	0	0	0	0	0	0	0	0	0	0	0	0	0	0	0
AB0167_C	0	0	0	0	0	0	0	0	0	0	0	0	0	0	0	0
AB0169_C	0	0	0	0	0	0	0	0	0	0	0	0	0	0	0	0
AB0170_C	0	0	0	0	0	0	0	0	0	0	0	0	0	0	0	0
AB0171_C	0	0	0	0	0	0	0	0	0	0	0	0	0	0	0	0
AB0172_C	0	0	0	0	0	0	0	0	0	0	0	0	0	0	0	0

	Dup 0	Dup 1	Dup 2	Dup 3	Dup 4	Dup 5	Dup 6	Dup 7	Dup 8	Dup 9	Dup 10	Dup 11	Dup 12	Dup 13	Dup 14	Dup 15
AB0173_C	0	0	0	0	0	0	0	0	0	0	0	0	0	0	0	0
AB0174_C	0	0	0	0	0	0	0	0	0	0	0	0	0	0	0	0
AB0175_C	0	0	0	0	0	0	0	0	0	0	0	0	0	0	0	0
AB0176_C	0	0	0	0	0	0	0	0	0	0	0	0	0	0	0	0
AB0177_C	0	0	0	0	0	0	0	0	0	0	0	0	0	0	0	0
AB0178_C	0	0	0	0	0	0	0	0	0	0	0	0	0	0	0	0
AB0179_C	0	0	0	0	0	0	0	0	0	0	0	0	0	0	0	0
AB0181_C	0	0	0	0	0	0	0	1	0	0	1	0	0	0	0	0
AB0182_C	0	0	0	0	0	0	0	1	0	0	0	0	0	0	0	0
AB0183_C	0	0	0	0	1	0	0	1	0	0	0	0	0	0	0	0
AB0184_C	0	0	0	0	0	0	0	1	0	0	0	0	0	0	0	0
AB0185_C	0	0	0	0	0	0	0	2	0	0	0	0	0	0	0	0
AB0186_C	0	0	0	0	0	0	0	1	0	0	0	0	0	0	0	0
AB0187_C	0	0	0	0	0	0	0	0	0	0	1	0	0	0	0	0
AB0188_C	0	1	0	0	0	0	0	0	0	0	0	0	0	0	0	0
AB0189_C	0	0	0	0	0	0	0	2	0	0	0	0	0	0	0	0
AB0190_C	0	0	0	0	0	0	0	0	0	0	2	0	0	0	0	0
AB0191_C	0	0	0	0	0	0	0	0	0	0	1	0	0	0	0	0
AB0192_C	0	0	0	0	0	0	0	0	0	0	1	0	0	0	0	0
AB0195_C	0	0	0	0	0	0	0	1	0	0	0	0	0	0	0	0
AB0196_C	0	0	0	0	0	0	0	0	0	0	1	0	0	0	0	0
AB0197_C	0	0	0	0	0	0	0	0	0	0	0	0	0	0	0	0
AB0198_C	0	0	0	0	0	0	0	0	0	0	0	0	0	0	0	0
AB0199_C	0	0	0	0	0	0	0	0	0	0	0	0	0	0	0	0
AB0200_C	0	0	0	0	0	0	0	0	0	0	0	0	0	0	0	0
AB0201_C	0	0	0	0	0	0	0	0	0	0	0	0	0	0	0	0
AB0202_C	0	0	0	0	0	0	0	0	0	0	0	0	0	0	0	0
AB0203_C	0	0	0	0	0	0	0	0	1	0	0	0	0	0	0	0
AB0204_C	0	0	0	0	0	0	0	1	0	0	1	0	0	0	0	0
AB0205_C	0	0	0	0	0	0	0	0	0	0	0	0	0	0	0	0
AB0206_C	0	0	0	0	0	0	0	0	0	0	0	0	0	0	0	0
AB0207_C	0	0	0	0	0	0	0	0	0	0	0	0	0	0	0	0
AB0208_C	0	0	0	0	0	0	0	0	0	0	0	0	0	0	0	0
AB0209_C	0	0	0	0	0	0	0	1	0	0	1	0	0	0	1	0
AB0210_C	0	0	0	0	0	0	0	0	0	0	0	0	0	0	0	0
AB0211_C	0	0	0	0	0	0	0	0	0	0	0	0	0	0	0	0
AB0212_C	0	0	0	0	0	0	0	2	0	0	0	0	0	0	0	0
AB0213_C	0	0	0	0	0	0	0	0	0	0	2	0	0	0	0	0
AB0215_C	0	0	0	0	0	0	0	0	0	0	0	0	0	0	0	0
AB0217_C	0	0	0	0	0	0	0	0	0	0	0	0	0	0	0	0
AB0218_C	0	0	0	0	0	0	0	0	0	0	0	0	0	0	0	0
AB0219_C	0	0	0	0	0	0	0	0	0	0	1	0	0	0	0	0
AB0221_C	0	0	0	0	0	0	0	1	0	0	0	0	0	0	0	0
AB0222_C	0	0	0	0	0	0	0	0	0	0	1	0	0	0	0	0
AB0223_C	0	0	0	0	0	0	0	0	0	0	1	0	0	0	0	0
AB0224_C	0	0	0	0	0	0	0	1	0	0	0	0	0	0	0	0
AB0226_C	0	0	0	0	0	0	0	1	0	0	0	0	0	0	0	0
AB0227_C	0	0	0	0	0	0	0	0	0	0	0	0	0	0	0	0
AB0228_C	0	0	0	0	0	0	0	0	1	0	0	0	0	0	0	0
AB0229_C	0	1	0	0	0	0	0	0	0	0	1	0	0	0	0	0
AB0231_C	0	0	0	0	0	0	0	0	0	0	0	0	0	0	0	0
AB0232_C	0	0	0	0	0	0	0	0	0	0	0	0	0	0	0	0
AB0233_C	0	0	0	0	0	0	0	0	0	0	0	0	0	0	0	0
AB0234_C	0	0	0	0	0	0	0	0	0	0	2	0	0	0	0	0
AB0235_C	0	0	0	0	0	0	0	0	0	0	0	0	0	0	0	0
AB0236_C	0	0	0	0	0	0	0	0	0	0	0	0	0	0	0	0
AB0237_C	0	0	0	0	0	0	0	1	0	0	1	0	0	0	0	0
AB0238_C	0	0	0	0	0	0	0	0	0	0	0	0	0	0	0	0
AB0239_C	0	0	0	0	0	0	0	0	0	0	0	0	0	0	0	0
AB0240_C	0	0	0	0	0	0	0	2	0	0	0	0	0	0	0	0
AB0241_C	0	0	0	0	0	0	0	0	0	0	0	0	0	0	0	0
AB0242_C	0	1	0	0	0	0	0	0	0	0	0	0	0	0	0	0
AB0243_C	0	0	0	0	0	0	0	0	0	0	1	0	0	0	0	0
AB0244_C	0	0	0	0	0	0	0	0	0	0	0	0	0	0	0	0
AB0246_C	0	1	0	0	0	0	0	0	0	0	1	0	0	0	0	0
AB0247_C	0	0	0	0	0	0	0	0	0	0	1	0	0	0	0	0
AB0248_C	0	0	0	0	0	0	0	1	0	0	0	0	0	0	0	0
AB0249_C	0	0	0	0	0	0	0	2	0	0	0	0	0	0	0	0

	Dup 0	Dup 1	Dup 2	Dup 3	Dup 4	Dup 5	Dup 6	Dup 7	Dup 8	Dup 9	Dup 10	Dup 11	Dup 12	Dup 13	Dup 14	Dup 15
AB0250_C	0	0	0	0	0	0	0	0	0	0	1	0	0	0	0	0
AB0251_C	0	0	0	0	0	0	0	0	0	0	0	0	0	0	0	0
AB0252_C	0	0	1	0	0	0	0	0	0	0	0	0	0	0	0	0
AB0253_C	0	0	0	0	0	0	0	0	0	0	0	0	0	0	0	0
AB0255_C	0	0	0	0	0	0	0	0	0	0	0	0	0	0	0	0
AB0256_C	0	0	0	0	0	0	0	0	0	0	0	0	0	0	0	0
AB0257_C	0	0	0	0	1	0	0	0	0	0	0	0	0	0	0	0
AB0258_C	0	0	0	0	0	0	0	0	0	0	1	0	0	0	0	0
AB0260_C	0	0	0	0	0	0	0	0	0	0	0	0	0	0	0	0
AB0261_C	0	0	0	0	0	0	0	0	0	0	0	0	0	0	0	0
AB0262_C	0	0	0	0	0	0	0	0	0	0	1	0	0	0	0	0
AB0263_C	0	0	0	0	0	0	0	1	0	0	0	0	0	0	0	0
AB0264_C	0	0	0	0	0	0	0	0	0	0	0	0	0	0	0	0
AB0265_C	0	0	0	0	0	0	0	0	0	0	0	0	0	0	0	0
AB0266_C	0	0	0	0	0	0	0	0	0	0	1	0	0	0	0	0
AB0267_C	0	0	0	0	0	0	0	2	0	0	0	0	0	0	0	0
AB0268_C	0	0	0	0	0	0	0	0	0	0	0	0	0	0	0	0
AB0270_C	0	0	0	0	0	0	0	0	0	0	0	0	0	0	0	0
AB0271_C	0	0	0	0	0	0	0	0	0	0	0	0	0	0	0	0
AB0272_C	0	0	0	0	0	0	0	0	0	0	0	0	0	0	0	0
AB0273_C	0	0	0	0	0	0	0	0	0	0	0	0	0	0	0	0
AB0274_C	0	0	0	0	0	0	0	0	0	0	0	0	0	0	0	0
AB0275_C	0	0	0	0	0	0	0	0	0	0	0	0	0	0	0	0
AB0276_C	0	0	0	0	0	0	0	1	0	0	1	0	0	0	0	0
AB0277_C	0	0	0	0	0	0	0	0	0	0	0	0	0	0	0	0
AB0278_C	0	0	0	0	0	0	0	0	0	0	0	0	0	0	0	0
AB0279_C	0	0	0	0	0	0	0	0	0	0	1	0	0	0	0	0
AB0280_C	0	0	0	0	0	0	0	0	0	0	0	0	0	0	0	0
AB0281_C	0	0	0	0	0	0	0	0	0	0	0	0	0	0	0	0
AB0282_C	0	1	0	0	0	0	0	0	0	0	0	0	0	0	0	0
AB0283_C	0	0	0	0	0	0	0	0	0	0	0	0	0	0	0	0
AB0284_C	0	0	0	0	0	0	0	0	0	0	0	0	0	0	0	0
AC0089_C	0	1	0	0	0	0	0	0	0	0	0	0	0	0	0	0
AC0090_C	0	0	0	0	0	0	0	0	0	0	0	0	0	0	0	0
AC0091_C	0	0	0	0	0	0	0	0	0	0	0	0	0	0	0	0
AC0092_C	0	2	0	0	0	0	0	0	0	0	0	0	0	0	0	0
AC0093_C	0	1	0	0	0	0	0	0	0	0	0	0	0	0	0	0
AC0094_C	0	0	0	0	0	0	0	0	0	0	0	0	0	0	0	0
AC0095_C	0	2	0	0	0	0	0	0	0	0	0	0	0	0	0	0
AC0096_C	0	0	0	0	0	0	0	0	0	0	0	0	0	0	0	0
AC0097_C	0	1	0	0	0	0	0	0	0	0	0	0	0	0	0	0
AC0098_C	0	1	0	0	0	0	0	0	0	0	0	0	0	0	0	0
AC0099_C	0	0	0	0	0	0	0	0	0	0	0	0	0	0	0	0
AC0100_C	0	1	0	0	0	0	0	0	0	0	0	0	0	0	0	0
AC0101_C	0	0	0	0	0	0	0	0	0	0	0	0	0	0	0	0
AC0102_C	0	1	0	0	0	0	0	0	0	0	0	0	0	0	0	0
AC0103_C	0	1	0	0	0	0	0	0	0	0	0	0	0	0	0	0
AC0104_C	0	1	0	0	0	0	0	0	0	0	0	0	0	0	0	0
AC0105_C	0	2	0	0	0	0	0	0	0	0	0	0	0	0	0	0
AC0106_C	0	0	0	0	0	0	0	0	0	0	0	0	0	0	0	0
AC0107_C	0	0	0	0	0	0	0	0	0	0	0	0	0	0	0	0
AC0108_C	0	1	0	0	0	0	0	0	0	0	0	0	0	0	0	0
AC0109_C	0	0	0	0	0	1	0	0	0	0	0	0	0	0	0	0
AC0110_C	0	1	0	0	0	0	0	0	0	0	0	0	0	0	0	0
AC0111_C	0	1	0	0	0	0	0	0	0	0	0	0	0	0	0	0
AC0112_C	0	0	0	0	0	0	0	0	0	0	0	0	0	0	0	0
AC0113_C	0	1	0	0	0	0	0	0	0	0	0	0	0	0	0	0
AC0114_C	0	1	0	0	0	0	0	0	0	0	0	0	0	0	0	0
AC0115_C	0	0	0	0	0	0	0	0	0	0	0	0	0	0	0	0
AC0116_C	0	0	0	0	0	0	0	0	0	0	0	0	0	0	0	0
AC0117_C	0	0	0	0	0	0	0	0	0	0	0	0	1	0	0	0
AC0118_C	0	1	0	0	0	0	0	0	0	0	0	0	0	0	0	0
AC0119_C	0	0	0	0	0	0	0	0	0	1	0	0	0	0	0	0
AC0120_C	0	1	0	0	0	0	0	0	0	0	0	0	0	0	0	0
AC0121_C	0	0	0	0	0	0	0	0	0	0	0	0	0	0	0	0
AC0122_C	0	0	0	0	0	0	0	0	0	0	0	0	0	0	0	0
AC0123_C	0	0	0	0	0	0	0	0	0	0	0	0	0	0	0	0
AC0124_C	0	1	0	0	0	0	0	0	0	0	0	0	0	0	0	0

	Dup 0	Dup 1	Dup 2	Dup 3	Dup 4	Dup 5	Dup 6	Dup 7	Dup 8	Dup 9	Dup 10	Dup 11	Dup 12	Dup 13	Dup 14	Dup 15
AC0125_C	0	2	0	0	0	0	0	0	0	0	0	0	0	0	0	0
AC0126_C	0	1	0	0	0	0	0	0	0	0	0	0	0	0	0	0
AC0127_C	0	0	0	0	0	0	0	0	0	0	0	0	0	0	0	0
AC0128_C	0	0	0	0	0	0	0	0	0	0	0	0	0	0	0	0
AC0129_C	0	1	0	0	0	0	0	0	0	0	0	0	0	0	0	0
AC0130_C	0	0	0	0	0	0	0	0	0	0	0	0	0	0	0	0
AC0131_C	0	0	0	0	0	0	0	0	0	0	0	0	0	0	0	0
AC0132_C	0	1	0	0	0	0	0	0	0	0	0	0	0	0	0	0
AC0133_C	0	0	0	0	0	0	0	0	0	0	0	0	0	0	0	0
AC0134_C	0	0	0	0	0	0	0	0	0	0	0	0	0	0	0	0
AC0135_C	0	1	0	0	0	0	0	0	0	0	0	0	0	0	0	0
AC0136_C	0	1	0	0	0	0	0	0	0	0	0	0	0	0	0	0
AC0137_C	0	0	0	0	0	0	0	0	0	0	0	0	0	0	0	0
AC0138_C	0	1	0	0	0	0	0	0	0	0	0	0	0	0	0	0
AC0139_C	0	1	0	0	0	0	0	0	0	0	0	0	0	0	0	0
AC0140_C	0	2	0	0	0	0	0	0	0	0	0	0	0	0	0	0
AC0141_C	0	0	0	0	0	0	0	0	0	0	0	0	0	0	0	0
AC0142_C	0	1	0	0	0	0	0	0	0	0	0	0	0	0	0	0
AC0143_C	0	1	0	0	0	0	0	0	0	0	0	0	0	0	0	0
AC0144_C	0	1	0	0	0	0	0	0	0	0	0	0	0	0	0	0
AC0145_C	0	1	0	0	0	0	0	0	0	0	0	0	0	0	0	0
AC0146_C	0	0	0	0	0	1	0	0	0	0	0	0	0	0	0	0
AC0147_C	0	0	0	0	0	0	0	0	0	0	0	0	0	0	0	0
AC0148_C	0	1	0	0	0	0	0	0	0	0	0	0	0	0	0	0
AC0149_C	0	1	0	0	0	0	0	0	0	0	0	0	0	0	0	0
AC0150_C	0	1	0	0	0	0	0	0	0	0	0	0	0	0	0	0
AC0151_C	0	1	0	0	0	0	0	0	0	0	0	0	0	0	0	0
AC0152_C	0	0	0	0	0	0	0	0	0	0	0	0	0	0	0	0
AC0153_C	0	0	0	0	0	0	0	0	0	0	0	0	0	0	0	0
AC0154_C	0	0	0	0	0	0	0	0	0	0	0	0	0	0	0	0
AC0155_C	0	0	0	0	0	0	0	0	0	0	0	0	0	0	0	0
AC0156_C	0	1	0	0	0	0	0	0	0	0	0	0	0	0	0	0
AC0157_C	0	0	0	0	0	0	0	0	0	0	0	0	0	0	0	0
AC0158_C	0	0	0	0	0	0	0	0	0	0	0	0	0	0	0	0
AC0159_C	0	0	0	0	0	0	0	0	0	0	0	0	0	0	0	0
AC0160_C	0	1	0	0	0	0	0	0	0	0	0	0	0	0	0	0
AC0161_C	0	0	0	0	0	0	0	0	0	0	0	0	0	0	0	0
AC0162_C	0	1	0	0	0	0	0	0	0	0	0	0	0	0	0	0
AC0163_C	0	1	0	0	0	0	0	0	0	0	0	0	0	0	0	0
AC0164_C	0	1	0	0	0	0	0	0	0	0	0	0	0	0	0	0
AC0166_C	0	2	0	0	0	0	0	0	0	0	0	0	0	0	0	0
AC0167_C	0	0	0	0	0	0	0	0	0	0	0	0	0	0	0	0
AC0168_C	0	2	0	0	0	0	0	0	0	0	0	0	0	0	0	0
AC0169_C	0	1	0	0	0	0	0	0	0	0	0	0	0	0	0	0
AC0170_C	0	0	0	0	0	0	0	0	0	0	0	0	0	0	0	0
AC0171_C	0	2	0	0	0	0	0	0	0	0	0	0	0	0	0	0
AC0172_C	0	1	0	0	0	0	0	0	0	0	0	0	0	0	0	0
AC0173_C	0	1	0	0	0	0	0	0	0	0	0	0	0	0	0	0
AC0174_C	0	1	0	0	0	0	0	0	0	0	0	0	0	0	0	0
AC0176_C	0	0	0	0	0	0	0	0	0	0	0	0	0	0	0	0
AC0177_C	0	2	0	0	0	0	0	0	0	0	0	0	0	0	0	0
AC0178_C	0	0	0	0	0	0	0	0	0	0	0	0	0	0	0	0
AC0179_C	0	0	0	0	0	0	0	0	0	0	0	0	0	0	0	0
AC0180_C	0	0	0	0	0	0	0	0	0	0	0	0	0	0	0	0
AC0181_C	0	1	0	0	0	0	0	0	0	0	0	0	0	0	0	0
AC0182_C	0	0	0	0	0	0	0	0	0	0	0	0	0	0	0	0
AC0183_C	0	1	0	0	0	0	0	0	0	0	0	0	0	0	0	0
AC0184_C	0	1	0	0	0	0	0	0	0	0	0	0	0	0	0	0
AC0185_C	0	0	0	0	0	1	0	0	0	0	0	0	0	0	0	0
AC0186_C	0	0	0	0	0	1	0	0	0	0	0	0	0	0	0	0
AC0187_C	0	2	0	0	0	0	0	0	0	0	0	0	0	0	0	0
AC0188_C	0	1	0	0	0	0	0	0	0	0	0	0	0	0	0	0
AC0189_C	0	1	0	0	0	0	0	0	0	0	0	0	0	0	0	0
AC0190_C	0	3	0	0	0	0	0	0	0	0	0	0	0	0	0	0
AC0191_C	0	1	0	0	0	0	0	0	0	0	0	0	0	0	0	0
AC0192_C	0	0	0	0	0	1	0	0	0	0	0	0	0	0	0	0
AC0193_C	0	NA	0	0	0	0	0	0	0	0	0	0	0	0	0	0
AC0194_C	0	1	0	0	0	0	0	0	0	0	0	0	0	0	0	0

	Dup 0	Dup 1	Dup 2	Dup 3	Dup 4	Dup 5	Dup 6	Dup 7	Dup 8	Dup 9	Dup 10	Dup 11	Dup 12	Dup 13	Dup 14	Dup 15
AC0195_C	0	1	0	0	0	0	0	0	0	0	0	0	0	0	0	0
AC0196_C	0	1	0	0	0	0	0	0	0	0	0	0	0	0	0	0
AC0197_C	0	1	0	0	0	0	0	0	0	0	0	0	0	0	0	0
AC0199_C	0	1	0	0	0	0	0	0	0	0	0	0	0	0	0	0
AC0200_C	0	0	0	0	0	0	0	0	0	0	0	0	0	0	0	0
AC0201_C	0	0	0	0	0	0	0	0	0	0	0	0	0	0	0	0
AC0202_C	0	0	0	0	0	0	0	0	0	0	0	0	0	0	0	0
AC0203_C	0	1	0	0	0	0	0	0	0	0	0	0	0	0	0	0
AG0082_C	0	0	0	0	0	0	0	0	0	0	0	0	0	0	0	0
AG0085_C	0	0	0	0	0	0	0	0	0	0	0	0	0	0	0	0
AG0089_C	0	0	0	0	0	0	0	0	0	0	0	0	0	0	0	0
AG0096_C	0	0	0	0	0	0	0	0	0	0	0	0	0	0	0	0
AG0097_C	0	0	0	0	0	0	0	0	0	0	0	0	0	0	0	0
AG0098_C	0	0	0	0	0	0	0	0	0	0	0	0	0	0	0	0
AG0100_C	0	0	0	0	0	0	0	0	0	0	0	0	0	0	0	0
AG0102_C	0	0	0	0	0	0	0	0	0	0	0	0	0	0	0	0
AG0104_C	0	0	0	0	0	0	0	0	0	0	0	0	0	0	0	0
AG0106_C	0	0	0	0	0	0	0	0	0	0	0	0	0	0	0	0
AG0108_C	0	0	0	0	0	0	0	0	0	0	0	0	0	0	0	0
AG0109_C	0	0	0	0	0	0	0	0	0	0	0	0	0	0	0	0
AG0111_C	0	0	0	0	0	0	0	0	0	0	0	0	0	0	0	0
AG0118_C	0	0	0	0	0	0	0	0	0	0	0	0	0	0	0	0
AG0120_C	0	0	0	0	0	0	0	0	0	0	0	0	0	0	0	0
AG0121_C	0	0	0	0	0	0	0	0	0	0	0	0	0	0	0	0
AG0123_C	0	0	0	0	0	0	0	0	0	0	0	0	0	0	0	0
AG0125_C	0	0	0	0	0	0	0	0	0	0	0	0	0	0	0	0
AG0126_C	0	0	0	0	0	0	0	0	0	0	0	0	0	0	0	0
AG0127_C	0	0	0	0	0	0	0	0	0	0	0	0	0	0	0	0
AG0128_C	0	0	0	0	0	0	0	0	0	0	0	0	0	0	0	0
AG0129_C	0	0	0	0	0	0	0	0	0	0	0	0	0	0	0	0
AG0133_C	0	0	0	0	0	0	0	0	0	0	0	0	0	0	0	0
AG0134_C	0	0	0	0	0	0	0	0	0	0	0	0	0	0	0	0
AG0136_C	0	0	0	0	0	0	0	0	0	0	0	0	0	0	0	0
AG0137_C	0	0	0	0	0	0	0	0	0	0	0	0	0	0	0	0
AG0138_C	0	0	0	0	0	0	0	0	0	0	0	0	0	0	0	0
AG0139_C	0	0	0	0	0	0	0	0	0	0	0	0	0	0	0	0
AG0141_C	0	0	0	0	0	0	0	0	0	0	0	0	0	0	0	0
AG0142_C	0	0	0	0	0	0	0	0	0	0	0	0	0	0	0	0
AG0143_C	0	0	0	0	0	0	0	0	0	0	0	0	0	0	0	0
AG0144_C	0	0	0	0	0	0	0	0	0	0	0	0	0	0	0	0
AG0145_C	0	0	0	0	0	0	0	0	0	0	0	0	0	0	0	0
AG0146_C	0	0	0	0	0	0	0	0	0	0	0	0	0	0	0	0
AG0147_C	0	0	0	0	0	0	0	0	0	0	0	0	0	0	0	0
AG0148_C	0	0	0	0	0	0	0	0	0	0	0	0	0	0	0	0
AG0152_C	0	0	0	0	0	0	0	0	0	0	0	0	0	0	0	0
AG0153_C	0	0	0	0	0	0	0	0	0	0	0	0	0	0	0	0
AG0156_C	0	0	0	0	0	0	0	0	0	0	0	0	0	0	0	0
AG0159_C	0	0	0	0	0	0	0	0	0	0	0	0	0	0	0	0
AG0162_C	0	0	0	0	0	0	0	0	0	0	0	0	0	0	0	0
AG0163_C	0	0	0	0	0	0	0	0	0	0	0	0	0	0	0	0
AG0169_C	0	0	0	0	0	0	0	0	0	0	0	0	0	0	0	0
AG0170_C	0	0	0	0	0	0	0	0	0	0	0	0	0	0	0	0
AG0172_C	0	0	0	0	0	0	0	0	0	0	0	0	0	0	0	0
AG0178_C	0	0	0	0	0	0	0	0	0	0	0	0	0	0	0	0
AG0179_C	0	0	0	0	0	0	0	0	0	0	0	0	0	0	0	0
AG0181_C	0	0	0	0	0	0	0	0	0	0	0	0	0	0	0	0
AG0183_C	0	0	0	0	0	0	0	0	0	0	0	0	0	0	0	0
AG0195_C	0	0	0	0	0	0	0	0	0	0	0	0	0	0	0	0
AG0197_C	0	0	0	0	0	0	0	0	0	0	0	0	0	0	0	0
AG0202_C	0	0	0	0	0	0	0	0	0	0	0	0	0	0	0	0
AG0203_C	0	0	0	0	0	0	0	0	0	0	0	0	0	0	0	0
AG0204_C	0	0	0	0	0	0	0	0	0	0	0	0	0	0	0	0
AG0206_C	0	0	0	0	0	0	0	0	0	0	0	0	0	0	0	0
AG0208_C	0	0	0	0	0	0	0	0	0	0	0	0	0	0	0	0
AG0214_C	0	0	0	0	0	0	0	0	0	0	0	0	0	0	0	0
AG0221_C	0	0	0	0	0	0	0	0	0	0	0	0	0	0	0	0
AG0223_C	0	0	0	0	0	0	0	0	0	0	0	0	0	0	0	0
AG0227_C	0	0	0	0	0	0	0	0	0	0	0	0	0	0	0	0

	Dup 0	Dup 1	Dup 2	Dup 3	Dup 4	Dup 5	Dup 6	Dup 7	Dup 8	Dup 9	Dup 10	Dup 11	Dup 12	Dup 13	Dup 14	Dup 15
AG0229_C	0	0	0	0	0	0	0	0	0	0	0	0	0	0	0	0
AG0230_C	0	0	0	0	0	0	0	0	0	0	0	0	0	0	0	0
AG0231_C	0	0	0	0	0	0	0	0	0	0	0	0	0	0	0	0
AG0232_C	0	0	0	0	0	0	0	0	0	0	0	0	0	0	0	0
AG0263_C	0	0	0	0	0	0	0	0	0	0	0	0	0	0	0	0
AJ0023_C	0	0	0	0	0	0	0	0	0	0	0	0	0	0	0	0
AJ0024_C	0	0	0	0	0	0	0	0	0	0	0	0	0	0	0	0
AJ0028_C	0	0	0	0	0	0	0	0	0	0	0	0	0	0	0	0
AJ0032_C	0	0	0	0	0	0	0	0	0	0	0	0	0	0	0	0
AJ0035_C	0	0	0	0	0	0	0	0	0	0	0	0	0	0	0	0
AJ0036_C	0	0	0	0	0	0	0	0	0	0	0	0	0	0	0	0
AJ0037_C	0	0	0	0	0	0	0	0	0	0	0	0	0	0	0	0
AJ0038_C	0	0	0	0	0	0	0	0	0	0	0	0	0	0	0	0
AJ0039_C	0	0	0	0	0	0	0	0	0	0	0	0	0	0	0	0
AJ0043_C	0	0	0	0	0	0	0	0	0	0	0	0	0	0	0	0
AJ0044_C	0	0	0	0	0	0	0	0	0	0	0	0	0	0	0	0
AJ0045_C	0	0	0	0	0	0	0	0	0	0	0	0	0	0	0	0
AJ0047_C	0	0	0	0	0	0	0	0	0	0	0	0	0	0	0	0
AJ0051_C	0	0	0	0	0	0	0	0	0	0	0	0	0	0	0	0
AJ0052_C	0	0	0	0	0	0	0	0	0	0	0	0	0	0	0	0
AJ0056_C	0	0	0	0	0	0	0	0	0	0	0	0	0	0	0	0
AJ0059_C	0	0	0	0	0	0	0	0	0	0	0	0	0	0	0	0
AJ0060_C	0	0	0	0	0	0	0	0	0	0	0	0	0	0	0	0
AJ0061_C	0	0	0	0	0	0	0	0	0	0	0	0	0	0	0	0
AJ0063_C	0	0	0	0	0	0	0	0	0	0	0	0	0	0	0	0
AJ0064_C	0	0	0	0	0	0	0	0	0	0	0	0	0	0	0	0
AJ0066_C	0	0	0	0	0	0	0	0	0	0	0	0	0	0	0	0
AJ0068_C	0	0	0	0	0	0	0	0	0	0	0	0	0	0	0	0
AJ0070_C	0	0	0	0	0	0	0	0	0	0	0	0	0	0	0	0
AJ0071_C	0	0	0	0	0	0	0	0	0	0	0	0	0	0	0	0
AJ0072_C	0	0	0	0	0	0	0	0	0	0	0	0	0	0	0	0
AJ0074_C	0	0	0	0	0	0	0	0	0	0	0	0	0	0	0	0
AJ0075_C	0	0	0	0	0	0	0	0	0	0	0	0	0	0	0	0
AJ0076_C	0	0	0	0	0	0	0	0	0	0	0	0	0	0	0	0
AJ0077_C	0	0	0	0	0	0	0	0	0	0	0	0	0	0	0	0
AJ0078_C	0	0	0	0	0	0	0	0	0	0	0	0	0	0	0	0
AJ0080_C	0	0	0	0	0	0	0	0	0	0	0	0	0	0	0	0
AJ0081_C	0	0	0	0	0	0	0	0	0	0	0	0	0	0	0	0
AJ0084_C	0	0	0	0	0	0	0	0	0	0	0	0	0	0	0	0
AJ0085_C	0	0	0	0	0	0	0	0	0	0	0	0	0	0	0	0
AJ0086_C	0	0	0	0	0	0	0	0	0	0	0	0	0	0	0	0
AJ0087_C	0	0	0	0	0	0	0	0	0	0	0	0	0	0	0	0
AJ0088_C	0	0	0	0	0	0	0	0	0	0	0	0	0	0	0	0
AJ0090_C	0	0	0	0	0	0	0	0	0	0	0	0	0	0	0	0
AJ0092_C	0	0	0	0	0	0	0	0	0	0	0	0	0	0	0	0
AJ0093_C	0	0	0	0	0	0	0	0	0	0	0	0	0	0	0	0
AJ0095_C	0	0	0	0	0	0	0	0	0	0	0	0	0	0	0	0
AJ0096_C	0	0	0	0	0	0	0	0	0	0	0	0	0	0	0	0
AJ0097_C	0	0	0	0	0	0	0	0	0	0	0	0	0	0	0	0
AJ0098_C	0	0	0	0	0	0	0	0	0	0	0	0	0	0	0	0
AJ0099_C	0	0	0	0	0	0	0	0	0	0	0	0	0	0	0	0
AJ0100_C	0	0	0	0	0	0	0	0	0	0	0	0	0	0	0	0
AJ0101_C	0	0	0	0	0	0	0	0	0	0	0	0	0	0	0	0
AJ0102_C	0	0	0	0	0	0	0	0	0	0	0	0	0	0	0	0
AJ0103_C	0	0	0	0	0	0	0	0	0	0	0	0	0	0	0	0
AJ0105_C	0	0	0	0	0	0	0	0	0	0	0	0	0	0	0	0
AJ0107_C	0	0	0	0	0	0	0	0	0	0	0	0	0	0	0	0
AJ0109_C	0	0	0	0	0	0	0	0	0	0	0	0	0	0	0	0
AJ0113_C	0	0	0	0	0	0	0	0	0	0	0	0	0	0	0	0
AJ0115_C	0	0	0	0	0	0	0	0	0	0	0	0	0	0	0	0
AJ0116_C	0	0	0	0	0	0	0	0	0	0	0	0	0	0	0	0
AJ0117_C	0	0	0	0	0	0	0	0	0	0	0	0	0	0	0	0
AJ0119_C	0	0	0	0	0	0	0	0	0	0	0	0	0	0	0	0
AJ0128_C	0	0	0	0	0	0	0	0	0	0	0	0	0	0	0	0
AJ0129_C	0	0	0	0	0	0	0	0	0	0	0	0	0	0	0	0
AJ0130_C	0	0	0	0	0	0	0	0	0	0	0	0	0	0	0	0
AJ0131_C	0	0	0	0	0	0	0	0	0	0	0	0	0	0	0	0
AJ0132_C	0	0	0	0	0	0	0	0	0	0	0	0	0	0	0	0

	Dup 0	Dup 1	Dup 2	Dup 3	Dup 4	Dup 5	Dup 6	Dup 7	Dup 8	Dup 9	Dup 10	Dup 11	Dup 12	Dup 13	Dup 14	Dup 15
AJ0133_C	0	0	0	0	0	0	0	0	0	0	0	0	0	0	0	0
AJ0134_C	0	0	0	0	0	0	0	0	0	0	0	0	0	0	0	0
AJ0135_C	0	0	0	0	0	0	0	0	0	0	0	0	0	0	0	0
AJ0136_C	0	0	0	0	0	0	0	0	0	0	0	0	0	0	0	0
AJ0137_C	0	0	0	0	0	0	0	0	0	0	0	0	0	0	0	0
AJ0138_C	0	0	0	0	0	0	0	0	0	0	0	0	0	0	0	0
AJ0139_C	0	0	0	0	0	0	0	0	0	0	0	0	0	0	0	0
AJ0140_C	0	0	0	0	0	0	0	0	0	0	0	0	0	0	0	0
AJ0141_C	0	0	0	0	0	0	0	0	0	0	0	0	0	0	0	0
AJ0142_C	0	0	0	0	0	0	0	0	0	0	0	0	0	0	0	0
AJ0143_C	0	0	0	0	0	0	0	0	0	0	0	0	0	0	0	0
AJ0144_C	0	0	0	0	0	0	0	0	0	0	0	0	0	0	0	0
AJ0145_C	0	0	0	0	0	0	0	0	0	0	0	0	0	0	0	0
AJ0146_C	0	0	0	0	0	0	0	0	0	0	0	0	0	0	0	0
AJ0147_C	0	0	0	0	0	0	0	0	0	0	0	0	0	0	0	0
AJ0148_C	0	0	0	0	0	0	0	0	0	0	0	0	0	0	0	0
AJ0149_C	0	0	0	0	0	0	0	0	0	0	0	0	0	0	0	0
AJ0150_C	0	0	0	0	0	0	0	0	0	0	0	0	0	0	0	0
AJ0151_C	0	0	0	0	0	0	0	0	0	0	0	0	0	0	0	0
AJ0152_C	0	0	0	0	0	0	0	0	0	0	0	0	0	0	0	0
AJ0153_C	0	0	0	0	0	0	0	0	0	0	0	0	0	0	0	0
AJ0154_C	0	0	0	0	0	0	0	0	0	0	0	0	0	0	0	0
AJ0155_C	0	0	0	0	0	0	0	0	0	0	0	0	0	0	0	0
AJ0156_C	0	0	0	0	0	0	0	0	0	0	0	0	0	0	0	0
AJ0157_C	0	0	0	0	0	0	0	0	0	0	0	0	0	0	0	0
AJ0158_C	0	0	0	0	0	0	0	0	0	0	0	0	0	0	0	0
AJ0159_C	0	0	0	0	0	0	0	0	0	0	0	0	0	0	0	0
AJ0161_C	0	0	0	0	0	0	0	0	0	0	0	0	0	0	0	0
AK0060_C	0	0	0	0	0	0	0	0	0	0	0	0	0	0	0	0
AK0062_C	0	0	0	0	0	0	0	0	0	0	0	0	0	0	0	0
AK0065_C	0	0	0	0	0	0	0	0	0	0	0	0	0	0	0	0
AK0066_C	0	0	0	0	0	0	0	0	0	0	0	0	0	0	0	0
AK0067_C	0	0	0	0	0	0	0	0	0	0	0	0	0	0	0	0
AK0068_C	0	0	0	0	0	0	0	0	0	0	0	0	0	0	0	0
AK0069_C	0	0	0	0	0	0	0	0	0	0	0	0	0	0	0	0
AK0070_C	0	0	0	0	0	0	0	0	0	0	0	0	0	0	0	0
AK0072_C	0	0	0	0	0	0	0	0	0	0	0	0	0	0	0	0
AK0073_C	0	0	0	0	0	0	0	0	0	0	0	0	0	0	0	0
AK0074_C	0	0	0	0	0	0	0	0	0	0	0	0	0	0	0	0
AK0075_C	0	0	0	0	0	0	0	0	0	0	0	0	0	0	0	0
AK0076_C	0	0	0	0	0	0	0	0	0	0	0	0	0	0	0	0
AK0077_C	0	0	0	0	0	0	0	0	0	0	0	0	0	0	0	0
AK0078_C	0	0	0	0	0	0	0	0	0	0	0	0	0	0	0	0
AK0079_C	0	0	0	0	0	0	0	0	0	0	0	0	0	0	0	0
AK0080_C	0	0	0	0	0	0	0	0	0	0	0	0	0	0	0	0
AK0081_C	0	0	0	0	0	0	0	0	0	0	0	0	0	0	0	0
AK0082_C	0	0	0	0	0	0	0	0	0	0	0	0	0	0	0	0
AK0085_C	0	0	0	0	0	0	0	0	0	0	0	0	0	0	0	0
AK0086_C	0	0	0	0	0	0	0	0	0	0	0	0	0	0	0	0
AK0087_C	0	0	0	0	0	0	0	0	0	0	0	0	0	0	0	0
AK0088_C	0	0	0	0	0	0	0	0	0	0	0	0	0	0	0	0
AK0089_C	0	0	0	0	0	0	0	0	0	0	0	0	0	0	0	0
AK0090_C	0	0	0	0	0	0	0	0	0	0	0	0	0	0	0	0
AK0091_C	0	0	0	0	0	0	0	0	0	0	0	0	0	0	0	0
AK0092_C	0	0	0	0	0	0	0	0	0	0	0	0	0	0	0	0
AK0093_C	0	0	0	0	0	0	0	0	0	0	0	0	0	0	0	0
AK0094_C	0	0	0	0	0	0	0	0	0	0	0	0	0	0	0	0
AK0095_C	0	0	0	0	0	0	0	0	0	0	0	0	0	0	0	0
AK0096_C	0	0	0	0	0	0	0	0	0	0	0	0	0	0	0	0
AK0098_C	0	0	0	0	0	0	0	0	0	0	0	0	0	0	0	0
AK0099_C	0	0	0	0	0	0	0	0	0	0	0	0	0	0	0	0
AK0100_C	0	0	0	0	0	0	0	0	0	0	0	0	0	0	0	0
AK0101_C	0	0	0	0	0	0	0	0	0	0	0	0	0	0	0	0
AK0102_C	0	0	0	0	0	0	0	0	0	0	0	0	0	0	0	0
AK0103_C	0	0	0	0	0	0	0	0	0	0	0	0	0	0	0	0
AK0104_C	0	0	0	0	0	0	0	0	0	0	0	0	0	0	0	0
AK0105_C	0	0	0	0	0	0	0	0	0	0	0	0	0	0	0	0
AK0106_C	0	0	0	0	0	0	0	0	0	0	0	0	0	0	0	0

	Dup 0	Dup 1	Dup 2	Dup 3	Dup 4	Dup 5	Dup 6	Dup 7	Dup 8	Dup 9	Dup 10	Dup 11	Dup 12	Dup 13	Dup 14	Dup 15
AK0107_C	0	0	0	0	0	0	0	0	0	0	0	0	0	0	0	0
AK0108_C	0	0	0	0	0	0	0	0	0	0	0	0	0	0	0	0
AK0109_C	0	0	0	0	0	0	0	0	0	0	0	0	0	0	0	0
AK0110_C	0	0	0	0	0	0	0	0	0	0	0	0	0	0	0	0
AK0116_C	0	0	0	0	0	0	0	0	0	0	0	0	0	0	0	0
AK0117_C	0	0	0	0	0	0	0	0	0	0	0	0	0	0	0	0
AK0119_C	0	0	0	0	0	0	0	0	0	0	0	0	0	0	0	0
AK0127_C	0	0	0	0	0	0	0	0	0	0	0	0	0	0	0	0
AN0007_C	0	0	0	0	0	0	0	0	0	0	0	0	0	0	0	0
AN0008_C	0	0	0	0	0	0	0	0	0	0	0	0	0	0	0	0
AN0009_C	0	0	0	0	0	0	0	0	0	0	0	0	0	0	0	0
AN0010_C	0	0	0	0	0	0	0	0	0	0	0	0	0	0	0	0
AN0011_C	0	0	0	0	0	0	0	0	0	0	0	0	0	0	0	0
AN0012_C	0	0	0	0	0	0	0	0	0	0	0	0	0	0	0	0
AN0013_C	0	0	0	0	0	0	0	0	0	0	0	0	0	0	0	0
AN0014_C	0	0	0	0	0	0	0	0	0	0	0	0	0	0	0	0
AN0015_C	0	0	0	0	0	0	0	0	0	0	0	0	0	0	0	0
AN0016_C	0	0	0	0	0	0	0	0	0	0	0	0	0	0	0	0
AN0017_C	0	0	0	0	0	0	0	0	0	0	0	0	0	0	0	0
AN0018_C	0	0	0	0	0	0	0	0	0	0	0	0	0	0	0	0
AN0019_C	0	0	0	0	0	0	0	0	0	0	0	0	0	0	0	0
AN0020_C	0	0	0	0	0	0	0	0	0	0	0	0	0	0	0	0
AN0022_C	0	0	0	0	0	0	0	0	0	0	0	0	0	0	0	0
AN0023_C	0	0	0	0	0	0	0	0	0	0	0	0	0	0	0	0
AN0024_C	0	0	0	0	0	0	0	0	0	0	0	0	0	0	0	0
AN0025_C	0	0	0	0	0	0	0	0	0	0	0	0	0	0	0	0
AN0026_C	0	0	0	0	0	0	0	0	0	0	0	0	0	0	0	0
AN0027_C	0	0	0	0	0	0	0	0	0	0	0	0	0	0	0	0
AN0028_C	0	0	0	0	0	0	1	0	0	0	0	0	0	0	0	0
AN0029_C	0	0	0	0	0	0	0	0	0	0	0	0	0	0	0	0
AN0030_C	0	0	0	0	0	0	0	0	0	0	0	0	0	0	0	0
AN0031_C	0	0	0	0	0	0	0	0	0	0	0	0	0	0	0	0
AN0032_C	0	0	0	0	0	0	0	0	0	0	0	0	0	0	0	0
AN0033_C	0	0	0	0	0	0	0	0	0	0	0	0	0	0	0	0
AN0034_C	0	0	0	0	0	0	0	0	0	0	0	0	0	0	0	0
AN0035_C	0	0	0	0	0	0	0	0	0	0	0	0	0	0	0	0
AN0036_C	0	0	0	0	0	0	0	0	0	0	0	0	0	0	0	0
AN0037_C	0	0	0	0	0	0	0	0	0	0	0	0	0	0	0	0
AN0038_C	0	0	0	0	0	0	0	0	0	0	0	0	0	0	0	0
AN0039_C	0	0	0	0	0	0	0	0	0	0	0	0	0	0	0	0
AN0040_C	0	0	0	0	0	0	0	0	0	0	0	0	0	0	0	0
AN0041_C	0	0	0	0	0	0	0	0	0	0	0	0	0	0	0	0
AN0042_C	0	0	0	0	0	0	0	0	0	0	0	0	0	0	0	0
AN0043_C	0	0	0	0	0	0	0	0	0	0	0	0	0	0	0	0
AN0044_C	0	0	0	0	0	0	0	0	0	0	0	0	0	0	0	0
AN0045_C	0	0	0	0	0	0	0	0	0	0	0	0	0	0	0	0
AN0046_C	0	0	0	0	0	0	0	0	0	0	0	0	0	0	0	0
AN0047_C	0	0	0	0	0	0	0	0	0	0	0	0	0	0	0	0
AN0048_C	0	0	0	0	0	0	0	0	0	0	0	0	0	0	0	0
AN0049_C	0	0	0	0	0	0	0	0	0	0	0	0	0	0	0	0
AN0050_C	0	0	0	0	0	0	0	0	0	0	0	0	0	0	0	0
AN0051_C	0	0	0	0	0	0	0	0	0	0	0	0	0	0	0	0
AN0053_C	0	0	0	0	0	0	0	0	0	0	0	0	0	0	0	0
AN0054_C	0	0	0	0	0	0	0	0	0	0	0	0	0	0	0	0
AN0055_C	0	0	0	0	0	0	0	0	0	0	0	0	0	0	0	0
AN0056_C	0	0	0	0	0	0	0	0	0	0	0	0	0	0	0	0
AN0057_C	0	0	0	0	0	0	0	0	0	0	0	0	0	0	0	0
AN0058_C	0	0	0	0	0	0	0	0	0	0	0	0	0	0	0	0
AN0059_C	0	0	0	0	0	0	0	0	0	0	0	0	0	0	0	0
AN0060_C	0	0	0	0	0	0	0	0	0	0	0	0	0	0	0	0
AN0061_C	0	0	0	0	0	0	0	0	0	0	0	0	0	0	0	0
AN0062_C	0	0	0	0	0	0	0	0	0	0	0	0	0	0	0	0
AN0063_C	0	0	0	0	0	0	0	0	0	0	0	0	0	0	0	0
AN0064_C	0	0	0	0	0	0	0	0	0	0	0	0	0	0	0	0
AN0065_C	0	0	0	0	0	0	0	0	0	0	0	0	0	0	0	0
AN0066_C	0	0	0	0	0	0	0	0	0	0	0	0	0	0	0	0
AN0067_C	0	0	0	0	0	0	0	0	0	0	0	0	0	0	0	0
AN0068_C	0	0	0	0	0	0	0	0	0	0	0	0	0	0	0	0

	Dup 0	Dup 1	Dup 2	Dup 3	Dup 4	Dup 5	Dup 6	Dup 7	Dup 8	Dup 9	Dup 10	Dup 11	Dup 12	Dup 13	Dup 14	Dup 15
AN0069_C	0	0	0	0	0	0	0	0	0	0	0	0	0	0	0	0
AN0070_C	0	0	0	0	0	0	0	0	0	0	0	0	0	0	0	0
AN0071_C	0	0	0	0	0	0	0	0	0	0	0	0	0	0	0	0
AN0072_C	0	0	0	0	0	0	0	0	0	0	0	0	0	0	0	0
AN0073_C	0	0	0	0	0	0	0	0	0	0	0	0	0	0	0	0
AN0074_C	0	0	0	0	0	0	0	0	0	0	0	0	0	0	0	0
AN0075_C	0	0	0	0	0	0	0	0	0	0	0	0	0	0	0	0
AN0076_C	0	0	0	0	0	0	0	0	0	0	0	0	0	0	0	0
AN0077_C	0	0	0	0	0	0	0	0	0	0	0	0	0	0	0	0
AN0079_C	0	0	0	0	0	0	0	0	0	0	0	0	0	0	0	0
AN0080_C	0	0	0	0	0	0	0	0	0	0	0	0	0	0	0	0
AN0081_C	0	0	0	0	0	0	0	0	0	0	0	0	0	0	0	0
AN0082_C	0	0	0	0	0	0	0	0	0	0	0	0	0	0	0	0
AN0083_C	0	0	0	0	0	0	0	0	0	0	0	0	0	0	0	0
AN0084_C	0	0	0	0	0	0	0	0	0	0	0	0	0	0	0	0
AN0085_C	0	0	0	0	0	0	0	0	0	0	0	0	0	0	0	0
AN0086_C	0	0	0	0	0	0	0	0	0	0	0	0	0	0	0	0
AN0087_C	0	0	0	0	0	0	0	0	0	0	0	0	0	0	0	0
AN0088_C	0	0	0	0	0	0	0	0	0	0	0	0	0	0	0	0
AN0089_C	0	0	0	0	0	0	0	0	0	0	0	0	0	0	0	0
AN0090_C	0	0	0	0	0	0	0	0	0	0	0	0	0	0	0	0
AN0091_C	0	0	0	0	0	0	0	0	0	0	0	0	0	0	0	0
AN0092_C	0	0	0	0	0	0	0	0	0	0	0	0	0	0	0	0
AN0093_C	0	0	0	0	0	0	0	0	0	0	0	0	0	0	0	0
AN0094_C	0	0	0	0	0	0	0	0	0	0	0	0	0	0	0	0
AN0095_C	0	0	0	0	0	0	0	0	0	0	0	0	0	0	0	0
AN0096_C	0	0	0	0	0	0	0	0	0	0	0	0	0	0	0	0
AN0097_C	0	0	0	0	0	0	0	0	0	0	0	0	0	0	0	0
AN0098_C	0	0	0	0	0	0	0	0	0	0	0	0	0	0	0	0
AN0099_C	0	0	0	0	0	0	0	0	0	0	0	0	0	0	0	0
AN0100_C	0	0	0	0	0	0	0	0	0	0	0	0	0	0	0	0
AN0101_C	0	0	0	0	0	0	0	0	0	0	0	0	0	0	0	0
AN0102_C	0	0	0	0	0	0	0	0	0	0	0	0	0	0	0	0
AN0103_C	0	0	0	0	0	0	0	0	0	0	0	0	0	0	0	0
AN0104_C	0	0	0	0	0	0	0	0	0	0	0	0	0	0	0	0
AN0105_C	0	0	0	0	0	0	0	0	0	0	0	0	0	0	0	0
AN0106_C	0	0	0	0	0	0	0	0	0	0	0	0	0	0	0	0
AN0107_C	0	0	0	0	0	0	0	0	0	0	0	0	0	0	0	0
AN0108_C	0	0	0	0	0	0	0	0	0	0	0	0	0	0	0	0
AN0109_C	0	0	0	0	0	0	0	0	0	0	0	0	0	0	0	0
AN0111_C	0	0	0	0	0	0	0	0	0	0	0	0	0	0	0	0
AN0112_C	0	0	0	0	0	0	0	0	0	0	0	0	0	0	0	0
AN0113_C	0	0	0	0	0	0	0	0	0	0	0	0	0	0	0	0
AN0114_C	0	0	0	0	0	0	0	0	0	0	0	0	0	0	0	0
AN0115_C	0	0	0	0	0	0	0	0	0	0	0	0	0	0	0	0
AN0117_C	0	0	0	0	0	0	0	0	0	0	0	0	0	0	0	0
AN0118_C	0	0	0	0	0	0	0	0	0	0	0	0	0	0	0	0
AN0119_C	0	0	0	0	0	0	0	0	0	0	0	0	0	0	0	0
AN0120_C	0	0	0	0	0	0	0	0	0	0	0	0	0	0	0	0
AN0121_C	0	0	0	0	0	0	0	0	0	0	0	0	0	0	0	0
AN0122_C	0	0	0	0	0	0	0	0	0	0	0	0	0	0	0	0
AN0123_C	0	0	0	0	0	0	0	0	0	0	0	0	0	0	0	0
AN0124_C	0	0	0	0	0	0	0	0	0	0	0	0	0	0	0	0
AN0125_C	0	0	0	0	0	0	0	0	0	0	0	0	0	0	0	0
AN0126_C	0	0	0	0	0	0	0	0	0	0	0	0	0	0	0	0
AN0127_C	0	0	0	0	0	0	0	0	0	0	0	0	0	0	0	0
AN0128_C	0	0	0	0	0	0	0	0	0	0	0	0	0	0	0	0
AN0129_C	0	0	0	0	0	0	0	0	0	0	0	0	0	0	0	0
AN0130_C	0	0	0	0	0	0	0	0	0	0	0	0	0	0	0	0
AN0131_C	0	0	0	0	0	0	0	0	0	0	0	0	0	0	0	0
AN0132_C	0	0	0	1	0	0	0	0	0	0	0	0	0	0	0	0
AN0134_C	0	0	0	0	0	0	0	0	0	0	0	0	0	0	0	0
AN0135_C	0	0	0	0	0	0	0	0	0	0	0	0	0	0	0	0
AN0136_C	0	0	0	0	0	0	0	0	0	0	0	0	0	0	0	0
AN0137_C	0	0	0	0	0	0	0	0	0	0	0	0	0	0	0	0
AN0138_C	0	0	0	0	0	0	0	0	0	0	0	0	0	0	0	0
AN0139_C	0	0	0	0	0	0	0	0	0	0	0	0	0	0	0	0
AN0140_C	0	0	0	0	0	0	0	0	0	0	0	0	0	0	0	0

	Dup 0	Dup 1	Dup 2	Dup 3	Dup 4	Dup 5	Dup 6	Dup 7	Dup 8	Dup 9	Dup 10	Dup 11	Dup 12	Dup 13	Dup 14	Dup 15
AN0141_C	0	0	0	0	0	0	0	0	0	0	0	0	0	0	0	0
AN0142_C	0	0	0	0	0	0	0	0	0	0	0	0	0	0	0	0
AN0143_C	0	0	0	0	0	0	0	0	0	0	0	0	0	0	0	0
AN0144_C	0	0	0	0	0	0	0	0	0	0	0	0	0	0	0	0
AN0147_C	0	0	0	0	0	0	0	0	0	0	0	0	0	0	0	0
AN0149_C	0	0	0	0	0	0	0	0	0	0	0	0	0	0	0	0
AN0151_C	0	0	0	0	0	0	0	0	0	0	0	0	0	0	0	0
AN0152_C	0	0	0	0	0	0	0	0	0	0	0	0	0	0	0	0
AN0153_C	0	0	0	0	0	0	0	0	0	0	0	0	0	0	0	0
AN0154_C	0	0	0	0	0	0	0	0	0	0	0	0	0	0	0	0
AN0155_C	0	0	0	0	0	0	0	0	0	0	0	0	0	0	0	0
AN0156_C	0	0	0	0	0	0	0	0	0	0	0	0	0	0	0	0
AN0157_C	0	0	0	0	0	0	0	0	0	0	0	0	0	0	0	0
AN0158_C	0	0	0	0	0	0	0	0	0	0	0	0	0	0	0	0
AN0159_C	0	0	0	0	0	0	0	0	0	0	0	0	0	0	0	0
AN0160_C	0	0	0	0	0	0	0	0	0	0	0	0	0	0	0	0
AN0162_C	0	0	0	0	0	0	0	0	0	0	0	0	0	0	0	0
AN0163_C	0	0	0	0	0	0	0	0	0	0	0	0	0	0	0	0
AN0164_C	0	0	0	0	0	0	0	0	0	0	0	0	0	0	0	0
AN0165_C	0	0	0	0	0	0	0	0	0	0	0	0	0	0	0	0
AN0166_C	0	0	0	0	0	0	0	0	0	0	0	0	0	0	0	0
AN0167_C	0	0	0	0	0	0	0	0	0	0	0	0	0	0	0	0
AN0168_C	0	0	0	0	0	0	0	0	0	0	0	0	0	0	0	0
AN0169_C	0	0	0	0	0	0	0	0	0	0	0	0	0	0	0	0
AN0170_C	0	0	0	0	0	0	0	0	0	0	0	0	0	0	0	0
AN0171_C	0	0	0	0	0	0	0	0	0	0	0	0	0	0	0	0
AN0172_C	0	0	0	0	0	0	0	0	0	0	0	0	0	0	0	0
AN0173_C	0	0	0	0	0	0	0	0	0	0	0	0	0	0	0	0
AN0174_C	0	0	0	0	0	0	0	0	0	0	0	0	0	0	0	0
AN0175_C	0	0	0	0	0	0	0	0	0	0	0	0	0	0	0	0
AN0176_C	0	0	0	0	0	0	0	0	0	0	0	0	0	0	0	0
AN0177_C	0	0	0	0	0	0	0	0	0	0	0	0	0	0	0	0
AN0178_C	0	0	0	0	0	0	0	0	0	0	0	0	0	0	0	0
AN0179_C	0	0	0	0	0	0	0	0	0	0	0	0	0	0	0	0
AN0180_C	0	0	0	0	0	0	0	0	0	0	0	0	0	0	0	0
AN0181_C	0	0	0	0	0	0	0	0	0	0	0	0	0	0	0	0
AN0182_C	0	0	0	0	0	0	0	0	0	0	0	0	0	0	0	0
AN0183_C	0	0	0	0	0	0	0	0	0	0	0	0	0	0	0	0
AN0184_C	0	0	0	0	0	0	0	0	0	0	0	0	0	0	0	0
AN0185_C	0	0	0	0	0	0	0	0	0	0	0	0	0	0	0	0
AN0186_C	0	0	0	0	0	0	0	0	0	0	0	0	0	0	0	0
AN0187_C	0	0	0	0	0	0	0	0	0	0	0	0	0	0	0	0
AN0188_C	0	0	0	0	0	0	0	0	0	0	0	0	0	0	0	0
AN0189_C	0	0	0	0	0	0	0	0	0	0	0	0	0	0	0	0
AN0190_C	0	0	0	0	0	0	0	0	0	0	0	0	0	0	0	0
AN0191_C	0	0	0	0	0	0	0	0	0	0	0	0	0	0	0	0
AN0192_C	0	0	0	0	0	0	0	0	0	0	0	0	0	0	0	0
AN0193_C	0	0	0	0	0	0	0	0	0	0	0	0	0	0	0	0
AN0194_C	0	0	0	0	0	0	0	0	0	0	0	0	0	0	0	0
AN0196_C	0	0	0	0	0	0	0	0	0	0	0	0	0	0	0	0
AN0197_C	0	0	0	0	0	0	0	0	0	0	0	0	0	0	0	0
AN0198_C	0	0	0	0	0	0	0	0	0	0	0	0	0	0	0	0
AN0199_C	0	0	0	0	0	0	0	0	0	0	0	0	0	0	0	0
AN0200_C	0	0	0	0	0	0	0	0	0	0	0	0	0	0	0	0
AN0201_C	0	0	0	0	0	0	0	0	0	0	0	0	0	0	0	0
AN0202_C	0	0	0	0	0	0	0	0	0	0	0	0	0	0	0	0
AN0203_C	0	0	0	0	0	0	0	0	0	0	0	0	0	0	0	0
AN0204_C	0	0	0	0	0	0	0	0	0	0	0	0	0	0	0	0
AN0205_C	0	0	0	0	0	0	0	0	0	0	0	0	0	0	0	0
AN0206_C	0	0	0	0	0	0	0	0	0	0	0	0	0	0	0	0
AN0207_C	0	0	0	0	0	0	0	0	0	0	0	0	0	0	0	0
AN0208_C	0	0	0	0	0	0	0	0	0	0	0	0	0	0	0	0
AN0209_C	0	0	0	0	0	0	0	0	0	0	0	0	0	0	0	0
AN0210_C	0	0	0	0	0	0	0	0	0	0	0	0	0	0	0	0
AN0212_C	0	0	0	0	0	0	0	0	0	0	0	0	0	0	0	0
AN0213_C	0	0	0	0	0	0	0	0	0	0	0	0	0	0	0	0
AN0214_C	0	0	0	0	0	0	0	0	0	0	0	0	0	0	0	0
AN0215_C	0	0	0	0	0	0	0	0	0	0	0	0	0	0	0	0

	Dup 0	Dup 1	Dup 2	Dup 3	Dup 4	Dup 5	Dup 6	Dup 7	Dup 8	Dup 9	Dup 10	Dup 11	Dup 12	Dup 13	Dup 14	Dup 15
AN0217_C	0	0	0	0	0	0	0	0	0	0	0	0	0	0	0	0
AN0218_C	0	0	0	0	0	0	0	0	0	0	0	0	0	0	0	0
AN0219_C	0	0	0	0	0	0	0	0	0	0	0	0	0	0	0	0
AN0220_C	0	0	0	0	0	0	0	0	0	0	0	0	0	0	0	0
AN0221_C	0	0	0	0	0	0	0	0	0	0	0	0	0	0	0	0
AN0222_C	0	0	0	0	0	0	0	0	0	0	0	0	0	0	0	0
AN0223_C	0	0	0	0	0	0	0	0	0	0	0	0	0	0	0	0
AN0224_C	0	0	0	0	0	0	0	0	0	0	0	0	0	0	0	0
AN0225_C	0	0	0	0	0	0	0	0	0	0	0	0	0	0	0	0
AN0226_C	0	0	0	0	0	0	0	0	0	0	0	0	0	0	0	0
AN0227_C	0	0	0	0	0	0	0	0	0	0	0	0	0	0	0	0
AN0228_C	0	0	0	0	0	0	0	0	0	0	0	0	0	0	0	0
AN0229_C	0	0	0	0	0	0	0	0	0	0	0	0	0	0	0	0
AN0230_C	0	0	0	0	0	0	0	0	0	0	0	0	0	0	0	0
AN0231_C	0	0	0	0	0	0	0	0	0	0	0	0	0	0	0	0
AN0232_C	0	0	0	0	0	0	0	0	0	0	0	0	0	0	0	0
AN0233_C	0	0	0	0	0	0	0	0	0	0	0	0	0	0	0	0
AN0234_C	0	0	0	0	0	0	0	0	0	0	0	0	0	0	0	0
AN0235_C	0	0	0	0	0	0	0	0	0	0	0	0	0	0	0	0
AN0236_C	0	0	0	0	0	0	0	0	0	0	0	0	0	0	0	0
AN0237_C	0	0	0	0	0	0	0	0	0	0	0	0	0	0	0	0
AN0238_C	0	0	0	0	0	0	0	0	0	0	0	0	0	0	0	0
AN0239_C	0	0	0	0	0	0	0	0	0	0	0	0	0	0	0	0
AN0240_C	0	0	0	0	0	0	0	0	0	0	0	0	0	0	0	0
AN0241_C	0	0	0	0	0	0	0	0	0	0	0	0	0	0	0	0
AN0242_C	0	0	0	0	0	0	0	0	0	0	0	0	0	0	0	0
AN0243_C	0	0	0	0	0	0	0	0	0	0	0	0	0	0	0	0
AN0244_C	0	0	0	0	0	0	0	0	0	0	0	0	0	0	0	0
AN0245_C	0	0	0	0	0	0	0	0	0	0	0	0	0	0	0	0
AN0246_C	0	0	0	0	0	0	0	0	0	0	0	0	0	0	0	0
AN0247_C	0	0	0	0	0	0	0	0	0	0	0	0	0	0	0	0
AN0248_C	0	0	0	0	0	0	0	0	0	0	0	0	0	0	0	0
AN0250_C	0	0	0	0	0	0	0	0	0	0	0	0	0	0	0	0
AN0251_C	0	0	0	0	0	0	0	0	0	0	0	0	0	0	0	0
AN0252_C	0	0	0	0	0	0	0	0	0	0	0	0	0	0	0	0
AN0253_C	0	0	0	0	0	0	0	0	0	0	0	0	0	0	0	0
AN0254_C	0	0	0	0	0	0	0	0	0	0	0	0	0	0	0	0
AN0255_C	0	0	0	0	0	0	0	0	0	0	0	0	0	0	0	0
AN0256_C	0	0	0	0	0	0	0	0	0	0	0	0	0	0	0	0
AN0258_C	0	0	0	0	0	0	0	0	0	0	0	0	0	0	0	0
AN0259_C	0	0	0	0	0	0	0	0	0	0	0	0	0	0	0	0
AN0260_C	0	0	0	0	0	0	0	0	0	0	0	0	0	0	0	0
AN0261_C	0	0	0	0	0	0	0	0	0	0	0	0	0	0	0	0
AN0262_C	0	0	0	0	0	0	0	0	0	0	0	0	0	0	0	0
AN0263_C	0	0	0	0	0	0	0	0	0	0	0	0	0	0	0	0
AN0264_C	0	0	0	0	0	0	0	0	0	0	0	0	0	0	0	0
AN0265_C	0	0	0	0	0	0	0	0	0	0	0	0	0	0	0	0
AN0266_C	0	0	0	0	0	0	0	0	0	0	0	0	0	0	0	0
AN0267_C	0	0	0	0	0	0	0	0	0	0	0	0	0	0	0	0
AN0268_C	0	0	0	0	0	0	0	0	0	0	0	0	0	0	0	0
AN0269_C	0	0	0	0	0	0	0	0	0	0	0	0	0	0	0	0
AN0270_C	0	0	0	0	0	0	0	0	0	0	0	0	0	0	0	0
AN0271_C	0	0	0	0	0	0	0	0	0	0	0	0	0	0	0	0
AN0272_C	0	0	0	0	0	0	0	0	0	0	0	0	0	0	0	0
AN0275_C	0	0	0	0	0	0	0	0	0	0	0	0	0	0	0	0
AN0276_C	0	0	0	0	0	0	0	0	0	0	0	0	0	0	0	0
AN0277_C	0	0	0	0	0	0	0	0	0	0	0	0	0	0	0	0
AN0278_C	0	0	0	0	0	0	0	0	0	0	0	0	0	0	0	0
AN0279_C	0	0	0	0	0	0	0	0	0	0	0	0	0	0	0	0
AN0280_C	0	0	0	0	0	0	0	0	0	0	0	0	0	0	0	0
AN0281_C	0	0	0	0	0	0	0	0	0	0	0	0	0	0	0	0
AN0282_C	0	0	0	0	0	0	0	0	0	0	0	0	0	0	0	0
AN0283_C	0	0	0	0	0	0	0	0	0	0	0	0	0	0	0	0
AN0284_C	0	0	0	0	0	0	0	0	0	0	0	0	0	0	0	0
AN0285_C	0	0	0	0	0	0	0	0	0	0	0	0	0	0	0	0
AN0286_C	0	0	0	0	0	0	0	0	0	0	0	0	0	0	0	0
AN0287_C	0	0	0	0	0	0	0	0	0	0	0	0	0	0	0	0
AN0288_C	0	0	0	0	0	0	0	0	0	0	0	0	0	0	0	0

	Dup 0	Dup 1	Dup 2	Dup 3	Dup 4	Dup 5	Dup 6	Dup 7	Dup 8	Dup 9	Dup 10	Dup 11	Dup 12	Dup 13	Dup 14	Dup 15
AN0289_C	0	0	0	0	0	0	0	0	0	0	0	0	0	0	0	0
AN0290_C	0	0	0	0	0	0	0	0	0	0	0	0	0	0	0	0
AN0291_C	0	0	0	0	0	0	0	0	0	0	0	0	0	0	0	0
AN0292_C	0	0	0	0	0	0	0	0	0	0	0	0	0	0	0	0
AN0293_C	0	0	0	0	0	0	0	0	0	0	0	0	0	0	0	0
AN0294_C	0	0	0	0	0	0	0	0	0	0	0	0	0	0	0	0
AN0295_C	0	0	0	0	0	0	0	0	0	0	0	0	0	0	0	0
AN0296_C	0	0	0	0	0	0	0	0	0	0	0	0	0	0	0	0
AN0297_C	0	0	0	0	0	0	0	0	0	0	0	0	0	0	0	0
AN0298_C	0	0	0	0	0	0	0	0	0	0	0	0	0	0	0	0
AN0299_C	0	0	0	0	0	0	0	0	0	0	0	0	0	0	0	0
AN0300_C	0	0	0	0	0	0	0	0	0	0	0	0	0	0	0	0
AN0301_C	0	0	0	0	0	0	0	0	0	0	0	0	0	0	0	0
AN0302_C	0	0	0	0	0	0	0	0	0	0	0	0	0	0	0	0
AN0303_C	0	0	0	0	0	0	0	0	0	0	0	0	0	0	0	0
AN0304_C	0	0	0	0	0	0	0	0	0	0	0	0	0	0	0	0
AN0305_C	0	0	0	0	0	0	0	0	0	0	0	0	0	0	0	0
AN0306_C	0	0	0	0	0	0	0	0	0	0	0	0	0	0	0	0
AN0307_C	0	0	0	0	0	0	0	0	0	0	0	0	0	0	0	0
AN0308_C	0	0	0	0	0	0	0	0	0	0	0	0	0	0	0	0
AN0309_C	0	0	0	0	0	0	0	0	0	0	0	0	0	0	0	0
AN0310_C	0	0	0	0	0	0	0	0	0	0	0	0	0	0	0	0
AN0311_C	0	0	0	0	0	0	0	0	0	0	0	0	0	0	0	0
AN0312_C	0	0	0	0	0	0	0	0	0	0	0	0	0	0	0	0
AN0313_C	0	0	0	0	0	0	0	0	0	0	0	0	0	0	0	0
AN0314_C	0	0	0	0	0	0	0	0	0	0	0	0	0	0	0	0
AN0315_C	0	0	0	0	0	0	0	0	0	0	0	0	0	0	0	0
AN0316_C	0	0	0	0	0	0	0	0	0	0	0	0	0	0	0	0
AN0317_C	0	0	0	0	0	0	0	0	0	0	0	0	0	0	0	0
AN0318_C	0	0	0	0	0	0	0	0	0	0	0	0	0	0	0	0
AN0319_C	0	0	0	0	0	0	0	0	0	0	0	0	0	0	0	0
AN0320_C	0	0	0	0	0	0	0	0	0	0	0	0	0	0	0	0
AN0321_C	0	0	0	0	0	0	0	0	0	0	0	0	0	0	0	0
AP0002_C	0	0	0	0	0	0	0	0	0	0	0	0	0	0	0	0
AP0005_C	0	0	0	0	0	0	0	0	0	0	0	0	0	0	0	0
AP0006_C	0	0	0	0	0	0	0	0	0	0	0	0	0	0	0	0
AP0007_C	0	0	0	0	0	0	0	0	0	0	0	0	0	0	0	0
AP0008_C	0	0	0	0	0	0	0	0	0	0	0	0	0	0	0	0
AP0009_C	0	0	0	0	0	0	0	0	0	0	0	0	0	0	0	0
AP0010_C	0	0	0	0	0	0	0	0	0	0	0	0	0	0	0	0
AP0011_C	0	0	0	0	0	0	0	0	0	0	0	0	0	0	0	0
AP0014_C	0	0	0	0	0	0	0	0	0	0	0	0	0	0	0	0
AP0017_C	0	0	0	0	0	0	0	0	0	0	0	0	0	0	0	0
AP0018_C	0	0	0	0	0	0	0	0	0	0	0	0	0	0	0	0
AP0019_C	0	0	0	0	0	0	0	0	0	0	0	0	0	0	0	0
AP0020_C	0	0	0	0	0	0	0	0	0	0	0	0	0	0	0	0
AP0021_C	0	0	0	0	0	0	0	0	0	0	0	0	0	0	0	0
AP0022_C	0	0	0	0	0	0	0	0	0	0	0	0	0	0	0	0
AP0023_C	0	0	0	0	0	0	0	0	0	0	0	0	0	0	0	0
AP0024_C	0	0	0	0	0	0	0	0	0	0	0	0	0	0	0	0
AP0025_C	0	0	0	0	0	0	0	0	0	0	0	0	0	0	0	0
AP0030_C	0	0	0	0	0	0	0	0	0	0	0	0	0	0	0	0
AP0031_C	0	0	0	0	0	0	0	0	0	0	0	0	0	0	0	0
AP0032_C	0	0	0	0	0	0	0	0	0	0	0	0	0	0	0	0
AP0033_C	0	0	0	0	0	0	0	0	0	0	0	0	0	0	0	0
AP0034_C	0	0	0	0	0	0	0	0	0	0	0	0	0	0	0	0
AP0035_C	0	0	0	0	0	0	0	0	0	0	0	0	0	0	0	0
AQ0001_C	0	0	0	0	0	0	0	0	0	0	0	0	0	0	0	0
AQ0002_C	0	0	0	0	0	0	0	0	0	0	0	0	0	0	0	0
AQ0004_C	0	0	0	0	0	0	0	0	0	0	0	0	0	0	0	0
AQ0005_C	0	0	0	0	0	0	0	0	0	0	0	0	0	0	0	0
AQ0011_C	0	0	0	0	0	0	0	0	0	0	0	0	0	0	0	0
AQ0012_C	0	0	0	0	0	0	0	0	0	0	0	0	0	0	0	0
AQ0013_C	0	0	0	0	0	0	0	0	0	0	0	0	0	0	0	0
AQ0014_C	0	0	0	0	0	0	0	0	0	0	0	0	0	0	0	0
AQ0015_C	0	0	0	0	0	0	0	0	0	0	0	0	0	0	0	0
AR0001_C	0	0	0	0	0	0	0	0	0	0	0	0	0	0	0	0
AR0007_C	0	0	0	0	0	0	0	0	0	0	0	0	0	0	0	0

	Dup 0	Dup 1	Dup 2	Dup 3	Dup 4	Dup 5	Dup 6	Dup 7	Dup 8	Dup 9	Dup 10	Dup 11	Dup 12	Dup 13	Dup 14	Dup 15
AR0008_C	0	0	0	0	0	0	0	0	0	0	0	0	0	0	0	0
AR0009_C	0	0	0	0	0	0	0	0	0	0	0	0	0	0	0	0
AR0010_C	0	0	0	0	0	0	0	0	0	0	0	0	0	0	0	0
AR0011_C	0	0	0	0	0	0	0	0	0	0	0	0	0	0	0	0
AR0012_C	0	0	0	0	0	0	0	0	0	0	0	0	0	0	0	0
AR0013_C	0	0	0	0	0	0	0	0	0	0	0	0	0	0	0	0
AR0014_C	0	0	0	0	0	0	0	0	0	0	0	0	0	0	0	0
AR0015_C	0	0	0	0	0	0	0	0	0	0	0	0	0	0	0	0
AR0016_C	0	0	0	0	0	0	0	0	0	0	0	0	0	0	0	0
AR0017_C	0	0	0	0	0	0	0	0	0	0	0	0	0	0	0	0
AR0018_C	0	0	0	0	0	0	0	0	0	0	0	0	0	0	0	0
AR0019_C	0	0	0	0	0	0	0	0	0	0	0	0	0	0	0	0
AR0020_C	0	0	0	0	0	0	0	0	0	0	0	0	0	0	0	0
AR0021_C	0	0	0	0	0	0	0	0	0	0	0	0	0	0	0	0
AR0022_C	0	0	0	0	0	0	0	0	0	0	0	0	0	0	0	0
AR0023_C	0	0	0	0	0	0	0	0	0	0	0	0	0	0	0	0
AR0024_C	0	0	0	0	0	0	0	0	0	0	0	0	0	0	0	0
AR0026_C	0	0	0	0	0	0	0	0	0	0	0	0	0	0	0	0
AR0027_C	0	0	0	0	0	0	0	0	0	0	0	0	0	0	0	0
AR0034_C	0	0	0	0	0	0	0	0	0	0	0	0	0	0	0	0
AR0035_C	0	0	0	0	0	0	0	0	0	0	0	0	0	0	0	0
AR0036_C	0	0	0	0	0	0	0	0	0	0	0	0	0	0	0	0
AR0038_C	0	0	0	0	0	0	0	0	0	0	0	0	0	0	0	0
AR0040_C	0	0	0	0	0	0	0	0	0	0	0	0	0	0	0	0
AR0042_C	0	0	0	0	0	0	0	0	0	0	0	0	0	0	0	0
AR0043_C	0	0	0	0	0	0	0	0	0	0	0	0	0	0	0	0
AR0044_C	0	0	0	0	0	0	0	0	0	0	0	0	0	0	0	0
AR0045_C	0	0	0	0	0	0	0	0	0	0	0	0	0	0	0	0
AR0046_C	0	0	0	0	0	0	0	0	0	0	0	0	0	0	0	0
AR0047_C	0	0	0	0	0	0	0	0	0	0	0	0	0	0	0	0
AR0048_C	0	0	0	0	0	0	0	0	0	0	0	0	0	0	0	0
AR0049_C	0	0	0	0	0	0	0	0	0	0	0	0	0	0	0	0
AR0050_C	0	0	0	0	0	0	0	0	0	0	0	0	0	0	0	0
AR0051_C	0	0	0	0	0	0	0	0	0	0	0	0	0	0	0	0
AR0052_C	0	0	0	0	0	0	0	0	0	0	0	0	0	0	0	0
AR0053_C	0	0	0	0	0	0	0	0	0	0	0	0	0	0	0	0
AR0054_C	0	0	0	0	0	0	0	0	0	0	0	0	0	0	0	0
AR0057_C	0	0	0	0	0	0	0	0	0	0	0	0	0	0	0	0
AR0059_C	0	0	0	0	0	0	0	0	0	0	0	0	0	0	0	0
AR0060_C	0	0	0	0	0	0	0	0	0	0	0	0	0	0	0	0
AR0061_C	0	0	0	0	0	0	0	0	0	0	0	0	0	0	0	0
AR0062_C	0	0	0	0	0	0	0	0	0	0	0	0	0	0	0	0
AR0063_C	0	0	0	0	0	0	0	0	0	0	0	0	0	0	0	0
AR0064_C	0	0	0	0	0	0	0	0	0	0	0	0	0	0	0	0
AR0065_C	0	0	0	0	0	0	0	0	0	0	0	0	0	0	0	0
AR0066_C	0	0	0	0	0	0	0	0	0	0	0	0	0	0	0	0
AR0069_C	0	0	0	0	0	0	0	0	0	0	0	0	0	0	0	0
AR0070_C	0	0	0	0	0	0	0	0	0	0	0	0	0	0	0	0
AR0071_C	0	0	0	0	0	0	0	0	0	0	0	0	0	0	0	0
AR0072_C	0	0	0	0	0	0	0	0	0	0	0	0	0	0	0	0
AR0073_C	0	0	0	0	0	0	0	0	0	0	0	0	0	0	0	0
AR0074_C	0	0	0	0	0	0	0	0	0	0	0	0	0	0	0	0
AR0075_C	0	0	0	0	0	0	0	0	0	0	0	0	0	0	0	0
AR0076_C	0	0	0	0	0	0	0	0	0	0	0	0	0	0	0	0
AR0077_C	0	0	0	0	0	0	0	0	0	0	0	0	0	0	0	0
AR0078_C	0	0	0	0	0	0	0	0	0	0	0	0	0	0	0	0
AR0079_C	0	0	0	0	0	0	0	0	0	0	0	0	0	0	0	0
AR0080_C	0	0	0	0	0	0	0	0	0	0	0	0	0	0	0	0
AR0081_C	0	0	0	0	0	0	0	0	0	0	0	0	0	0	0	0
AR0082_C	0	0	0	0	0	0	0	0	0	0	0	0	0	0	0	0
AR0083_C	0	0	0	0	0	0	0	0	0	0	0	0	0	0	0	0
AR0084_C	0	0	0	0	0	0	0	0	0	0	0	0	0	0	0	0
AR0085_C	0	0	0	0	0	0	0	0	0	0	0	0	0	0	0	0
AR0086_C	0	0	0	0	0	0	0	0	0	0	0	0	0	0	0	0
AR0087_C	0	0	0	0	0	0	0	0	0	0	0	0	0	0	0	0
AR0088_C	0	0	0	0	0	0	0	0	0	0	0	0	0	0	0	0
AR0089_C	0	0	0	0	0	0	0	0	0	0	0	0	0	0	0	0
AR0090_C	0	0	0	0	0	0	0	0	0	0	0	0	0	0	0	0

	Dup 0	Dup 1	Dup 2	Dup 3	Dup 4	Dup 5	Dup 6	Dup 7	Dup 8	Dup 9	Dup 10	Dup 11	Dup 12	Dup 13	Dup 14	Dup 15
AR0092_C	0	0	0	0	0	0	0	0	0	0	0	0	0	0	0	0
AR0093_C	0	0	0	0	0	0	0	0	0	0	0	0	0	0	0	0
AR0095_C	0	0	0	0	0	0	0	0	0	0	0	0	0	0	0	0
AR0096_C	0	0	0	0	0	0	0	0	0	0	0	0	0	0	0	0
AR0097_C	0	0	0	0	0	0	0	0	0	0	0	0	0	0	0	0
AR0098_C	0	0	0	0	0	0	0	0	0	0	0	0	0	0	0	0
AR0099_C	0	0	0	0	0	0	0	0	0	0	0	0	0	0	0	0
AR0100_C	0	0	0	0	0	0	0	0	0	0	0	0	0	0	0	0
AS0001_C	0	0	0	0	0	0	0	0	0	0	0	0	0	0	0	0
AS0002_C	0	0	0	0	0	0	0	0	0	0	0	0	0	0	0	0
AS0003_C	0	0	0	0	0	0	0	0	0	0	0	0	0	0	0	0
AS0004_C	0	0	0	0	0	0	0	0	0	0	0	0	0	0	0	0
AS0005_C	0	0	0	0	0	0	0	0	0	0	0	0	0	0	0	0
AS0006_C	0	0	0	0	0	0	0	0	0	0	0	0	0	0	0	0
AS0007_C	0	0	0	0	0	0	0	0	0	0	0	0	0	0	0	0
AS0008_C	0	0	0	0	0	0	0	0	0	0	0	0	0	0	0	0
AS0009_C	0	0	0	0	0	0	0	0	0	0	0	0	0	0	0	0
AS0010_C	0	0	0	0	0	0	0	0	0	0	0	0	0	0	0	0
AS0011_C	0	0	0	0	0	0	0	0	0	0	0	0	0	0	0	0
AS0012_C	0	0	0	0	0	0	0	0	0	0	0	0	0	0	0	0
AS0013_C	0	0	0	0	0	0	0	0	0	0	0	0	0	0	0	0
AS0014_C	0	0	0	0	0	0	0	0	0	0	0	0	0	0	0	0
AS0015_C	0	0	0	0	0	0	0	0	0	0	0	0	0	0	0	0
AS0016_C	0	0	0	0	0	0	0	0	0	0	0	0	0	0	0	0
AS0017_C	0	0	0	0	0	0	0	0	0	0	0	0	0	0	0	0
AS0018_C	0	0	0	0	0	0	0	0	0	0	0	0	0	0	0	0
AS0019_C	0	0	0	0	0	0	0	0	0	0	0	0	0	0	0	0
AS0020_C	0	0	0	0	0	0	0	0	0	0	0	0	0	0	0	0
AS0021_C	0	0	0	0	0	0	0	0	0	0	0	0	0	0	0	0
AS0022_C	0	0	0	0	0	0	0	0	0	0	0	0	0	0	0	0
AS0024_C	0	0	0	0	0	0	0	0	0	0	0	0	0	0	0	0
AS0025_C	0	0	0	0	0	0	0	0	0	0	0	0	0	0	0	0
AS0026_C	0	0	0	0	0	0	0	0	0	0	0	0	0	0	0	0
AS0027_C	0	0	0	0	0	0	0	0	0	0	0	0	0	0	0	0
AS0028_C	0	0	0	0	0	0	0	0	0	0	0	0	0	0	0	0
AS0029_C	0	0	0	0	0	0	0	0	0	0	0	0	0	0	0	0
AS0030_C	0	0	0	0	0	0	0	0	0	0	0	0	0	0	0	0
AS0032_C	0	0	0	0	0	0	0	0	0	0	0	0	0	0	0	0
AS0033_C	0	0	0	0	0	0	0	0	0	0	0	0	0	0	0	0
AS0034_C	0	0	0	0	0	0	0	0	0	0	0	0	0	0	0	0
AS0035_C	0	0	0	0	0	0	0	0	0	0	0	0	0	0	0	0
AS0036_C	0	0	0	0	0	0	0	0	0	0	0	0	0	0	0	0
AS0037_C	0	0	0	0	0	0	0	0	0	0	0	0	0	0	0	0
AS0039_C	0	0	0	0	0	0	0	0	0	0	0	0	0	0	0	0
AS0040_C	0	0	0	0	0	0	0	0	0	0	0	0	0	0	0	0
AS0041_C	0	0	0	0	0	0	0	0	0	0	0	0	0	0	0	0
AS0042_C	0	0	0	0	0	0	0	0	0	0	0	0	0	0	0	0
AS0044_C	0	0	0	0	0	0	0	0	0	0	0	0	0	0	0	0
AS0045_C	0	0	0	0	0	0	0	0	0	0	0	0	0	0	0	0
AS0046_C	0	0	0	0	0	0	0	0	0	0	0	0	0	0	0	0
AS0047_C	0	0	0	0	0	0	0	0	0	0	0	0	0	0	0	0
AS0048_C	0	0	0	0	0	0	0	0	0	0	0	0	0	0	0	0
AS0049_C	0	0	0	0	0	0	0	0	0	0	0	0	0	0	0	0
AS0051_C	0	0	0	0	0	0	0	0	0	0	0	0	0	0	0	0
AS0052_C	0	0	0	0	0	0	0	0	0	0	0	0	0	0	0	0
AS0053_C	0	0	0	0	0	0	0	0	0	0	0	0	0	0	0	0
AS0054_C	0	0	0	0	0	0	0	0	0	0	0	0	0	0	0	0
AS0055_C	0	0	0	0	0	0	0	0	0	0	0	0	0	0	0	0
AS0056_C	0	0	0	0	0	0	0	0	0	0	0	0	0	0	0	0
AS0057_C	0	0	0	0	0	0	0	0	0	0	0	0	0	0	0	0
AS0058_C	0	0	0	0	0	0	0	0	0	0	0	0	0	0	0	0
AS0059_C	0	0	0	0	0	0	0	0	0	0	0	0	0	0	0	0
AS0060_C	0	0	0	0	0	0	0	0	0	0	0	0	0	0	0	0
AS0062_C	0	0	0	0	0	0	0	0	0	0	0	0	0	0	0	0
AS0064_C	0	0	0	0	0	0	0	0	0	0	0	0	0	0	0	0
AS0065_C	0	0	0	0	0	0	0	0	0	0	0	0	0	0	0	0
AS0066_C	0	0	0	0	0	0	0	0	0	0	0	0	0	0	0	0
AS0068_C	0	0	0	0	0	0	0	0	0	0	0	0	0	0	0	0

	Dup 0	Dup 1	Dup 2	Dup 3	Dup 4	Dup 5	Dup 6	Dup 7	Dup 8	Dup 9	Dup 10	Dup 11	Dup 12	Dup 13	Dup 14	Dup 15
AS0069_C	0	0	0	0	0	0	0	0	0	0	0	0	0	0	0	0
AS0070_C	0	0	0	0	0	0	0	0	0	0	0	0	0	0	0	0
AS0071_C	0	0	0	0	0	0	0	0	0	0	0	0	0	0	0	0
AS0072_C	0	0	0	0	0	0	0	0	0	0	0	0	0	0	0	0
AS0073_C	0	0	0	0	0	0	0	0	0	0	0	0	0	0	0	0
AS0074_C	0	0	0	0	0	0	0	0	0	0	0	0	0	0	0	0
AS0076_C	0	0	0	0	0	0	0	0	0	0	0	0	0	0	0	0
AS0077_C	0	0	0	0	0	0	0	0	0	0	0	0	0	0	0	0
AS0078_C	0	0	0	0	0	0	0	0	0	0	0	0	0	0	0	0
AV0001_C	0	0	0	0	0	0	0	0	0	0	0	0	0	0	0	0
AV0002_C	0	0	0	0	0	0	0	0	0	0	0	0	0	0	0	0
AV0003_C	0	0	0	0	0	0	0	0	0	0	0	0	0	0	0	0
AV0004_C	0	0	0	0	0	0	0	0	0	0	0	0	0	0	0	0
AV0005_C	0	0	0	0	0	0	0	0	0	0	0	0	0	0	0	0
AV0006_C	0	0	0	0	0	0	0	0	0	0	0	0	0	0	0	0
AV0007_C	0	0	0	0	0	0	0	0	0	0	0	0	0	0	0	0
AV0008_C	0	0	0	0	0	0	0	0	0	0	0	0	0	0	0	0
AV0009_C	0	0	0	0	0	0	0	0	0	0	0	0	0	0	0	0
AV0010_C	0	0	0	0	0	0	0	0	0	0	0	0	0	0	0	0
AV0011_C	0	0	0	0	0	0	0	0	0	0	0	0	0	0	0	0
AV0012_C	0	0	0	0	0	0	0	0	0	0	0	0	0	0	0	0
AV0013_C	0	0	0	0	0	0	0	0	0	0	0	0	0	0	0	0
AV0014_C	0	0	0	0	0	0	0	0	0	0	0	0	0	0	0	0
AV0015_C	0	0	0	0	0	0	0	0	0	0	0	0	0	0	0	0
AV0017_C	0	0	0	0	0	0	0	0	0	0	0	0	0	0	0	0
AV0018_C	0	0	0	0	0	0	0	0	0	0	0	0	0	0	0	0
AV0020_C	0	0	0	0	0	0	0	0	0	0	0	0	0	0	0	0
AV0021_C	0	0	0	0	0	0	0	0	0	0	0	0	0	0	0	0
AV0022_C	0	0	0	0	0	0	0	0	0	0	0	0	0	0	0	0
AV0024_C	0	0	0	0	0	0	0	0	0	0	0	0	0	0	0	0
AV0025_C	0	0	0	0	0	0	0	0	0	0	0	0	0	0	0	0
AV0026_C	0	0	0	0	0	0	0	0	0	0	0	0	0	0	0	0
AV0027_C	0	0	0	0	0	0	0	0	0	0	0	0	0	0	0	0
AV0028_C	0	0	0	0	0	0	0	0	0	0	0	0	0	0	0	0
AV0029_C	0	0	0	0	0	0	0	0	0	0	0	0	0	0	0	0
AV0030_C	0	0	0	0	0	0	0	0	0	0	0	0	0	0	0	0
AV0031_C	0	0	1	0	0	0	0	0	0	0	0	0	0	0	0	0
AV0032_C	0	0	0	0	0	0	0	0	0	0	0	0	0	0	0	0
AV0033_C	0	0	0	0	0	0	0	0	1	0	0	0	0	0	0	0
AV0034_C	0	0	0	0	0	0	0	0	0	0	0	0	0	0	0	0
AV0035_C	0	0	0	0	0	0	0	0	0	0	0	0	0	0	0	0
AV0036_C	0	0	0	0	0	0	0	0	0	0	0	0	0	0	0	0
AV0037_C	0	0	0	0	0	0	0	0	0	0	0	0	0	0	0	0
AV0038_C	0	0	0	0	0	0	0	1	0	0	0	0	0	0	0	0
AV0039_C	0	0	0	0	0	0	0	0	0	0	0	0	0	0	0	0
AV0040_C	0	0	0	0	0	0	0	1	0	0	0	0	0	0	0	0
AV0041_C	0	0	0	0	0	0	0	0	0	0	0	0	0	0	0	0
AV0042_C	0	0	0	0	0	0	0	0	0	0	0	0	0	0	0	0
AV0043_C	0	0	0	0	0	0	0	0	0	0	0	0	0	0	0	0
AV0044_C	0	0	0	0	0	0	0	0	0	0	0	0	0	0	0	0
AV0045_C	0	0	0	0	0	0	0	0	0	0	0	0	0	0	0	0
AV0046_C	0	0	0	0	0	0	0	0	0	0	0	0	0	0	0	0
AV0047_C	0	0	0	0	0	0	0	0	0	0	0	0	0	0	0	0
AY0006_C	0	0	0	0	0	0	0	1	0	0	0	2	0	0	0	0
AY0007_C	0	0	0	0	0	0	0	0	0	0	0	1	0	0	1	1
AY0010_C	0	0	0	0	0	0	0	0	0	0	0	0	0	0	3	0
AY0011_C	0	0	0	0	0	0	0	0	0	0	0	1	0	0	1	0
AY0012_C	0	0	0	0	0	0	0	0	0	0	0	1	0	0	1	0
AY0013_C	0	0	0	0	0	0	0	1	0	0	0	2	0	0	1	0
AY0015_C	0	0	0	0	0	0	0	0	0	0	0	4	0	0	0	0
AY0016_C	0	0	0	0	0	0	0	0	0	0	0	3	0	0	1	1
AY0017_C	0	0	0	0	0	0	0	0	0	0	0	0	0	0	1	0
AY0018_C	0	0	0	0	0	0	0	1	0	0	0	0	0	0	0	1
AY0019_C	0	0	0	0	0	0	0	0	0	0	0	0	0	0	1	0
AY0020_C	0	0	0	0	0	0	0	1	0	0	0	1	0	0	1	0
AY0021_C	0	0	0	0	0	0	0	0	0	0	0	0	0	0	0	0
AY0023_C	0	0	0	0	0	0	0	1	0	0	0	3	0	0	0	1
AY0024_C	0	0	0	0	0	0	0	0	0	0	0	0	0	0	0	2

	Dup 0	Dup 1	Dup 2	Dup 3	Dup 4	Dup 5	Dup 6	Dup 7	Dup 8	Dup 9	Dup 10	Dup 11	Dup 12	Dup 13	Dup 14	Dup 15
AY0025_C	0	0	0	0	0	0	0	0	0	0	0	0	0	0	1	0
AY0026_C	0	0	0	0	0	0	0	0	0	0	0	3	0	0	0	0
AY0027_C	0	0	0	0	0	0	0	0	0	0	0	0	0	0	0	1
AY0029_C	0	0	0	0	0	0	0	1	0	0	0	0	0	0	1	1
AY0031_C	0	0	0	0	0	0	0	1	0	0	0	0	0	0	0	0
AY0032_C	0	0	0	0	0	0	0	0	0	0	0	0	0	0	1	1
AY0033_C	0	0	0	0	0	0	0	0	0	0	0	2	0	0	1	0
AY0034_C	0	0	0	0	0	0	0	0	0	0	0	0	0	0	0	1
AY0035_C	0	0	0	0	0	0	0	0	0	0	0	0	0	0	0	1
AY0036_C	0	0	0	0	0	0	0	0	0	0	0	0	0	0	1	0
AY0038_C	0	0	0	0	0	0	0	1	0	0	0	0	0	0	1	0
AY0039_C	0	0	0	0	0	0	0	1	0	0	0	2	0	1	0	1
AY0040_C	0	0	0	0	0	0	0	0	0	0	0	0	0	0	2	0
AY0041_C	0	0	0	0	0	0	0	1	0	0	0	0	0	0	0	0
AY0042_C	0	0	0	0	0	0	0	1	0	0	0	0	0	0	0	0
AY0043_C	0	0	0	0	0	0	0	0	0	0	0	1	0	0	1	1
AY0045_C	0	0	0	0	0	0	0	1	0	0	0	1	0	0	1	0
AY0046_C	0	0	0	0	0	0	0	0	0	0	0	0	0	0	0	0
AY0047_C	0	0	0	0	0	0	0	1	0	0	0	0	0	0	0	0
AY0048_C	0	0	0	0	0	0	0	1	0	0	0	0	0	0	0	1
AY0049_C	0	0	0	0	0	0	0	0	0	0	0	0	0	0	1	0
AY0050_C	0	0	0	0	0	0	0	0	0	0	0	0	0	0	0	1
AY0052_C	0	0	0	0	0	0	0	0	0	0	0	2	0	0	1	0
AY0053_C	0	0	0	0	0	0	0	0	0	0	0	0	0	0	0	1
AY0054_C	0	0	0	0	0	0	0	0	0	0	0	0	0	0	1	0
AY0055_C	0	0	0	0	0	0	0	1	0	0	0	2	0	0	0	0
AY0056_C	0	0	0	0	0	0	0	0	0	0	0	0	0	0	1	0
AY0057_C	0	0	0	0	0	0	0	0	0	0	0	2	0	0	0	1
AY0058_C	0	0	0	0	0	0	0	0	0	0	0	2	0	0	0	0
AY0059_C	0	0	0	0	0	0	0	0	0	0	0	0	0	0	0	1
AY0060_C	0	0	0	0	0	0	0	1	0	0	0	2	0	0	0	0
AY0061_C	0	0	0	0	0	0	0	0	0	0	0	0	0	0	1	0
AY0062_C	0	0	0	0	0	0	0	1	0	0	0	0	0	0	0	1
AY0063_C	0	0	0	0	0	0	0	0	0	0	0	0	0	0	1	0
AY0064_C	0	0	0	0	0	0	0	0	0	0	0	2	0	0	0	0
AY0065_C	0	0	0	0	0	0	0	0	0	0	0	0	0	0	1	0
AY0066_C	0	0	0	0	0	0	0	0	0	0	0	0	0	0	0	1
AY0067_C	0	0	0	0	0	0	0	0	0	0	0	0	0	0	1	0
AY0068_C	0	0	0	0	0	0	0	0	0	0	0	2	0	0	1	0
AY0069_C	0	0	0	0	0	0	0	0	0	0	0	0	0	0	NA	2
AY0070_C	0	0	0	0	0	0	0	0	0	0	0	0	0	0	1	1
AY0072_C	0	0	0	0	0	0	0	0	0	0	0	0	0	0	1	0
AY0074_C	0	0	0	0	0	0	0	1	0	0	0	0	0	0	0	0
AY0076_C	0	0	0	0	0	0	0	1	0	0	0	2	0	0	2	0
AY0077_C	0	0	0	0	0	0	0	1	0	0	0	2	0	0	0	0
AY0078_C	0	0	0	0	0	0	0	0	0	0	0	2	0	0	0	1
AY0079_C	0	0	0	0	0	0	0	1	0	0	0	0	0	0	0	1
AY0080_C	0	0	0	0	0	0	0	0	0	0	0	2	0	0	0	0
AY0082_C	0	0	0	0	0	0	0	0	0	0	0	0	0	0	0	2
AY0083_C	0	0	0	0	0	0	0	0	0	0	0	0	0	0	1	1
AY0085_C	0	0	0	0	0	0	0	1	0	0	0	2	0	0	0	0
AY0087_C	0	0	0	0	0	0	0	0	0	0	0	0	0	0	0	1
AY0088_C	0	0	0	0	0	0	0	1	0	0	0	2	0	0	0	0
AY0089_C	0	0	0	0	0	0	0	0	0	0	0	1	0	0	1	1
AY0090_C	0	0	0	0	0	0	0	0	0	0	0	3	0	0	0	0
AY0091_C	0	0	0	0	0	0	0	0	0	0	0	0	0	0	0	1
