# Supplementary material for: Whole-genome sequencing reveals high complexity of copy number variation at insecticide resistance loci in malaria mosquitoes
Source: Genome Res. 2019 Aug;29(8):1250–61. doi: 10.1101/gr.245795.118 (PMC6673711; doi:10.1101/gr.245795.118)
Supplement: Supplemental Material [file supp_gr.245795.118_Supplementary_Data_S5.pdf]

Electronic Supplementary Material S5

Description of CNV alleles detected in the GSTU - GSTE cluster region in Ag1000G phase 2.

Overview of all duplications in the GSTU - GSTE region.

Eleven CNV alleles were found that could be categorised according to their footprint of discordant reads or reads mapping to the CNV breakpoints. The regions covered by these CNVs are shown in Fig. **GST_S1**.

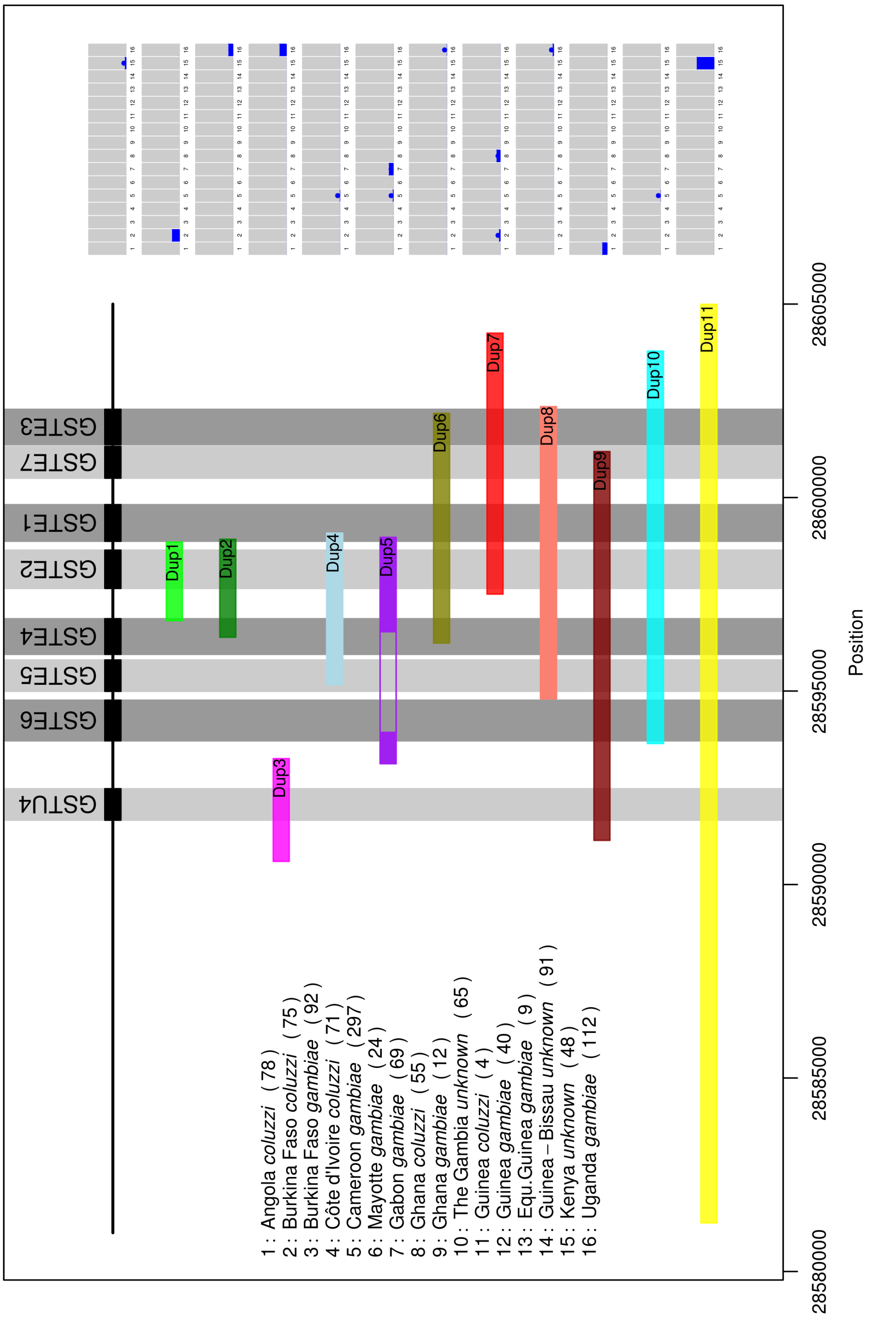

Fig. GST_S1: Overview of the CNVs in GSTE cluster genes present in Phase 2 of the Ag1000G dataset (alleles are named Gstue_Dup*, abbreviated to Dup* in the plot). Position on chromosome 3R is shown on the X axis. Barplots on the right show the proportion of samples that carry a given CNV in each of the Phase 2 populations. Numbers below the barplots are numeric population IDs detailed on the far left (numbers in brackets indicate the total number of samples from that population). Blue points on the barplots indicate that at least one sample in this population carried the CNV. The top barplot (Gstue_Dup0) shows CNVs that could not be categorised, each subsequent barplot represents the CNV shown to its left (Gstue_Dup1 - Dup11). The white box inside Gstue_Dup5 indicates the region of that duplication that has undergone subsequent deletion.

Duplication type 1

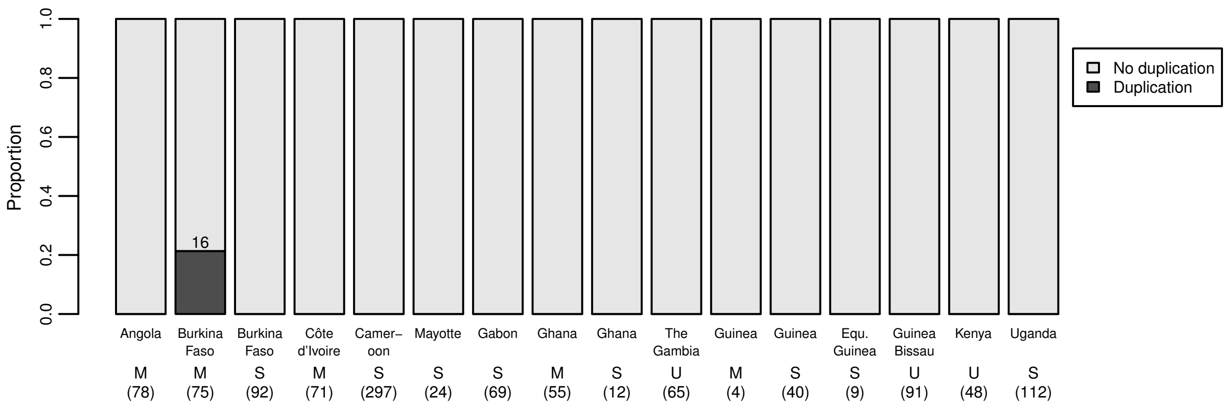

Fig. GST_S2: Barplot showing the proportion of samples that carry the Gstue_Dup1 duplication in each of the Phase 2 populations. Numbers above the dark grey bars indicate the absolute number of samples carrying the duplication. S = *Anopheles gambiae*, M = *Anopheles coluzzi*, U = species undetermined. Numbers in brackets indicate the total number of samples from that population.

Gstue_Dup1 was supported by face-away read pairs whose forward-facing read mapped in the interval 28596750 - 28597050 and whose reverse-facing read mapped in the interval 28598550 - 28598850 (Fig. GST_S3). Gstue_Dup1 was also supported by reads soft-clipped at the breakpoints (positions 28596818 and 28598850), with the clipped bases at each breakpoint aligning at the other breakpoint.

Gstue_Dup1 breakpoint:

```
AAGCGAATTCCTGTTTT T CGTTTGAATGGCGTTTCGGGCT
 end of the dup ^   ^ start of the dup
position 28598848   position 28596820
```

The T could sit on either side of the breakpoint.

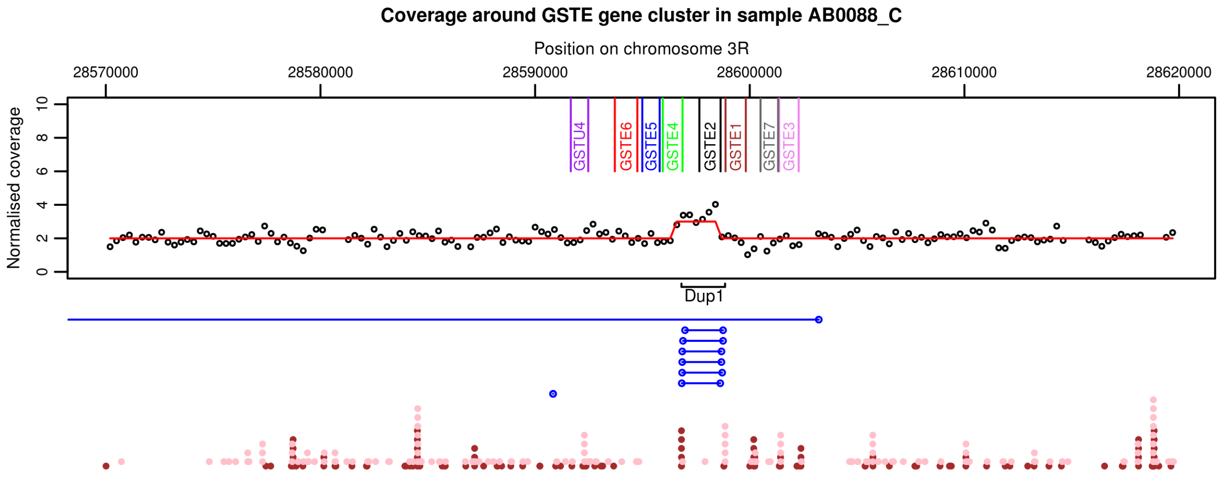

Fig. GST_S3: Example of coverage in an individual carrying the Gstue_Dup1 duplication. Open black circles indicate coverage at each position. The red line shows the HMM estimation of the coverage state at each position. Coloured vertical lines represent the positions of the GST genes. Pairs of blue points connected by lines indicate pairs of face-away reads. Reads soft-clipped before the alignment start point (dark brown points) and after the alignment end point (light brown points) are present at the start and end points of the duplication (clipped at positions 28596818 and 28598850 respectively). In each case, the clipped bases align to the other end of the duplication, as expected.

Gstue_Dup1 was only present in *An. coluzzii* from Burkina Faso (Table GST_S1.1). Estimates of copy number for Gstue_Dup1 in these populations indicated that all but one sample had a copy number of 1, with the remaining sample having a copy number of 3. Assuming that samples with a copy number of 1 are heterozygotes and that the sample with a copy number of 3 is a homozygote with incorrectly-called coverage, the allele distribution is consistent with Hardy-Weinberg (HW) expectations ($P = 1$).

Table GST_S1.1: Coverage calls for Gstue_Dup1. NAs were produced if coverage was too variable or if the duplication completely overlapped with another duplication whose coverage could also not be called.

copy number	AO col	BF col	BF gam	CI col	CM gam	FR gam	GA gam	GH col	GH gam	GM	GN col	GN gam	GQ gam	GW	KE	UG gam
NA	0	1	0	0	0	0	0	0	0	0	0	0	0	0	0	0
0	78	59	92	71	297	24	69	55	12	65	4	40	9	91	48	112
1	0	14	0	0	0	0	0	0	0	0	0	0	0	0	0	0
3	0	1	0	0	0	0	0	0	0	0	0	0	0	0	0	0

Table GST_S1.2: Coverage calls for all duplications in individuals that carry Gstue_Dup1.

	Dup 0	Dup 1	Dup 2	Dup 3	Dup 4	Dup 5	Dup 6	Dup 7	Dup 8	Dup 9	Dup 10	Dup 11
AB0088_C	0	1	0	0	0	0	0	0	0	0	0	0
AB0095_C	0	1	0	0	0	0	0	0	0	0	0	0
AB0097_C	0	1	0	0	0	0	0	0	0	0	0	0
AB0101_C	0	1	0	0	0	0	0	0	0	0	0	0
AB0123_C	0	3	0	0	0	0	0	0	0	0	0	0
AB0138_C	0	1	0	0	0	0	0	0	0	0	0	0
AB0139_C	0	NA	0	0	0	0	0	1	0	0	0	0
AB0182_C	0	1	0	0	0	0	0	0	0	0	0	0
AB0188_C	0	1	0	0	0	0	0	0	0	0	0	0
AB0204_C	0	1	0	0	0	0	0	0	0	0	0	0
AB0215_C	0	1	0	0	0	0	0	0	0	0	0	0
AB0246_C	0	1	0	0	0	0	0	0	0	0	0	0
AB0248_C	0	1	0	0	0	0	0	0	0	0	0	0
AB0250_C	0	1	0	0	0	0	0	0	0	0	0	0
AB0263_C	0	1	0	0	0	0	0	0	0	0	0	0
AB0279_C	0	1	0	0	0	0	0	0	0	0	0	0

Duplication type 2

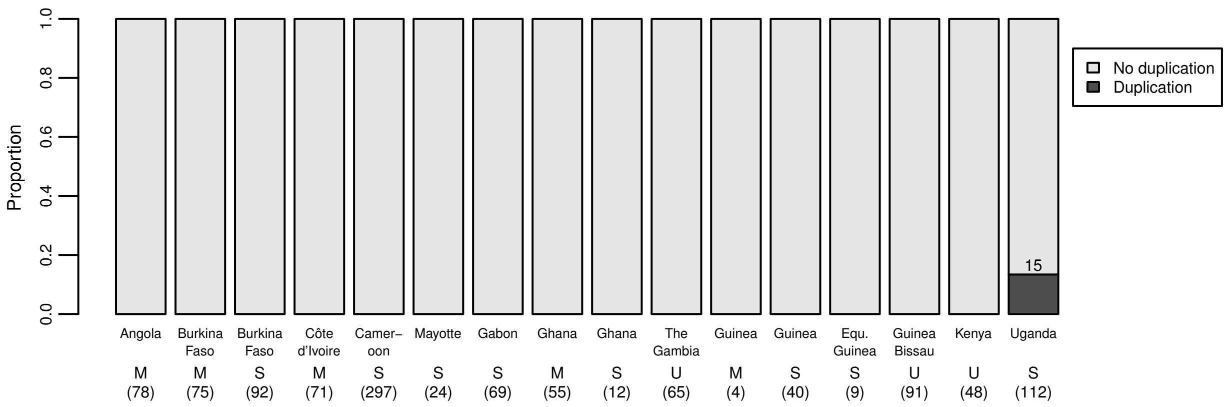

Fig. GST_S4: Barplot showing the proportion of samples that carry the Gstue_Dup2 duplication in each of the Phase 2 populations. Numbers above the dark grey bars indicate the absolute number of samples carrying the duplication. S = *Anopheles gambiae*, M = *Anopheles coluzzi*, U = species undetermined. Numbers in brackets indicate the total number of samples from that population.

We were unable to identify discordant read pairs or soft-clipped reads that consistently identified Gstue_Dup2 without false positives. Gstue_Dup2 was associated with reads that were soft-clipped at positions 28596390 and 28598923, the start and end of the duplication respectively, and whose clipped sequence was consistently the same, although the clipped sequence aligned to multiple places in the genome. We therefore called Gstue_Dup2 on the basis of the presence of soft-clipped reads at these two positions, whose clipped sequence ended with GGGGG and started with TTCCC respectively. This method produced two apparent false positives, that carry these soft-clipped reads but have no evidence of increased coverage. All samples that carried Gstue_Dup2 also carried Gstue_Dup3.

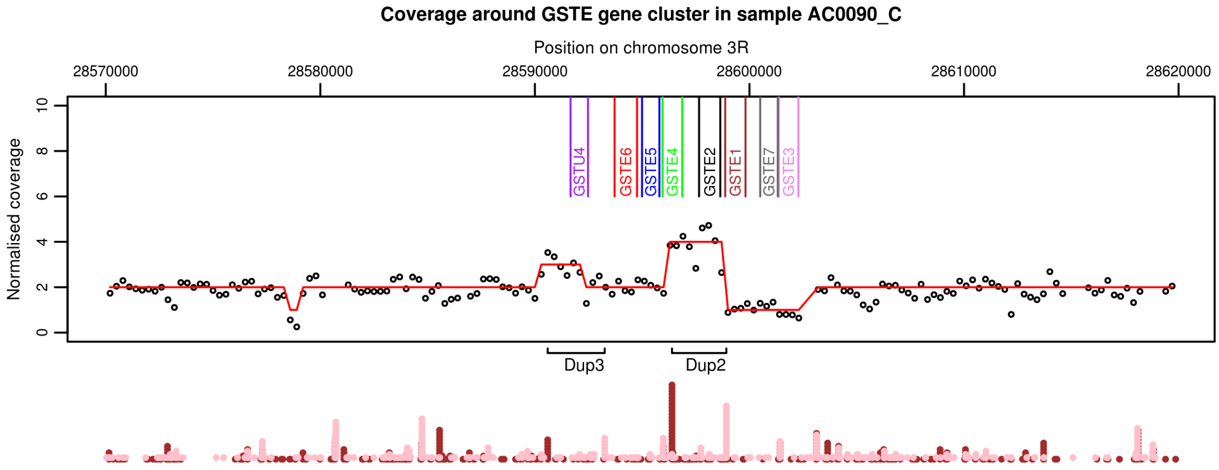

Fig. GST_S5: Example of coverage in an individual carrying the Gstue_Dup2 duplication. Open black circles indicate coverage at each position. The red line shows the HMM estimation of the coverage state at each position. Coloured vertical lines represent the positions of the GST genes. Reads soft-clipped before the alignment start point (dark brown points) and after the alignment end point (light brown points) are present at the start and end points of the duplication (clipped at positions 28596390 and 28598923 respectively).

Gstue_Dup2 was only found in *An. gambiae* from Uganda. Apart from the two samples that are probable false-positives described above, estimations of copy number ranged from 1 - 5 (Table GST_S2.1 & Table GST_S2.2), suggesting that both duplications and higher-order amplifications are present for Gstue_Dup2. This is further supported by the fact that the sample with a copy number of 5 only had a Gstue_Dup3 copy number of 1, suggesting that this sample was heterozygote for the duplicated haplotype (since Gstue_Dup2 is always found on a Gstue_Dup3 background). It is therefore not possible to make allele calls based on coverage for Gstue_Dup2.

Table GST_S2.1: Coverage calls for Gstue_Dup2. NAs were produced if coverage was too variable or if the duplication completely overlapped with another duplication whose coverage could also not be called.

copy number	AO col	BF col	BF gam	CI col	CM gam	FR gam	GA gam	GH col	GH gam	GM	GN col	GN gam	GQ gam	GW	KE	UG gam
NA	0	0	0	0	0	0	0	0	0	0	0	0	0	0	0	1
0	78	75	92	71	297	24	69	55	12	65	4	40	9	91	48	99
1	0	0	0	0	0	0	0	0	0	0	0	0	0	0	0	4
2	0	0	0	0	0	0	0	0	0	0	0	0	0	0	0	4
3	0	0	0	0	0	0	0	0	0	0	0	0	0	0	0	3
5	0	0	0	0	0	0	0	0	0	0	0	0	0	0	0	1

Table GST_S2.2: Coverage calls for all duplications in individuals that carry Gstue_Dup2.

	Dup 0	Dup 1	Dup 2	Dup 3	Dup 4	Dup 5	Dup 6	Dup 7	Dup 8	Dup 9	Dup 10	Dup 11
AC0089_C	0	0	1	1	0	0	0	0	0	0	0	0
AC0090_C	0	0	2	1	0	0	0	0	0	0	0	0
AC0102_C	0	0	0	1	0	0	0	0	0	0	0	0
AC0108_C	0	0	3	2	0	0	0	0	0	0	0	0
AC0124_C	0	0	2	1	0	0	0	0	0	0	0	0
AC0138_C	0	0	0	1	0	0	0	0	0	0	0	0
AC0148_C	0	0	2	1	0	0	0	0	0	0	0	0
AC0156_C	0	0	1	1	0	0	0	0	0	0	0	0
AC0157_C	0	0	2	1	0	0	0	0	0	0	0	0
AC0160_C	0	0	1	1	0	0	0	0	0	0	0	0
AC0167_C	0	0	3	1	0	0	0	0	0	0	0	0
AC0183_C	0	0	NA	1	0	0	0	0	NA	0	0	0
AC0188_C	0	0	3	0	0	0	0	0	0	0	0	0
AC0200_C	0	0	1	1	0	0	0	0	0	0	0	0
AC0203_C	0	0	5	1	0	0	0	0	0	0	0	0

Duplication type 3

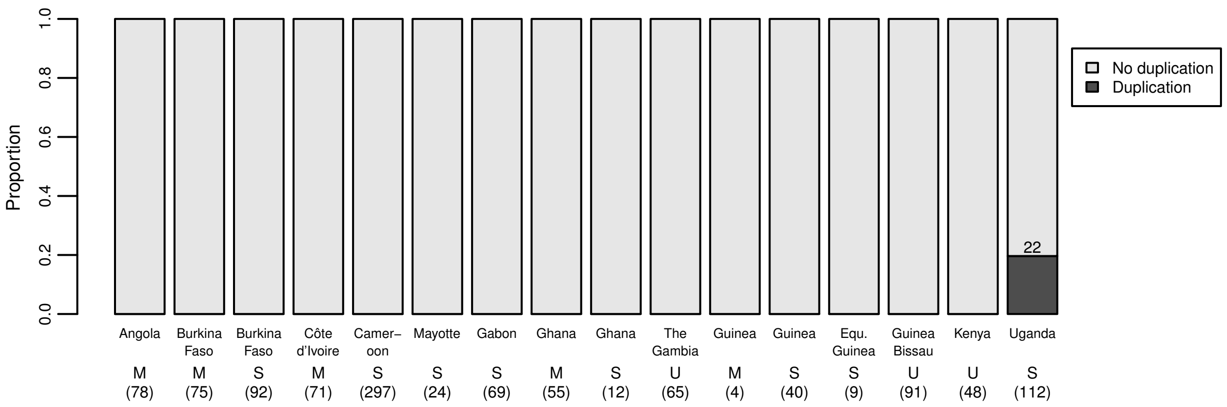

Fig. GST_S6: Barplot showing the proportion of samples that carry the Gstue_Dup3 duplication in each of the Phase 2 populations. Numbers above the dark grey bars indicate the absolute number of samples carrying the duplication. S = *Anopheles gambiae*, M = *Anopheles coluzzi*, U = species undetermined. Numbers in brackets indicate the total number of samples from that population.

Gstue_Dup3 was supported by face-away read pairs whose forward-facing read mapped in the interval 28590500 - 28590800 and whose reverse-facing read mapped in the interval 28592950 - 28593250 (Fig. Fig. **GST_S7**). Gstue_Dup3 was also supported by reads soft-clipped at the breakpoints (positions 28590597 and 28593254), with the clipped bases at each breakpoint aligning at the other breakpoint.

Gstue_Dup3 breakpoint:

```
GAAAAATCTAGTTGAAACT T AGGGCTATAGTATATATC
   end of the dup ^   ^ start of the dup
  position 28593252   position 28590599
```

The T could sit on either side of the breakpoint.

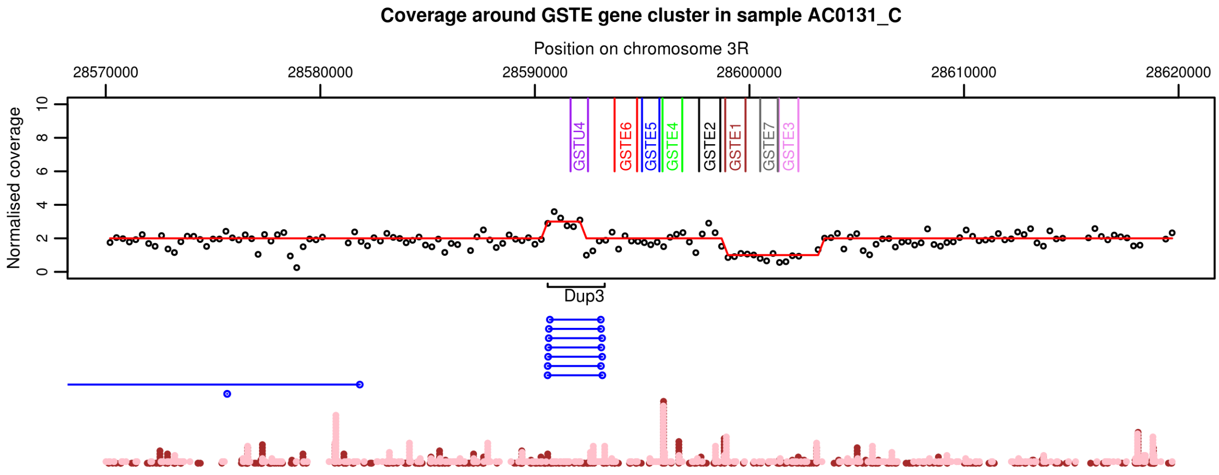

Fig. GST_S7: Example of coverage in an individual carrying the Gstue_Dup3 duplication. Open black circles indicate coverage at each position. The red line shows the HMM estimation of the coverage state at each position. Coloured vertical lines represent the positions of the GST genes. Pairs of blue points connected by lines indicate pairs of face-away reads. Reads soft-clipped before the alignment start point (dark brown points) and after the alignment end point (light brown points) are present at the start and end points of the duplication (clipped at positions 28590597 and 28593254 respectively). In each case, the clipped bases align to the other end of the duplication, as expected.

Gstue_Dup3 was only found in *An. gambiae* from Uganda. Estimates of copy number ranged from 3 to 4, with two samples having an apparent copy number of 0 since the HMM did not detect the duplication in these cases (Table GST_S3.1). Assuming that samples a copy number of 1 are heterozygotes, and that samples with a copy number of 2 are homozygotes for Gstue_Dup3 (Table GST_S3.2), the allele distribution is consistent with Hardy-Weinberg (HW) expectations ($P = 1$).

Table GST_S3.1: Coverage calls for Gstue_Dup3. NAs were produced if coverage was too variable or if the duplication completely overlapped with another duplication whose coverage could also not be called.

copy number	AO col	BF col	BF gam	CI col	CM gam	FR gam	GA gam	GH col	GH gam	GM	GN col	GN gam	GQ gam	GW	KE	UG gam
0	78	75	92	71	297	24	69	55	12	65	4	40	9	91	48	92
1	0	0	0	0	0	0	0	0	0	0	0	0	0	0	0	19
2	0	0	0	0	0	0	0	0	0	0	0	0	0	0	0	1

Table GST_S3.2: Coverage calls for all duplications in individuals that carry Gstue_Dup3.

	Dup 0	Dup 1	Dup 2	Dup 3	Dup 4	Dup 5	Dup 6	Dup 7	Dup 8	Dup 9	Dup 10	Dup 11
AC0089_C	0	0	1	1	0	0	0	0	0	0	0	0
AC0090_C	0	0	2	1	0	0	0	0	0	0	0	0
AC0096_C	0	0	0	1	0	0	0	0	0	0	0	0
AC0102_C	0	0	0	1	0	0	0	0	0	0	0	0
AC0108_C	0	0	3	2	0	0	0	0	0	0	0	0
AC0124_C	0	0	2	1	0	0	0	0	0	0	0	0
AC0131_C	0	0	0	1	0	0	0	0	0	0	0	0
AC0138_C	0	0	0	1	0	0	0	0	0	0	0	0
AC0139_C	0	0	0	1	0	0	0	0	0	0	0	0
AC0145_C	0	0	0	1	0	0	0	0	0	0	0	0
AC0148_C	0	0	2	1	0	0	0	0	0	0	0	0
AC0155_C	0	0	0	1	0	0	0	0	0	0	0	0
AC0156_C	0	0	1	1	0	0	0	0	0	0	0	0
AC0157_C	0	0	2	1	0	0	0	0	0	0	0	0
AC0160_C	0	0	1	1	0	0	0	0	0	0	0	0
AC0162_C	0	0	0	1	0	0	0	0	0	0	0	0
AC0167_C	0	0	3	1	0	0	0	0	0	0	0	0
AC0176_C	0	0	0	0	0	0	0	0	0	0	0	0
AC0183_C	0	0	NA	1	0	0	0	0	NA	0	0	0
AC0188_C	0	0	3	0	0	0	0	0	0	0	0	0
AC0200_C	0	0	1	1	0	0	0	0	0	0	0	0
AC0203_C	0	0	5	1	0	0	0	0	0	0	0	0

Duplication type 4

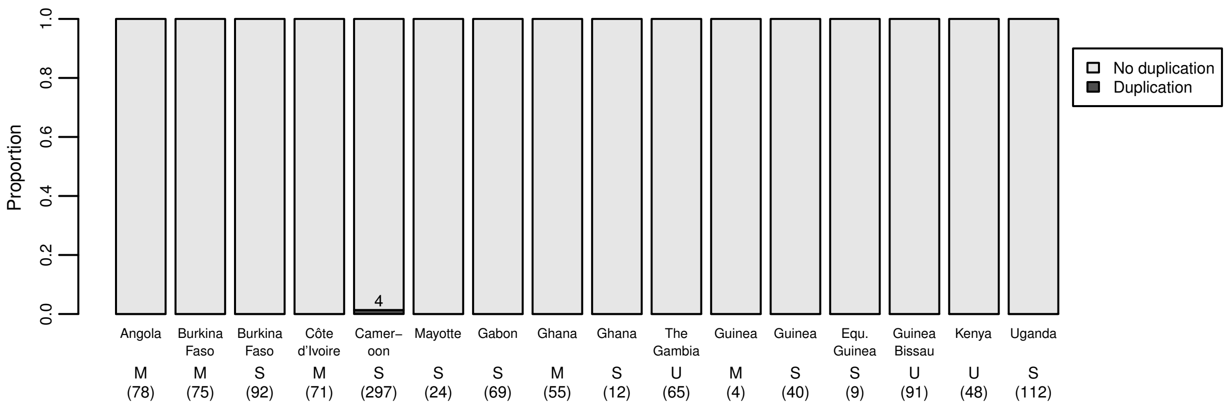

Fig. GST_S8: Barplot showing the proportion of samples that carry the Gstue_Dup4 duplication in each of the Phase 2 populations. Numbers above the dark grey bars indicate the absolute number of samples carrying the duplication. S = *Anopheles gambiae*, M = *Anopheles coluzzi*, U = species undetermined. Numbers in brackets indicate the total number of samples from that population.

Gstue_Dup4 was supported by face-away read pairs whose forward-facing read mapped in the interval 28595050 - 28595350 and whose reverse-facing read mapped in the interval 28598750 - 28599050 (Fig. GST_S9). Gstue_Dup4 was also supported by reads soft-clipped at the breakpoints (positions 28595162 and 28599081), with the clipped bases at each breakpoint aligning at the other breakpoint.

Gstue_Dup4 breakpoint:

```
GTGCACTCGCGGGAACTCGGACCTT TCCATCGGGA AGAACTCCTCCATCGTCGCAATCGTTG
             end of the dup ^               ^ start of the dup
            Position 28599070               Position 28595173
```

The sequence TCCATCGGGA could sit on either side of the breakpoint as it is present at both ends in the reference genome.

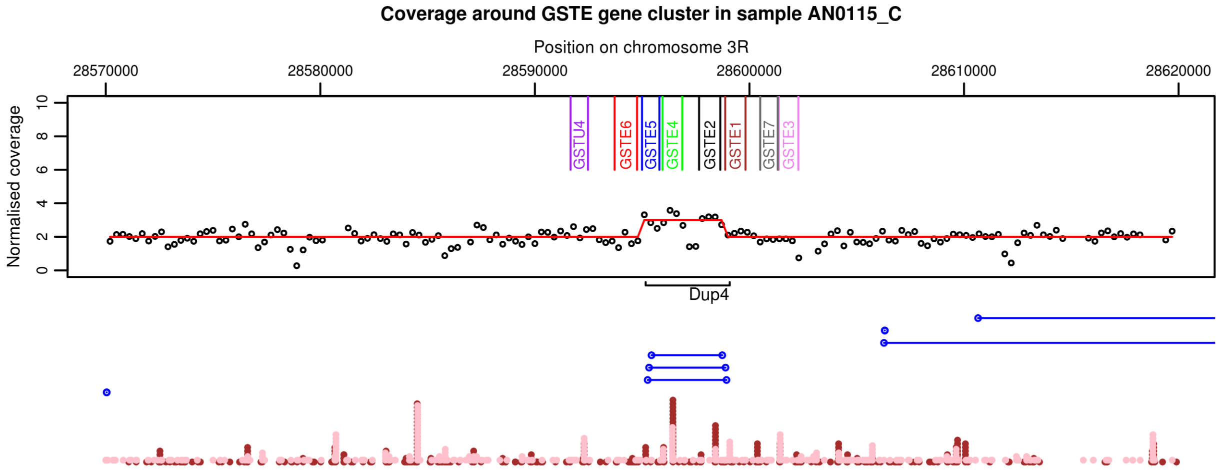

Fig. GST_S9: Example of coverage in an individual carrying the Gstue_Dup4 duplication. Open black circles indicate coverage at each position. The red line shows the HMM estimation of the coverage state at each position. Coloured vertical lines represent the positions of the GST genes. Pairs of blue points connected by lines indicate pairs of face-away reads. Reads soft-clipped before the alignment start point (dark brown points) and after the alignment end point (light brown points) are present at the start and end points of the duplication (clipped at positions 28595162 and 28599081 respectively). In each case, the clipped bases align to the other end of the duplication, as expected.

The four samples that carry Gstue_Dup4 all have a copy number of 1 (Tables GST_S4.1 & 4.2). Gstue_Dup4 is therefore a single copy duplication and all samples are heterozygous.

Table GST_S4.1: Coverage calls for Gstue_Dup4. NAs were produced if coverage was too variable or if the duplication completely overlapped with another duplication whose coverage could also not be called.

copy number	AO col	BF col	BF gam	CI col	CM gam	FR gam	GA gam	GH col	GH gam	GM	GN col	GN gam	GQ gam	GW	KE	UG gam
0	78	75	92	71	293	24	69	55	12	65	4	40	9	91	48	112
1	0	0	0	0	4	0	0	0	0	0	0	0	0	0	0	0

Table GST_S4.2: Coverage calls for all duplications in individuals that carry Gstue_Dup4.

	Dup 0	Dup 1	Dup 2	Dup 3	Dup 4	Dup 5	Dup 6	Dup 7	Dup 8	Dup 9	Dup 10	Dup 11
AN0014_C	0	0	0	0	1	0	0	0	0	0	0	0
AN0064_C	0	0	0	0	1	0	0	0	0	0	0	0
AN0073_C	0	0	0	0	1	0	0	0	0	0	0	0
AN0115_C	0	0	0	0	1	0	0	0	0	0	0	0

Duplication type 5

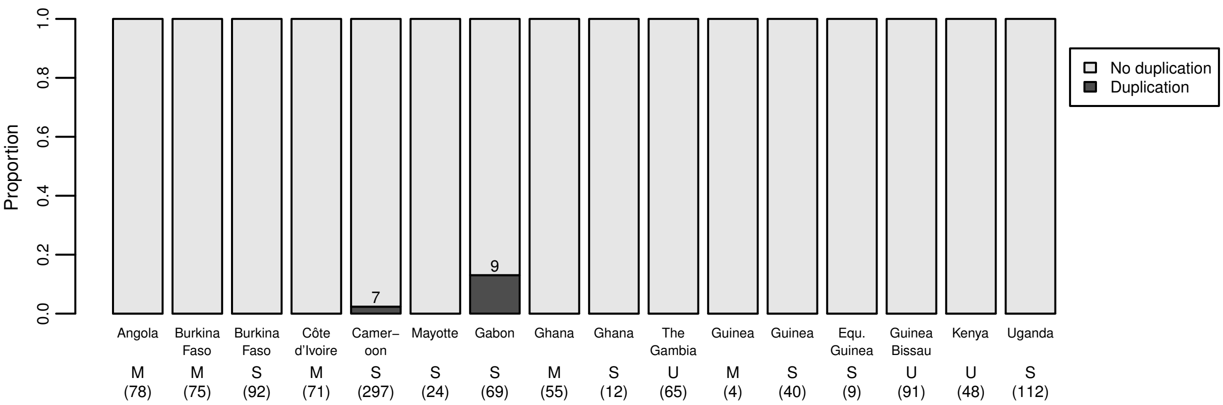

Fig. GST_S10: Barplot showing the proportion of samples that carry the Gstue_Dup5 duplication in each of the Phase 2 populations. Numbers above the dark grey bars indicate the absolute number of samples carrying the duplication. S = *Anopheles gambiae*, M = *Anopheles coluzzi*, U = species undetermined. Numbers in brackets indicate the total number of samples from that population.

Gstue_Dup5 was not associated with any discordant read pairs. Reads were found that were soft-clipped at the start point (28593122) and end point (28598971) of the duplication in samples that appear to carry Gstue_Dup5 based on the region of elevated coverage. The sequences of soft-clipped bases are consistently the same at each position but align to several places in the genome. We therefore called Gstue_Dup5 on the basis of the presence of soft-clipped reads at these two positions, whose clipped bases ended with `TACTG` and started with `ATTTA` respectively. This method produced one apparent false positive with no evidence of increased coverage. A deletion inside Gstue_Dup5 was supported by reads pairs that mapped facing towards each-other, with the first read mapping in the interval 28593600 - 28593900 and its mate mapping in the interval 28596500 - 28596800 (Fig. **GST_S11**).

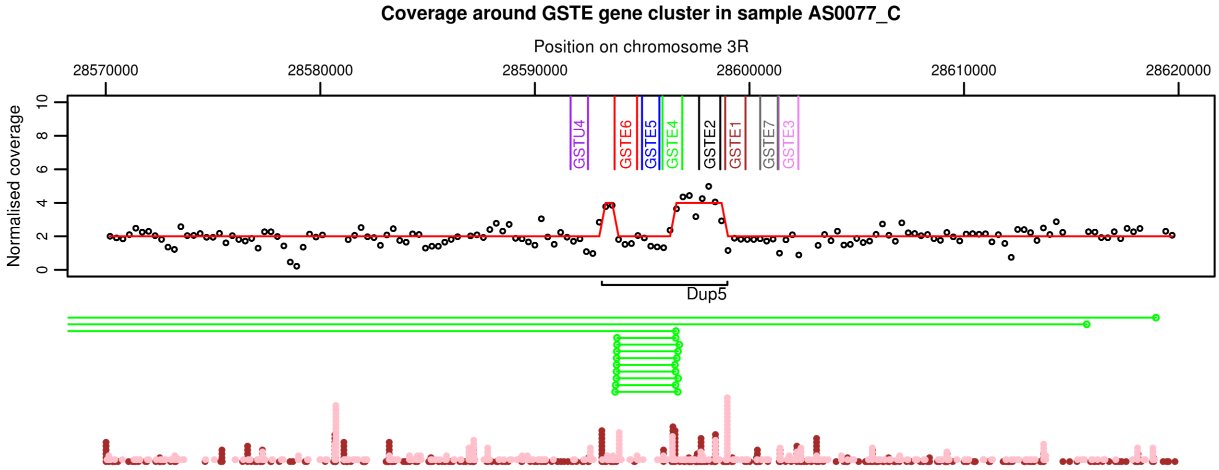

Fig. GST_S11: Example of coverage in an individual carrying the Gstue_Dup5 duplication. Open black circles indicate coverage at each position. The red line shows the HMM estimation of the coverage state at each position. Coloured vertical lines represent the positions of the GST genes. Reads soft-clipped before the alignment start point (dark brown points) and after the alignment end point (light brown points) are present at the start and end points of the duplication (clipped at positions 28593122 and 28598971 respectively). Green points joined by horizontal lines indicate pairs of reads that mapped facing each-other but more than 1000bp apart, indicating a possible deletion.

Gstue_Dup5 was found in *An. gambiae* from Cameroon and Gabon (Table GST_S5.1). Estimates of copy number indicated that copy number was consistently 1 (or 0 in samples where the HMM did not detect a duplication) in Cameroon and 4 in Gabon (Table GST_S5.2), suggesting that Gstue_Dup5 might be a duplication in Cameroon and a triplication in Gabon.

Table GST_S5.1: Coverage calls for Gstue_Dup5. NAs were produced if coverage was too variable or if the duplication completely overlapped with another duplication whose coverage could also not be called.

copy number	AO col	BF col	BF gam	CI col	CM gam	FR gam	GA gam	GH col	GH gam	GM	GN col	GN gam	GQ gam	GW	KE	UG gam
0	78	75	92	71	293	24	60	55	12	65	4	40	9	91	48	112
1	0	0	0	0	4	0	0	0	0	0	0	0	0	0	0	0
2	0	0	0	0	0	0	9	0	0	0	0	0	0	0	0	0

Table GST_S5.2: Coverage calls for all duplications in individuals that carry Gstue_Dup5.

	Dup 0	Dup 1	Dup 2	Dup 3	Dup 4	Dup 5	Dup 6	Dup 7	Dup 8	Dup 9	Dup 10	Dup 11
AN0030_C	0	0	0	0	0	1	0	0	0	0	0	0
AN0045_C	0	0	0	0	0	0	0	0	0	0	0	0
AN0061_C	0	0	0	0	0	1	0	0	0	0	0	0
AN0079_C	0	0	0	0	0	0	0	0	0	0	0	0
AN0134_C	0	0	0	0	0	1	0	0	0	0	0	0
AN0149_C	0	0	0	0	0	0	0	0	0	0	0	0
AN0252_C	0	0	0	0	0	1	0	0	0	0	0	0
AS0006_C	0	0	0	0	0	2	0	0	0	0	0	0
AS0033_C	0	0	0	0	0	2	0	0	0	0	0	0
AS0035_C	0	0	0	0	0	2	0	0	0	0	0	0
AS0040_C	0	0	0	0	0	2	0	0	0	0	0	0
AS0047_C	0	0	0	0	0	2	0	0	0	0	0	0
AS0052_C	0	0	0	0	0	2	0	0	0	0	0	0
AS0058_C	0	0	0	0	0	2	0	0	0	0	0	0
AS0076_C	0	0	0	0	0	2	0	0	0	0	0	0
AS0077_C	0	0	0	0	0	2	0	0	0	0	0	0

Duplication type 6

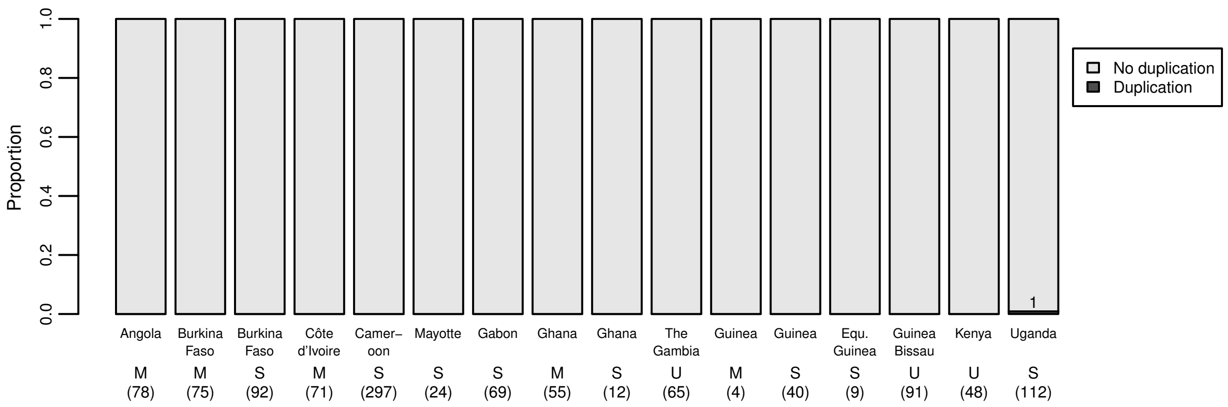

Fig. GST_S12: Barplot showing the proportion of samples that carry the Gstue_Dup6 duplication in each of the Phase 2 populations. Numbers above the dark grey bars indicate the absolute number of samples carrying the duplication. S = *Anopheles gambiae*, M = *Anopheles coluzzi*, U = species undetermined. Numbers in brackets indicate the total number of samples from that population.

Gstue_Dup6 was supported by face-away read pairs whose forward-facing read mapped in the interval 28601900 - 28602200 and whose reverse-facing read mapped in the interval 28596250 - 28596550 (Fig. **GST_S13**). Gstue_Dup6 was also supported by reads soft-clipped at the breakpoints (positions 28596241 and 28602177), with the clipped bases at each breakpoint aligning at the other breakpoint.

Gstue_Dup6 breakpoint:

```
GGCGGGTACTGTACAACA C GAAGCAATGCTGGCGATG
  end of the dup ^   ^ start of the dup
 position 28602175   position 28596243
```

The C could sit on either side of the breakpoint.

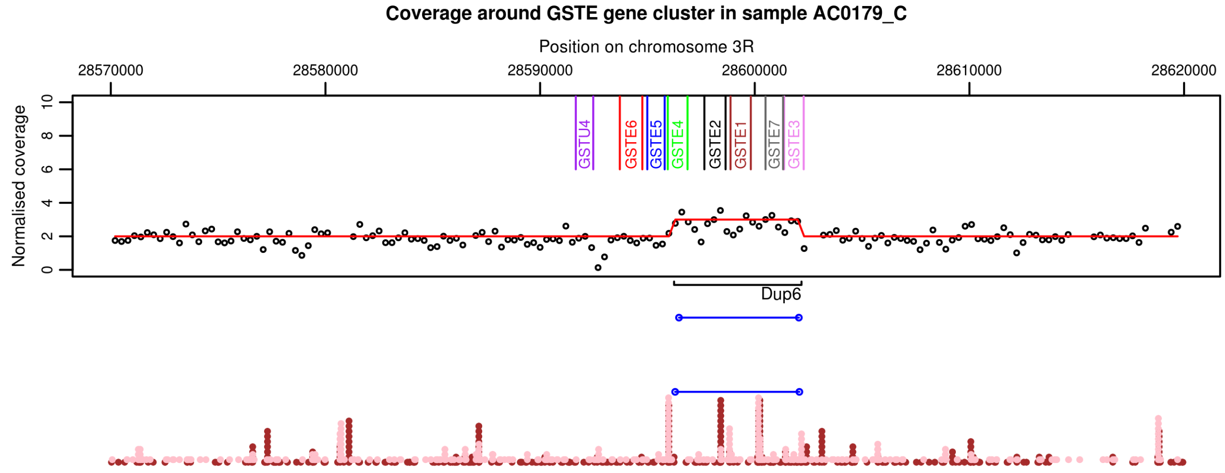

Fig. GST_S13: Coverage in the individual carrying the Gstue_Dup6 duplication. Open black circles indicate coverage at each position. The red line shows the HMM estimation of the coverage state at each position. Coloured vertical lines represent the positions of the GST genes. Pairs of blue points connected by lines indicate pairs of face-away reads. Reads soft-clipped before the alignment start point (dark brown points) and after the alignment end point (light brown points) are present at the start and end points of the duplication (clipped at positions 28595162 and 28599081 respectively). In each case, the clipped bases align to the other end of the duplication, as expected.

The only sample that carries Gstue_Dup6 has a copy number of 1 (Tables GST_S6.1 & 6.2). Gstue_Dup6 is therefore a single copy duplication and the sample is heterozygous.

Table GST_S6.1: Coverage calls for Gstue_Dup6. NAs were produced if coverage was too variable or if the duplication completely overlapped with another duplication whose coverage could also not be called.

copy number	AO col	BF col	BF gam	CI col	CM gam	FR gam	GA gam	GH col	GH gam	GM	GN col	GN gam	GQ gam	GW	KE	UG gam
0	78	75	92	71	297	24	69	55	12	65	4	40	9	91	48	111
1	0	0	0	0	0	0	0	0	0	0	0	0	0	0	0	1

Table GST_S6.2: Coverage calls for all duplications in individuals that carry Gstue_Dup6.

	Dup 0	Dup 1	Dup 2	Dup 3	Dup 4	Dup 5	Dup 6	Dup 7	Dup 8	Dup 9	Dup 10	Dup 11
AC0179_C	0	0	0	0	0	0	1	0	0	0	0	0

Duplication type 7

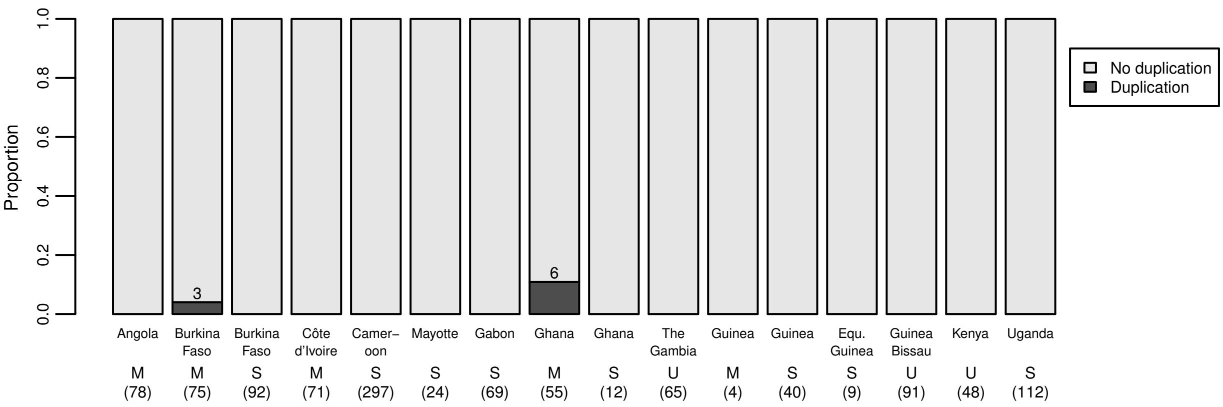

Fig. GST_S14: Barplot showing the proportion of samples that carry the Gstue_Dup7 duplication in each of the Phase 2 populations. Numbers above the dark grey bars indicate the absolute number of samples carrying the duplication. S = *Anopheles gambiae*, M = *Anopheles coluzzi*, U = species undetermined. Numbers in brackets indicate the total number of samples from that population.

Gstue_Dup7 was supported by reads whose mates did not map to chromosome 3R (Fig. **GST_S15**). The start point was associated with reads mapping in the interval 28597400 - 28597700 and whose mates mapped to some region on another chromosome, for example 2L:3696450-3696750. The end point was associated with reads mapping in the interval 28603950 - 28604250 and whose mates mapped to some region on another chromosome, for example UNKN:26597300-26597600. Reads soft-clipped at the breakpoints (positions 28597504 and 28604250) were also present (Fig. **GST_S15**), with the clipped bases aligning to many different places in the genome.

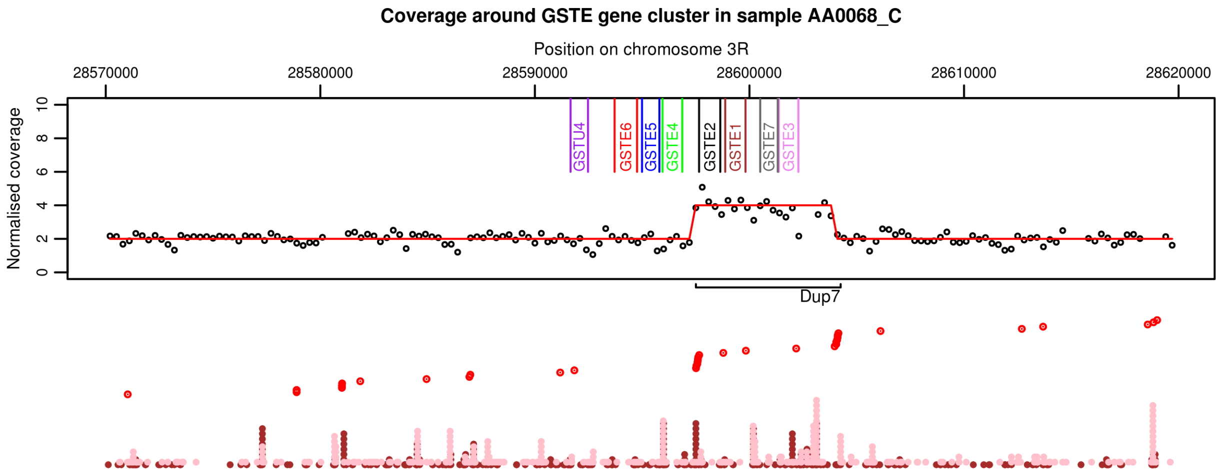

Fig. GST_S15: Example of coverage in an individual carrying the Gstue_Dup7 duplication. Open black circles indicate coverage at each position. The red line shows the HMM estimation of the coverage state at each position. Coloured vertical lines represent the positions of the GST genes. Red points below the graph indicate reads whose mapped mapped to other chromosomes. Reads soft-clipped before the alignment start point (dark brown points) and after the alignment end point (light brown points) are present at the start and end points of the duplication (clipped at positions 28597504 and 28604250 respectively).

All of the samples in which Gstue_Dup7 was found are in *An. coluzzii* from Burkina Faso and Ghana (Table GST_S7.1). Estimates of copy number for Gstue_Dup7 in these populations indicated that no sample had a copy number higher than 2. Assuming that samples with a copy number of 3 are heterozygotes and that samples with a copy number of 2 are homozygote for Gstue_Dup7 (Table GST_S7.2), the allele distribution is not consistent with HW expectations ($P = 0.04$ and $P < 0.0001$ in Burkina Faso and Ghana respectively). It is therefore likely that Gstue_Dup7 exists as both a duplication and a triplication

Table GST_S7.1: Coverage calls for Gstue_Dup7. NAs were produced if coverage was too variable or if the duplication completely overlapped with another duplication whose coverage could also not be called.

copy number	AO col	BF col	BF gam	CI col	CM gam	FR gam	GA gam	GH col	GH gam	GM	GN col	GN gam	GQ gam	GW	KE	UG gam
0	78	72	92	71	297	24	69	49	12	65	4	40	9	91	48	112
1	0	2	0	0	0	0	0	1	0	0	0	0	0	0	0	0
2	0	1	0	0	0	0	0	5	0	0	0	0	0	0	0	0

Table GST_S7.2: Coverage calls for all duplications in individuals that carry Gstue_Dup7.

	Dup 0	Dup 1	Dup 2	Dup 3	Dup 4	Dup 5	Dup 6	Dup 7	Dup 8	Dup 9	Dup 10	Dup 11
AA0066_C	0	0	0	0	0	0	0	2	0	0	0	0
AA0068_C	0	0	0	0	0	0	0	2	0	0	0	0
AA0074_C	0	0	0	0	0	0	0	2	0	0	0	0
AA0097_C	0	0	0	0	0	0	0	1	0	0	0	0
AA0100_C	0	0	0	0	0	0	0	2	0	0	0	0
AA0135_C	0	0	0	0	0	0	0	2	0	0	0	0
AB0139_C	0	NA	0	0	0	0	0	1	0	0	0	0
AB0229_C	0	0	0	0	0	0	0	2	0	0	0	0
AB0266_C	0	0	0	0	0	0	0	1	0	0	0	0

Duplication type 8

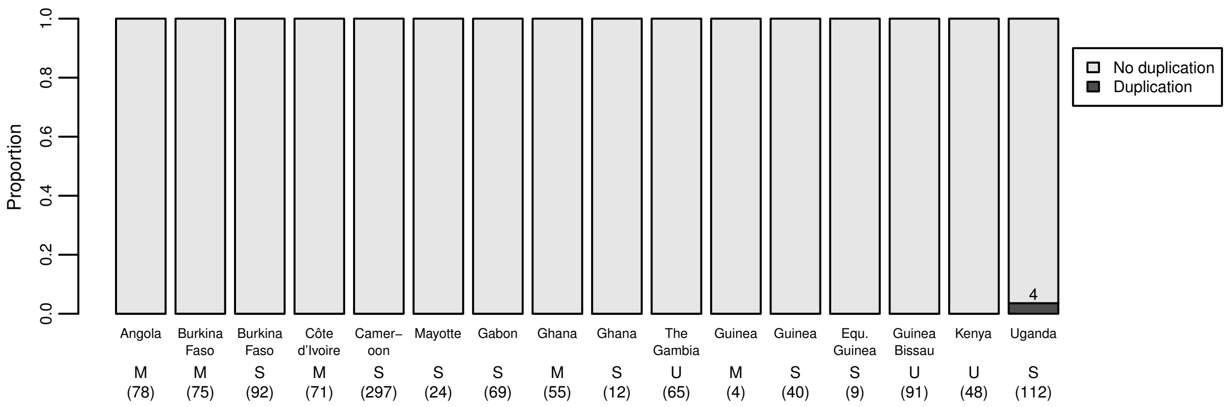

Fig. GST_S16: Barplot showing the proportion of samples that carry the Gstue_Dup8 duplication in each of the Phase 2 populations. Numbers above the dark grey bars indicate the absolute number of samples carrying the duplication. S = *Anopheles gambiae*, M = *Anopheles coluzzi*, U = species undetermined. Numbers in brackets indicate the total number of samples from that population.

Gstue_Dup8 was not associated with any discordant read pairs. Reads were found that were soft-clipped at the start point (28594797) and end point (28602349) of the duplication in samples that appear to carry Gstue_Dup8 based on the region of coverage increase. The sequences of clipped bases are consistently the same at each position, but align to several places in the genome. We therefore called Gstue_Dup8 on the basis of the presence of soft-clipped reads at these two positions, whose clipped bases ended with `CCCTG` and started with `CAGGG` respectively. Interestingly, the bases soft-clipped before the start point are the reverse complement of those soft-clipped after the end point:

```
   reverse complement of bases soft clipped before start point
CAGGGTTTCACACTTTATCTCAAGACCGCGGGACCCCTTCCCGAATCTGTCTTAGCCAAAGCCAAGATTGCGGTATGAT
CAGGGTTTCACACTTTATCTCAAGACCGCGGGACCCCTTCCCGAATCTGTCTAAGCCAAAGCCAAGACTGCGGTATGA
   bases soft clipped after end point
```

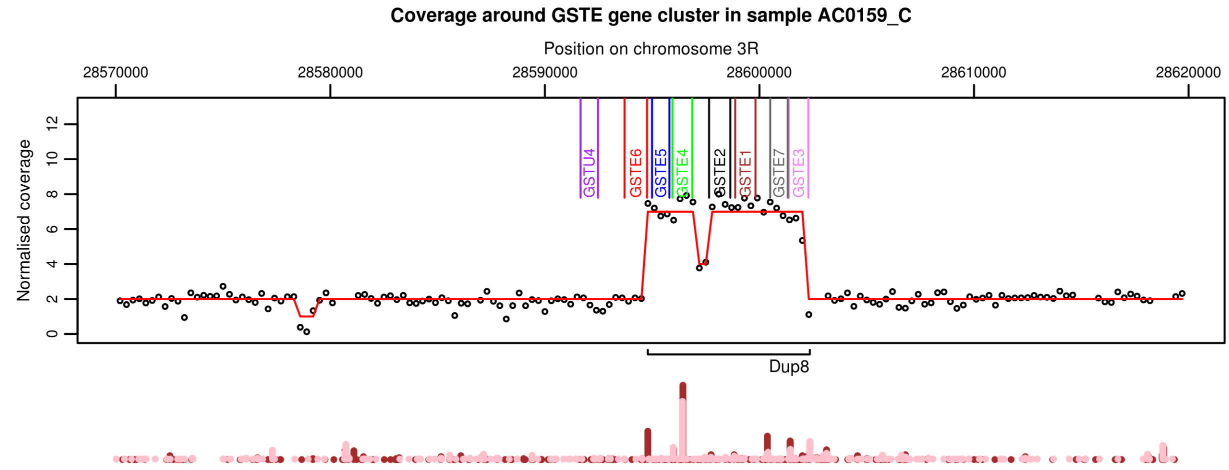

Fig. GST_S17: Example of coverage in an individual carrying the Gstue_Dup8 duplication. Open black circles indicate coverage at each position. The red line shows the HMM estimation of the coverage state at each position. Coloured vertical lines represent the positions of the GST genes. Reads soft-clipped before the alignment start point (dark brown points) and after the alignment end point (light brown points) are present at the start and end points of the duplication (clipped at positions 28594797 and 28602349 respectively).

Coverage calls could be obtained for three out of the four samples in which Gstue_Dup8 was found (Tables GST_S8.1 & 8.2). Estimates of copy number indicated that copy number ranges from 3 to 5, suggesting that Gstue_Dup8 is an amplification of more than two fold. It is not possible to confidently describe the duplication and thus call alleles from these data.

Table GST_S8.1: Coverage calls for Gstue_Dup8. NAs were produced if coverage was too variable or if the duplication completely overlapped with another duplication whose coverage could also not be called.

copy number	AO col	BF col	BF gam	CI col	CM gam	FR gam	GA gam	GH col	GH gam	GM	GN col	GN gam	GQ gam	GW	KE	UG gam
NA	0	0	0	0	0	0	0	0	0	0	0	0	0	0	0	1
0	78	75	92	71	297	24	69	55	12	65	4	40	9	91	48	108
3	0	0	0	0	0	0	0	0	0	0	0	0	0	0	0	1
4	0	0	0	0	0	0	0	0	0	0	0	0	0	0	0	1
5	0	0	0	0	0	0	0	0	0	0	0	0	0	0	0	1

Table GST_S8.2: Coverage calls for all duplications in individuals that carry Gstue_Dup8.

	Dup 0	Dup 1	Dup 2	Dup 3	Dup 4	Dup 5	Dup 6	Dup 7	Dup 8	Dup 9	Dup 10	Dup 11
AC0091_C	0	0	0	0	0	0	0	0	3	0	0	0
AC0159_C	0	0	0	0	0	0	0	0	5	0	0	0
AC0173_C	0	0	0	0	0	0	0	0	4	0	0	0
AC0183_C	0	0	NA	1	0	0	0	0	NA	0	0	0

Duplication type 9

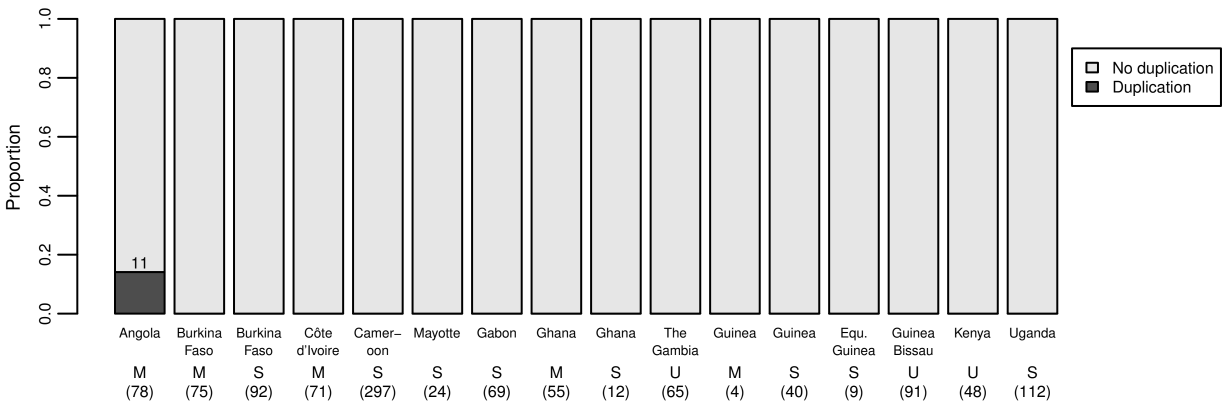

Fig. GST_S18: Barplot showing the proportion of samples that carry the Gstue_Dup9 duplication in each of the Phase 2 populations. Numbers above the dark grey bars indicate the absolute number of samples carrying the duplication. S = *Anopheles gambiae*, M = *Anopheles coluzzi*, U = species undetermined. Numbers in brackets indicate the total number of samples from that population.

Gstue_Dup9 was supported by face-away read pairs whose forward-facing read mapped in the interval 28591050 - 28591350 and whose reverse-facing read mapped in the interval 28600850 - 28601150 (Fig. **GST_S19**). Gstue_Dup9 was also supported by reads soft-clipped at the breakpoints (positions 28591140 and 28601188), with the clipped bases at each breakpoint aligning at the other breakpoint.

Gstue_Dup9 breakpoint:

```
GCTGGACGAGTCCAAGTT   GATGAAGAAGAGAG   ATGAAATGTGTGCGTCATG
  end of the dup ^    inserted seq    ^ start of the dup
 position 28601187                    position 28591144
```

The sequence `GATGAAGAAGAGAG` is inserted between the sequences on either side of the breakpoint.

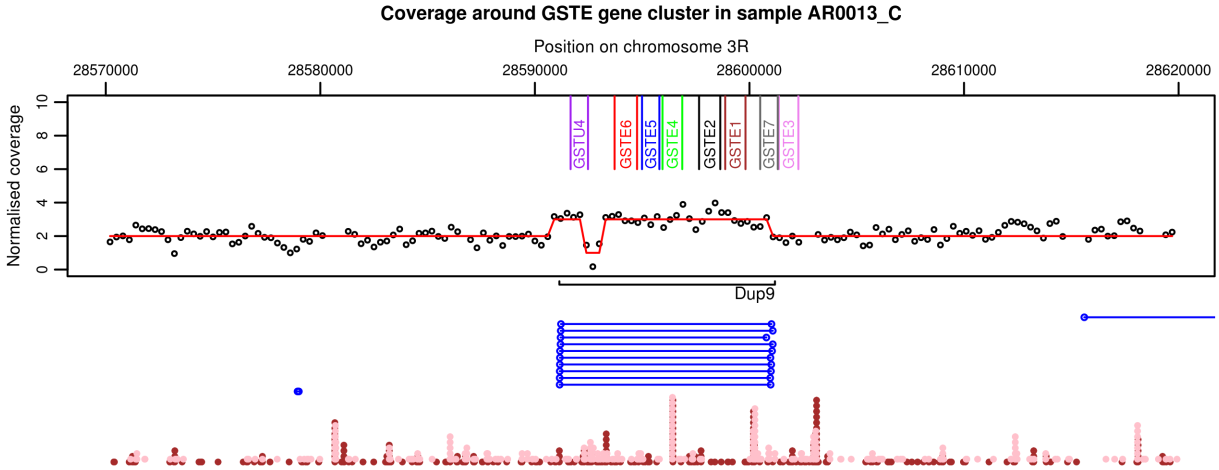

Fig. GST_S19: Example of coverage in an individual carrying the Gstue_Dup9 duplication. Open black circles indicate coverage at each position. The red line shows the HMM estimation of the coverage state at each position. Coloured vertical lines represent the positions of the GST genes. Pairs of blue points connected by lines indicate pairs of face-away reads. Reads soft-clipped before the alignment start point (dark brown points) and after the alignment end point (light brown points) are present at the start and end points of the duplication (clipped at positions 28591140 and 28601188 respectively). In each case, the clipped bases align to the other end of the duplication, as expected.

Gstue_Dup9 was found in 11 samples from Angola (Table GST_S9.1). Estimates of copy number in this population indicated that no sample has a copy number higher than 1 (Table GST_S9.2). Assuming samples with a copy number of 1 are heterzygotes for Gstue_Dup9, the allele distribution is consistent with HW expectations ($P = 1$).

Table GST_S9.1: Coverage calls for Gstue_Dup9. NAs were produced if coverage was too variable or if the duplication completely overlapped with another duplication whose coverage could also not be called.

copy number	AO col	BF col	BF gam	CI col	CM gam	FR gam	GA gam	GH col	GH gam	GM	GN col	GN gam	GQ gam	GW	KE	UG gam
NA	2	0	0	0	0	0	0	0	0	0	0	0	0	0	0	0
0	69	75	92	71	297	24	69	55	12	65	4	40	9	91	48	112
1	7	0	0	0	0	0	0	0	0	0	0	0	0	0	0	0

Table GST_S9.2: Coverage calls for all duplications in individuals that carry Gstue_Dup9.

	Dup 0	Dup 1	Dup 2	Dup 3	Dup 4	Dup 5	Dup 6	Dup 7	Dup 8	Dup 9	Dup 10	Dup 11
AR0013_C	0	0	0	0	0	0	0	0	0	1	0	0
AR0015_C	0	0	0	0	0	0	0	0	0	1	0	0
AR0022_C	0	0	0	0	0	0	0	0	0	1	0	0
AR0026_C	0	0	0	0	0	0	0	0	0	0	0	0
AR0036_C	0	0	0	0	0	0	0	0	0	0	0	0
AR0049_C	0	0	0	0	0	0	0	0	0	1	0	0
AR0069_C	0	0	0	0	0	0	0	0	0	1	0	0
AR0079_C	0	0	0	0	0	0	0	0	0	NA	0	0
AR0082_C	0	0	0	0	0	0	0	0	0	NA	0	0
AR0097_C	0	0	0	0	0	0	0	0	0	1	0	0
AR0098_C	0	0	0	0	0	0	0	0	0	1	0	0

Duplication type 10

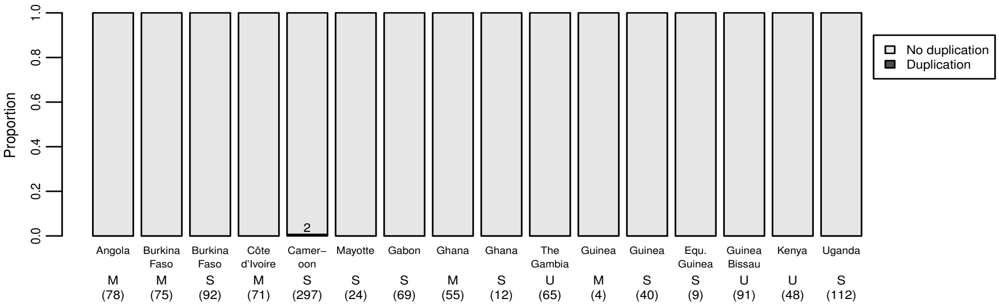

Fig. GST_S20: Barplot showing the proportion of samples that carry the Gstue_Dup10 duplication in each of the Phase 2 populations. Numbers above the dark grey bars indicate the absolute number of samples carrying the duplication. S = *Anopheles gambiae*, M = *Anopheles coluzzi*, U = species undetermined. Numbers in brackets indicate the total number of samples from that population.

Gstue_Dup10 was found in two individuals. In the first, it was associated with face-away read pairs whose forward-facing read mapped in the interval 28593690 - 28593750 and whose reverse-facing read mapped in the interval 28603500 - 28603645 (Fig. **GST_S21**). Two soft-clipped reads also mapped to the end of the duplication (28603786). The clipped bases from these reads began with the sequence `AAGACGCAGCGAATGTACTTTTTCGCT`, before continuing with a sequence that mapped to the start of the duplication. The second individual lacked the face-away reads and the soft-clipped reads at the end of the duplication, but had three soft-clipped reads that mapped to the start of the duplication (28593642) and whose clipped bases started with the same inserted sequence as above, followed by a sequence that mapped to the same point mapped by the first individual's soft-clipped reads. We therefore consider that the duplication breakpoint looks as follows.

Gstue_Dup10 breakpoint:

```
ACGCTTTTAATTTTATTC AAGACGCAGCGAATGTACTTTTTCGCT AAGAGGACGCAGCGGGTC
  end of the dup ^          inserted seq         ^ start of the dup
 position 28603785                               position 28593643
```

The sequence `AAGACGCAGCGAATGTACTTTTTCGCT` is inserted between the sequences on either side of the breakpoint.

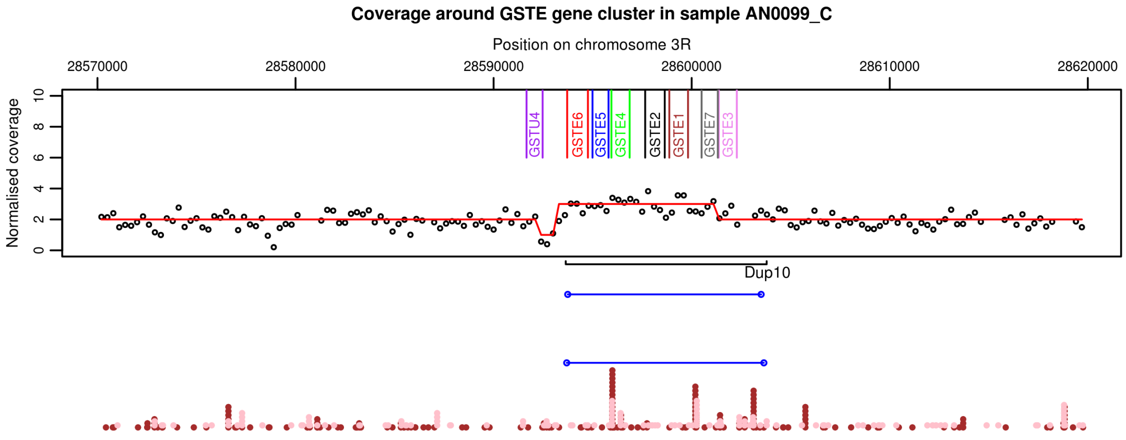

Fig. GST_S21: Example of coverage in an individual carrying the Gstue_Dup10 duplication. Open black circles indicate coverage at each position. The red line shows the HMM estimation of the coverage state at each position. Coloured vertical lines represent the positions of the GST genes. Pairs of blue points connected by lines indicate pairs of face-away reads. Reads soft-clipped after the alignment end point (light brown points) are present at the end point of the duplication (clipped at position 28603786). Reads soft-clipped before the alignment start point (dark brown points) are not found at the start point in this sample, but are present at position 28593642 in another sample carrying the same duplication. The clipped bases from both of these breakpoints align to the other end of the duplication, as expected, but with an inserted sequence of 27bp.

The two samples that carry Gstue_Dup10 have a copy number of 1 (Tables GST_S10.1 & 10.2). Gstue_Dup10 is therefore a single copy duplication and the samples are heterozygous.

Table GST_S10.1: Coverage calls for Gstue_Dup10. NAs were produced if coverage was too variable or if the duplication completely overlapped with another duplication whose coverage could also not be called.

copy number	AO col	BF col	BF gam	CI col	CM gam	FR gam	GA gam	GH col	GH gam	GM	GN col	GN gam	GQ gam	GW	KE	UG gam
0	78	75	92	71	295	24	69	55	12	65	4	40	9	91	48	112
1	0	0	0	0	2	0	0	0	0	0	0	0	0	0	0	0

Table GST_S10.2: Coverage calls for all duplications in individuals that carry Gstue_Dup10.

	Dup 0	Dup 1	Dup 2	Dup 3	Dup 4	Dup 5	Dup 6	Dup 7	Dup 8	Dup 9	Dup 10	Dup 11
AN0047_C	0	0	0	0	0	0	0	0	0	0	1	0
AN0099_C	0	0	0	0	0	0	0	0	0	0	1	0

Duplication type 11

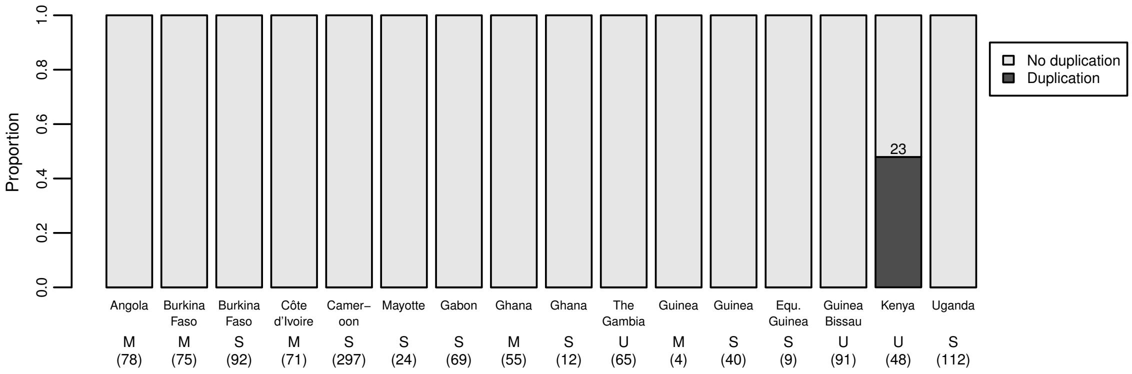

Fig. GST_S22: Barplot showing the proportion of samples that carry the Gstue_Dup11 duplication in each of the Phase 2 populations. Numbers above the dark grey bars indicate the absolute number of samples carrying the duplication. S = *Anopheles gambiae*, M = *Anopheles coluzzi*, U = species undetermined. Numbers in brackets indicate the total number of samples from that population.

Gstue_Dup11 was supported by reads whose mates did not map to chromosome 3R (Fig. **GST_S23**). The start point was associated with reads mapping in the interval 28581250 - 28581550 and whose mates mapped to some region on another chromosome, for example UNKN:29210650-29210950. The end point was associated with reads mapping in the interval 28604650 - 28604950 and whose mates mapped to some region on another chromosome, for example UNKN:29210650-29210950. Reads were found that were soft-clipped at the start point (28581256) and end point (28604994) of the duplication in samples that appear to carry Gstue_Dup11 based on the region of coverage increase. The sequences of clipped bases are consistently the same at each position, but align to several places in the genome. We therefore also called Gstue_Dup11 on the basis of the presence of soft-clipped reads at these two positions, whose clipped bases ended with `TTACC` and started with `GGTAA` respectively. Interestingly, the bases-soft clipped before the start point were the reverse complement of those soft-clipped after the end point:

```
   reverse complement of bases soft clipped before start point
GGTAAAAGATTTTTTAAGGCCCCCCCTATGGCAATCGAAGCAGCCCTGACAAGAAAAACGAGATAGTGTGCGGTTGATCG
GGTAAAAGATTTTTTAAGGCCCCCCCTATGGCAATCGAAGCAGCCCTGACAAGAAAAACGAGATAGTGTGCGGTTGATCG
   bases soft clipped after end point
```

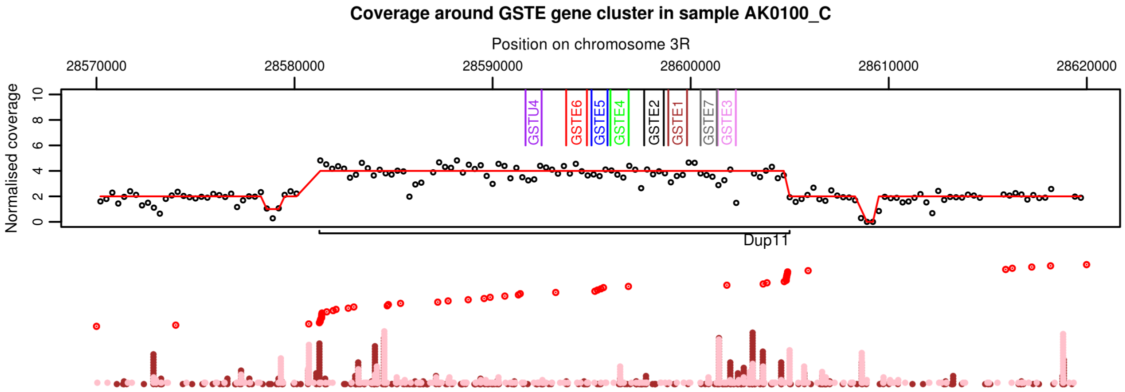

Fig. GST_S23: Example of coverage in an individual carrying the Gstue_Dup11 duplication. Open black circles indicate coverage at each position. The red line shows the HMM estimation of the coverage state at each position. Coloured vertical lines represent the positions of the GST genes. Reads soft-clipped before the alignment start point (dark brown points) and after the alignment end point (light brown points) are present at the start and end points of the duplication (clipped at positions 28581256 and 28604994 respectively).

Gstue_Dup11 was found only in KE and a lot of samples fail to get coverage calls (Table GST_S11.1), perhaps because of the erratic coverage in samples from this population. Where esimations of copy number could be made, they ranged from 3 to 4. Assuming that samples with a copy number of 1 are heterozygotes and that samples with a copy number of 2 are homozygotes for Gstue_Dup11 (Table GST_S11.2), the allele distribution is consistent with HW expectations ($P = 1$).

Table GST_S11.1: Coverage calls for Gstue_Dup11. NAs were produced if coverage was too variable or if the duplication completely overlapped with another duplication whose coverage could also not be called.

copy number	AO col	BF col	BF gam	CI col	CM gam	FR gam	GA gam	GH col	GH gam	GM	GN col	GN gam	GQ gam	GW	KE	UG gam
NA	0	0	0	0	0	0	0	0	0	0	0	0	0	0	9	0
0	78	75	92	71	297	24	69	55	12	65	4	40	9	91	25	112
1	0	0	0	0	0	0	0	0	0	0	0	0	0	0	13	0
2	0	0	0	0	0	0	0	0	0	0	0	0	0	0	1	0

Table GST_S11.2: Coverage calls for all duplications in individuals that carry Gstue_Dup11.

	Dup 0	Dup 1	Dup 2	Dup 3	Dup 4	Dup 5	Dup 6	Dup 7	Dup 8	Dup 9	Dup 10	Dup 11
AK0062_C	0	0	0	0	0	0	0	0	0	0	0	NA
AK0073_C	0	0	0	0	0	0	0	0	0	0	0	1
AK0074_C	0	0	0	0	0	0	0	0	0	0	0	1
AK0076_C	0	0	0	0	0	0	0	0	0	0	0	1
AK0079_C	0	0	0	0	0	0	0	0	0	0	0	1
AK0080_C	0	0	0	0	0	0	0	0	0	0	0	1
AK0081_C	0	0	0	0	0	0	0	0	0	0	0	1
AK0087_C	0	0	0	0	0	0	0	0	0	0	0	NA
AK0089_C	0	0	0	0	0	0	0	0	0	0	0	NA
AK0090_C	0	0	0	0	0	0	0	0	0	0	0	NA
AK0091_C	0	0	0	0	0	0	0	0	0	0	0	NA
AK0093_C	0	0	0	0	0	0	0	0	0	0	0	NA
AK0095_C	0	0	0	0	0	0	0	0	0	0	0	NA
AK0096_C	0	0	0	0	0	0	0	0	0	0	0	1
AK0100_C	0	0	0	0	0	0	0	0	0	0	0	2
AK0101_C	0	0	0	0	0	0	0	0	0	0	0	1
AK0103_C	0	0	0	0	0	0	0	0	0	0	0	1
AK0104_C	0	0	0	0	0	0	0	0	0	0	0	1
AK0107_C	0	0	0	0	0	0	0	0	0	0	0	1
AK0108_C	0	0	0	0	0	0	0	0	0	0	0	1
AK0110_C	0	0	0	0	0	0	0	0	0	0	0	1
AK0117_C	0	0	0	0	0	0	0	0	0	0	0	NA
AK0127_C	0	0	0	0	0	0	0	0	0	0	0	NA

Table GST_S12: Coverage calls for all duplications in the Gstu-Gste cluster in all individuals.

	Dup 0	Dup 1	Dup 2	Dup 3	Dup 4	Dup 5	Dup 6	Dup 7	Dup 8	Dup 9	Dup 10	Dup 11
AA0040_C	0	0	0	0	0	0	0	0	0	0	0	0
AA0041_C	0	0	0	0	0	0	0	0	0	0	0	0
AA0042_C	0	0	0	0	0	0	0	0	0	0	0	0
AA0043_C	0	0	0	0	0	0	0	0	0	0	0	0
AA0044_C	0	0	0	0	0	0	0	0	0	0	0	0
AA0048_C	0	0	0	0	0	0	0	0	0	0	0	0
AA0049_C	0	0	0	0	0	0	0	0	0	0	0	0
AA0050_C	0	0	0	0	0	0	0	0	0	0	0	0
AA0051_C	0	0	0	0	0	0	0	0	0	0	0	0
AA0052_C	0	0	0	0	0	0	0	0	0	0	0	0
AA0053_C	0	0	0	0	0	0	0	0	0	0	0	0
AA0054_C	0	0	0	0	0	0	0	0	0	0	0	0
AA0055_C	0	0	0	0	0	0	0	0	0	0	0	0
AA0056_C	0	0	0	0	0	0	0	0	0	0	0	0
AA0060_C	0	0	0	0	0	0	0	0	0	0	0	0
AA0061_C	0	0	0	0	0	0	0	0	0	0	0	0
AA0063_C	0	0	0	0	0	0	0	0	0	0	0	0
AA0064_C	0	0	0	0	0	0	0	0	0	0	0	0
AA0066_C	0	0	0	0	0	0	0	2	0	0	0	0
AA0067_C	0	0	0	0	0	0	0	0	0	0	0	0
AA0068_C	0	0	0	0	0	0	0	2	0	0	0	0
AA0072_C	0	0	0	0	0	0	0	0	0	0	0	0
AA0073_C	0	0	0	0	0	0	0	0	0	0	0	0
AA0074_C	0	0	0	0	0	0	0	2	0	0	0	0
AA0075_C	0	0	0	0	0	0	0	0	0	0	0	0
AA0076_C	0	0	0	0	0	0	0	0	0	0	0	0
AA0077_C	0	0	0	0	0	0	0	0	0	0	0	0
AA0080_C	0	0	0	0	0	0	0	0	0	0	0	0
AA0084_C	0	0	0	0	0	0	0	0	0	0	0	0
AA0085_C	0	0	0	0	0	0	0	0	0	0	0	0
AA0086_C	0	0	0	0	0	0	0	0	0	0	0	0
AA0087_C	0	0	0	0	0	0	0	0	0	0	0	0
AA0088_C	0	0	0	0	0	0	0	0	0	0	0	0
AA0089_C	0	0	0	0	0	0	0	0	0	0	0	0
AA0090_C	0	0	0	0	0	0	0	0	0	0	0	0
AA0091_C	0	0	0	0	0	0	0	0	0	0	0	0
AA0096_C	0	0	0	0	0	0	0	0	0	0	0	0
AA0097_C	0	0	0	0	0	0	0	1	0	0	0	0
AA0098_C	0	0	0	0	0	0	0	0	0	0	0	0
AA0099_C	0	0	0	0	0	0	0	0	0	0	0	0
AA0100_C	0	0	0	0	0	0	0	2	0	0	0	0
AA0101_C	0	0	0	0	0	0	0	0	0	0	0	0
AA0102_C	0	0	0	0	0	0	0	0	0	0	0	0
AA0103_C	0	0	0	0	0	0	0	0	0	0	0	0
AA0104_C	0	0	0	0	0	0	0	0	0	0	0	0
AA0107_C	0	0	0	0	0	0	0	0	0	0	0	0
AA0108_C	0	0	0	0	0	0	0	0	0	0	0	0
AA0109_C	0	0	0	0	0	0	0	0	0	0	0	0
AA0110_C	0	0	0	0	0	0	0	0	0	0	0	0
AA0111_C	0	0	0	0	0	0	0	0	0	0	0	0
AA0113_C	0	0	0	0	0	0	0	0	0	0	0	0
AA0114_C	0	0	0	0	0	0	0	0	0	0	0	0
AA0115_C	0	0	0	0	0	0	0	0	0	0	0	0
AA0116_C	0	0	0	0	0	0	0	0	0	0	0	0
AA0122_C	0	0	0	0	0	0	0	0	0	0	0	0
AA0123_C	0	0	0	0	0	0	0	0	0	0	0	0
AA0124_C	0	0	0	0	0	0	0	0	0	0	0	0
AA0125_C	0	0	0	0	0	0	0	0	0	0	0	0
AA0127_C	0	0	0	0	0	0	0	0	0	0	0	0
AA0132_C	0	0	0	0	0	0	0	0	0	0	0	0
AA0133_C	0	0	0	0	0	0	0	0	0	0	0	0
AA0134_C	0	0	0	0	0	0	0	0	0	0	0	0
AA0135_C	0	0	0	0	0	0	0	2	0	0	0	0
AA0136_C	0	0	0	0	0	0	0	0	0	0	0	0
AA0139_C	0	0	0	0	0	0	0	0	0	0	0	0
AA0140_C	0	0	0	0	0	0	0	0	0	0	0	0

	Dup 0	Dup 1	Dup 2	Dup 3	Dup 4	Dup 5	Dup 6	Dup 7	Dup 8	Dup 9	Dup 10	Dup 11
AA0141_C	0	0	0	0	0	0	0	0	0	0	0	0
AB0085_C	0	0	0	0	0	0	0	0	0	0	0	0
AB0087_C	0	0	0	0	0	0	0	0	0	0	0	0
AB0088_C	0	1	0	0	0	0	0	0	0	0	0	0
AB0089_C	0	0	0	0	0	0	0	0	0	0	0	0
AB0090_C	0	0	0	0	0	0	0	0	0	0	0	0
AB0091_C	0	0	0	0	0	0	0	0	0	0	0	0
AB0092_C	0	0	0	0	0	0	0	0	0	0	0	0
AB0094_C	0	0	0	0	0	0	0	0	0	0	0	0
AB0095_C	0	1	0	0	0	0	0	0	0	0	0	0
AB0097_C	0	1	0	0	0	0	0	0	0	0	0	0
AB0098_C	0	0	0	0	0	0	0	0	0	0	0	0
AB0099_C	0	0	0	0	0	0	0	0	0	0	0	0
AB0100_C	0	0	0	0	0	0	0	0	0	0	0	0
AB0101_C	0	1	0	0	0	0	0	0	0	0	0	0
AB0103_C	0	0	0	0	0	0	0	0	0	0	0	0
AB0104_C	0	0	0	0	0	0	0	0	0	0	0	0
AB0108_C	0	0	0	0	0	0	0	0	0	0	0	0
AB0109_C	0	0	0	0	0	0	0	0	0	0	0	0
AB0110_C	0	0	0	0	0	0	0	0	0	0	0	0
AB0111_C	0	0	0	0	0	0	0	0	0	0	0	0
AB0112_C	0	0	0	0	0	0	0	0	0	0	0	0
AB0113_C	0	0	0	0	0	0	0	0	0	0	0	0
AB0114_C	0	0	0	0	0	0	0	0	0	0	0	0
AB0115_C	0	0	0	0	0	0	0	0	0	0	0	0
AB0117_C	0	0	0	0	0	0	0	0	0	0	0	0
AB0118_C	0	0	0	0	0	0	0	0	0	0	0	0
AB0119_C	0	0	0	0	0	0	0	0	0	0	0	0
AB0122_C	0	0	0	0	0	0	0	0	0	0	0	0
AB0123_C	0	3	0	0	0	0	0	0	0	0	0	0
AB0124_C	0	0	0	0	0	0	0	0	0	0	0	0
AB0126_C	0	0	0	0	0	0	0	0	0	0	0	0
AB0127_C	0	0	0	0	0	0	0	0	0	0	0	0
AB0128_C	0	0	0	0	0	0	0	0	0	0	0	0
AB0129_C	0	0	0	0	0	0	0	0	0	0	0	0
AB0130_C	0	0	0	0	0	0	0	0	0	0	0	0
AB0133_C	0	0	0	0	0	0	0	0	0	0	0	0
AB0134_C	0	0	0	0	0	0	0	0	0	0	0	0
AB0135_C	0	0	0	0	0	0	0	0	0	0	0	0
AB0136_C	0	0	0	0	0	0	0	0	0	0	0	0
AB0137_C	0	0	0	0	0	0	0	0	0	0	0	0
AB0138_C	0	1	0	0	0	0	0	0	0	0	0	0
AB0139_C	0	NA	0	0	0	0	0	1	0	0	0	0
AB0140_C	0	0	0	0	0	0	0	0	0	0	0	0
AB0142_C	0	0	0	0	0	0	0	0	0	0	0	0
AB0143_C	0	0	0	0	0	0	0	0	0	0	0	0
AB0145_C	0	0	0	0	0	0	0	0	0	0	0	0
AB0146_C	0	0	0	0	0	0	0	0	0	0	0	0
AB0147_C	0	0	0	0	0	0	0	0	0	0	0	0
AB0148_C	0	0	0	0	0	0	0	0	0	0	0	0
AB0150_C	0	0	0	0	0	0	0	0	0	0	0	0
AB0151_C	0	0	0	0	0	0	0	0	0	0	0	0
AB0153_C	0	0	0	0	0	0	0	0	0	0	0	0
AB0155_C	0	0	0	0	0	0	0	0	0	0	0	0
AB0157_C	0	0	0	0	0	0	0	0	0	0	0	0
AB0158_C	0	0	0	0	0	0	0	0	0	0	0	0
AB0159_C	0	0	0	0	0	0	0	0	0	0	0	0
AB0160_C	0	0	0	0	0	0	0	0	0	0	0	0
AB0161_C	0	0	0	0	0	0	0	0	0	0	0	0
AB0162_C	0	0	0	0	0	0	0	0	0	0	0	0
AB0164_C	0	0	0	0	0	0	0	0	0	0	0	0
AB0165_C	0	0	0	0	0	0	0	0	0	0	0	0
AB0166_C	0	0	0	0	0	0	0	0	0	0	0	0
AB0167_C	0	0	0	0	0	0	0	0	0	0	0	0
AB0169_C	0	0	0	0	0	0	0	0	0	0	0	0
AB0170_C	0	0	0	0	0	0	0	0	0	0	0	0
AB0171_C	0	0	0	0	0	0	0	0	0	0	0	0
AB0172_C	0	0	0	0	0	0	0	0	0	0	0	0

	Dup 0	Dup 1	Dup 2	Dup 3	Dup 4	Dup 5	Dup 6	Dup 7	Dup 8	Dup 9	Dup 10	Dup 11
AB0173_C	0	0	0	0	0	0	0	0	0	0	0	0
AB0174_C	0	0	0	0	0	0	0	0	0	0	0	0
AB0175_C	0	0	0	0	0	0	0	0	0	0	0	0
AB0176_C	0	0	0	0	0	0	0	0	0	0	0	0
AB0177_C	0	0	0	0	0	0	0	0	0	0	0	0
AB0178_C	0	0	0	0	0	0	0	0	0	0	0	0
AB0179_C	0	0	0	0	0	0	0	0	0	0	0	0
AB0181_C	0	0	0	0	0	0	0	0	0	0	0	0
AB0182_C	0	1	0	0	0	0	0	0	0	0	0	0
AB0183_C	0	0	0	0	0	0	0	0	0	0	0	0
AB0184_C	0	0	0	0	0	0	0	0	0	0	0	0
AB0185_C	0	0	0	0	0	0	0	0	0	0	0	0
AB0186_C	0	0	0	0	0	0	0	0	0	0	0	0
AB0187_C	0	0	0	0	0	0	0	0	0	0	0	0
AB0188_C	0	1	0	0	0	0	0	0	0	0	0	0
AB0189_C	0	0	0	0	0	0	0	0	0	0	0	0
AB0190_C	0	0	0	0	0	0	0	0	0	0	0	0
AB0191_C	0	0	0	0	0	0	0	0	0	0	0	0
AB0192_C	0	0	0	0	0	0	0	0	0	0	0	0
AB0195_C	0	0	0	0	0	0	0	0	0	0	0	0
AB0196_C	0	0	0	0	0	0	0	0	0	0	0	0
AB0197_C	0	0	0	0	0	0	0	0	0	0	0	0
AB0198_C	0	0	0	0	0	0	0	0	0	0	0	0
AB0199_C	0	0	0	0	0	0	0	0	0	0	0	0
AB0200_C	0	0	0	0	0	0	0	0	0	0	0	0
AB0201_C	0	0	0	0	0	0	0	0	0	0	0	0
AB0202_C	0	0	0	0	0	0	0	0	0	0	0	0
AB0203_C	0	0	0	0	0	0	0	0	0	0	0	0
AB0204_C	0	1	0	0	0	0	0	0	0	0	0	0
AB0205_C	0	0	0	0	0	0	0	0	0	0	0	0
AB0206_C	0	0	0	0	0	0	0	0	0	0	0	0
AB0207_C	0	0	0	0	0	0	0	0	0	0	0	0
AB0208_C	0	0	0	0	0	0	0	0	0	0	0	0
AB0209_C	0	0	0	0	0	0	0	0	0	0	0	0
AB0210_C	0	0	0	0	0	0	0	0	0	0	0	0
AB0211_C	0	0	0	0	0	0	0	0	0	0	0	0
AB0212_C	0	0	0	0	0	0	0	0	0	0	0	0
AB0213_C	0	0	0	0	0	0	0	0	0	0	0	0
AB0215_C	0	1	0	0	0	0	0	0	0	0	0	0
AB0217_C	0	0	0	0	0	0	0	0	0	0	0	0
AB0218_C	0	0	0	0	0	0	0	0	0	0	0	0
AB0219_C	0	0	0	0	0	0	0	0	0	0	0	0
AB0221_C	0	0	0	0	0	0	0	0	0	0	0	0
AB0222_C	0	0	0	0	0	0	0	0	0	0	0	0
AB0223_C	0	0	0	0	0	0	0	0	0	0	0	0
AB0224_C	0	0	0	0	0	0	0	0	0	0	0	0
AB0226_C	0	0	0	0	0	0	0	0	0	0	0	0
AB0227_C	0	0	0	0	0	0	0	0	0	0	0	0
AB0228_C	0	0	0	0	0	0	0	0	0	0	0	0
AB0229_C	0	0	0	0	0	0	0	2	0	0	0	0
AB0231_C	0	0	0	0	0	0	0	0	0	0	0	0
AB0232_C	0	0	0	0	0	0	0	0	0	0	0	0
AB0233_C	0	0	0	0	0	0	0	0	0	0	0	0
AB0234_C	0	0	0	0	0	0	0	0	0	0	0	0
AB0235_C	0	0	0	0	0	0	0	0	0	0	0	0
AB0236_C	0	0	0	0	0	0	0	0	0	0	0	0
AB0237_C	0	0	0	0	0	0	0	0	0	0	0	0
AB0238_C	0	0	0	0	0	0	0	0	0	0	0	0
AB0239_C	0	0	0	0	0	0	0	0	0	0	0	0
AB0240_C	0	0	0	0	0	0	0	0	0	0	0	0
AB0241_C	0	0	0	0	0	0	0	0	0	0	0	0
AB0242_C	0	0	0	0	0	0	0	0	0	0	0	0
AB0243_C	0	0	0	0	0	0	0	0	0	0	0	0
AB0244_C	0	0	0	0	0	0	0	0	0	0	0	0
AB0246_C	0	1	0	0	0	0	0	0	0	0	0	0
AB0247_C	0	0	0	0	0	0	0	0	0	0	0	0
AB0248_C	0	1	0	0	0	0	0	0	0	0	0	0
AB0249_C	0	0	0	0	0	0	0	0	0	0	0	0

	Dup 0	Dup 1	Dup 2	Dup 3	Dup 4	Dup 5	Dup 6	Dup 7	Dup 8	Dup 9	Dup 10	Dup 11
AB0250_C	0	1	0	0	0	0	0	0	0	0	0	0
AB0251_C	0	0	0	0	0	0	0	0	0	0	0	0
AB0252_C	0	0	0	0	0	0	0	0	0	0	0	0
AB0253_C	0	0	0	0	0	0	0	0	0	0	0	0
AB0255_C	0	0	0	0	0	0	0	0	0	0	0	0
AB0256_C	0	0	0	0	0	0	0	0	0	0	0	0
AB0257_C	0	0	0	0	0	0	0	0	0	0	0	0
AB0258_C	0	0	0	0	0	0	0	0	0	0	0	0
AB0260_C	0	0	0	0	0	0	0	0	0	0	0	0
AB0261_C	0	0	0	0	0	0	0	0	0	0	0	0
AB0262_C	0	0	0	0	0	0	0	0	0	0	0	0
AB0263_C	0	1	0	0	0	0	0	0	0	0	0	0
AB0264_C	0	0	0	0	0	0	0	0	0	0	0	0
AB0265_C	0	0	0	0	0	0	0	0	0	0	0	0
AB0266_C	0	0	0	0	0	0	0	1	0	0	0	0
AB0267_C	0	0	0	0	0	0	0	0	0	0	0	0
AB0268_C	0	0	0	0	0	0	0	0	0	0	0	0
AB0270_C	0	0	0	0	0	0	0	0	0	0	0	0
AB0271_C	0	0	0	0	0	0	0	0	0	0	0	0
AB0272_C	0	0	0	0	0	0	0	0	0	0	0	0
AB0273_C	0	0	0	0	0	0	0	0	0	0	0	0
AB0274_C	0	0	0	0	0	0	0	0	0	0	0	0
AB0275_C	0	0	0	0	0	0	0	0	0	0	0	0
AB0276_C	0	0	0	0	0	0	0	0	0	0	0	0
AB0277_C	0	0	0	0	0	0	0	0	0	0	0	0
AB0278_C	0	0	0	0	0	0	0	0	0	0	0	0
AB0279_C	0	1	0	0	0	0	0	0	0	0	0	0
AB0280_C	0	0	0	0	0	0	0	0	0	0	0	0
AB0281_C	0	0	0	0	0	0	0	0	0	0	0	0
AB0282_C	0	0	0	0	0	0	0	0	0	0	0	0
AB0283_C	0	0	0	0	0	0	0	0	0	0	0	0
AB0284_C	0	0	0	0	0	0	0	0	0	0	0	0
AC0089_C	0	0	1	1	0	0	0	0	0	0	0	0
AC0090_C	0	0	2	1	0	0	0	0	0	0	0	0
AC0091_C	0	0	0	0	0	0	0	0	3	0	0	0
AC0092_C	0	0	0	0	0	0	0	0	0	0	0	0
AC0093_C	0	0	0	0	0	0	0	0	0	0	0	0
AC0094_C	0	0	0	0	0	0	0	0	0	0	0	0
AC0095_C	0	0	0	0	0	0	0	0	0	0	0	0
AC0096_C	0	0	0	1	0	0	0	0	0	0	0	0
AC0097_C	0	0	0	0	0	0	0	0	0	0	0	0
AC0098_C	0	0	0	0	0	0	0	0	0	0	0	0
AC0099_C	0	0	0	0	0	0	0	0	0	0	0	0
AC0100_C	0	0	0	0	0	0	0	0	0	0	0	0
AC0101_C	0	0	0	0	0	0	0	0	0	0	0	0
AC0102_C	0	0	0	1	0	0	0	0	0	0	0	0
AC0103_C	0	0	0	0	0	0	0	0	0	0	0	0
AC0104_C	0	0	0	0	0	0	0	0	0	0	0	0
AC0105_C	0	0	0	0	0	0	0	0	0	0	0	0
AC0106_C	0	0	0	0	0	0	0	0	0	0	0	0
AC0107_C	0	0	0	0	0	0	0	0	0	0	0	0
AC0108_C	0	0	3	2	0	0	0	0	0	0	0	0
AC0109_C	0	0	0	0	0	0	0	0	0	0	0	0
AC0110_C	0	0	0	0	0	0	0	0	0	0	0	0
AC0111_C	0	0	0	0	0	0	0	0	0	0	0	0
AC0112_C	0	0	0	0	0	0	0	0	0	0	0	0
AC0113_C	0	0	0	0	0	0	0	0	0	0	0	0
AC0114_C	0	0	0	0	0	0	0	0	0	0	0	0
AC0115_C	0	0	0	0	0	0	0	0	0	0	0	0
AC0116_C	0	0	0	0	0	0	0	0	0	0	0	0
AC0117_C	0	0	0	0	0	0	0	0	0	0	0	0
AC0118_C	0	0	0	0	0	0	0	0	0	0	0	0
AC0119_C	0	0	0	0	0	0	0	0	0	0	0	0
AC0120_C	0	0	0	0	0	0	0	0	0	0	0	0
AC0121_C	0	0	0	0	0	0	0	0	0	0	0	0
AC0122_C	0	0	0	0	0	0	0	0	0	0	0	0
AC0123_C	0	0	0	0	0	0	0	0	0	0	0	0
AC0124_C	0	0	2	1	0	0	0	0	0	0	0	0

	Dup 0	Dup 1	Dup 2	Dup 3	Dup 4	Dup 5	Dup 6	Dup 7	Dup 8	Dup 9	Dup 10	Dup 11
AC0125_C	0	0	0	0	0	0	0	0	0	0	0	0
AC0126_C	0	0	0	0	0	0	0	0	0	0	0	0
AC0127_C	0	0	0	0	0	0	0	0	0	0	0	0
AC0128_C	0	0	0	0	0	0	0	0	0	0	0	0
AC0129_C	0	0	0	0	0	0	0	0	0	0	0	0
AC0130_C	0	0	0	0	0	0	0	0	0	0	0	0
AC0131_C	0	0	0	1	0	0	0	0	0	0	0	0
AC0132_C	0	0	0	0	0	0	0	0	0	0	0	0
AC0133_C	0	0	0	0	0	0	0	0	0	0	0	0
AC0134_C	0	0	0	0	0	0	0	0	0	0	0	0
AC0135_C	0	0	0	0	0	0	0	0	0	0	0	0
AC0136_C	0	0	0	0	0	0	0	0	0	0	0	0
AC0137_C	0	0	0	0	0	0	0	0	0	0	0	0
AC0138_C	0	0	0	1	0	0	0	0	0	0	0	0
AC0139_C	0	0	0	1	0	0	0	0	0	0	0	0
AC0140_C	0	0	0	0	0	0	0	0	0	0	0	0
AC0141_C	0	0	0	0	0	0	0	0	0	0	0	0
AC0142_C	0	0	0	0	0	0	0	0	0	0	0	0
AC0143_C	0	0	0	0	0	0	0	0	0	0	0	0
AC0144_C	0	0	0	0	0	0	0	0	0	0	0	0
AC0145_C	0	0	0	1	0	0	0	0	0	0	0	0
AC0146_C	0	0	0	0	0	0	0	0	0	0	0	0
AC0147_C	0	0	0	0	0	0	0	0	0	0	0	0
AC0148_C	0	0	2	1	0	0	0	0	0	0	0	0
AC0149_C	0	0	0	0	0	0	0	0	0	0	0	0
AC0150_C	0	0	0	0	0	0	0	0	0	0	0	0
AC0151_C	0	0	0	0	0	0	0	0	0	0	0	0
AC0152_C	0	0	0	0	0	0	0	0	0	0	0	0
AC0153_C	0	0	0	0	0	0	0	0	0	0	0	0
AC0154_C	0	0	0	0	0	0	0	0	0	0	0	0
AC0155_C	0	0	0	1	0	0	0	0	0	0	0	0
AC0156_C	0	0	1	1	0	0	0	0	0	0	0	0
AC0157_C	0	0	2	1	0	0	0	0	0	0	0	0
AC0158_C	0	0	0	0	0	0	0	0	0	0	0	0
AC0159_C	0	0	0	0	0	0	0	0	5	0	0	0
AC0160_C	0	0	1	1	0	0	0	0	0	0	0	0
AC0161_C	0	0	0	0	0	0	0	0	0	0	0	0
AC0162_C	0	0	0	1	0	0	0	0	0	0	0	0
AC0163_C	0	0	0	0	0	0	0	0	0	0	0	0
AC0164_C	0	0	0	0	0	0	0	0	0	0	0	0
AC0166_C	0	0	0	0	0	0	0	0	0	0	0	0
AC0167_C	0	0	3	1	0	0	0	0	0	0	0	0
AC0168_C	0	0	0	0	0	0	0	0	0	0	0	0
AC0169_C	0	0	0	0	0	0	0	0	0	0	0	0
AC0170_C	0	0	0	0	0	0	0	0	0	0	0	0
AC0171_C	0	0	0	0	0	0	0	0	0	0	0	0
AC0172_C	0	0	0	0	0	0	0	0	0	0	0	0
AC0173_C	0	0	0	0	0	0	0	0	4	0	0	0
AC0174_C	0	0	0	0	0	0	0	0	0	0	0	0
AC0176_C	0	0	0	0	0	0	0	0	0	0	0	0
AC0177_C	0	0	0	0	0	0	0	0	0	0	0	0
AC0178_C	0	0	0	0	0	0	0	0	0	0	0	0
AC0179_C	0	0	0	0	0	0	1	0	0	0	0	0
AC0180_C	0	0	0	0	0	0	0	0	0	0	0	0
AC0181_C	0	0	0	0	0	0	0	0	0	0	0	0
AC0182_C	0	0	0	0	0	0	0	0	0	0	0	0
AC0183_C	0	0	NA	1	0	0	0	0	NA	0	0	0
AC0184_C	0	0	0	0	0	0	0	0	0	0	0	0
AC0185_C	0	0	0	0	0	0	0	0	0	0	0	0
AC0186_C	0	0	0	0	0	0	0	0	0	0	0	0
AC0187_C	0	0	0	0	0	0	0	0	0	0	0	0
AC0188_C	0	0	3	0	0	0	0	0	0	0	0	0
AC0189_C	0	0	0	0	0	0	0	0	0	0	0	0
AC0190_C	0	0	0	0	0	0	0	0	0	0	0	0
AC0191_C	0	0	0	0	0	0	0	0	0	0	0	0
AC0192_C	0	0	0	0	0	0	0	0	0	0	0	0
AC0193_C	0	0	0	0	0	0	0	0	0	0	0	0
AC0194_C	0	0	0	0	0	0	0	0	0	0	0	0

	Dup 0	Dup 1	Dup 2	Dup 3	Dup 4	Dup 5	Dup 6	Dup 7	Dup 8	Dup 9	Dup 10	Dup 11
AC0195_C	0	0	0	0	0	0	0	0	0	0	0	0
AC0196_C	0	0	0	0	0	0	0	0	0	0	0	0
AC0197_C	0	0	0	0	0	0	0	0	0	0	0	0
AC0199_C	0	0	0	0	0	0	0	0	0	0	0	0
AC0200_C	0	0	1	1	0	0	0	0	0	0	0	0
AC0201_C	0	0	0	0	0	0	0	0	0	0	0	0
AC0202_C	0	0	0	0	0	0	0	0	0	0	0	0
AC0203_C	0	0	5	1	0	0	0	0	0	0	0	0
AG0082_C	0	0	0	0	0	0	0	0	0	0	0	0
AG0085_C	0	0	0	0	0	0	0	0	0	0	0	0
AG0089_C	0	0	0	0	0	0	0	0	0	0	0	0
AG0096_C	0	0	0	0	0	0	0	0	0	0	0	0
AG0097_C	0	0	0	0	0	0	0	0	0	0	0	0
AG0098_C	0	0	0	0	0	0	0	0	0	0	0	0
AG0100_C	0	0	0	0	0	0	0	0	0	0	0	0
AG0102_C	0	0	0	0	0	0	0	0	0	0	0	0
AG0104_C	0	0	0	0	0	0	0	0	0	0	0	0
AG0106_C	0	0	0	0	0	0	0	0	0	0	0	0
AG0108_C	0	0	0	0	0	0	0	0	0	0	0	0
AG0109_C	0	0	0	0	0	0	0	0	0	0	0	0
AG0111_C	0	0	0	0	0	0	0	0	0	0	0	0
AG0118_C	0	0	0	0	0	0	0	0	0	0	0	0
AG0120_C	0	0	0	0	0	0	0	0	0	0	0	0
AG0121_C	0	0	0	0	0	0	0	0	0	0	0	0
AG0123_C	0	0	0	0	0	0	0	0	0	0	0	0
AG0125_C	0	0	0	0	0	0	0	0	0	0	0	0
AG0126_C	0	0	0	0	0	0	0	0	0	0	0	0
AG0127_C	0	0	0	0	0	0	0	0	0	0	0	0
AG0128_C	0	0	0	0	0	0	0	0	0	0	0	0
AG0129_C	0	0	0	0	0	0	0	0	0	0	0	0
AG0133_C	0	0	0	0	0	0	0	0	0	0	0	0
AG0134_C	0	0	0	0	0	0	0	0	0	0	0	0
AG0136_C	0	0	0	0	0	0	0	0	0	0	0	0
AG0137_C	0	0	0	0	0	0	0	0	0	0	0	0
AG0138_C	0	0	0	0	0	0	0	0	0	0	0	0
AG0139_C	0	0	0	0	0	0	0	0	0	0	0	0
AG0141_C	0	0	0	0	0	0	0	0	0	0	0	0
AG0142_C	0	0	0	0	0	0	0	0	0	0	0	0
AG0143_C	0	0	0	0	0	0	0	0	0	0	0	0
AG0144_C	0	0	0	0	0	0	0	0	0	0	0	0
AG0145_C	0	0	0	0	0	0	0	0	0	0	0	0
AG0146_C	0	0	0	0	0	0	0	0	0	0	0	0
AG0147_C	0	0	0	0	0	0	0	0	0	0	0	0
AG0148_C	0	0	0	0	0	0	0	0	0	0	0	0
AG0152_C	0	0	0	0	0	0	0	0	0	0	0	0
AG0153_C	0	0	0	0	0	0	0	0	0	0	0	0
AG0156_C	0	0	0	0	0	0	0	0	0	0	0	0
AG0159_C	0	0	0	0	0	0	0	0	0	0	0	0
AG0162_C	0	0	0	0	0	0	0	0	0	0	0	0
AG0163_C	0	0	0	0	0	0	0	0	0	0	0	0
AG0169_C	0	0	0	0	0	0	0	0	0	0	0	0
AG0170_C	0	0	0	0	0	0	0	0	0	0	0	0
AG0172_C	0	0	0	0	0	0	0	0	0	0	0	0
AG0178_C	0	0	0	0	0	0	0	0	0	0	0	0
AG0179_C	0	0	0	0	0	0	0	0	0	0	0	0
AG0181_C	0	0	0	0	0	0	0	0	0	0	0	0
AG0183_C	0	0	0	0	0	0	0	0	0	0	0	0
AG0195_C	0	0	0	0	0	0	0	0	0	0	0	0
AG0197_C	0	0	0	0	0	0	0	0	0	0	0	0
AG0202_C	0	0	0	0	0	0	0	0	0	0	0	0
AG0203_C	0	0	0	0	0	0	0	0	0	0	0	0
AG0204_C	0	0	0	0	0	0	0	0	0	0	0	0
AG0206_C	0	0	0	0	0	0	0	0	0	0	0	0
AG0208_C	0	0	0	0	0	0	0	0	0	0	0	0
AG0214_C	0	0	0	0	0	0	0	0	0	0	0	0
AG0221_C	0	0	0	0	0	0	0	0	0	0	0	0
AG0223_C	0	0	0	0	0	0	0	0	0	0	0	0
AG0227_C	0	0	0	0	0	0	0	0	0	0	0	0

	Dup 0	Dup 1	Dup 2	Dup 3	Dup 4	Dup 5	Dup 6	Dup 7	Dup 8	Dup 9	Dup 10	Dup 11
AG0229_C	0	0	0	0	0	0	0	0	0	0	0	0
AG0230_C	0	0	0	0	0	0	0	0	0	0	0	0
AG0231_C	0	0	0	0	0	0	0	0	0	0	0	0
AG0232_C	0	0	0	0	0	0	0	0	0	0	0	0
AG0263_C	0	0	0	0	0	0	0	0	0	0	0	0
AJ0023_C	0	0	0	0	0	0	0	0	0	0	0	0
AJ0024_C	0	0	0	0	0	0	0	0	0	0	0	0
AJ0028_C	0	0	0	0	0	0	0	0	0	0	0	0
AJ0032_C	0	0	0	0	0	0	0	0	0	0	0	0
AJ0035_C	0	0	0	0	0	0	0	0	0	0	0	0
AJ0036_C	0	0	0	0	0	0	0	0	0	0	0	0
AJ0037_C	0	0	0	0	0	0	0	0	0	0	0	0
AJ0038_C	0	0	0	0	0	0	0	0	0	0	0	0
AJ0039_C	0	0	0	0	0	0	0	0	0	0	0	0
AJ0043_C	0	0	0	0	0	0	0	0	0	0	0	0
AJ0044_C	0	0	0	0	0	0	0	0	0	0	0	0
AJ0045_C	0	0	0	0	0	0	0	0	0	0	0	0
AJ0047_C	0	0	0	0	0	0	0	0	0	0	0	0
AJ0051_C	0	0	0	0	0	0	0	0	0	0	0	0
AJ0052_C	0	0	0	0	0	0	0	0	0	0	0	0
AJ0056_C	0	0	0	0	0	0	0	0	0	0	0	0
AJ0059_C	0	0	0	0	0	0	0	0	0	0	0	0
AJ0060_C	0	0	0	0	0	0	0	0	0	0	0	0
AJ0061_C	0	0	0	0	0	0	0	0	0	0	0	0
AJ0063_C	0	0	0	0	0	0	0	0	0	0	0	0
AJ0064_C	0	0	0	0	0	0	0	0	0	0	0	0
AJ0066_C	0	0	0	0	0	0	0	0	0	0	0	0
AJ0068_C	0	0	0	0	0	0	0	0	0	0	0	0
AJ0070_C	0	0	0	0	0	0	0	0	0	0	0	0
AJ0071_C	0	0	0	0	0	0	0	0	0	0	0	0
AJ0072_C	0	0	0	0	0	0	0	0	0	0	0	0
AJ0074_C	0	0	0	0	0	0	0	0	0	0	0	0
AJ0075_C	0	0	0	0	0	0	0	0	0	0	0	0
AJ0076_C	0	0	0	0	0	0	0	0	0	0	0	0
AJ0077_C	0	0	0	0	0	0	0	0	0	0	0	0
AJ0078_C	0	0	0	0	0	0	0	0	0	0	0	0
AJ0080_C	0	0	0	0	0	0	0	0	0	0	0	0
AJ0081_C	0	0	0	0	0	0	0	0	0	0	0	0
AJ0084_C	0	0	0	0	0	0	0	0	0	0	0	0
AJ0085_C	0	0	0	0	0	0	0	0	0	0	0	0
AJ0086_C	0	0	0	0	0	0	0	0	0	0	0	0
AJ0087_C	0	0	0	0	0	0	0	0	0	0	0	0
AJ0088_C	0	0	0	0	0	0	0	0	0	0	0	0
AJ0090_C	0	0	0	0	0	0	0	0	0	0	0	0
AJ0092_C	0	0	0	0	0	0	0	0	0	0	0	0
AJ0093_C	0	0	0	0	0	0	0	0	0	0	0	0
AJ0095_C	0	0	0	0	0	0	0	0	0	0	0	0
AJ0096_C	0	0	0	0	0	0	0	0	0	0	0	0
AJ0097_C	0	0	0	0	0	0	0	0	0	0	0	0
AJ0098_C	0	0	0	0	0	0	0	0	0	0	0	0
AJ0099_C	0	0	0	0	0	0	0	0	0	0	0	0
AJ0100_C	0	0	0	0	0	0	0	0	0	0	0	0
AJ0101_C	0	0	0	0	0	0	0	0	0	0	0	0
AJ0102_C	0	0	0	0	0	0	0	0	0	0	0	0
AJ0103_C	0	0	0	0	0	0	0	0	0	0	0	0
AJ0105_C	0	0	0	0	0	0	0	0	0	0	0	0
AJ0107_C	0	0	0	0	0	0	0	0	0	0	0	0
AJ0109_C	0	0	0	0	0	0	0	0	0	0	0	0
AJ0113_C	0	0	0	0	0	0	0	0	0	0	0	0
AJ0115_C	0	0	0	0	0	0	0	0	0	0	0	0
AJ0116_C	0	0	0	0	0	0	0	0	0	0	0	0
AJ0117_C	0	0	0	0	0	0	0	0	0	0	0	0
AJ0119_C	0	0	0	0	0	0	0	0	0	0	0	0
AJ0128_C	0	0	0	0	0	0	0	0	0	0	0	0
AJ0129_C	0	0	0	0	0	0	0	0	0	0	0	0
AJ0130_C	0	0	0	0	0	0	0	0	0	0	0	0
AJ0131_C	0	0	0	0	0	0	0	0	0	0	0	0
AJ0132_C	0	0	0	0	0	0	0	0	0	0	0	0

	Dup 0	Dup 1	Dup 2	Dup 3	Dup 4	Dup 5	Dup 6	Dup 7	Dup 8	Dup 9	Dup 10	Dup 11
AJ0133_C	0	0	0	0	0	0	0	0	0	0	0	0
AJ0134_C	0	0	0	0	0	0	0	0	0	0	0	0
AJ0135_C	0	0	0	0	0	0	0	0	0	0	0	0
AJ0136_C	0	0	0	0	0	0	0	0	0	0	0	0
AJ0137_C	0	0	0	0	0	0	0	0	0	0	0	0
AJ0138_C	0	0	0	0	0	0	0	0	0	0	0	0
AJ0139_C	0	0	0	0	0	0	0	0	0	0	0	0
AJ0140_C	0	0	0	0	0	0	0	0	0	0	0	0
AJ0141_C	0	0	0	0	0	0	0	0	0	0	0	0
AJ0142_C	0	0	0	0	0	0	0	0	0	0	0	0
AJ0143_C	0	0	0	0	0	0	0	0	0	0	0	0
AJ0144_C	0	0	0	0	0	0	0	0	0	0	0	0
AJ0145_C	0	0	0	0	0	0	0	0	0	0	0	0
AJ0146_C	0	0	0	0	0	0	0	0	0	0	0	0
AJ0147_C	0	0	0	0	0	0	0	0	0	0	0	0
AJ0148_C	0	0	0	0	0	0	0	0	0	0	0	0
AJ0149_C	0	0	0	0	0	0	0	0	0	0	0	0
AJ0150_C	0	0	0	0	0	0	0	0	0	0	0	0
AJ0151_C	0	0	0	0	0	0	0	0	0	0	0	0
AJ0152_C	0	0	0	0	0	0	0	0	0	0	0	0
AJ0153_C	0	0	0	0	0	0	0	0	0	0	0	0
AJ0154_C	0	0	0	0	0	0	0	0	0	0	0	0
AJ0155_C	0	0	0	0	0	0	0	0	0	0	0	0
AJ0156_C	0	0	0	0	0	0	0	0	0	0	0	0
AJ0157_C	0	0	0	0	0	0	0	0	0	0	0	0
AJ0158_C	0	0	0	0	0	0	0	0	0	0	0	0
AJ0159_C	0	0	0	0	0	0	0	0	0	0	0	0
AJ0161_C	0	0	0	0	0	0	0	0	0	0	0	0
AK0060_C	0	0	0	0	0	0	0	0	0	0	0	0
AK0062_C	0	0	0	0	0	0	0	0	0	0	0	NA
AK0065_C	0	0	0	0	0	0	0	0	0	0	0	0
AK0066_C	0	0	0	0	0	0	0	0	0	0	0	0
AK0067_C	0	0	0	0	0	0	0	0	0	0	0	0
AK0068_C	0	0	0	0	0	0	0	0	0	0	0	0
AK0069_C	0	0	0	0	0	0	0	0	0	0	0	0
AK0070_C	0	0	0	0	0	0	0	0	0	0	0	0
AK0072_C	0	0	0	0	0	0	0	0	0	0	0	0
AK0073_C	0	0	0	0	0	0	0	0	0	0	0	1
AK0074_C	0	0	0	0	0	0	0	0	0	0	0	1
AK0075_C	0	0	0	0	0	0	0	0	0	0	0	0
AK0076_C	0	0	0	0	0	0	0	0	0	0	0	1
AK0077_C	0	0	0	0	0	0	0	0	0	0	0	0
AK0078_C	0	0	0	0	0	0	0	0	0	0	0	0
AK0079_C	0	0	0	0	0	0	0	0	0	0	0	1
AK0080_C	0	0	0	0	0	0	0	0	0	0	0	1
AK0081_C	0	0	0	0	0	0	0	0	0	0	0	1
AK0082_C	0	0	0	0	0	0	0	0	0	0	0	0
AK0085_C	0	0	0	0	0	0	0	0	0	0	0	0
AK0086_C	0	0	0	0	0	0	0	0	0	0	0	0
AK0087_C	0	0	0	0	0	0	0	0	0	0	0	NA
AK0088_C	0	0	0	0	0	0	0	0	0	0	0	0
AK0089_C	0	0	0	0	0	0	0	0	0	0	0	NA
AK0090_C	0	0	0	0	0	0	0	0	0	0	0	NA
AK0091_C	0	0	0	0	0	0	0	0	0	0	0	NA
AK0092_C	0	0	0	0	0	0	0	0	0	0	0	0
AK0093_C	0	0	0	0	0	0	0	0	0	0	0	NA
AK0094_C	0	0	0	0	0	0	0	0	0	0	0	0
AK0095_C	0	0	0	0	0	0	0	0	0	0	0	NA
AK0096_C	0	0	0	0	0	0	0	0	0	0	0	1
AK0098_C	0	0	0	0	0	0	0	0	0	0	0	0
AK0099_C	0	0	0	0	0	0	0	0	0	0	0	0
AK0100_C	0	0	0	0	0	0	0	0	0	0	0	2
AK0101_C	0	0	0	0	0	0	0	0	0	0	0	1
AK0102_C	0	0	0	0	0	0	0	0	0	0	0	0
AK0103_C	0	0	0	0	0	0	0	0	0	0	0	1
AK0104_C	0	0	0	0	0	0	0	0	0	0	0	1
AK0105_C	0	0	0	0	0	0	0	0	0	0	0	0
AK0106_C	0	0	0	0	0	0	0	0	0	0	0	0

	Dup 0	Dup 1	Dup 2	Dup 3	Dup 4	Dup 5	Dup 6	Dup 7	Dup 8	Dup 9	Dup 10	Dup 11
AK0107_C	0	0	0	0	0	0	0	0	0	0	0	1
AK0108_C	0	0	0	0	0	0	0	0	0	0	0	1
AK0109_C	0	0	0	0	0	0	0	0	0	0	0	0
AK0110_C	0	0	0	0	0	0	0	0	0	0	0	1
AK0116_C	0	0	0	0	0	0	0	0	0	0	0	0
AK0117_C	0	0	0	0	0	0	0	0	0	0	0	NA
AK0119_C	0	0	0	0	0	0	0	0	0	0	0	0
AK0127_C	0	0	0	0	0	0	0	0	0	0	0	NA
AN0007_C	0	0	0	0	0	0	0	0	0	0	0	0
AN0008_C	0	0	0	0	0	0	0	0	0	0	0	0
AN0009_C	0	0	0	0	0	0	0	0	0	0	0	0
AN0010_C	0	0	0	0	0	0	0	0	0	0	0	0
AN0011_C	0	0	0	0	0	0	0	0	0	0	0	0
AN0012_C	0	0	0	0	0	0	0	0	0	0	0	0
AN0013_C	0	0	0	0	0	0	0	0	0	0	0	0
AN0014_C	0	0	0	0	1	0	0	0	0	0	0	0
AN0015_C	0	0	0	0	0	0	0	0	0	0	0	0
AN0016_C	0	0	0	0	0	0	0	0	0	0	0	0
AN0017_C	0	0	0	0	0	0	0	0	0	0	0	0
AN0018_C	0	0	0	0	0	0	0	0	0	0	0	0
AN0019_C	0	0	0	0	0	0	0	0	0	0	0	0
AN0020_C	0	0	0	0	0	0	0	0	0	0	0	0
AN0022_C	0	0	0	0	0	0	0	0	0	0	0	0
AN0023_C	0	0	0	0	0	0	0	0	0	0	0	0
AN0024_C	0	0	0	0	0	0	0	0	0	0	0	0
AN0025_C	0	0	0	0	0	0	0	0	0	0	0	0
AN0026_C	0	0	0	0	0	0	0	0	0	0	0	0
AN0027_C	0	0	0	0	0	0	0	0	0	0	0	0
AN0028_C	0	0	0	0	0	0	0	0	0	0	0	0
AN0029_C	0	0	0	0	0	0	0	0	0	0	0	0
AN0030_C	0	0	0	0	0	1	0	0	0	0	0	0
AN0031_C	0	0	0	0	0	0	0	0	0	0	0	0
AN0032_C	0	0	0	0	0	0	0	0	0	0	0	0
AN0033_C	0	0	0	0	0	0	0	0	0	0	0	0
AN0034_C	0	0	0	0	0	0	0	0	0	0	0	0
AN0035_C	0	0	0	0	0	0	0	0	0	0	0	0
AN0036_C	0	0	0	0	0	0	0	0	0	0	0	0
AN0037_C	0	0	0	0	0	0	0	0	0	0	0	0
AN0038_C	0	0	0	0	0	0	0	0	0	0	0	0
AN0039_C	0	0	0	0	0	0	0	0	0	0	0	0
AN0040_C	0	0	0	0	0	0	0	0	0	0	0	0
AN0041_C	0	0	0	0	0	0	0	0	0	0	0	0
AN0042_C	0	0	0	0	0	0	0	0	0	0	0	0
AN0043_C	0	0	0	0	0	0	0	0	0	0	0	0
AN0044_C	0	0	0	0	0	0	0	0	0	0	0	0
AN0045_C	0	0	0	0	0	0	0	0	0	0	0	0
AN0046_C	0	0	0	0	0	0	0	0	0	0	0	0
AN0047_C	0	0	0	0	0	0	0	0	0	0	1	0
AN0048_C	0	0	0	0	0	0	0	0	0	0	0	0
AN0049_C	0	0	0	0	0	0	0	0	0	0	0	0
AN0050_C	0	0	0	0	0	0	0	0	0	0	0	0
AN0051_C	0	0	0	0	0	0	0	0	0	0	0	0
AN0053_C	0	0	0	0	0	0	0	0	0	0	0	0
AN0054_C	0	0	0	0	0	0	0	0	0	0	0	0
AN0055_C	0	0	0	0	0	0	0	0	0	0	0	0
AN0056_C	0	0	0	0	0	0	0	0	0	0	0	0
AN0057_C	0	0	0	0	0	0	0	0	0	0	0	0
AN0058_C	0	0	0	0	0	0	0	0	0	0	0	0
AN0059_C	0	0	0	0	0	0	0	0	0	0	0	0
AN0060_C	0	0	0	0	0	0	0	0	0	0	0	0
AN0061_C	0	0	0	0	0	1	0	0	0	0	0	0
AN0062_C	0	0	0	0	0	0	0	0	0	0	0	0
AN0063_C	0	0	0	0	0	0	0	0	0	0	0	0
AN0064_C	0	0	0	0	1	0	0	0	0	0	0	0
AN0065_C	0	0	0	0	0	0	0	0	0	0	0	0
AN0066_C	0	0	0	0	0	0	0	0	0	0	0	0
AN0067_C	0	0	0	0	0	0	0	0	0	0	0	0
AN0068_C	0	0	0	0	0	0	0	0	0	0	0	0

	Dup 0	Dup 1	Dup 2	Dup 3	Dup 4	Dup 5	Dup 6	Dup 7	Dup 8	Dup 9	Dup 10	Dup 11
AN0069_C	0	0	0	0	0	0	0	0	0	0	0	0
AN0070_C	0	0	0	0	0	0	0	0	0	0	0	0
AN0071_C	0	0	0	0	0	0	0	0	0	0	0	0
AN0072_C	0	0	0	0	0	0	0	0	0	0	0	0
AN0073_C	0	0	0	0	1	0	0	0	0	0	0	0
AN0074_C	0	0	0	0	0	0	0	0	0	0	0	0
AN0075_C	0	0	0	0	0	0	0	0	0	0	0	0
AN0076_C	0	0	0	0	0	0	0	0	0	0	0	0
AN0077_C	0	0	0	0	0	0	0	0	0	0	0	0
AN0079_C	0	0	0	0	0	0	0	0	0	0	0	0
AN0080_C	0	0	0	0	0	0	0	0	0	0	0	0
AN0081_C	0	0	0	0	0	0	0	0	0	0	0	0
AN0082_C	0	0	0	0	0	0	0	0	0	0	0	0
AN0083_C	0	0	0	0	0	0	0	0	0	0	0	0
AN0084_C	0	0	0	0	0	0	0	0	0	0	0	0
AN0085_C	0	0	0	0	0	0	0	0	0	0	0	0
AN0086_C	0	0	0	0	0	0	0	0	0	0	0	0
AN0087_C	0	0	0	0	0	0	0	0	0	0	0	0
AN0088_C	0	0	0	0	0	0	0	0	0	0	0	0
AN0089_C	0	0	0	0	0	0	0	0	0	0	0	0
AN0090_C	0	0	0	0	0	0	0	0	0	0	0	0
AN0091_C	0	0	0	0	0	0	0	0	0	0	0	0
AN0092_C	0	0	0	0	0	0	0	0	0	0	0	0
AN0093_C	0	0	0	0	0	0	0	0	0	0	0	0
AN0094_C	0	0	0	0	0	0	0	0	0	0	0	0
AN0095_C	0	0	0	0	0	0	0	0	0	0	0	0
AN0096_C	0	0	0	0	0	0	0	0	0	0	0	0
AN0097_C	0	0	0	0	0	0	0	0	0	0	0	0
AN0098_C	0	0	0	0	0	0	0	0	0	0	0	0
AN0099_C	0	0	0	0	0	0	0	0	0	0	1	0
AN0100_C	0	0	0	0	0	0	0	0	0	0	0	0
AN0101_C	0	0	0	0	0	0	0	0	0	0	0	0
AN0102_C	0	0	0	0	0	0	0	0	0	0	0	0
AN0103_C	0	0	0	0	0	0	0	0	0	0	0	0
AN0104_C	0	0	0	0	0	0	0	0	0	0	0	0
AN0105_C	0	0	0	0	0	0	0	0	0	0	0	0
AN0106_C	0	0	0	0	0	0	0	0	0	0	0	0
AN0107_C	0	0	0	0	0	0	0	0	0	0	0	0
AN0108_C	0	0	0	0	0	0	0	0	0	0	0	0
AN0109_C	0	0	0	0	0	0	0	0	0	0	0	0
AN0111_C	0	0	0	0	0	0	0	0	0	0	0	0
AN0112_C	0	0	0	0	0	0	0	0	0	0	0	0
AN0113_C	0	0	0	0	0	0	0	0	0	0	0	0
AN0114_C	0	0	0	0	0	0	0	0	0	0	0	0
AN0115_C	0	0	0	0	1	0	0	0	0	0	0	0
AN0117_C	0	0	0	0	0	0	0	0	0	0	0	0
AN0118_C	0	0	0	0	0	0	0	0	0	0	0	0
AN0119_C	0	0	0	0	0	0	0	0	0	0	0	0
AN0120_C	0	0	0	0	0	0	0	0	0	0	0	0
AN0121_C	0	0	0	0	0	0	0	0	0	0	0	0
AN0122_C	0	0	0	0	0	0	0	0	0	0	0	0
AN0123_C	0	0	0	0	0	0	0	0	0	0	0	0
AN0124_C	0	0	0	0	0	0	0	0	0	0	0	0
AN0125_C	0	0	0	0	0	0	0	0	0	0	0	0
AN0126_C	0	0	0	0	0	0	0	0	0	0	0	0
AN0127_C	0	0	0	0	0	0	0	0	0	0	0	0
AN0128_C	0	0	0	0	0	0	0	0	0	0	0	0
AN0129_C	0	0	0	0	0	0	0	0	0	0	0	0
AN0130_C	0	0	0	0	0	0	0	0	0	0	0	0
AN0131_C	0	0	0	0	0	0	0	0	0	0	0	0
AN0132_C	0	0	0	0	0	0	0	0	0	0	0	0
AN0134_C	0	0	0	0	0	1	0	0	0	0	0	0
AN0135_C	0	0	0	0	0	0	0	0	0	0	0	0
AN0136_C	0	0	0	0	0	0	0	0	0	0	0	0
AN0137_C	0	0	0	0	0	0	0	0	0	0	0	0
AN0138_C	0	0	0	0	0	0	0	0	0	0	0	0
AN0139_C	0	0	0	0	0	0	0	0	0	0	0	0
AN0140_C	0	0	0	0	0	0	0	0	0	0	0	0

	Dup 0	Dup 1	Dup 2	Dup 3	Dup 4	Dup 5	Dup 6	Dup 7	Dup 8	Dup 9	Dup 10	Dup 11
AN0141_C	0	0	0	0	0	0	0	0	0	0	0	0
AN0142_C	0	0	0	0	0	0	0	0	0	0	0	0
AN0143_C	0	0	0	0	0	0	0	0	0	0	0	0
AN0144_C	0	0	0	0	0	0	0	0	0	0	0	0
AN0147_C	0	0	0	0	0	0	0	0	0	0	0	0
AN0149_C	0	0	0	0	0	0	0	0	0	0	0	0
AN0151_C	0	0	0	0	0	0	0	0	0	0	0	0
AN0152_C	0	0	0	0	0	0	0	0	0	0	0	0
AN0153_C	0	0	0	0	0	0	0	0	0	0	0	0
AN0154_C	0	0	0	0	0	0	0	0	0	0	0	0
AN0155_C	0	0	0	0	0	0	0	0	0	0	0	0
AN0156_C	0	0	0	0	0	0	0	0	0	0	0	0
AN0157_C	0	0	0	0	0	0	0	0	0	0	0	0
AN0158_C	0	0	0	0	0	0	0	0	0	0	0	0
AN0159_C	0	0	0	0	0	0	0	0	0	0	0	0
AN0160_C	0	0	0	0	0	0	0	0	0	0	0	0
AN0162_C	0	0	0	0	0	0	0	0	0	0	0	0
AN0163_C	0	0	0	0	0	0	0	0	0	0	0	0
AN0164_C	0	0	0	0	0	0	0	0	0	0	0	0
AN0165_C	0	0	0	0	0	0	0	0	0	0	0	0
AN0166_C	0	0	0	0	0	0	0	0	0	0	0	0
AN0167_C	0	0	0	0	0	0	0	0	0	0	0	0
AN0168_C	0	0	0	0	0	0	0	0	0	0	0	0
AN0169_C	0	0	0	0	0	0	0	0	0	0	0	0
AN0170_C	0	0	0	0	0	0	0	0	0	0	0	0
AN0171_C	0	0	0	0	0	0	0	0	0	0	0	0
AN0172_C	0	0	0	0	0	0	0	0	0	0	0	0
AN0173_C	0	0	0	0	0	0	0	0	0	0	0	0
AN0174_C	0	0	0	0	0	0	0	0	0	0	0	0
AN0175_C	0	0	0	0	0	0	0	0	0	0	0	0
AN0176_C	0	0	0	0	0	0	0	0	0	0	0	0
AN0177_C	0	0	0	0	0	0	0	0	0	0	0	0
AN0178_C	0	0	0	0	0	0	0	0	0	0	0	0
AN0179_C	0	0	0	0	0	0	0	0	0	0	0	0
AN0180_C	0	0	0	0	0	0	0	0	0	0	0	0
AN0181_C	0	0	0	0	0	0	0	0	0	0	0	0
AN0182_C	0	0	0	0	0	0	0	0	0	0	0	0
AN0183_C	0	0	0	0	0	0	0	0	0	0	0	0
AN0184_C	0	0	0	0	0	0	0	0	0	0	0	0
AN0185_C	0	0	0	0	0	0	0	0	0	0	0	0
AN0186_C	0	0	0	0	0	0	0	0	0	0	0	0
AN0187_C	0	0	0	0	0	0	0	0	0	0	0	0
AN0188_C	0	0	0	0	0	0	0	0	0	0	0	0
AN0189_C	0	0	0	0	0	0	0	0	0	0	0	0
AN0190_C	0	0	0	0	0	0	0	0	0	0	0	0
AN0191_C	0	0	0	0	0	0	0	0	0	0	0	0
AN0192_C	0	0	0	0	0	0	0	0	0	0	0	0
AN0193_C	0	0	0	0	0	0	0	0	0	0	0	0
AN0194_C	0	0	0	0	0	0	0	0	0	0	0	0
AN0196_C	0	0	0	0	0	0	0	0	0	0	0	0
AN0197_C	0	0	0	0	0	0	0	0	0	0	0	0
AN0198_C	0	0	0	0	0	0	0	0	0	0	0	0
AN0199_C	0	0	0	0	0	0	0	0	0	0	0	0
AN0200_C	0	0	0	0	0	0	0	0	0	0	0	0
AN0201_C	0	0	0	0	0	0	0	0	0	0	0	0
AN0202_C	0	0	0	0	0	0	0	0	0	0	0	0
AN0203_C	0	0	0	0	0	0	0	0	0	0	0	0
AN0204_C	0	0	0	0	0	0	0	0	0	0	0	0
AN0205_C	0	0	0	0	0	0	0	0	0	0	0	0
AN0206_C	0	0	0	0	0	0	0	0	0	0	0	0
AN0207_C	0	0	0	0	0	0	0	0	0	0	0	0
AN0208_C	0	0	0	0	0	0	0	0	0	0	0	0
AN0209_C	0	0	0	0	0	0	0	0	0	0	0	0
AN0210_C	0	0	0	0	0	0	0	0	0	0	0	0
AN0212_C	0	0	0	0	0	0	0	0	0	0	0	0
AN0213_C	0	0	0	0	0	0	0	0	0	0	0	0
AN0214_C	0	0	0	0	0	0	0	0	0	0	0	0
AN0215_C	0	0	0	0	0	0	0	0	0	0	0	0

	Dup 0	Dup 1	Dup 2	Dup 3	Dup 4	Dup 5	Dup 6	Dup 7	Dup 8	Dup 9	Dup 10	Dup 11
AN0217_C	0	0	0	0	0	0	0	0	0	0	0	0
AN0218_C	0	0	0	0	0	0	0	0	0	0	0	0
AN0219_C	0	0	0	0	0	0	0	0	0	0	0	0
AN0220_C	0	0	0	0	0	0	0	0	0	0	0	0
AN0221_C	0	0	0	0	0	0	0	0	0	0	0	0
AN0222_C	0	0	0	0	0	0	0	0	0	0	0	0
AN0223_C	0	0	0	0	0	0	0	0	0	0	0	0
AN0224_C	0	0	0	0	0	0	0	0	0	0	0	0
AN0225_C	0	0	0	0	0	0	0	0	0	0	0	0
AN0226_C	0	0	0	0	0	0	0	0	0	0	0	0
AN0227_C	0	0	0	0	0	0	0	0	0	0	0	0
AN0228_C	0	0	0	0	0	0	0	0	0	0	0	0
AN0229_C	0	0	0	0	0	0	0	0	0	0	0	0
AN0230_C	0	0	0	0	0	0	0	0	0	0	0	0
AN0231_C	0	0	0	0	0	0	0	0	0	0	0	0
AN0232_C	0	0	0	0	0	0	0	0	0	0	0	0
AN0233_C	0	0	0	0	0	0	0	0	0	0	0	0
AN0234_C	0	0	0	0	0	0	0	0	0	0	0	0
AN0235_C	0	0	0	0	0	0	0	0	0	0	0	0
AN0236_C	0	0	0	0	0	0	0	0	0	0	0	0
AN0237_C	0	0	0	0	0	0	0	0	0	0	0	0
AN0238_C	0	0	0	0	0	0	0	0	0	0	0	0
AN0239_C	0	0	0	0	0	0	0	0	0	0	0	0
AN0240_C	0	0	0	0	0	0	0	0	0	0	0	0
AN0241_C	0	0	0	0	0	0	0	0	0	0	0	0
AN0242_C	0	0	0	0	0	0	0	0	0	0	0	0
AN0243_C	0	0	0	0	0	0	0	0	0	0	0	0
AN0244_C	0	0	0	0	0	0	0	0	0	0	0	0
AN0245_C	0	0	0	0	0	0	0	0	0	0	0	0
AN0246_C	0	0	0	0	0	0	0	0	0	0	0	0
AN0247_C	0	0	0	0	0	0	0	0	0	0	0	0
AN0248_C	0	0	0	0	0	0	0	0	0	0	0	0
AN0250_C	0	0	0	0	0	0	0	0	0	0	0	0
AN0251_C	0	0	0	0	0	0	0	0	0	0	0	0
AN0252_C	0	0	0	0	0	1	0	0	0	0	0	0
AN0253_C	0	0	0	0	0	0	0	0	0	0	0	0
AN0254_C	0	0	0	0	0	0	0	0	0	0	0	0
AN0255_C	0	0	0	0	0	0	0	0	0	0	0	0
AN0256_C	0	0	0	0	0	0	0	0	0	0	0	0
AN0258_C	0	0	0	0	0	0	0	0	0	0	0	0
AN0259_C	0	0	0	0	0	0	0	0	0	0	0	0
AN0260_C	0	0	0	0	0	0	0	0	0	0	0	0
AN0261_C	0	0	0	0	0	0	0	0	0	0	0	0
AN0262_C	0	0	0	0	0	0	0	0	0	0	0	0
AN0263_C	0	0	0	0	0	0	0	0	0	0	0	0
AN0264_C	0	0	0	0	0	0	0	0	0	0	0	0
AN0265_C	0	0	0	0	0	0	0	0	0	0	0	0
AN0266_C	0	0	0	0	0	0	0	0	0	0	0	0
AN0267_C	0	0	0	0	0	0	0	0	0	0	0	0
AN0268_C	0	0	0	0	0	0	0	0	0	0	0	0
AN0269_C	0	0	0	0	0	0	0	0	0	0	0	0
AN0270_C	0	0	0	0	0	0	0	0	0	0	0	0
AN0271_C	0	0	0	0	0	0	0	0	0	0	0	0
AN0272_C	0	0	0	0	0	0	0	0	0	0	0	0
AN0275_C	0	0	0	0	0	0	0	0	0	0	0	0
AN0276_C	0	0	0	0	0	0	0	0	0	0	0	0
AN0277_C	0	0	0	0	0	0	0	0	0	0	0	0
AN0278_C	0	0	0	0	0	0	0	0	0	0	0	0
AN0279_C	0	0	0	0	0	0	0	0	0	0	0	0
AN0280_C	0	0	0	0	0	0	0	0	0	0	0	0
AN0281_C	0	0	0	0	0	0	0	0	0	0	0	0
AN0282_C	0	0	0	0	0	0	0	0	0	0	0	0
AN0283_C	0	0	0	0	0	0	0	0	0	0	0	0
AN0284_C	0	0	0	0	0	0	0	0	0	0	0	0
AN0285_C	0	0	0	0	0	0	0	0	0	0	0	0
AN0286_C	0	0	0	0	0	0	0	0	0	0	0	0
AN0287_C	0	0	0	0	0	0	0	0	0	0	0	0
AN0288_C	0	0	0	0	0	0	0	0	0	0	0	0

	Dup 0	Dup 1	Dup 2	Dup 3	Dup 4	Dup 5	Dup 6	Dup 7	Dup 8	Dup 9	Dup 10	Dup 11
AN0289_C	0	0	0	0	0	0	0	0	0	0	0	0
AN0290_C	0	0	0	0	0	0	0	0	0	0	0	0
AN0291_C	0	0	0	0	0	0	0	0	0	0	0	0
AN0292_C	0	0	0	0	0	0	0	0	0	0	0	0
AN0293_C	0	0	0	0	0	0	0	0	0	0	0	0
AN0294_C	0	0	0	0	0	0	0	0	0	0	0	0
AN0295_C	0	0	0	0	0	0	0	0	0	0	0	0
AN0296_C	0	0	0	0	0	0	0	0	0	0	0	0
AN0297_C	0	0	0	0	0	0	0	0	0	0	0	0
AN0298_C	0	0	0	0	0	0	0	0	0	0	0	0
AN0299_C	0	0	0	0	0	0	0	0	0	0	0	0
AN0300_C	0	0	0	0	0	0	0	0	0	0	0	0
AN0301_C	0	0	0	0	0	0	0	0	0	0	0	0
AN0302_C	0	0	0	0	0	0	0	0	0	0	0	0
AN0303_C	0	0	0	0	0	0	0	0	0	0	0	0
AN0304_C	0	0	0	0	0	0	0	0	0	0	0	0
AN0305_C	0	0	0	0	0	0	0	0	0	0	0	0
AN0306_C	0	0	0	0	0	0	0	0	0	0	0	0
AN0307_C	0	0	0	0	0	0	0	0	0	0	0	0
AN0308_C	0	0	0	0	0	0	0	0	0	0	0	0
AN0309_C	0	0	0	0	0	0	0	0	0	0	0	0
AN0310_C	0	0	0	0	0	0	0	0	0	0	0	0
AN0311_C	0	0	0	0	0	0	0	0	0	0	0	0
AN0312_C	0	0	0	0	0	0	0	0	0	0	0	0
AN0313_C	0	0	0	0	0	0	0	0	0	0	0	0
AN0314_C	0	0	0	0	0	0	0	0	0	0	0	0
AN0315_C	0	0	0	0	0	0	0	0	0	0	0	0
AN0316_C	0	0	0	0	0	0	0	0	0	0	0	0
AN0317_C	0	0	0	0	0	0	0	0	0	0	0	0
AN0318_C	0	0	0	0	0	0	0	0	0	0	0	0
AN0319_C	0	0	0	0	0	0	0	0	0	0	0	0
AN0320_C	0	0	0	0	0	0	0	0	0	0	0	0
AN0321_C	0	0	0	0	0	0	0	0	0	0	0	0
AP0002_C	0	0	0	0	0	0	0	0	0	0	0	0
AP0005_C	0	0	0	0	0	0	0	0	0	0	0	0
AP0006_C	0	0	0	0	0	0	0	0	0	0	0	0
AP0007_C	0	0	0	0	0	0	0	0	0	0	0	0
AP0008_C	0	0	0	0	0	0	0	0	0	0	0	0
AP0009_C	0	0	0	0	0	0	0	0	0	0	0	0
AP0010_C	0	0	0	0	0	0	0	0	0	0	0	0
AP0011_C	0	0	0	0	0	0	0	0	0	0	0	0
AP0014_C	0	0	0	0	0	0	0	0	0	0	0	0
AP0017_C	0	0	0	0	0	0	0	0	0	0	0	0
AP0018_C	0	0	0	0	0	0	0	0	0	0	0	0
AP0019_C	0	0	0	0	0	0	0	0	0	0	0	0
AP0020_C	0	0	0	0	0	0	0	0	0	0	0	0
AP0021_C	0	0	0	0	0	0	0	0	0	0	0	0
AP0022_C	0	0	0	0	0	0	0	0	0	0	0	0
AP0023_C	0	0	0	0	0	0	0	0	0	0	0	0
AP0024_C	0	0	0	0	0	0	0	0	0	0	0	0
AP0025_C	0	0	0	0	0	0	0	0	0	0	0	0
AP0030_C	0	0	0	0	0	0	0	0	0	0	0	0
AP0031_C	0	0	0	0	0	0	0	0	0	0	0	0
AP0032_C	0	0	0	0	0	0	0	0	0	0	0	0
AP0033_C	0	0	0	0	0	0	0	0	0	0	0	0
AP0034_C	0	0	0	0	0	0	0	0	0	0	0	0
AP0035_C	0	0	0	0	0	0	0	0	0	0	0	0
AQ0001_C	0	0	0	0	0	0	0	0	0	0	0	0
AQ0002_C	0	0	0	0	0	0	0	0	0	0	0	0
AQ0004_C	0	0	0	0	0	0	0	0	0	0	0	0
AQ0005_C	0	0	0	0	0	0	0	0	0	0	0	0
AQ0011_C	0	0	0	0	0	0	0	0	0	0	0	0
AQ0012_C	0	0	0	0	0	0	0	0	0	0	0	0
AQ0013_C	0	0	0	0	0	0	0	0	0	0	0	0
AQ0014_C	0	0	0	0	0	0	0	0	0	0	0	0
AQ0015_C	0	0	0	0	0	0	0	0	0	0	0	0
AR0001_C	0	0	0	0	0	0	0	0	0	0	0	0
AR0007_C	0	0	0	0	0	0	0	0	0	0	0	0

	Dup 0	Dup 1	Dup 2	Dup 3	Dup 4	Dup 5	Dup 6	Dup 7	Dup 8	Dup 9	Dup 10	Dup 11
AR0008_C	0	0	0	0	0	0	0	0	0	0	0	0
AR0009_C	0	0	0	0	0	0	0	0	0	0	0	0
AR0010_C	0	0	0	0	0	0	0	0	0	0	0	0
AR0011_C	0	0	0	0	0	0	0	0	0	0	0	0
AR0012_C	0	0	0	0	0	0	0	0	0	0	0	0
AR0013_C	0	0	0	0	0	0	0	0	0	1	0	0
AR0014_C	0	0	0	0	0	0	0	0	0	0	0	0
AR0015_C	0	0	0	0	0	0	0	0	0	1	0	0
AR0016_C	0	0	0	0	0	0	0	0	0	0	0	0
AR0017_C	0	0	0	0	0	0	0	0	0	0	0	0
AR0018_C	0	0	0	0	0	0	0	0	0	0	0	0
AR0019_C	0	0	0	0	0	0	0	0	0	0	0	0
AR0020_C	0	0	0	0	0	0	0	0	0	0	0	0
AR0021_C	0	0	0	0	0	0	0	0	0	0	0	0
AR0022_C	0	0	0	0	0	0	0	0	0	1	0	0
AR0023_C	0	0	0	0	0	0	0	0	0	0	0	0
AR0024_C	0	0	0	0	0	0	0	0	0	0	0	0
AR0026_C	0	0	0	0	0	0	0	0	0	0	0	0
AR0027_C	0	0	0	0	0	0	0	0	0	0	0	0
AR0034_C	0	0	0	0	0	0	0	0	0	0	0	0
AR0035_C	0	0	0	0	0	0	0	0	0	0	0	0
AR0036_C	0	0	0	0	0	0	0	0	0	0	0	0
AR0038_C	0	0	0	0	0	0	0	0	0	0	0	0
AR0040_C	0	0	0	0	0	0	0	0	0	0	0	0
AR0042_C	0	0	0	0	0	0	0	0	0	0	0	0
AR0043_C	0	0	0	0	0	0	0	0	0	0	0	0
AR0044_C	0	0	0	0	0	0	0	0	0	0	0	0
AR0045_C	0	0	0	0	0	0	0	0	0	0	0	0
AR0046_C	0	0	0	0	0	0	0	0	0	0	0	0
AR0047_C	0	0	0	0	0	0	0	0	0	0	0	0
AR0048_C	0	0	0	0	0	0	0	0	0	0	0	0
AR0049_C	0	0	0	0	0	0	0	0	0	1	0	0
AR0050_C	0	0	0	0	0	0	0	0	0	0	0	0
AR0051_C	0	0	0	0	0	0	0	0	0	0	0	0
AR0052_C	0	0	0	0	0	0	0	0	0	0	0	0
AR0053_C	0	0	0	0	0	0	0	0	0	0	0	0
AR0054_C	0	0	0	0	0	0	0	0	0	0	0	0
AR0057_C	0	0	0	0	0	0	0	0	0	0	0	0
AR0059_C	0	0	0	0	0	0	0	0	0	0	0	0
AR0060_C	0	0	0	0	0	0	0	0	0	0	0	0
AR0061_C	0	0	0	0	0	0	0	0	0	0	0	0
AR0062_C	0	0	0	0	0	0	0	0	0	0	0	0
AR0063_C	0	0	0	0	0	0	0	0	0	0	0	0
AR0064_C	0	0	0	0	0	0	0	0	0	0	0	0
AR0065_C	0	0	0	0	0	0	0	0	0	0	0	0
AR0066_C	0	0	0	0	0	0	0	0	0	0	0	0
AR0069_C	0	0	0	0	0	0	0	0	0	1	0	0
AR0070_C	0	0	0	0	0	0	0	0	0	0	0	0
AR0071_C	0	0	0	0	0	0	0	0	0	0	0	0
AR0072_C	0	0	0	0	0	0	0	0	0	0	0	0
AR0073_C	0	0	0	0	0	0	0	0	0	0	0	0
AR0074_C	0	0	0	0	0	0	0	0	0	0	0	0
AR0075_C	0	0	0	0	0	0	0	0	0	0	0	0
AR0076_C	0	0	0	0	0	0	0	0	0	0	0	0
AR0077_C	0	0	0	0	0	0	0	0	0	0	0	0
AR0078_C	0	0	0	0	0	0	0	0	0	0	0	0
AR0079_C	0	0	0	0	0	0	0	0	0	NA	0	0
AR0080_C	0	0	0	0	0	0	0	0	0	0	0	0
AR0081_C	0	0	0	0	0	0	0	0	0	0	0	0
AR0082_C	0	0	0	0	0	0	0	0	0	NA	0	0
AR0083_C	0	0	0	0	0	0	0	0	0	0	0	0
AR0084_C	0	0	0	0	0	0	0	0	0	0	0	0
AR0085_C	0	0	0	0	0	0	0	0	0	0	0	0
AR0086_C	0	0	0	0	0	0	0	0	0	0	0	0
AR0087_C	0	0	0	0	0	0	0	0	0	0	0	0
AR0088_C	0	0	0	0	0	0	0	0	0	0	0	0
AR0089_C	0	0	0	0	0	0	0	0	0	0	0	0
AR0090_C	0	0	0	0	0	0	0	0	0	0	0	0

	Dup 0	Dup 1	Dup 2	Dup 3	Dup 4	Dup 5	Dup 6	Dup 7	Dup 8	Dup 9	Dup 10	Dup 11
AR0092_C	0	0	0	0	0	0	0	0	0	0	0	0
AR0093_C	0	0	0	0	0	0	0	0	0	0	0	0
AR0095_C	0	0	0	0	0	0	0	0	0	0	0	0
AR0096_C	0	0	0	0	0	0	0	0	0	0	0	0
AR0097_C	0	0	0	0	0	0	0	0	0	1	0	0
AR0098_C	0	0	0	0	0	0	0	0	0	1	0	0
AR0099_C	0	0	0	0	0	0	0	0	0	0	0	0
AR0100_C	0	0	0	0	0	0	0	0	0	0	0	0
AS0001_C	0	0	0	0	0	0	0	0	0	0	0	0
AS0002_C	0	0	0	0	0	0	0	0	0	0	0	0
AS0003_C	0	0	0	0	0	0	0	0	0	0	0	0
AS0004_C	0	0	0	0	0	0	0	0	0	0	0	0
AS0005_C	0	0	0	0	0	0	0	0	0	0	0	0
AS0006_C	0	0	0	0	0	2	0	0	0	0	0	0
AS0007_C	0	0	0	0	0	0	0	0	0	0	0	0
AS0008_C	0	0	0	0	0	0	0	0	0	0	0	0
AS0009_C	0	0	0	0	0	0	0	0	0	0	0	0
AS0010_C	0	0	0	0	0	0	0	0	0	0	0	0
AS0011_C	0	0	0	0	0	0	0	0	0	0	0	0
AS0012_C	0	0	0	0	0	0	0	0	0	0	0	0
AS0013_C	0	0	0	0	0	0	0	0	0	0	0	0
AS0014_C	0	0	0	0	0	0	0	0	0	0	0	0
AS0015_C	0	0	0	0	0	0	0	0	0	0	0	0
AS0016_C	0	0	0	0	0	0	0	0	0	0	0	0
AS0017_C	0	0	0	0	0	0	0	0	0	0	0	0
AS0018_C	0	0	0	0	0	0	0	0	0	0	0	0
AS0019_C	0	0	0	0	0	0	0	0	0	0	0	0
AS0020_C	0	0	0	0	0	0	0	0	0	0	0	0
AS0021_C	0	0	0	0	0	0	0	0	0	0	0	0
AS0022_C	0	0	0	0	0	0	0	0	0	0	0	0
AS0024_C	0	0	0	0	0	0	0	0	0	0	0	0
AS0025_C	0	0	0	0	0	0	0	0	0	0	0	0
AS0026_C	0	0	0	0	0	0	0	0	0	0	0	0
AS0027_C	0	0	0	0	0	0	0	0	0	0	0	0
AS0028_C	0	0	0	0	0	0	0	0	0	0	0	0
AS0029_C	0	0	0	0	0	0	0	0	0	0	0	0
AS0030_C	0	0	0	0	0	0	0	0	0	0	0	0
AS0032_C	0	0	0	0	0	0	0	0	0	0	0	0
AS0033_C	0	0	0	0	0	2	0	0	0	0	0	0
AS0034_C	0	0	0	0	0	0	0	0	0	0	0	0
AS0035_C	0	0	0	0	0	2	0	0	0	0	0	0
AS0036_C	0	0	0	0	0	0	0	0	0	0	0	0
AS0037_C	0	0	0	0	0	0	0	0	0	0	0	0
AS0039_C	0	0	0	0	0	0	0	0	0	0	0	0
AS0040_C	0	0	0	0	0	2	0	0	0	0	0	0
AS0041_C	0	0	0	0	0	0	0	0	0	0	0	0
AS0042_C	0	0	0	0	0	0	0	0	0	0	0	0
AS0044_C	0	0	0	0	0	0	0	0	0	0	0	0
AS0045_C	0	0	0	0	0	0	0	0	0	0	0	0
AS0046_C	0	0	0	0	0	0	0	0	0	0	0	0
AS0047_C	0	0	0	0	0	2	0	0	0	0	0	0
AS0048_C	0	0	0	0	0	0	0	0	0	0	0	0
AS0049_C	0	0	0	0	0	0	0	0	0	0	0	0
AS0051_C	0	0	0	0	0	0	0	0	0	0	0	0
AS0052_C	0	0	0	0	0	2	0	0	0	0	0	0
AS0053_C	0	0	0	0	0	0	0	0	0	0	0	0
AS0054_C	0	0	0	0	0	0	0	0	0	0	0	0
AS0055_C	0	0	0	0	0	0	0	0	0	0	0	0
AS0056_C	0	0	0	0	0	0	0	0	0	0	0	0
AS0057_C	0	0	0	0	0	0	0	0	0	0	0	0
AS0058_C	0	0	0	0	0	2	0	0	0	0	0	0
AS0059_C	0	0	0	0	0	0	0	0	0	0	0	0
AS0060_C	0	0	0	0	0	0	0	0	0	0	0	0
AS0062_C	0	0	0	0	0	0	0	0	0	0	0	0
AS0064_C	0	0	0	0	0	0	0	0	0	0	0	0
AS0065_C	0	0	0	0	0	0	0	0	0	0	0	0
AS0066_C	0	0	0	0	0	0	0	0	0	0	0	0
AS0068_C	0	0	0	0	0	0	0	0	0	0	0	0

	Dup 0	Dup 1	Dup 2	Dup 3	Dup 4	Dup 5	Dup 6	Dup 7	Dup 8	Dup 9	Dup 10	Dup 11
AS0069_C	0	0	0	0	0	0	0	0	0	0	0	0
AS0070_C	0	0	0	0	0	0	0	0	0	0	0	0
AS0071_C	0	0	0	0	0	0	0	0	0	0	0	0
AS0072_C	0	0	0	0	0	0	0	0	0	0	0	0
AS0073_C	0	0	0	0	0	0	0	0	0	0	0	0
AS0074_C	0	0	0	0	0	0	0	0	0	0	0	0
AS0076_C	0	0	0	0	0	2	0	0	0	0	0	0
AS0077_C	0	0	0	0	0	2	0	0	0	0	0	0
AS0078_C	0	0	0	0	0	0	0	0	0	0	0	0
AV0001_C	0	0	0	0	0	0	0	0	0	0	0	0
AV0002_C	0	0	0	0	0	0	0	0	0	0	0	0
AV0003_C	0	0	0	0	0	0	0	0	0	0	0	0
AV0004_C	0	0	0	0	0	0	0	0	0	0	0	0
AV0005_C	0	0	0	0	0	0	0	0	0	0	0	0
AV0006_C	0	0	0	0	0	0	0	0	0	0	0	0
AV0007_C	0	0	0	0	0	0	0	0	0	0	0	0
AV0008_C	0	0	0	0	0	0	0	0	0	0	0	0
AV0009_C	0	0	0	0	0	0	0	0	0	0	0	0
AV0010_C	0	0	0	0	0	0	0	0	0	0	0	0
AV0011_C	0	0	0	0	0	0	0	0	0	0	0	0
AV0012_C	0	0	0	0	0	0	0	0	0	0	0	0
AV0013_C	0	0	0	0	0	0	0	0	0	0	0	0
AV0014_C	0	0	0	0	0	0	0	0	0	0	0	0
AV0015_C	0	0	0	0	0	0	0	0	0	0	0	0
AV0017_C	0	0	0	0	0	0	0	0	0	0	0	0
AV0018_C	0	0	0	0	0	0	0	0	0	0	0	0
AV0020_C	0	0	0	0	0	0	0	0	0	0	0	0
AV0021_C	0	0	0	0	0	0	0	0	0	0	0	0
AV0022_C	0	0	0	0	0	0	0	0	0	0	0	0
AV0024_C	0	0	0	0	0	0	0	0	0	0	0	0
AV0025_C	0	0	0	0	0	0	0	0	0	0	0	0
AV0026_C	0	0	0	0	0	0	0	0	0	0	0	0
AV0027_C	0	0	0	0	0	0	0	0	0	0	0	0
AV0028_C	0	0	0	0	0	0	0	0	0	0	0	0
AV0029_C	0	0	0	0	0	0	0	0	0	0	0	0
AV0030_C	0	0	0	0	0	0	0	0	0	0	0	0
AV0031_C	0	0	0	0	0	0	0	0	0	0	0	0
AV0032_C	0	0	0	0	0	0	0	0	0	0	0	0
AV0033_C	0	0	0	0	0	0	0	0	0	0	0	0
AV0034_C	0	0	0	0	0	0	0	0	0	0	0	0
AV0035_C	0	0	0	0	0	0	0	0	0	0	0	0
AV0036_C	0	0	0	0	0	0	0	0	0	0	0	0
AV0037_C	0	0	0	0	0	0	0	0	0	0	0	0
AV0038_C	0	0	0	0	0	0	0	0	0	0	0	0
AV0039_C	0	0	0	0	0	0	0	0	0	0	0	0
AV0040_C	0	0	0	0	0	0	0	0	0	0	0	0
AV0041_C	0	0	0	0	0	0	0	0	0	0	0	0
AV0042_C	0	0	0	0	0	0	0	0	0	0	0	0
AV0043_C	0	0	0	0	0	0	0	0	0	0	0	0
AV0044_C	0	0	0	0	0	0	0	0	0	0	0	0
AV0045_C	0	0	0	0	0	0	0	0	0	0	0	0
AV0046_C	0	0	0	0	0	0	0	0	0	0	0	0
AV0047_C	0	0	0	0	0	0	0	0	0	0	0	0
AY0006_C	0	0	0	0	0	0	0	0	0	0	0	0
AY0007_C	0	0	0	0	0	0	0	0	0	0	0	0
AY0010_C	0	0	0	0	0	0	0	0	0	0	0	0
AY0011_C	0	0	0	0	0	0	0	0	0	0	0	0
AY0012_C	0	0	0	0	0	0	0	0	0	0	0	0
AY0013_C	0	0	0	0	0	0	0	0	0	0	0	0
AY0015_C	0	0	0	0	0	0	0	0	0	0	0	0
AY0016_C	0	0	0	0	0	0	0	0	0	0	0	0
AY0017_C	0	0	0	0	0	0	0	0	0	0	0	0
AY0018_C	0	0	0	0	0	0	0	0	0	0	0	0
AY0019_C	0	0	0	0	0	0	0	0	0	0	0	0
AY0020_C	0	0	0	0	0	0	0	0	0	0	0	0
AY0021_C	0	0	0	0	0	0	0	0	0	0	0	0
AY0023_C	0	0	0	0	0	0	0	0	0	0	0	0
AY0024_C	0	0	0	0	0	0	0	0	0	0	0	0

	Dup 0	Dup 1	Dup 2	Dup 3	Dup 4	Dup 5	Dup 6	Dup 7	Dup 8	Dup 9	Dup 10	Dup 11
AY0025_C	0	0	0	0	0	0	0	0	0	0	0	0
AY0026_C	0	0	0	0	0	0	0	0	0	0	0	0
AY0027_C	0	0	0	0	0	0	0	0	0	0	0	0
AY0029_C	0	0	0	0	0	0	0	0	0	0	0	0
AY0031_C	0	0	0	0	0	0	0	0	0	0	0	0
AY0032_C	0	0	0	0	0	0	0	0	0	0	0	0
AY0033_C	0	0	0	0	0	0	0	0	0	0	0	0
AY0034_C	0	0	0	0	0	0	0	0	0	0	0	0
AY0035_C	0	0	0	0	0	0	0	0	0	0	0	0
AY0036_C	0	0	0	0	0	0	0	0	0	0	0	0
AY0038_C	0	0	0	0	0	0	0	0	0	0	0	0
AY0039_C	0	0	0	0	0	0	0	0	0	0	0	0
AY0040_C	0	0	0	0	0	0	0	0	0	0	0	0
AY0041_C	0	0	0	0	0	0	0	0	0	0	0	0
AY0042_C	0	0	0	0	0	0	0	0	0	0	0	0
AY0043_C	0	0	0	0	0	0	0	0	0	0	0	0
AY0045_C	0	0	0	0	0	0	0	0	0	0	0	0
AY0046_C	0	0	0	0	0	0	0	0	0	0	0	0
AY0047_C	0	0	0	0	0	0	0	0	0	0	0	0
AY0048_C	0	0	0	0	0	0	0	0	0	0	0	0
AY0049_C	0	0	0	0	0	0	0	0	0	0	0	0
AY0050_C	0	0	0	0	0	0	0	0	0	0	0	0
AY0052_C	0	0	0	0	0	0	0	0	0	0	0	0
AY0053_C	0	0	0	0	0	0	0	0	0	0	0	0
AY0054_C	0	0	0	0	0	0	0	0	0	0	0	0
AY0055_C	0	0	0	0	0	0	0	0	0	0	0	0
AY0056_C	0	0	0	0	0	0	0	0	0	0	0	0
AY0057_C	0	0	0	0	0	0	0	0	0	0	0	0
AY0058_C	0	0	0	0	0	0	0	0	0	0	0	0
AY0059_C	0	0	0	0	0	0	0	0	0	0	0	0
AY0060_C	0	0	0	0	0	0	0	0	0	0	0	0
AY0061_C	0	0	0	0	0	0	0	0	0	0	0	0
AY0062_C	0	0	0	0	0	0	0	0	0	0	0	0
AY0063_C	0	0	0	0	0	0	0	0	0	0	0	0
AY0064_C	0	0	0	0	0	0	0	0	0	0	0	0
AY0065_C	0	0	0	0	0	0	0	0	0	0	0	0
AY0066_C	0	0	0	0	0	0	0	0	0	0	0	0
AY0067_C	0	0	0	0	0	0	0	0	0	0	0	0
AY0068_C	0	0	0	0	0	0	0	0	0	0	0	0
AY0069_C	0	0	0	0	0	0	0	0	0	0	0	0
AY0070_C	0	0	0	0	0	0	0	0	0	0	0	0
AY0072_C	0	0	0	0	0	0	0	0	0	0	0	0
AY0074_C	0	0	0	0	0	0	0	0	0	0	0	0
AY0076_C	0	0	0	0	0	0	0	0	0	0	0	0
AY0077_C	0	0	0	0	0	0	0	0	0	0	0	0
AY0078_C	0	0	0	0	0	0	0	0	0	0	0	0
AY0079_C	0	0	0	0	0	0	0	0	0	0	0	0
AY0080_C	0	0	0	0	0	0	0	0	0	0	0	0
AY0082_C	0	0	0	0	0	0	0	0	0	0	0	0
AY0083_C	0	0	0	0	0	0	0	0	0	0	0	0
AY0085_C	0	0	0	0	0	0	0	0	0	0	0	0
AY0087_C	0	0	0	0	0	0	0	0	0	0	0	0
AY0088_C	0	0	0	0	0	0	0	0	0	0	0	0
AY0089_C	0	0	0	0	0	0	0	0	0	0	0	0
AY0090_C	0	0	0	0	0	0	0	0	0	0	0	0
AY0091_C	0	0	0	0	0	0	0	0	0	0	0	0
