# Supplementary material for: Whole-genome sequencing reveals high complexity of copy number variation at insecticide resistance loci in malaria mosquitoes
Source: Genome Res. 2019 Aug;29(8):1250–61. doi: 10.1101/gr.245795.118 (PMC6673711; doi:10.1101/gr.245795.118)
Supplement: Supplemental Material [file supp_gr.245795.118_Supplementary_Data_S6.pdf]

Electronic Supplementary Material S6

Description of CNV alleles detected in the CYP6M - CYP6Z region in Ag1000G phase 2.

Overview of CNVs in the CYP6M - CYP6Z region.

Two CNV alleles were found, one that covered CYP6M2 only and another that covered CYP6Z1, CYP6Z2 and CYP6Z3. The regions covered by these CNVs are shown in Fig. MZ_S1.

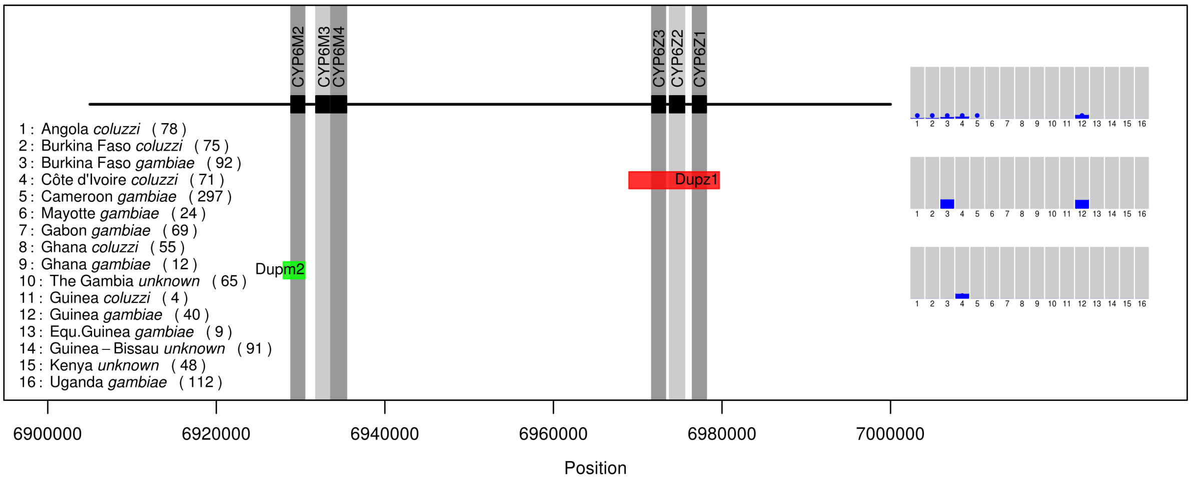

Fig. MZ_S1: Overview of all the CNVs in the region ranging from CYP6M2 to CYP6Z1 found in Phase 2 of the Ag1000G dataset (alleles are named Cyp6m_Dup1 and Cyp6z_Dup1, abbreviated to Dupm2 and Dupz1 in the plot). Position on chromosome 3R is shown on the X axis. Barplots on the right show the proportion of samples that carry a given CNV in each of the Phase 2 populations. Numbers below the barplots are numeric population IDs detailed to the left of the barplots (numbers in brackets indicate the total number of samples from that population). Blue points on the barplots indicate that at least one sample in this population carried the CNV. The top barplot (Cyp6mz_Dup0) shows CNVs that could not be categorised, each subsequent barplot represents the CNV shown to its left.

Notes

Twelve samples have CNVs according to coverage but that could not be assigned to a duplication type based on discordant read or breakpoint read data (Cyp6mz_Dup0 in Fig. **MZ_S1**). Based on visual inspection of coverage, most of these are likely to be instances of Cyp6m_Dup1 and Cyp6z_Dup1 that could not be detected using the discordant or breakpoint reads.

Duplication in CYP6Z1

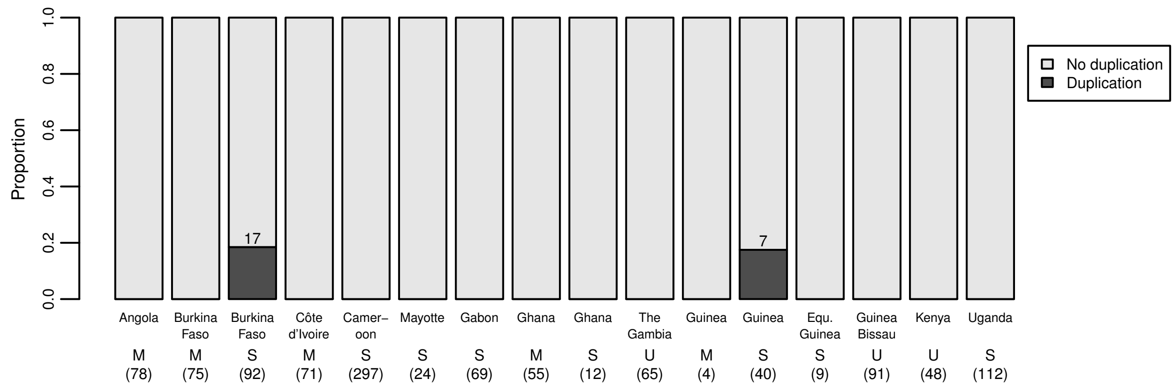

Fig. MZ_S2: Barplot showing the proportion of samples that carry the Cyp6z_Dup1 duplication in each of the Phase 2 populations. Numbers above the dark grey bars indicate the absolute number of samples carrying the duplication. S = *Anopheles gambiae*, M = *Anopheles coluzzi*, U = species undetermined. Numbers in brackets indicate the total number of samples from that population.

Cyp6z_Dup1 was supported by face-away read pairs whose forward-facing read mapped in the interval 6968950 - 6969250 and whose reverse-facing read mapped in the interval 6979300 - 6979600 (Fig. **MZ_S3**). However, these discordant reads were not present in all samples carrying Cyp6z_Dup1. Cyp6z_Dup1 was also supported by reads mapping to the duplication breakpoint (soft-clipped at positions 6968962 and 6979681 for the start point and end point respectively). These were also not always present and many samples remained that appear to carry Cyp6z_Dup1 according to the HMM but could not be confirmed using discordant or soft-clipped reads.

Cyp6z_Dup1 breakpoint:

```
TCGAATCAATCTTCGCA CTCC AGGTTAAACAACAGCGG
 end of the dup ^      ^ start of the dup
 position 6979676      position 6968967
```

The CTCC could sit on either side of the breakpoint.

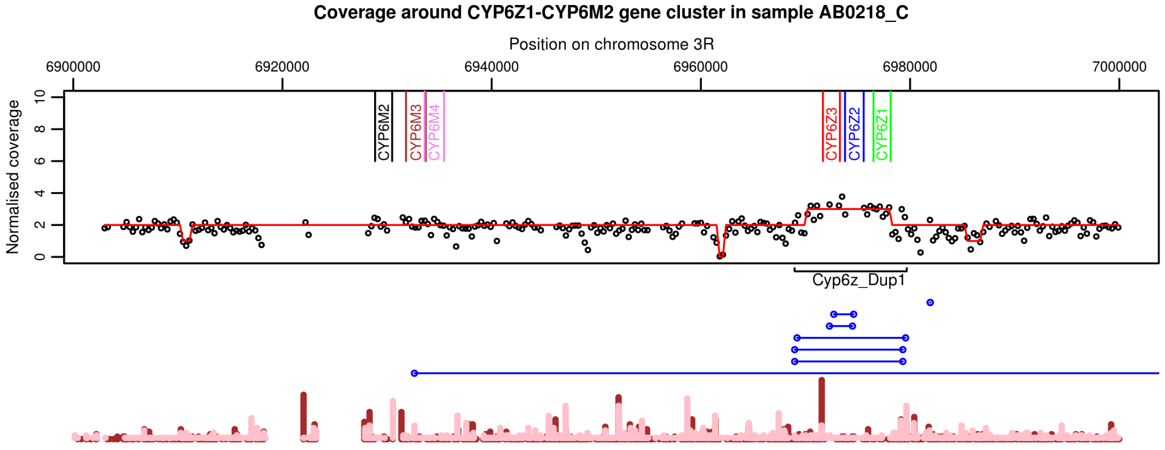

Fig. MZ_S3: Example of coverage in an individual carrying the Cyp6z_Dup1 duplication. Open black circles indicate coverage at each position. The red line shows the HMM estimation of the coverage state at each position. Vertical lines represent the positions of the various genes. Pairs of blue points connected by lines indicate pairs of face-away reads. Reads soft-clipped before the alignment start point (dark brown points) and after the alignment end point (light brown points) are present at the start and end points of the duplication (clipped at positions 6968962 and 6979681 respectively). In each case, the clipped bases align to the other end of the duplication, as expected.

All of the samples with Cyp6z_Dup1 were found in *An. gambiae* from Burkina Faso and Guinea Tables MZ_S1.1 & 1.2). Estimates of copy number for Cyp6z_Dup1 in these populations indicated that all sample had a copy number of 1. Assuming that these individuals are all heterozygotes for Cyp6z_Dup1, the allele distribution is consistent with Hardy-Weinberg (HW) expectations ($P = 1$ in both Burkina Faso and Guinea).

Table ZM_S1.1: Coverage calls for Cyp6z_Dup1. NAs were produced if coverage was too variable or if the duplication completely overlapped with another duplication whose coverage could also not be called.

copy number	AO col	BF col	BF gam	CI col	CM gam	FR gam	GA gam	GH col	GH gam	GM	GN col	GN gam	GQ gam	GW	KE	UG gam
0	78	75	77	71	297	24	69	55	12	65	4	35	9	91	48	112
1	0	0	15	0	0	0	0	0	0	0	0	5	0	0	0	0

Table ZM_S1.2: Coverage calls for all duplications in individuals that carry Cyp6z_Dup1.

	Cyp6mz _Dup0	Cyp6m _Dup1	Cyp6z _Dup1
AB0103_C	0	1	0
AB0127_C	0	1	0
AB0130_C	0	1	0
AB0153_C	0	1	0
AB0161_C	0	1	0
AB0173_C	0	1	0
AB0178_C	0	1	0
AB0197_C	0	1	0
AB0198_C	0	1	0
AB0202_C	0	1	0
AB0203_C	0	1	0
AB0218_C	0	1	0
AB0256_C	0	1	0
AB0275_C	0	1	0
AB0278_C	0	1	0
AV0002_C	0	1	0
AV0003_C	0	1	0
AV0014_C	0	1	0
AV0015_C	0	1	0
AV0047_C	0	1	0

Duplication in CYP6M2

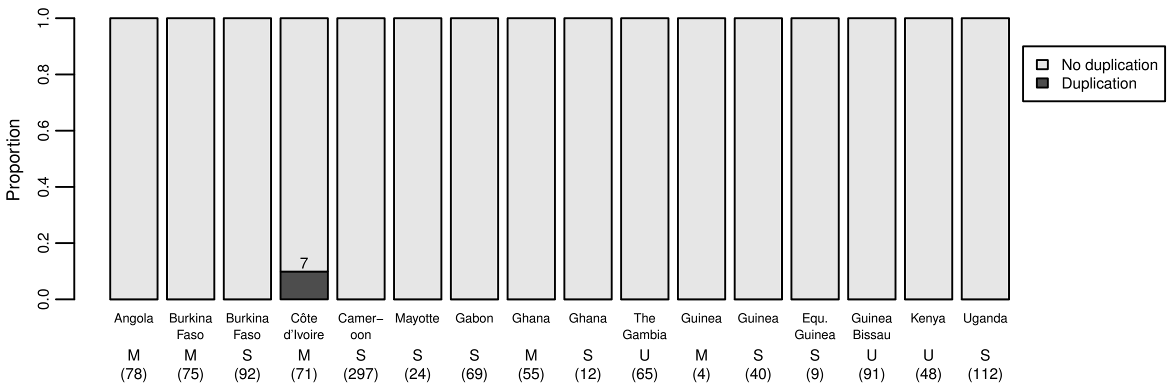

Fig. MZ_S4: Barplot showing the proportion of samples that carry the Cyp6m_Dup1 duplication in each of the Phase 2 populations. Numbers above the dark grey bars indicate the absolute number of samples carrying the duplication. S = *Anopheles gambiae*, M = *Anopheles coluzzi*, U = species undetermined. Numbers in brackets indicate the total number of samples from that population.

Cyp6m_Dup1 was not supported by any discordant reads that we could find. (Fig. **MZ_S5**). Looking for soft-clipped reads that were present only in samples carrying Cyp6m_Dup1 according to the HMM, we found reads soft clipped at the start point (position 6927942), but found no such reads at the end point. These soft-clipped reads were not always present and many samples remained that appeared to carry Cyp6m_Dup1 according to the HMM but could not be confirmed using the soft-clipped reads.

Cyp6m_Dup1 breakpoint:

```
TATATTATACCAAATTATTA   TACAAACCAAATTATACAAAA
       end of the dup ^   ^ start of the dup
       position ???????   position 6927943
```

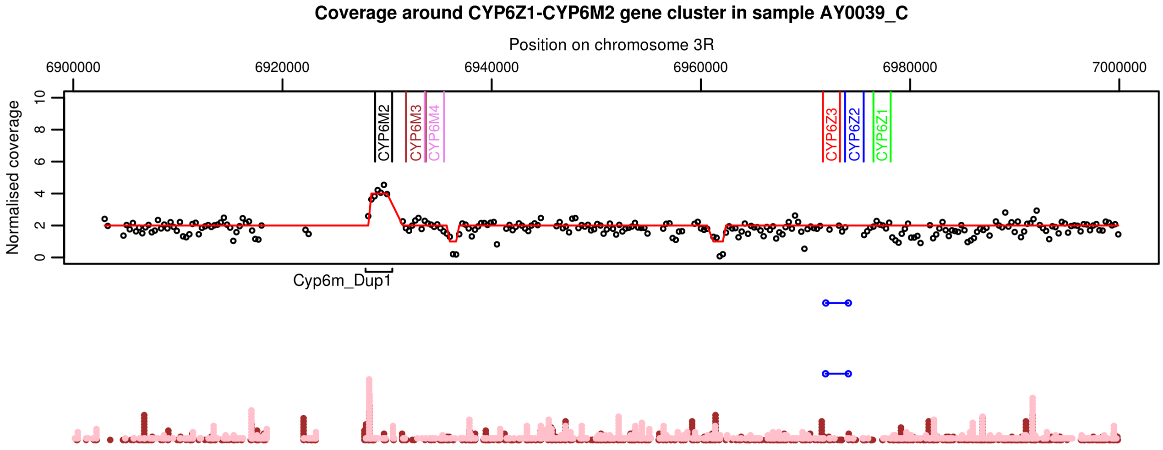

Fig. MZ_S5: Coverage in the individual carrying the Cyp6m_Dup1 duplication. Open black circles indicate coverage at each position. The red line shows the HMM estimation of the coverage state at each position. Vertical lines represent the positions of the various gene. Pairs of blue points connected by lines indicate pairs of face-away reads. Reads soft-clipped before the alignment start point (dark brown points) are present at the start of the duplication (clipped at position 6927942). Light brown points show reads soft-clipped after the alignment end point.

Samples with Cyp6m_Dup1 are mostly found in *An. coluzzii* from Côte d'Ivoire (Tables ZM_S1.1 & 1.2), with one sample in Burkina Faso that appears to carry this duplication according to the HMM but which could not be confirmed using soft-clipped reads. Estimates of copy number indicate that the copy number for samples with Cyp6m_Dup1 is consistently 2, suggesting that Cyp6m_Dup1 is a triplication. Assuming samples with a copy number of 2 are heterozygotes for Cyp6m_Dup1, the allele distribution is consistent with HW expectations ($P = 1$).

Table ZM_S2.1: Coverage calls for Cyp6m_Dup1. NAs were produced if coverage was too variable or if the duplication completely overlapped with another duplication whose coverage could also not be called.

copy number	AO col	BF col	BF gam	CI col	CM gam	FR gam	GA gam	GH col	GH gam	GM	GN col	GN gam	GQ gam	GW	KE	UG gam
0	78	75	92	66	297	24	69	55	12	65	4	40	9	91	48	112
2	0	0	0	5	0	0	0	0	0	0	0	0	0	0	0	0

Table ZM_S2.2: Coverage calls for all duplications in individuals that carry Cyp6m_Dup1.

	Cyp6mz _Dup0	Cyp6m _Dup1	Cyp6z _Dup1
AY0017_C	0	0	2
AY0039_C	0	0	2
AY0053_C	0	0	2
AY0060_C	0	0	2
AY0069_C	0	0	2

Table ZM_S3: Coverage calls for all duplications in the Cyp6z-Cyp6m cluster in all individuals.

	Cyp6mz _Dup0	Cyp6m _Dup1	Cyp6z _Dup1
AA0040_C	0	0	0
AA0041_C	0	0	0
AA0042_C	0	0	0
AA0043_C	0	0	0
AA0044_C	0	0	0
AA0048_C	0	0	0
AA0049_C	0	0	0
AA0050_C	0	0	0
AA0051_C	0	0	0
AA0052_C	0	0	0
AA0053_C	0	0	0
AA0054_C	0	0	0
AA0055_C	0	0	0
AA0056_C	0	0	0
AA0060_C	0	0	0
AA0061_C	0	0	0
AA0063_C	0	0	0
AA0064_C	0	0	0
AA0066_C	0	0	0
AA0067_C	0	0	0
AA0068_C	0	0	0
AA0072_C	0	0	0
AA0073_C	0	0	0
AA0074_C	0	0	0
AA0075_C	0	0	0
AA0076_C	0	0	0
AA0077_C	0	0	0
AA0080_C	0	0	0
AA0084_C	0	0	0
AA0085_C	0	0	0
AA0086_C	0	0	0
AA0087_C	0	0	0
AA0088_C	0	0	0
AA0089_C	0	0	0
AA0090_C	0	0	0
AA0091_C	0	0	0
AA0096_C	0	0	0
AA0097_C	0	0	0
AA0098_C	0	0	0
AA0099_C	0	0	0
AA0100_C	0	0	0
AA0101_C	0	0	0
AA0102_C	0	0	0
AA0103_C	0	0	0
AA0104_C	0	0	0
AA0107_C	0	0	0
AA0108_C	0	0	0
AA0109_C	0	0	0
AA0110_C	0	0	0
AA0111_C	0	0	0
AA0113_C	0	0	0
AA0114_C	0	0	0
AA0115_C	0	0	0
AA0116_C	0	0	0
AA0122_C	0	0	0
AA0123_C	0	0	0
AA0124_C	0	0	0
AA0125_C	0	0	0
AA0127_C	0	0	0
AA0132_C	0	0	0
AA0133_C	0	0	0
AA0134_C	0	0	0
AA0135_C	0	0	0
AA0136_C	0	0	0
AA0139_C	0	0	0
AA0140_C	0	0	0
AA0141_C	0	0	0
AB0085_C	0	0	0

	Cyp6mz _Dup0	Cyp6m _Dup1	Cyp6z _Dup1
AB0087_C	0	0	0
AB0088_C	0	0	0
AB0089_C	0	0	0
AB0090_C	0	0	0
AB0091_C	0	0	0
AB0092_C	0	0	0
AB0094_C	0	0	0
AB0095_C	0	0	0
AB0097_C	0	0	0
AB0098_C	0	0	0
AB0099_C	0	0	0
AB0100_C	0	0	0
AB0101_C	0	0	0
AB0103_C	0	1	0
AB0104_C	0	0	0
AB0108_C	0	0	0
AB0109_C	0	0	0
AB0110_C	0	0	0
AB0111_C	0	0	0
AB0112_C	0	0	0
AB0113_C	0	0	0
AB0114_C	0	0	0
AB0115_C	0	0	0
AB0117_C	0	0	0
AB0118_C	0	0	0
AB0119_C	0	0	0
AB0122_C	0	0	0
AB0123_C	0	0	0
AB0124_C	0	0	0
AB0126_C	0	0	0
AB0127_C	0	1	0
AB0128_C	0	0	0
AB0129_C	0	0	0
AB0130_C	0	1	0
AB0133_C	0	0	0
AB0134_C	0	0	0
AB0135_C	0	0	0
AB0136_C	0	0	0
AB0137_C	0	0	0
AB0138_C	0	0	0
AB0139_C	0	0	0
AB0140_C	0	0	0
AB0142_C	0	0	0
AB0143_C	0	0	0
AB0145_C	0	0	0
AB0146_C	0	0	0
AB0147_C	0	0	0
AB0148_C	0	0	0
AB0150_C	0	0	0
AB0151_C	0	0	0
AB0153_C	0	1	0
AB0155_C	0	0	0
AB0157_C	0	0	0
AB0158_C	0	0	0
AB0159_C	0	0	0
AB0160_C	0	0	0
AB0161_C	0	1	0
AB0162_C	0	0	0
AB0164_C	0	0	0
AB0165_C	0	0	0
AB0166_C	0	0	0
AB0167_C	0	0	0
AB0169_C	0	0	0
AB0170_C	0	0	0
AB0171_C	0	0	0
AB0172_C	0	0	0
AB0173_C	0	1	0
AB0174_C	0	0	0
AB0175_C	0	0	0
AB0176_C	0	0	0

	Cyp6mz _Dup0	Cyp6m _Dup1	Cyp6z _Dup1
AB0177_C	0	0	0
AB0178_C	0	1	0
AB0179_C	0	0	0
AB0181_C	0	0	0
AB0182_C	0	0	0
AB0183_C	0	0	0
AB0184_C	0	0	0
AB0185_C	0	0	0
AB0186_C	0	0	0
AB0187_C	0	0	0
AB0188_C	0	0	0
AB0189_C	0	0	0
AB0190_C	0	0	0
AB0191_C	0	0	0
AB0192_C	0	0	0
AB0195_C	0	0	0
AB0196_C	0	0	0
AB0197_C	0	1	0
AB0198_C	0	1	0
AB0199_C	0	0	0
AB0200_C	0	0	0
AB0201_C	0	0	0
AB0202_C	0	1	0
AB0203_C	0	1	0
AB0204_C	0	0	0
AB0205_C	0	0	0
AB0206_C	0	0	0
AB0207_C	0	0	0
AB0208_C	0	0	0
AB0209_C	0	0	0
AB0210_C	0	0	0
AB0211_C	0	0	0
AB0212_C	0	0	0
AB0213_C	0	0	0
AB0215_C	0	0	0
AB0217_C	0	0	0
AB0218_C	0	1	0
AB0219_C	0	0	0
AB0221_C	0	0	0
AB0222_C	0	0	0
AB0223_C	0	0	0
AB0224_C	0	0	0
AB0226_C	0	0	0
AB0227_C	0	0	0
AB0228_C	0	0	0
AB0229_C	0	0	0
AB0231_C	0	0	0
AB0232_C	0	0	0
AB0233_C	0	0	0
AB0234_C	0	0	0
AB0235_C	0	0	0
AB0236_C	0	0	0
AB0237_C	0	0	0
AB0238_C	0	0	0
AB0239_C	0	0	0
AB0240_C	0	0	0
AB0241_C	0	0	0
AB0242_C	0	0	0
AB0243_C	0	0	0
AB0244_C	0	0	0
AB0246_C	0	0	0
AB0247_C	0	0	0
AB0248_C	0	0	0
AB0249_C	0	0	0
AB0250_C	0	0	0
AB0251_C	0	0	0
AB0252_C	0	0	0
AB0253_C	0	0	0
AB0255_C	0	0	0
AB0256_C	0	1	0

	Cyp6mz _Dup0	Cyp6m _Dup1	Cyp6z _Dup1
AB0257_C	0	0	0
AB0258_C	0	0	0
AB0260_C	0	0	0
AB0261_C	0	0	0
AB0262_C	0	0	0
AB0263_C	0	0	0
AB0264_C	0	0	0
AB0265_C	0	0	0
AB0266_C	0	0	0
AB0267_C	0	0	0
AB0268_C	0	0	0
AB0270_C	0	0	0
AB0271_C	0	0	0
AB0272_C	0	0	0
AB0273_C	0	0	0
AB0274_C	0	0	0
AB0275_C	0	1	0
AB0276_C	0	0	0
AB0277_C	0	0	0
AB0278_C	0	1	0
AB0279_C	0	0	0
AB0280_C	0	0	0
AB0281_C	0	0	0
AB0282_C	0	0	0
AB0283_C	0	0	0
AB0284_C	0	0	0
AC0089_C	0	0	0
AC0090_C	0	0	0
AC0091_C	0	0	0
AC0092_C	0	0	0
AC0093_C	0	0	0
AC0094_C	0	0	0
AC0095_C	0	0	0
AC0096_C	0	0	0
AC0097_C	0	0	0
AC0098_C	0	0	0
AC0099_C	0	0	0
AC0100_C	0	0	0
AC0101_C	0	0	0
AC0102_C	0	0	0
AC0103_C	0	0	0
AC0104_C	0	0	0
AC0105_C	0	0	0
AC0106_C	0	0	0
AC0107_C	0	0	0
AC0108_C	0	0	0
AC0109_C	0	0	0
AC0110_C	0	0	0
AC0111_C	0	0	0
AC0112_C	0	0	0
AC0113_C	0	0	0
AC0114_C	0	0	0
AC0115_C	0	0	0
AC0116_C	0	0	0
AC0117_C	0	0	0
AC0118_C	0	0	0
AC0119_C	0	0	0
AC0120_C	0	0	0
AC0121_C	0	0	0
AC0122_C	0	0	0
AC0123_C	0	0	0
AC0124_C	0	0	0
AC0125_C	0	0	0
AC0126_C	0	0	0
AC0127_C	0	0	0
AC0128_C	0	0	0
AC0129_C	0	0	0
AC0130_C	0	0	0
AC0131_C	0	0	0
AC0132_C	0	0	0

	Cyp6mz _Dup0	Cyp6m _Dup1	Cyp6z _Dup1
AC0133_C	0	0	0
AC0134_C	0	0	0
AC0135_C	0	0	0
AC0136_C	0	0	0
AC0137_C	0	0	0
AC0138_C	0	0	0
AC0139_C	0	0	0
AC0140_C	0	0	0
AC0141_C	0	0	0
AC0142_C	0	0	0
AC0143_C	0	0	0
AC0144_C	0	0	0
AC0145_C	0	0	0
AC0146_C	0	0	0
AC0147_C	0	0	0
AC0148_C	0	0	0
AC0149_C	0	0	0
AC0150_C	0	0	0
AC0151_C	0	0	0
AC0152_C	0	0	0
AC0153_C	0	0	0
AC0154_C	0	0	0
AC0155_C	0	0	0
AC0156_C	0	0	0
AC0157_C	0	0	0
AC0158_C	0	0	0
AC0159_C	0	0	0
AC0160_C	0	0	0
AC0161_C	0	0	0
AC0162_C	0	0	0
AC0163_C	0	0	0
AC0164_C	0	0	0
AC0166_C	0	0	0
AC0167_C	0	0	0
AC0168_C	0	0	0
AC0169_C	0	0	0
AC0170_C	0	0	0
AC0171_C	0	0	0
AC0172_C	0	0	0
AC0173_C	0	0	0
AC0174_C	0	0	0
AC0176_C	0	0	0
AC0177_C	0	0	0
AC0178_C	0	0	0
AC0179_C	0	0	0
AC0180_C	0	0	0
AC0181_C	0	0	0
AC0182_C	0	0	0
AC0183_C	0	0	0
AC0184_C	0	0	0
AC0185_C	0	0	0
AC0186_C	0	0	0
AC0187_C	0	0	0
AC0188_C	0	0	0
AC0189_C	0	0	0
AC0190_C	0	0	0
AC0191_C	0	0	0
AC0192_C	0	0	0
AC0193_C	0	0	0
AC0194_C	0	0	0
AC0195_C	0	0	0
AC0196_C	0	0	0
AC0197_C	0	0	0
AC0199_C	0	0	0
AC0200_C	0	0	0
AC0201_C	0	0	0
AC0202_C	0	0	0
AC0203_C	0	0	0
AG0082_C	0	0	0
AG0085_C	0	0	0

	Cyp6mz _Dup0	Cyp6m _Dup1	Cyp6z _Dup1
AG0089_C	0	0	0
AG0096_C	0	0	0
AG0097_C	0	0	0
AG0098_C	0	0	0
AG0100_C	0	0	0
AG0102_C	0	0	0
AG0104_C	0	0	0
AG0106_C	0	0	0
AG0108_C	0	0	0
AG0109_C	0	0	0
AG0111_C	0	0	0
AG0118_C	0	0	0
AG0120_C	0	0	0
AG0121_C	0	0	0
AG0123_C	0	0	0
AG0125_C	0	0	0
AG0126_C	0	0	0
AG0127_C	0	0	0
AG0128_C	0	0	0
AG0129_C	0	0	0
AG0133_C	0	0	0
AG0134_C	0	0	0
AG0136_C	0	0	0
AG0137_C	0	0	0
AG0138_C	0	0	0
AG0139_C	0	0	0
AG0141_C	0	0	0
AG0142_C	0	0	0
AG0143_C	0	0	0
AG0144_C	0	0	0
AG0145_C	0	0	0
AG0146_C	0	0	0
AG0147_C	0	0	0
AG0148_C	0	0	0
AG0152_C	0	0	0
AG0153_C	0	0	0
AG0156_C	0	0	0
AG0159_C	0	0	0
AG0162_C	0	0	0
AG0163_C	0	0	0
AG0169_C	0	0	0
AG0170_C	0	0	0
AG0172_C	0	0	0
AG0178_C	0	0	0
AG0179_C	0	0	0
AG0181_C	0	0	0
AG0183_C	0	0	0
AG0195_C	0	0	0
AG0197_C	0	0	0
AG0202_C	0	0	0
AG0203_C	0	0	0
AG0204_C	0	0	0
AG0206_C	0	0	0
AG0208_C	0	0	0
AG0214_C	0	0	0
AG0221_C	0	0	0
AG0223_C	0	0	0
AG0227_C	0	0	0
AG0229_C	0	0	0
AG0230_C	0	0	0
AG0231_C	0	0	0
AG0232_C	0	0	0
AG0263_C	0	0	0
AJ0023_C	0	0	0
AJ0024_C	0	0	0
AJ0028_C	0	0	0
AJ0032_C	0	0	0
AJ0035_C	0	0	0
AJ0036_C	0	0	0
AJ0037_C	0	0	0

	Cyp6mz _Dup0	Cyp6m _Dup1	Cyp6z _Dup1
AJ0038_C	0	0	0
AJ0039_C	0	0	0
AJ0043_C	0	0	0
AJ0044_C	0	0	0
AJ0045_C	0	0	0
AJ0047_C	0	0	0
AJ0051_C	0	0	0
AJ0052_C	0	0	0
AJ0056_C	0	0	0
AJ0059_C	0	0	0
AJ0060_C	0	0	0
AJ0061_C	0	0	0
AJ0063_C	0	0	0
AJ0064_C	0	0	0
AJ0066_C	0	0	0
AJ0068_C	0	0	0
AJ0070_C	0	0	0
AJ0071_C	0	0	0
AJ0072_C	0	0	0
AJ0074_C	0	0	0
AJ0075_C	0	0	0
AJ0076_C	0	0	0
AJ0077_C	0	0	0
AJ0078_C	0	0	0
AJ0080_C	0	0	0
AJ0081_C	0	0	0
AJ0084_C	0	0	0
AJ0085_C	0	0	0
AJ0086_C	0	0	0
AJ0087_C	0	0	0
AJ0088_C	0	0	0
AJ0090_C	0	0	0
AJ0092_C	0	0	0
AJ0093_C	0	0	0
AJ0095_C	0	0	0
AJ0096_C	0	0	0
AJ0097_C	0	0	0
AJ0098_C	0	0	0
AJ0099_C	0	0	0
AJ0100_C	0	0	0
AJ0101_C	0	0	0
AJ0102_C	0	0	0
AJ0103_C	0	0	0
AJ0105_C	0	0	0
AJ0107_C	0	0	0
AJ0109_C	0	0	0
AJ0113_C	0	0	0
AJ0115_C	0	0	0
AJ0116_C	0	0	0
AJ0117_C	0	0	0
AJ0119_C	0	0	0
AJ0128_C	0	0	0
AJ0129_C	0	0	0
AJ0130_C	0	0	0
AJ0131_C	0	0	0
AJ0132_C	0	0	0
AJ0133_C	0	0	0
AJ0134_C	0	0	0
AJ0135_C	0	0	0
AJ0136_C	0	0	0
AJ0137_C	0	0	0
AJ0138_C	0	0	0
AJ0139_C	0	0	0
AJ0140_C	0	0	0
AJ0141_C	0	0	0
AJ0142_C	0	0	0
AJ0143_C	0	0	0
AJ0144_C	0	0	0
AJ0145_C	0	0	0
AJ0146_C	0	0	0

	Cyp6mz _Dup0	Cyp6m _Dup1	Cyp6z _Dup1
AJ0147_C	0	0	0
AJ0148_C	0	0	0
AJ0149_C	0	0	0
AJ0150_C	0	0	0
AJ0151_C	0	0	0
AJ0152_C	0	0	0
AJ0153_C	0	0	0
AJ0154_C	0	0	0
AJ0155_C	0	0	0
AJ0156_C	0	0	0
AJ0157_C	0	0	0
AJ0158_C	0	0	0
AJ0159_C	0	0	0
AJ0161_C	0	0	0
AK0060_C	0	0	0
AK0062_C	0	0	0
AK0065_C	0	0	0
AK0066_C	0	0	0
AK0067_C	0	0	0
AK0068_C	0	0	0
AK0069_C	0	0	0
AK0070_C	0	0	0
AK0072_C	0	0	0
AK0073_C	0	0	0
AK0074_C	0	0	0
AK0075_C	0	0	0
AK0076_C	0	0	0
AK0077_C	0	0	0
AK0078_C	0	0	0
AK0079_C	0	0	0
AK0080_C	0	0	0
AK0081_C	0	0	0
AK0082_C	0	0	0
AK0085_C	0	0	0
AK0086_C	0	0	0
AK0087_C	0	0	0
AK0088_C	0	0	0
AK0089_C	0	0	0
AK0090_C	0	0	0
AK0091_C	0	0	0
AK0092_C	0	0	0
AK0093_C	0	0	0
AK0094_C	0	0	0
AK0095_C	0	0	0
AK0096_C	0	0	0
AK0098_C	0	0	0
AK0099_C	0	0	0
AK0100_C	0	0	0
AK0101_C	0	0	0
AK0102_C	0	0	0
AK0103_C	0	0	0
AK0104_C	0	0	0
AK0105_C	0	0	0
AK0106_C	0	0	0
AK0107_C	0	0	0
AK0108_C	0	0	0
AK0109_C	0	0	0
AK0110_C	0	0	0
AK0116_C	0	0	0
AK0117_C	0	0	0
AK0119_C	0	0	0
AK0127_C	0	0	0
AN0007_C	0	0	0
AN0008_C	0	0	0
AN0009_C	0	0	0
AN0010_C	0	0	0
AN0011_C	0	0	0
AN0012_C	0	0	0
AN0013_C	0	0	0
AN0014_C	0	0	0

	Cyp6mz _Dup0	Cyp6m _Dup1	Cyp6z _Dup1
AN0015_C	0	0	0
AN0016_C	0	0	0
AN0017_C	0	0	0
AN0018_C	0	0	0
AN0019_C	0	0	0
AN0020_C	0	0	0
AN0022_C	0	0	0
AN0023_C	0	0	0
AN0024_C	0	0	0
AN0025_C	0	0	0
AN0026_C	0	0	0
AN0027_C	0	0	0
AN0028_C	0	0	0
AN0029_C	0	0	0
AN0030_C	0	0	0
AN0031_C	0	0	0
AN0032_C	0	0	0
AN0033_C	0	0	0
AN0034_C	0	0	0
AN0035_C	0	0	0
AN0036_C	0	0	0
AN0037_C	0	0	0
AN0038_C	0	0	0
AN0039_C	0	0	0
AN0040_C	0	0	0
AN0041_C	0	0	0
AN0042_C	0	0	0
AN0043_C	0	0	0
AN0044_C	0	0	0
AN0045_C	0	0	0
AN0046_C	0	0	0
AN0047_C	0	0	0
AN0048_C	0	0	0
AN0049_C	0	0	0
AN0050_C	0	0	0
AN0051_C	0	0	0
AN0053_C	0	0	0
AN0054_C	0	0	0
AN0055_C	0	0	0
AN0056_C	0	0	0
AN0057_C	0	0	0
AN0058_C	0	0	0
AN0059_C	0	0	0
AN0060_C	0	0	0
AN0061_C	0	0	0
AN0062_C	0	0	0
AN0063_C	0	0	0
AN0064_C	0	0	0
AN0065_C	0	0	0
AN0066_C	0	0	0
AN0067_C	0	0	0
AN0068_C	0	0	0
AN0069_C	0	0	0
AN0070_C	0	0	0
AN0071_C	0	0	0
AN0072_C	0	0	0
AN0073_C	0	0	0
AN0074_C	0	0	0
AN0075_C	0	0	0
AN0076_C	0	0	0
AN0077_C	0	0	0
AN0079_C	0	0	0
AN0080_C	0	0	0
AN0081_C	0	0	0
AN0082_C	0	0	0
AN0083_C	0	0	0
AN0084_C	0	0	0
AN0085_C	0	0	0
AN0086_C	0	0	0
AN0087_C	0	0	0

	Cyp6mz _Dup0	Cyp6m _Dup1	Cyp6z _Dup1
AN0088_C	0	0	0
AN0089_C	0	0	0
AN0090_C	0	0	0
AN0091_C	0	0	0
AN0092_C	0	0	0
AN0093_C	0	0	0
AN0094_C	0	0	0
AN0095_C	0	0	0
AN0096_C	0	0	0
AN0097_C	0	0	0
AN0098_C	0	0	0
AN0099_C	0	0	0
AN0100_C	0	0	0
AN0101_C	0	0	0
AN0102_C	0	0	0
AN0103_C	0	0	0
AN0104_C	0	0	0
AN0105_C	0	0	0
AN0106_C	0	0	0
AN0107_C	0	0	0
AN0108_C	0	0	0
AN0109_C	0	0	0
AN0111_C	0	0	0
AN0112_C	0	0	0
AN0113_C	0	0	0
AN0114_C	0	0	0
AN0115_C	0	0	0
AN0117_C	0	0	0
AN0118_C	0	0	0
AN0119_C	0	0	0
AN0120_C	0	0	0
AN0121_C	0	0	0
AN0122_C	0	0	0
AN0123_C	0	0	0
AN0124_C	0	0	0
AN0125_C	0	0	0
AN0126_C	0	0	0
AN0127_C	0	0	0
AN0128_C	0	0	0
AN0129_C	0	0	0
AN0130_C	0	0	0
AN0131_C	0	0	0
AN0132_C	0	0	0
AN0134_C	0	0	0
AN0135_C	0	0	0
AN0136_C	0	0	0
AN0137_C	0	0	0
AN0138_C	0	0	0
AN0139_C	0	0	0
AN0140_C	0	0	0
AN0141_C	0	0	0
AN0142_C	0	0	0
AN0143_C	0	0	0
AN0144_C	0	0	0
AN0147_C	0	0	0
AN0149_C	0	0	0
AN0151_C	0	0	0
AN0152_C	0	0	0
AN0153_C	0	0	0
AN0154_C	0	0	0
AN0155_C	0	0	0
AN0156_C	0	0	0
AN0157_C	0	0	0
AN0158_C	0	0	0
AN0159_C	0	0	0
AN0160_C	0	0	0
AN0162_C	0	0	0
AN0163_C	0	0	0
AN0164_C	0	0	0
AN0165_C	0	0	0

	Cyp6mz _Dup0	Cyp6m _Dup1	Cyp6z _Dup1
AN0166_C	0	0	0
AN0167_C	0	0	0
AN0168_C	0	0	0
AN0169_C	0	0	0
AN0170_C	0	0	0
AN0171_C	0	0	0
AN0172_C	0	0	0
AN0173_C	0	0	0
AN0174_C	0	0	0
AN0175_C	0	0	0
AN0176_C	0	0	0
AN0177_C	0	0	0
AN0178_C	0	0	0
AN0179_C	0	0	0
AN0180_C	0	0	0
AN0181_C	0	0	0
AN0182_C	0	0	0
AN0183_C	0	0	0
AN0184_C	0	0	0
AN0185_C	0	0	0
AN0186_C	0	0	0
AN0187_C	0	0	0
AN0188_C	0	0	0
AN0189_C	0	0	0
AN0190_C	0	0	0
AN0191_C	0	0	0
AN0192_C	0	0	0
AN0193_C	0	0	0
AN0194_C	0	0	0
AN0196_C	0	0	0
AN0197_C	0	0	0
AN0198_C	0	0	0
AN0199_C	0	0	0
AN0200_C	0	0	0
AN0201_C	0	0	0
AN0202_C	0	0	0
AN0203_C	0	0	0
AN0204_C	0	0	0
AN0205_C	0	0	0
AN0206_C	0	0	0
AN0207_C	0	0	0
AN0208_C	0	0	0
AN0209_C	0	0	0
AN0210_C	0	0	0
AN0212_C	0	0	0
AN0213_C	0	0	0
AN0214_C	0	0	0
AN0215_C	0	0	0
AN0217_C	0	0	0
AN0218_C	0	0	0
AN0219_C	0	0	0
AN0220_C	0	0	0
AN0221_C	0	0	0
AN0222_C	0	0	0
AN0223_C	0	0	0
AN0224_C	0	0	0
AN0225_C	0	0	0
AN0226_C	0	0	0
AN0227_C	0	0	0
AN0228_C	0	0	0
AN0229_C	0	0	0
AN0230_C	0	0	0
AN0231_C	0	0	0
AN0232_C	0	0	0
AN0233_C	0	0	0
AN0234_C	0	0	0
AN0235_C	0	0	0
AN0236_C	0	0	0
AN0237_C	0	0	0
AN0238_C	0	0	0

	Cyp6mz _Dup0	Cyp6m _Dup1	Cyp6z _Dup1
AN0239_C	0	0	0
AN0240_C	0	0	0
AN0241_C	0	0	0
AN0242_C	0	0	0
AN0243_C	0	0	0
AN0244_C	0	0	0
AN0245_C	0	0	0
AN0246_C	0	0	0
AN0247_C	0	0	0
AN0248_C	0	0	0
AN0250_C	0	0	0
AN0251_C	0	0	0
AN0252_C	0	0	0
AN0253_C	0	0	0
AN0254_C	0	0	0
AN0255_C	0	0	0
AN0256_C	0	0	0
AN0258_C	0	0	0
AN0259_C	0	0	0
AN0260_C	0	0	0
AN0261_C	0	0	0
AN0262_C	0	0	0
AN0263_C	0	0	0
AN0264_C	0	0	0
AN0265_C	0	0	0
AN0266_C	0	0	0
AN0267_C	0	0	0
AN0268_C	0	0	0
AN0269_C	0	0	0
AN0270_C	0	0	0
AN0271_C	0	0	0
AN0272_C	0	0	0
AN0275_C	0	0	0
AN0276_C	0	0	0
AN0277_C	0	0	0
AN0278_C	0	0	0
AN0279_C	0	0	0
AN0280_C	0	0	0
AN0281_C	0	0	0
AN0282_C	0	0	0
AN0283_C	0	0	0
AN0284_C	0	0	0
AN0285_C	0	0	0
AN0286_C	0	0	0
AN0287_C	0	0	0
AN0288_C	0	0	0
AN0289_C	0	0	0
AN0290_C	0	0	0
AN0291_C	0	0	0
AN0292_C	0	0	0
AN0293_C	0	0	0
AN0294_C	0	0	0
AN0295_C	0	0	0
AN0296_C	0	0	0
AN0297_C	0	0	0
AN0298_C	0	0	0
AN0299_C	0	0	0
AN0300_C	0	0	0
AN0301_C	0	0	0
AN0302_C	0	0	0
AN0303_C	0	0	0
AN0304_C	0	0	0
AN0305_C	0	0	0
AN0306_C	0	0	0
AN0307_C	0	0	0
AN0308_C	0	0	0
AN0309_C	0	0	0
AN0310_C	0	0	0
AN0311_C	0	0	0
AN0312_C	0	0	0

	Cyp6mz _Dup0	Cyp6m _Dup1	Cyp6z _Dup1
AN0313_C	0	0	0
AN0314_C	0	0	0
AN0315_C	0	0	0
AN0316_C	0	0	0
AN0317_C	0	0	0
AN0318_C	0	0	0
AN0319_C	0	0	0
AN0320_C	0	0	0
AN0321_C	0	0	0
AP0002_C	0	0	0
AP0005_C	0	0	0
AP0006_C	0	0	0
AP0007_C	0	0	0
AP0008_C	0	0	0
AP0009_C	0	0	0
AP0010_C	0	0	0
AP0011_C	0	0	0
AP0014_C	0	0	0
AP0017_C	0	0	0
AP0018_C	0	0	0
AP0019_C	0	0	0
AP0020_C	0	0	0
AP0021_C	0	0	0
AP0022_C	0	0	0
AP0023_C	0	0	0
AP0024_C	0	0	0
AP0025_C	0	0	0
AP0030_C	0	0	0
AP0031_C	0	0	0
AP0032_C	0	0	0
AP0033_C	0	0	0
AP0034_C	0	0	0
AP0035_C	0	0	0
AQ0001_C	0	0	0
AQ0002_C	0	0	0
AQ0004_C	0	0	0
AQ0005_C	0	0	0
AQ0011_C	0	0	0
AQ0012_C	0	0	0
AQ0013_C	0	0	0
AQ0014_C	0	0	0
AQ0015_C	0	0	0
AR0001_C	0	0	0
AR0007_C	0	0	0
AR0008_C	0	0	0
AR0009_C	0	0	0
AR0010_C	0	0	0
AR0011_C	0	0	0
AR0012_C	0	0	0
AR0013_C	0	0	0
AR0014_C	0	0	0
AR0015_C	0	0	0
AR0016_C	0	0	0
AR0017_C	0	0	0
AR0018_C	0	0	0
AR0019_C	0	0	0
AR0020_C	0	0	0
AR0021_C	0	0	0
AR0022_C	0	0	0
AR0023_C	0	0	0
AR0024_C	0	0	0
AR0026_C	0	0	0
AR0027_C	0	0	0
AR0034_C	0	0	0
AR0035_C	0	0	0
AR0036_C	0	0	0
AR0038_C	0	0	0
AR0040_C	0	0	0
AR0042_C	0	0	0
AR0043_C	0	0	0

	Cyp6mz _Dup0	Cyp6m _Dup1	Cyp6z _Dup1
AR0044_C	0	0	0
AR0045_C	0	0	0
AR0046_C	0	0	0
AR0047_C	0	0	0
AR0048_C	0	0	0
AR0049_C	0	0	0
AR0050_C	0	0	0
AR0051_C	0	0	0
AR0052_C	0	0	0
AR0053_C	0	0	0
AR0054_C	0	0	0
AR0057_C	0	0	0
AR0059_C	0	0	0
AR0060_C	0	0	0
AR0061_C	0	0	0
AR0062_C	0	0	0
AR0063_C	0	0	0
AR0064_C	0	0	0
AR0065_C	0	0	0
AR0066_C	0	0	0
AR0069_C	0	0	0
AR0070_C	0	0	0
AR0071_C	0	0	0
AR0072_C	0	0	0
AR0073_C	0	0	0
AR0074_C	0	0	0
AR0075_C	0	0	0
AR0076_C	0	0	0
AR0077_C	0	0	0
AR0078_C	0	0	0
AR0079_C	0	0	0
AR0080_C	0	0	0
AR0081_C	0	0	0
AR0082_C	0	0	0
AR0083_C	0	0	0
AR0084_C	0	0	0
AR0085_C	0	0	0
AR0086_C	0	0	0
AR0087_C	0	0	0
AR0088_C	0	0	0
AR0089_C	0	0	0
AR0090_C	0	0	0
AR0092_C	0	0	0
AR0093_C	0	0	0
AR0095_C	0	0	0
AR0096_C	0	0	0
AR0097_C	0	0	0
AR0098_C	0	0	0
AR0099_C	0	0	0
AR0100_C	0	0	0
AS0001_C	0	0	0
AS0002_C	0	0	0
AS0003_C	0	0	0
AS0004_C	0	0	0
AS0005_C	0	0	0
AS0006_C	0	0	0
AS0007_C	0	0	0
AS0008_C	0	0	0
AS0009_C	0	0	0
AS0010_C	0	0	0
AS0011_C	0	0	0
AS0012_C	0	0	0
AS0013_C	0	0	0
AS0014_C	0	0	0
AS0015_C	0	0	0
AS0016_C	0	0	0
AS0017_C	0	0	0
AS0018_C	0	0	0
AS0019_C	0	0	0
AS0020_C	0	0	0

	Cyp6mz _Dup0	Cyp6m _Dup1	Cyp6z _Dup1
AS0021_C	0	0	0
AS0022_C	0	0	0
AS0024_C	0	0	0
AS0025_C	0	0	0
AS0026_C	0	0	0
AS0027_C	0	0	0
AS0028_C	0	0	0
AS0029_C	0	0	0
AS0030_C	0	0	0
AS0032_C	0	0	0
AS0033_C	0	0	0
AS0034_C	0	0	0
AS0035_C	0	0	0
AS0036_C	0	0	0
AS0037_C	0	0	0
AS0039_C	0	0	0
AS0040_C	0	0	0
AS0041_C	0	0	0
AS0042_C	0	0	0
AS0044_C	0	0	0
AS0045_C	0	0	0
AS0046_C	0	0	0
AS0047_C	0	0	0
AS0048_C	0	0	0
AS0049_C	0	0	0
AS0051_C	0	0	0
AS0052_C	0	0	0
AS0053_C	0	0	0
AS0054_C	0	0	0
AS0055_C	0	0	0
AS0056_C	0	0	0
AS0057_C	0	0	0
AS0058_C	0	0	0
AS0059_C	0	0	0
AS0060_C	0	0	0
AS0062_C	0	0	0
AS0064_C	0	0	0
AS0065_C	0	0	0
AS0066_C	0	0	0
AS0068_C	0	0	0
AS0069_C	0	0	0
AS0070_C	0	0	0
AS0071_C	0	0	0
AS0072_C	0	0	0
AS0073_C	0	0	0
AS0074_C	0	0	0
AS0076_C	0	0	0
AS0077_C	0	0	0
AS0078_C	0	0	0
AV0001_C	0	0	0
AV0002_C	0	1	0
AV0003_C	0	1	0
AV0004_C	0	0	0
AV0005_C	0	0	0
AV0006_C	0	0	0
AV0007_C	0	0	0
AV0008_C	0	0	0
AV0009_C	0	0	0
AV0010_C	0	0	0
AV0011_C	0	0	0
AV0012_C	0	0	0
AV0013_C	0	0	0
AV0014_C	0	1	0
AV0015_C	0	1	0
AV0017_C	0	0	0
AV0018_C	0	0	0
AV0020_C	0	0	0
AV0021_C	0	0	0
AV0022_C	0	0	0
AV0024_C	0	0	0

	Cyp6mz _Dup0	Cyp6m _Dup1	Cyp6z _Dup1
AV0025_C	0	0	0
AV0026_C	0	0	0
AV0027_C	0	0	0
AV0028_C	0	0	0
AV0029_C	0	0	0
AV0030_C	0	0	0
AV0031_C	0	0	0
AV0032_C	0	0	0
AV0033_C	0	0	0
AV0034_C	0	0	0
AV0035_C	0	0	0
AV0036_C	0	0	0
AV0037_C	0	0	0
AV0038_C	0	0	0
AV0039_C	0	0	0
AV0040_C	0	0	0
AV0041_C	0	0	0
AV0042_C	0	0	0
AV0043_C	0	0	0
AV0044_C	0	0	0
AV0045_C	0	0	0
AV0046_C	0	0	0
AV0047_C	0	1	0
AY0006_C	0	0	0
AY0007_C	0	0	0
AY0010_C	0	0	0
AY0011_C	0	0	0
AY0012_C	0	0	0
AY0013_C	0	0	0
AY0015_C	0	0	0
AY0016_C	0	0	0
AY0017_C	0	0	2
AY0018_C	0	0	0
AY0019_C	0	0	0
AY0020_C	0	0	0
AY0021_C	0	0	0
AY0023_C	0	0	0
AY0024_C	0	0	0
AY0025_C	0	0	0
AY0026_C	0	0	0
AY0027_C	0	0	0
AY0029_C	0	0	0
AY0031_C	0	0	0
AY0032_C	0	0	0
AY0033_C	0	0	0
AY0034_C	0	0	0
AY0035_C	0	0	0
AY0036_C	0	0	0
AY0038_C	0	0	0
AY0039_C	0	0	2
AY0040_C	0	0	0
AY0041_C	0	0	0
AY0042_C	0	0	0
AY0043_C	0	0	0
AY0045_C	0	0	0
AY0046_C	0	0	0
AY0047_C	0	0	0
AY0048_C	0	0	0
AY0049_C	0	0	0
AY0050_C	0	0	0
AY0052_C	0	0	0
AY0053_C	0	0	2
AY0054_C	0	0	0
AY0055_C	0	0	0
AY0056_C	0	0	0
AY0057_C	0	0	0
AY0058_C	0	0	0
AY0059_C	0	0	0
AY0060_C	0	0	2
AY0061_C	0	0	0

	Cyp6mz _Dup0	Cyp6m _Dup1	Cyp6z _Dup1
AY0062_C	0	0	0
AY0063_C	0	0	0
AY0064_C	0	0	0
AY0065_C	0	0	0
AY0066_C	0	0	0
AY0067_C	0	0	0
AY0068_C	0	0	0
AY0069_C	0	0	2
AY0070_C	0	0	0
AY0072_C	0	0	0
AY0074_C	0	0	0
AY0076_C	0	0	0
AY0077_C	0	0	0
AY0078_C	0	0	0
AY0079_C	0	0	0
AY0080_C	0	0	0
AY0082_C	0	0	0
AY0083_C	0	0	0
AY0085_C	0	0	0
AY0087_C	0	0	0
AY0088_C	0	0	0
AY0089_C	0	0	0
AY0090_C	0	0	0
AY0091_C	0	0	0
