# Supplementary material for: Whole-genome sequencing reveals high complexity of copy number variation at insecticide resistance loci in malaria mosquitoes
Source: Genome Res. 2019 Aug;29(8):1250–61. doi: 10.1101/gr.245795.118 (PMC6673711; doi:10.1101/gr.245795.118)
Supplement: Supplemental Material [file supp_gr.245795.118_Supplementary_Data_S7.pdf]

Electronic Supplementary Material S7
Description of duplication types detected in the CYP9K1 region in Ag1000G phase 2.

Overview of all duplications in the CYP9K1 region.

Sixteen CNV alleles were found that could be categorised according to their footprint of discordant reads or reads mapping to the CNV breakpoints. The regions covered by these CNVs are shown in Fig. **9K1_S1**.

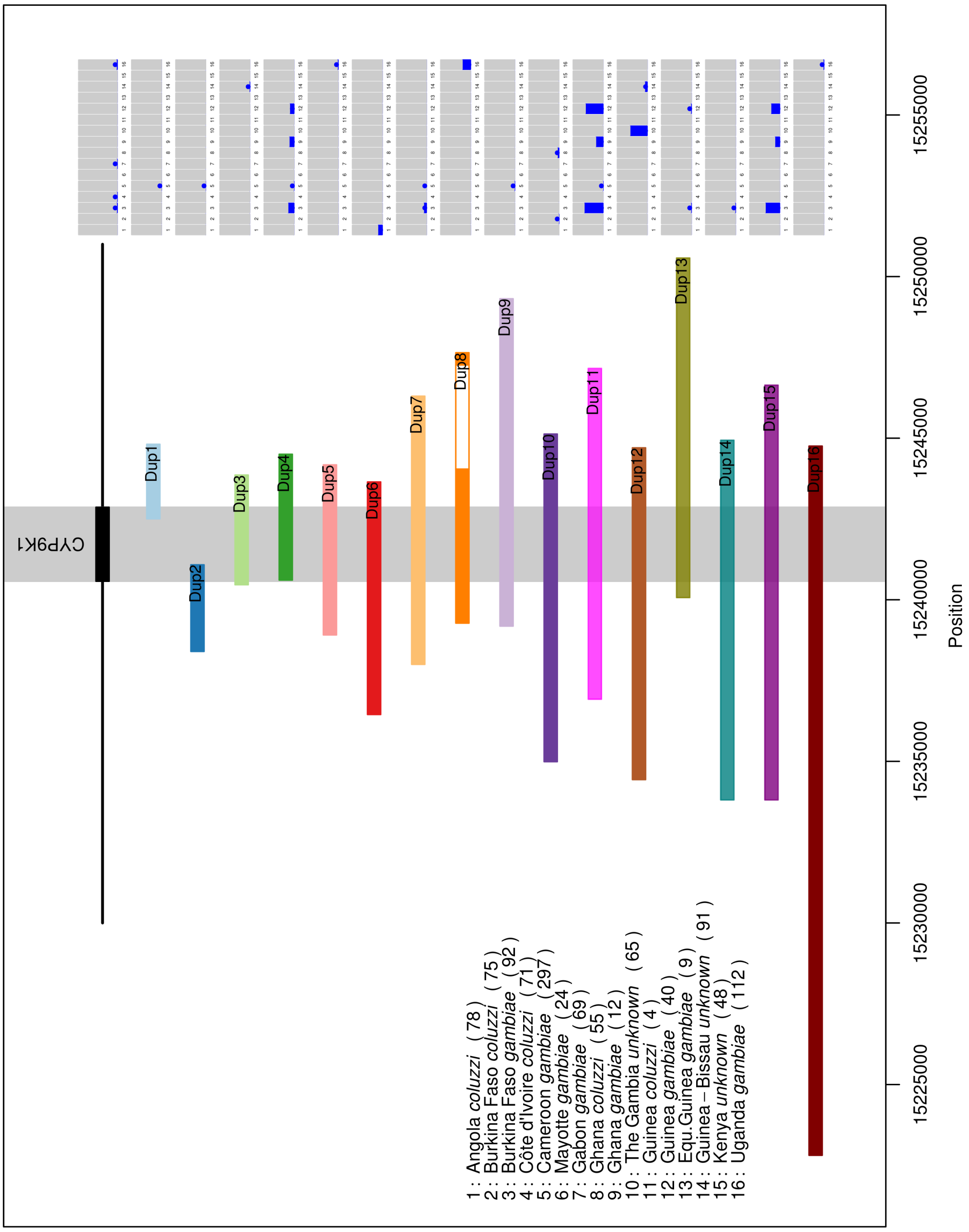

Fig. 9K1_S1: Overview of the CNVs on or around CYP9K1 present in Phase 2 of the Ag1000G dataset. Position on chromosome X is shown on the X axis. Barplots on the right show the proportion of samples that carry a given CNV in each of the Phase 2 populations. Numbers below the barplots are numeric population IDs detailed on the far left (numbers in brackets indicate the total number of samples from that population). Blue points on the barplots indicate that at least one sample in this population carried the CNV. The top barplot (Cyp9k1_Dup0) shows duplications that could not be categorised, each subsequent barplot represents the CNV shown to its left (Cyp9k1_Dup1 - Dup16). The white box inside Cyp9k1_Dup8 indicates the region of that duplication that has undergone subsequent deletion. The extent of Cyp9k1_Dup7, Cyp9k1_Dup14 and Cyp9k1_Dup15 could not be accurately determine and may thus be incorrect.

Notes

CYP9K1 spans the range 15240572 - 15242864 on Chromosome X. The region 5' of position 15235000 has very variable coverage (eg: Fig. **9K1_S3**) and is thus impossible to interpret as far as CNVs are concerned. Most of the CNVs we detected don't involve that variable region, but a few do, and the CNVs that couldn't be described may well involve that region.

While 16 CNV alleles are described below, there remain 7 samples that carry CNVs that don't fit any of the descriptions below (Cyp9k1_Dup0 in Fig. **9K1_S1**). Whether these belong to other duplication types or fit into the classification below but could not be called with the available data is not known.

Duplication type 1

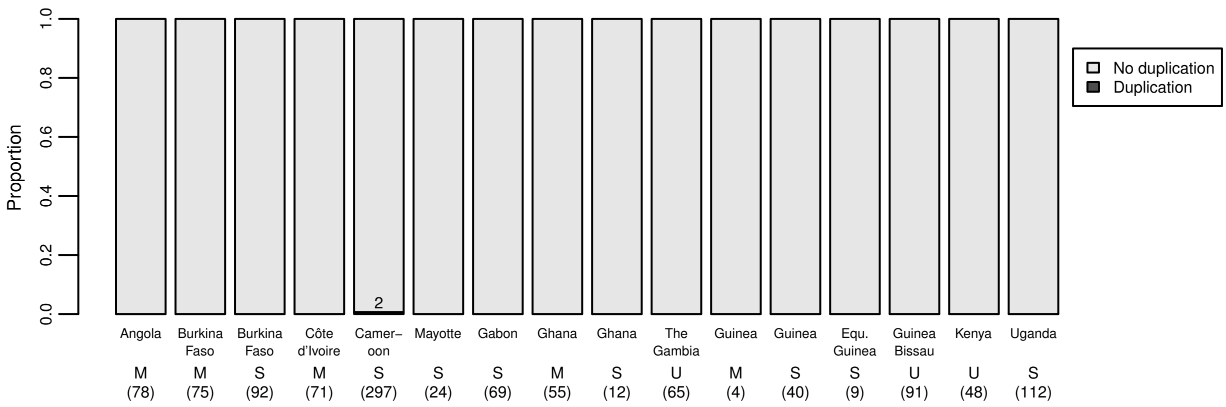

Fig. 9K1_S2: Barplot showing the proportion of samples that carry the Cyp9k1_Dup1 duplication in each of the Phase 2 populations. Numbers above the dark grey bars indicate the absolute number of samples carrying the duplication. S = *Anopheles gambiae*, M = *Anopheles coluzzi*, U = species undetermined. Numbers in brackets indicate the total number of samples from that population.

Cyp9k1_Dup1 was supported by face-away read pairs whose forward-facing read mapped in the interval 15242500 - 15242800 and whose reverse-facing read mapped in the interval 15244500 - 15244800 (Fig. **9K1_S3**). Cyp9k1_Dup1 was also supported by reads soft-clipped at the breakpoints (positions 15242505 and 15244812), with the clipped bases at each breakpoint aligning at the other breakpoint. Cyp9k1_Dup1 did not cover the whole of CYP9K1 (Fig. **9K1_S1**). The start point of this duplication is near the end of the second (and last) exon.

Cyp9k1_Dup1 breakpoint:

```
CATCGCTTTGCCGTTTG   CATATCGCTCAGCACATC
 end of the dup ^   ^ start of the dup
position 15244811   position 15242506
```

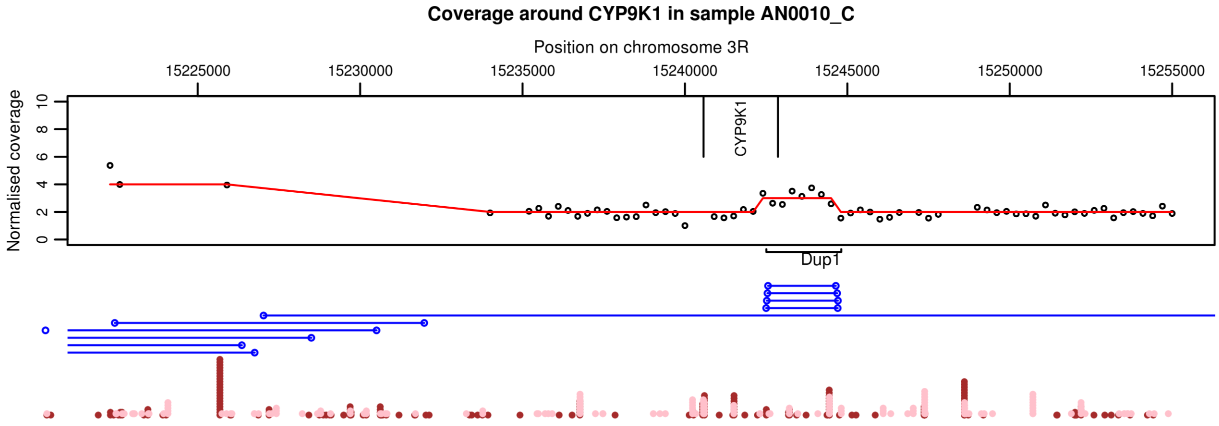

Fig. 9K1_S3: Example of coverage in an individual carrying the Cyp9k1_Dup1 duplication. Open black circles indicate coverage at each position. The red line shows the HMM estimation of the coverage state at each position. The HMM model does not detect the duplication, but the presence of the face-away reads coincides with a visible local increase in coverage. Black vertical lines represent the positions of the CYP9K1 gene. Pairs of blue points connected by lines indicate pairs of face-away reads. Reads soft-clipped before the alignment start point (dark brown points) and after the alignment end point (light brown points) are present at the start and end points of the duplication (clipped at positions 15242505 and 15244812 respectively). In each case, the clipped bases align to the other end of the duplication, as expected.

Both samples that carry Cyp9k1_Dup1 are female and have a copy number of 1 (Tables 9K1_S1.1 & 1.2). Cyp9k1_Dup1 is therefore a single copy duplication and the samples are heterozygous.

Table 9K1_1.1: Coverage calls for Cyp9k1_Dup1. NAs were produced if coverage was too variable or if the duplication completely overlapped with another duplication whose coverage could also not be called.

copy number	AO col	BF col	BF gam	CI col	CM gam	FR gam	GA gam	GH col	GH gam	GM	GN col	GN gam	GQ gam	GW	KE	UG gam
0	78	75	92	71	295	24	69	55	12	65	4	40	9	91	48	112
1	0	0	0	0	2	0	0	0	0	0	0	0	0	0	0	0

Table 9K1_1.2: Coverage calls for all duplications in individuals that carry Cyp9k1_Dup1. Male samples are indicated in red.

	Dup 0	Dup 1	Dup 2	Dup 3	Dup 4	Dup 5	Dup 6	Dup 7	Dup 8	Dup 9	Dup 10	Dup 11	Dup 12	Dup 13	Dup 14	Dup 15	Dup 16
AN0010_C	0	1	0	0	0	0	0	0	0	0	0	0	0	0	0	0	0
AN0283_C	0	1	0	0	0	0	0	0	0	0	0	0	0	0	0	0	0

Duplication type 2

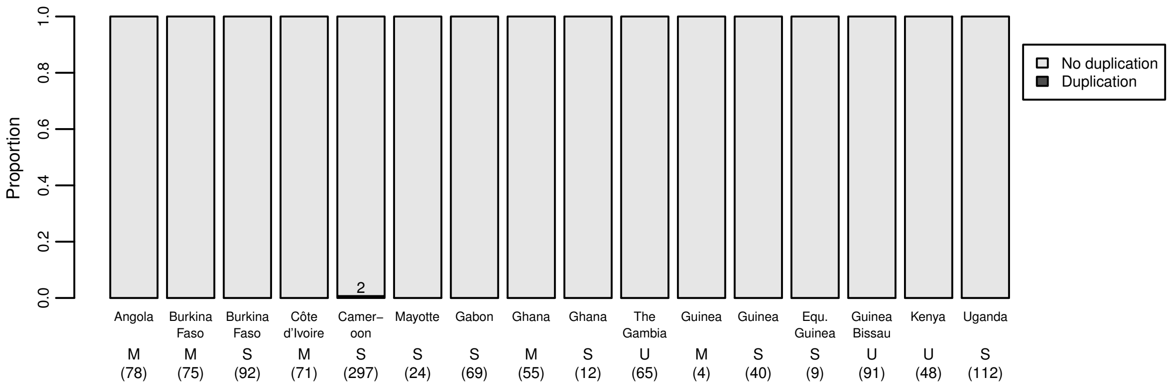

Fig. 9K1_S4: Barplot showing the proportion of samples that carry the Cyp9k1_Dup2 duplication in each of the Phase 2 populations. Numbers above the dark grey bars indicate the absolute number of samples carrying the duplication. S = *Anopheles gambiae*, M = *Anopheles coluzzi*, U = species undetermined. Numbers in brackets indicate the total number of samples from that population.

Cyp9k1_Dup2 was supported by face-away read pairs whose forward-facing read mapped in the interval 15238300 - 15238600 and whose reverse-facing read mapped in the interval 15240800 - 15241100 (Fig. **9K1_S5**). Cyp9k1_Dup2 was also supported by reads soft-clipped at the breakpoints (positions 15238400 and 15241082), with the clipped bases at each breakpoint aligning at the other breakpoint.Cyp9k1_Dup2 did not cover the whole of CYP9K1 (Fig. **9K1_S1**). The end point of this duplication is about half way through the first exon.

Cyp9k1_Dup2 breakpoint:

```
GTCGCGCATGATGGCCGCGGCCTGTATCCAC CGGTAGCATGATGGCCGCGGCC GGTAGCCGCTTACTGTTGGT
                 end of the dup ^      inserted seq      ^ start of the dup
               position 15241081                         position 15238401
```

The sequence `CGGTAGCATGATGGCCGCGGCC` is inserted between the sequences on either side of the breakpoint. Interestingly most of this insertion is a duplication of the sequence a few bases left of the breakpoint.

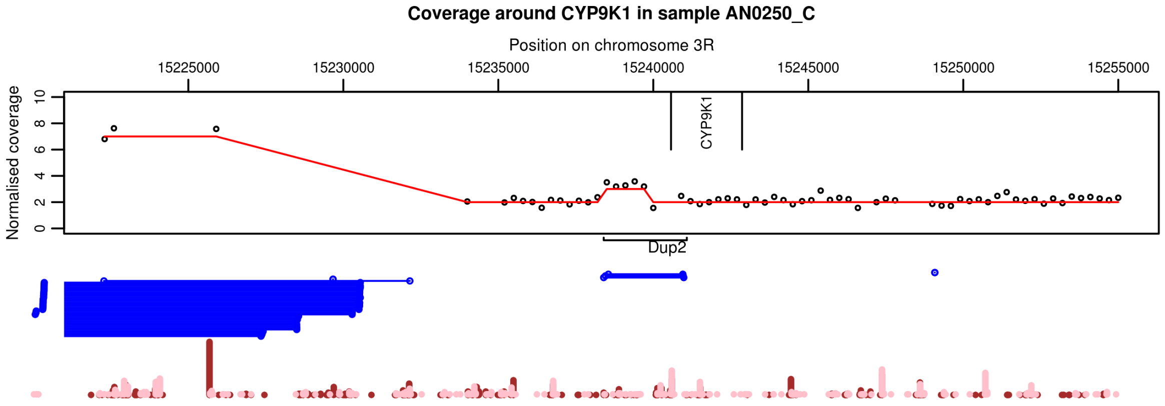

Fig. 9K1_S5: Example of coverage in an individual carrying the Cyp9k1_Dup2 duplication. Open black circles indicate coverage at each position. The red line shows the HMM estimation of the coverage state at each position. Black vertical lines represent the positions of the CYP9K1 gene. Pairs of blue points connected by lines indicate pairs of face-away reads. Reads soft-clipped before the alignment start point (dark brown points) are present at the start point of the duplication (clipped at position 15238400). Light brown points indicate reads soft-clipped after the alignment end point and are present at the end point of the duplication in a different sample (clipped at position 15241082). In each case, the clipped bases align to the other end of the duplication, as expected.

Both samples that carry Cyp9k1_Dup2 are female and have a copy number of 1 (Tables 9K1_S2.1 & 2.2). Cyp9k1_Dup2 is therefore a single copy duplication and the samples are heterozygous.

Table 9K1_2.1: Coverage calls for Cyp9k1_Dup2. NAs were produced if coverage was too variable or if the duplication completely overlapped with another duplication whose coverage could also not be called.

copy number	AO col	BF col	BF gam	CI col	CM gam	FR gam	GA gam	GH col	GH gam	GM	GN col	GN gam	GQ gam	GW	KE	UG gam
0	78	75	92	71	295	24	69	55	12	65	4	40	9	91	48	112
1	0	0	0	0	2	0	0	0	0	0	0	0	0	0	0	0

Table 9K1_2.2: Coverage calls for all duplications in individuals that carry Cyp9k1_Dup2. Male samples are indicated in red.

	Dup 0	Dup 1	Dup 2	Dup 3	Dup 4	Dup 5	Dup 6	Dup 7	Dup 8	Dup 9	Dup 10	Dup 11	Dup 12	Dup 13	Dup 14	Dup 15	Dup 16
AN0250_C	0	0	1	0	0	0	0	0	0	0	0	0	0	0	0	0	0
AN0280_C	0	0	1	0	0	0	0	0	0	0	0	0	0	0	0	0	0

Duplication type 3

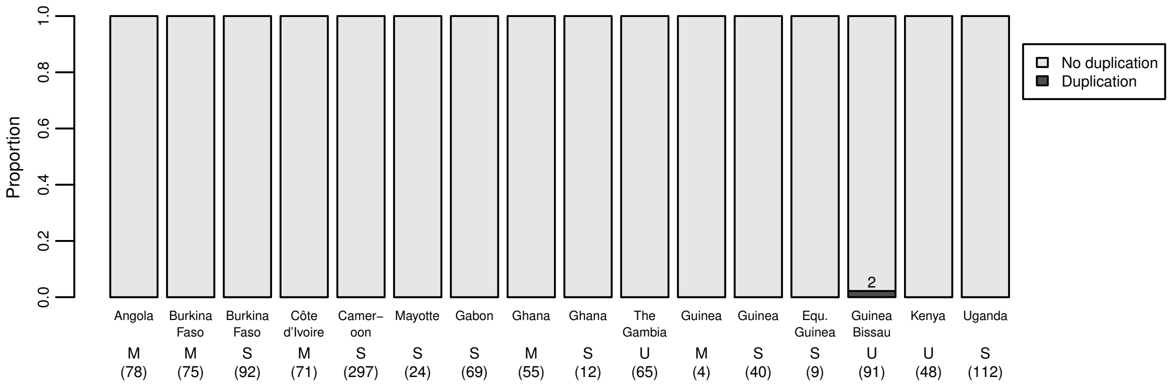

Fig. 9K1_S6: Barplot showing the proportion of samples that carry the Cyp9k1_Dup3 duplication in each of the Phase 2 populations. Numbers above the dark grey bars indicate the absolute number of samples carrying the duplication. S = *Anopheles gambiae*, M = *Anopheles coluzzi*, U = species undetermined. Numbers in brackets indicate the total number of samples from that population.

Cyp9k1_Dup3 was supported by face-away read pairs whose forward-facing read mapped in the interval 15240300 - 15240600 and whose reverse-facing read mapped in the interval 15243450 - 15243750 (Fig. 9K1_S7). Cyp9k1_Dup3 was also supported by reads soft-clipped at the end of the duplication (position 15243860), with the clipped bases aligning at 15240464. No reads were found that were soft-clipped at position 15240464, but this may be because the reads that spanned the breakpoint mapped more easily at the beginning than at the end.

Cyp9k1_Dup3 breakpoint:

```
AGGAAACTAATATATGTATTTA         T        GAACTAAATCACCGGTTAGG
         end of the dup ^   inserted seq   ^ start of the dup
      position 15243859                    position 15240464
```

The T is inserted between the sequences on either side of the breakpoint.

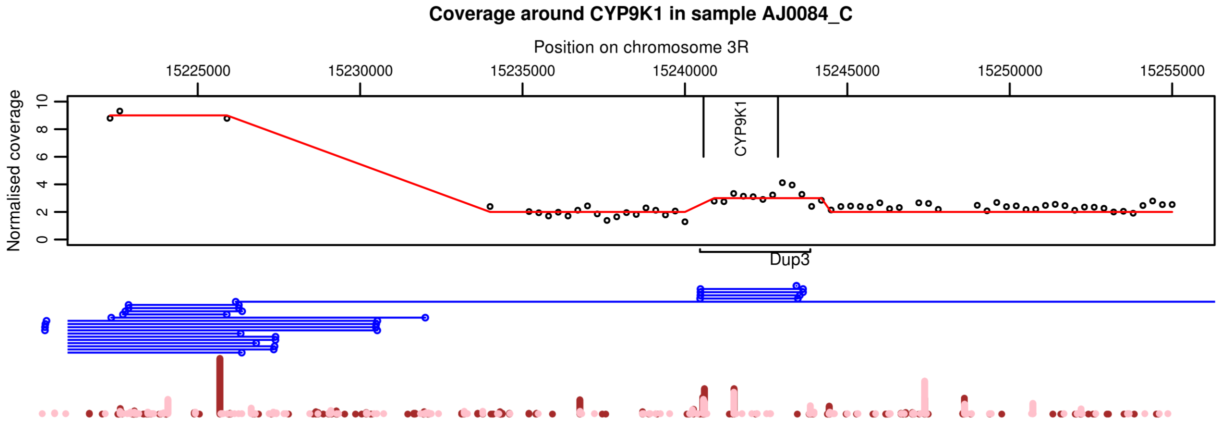

Fig. 9K1_S7: Example of coverage in an individual carrying the Cyp9k1_Dup3 duplication. Open black circles indicate coverage at each position. The red line shows the HMM estimation of the coverage state at each position. The HMM model does not detect the duplication, but the presence of the face-away reads coincides with a visible local increase in coverage. Black vertical lines represent the positions of the CYP9K1 gene. Pairs of blue points connected by lines indicate pairs of face-away reads. Reads soft-clipped after the alignment end point (light brown points) are present at the end point of the duplication (clipped at position 15243860). Dark brown points indicate reads soft-clipped before the alignment start point.

Both samples that carry Cyp9k1_Dup3 are female and have a copy number of 1 (Tables 9K1_S3.1 & 3.2). Cyp9k1_Dup3 is therefore a single copy duplication and the samples are heterozygous.

Table 9K1_3.1: Coverage calls for Cyp9k1_Dup3. NAs were produced if coverage was too variable or if the duplication completely overlapped with another duplication whose coverage could also not be called.

copy number	AO col	BF col	BF gam	CI col	CM gam	FR gam	GA gam	GH col	GH gam	GM	GN col	GN gam	GQ gam	GW	KE	UG gam
0	78	75	92	71	297	24	69	55	12	65	4	40	9	89	48	112
1	0	0	0	0	0	0	0	0	0	0	0	0	0	2	0	0

Table 9K1_3.2: Coverage calls for all duplications in individuals that carry Cyp9k1_Dup3. Male samples are indicated in red.

	Dup 0	Dup 1	Dup 2	Dup 3	Dup 4	Dup 5	Dup 6	Dup 7	Dup 8	Dup 9	Dup 10	Dup 11	Dup 12	Dup 13	Dup 14	Dup 15	Dup 16
AJ0059_C	0	0	0	1	0	0	0	0	0	0	0	0	0	0	0	0	0
AJ0084_C	0	0	0	1	0	0	0	0	0	0	0	0	0	0	0	0	0

Duplication type 4

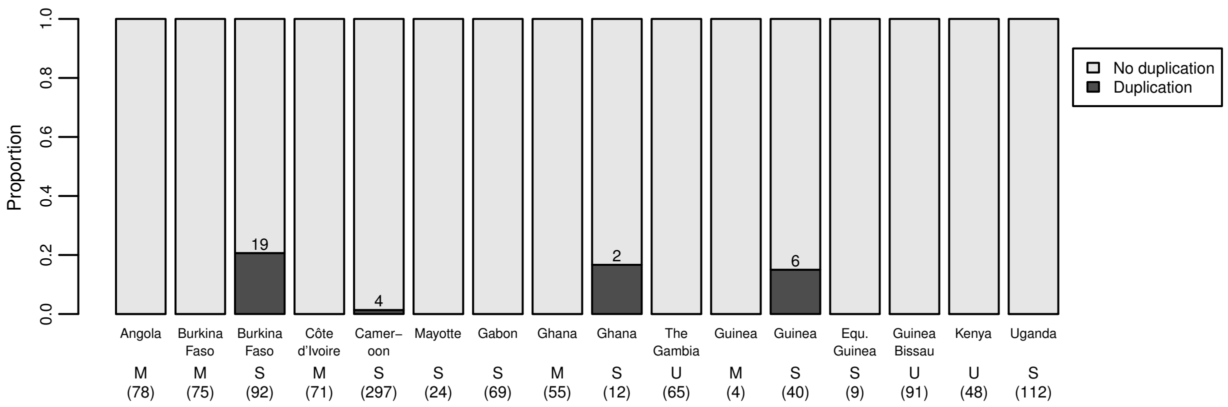

Fig. 9K1_S8: Barplot showing the proportion of samples that carry the Cyp9k1_Dup4 duplication in each of the Phase 2 populations. Numbers above the dark grey bars indicate the absolute number of samples carrying the duplication. S = *Anopheles gambiae*, M = *Anopheles coluzzi*, U = species undetermined. Numbers in brackets indicate the total number of samples from that population.

Cyp9k1_Dup4 was supported by face-away read pairs whose forward-facing read mapped in the interval 15240600 - 15240900 and whose reverse-facing read mapped in the interval 15244200 - 15244500 (Fig. 9K1_S9). Cyp9k1_Dup4 was also supported by reads soft-clipped at the breakpoints (positions 15240608 and 15244503), with the clipped bases at each breakpoint aligning at the other breakpoint.

Cyp9k1_Dup4 breakpoint:

```
GTTATTAAGAATGTGAAAT         A         CTGGACACTAAACGCTCAA
   end of the dup ^   inserted seq    ^ start of the dup
   position 15244502                  position 15240609
```

The A is inserted between the sequences on either side of the breakpoint.

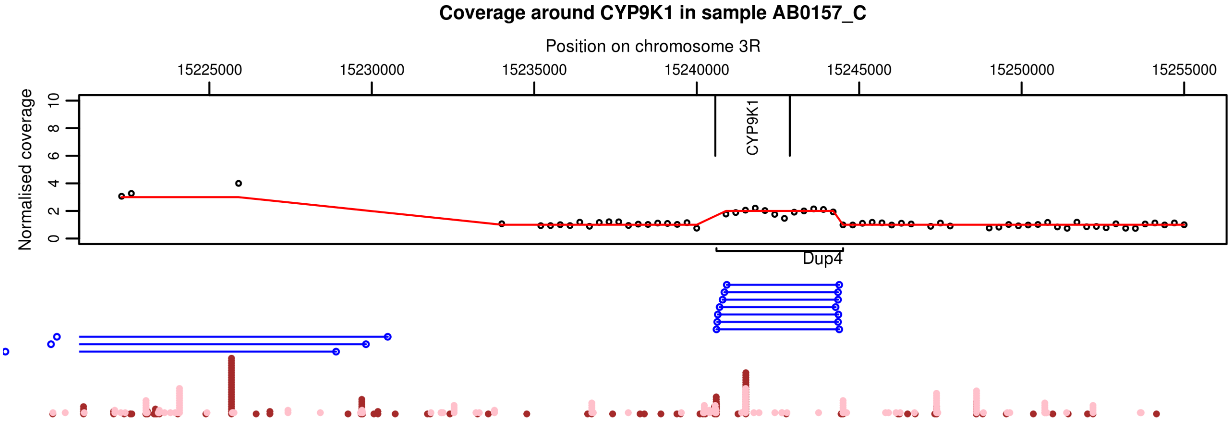

Fig. 9K1_S9: Example of coverage in an individual carrying the Cyp9k1_Dup4 duplication. Open black circles indicate coverage at each position. The red line shows the HMM estimation of the coverage state at each position. Black vertical lines represent the positions of the CYP9K1 gene. Pairs of blue points connected by lines indicate pairs of face-away reads. Reads soft-clipped before the alignment start point (dark brown points) and after the alignment end point (light brown points) are present at the start and end points of the duplication (clipped at positions 15240608 and 15244503 respectively). In each case, the clipped bases align to the other end of the duplication, as expected.

Cyp9k1_Dup4 is predominantly found in *An. gambiae* from Burkina Faso, although it was also found in Cameroon, Ghana and Guinea (Table 9K1_S4.1). Three samples that carry Cyp9k1_Dup4 are male. Estimates of copy number indicate that all three males have a copy number of 1 (Table 9K1_S4.2), suggesting that Cyp9k1_Dup4 is a single copy duplication. In females, copy number was usually 1, with a single sample having a copy number of 2 and some samples have a call of 2 when found alongside other duplications with high copy numbers (Table 9K1_S4.2). Assuming that females with a copy number of 1 are heterozygotes and that females with a copy number of 2 are homozygotes for Cyp9k1_Dup4, the allele distribution in females in Burkina Faso was consistent with Hardy-Weinberg (HW) expectations ($P = 1$).

Table 9K1_4.1: Coverage calls for Cyp9k1_Dup4. NAs were produced if coverage was too variable or if the duplication completely overlapped with another duplication whose coverage could also not be called.

copy number	AO col	BF col	BF gam	CI col	CM gam	FR gam	GA gam	GH col	GH gam	GM	GN col	GN gam	GQ gam	GW	KE	UG gam
NA	0	0	1	0	0	0	0	0	0	0	0	0	0	0	0	0
0	78	75	77	71	293	24	69	55	10	65	4	38	9	91	48	112
1	0	0	14	0	4	0	0	0	2	0	0	1	0	0	0	0
2	0	0	0	0	0	0	0	0	0	0	0	1	0	0	0	0

Table 9K1_4.2: Coverage calls for all duplications in individuals that carry Cyp9k1_Dup4. Male samples are indicated in red.

	Dup 0	Dup 1	Dup 2	Dup 3	Dup 4	Dup 5	Dup 6	Dup 7	Dup 8	Dup 9	Dup 10	Dup 11	Dup 12	Dup 13	Dup 14	Dup 15	Dup 16
AA0060_C	0	0	0	0	1	0	0	0	0	0	0	0	0	0	0	0	0
AA0122_C	0	0	0	0	1	0	0	0	0	0	0	0	0	0	0	0	0
AB0127_C	0	0	0	0	1	0	0	0	0	0	0	0	0	0	0	0	0
AB0133_C	0	0	0	0	1	0	0	0	0	0	0	0	0	0	0	0	0
AB0134_C	0	0	0	0	1	0	0	0	0	0	0	2	0	0	0	0	0
AB0135_C	0	0	0	0	0	0	0	0	0	0	0	3	0	0	0	2	0
AB0147_C	0	0	0	0	1	0	0	0	0	0	0	2	0	0	0	0	0
AB0157_C	0	0	0	0	1	0	0	0	0	0	0	0	0	0	0	0	0
AB0161_C	0	0	0	0	1	0	0	0	0	0	0	0	0	0	0	0	0
AB0200_C	0	0	0	0	1	0	0	0	0	0	0	1	0	0	0	0	0
AB0203_C	0	0	0	0	0	0	0	0	0	0	0	4	0	0	0	4	0
AB0208_C	0	0	0	0	0	0	0	0	0	0	0	5	0	0	0	0	0
AB0232_C	0	0	0	0	1	0	0	0	0	0	0	2	0	0	0	0	0
AB0235_C	0	0	0	0	1	0	0	0	0	0	0	0	0	0	0	0	0
AB0251_C	0	0	0	0	0	0	0	0	0	0	0	2	0	0	0	0	0
AB0256_C	0	0	0	0	1	0	0	0	0	0	0	0	0	0	0	4	0
AB0268_C	0	0	0	0	1	0	0	0	0	0	0	2	0	0	0	0	0
AB0270_C	0	0	0	0	1	0	0	0	0	0	0	0	0	0	0	2	0
AB0275_C	0	0	0	0	NA	0	0	0	0	0	0	0	0	0	0	NA	0
AB0278_C	0	0	0	0	1	0	0	0	0	0	0	2	0	0	0	0	0
AB0280_C	0	0	0	0	1	0	0	0	0	0	0	0	0	0	0	2	0
AN0058_C	0	0	0	0	1	0	0	0	0	0	0	0	0	0	0	0	0
AN0080_C	0	0	0	0	1	0	0	0	0	0	0	0	0	0	0	0	0
AN0265_C	0	0	0	0	1	0	0	0	0	0	0	0	0	0	0	0	0
AN0266_C	0	0	0	0	1	0	0	0	0	0	0	0	0	0	0	0	0
AV0011_C	0	0	0	0	0	0	0	0	0	0	0	2	0	0	0	0	0
AV0012_C	0	0	0	0	0	0	0	0	0	0	0	2	0	0	0	0	0
AV0029_C	0	0	0	0	0	0	0	0	0	0	0	0	0	2	0	2	0
AV0032_C	0	0	0	0	0	0	0	0	0	0	0	2	0	0	0	0	0
AV0036_C	0	0	0	0	1	0	0	0	0	0	0	0	0	0	0	2	0
AV0039_C	0	0	0	0	2	0	0	0	0	0	0	0	0	0	0	0	0

Duplication type 5

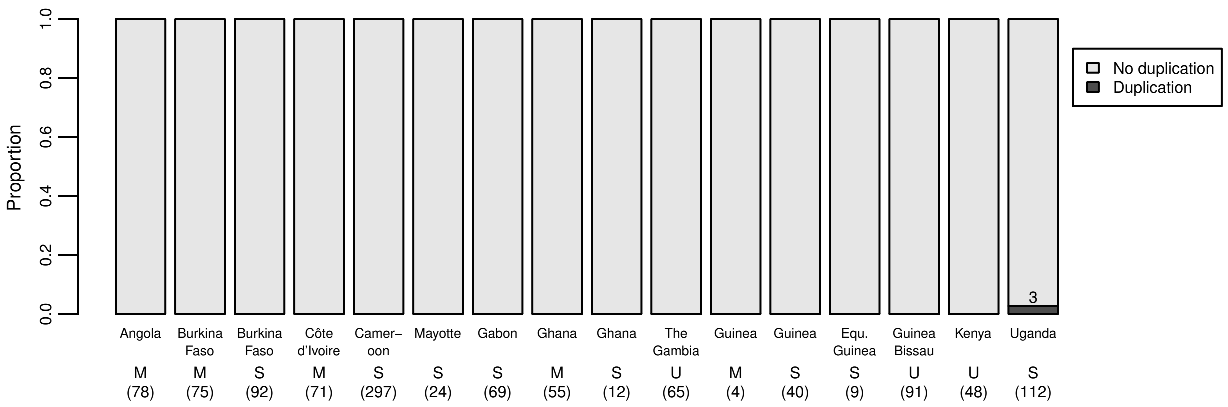

Fig. 9K1_S10: Barplot showing the proportion of samples that carry the Cyp9k1_Dup5 duplication in each of the Phase 2 populations. Numbers above the dark grey bars indicate the absolute number of samples carrying the duplication. S = *Anopheles gambiae*, M = *Anopheles coluzzi*, U = species undetermined. Numbers in brackets indicate the total number of samples from that population.

Cyp9k1_Dup5 was supported by face-away read pairs whose forward-facing read mapped in the interval 15238800 - 15239100 and whose reverse-facing read mapped in the interval 15243850 - 15244150 (**Fig. 9K1_S11**). Cyp9k1_Dup5 was also supported by reads soft-clipped at the breakpoints (positions 15238911 and 15244175), with the clipped bases at each breakpoint aligning at the other breakpoint.

Cyp9k1_Dup5 breakpoint:

```
TTGGACCATCATTGCAC TA AGTAAAAATAATTGTA
 end of the dup ^     ^ start of the dup
position 15244172    position 15238914
```

The TA could sit on either side of the breakpoint.

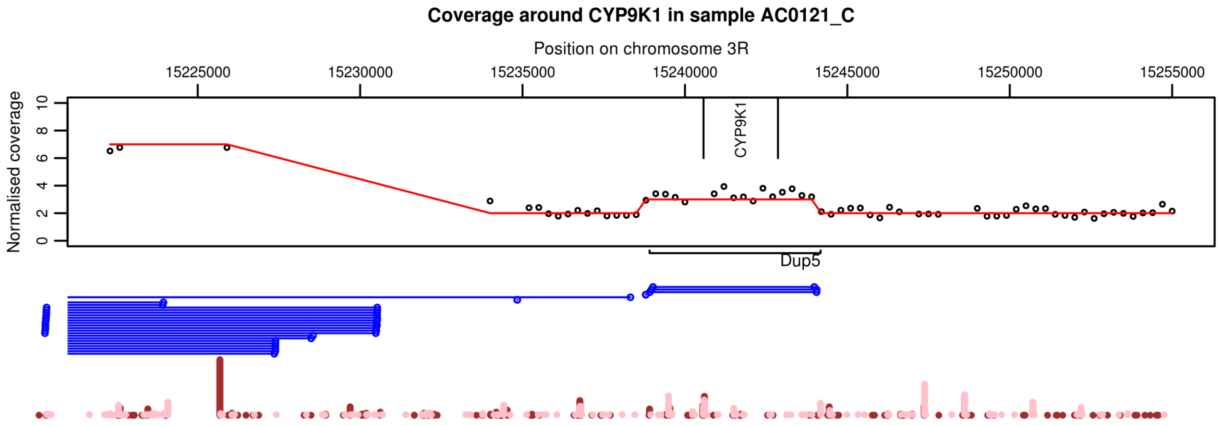

Fig. 9K1_S11: Example of coverage in an individual carrying the Cyp9k1_Dup5 duplication. Open black circles indicate coverage at each position. The red line shows the HMM estimation of the coverage state at each position. Black vertical lines represent the positions of the CYP9K1 gene. Pairs of blue points connected by lines indicate pairs of face-away reads. Reads soft-clipped before the alignment start point (dark brown points) and after the alignment end point (light brown points) are present at the start and end points of the duplication (clipped at positions 15238911 and 15244175 respectively). In each case, the clipped bases align to the other end of the duplication, as expected.

Cyp9k1_Dup5 was found in three samples, all of which were female. One sample had no coverage calls. (Tables 9K1_S5.1 & 5.2). Estimates of copy number from the other two samples indicated that both had a copy number of 1, suggesting that Cyp9k1_Dup5 is a single copy duplication and the samples are heterozygous.

Table 9K1_5.1: Coverage calls for Cyp9k1_Dup5. NAs were produced if coverage was too variable or if the duplication completely overlapped with another duplication whose coverage could also not be called.

copy number	AO col	BF col	BF gam	CI col	CM gam	FR gam	GA gam	GH col	GH gam	GM	GN col	GN gam	GQ gam	GW	KE	UG gam
NA	0	0	0	0	0	0	0	0	0	0	0	0	0	0	0	1
0	78	75	92	71	297	24	69	55	12	65	4	40	9	91	48	109
1	0	0	0	0	0	0	0	0	0	0	0	0	0	0	0	2

Table 9K1_5.2: Coverage calls for all duplications in individuals that carry Cyp9k1_Dup5. Male samples are indicated in red.

	Dup 0	Dup 1	Dup 2	Dup 3	Dup 4	Dup 5	Dup 6	Dup 7	Dup 8	Dup 9	Dup 10	Dup 11	Dup 12	Dup 13	Dup 14	Dup 15	Dup 16
AC0121_C	0	0	0	0	0	1	0	0	0	0	0	0	0	0	0	0	0
AC0149_C	0	0	0	0	0	1	0	0	0	0	0	0	0	0	0	0	0
AC0199_C	0	0	0	0	0	NA	0	0	NA	0	0	0	0	0	0	0	0

Duplication type 6

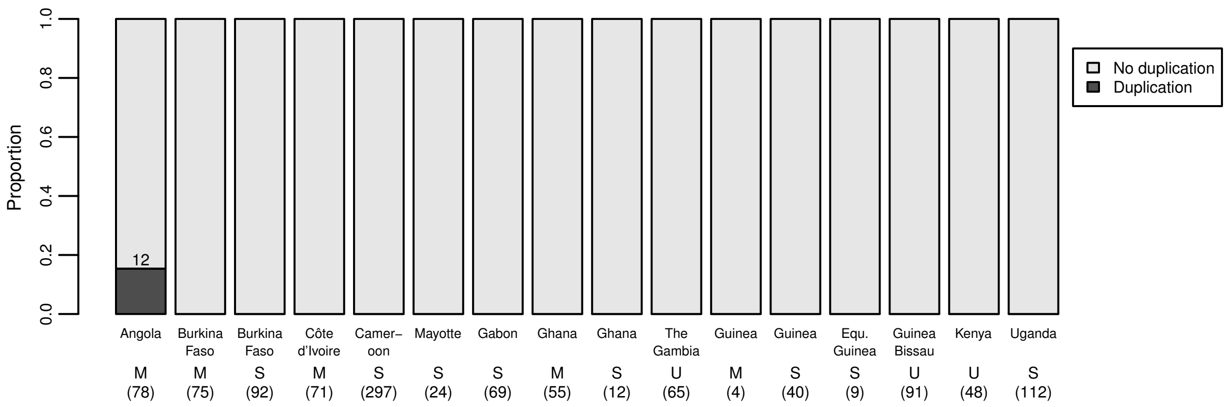

Fig. 9K1_S12: Barplot showing the proportion of samples that carry the Cyp9k1_Dup6 duplication in each of the Phase 2 populations. Numbers above the dark grey bars indicate the absolute number of samples carrying the duplication. S = *Anopheles gambiae*, M = *Anopheles coluzzi*, U = species undetermined. Numbers in brackets indicate the total number of samples from that population.

Cyp9k1_Dup6 was supported by face-away read pairs whose forward-facing read mapped in the interval 15236400 - 15236700 and whose reverse-facing read mapped in the interval 15243250 - 15243550 (Fig. 9K1_S13). Cyp9k1_Dup6 was also supported by reads soft-clipped at the breakpoints (positions 15236449 and 15243646), with the clipped bases at each breakpoint aligning at the other breakpoint.

Cyp9k1_Dup6 breakpoint:

```
GATTGACGCAGTGGTTATC GTTTTTGCGGTTTTTT GTAGTACGTAGAGATGG
     end of the dup ^   inserted seq   ^ start of the dup
  position 15243646                    position 15236450
```

The sequence GTTTTTGCGGTTTTTT is inserted between the sequences on either side of the breakpoint.

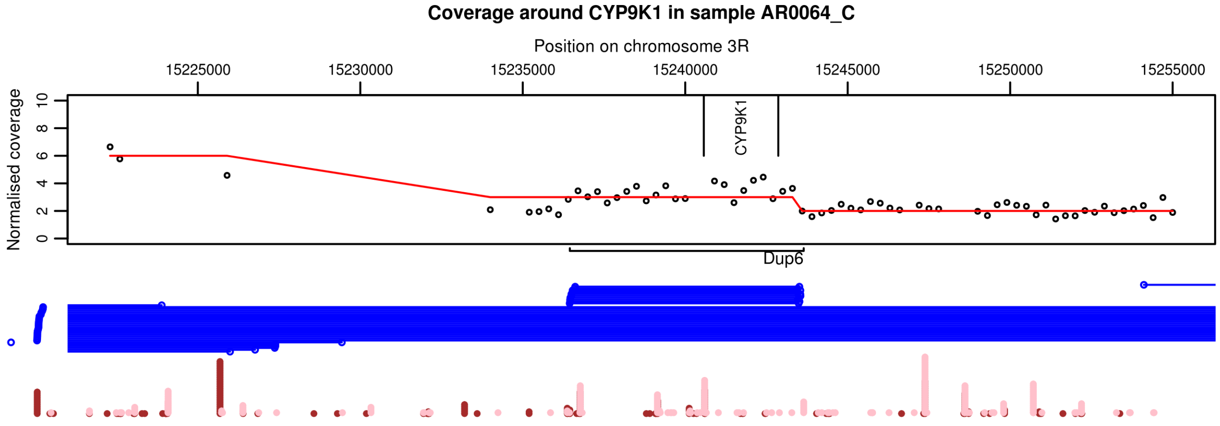

Fig. 9K1_S13: Example of coverage in an individual carrying the Cyp9k1_Dup6 duplication. Open black circles indicate coverage at each position. The red line shows the HMM estimation of the coverage state at each position. Black vertical lines represent the positions of the CYP9K1 gene. Pairs of blue points connected by lines indicate pairs of face-away reads. Reads soft-clipped before the alignment start point (dark brown points) and after the alignment end point (light brown points) are present at the start and end points of the duplication (clipped at positions 15236449 and 15243646 respectively). In each case, the clipped bases align to the other end of the duplication, as expected.

All 12 samples with Cyp9k1_Dup6 were found in *An. coluzzii* from Angola (Table 9K1_S6.1) and all were female (Table 9K1_S6.2). Estimates of copy number indicated that no samples had a copy number higher than 2. Assuming that females with a copy number of 1 are heterozygotes and that females with a copy number of 2 are homozygotes for Cyp9k1_Dup6, the allele distribution in females is consistent with HW expectations ($P = 0.42$).

Table 9K1_6.1: Coverage calls for Cyp9k1_Dup6. NAs were produced if coverage was too variable or if the duplication completely overlapped with another duplication whose coverage could also not be called.

copy number	AO col	BF col	BF gam	CI col	CM gam	FR gam	GA gam	GH col	GH gam	GM	GN col	GN gam	GQ gam	GW	KE	UG gam
0	66	75	92	71	297	24	69	55	12	65	4	40	9	91	48	112
1	11	0	0	0	0	0	0	0	0	0	0	0	0	0	0	0
2	1	0	0	0	0	0	0	0	0	0	0	0	0	0	0	0

Table 9K1_6.2: Coverage calls for all duplications in individuals that carry Cyp9k1_Dup6. Male samples are indicated in red.

	Dup 0	Dup 1	Dup 2	Dup 3	Dup 4	Dup 5	Dup 6	Dup 7	Dup 8	Dup 9	Dup 10	Dup 11	Dup 12	Dup 13	Dup 14	Dup 15	Dup 16
AR0013_C	0	0	0	0	0	0	1	0	0	0	0	0	0	0	0	0	0
AR0024_C	0	0	0	0	0	0	1	0	0	0	0	0	0	0	0	0	0
AR0035_C	0	0	0	0	0	0	1	0	0	0	0	0	0	0	0	0	0
AR0036_C	0	0	0	0	0	0	2	0	0	0	0	0	0	0	0	0	0
AR0038_C	0	0	0	0	0	0	1	0	0	0	0	0	0	0	0	0	0
AR0049_C	0	0	0	0	0	0	1	0	0	0	0	0	0	0	0	0	0
AR0064_C	0	0	0	0	0	0	1	0	0	0	0	0	0	0	0	0	0
AR0065_C	0	0	0	0	0	0	1	0	0	0	0	0	0	0	0	0	0
AR0070_C	0	0	0	0	0	0	1	0	0	0	0	0	0	0	0	0	0
AR0079_C	0	0	0	0	0	0	1	0	0	0	0	0	0	0	0	0	0
AR0096_C	0	0	0	0	0	0	1	0	0	0	0	0	0	0	0	0	0
AR0098_C	0	0	0	0	0	0	1	0	0	0	0	0	0	0	0	0	0

Duplication type 7

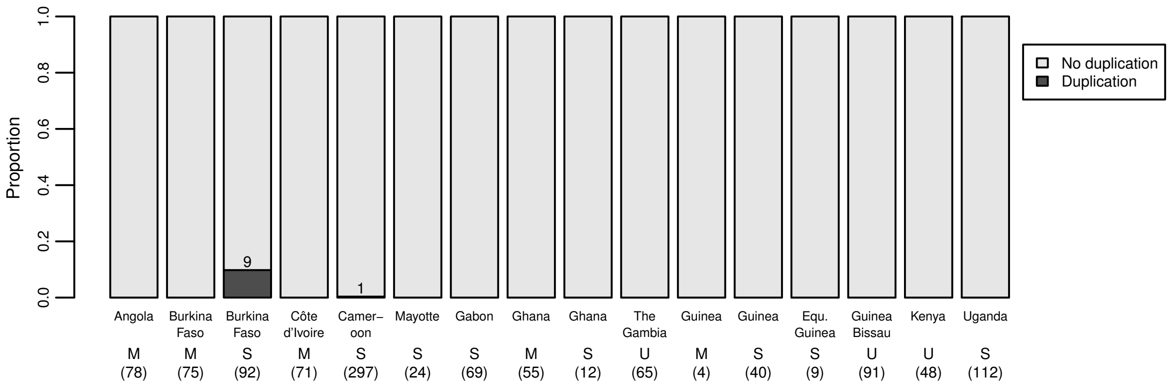

Fig. 9K1_S14: Barplot showing the proportion of samples that carry the Cyp9k1_Dup7 duplication in each of the Phase 2 populations. Numbers above the dark grey bars indicate the absolute number of samples carrying the duplication. S = *Anopheles gambiae*, M = *Anopheles coluzzi*, U = species undetermined. Numbers in brackets indicate the total number of samples from that population.

Cyp9k1_Dup7 was associated with same-strand read pairs for which one read mapped in the interval 15245400 - 15245700 and the other read mapped in the interval 15246900 - 15247200 (Fig. **9K1_S15**). These read pairs are probably only correlated with the presence of Cyp9k1_Dup7, since they do not match the range of coverage increase and thus are unlikely to be directly linked to the duplication. Also, the coverage within the region defined by the same-strand read pairs is lower that of the rest of the region, speculatively suggesting that the same-strand reads mark a deletion from an invertion duplication. The duplication was detected using the same-strand read pairs and soft-clipped reads mapping to the breakpoints associated with the same-strand reads (reads soft clipped at position 15245768 and 15247258).

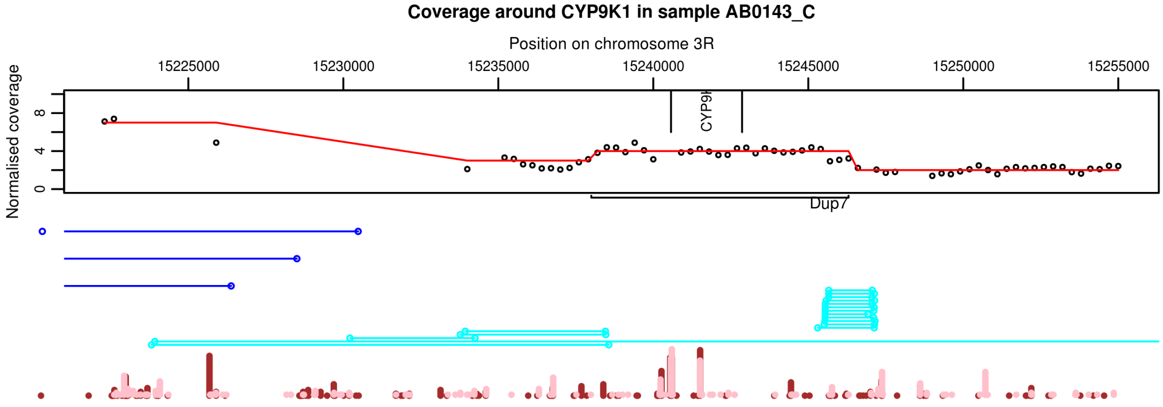

Fig. 9K1_S15: Example of coverage in an individual carrying the dup15 duplication. Open black circles indicate coverage at each position. The red line shows the HMM estimation of the coverage state at each position. Black vertical lines represent the positions of the CYP9K1 gene. Pairs of dark blue points connected by lines indicate pairs of face-away reads. Pairs of cyan points connected by lines indicate pairs of same-strand reads. The alignment start and end points could not be determined accurately and the extent of the duplication represented here is not necessarily correct.

Cyp9k1_Dup7 was found in *An. coluzzii* from Burkina Faso, and in a single sample from Cameroon (Table 9K1_S7.1) Estimates of copy number for Cyp9k1_Dup7 were difficult to obtain as it was often found alongside other duplications covering a similar region (Table 9K1_S7.2). The single male sample with Cyp9k1_Dup7 had a copy number of 2, suggesting that a triplication is present for Cyp9k1_Dup7. Given these difficulties, it was not possible to obtain genotype calls for Cyp9k1_Dup7.

Table 9K1_7.1: Coverage calls for Cyp9k1_Dup7. NAs were produced if coverage was too variable or if the duplication completely overlapped with another duplication whose coverage could also not be called.

copy number	AO col	BF col	BF gam	CI col	CM gam	FR gam	GA gam	GH col	GH gam	GM	GN col	GN gam	GQ gam	GW	KE	UG gam
NA	0	0	4	0	0	0	0	0	0	0	0	0	0	0	0	0
0	78	75	86	71	296	24	69	55	12	65	4	40	9	91	48	112
2	0	0	2	0	1	0	0	0	0	0	0	0	0	0	0	0

Table 9K1_7.2: Coverage calls for all duplications in individuals that carry Cyp9k1_Dup7. Male samples are indicated in red.

	Dup 0	Dup 1	Dup 2	Dup 3	Dup 4	Dup 5	Dup 6	Dup 7	Dup 8	Dup 9	Dup 10	Dup 11	Dup 12	Dup 13	Dup 14	Dup 15	Dup 16
AB0143_C	0	0	0	0	0	0	0	2	0	0	0	0	0	0	0	0	0
AB0148_C	0	0	0	0	0	0	0	0	0	0	0	0	0	0	0	3	0
AB0158_C	0	0	0	0	0	0	0	2	0	0	0	0	0	0	0	0	0
AB0197_C	0	0	0	0	0	0	0	NA	0	0	0	0	0	0	0	NA	0
AB0201_C	0	0	0	0	0	0	0	0	0	0	0	0	0	0	0	4	0
AB0233_C	0	0	0	0	0	0	0	0	0	0	0	3	0	0	0	0	0
AB0238_C	0	0	0	0	0	0	0	NA	0	0	0	0	0	0	0	0	0
AB0260_C	0	0	0	0	0	0	0	NA	0	0	0	NA	0	0	0	3	0
AB0273_C	0	0	0	0	0	0	0	NA	0	0	0	NA	0	0	0	5	0
AN0172_C	0	0	0	0	0	0	0	2	0	0	0	0	0	0	0	0	0

Duplication type 8

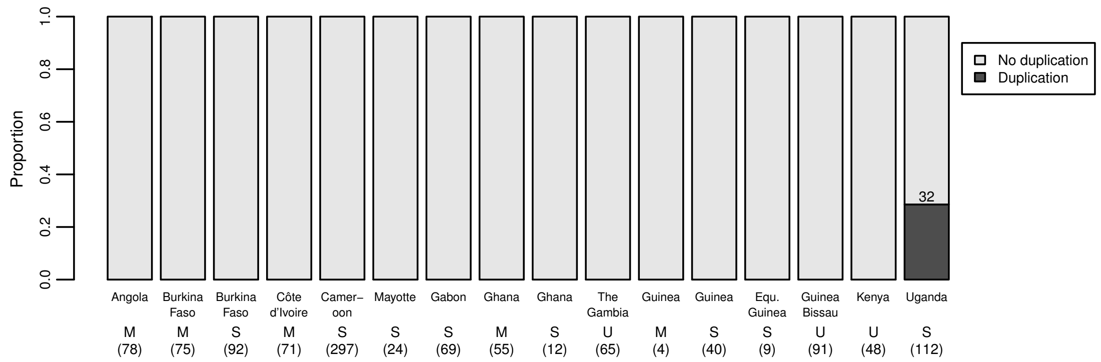

Fig. 9K1_S16: Barplot showing the proportion of samples that carry the Cyp9k1_Dup8 duplication in each of the Phase 2 populations. Numbers above the dark grey bars indicate the absolute number of samples carrying the duplication. S = *Anopheles gambiae*, M = *Anopheles coluzzi*, U = species undetermined. Numbers in brackets indicate the total number of samples from that population.

Cyp9k1_Dup8 was supported by face-away read pairs whose forward-facing read mapped in the interval 15239200 - 15239500 and whose reverse-facing read mapped in the interval 15247250 - 15247550 (Fig. **9K1_S17**). A deletion inside Cyp9k1_Dup8 was supported by reads pairs that mapped facing towards each-other, with the first read mapping around position 15243800 and its mate mapping around position 15247300 (Fig. **9K1_S17**). Cyp9k1_Dup8 was also supported by reads soft-clipped at the breakpoints (positions 15239276 and 15247645), with the clipped bases at each breakpoint aligning at the other breakpoint.

Cyp9k1_Dup8 breakpoint:

```
GCTGTTGCAATGCTACAA CGTA TTGCTTAAGAAAAGTA
  end of the dup ^       ^ start of the dup
 position 15247640       position 15239281
```

The `CGTA` could sit on either side of the breakpoint.

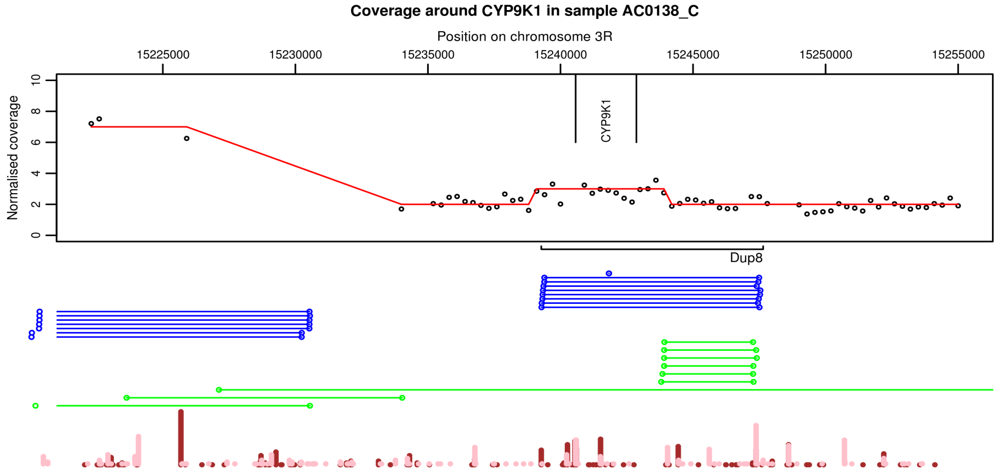

Fig. 9K1_S17: Example of coverage in an individual carrying the Cyp9k1_Dup8 duplication. Open black circles indicate coverage at each position. The red line shows the HMM estimation of the coverage state at each position. Black vertical lines represent the positions of the CYP9K1 gene. Pairs of blue points connected by lines indicate pairs of face-away reads. Reads soft-clipped before the alignment start point (dark brown points) and after the alignment end point (light brown points) are present at the start and end points of the duplication (clipped at positions 15239276 and 15247645 respectively). In each case, the clipped bases align to the other end of the duplication, as expected. Green points joined by horizontal lines indicate pairs of reads that mapped facing each-other but more than 1000bp apart, indicating a possible deletion.

All samples with Cyp9k1_Dup8 were found were An. gambiae from Uganda, and all are female. Estimates of copy number for Cyp9k1_Dup8 in this population indicated no sample had a copy number higher than 2 (Table 9K1_S8.1). Assuming that samples with a copy number of 1 are heterozygotes and that samples with a copy number of 2 are homozygotes for Cyp9k1_Dup8, the allele distribution is consistent with HW expectations ($P = 0.72$).

Table 9K1_8.1: Coverage calls for Cyp9k1_Dup8. NAs were produced if coverage was too variable or if the duplication completely overlapped with another duplication whose coverage could also not be called.

copy number	AO col	BF col	BF gam	CI col	CM gam	FR gam	GA gam	GH col	GH gam	GM	GN col	GN gam	GQ gam	GW	KE	UG gam
NA	0	0	0	0	0	0	0	0	0	0	0	0	0	0	0	1
0	78	75	92	71	297	24	69	55	12	65	4	40	9	91	48	80
1	0	0	0	0	0	0	0	0	0	0	0	0	0	0	0	28
2	0	0	0	0	0	0	0	0	0	0	0	0	0	0	0	3

Table 9K1_8.2: Coverage calls for all duplications in individuals that carry Cyp9k1_Dup8. Male samples are indicated in red.

	Dup 0	Dup 1	Dup 2	Dup 3	Dup 4	Dup 5	Dup 6	Dup 7	Dup 8	Dup 9	Dup 10	Dup 11	Dup 12	Dup 13	Dup 14	Dup 15	Dup 16
AC0090_C	0	0	0	0	0	0	0	0	1	0	0	0	0	0	0	0	0
AC0094_C	0	0	0	0	0	0	0	0	1	0	0	0	0	0	0	0	0
AC0097_C	0	0	0	0	0	0	0	0	1	0	0	0	0	0	0	0	0
AC0101_C	0	0	0	0	0	0	0	0	1	0	0	0	0	0	0	0	0
AC0102_C	0	0	0	0	0	0	0	0	1	0	0	0	0	0	0	0	0
AC0106_C	0	0	0	0	0	0	0	0	1	0	0	0	0	0	0	0	0
AC0107_C	0	0	0	0	0	0	0	0	2	0	0	0	0	0	0	0	0
AC0108_C	0	0	0	0	0	0	0	0	1	0	0	0	0	0	0	0	0
AC0117_C	0	0	0	0	0	0	0	0	1	0	0	0	0	0	0	0	0
AC0122_C	0	0	0	0	0	0	0	0	1	0	0	0	0	0	0	0	0
AC0124_C	0	0	0	0	0	0	0	0	1	0	0	0	0	0	0	0	0
AC0128_C	0	0	0	0	0	0	0	0	1	0	0	0	0	0	0	0	0
AC0132_C	0	0	0	0	0	0	0	0	2	0	0	0	0	0	0	0	0
AC0137_C	0	0	0	0	0	0	0	0	1	0	0	0	0	0	0	0	0
AC0138_C	0	0	0	0	0	0	0	0	1	0	0	0	0	0	0	0	0
AC0139_C	0	0	0	0	0	0	0	0	1	0	0	0	0	0	0	0	0
AC0141_C	0	0	0	0	0	0	0	0	1	0	0	0	0	0	0	0	0
AC0153_C	0	0	0	0	0	0	0	0	1	0	0	0	0	0	0	0	0
AC0155_C	0	0	0	0	0	0	0	0	1	0	0	0	0	0	0	0	0
AC0159_C	0	0	0	0	0	0	0	0	1	0	0	0	0	0	0	0	0
AC0160_C	0	0	0	0	0	0	0	0	1	0	0	0	0	0	0	0	0
AC0164_C	0	0	0	0	0	0	0	0	1	0	0	0	0	0	0	0	0
AC0166_C	0	0	0	0	0	0	0	0	1	0	0	0	0	0	0	0	0
AC0168_C	0	0	0	0	0	0	0	0	1	0	0	0	0	0	0	0	0
AC0170_C	0	0	0	0	0	0	0	0	1	0	0	0	0	0	0	0	0
AC0180_C	0	0	0	0	0	0	0	0	1	0	0	0	0	0	0	0	0
AC0187_C	0	0	0	0	0	0	0	0	2	0	0	0	0	0	0	0	0
AC0189_C	0	0	0	0	0	0	0	0	1	0	0	0	0	0	0	0	0
AC0199_C	0	0	0	0	0	NA	0	0	NA	0	0	0	0	0	0	0	0
AC0200_C	0	0	0	0	0	0	0	0	1	0	0	0	0	0	0	0	0
AC0202_C	0	0	0	0	0	0	0	0	1	0	0	0	0	0	0	0	0
AC0203_C	0	0	0	0	0	0	0	0	1	0	0	0	0	0	0	0	0

Duplication type 9

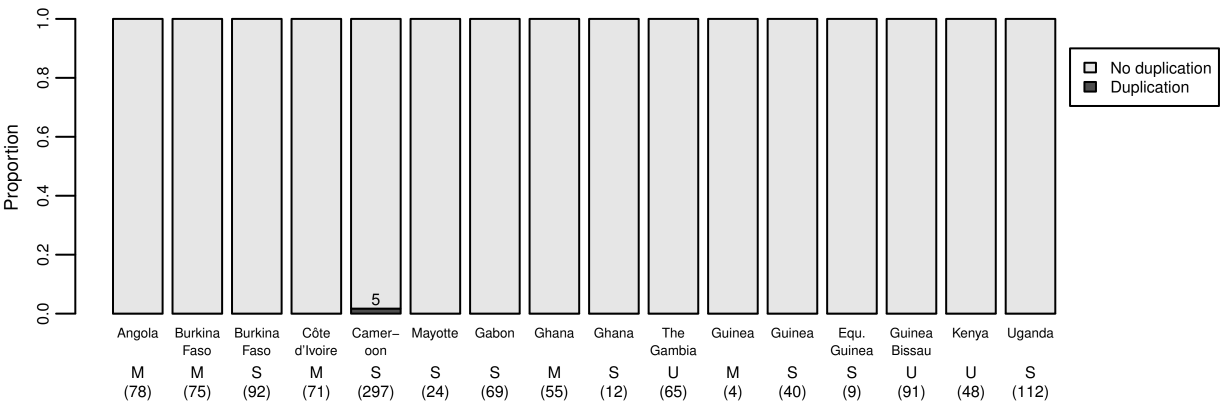

Fig. 9K1_S18: Barplot showing the proportion of samples that carry the Cyp9k1_Dup9 duplication in each of the Phase 2 populations. Numbers above the dark grey bars indicate the absolute number of samples carrying the duplication. S = *Anopheles gambiae*, M = *Anopheles coluzzi*, U = species undetermined. Numbers in brackets indicate the total number of samples from that population.

Cyp9k1_Dup9 was supported by face-away read pairs whose forward-facing read mapped in the interval 15239100 - 15239400 and whose reverse-facing read mapped in the interval 15248900 - 15249200 (Fig. 9K1_S19). Cyp9k1_Dup9 was also supported by reads soft-clipped at the breakpoints (positions 15239184 and 15249314), with the clipped bases at each breakpoint aligning at the other breakpoint.

Cyp9k1_Dup9 breakpoint:

```
GGCCTCCTAGATACACG GT GAACAGTGAAGTACTATTTTTTTCG
 end of the dup ^     ^ start of the dup
position 15249302     position 15239187
```

The GT could sit on either side of the breakpoint.

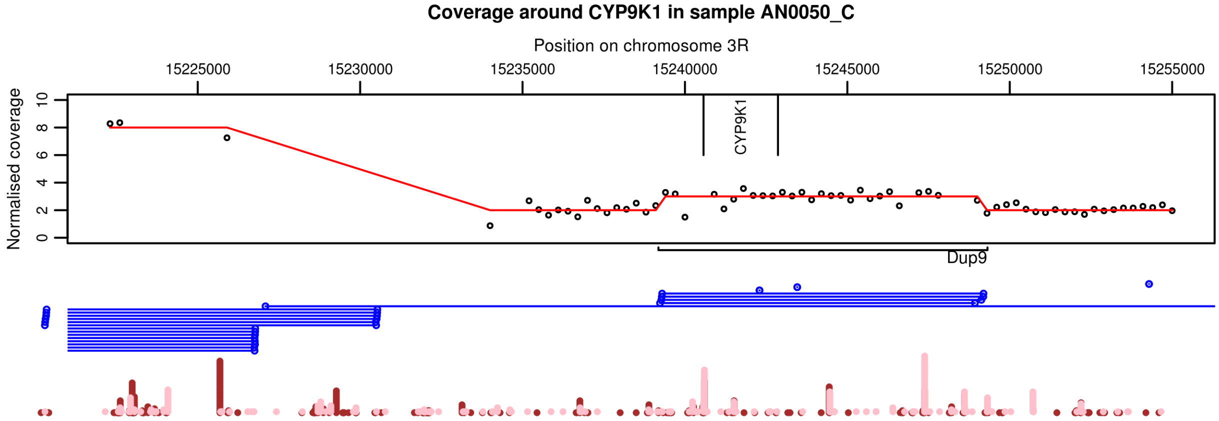

Fig. 9K1_S19: Example of coverage in an individual carrying the Cyp9k1_Dup9 duplication. Open black circles indicate coverage at each position. The red line shows the HMM estimation of the coverage state at each position. Black vertical lines represent the positions of the CYP9K1 gene. Pairs of blue points connected by lines indicate pairs of face-away reads. Reads soft-clipped before the alignment start point (dark brown points) and after the alignment end point (light brown points) are present at the start and end points of the duplication (clipped at positions 15239184 and 15249314 respectively). In each case, the clipped bases align to the other end of the duplication, as expected.

All five samples with Cyp9k1_Dup9 were *An. gambiae* from Cameroon (Table 9K1_S9.1), comprising four females and one male. Estimates of copy number indicated that the male had a copy number of 1 and all of the females also had a copy number of 1 (Table 9K1_S9.2), Cyp9k1_Dup9 is therefore a single-copy duplication and all females samples are heterozygous.

Table 9K1_9.1: Coverage calls for Cyp9k1_Dup9. NAs were produced if coverage was too variable or if the duplication completely overlapped with another duplication whose coverage could also not be called.

copy number	AO col	BF col	BF gam	CI col	CM gam	FR gam	GA gam	GH col	GH gam	GM	GN col	GN gam	GQ gam	GW	KE	UG gam
0	78	75	92	71	292	24	69	55	12	65	4	40	9	91	48	112
1	0	0	0	0	5	0	0	0	0	0	0	0	0	0	0	0

Table 9K1_9.2: Coverage calls for all duplications in individuals that carry Cyp9k1_Dup9. Male samples are indicated in red.

	Dup 0	Dup 1	Dup 2	Dup 3	Dup 4	Dup 5	Dup 6	Dup 7	Dup 8	Dup 9	Dup 10	Dup 11	Dup 12	Dup 13	Dup 14	Dup 15	Dup 16
AN0050_C	0	0	0	0	0	0	0	0	0	1	0	0	0	0	0	0	0
AN0059_C	0	0	0	0	0	0	0	0	0	1	0	0	0	0	0	0	0
AN0105_C	0	0	0	0	0	0	0	0	0	1	0	0	0	0	0	0	0
AN0236_C	0	0	0	0	0	0	0	0	0	1	0	0	0	0	0	0	0
AN0299_C	0	0	0	0	0	0	0	0	0	1	0	0	0	0	0	0	0

Duplication type 10

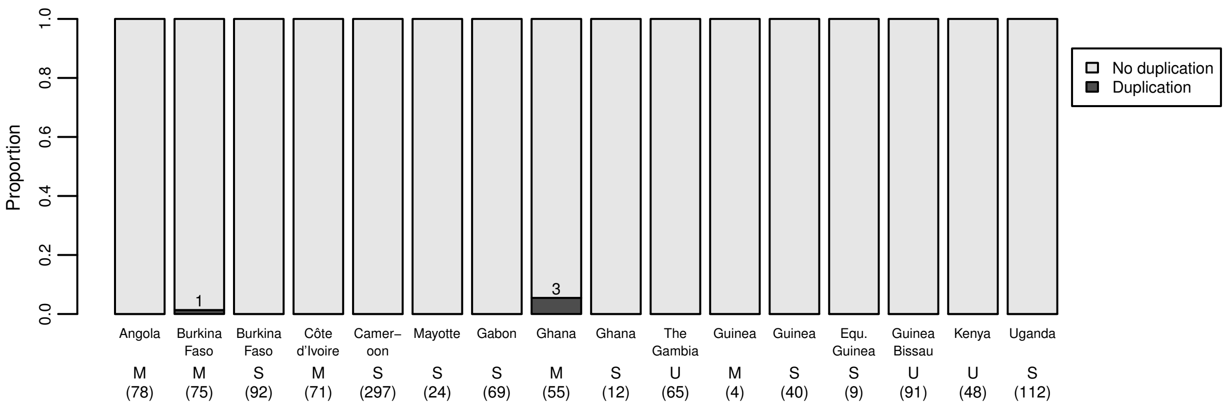

Fig. 9K1_S20: Barplot showing the proportion of samples that carry the Cyp9k1_Dup10 duplication in each of the Phase 2 populations. Numbers above the dark grey bars indicate the absolute number of samples carrying the duplication. S = *Anopheles gambiae*, M = *Anopheles coluzzi*, U = species undetermined. Numbers in brackets indicate the total number of samples from that population.

Cyp9k1_Dup10 was supported by face-away read pairs whose forward-facing read mapped in the interval 15234900 - 15235200 and whose reverse-facing read mapped in the interval 15244750 - 15245050 (Fig. **9K1_S21**). Cyp9k1_Dup10 was also supported by reads soft-clipped at the breakpoints (positions 15234989 and 15245128), with the clipped bases at each breakpoint aligning at the other breakpoint.

Cyp9k1_Dup10 breakpoint:

```
ACACAGGCTCGACACATCCGCCACG C ATTCTGAAAAGCCTTATCAGTT
             end of the dup ^   ^ start of the dup
            position 15245126   position 15234991
```

The C could sit on either side of the breakpoint.

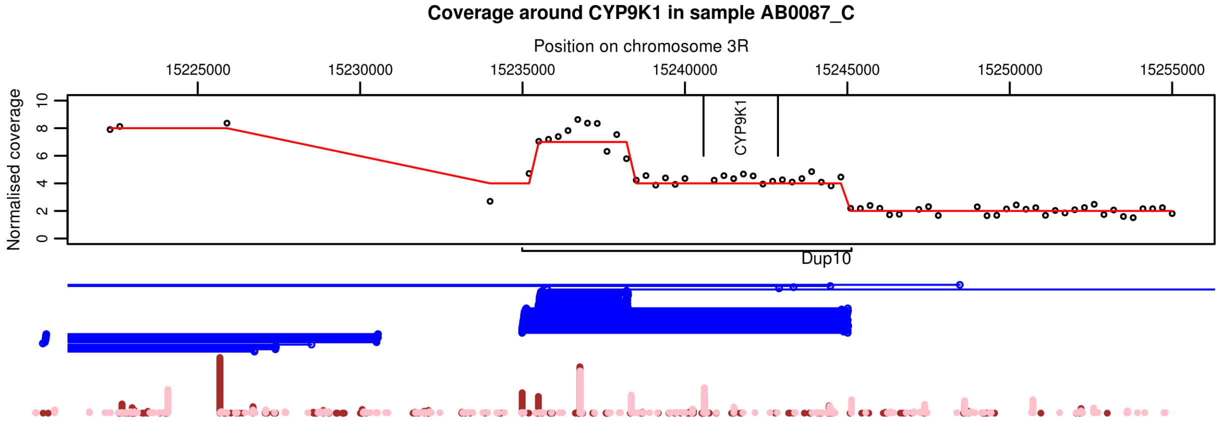

Fig. 9K1_S21: Example of coverage in an individual carrying the Cyp9k1_Dup10 duplication. Open black circles indicate coverage at each position. The red line shows the HMM estimation of the coverage state at each position. Black vertical lines represent the positions of the CYP9K1 gene. Pairs of blue points connected by lines indicate pairs of face-away reads. Reads soft-clipped before the alignment start point (dark brown points) and after the alignment end point (light brown points) are present at the start and end points of the duplication (clipped at positions 15234989 and 15245128 respectively). In each case, the clipped bases align to the other end of the duplication, as expected.

Cyp9k1_Dup10 was found in *An. coluzzii* from Ghana (three females) and from Burkina Faso (one female). Estimates of copy number indicated that all three samples from Ghana had a copy number of 1, while the single sample from Burkina Faso had a copy number of 2 (Table 9K1_S10.1). Assuming that samples with a copy number of 1 are heterozygotes and that samples with a copy number of 2 are homozygotes for Cyp9k1_Dup10, the allele distribution in Burkina Faso is significantly different from HW expectations ($P = 0.007$). It may therefore be that Cyp9k1_Dup10 is a single copy duplication in Ghana, with a rare triplication existing in Burkina Faso.

Table 9K1_10.1: Coverage calls for Cyp9k1_Dup10. NAs were produced if coverage was too variable or if the duplication completely overlapped with another duplication whose coverage could also not be called.

copy number	AO col	BF col	BF gam	CI col	CM gam	FR gam	GA gam	GH col	GH gam	GM	GN col	GN gam	GQ gam	GW	KE	UG gam
0	78	74	92	71	297	24	69	52	12	65	4	40	9	91	48	112
1	0	0	0	0	0	0	0	3	0	0	0	0	0	0	0	0
2	0	1	0	0	0	0	0	0	0	0	0	0	0	0	0	0

Table 9K1_10.2: Coverage calls for all duplications in individuals that carry Cyp9k1_Dup10. Male samples are indicated in red.

	Dup 0	Dup 1	Dup 2	Dup 3	Dup 4	Dup 5	Dup 6	Dup 7	Dup 8	Dup 9	Dup 10	Dup 11	Dup 12	Dup 13	Dup 14	Dup 15	Dup 16
AA0074_C	0	0	0	0	0	0	0	0	0	0	1	0	0	0	0	0	0
AA0132_C	0	0	0	0	0	0	0	0	0	0	1	0	0	0	0	0	0
AA0134_C	0	0	0	0	0	0	0	0	0	0	1	0	0	0	0	0	0
AB0087_C	0	0	0	0	0	0	0	0	0	0	2	0	0	0	0	0	0

Duplication type 11

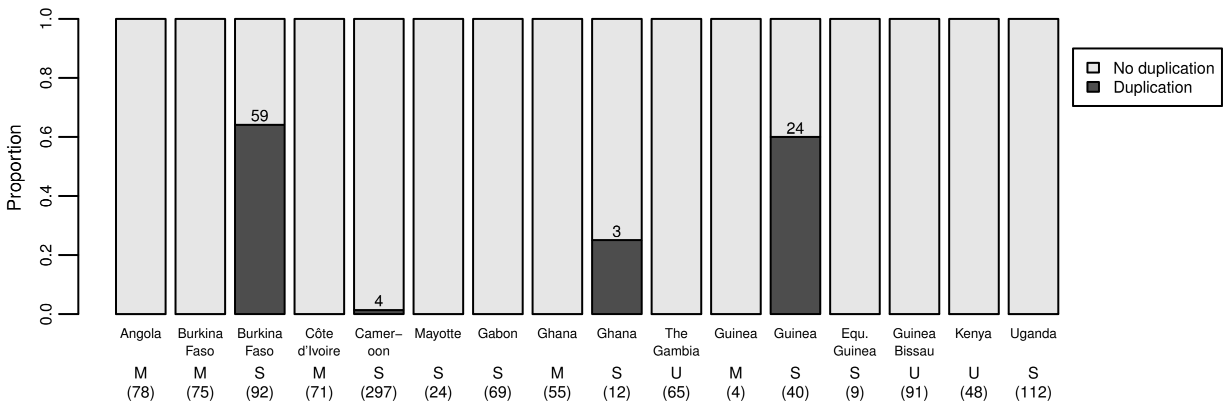

Fig. 9K1_S22: Barplot showing the proportion of samples that carry the Cyp9k1_Dup11 duplication in each of the Phase 2 populations. Numbers above the dark grey bars indicate the absolute number of samples carrying the duplication. S = *Anopheles gambiae*, M = *Anopheles coluzzi*, U = species undetermined. Numbers in brackets indicate the total number of samples from that population.

Cyp9k1_Dup11 was supported by face-away read pairs whose forward-facing read mapped in the interval 15236900 - 15237200 and whose reverse-facing read mapped in the interval 15246800 - 15247100 (Fig. **9K1_S23**). Cyp9k1_Dup11 was also supported by reads soft-clipped at the breakpoints (positions 15236922 and 15247159), with the clipped bases at each breakpoint aligning at the other breakpoint.

Cyp9k1_Dup11 breakpoint:

```
TAGTTATGTTTCTAGTTTTATTACATAT TATCTAGTTTTATTAC ATACGTGCTACCGAGTTTTAACCGT
                 end of the dup ^   inserted seq    ^ start of the dup
               position 15247158                    position 15236923
```

The sequence TATCTAGTTTTATTAC is inserted between the sequences on either side of the breakpoint. Interestingly, it is a near-exact repeat of the sequence before the breakpoint.

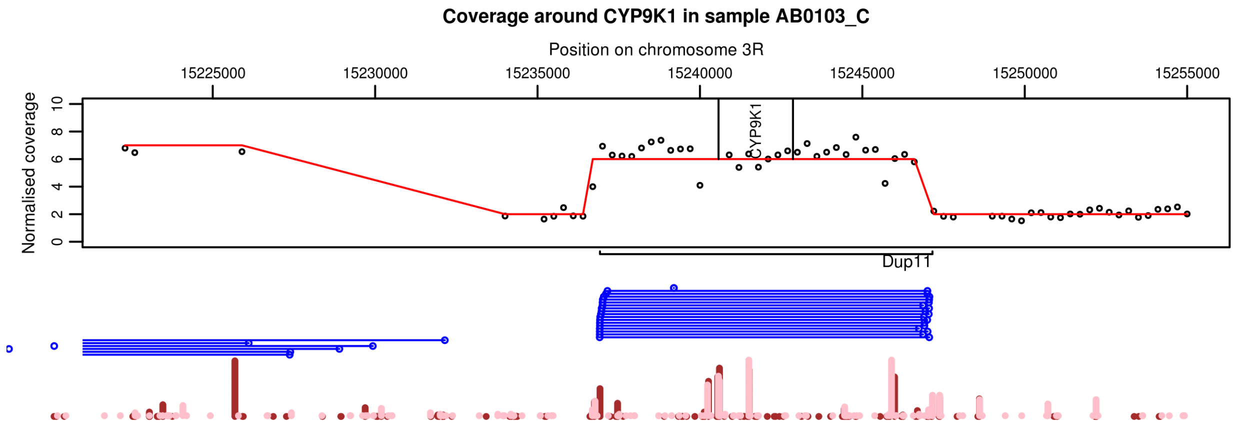

Fig. 9K1_S23: Example of coverage in an individual carrying the Cyp9k1_Dup11 duplication. Open black circles indicate coverage at each position. The red line shows the HMM estimation of the coverage state at each position. Black vertical lines represent the positions of the CYP9K1 gene. Pairs of blue points connected by lines indicate pairs of face-away reads. Reads soft-clipped before the alignment start point (dark brown points) and after the alignment end point (light brown points) are present at the start and end points of the duplication (clipped at positions 15236922 and 15247159 respectively). In each case, the clipped bases align to the other end of the duplication, as expected.

Cyp9k1_Dup11 was found in *An. gambiae* from Burkina Faso and Guinea (Table 9K1_S11.1). Estimates of copy number ranged from 1 to 5 in females and 1 to 3 in males, indicating that both single copy duplications and higher-order amplificaions exist for Cyp9k1_Dup11. Furthermore, Cyp9k1_Dup11 is often found alongside other high copy number duplications, such as Cyp9k1_Dup15 (Table 9K1_S11.2), bringing further uncertainty to the Cyp9k1_Dup11 coverage calls. It was therefore not possible to make confident genotype calls for Cyp9k1_Dup11.

Table 9K1_11.1: Coverage calls for Cyp9k1_Dup11. NAs were produced if coverage was too variable or if the duplication completely overlapped with another duplication whose coverage could also not be called.

copy number	AO col	BF col	BF gam	CI col	CM gam	FR gam	GA gam	GH col	GH gam	GM	GN col	GN gam	GQ gam	GW	KE	UG gam
NA	0	0	4	0	0	0	0	0	0	0	0	0	0	0	0	0
0	78	75	33	71	293	24	69	55	9	65	4	19	9	91	48	112
1	0	0	6	0	2	0	0	0	0	0	0	1	0	0	0	0
2	0	0	29	0	2	0	0	0	3	0	0	15	0	0	0	0
3	0	0	7	0	0	0	0	0	0	0	0	3	0	0	0	0
4	0	0	12	0	0	0	0	0	0	0	0	1	0	0	0	0
5	0	0	1	0	0	0	0	0	0	0	0	1	0	0	0	0

Table 9K1_11.2: Coverage calls for all duplications in individuals that carry Cyp9k1_Dup11. Male samples are indicated in red.

	Dup 0	Dup 1	Dup 2	Dup 3	Dup 4	Dup 5	Dup 6	Dup 7	Dup 8	Dup 9	Dup 10	Dup 11	Dup 12	Dup 13	Dup 14	Dup 15	Dup 16
AA0072_C	0	0	0	0	0	0	0	0	0	0	0	2	0	0	0	0	0
AA0085_C	0	0	0	0	0	0	0	0	0	0	0	2	0	0	0	0	0
AA0133_C	0	0	0	0	0	0	0	0	0	0	0	2	0	0	0	2	0
AB0085_C	0	0	0	0	0	0	0	0	0	0	0	4	0	0	0	2	0
AB0103_C	0	0	0	0	0	0	0	0	0	0	0	4	0	0	0	0	0
AB0104_C	0	0	0	0	0	0	0	0	0	0	0	2	0	0	0	1	0
AB0108_C	0	0	0	0	0	0	0	0	0	0	0	2	0	0	0	0	0
AB0117_C	0	0	0	0	0	0	0	0	0	0	0	2	0	0	0	0	0
AB0119_C	0	0	0	0	0	0	0	0	0	0	0	2	0	0	0	0	0
AB0128_C	0	0	0	0	0	0	0	0	0	0	0	3	0	0	0	0	0
AB0134_C	0	0	0	0	1	0	0	0	0	0	0	2	0	0	0	0	0
AB0135_C	0	0	0	0	0	0	0	0	0	0	0	3	0	0	0	2	0
AB0136_C	0	0	0	0	0	0	0	0	0	0	0	4	0	0	0	0	0
AB0145_C	0	0	0	0	0	0	0	0	0	0	0	2	0	0	0	0	0
AB0146_C	0	0	0	0	0	0	0	0	0	0	0	3	0	0	0	2	0
AB0147_C	0	0	0	0	1	0	0	0	0	0	0	2	0	0	0	0	0
AB0151_C	0	0	0	0	0	0	0	0	0	0	0	2	0	0	0	0	0
AB0153_C	0	0	0	0	0	0	0	0	0	0	0	1	0	0	0	0	0
AB0159_C	0	0	0	0	0	0	0	0	0	0	0	2	0	0	0	0	0
AB0162_C	0	0	0	0	0	0	0	0	0	0	0	2	0	0	0	0	0
AB0171_C	0	0	0	0	0	0	0	0	0	0	0	2	0	0	0	2	0
AB0173_C	0	0	0	0	0	0	0	0	0	0	0	NA	0	0	0	2	0
AB0174_C	0	0	0	0	0	0	0	0	0	0	0	3	0	0	0	0	0
AB0177_C	0	0	0	0	0	0	0	0	0	0	0	2	0	0	0	1	0
AB0178_C	0	0	0	0	0	0	0	0	0	0	0	2	0	0	0	2	0
AB0179_C	0	0	0	0	0	0	0	0	0	0	0	2	0	0	0	2	0
AB0198_C	0	0	0	0	0	0	0	0	0	0	0	4	0	0	0	4	0
AB0199_C	0	0	0	0	0	0	0	0	0	0	0	2	0	0	0	2	0
AB0200_C	0	0	0	0	1	0	0	0	0	0	0	1	0	0	0	0	0
AB0202_C	0	0	0	0	0	0	0	0	0	0	0	2	0	0	0	2	0
AB0203_C	0	0	0	0	0	0	0	0	0	0	0	4	0	0	0	4	0
AB0205_C	0	0	0	0	0	0	0	0	0	0	0	2	0	0	0	5	0
AB0207_C	0	0	0	0	0	0	0	0	0	0	0	2	0	0	0	0	0
AB0208_C	0	0	0	0	0	0	0	0	0	0	0	5	0	0	0	0	0
AB0211_C	0	0	0	0	0	0	0	0	0	0	0	2	0	0	0	2	0
AB0217_C	0	0	0	0	0	0	0	0	0	0	0	4	0	0	0	0	0
AB0218_C	0	0	0	0	0	0	0	0	0	0	0	3	0	0	0	0	0
AB0228_C	0	0	0	0	0	0	0	0	0	0	0	2	0	0	0	2	0
AB0231_C	0	0	0	0	0	0	0	0	0	0	0	NA	0	0	0	5	0
AB0232_C	0	0	0	0	1	0	0	0	0	0	0	2	0	0	0	0	0
AB0233_C	0	0	0	0	0	0	0	0	0	0	0	3	0	0	0	0	0
AB0236_C	0	0	0	0	0	0	0	0	0	0	0	1	0	0	0	0	0
AB0239_C	0	0	0	0	0	0	0	0	0	0	0	2	0	0	0	2	0
AB0241_C	0	0	0	0	0	0	0	0	0	0	0	4	0	0	0	0	0
AB0244_C	0	0	0	0	0	0	0	0	0	0	0	2	0	0	0	1	0
AB0251_C	0	0	0	0	0	0	0	0	0	0	0	2	0	0	0	0	0
AB0252_C	0	0	0	0	0	0	0	0	0	0	0	2	0	0	0	0	0
AB0253_C	0	0	0	0	0	0	0	0	0	0	0	4	0	0	0	0	0
AB0255_C	0	0	0	0	0	0	0	0	0	0	0	4	0	2	0	0	0
AB0260_C	0	0	0	0	0	0	0	NA	0	0	0	NA	0	0	0	3	0
AB0261_C	0	0	0	0	0	0	0	0	0	0	0	4	0	0	0	2	0
AB0264_C	0	0	0	0	0	0	0	0	0	0	0	2	0	0	0	3	0
AB0265_C	0	0	0	0	0	0	0	0	0	0	0	1	0	0	0	6	0
AB0268_C	0	0	0	0	1	0	0	0	0	0	0	2	0	0	0	0	0
AB0271_C	0	0	0	0	0	0	0	0	0	0	0	4	0	0	0	0	0
AB0272_C	0	0	0	0	0	0	0	0	0	0	0	4	0	0	0	0	0
AB0273_C	0	0	0	0	0	0	0	NA	0	0	0	NA	0	0	0	5	0
AB0274_C	0	0	0	0	0	0	0	0	0	0	0	1	0	0	0	1	0
AB0277_C	0	0	0	0	0	0	0	0	0	0	0	3	0	0	0	0	0
AB0278_C	0	0	0	0	1	0	0	0	0	0	0	2	0	0	0	0	0
AB0283_C	0	0	0	0	0	0	0	0	0	0	0	1	0	0	0	3	0
AB0284_C	0	0	0	0	0	0	0	0	0	0	0	2	0	0	0	2	0
AN0027_C	0	0	0	0	0	0	0	0	0	0	0	1	0	0	0	0	0
AN0033_C	0	0	0	0	0	0	0	0	0	0	0	2	0	0	0	0	0
AN0198_C	0	0	0	0	0	0	0	0	0	0	0	1	0	0	0	0	0
AN0218_C	0	0	0	0	0	0	0	0	0	0	0	2	0	0	0	0	0
AV0001_C	0	0	0	0	0	0	0	0	0	0	0	3	0	0	0	0	0
AV0002_C	0	0	0	0	0	0	0	0	0	0	0	3	0	0	0	0	0
AV0003_C	0	0	0	0	0	0	0	0	0	0	0	2	0	0	0	0	0
AV0006_C	0	0	0	0	0	0	0	0	0	0	0	2	0	0	0	0	0
AV0009_C	0	0	0	0	0	0	0	0	0	0	0	0	0	0	0	5	0
AV0010_C	0	0	0	0	0	0	0	0	0	0	0	2	0	0	0	0	0
AV0011_C	0	0	0	0	0	0	0	0	0	0	0	2	0	0	0	0	0
AV0012_C	0	0	0	0	0	0	0	0	0	0	0	2	0	0	0	0	0
AV0014_C	0	0	0	0	0	0	0	0	0	0	0	1	0	0	0	0	0
AV0015_C	0	0	0	0	0	0	0	0	0	0	0	5	0	0	0	0	0
AV0017_C	0	0	0	0	0	0	0	0	0	0	0	2	0	0	0	0	0
AV0021_C	0	0	0	0	0	0	0	0	0	0	0	2	0	0	0	0	0
AV0025_C	0	0	0	0	0	0	0	0	0	0	0	2	0	0	0	0	0
AV0028_C	0	0	0	0	0	0	0	0	0	0	0	2	0	0	0	0	0
AV0030_C	0	0	0	0	0	0	0	0	0	0	0	0	0	0	0	0	0
AV0031_C	0	0	0	0	0	0	0	0	0	0	0	2	0	0	0	0	0
AV0032_C	0	0	0	0	0	0	0	0	0	0	0	2	0	0	0	0	0
AV0033_C	0	0	0	0	0	0	0	0	0	0	0	3	0	0	0	0	0
AV0034_C	0	0	0	0	0	0	0	0	0	0	0	4	0	0	0	0	0
AV0035_C	0	0	0	0	0	0	0	0	0	0	0	2	0	0	0	0	0
AV0037_C	0	0	0	0	0	0	0	0	0	0	0	2	0	0	0	0	0
AV0043_C	0	0	0	0	0	0	0	0	0	0	0	2	0	0	0	0	0
AV0044_C	0	0	0	0	0	0	0	0	0	0	0	2	0	0	0	1	0
AV0045_C	0	0	0	0	0	0	0	0	0	0	0	0	0	0	0	3	0

Duplication type 12

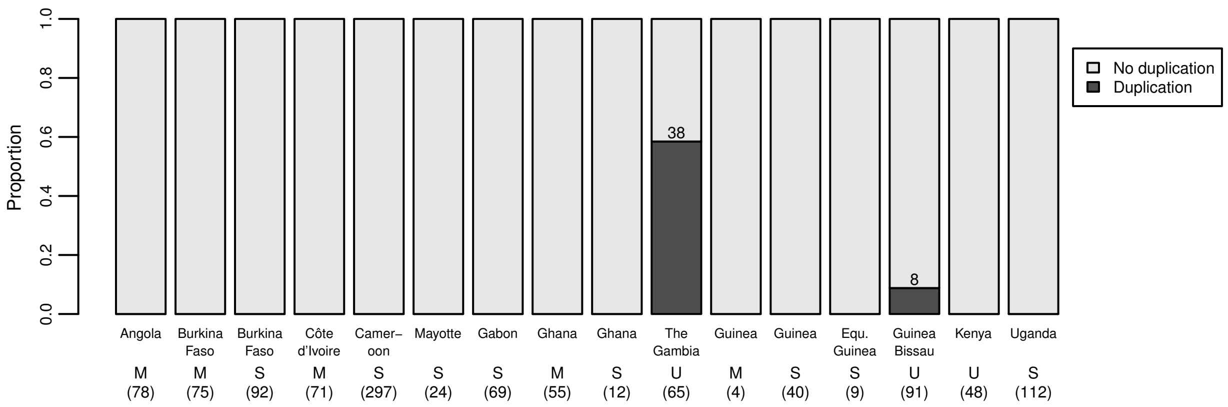

Fig. 9K1_S24: Barplot showing the proportion of samples that carry the Cyp9k1_Dup12 duplication in each of the Phase 2 populations. Numbers above the dark grey bars indicate the absolute number of samples carrying the duplication. S = *Anopheles gambiae*, M = *Anopheles coluzzi*, U = species undetermined. Numbers in brackets indicate the total number of samples from that population.

Cyp9k1_Dup12 was supported by face-away read pairs whose forward-facing read mapped in the interval 15234400 - 15234700 and whose reverse-facing read mapped in the interval 15244350 - 15244650 (Fig. **9K1_S25**). Cyp9k1_Dup12 was also supported by reads soft-clipped at the breakpoints (positions 15234434 and 15244702), with the clipped bases at each breakpoint aligning at the other breakpoint.

Cyp9k1_Dup12 breakpoint:

```
TGGGAACTGCTTGTGACAA G TACTATATAGGTACT
   end of the dup ^   ^ start of the dup
  position 15244700   position 15234436
```

The G could sit on either side of the breakpoint.

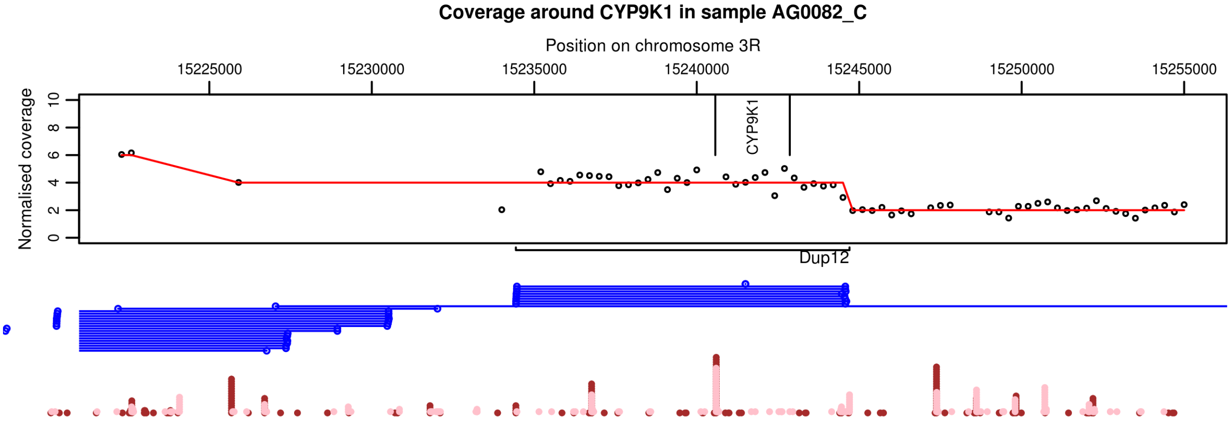

Fig. 9K1_S25: Example of coverage in an individual carrying the Cyp9k1_Dup12 duplication. Open black circles indicate coverage at each position. The red line shows the HMM estimation of the coverage state at each position. Black vertical lines represent the positions of the CYP9K1 gene. Pairs of blue points connected by lines indicate pairs of face-away reads. Reads soft-clipped before the alignment start point (dark brown points) and after the alignment end point (light brown points) are present at the start and end points of the duplication (clipped at positions 15234434 and 15244702 respectively). In each case, the clipped bases align to the other end of the duplication, as expected.

All samples with Cyp9k1_Dup12 were found in Guinea-Bissau and the Gambia, and all were female Estimates of copy number indicated that no samples had a copy number higher than 2 (Table 9K1_S12.2). Assuming that samples with a copy number of 1 are heterozygotes and that samples with a copy number of 2 are homozygotes for Cyp9k1_Dup12, the allele distribution expectations were consistent with HW expectations ($P = 1$ and $P = 0.79$ in Guinea-Bissau and the Gambia respectively).

Table 9K1_12.1: Coverage calls for Cyp9k1_Dup12. NAs were produced if coverage was too variable or if the duplication completely overlapped with another duplication whose coverage could also not be called.

copy number	AO col	BF col	BF gam	CI col	CM gam	FR gam	GA gam	GH col	GH gam	GM	GN col	GN gam	GQ gam	GW	KE	UG gam
0	78	75	92	71	297	24	69	55	12	27	4	40	9	83	48	112
1	0	0	0	0	0	0	0	0	0	29	0	0	0	8	0	0
2	0	0	0	0	0	0	0	0	0	9	0	0	0	0	0	0

Table 9K1_12.2: Coverage calls for all duplications in individuals that carry Cyp9k1_Dup12. Male samples are indicated in red.

	Dup 0	Dup 1	Dup 2	Dup 3	Dup 4	Dup 5	Dup 6	Dup 7	Dup 8	Dup 9	Dup 10	Dup 11	Dup 12	Dup 13	Dup 14	Dup 15	Dup 16
AG0082_C	0	0	0	0	0	0	0	0	0	0	0	0	2	0	0	0	0
AG0085_C	0	0	0	0	0	0	0	0	0	0	0	0	1	0	0	0	0
AG0089_C	0	0	0	0	0	0	0	0	0	0	0	0	2	0	0	0	0
AG0100_C	0	0	0	0	0	0	0	0	0	0	0	0	1	0	0	0	0
AG0102_C	0	0	0	0	0	0	0	0	0	0	0	0	1	0	0	0	0
AG0108_C	0	0	0	0	0	0	0	0	0	0	0	0	1	0	0	0	0
AG0120_C	0	0	0	0	0	0	0	0	0	0	0	0	1	0	0	0	0
AG0123_C	0	0	0	0	0	0	0	0	0	0	0	0	1	0	0	0	0
AG0125_C	0	0	0	0	0	0	0	0	0	0	0	0	1	0	0	0	0
AG0126_C	0	0	0	0	0	0	0	0	0	0	0	0	1	0	0	0	0
AG0127_C	0	0	0	0	0	0	0	0	0	0	0	0	1	0	0	0	0
AG0129_C	0	0	0	0	0	0	0	0	0	0	0	0	1	0	0	0	0
AG0134_C	0	0	0	0	0	0	0	0	0	0	0	0	1	0	0	0	0
AG0137_C	0	0	0	0	0	0	0	0	0	0	0	0	1	0	0	0	0
AG0138_C	0	0	0	0	0	0	0	0	0	0	0	0	1	0	0	0	0
AG0139_C	0	0	0	0	0	0	0	0	0	0	0	0	1	0	0	0	0
AG0143_C	0	0	0	0	0	0	0	0	0	0	0	0	1	0	0	0	0
AG0144_C	0	0	0	0	0	0	0	0	0	0	0	0	2	0	0	0	0
AG0148_C	0	0	0	0	0	0	0	0	0	0	0	0	1	0	0	0	0
AG0152_C	0	0	0	0	0	0	0	0	0	0	0	0	1	0	0	0	0
AG0153_C	0	0	0	0	0	0	0	0	0	0	0	0	1	0	0	0	0
AG0156_C	0	0	0	0	0	0	0	0	0	0	0	0	1	0	0	0	0
AG0162_C	0	0	0	0	0	0	0	0	0	0	0	0	2	0	0	0	0
AG0169_C	0	0	0	0	0	0	0	0	0	0	0	0	1	0	0	0	0
AG0170_C	0	0	0	0	0	0	0	0	0	0	0	0	1	0	0	0	0
AG0178_C	0	0	0	0	0	0	0	0	0	0	0	0	1	0	0	0	0
AG0197_C	0	0	0	0	0	0	0	0	0	0	0	0	1	0	0	0	0
AG0202_C	0	0	0	0	0	0	0	0	0	0	0	0	1	0	0	0	0
AG0204_C	0	0	0	0	0	0	0	0	0	0	0	0	1	0	0	0	0
AG0206_C	0	0	0	0	0	0	0	0	0	0	0	0	1	0	0	0	0
AG0208_C	0	0	0	0	0	0	0	0	0	0	0	0	1	0	0	0	0
AG0214_C	0	0	0	0	0	0	0	0	0	0	0	0	2	0	0	0	0
AG0221_C	0	0	0	0	0	0	0	0	0	0	0	0	2	0	0	0	0
AG0223_C	0	0	0	0	0	0	0	0	0	0	0	0	2	0	0	0	0
AG0227_C	0	0	0	0	0	0	0	0	0	0	0	0	2	0	0	0	0
AG0230_C	0	0	0	0	0	0	0	0	0	0	0	0	1	0	0	0	0
AG0231_C	0	0	0	0	0	0	0	0	0	0	0	0	1	0	0	0	0
AG0232_C	0	0	0	0	0	0	0	0	0	0	0	0	2	0	0	0	0
AJ0074_C	0	0	0	0	0	0	0	0	0	0	0	0	1	0	0	0	0
AJ0086_C	0	0	0	0	0	0	0	0	0	0	0	0	1	0	0	0	0
AJ0087_C	0	0	0	0	0	0	0	0	0	0	0	0	1	0	0	0	0
AJ0088_C	0	0	0	0	0	0	0	0	0	0	0	0	1	0	0	0	0
AJ0092_C	0	0	0	0	0	0	0	0	0	0	0	0	1	0	0	0	0
AJ0139_C	0	0	0	0	0	0	0	0	0	0	0	0	1	0	0	0	0
AJ0140_C	0	0	0	0	0	0	0	0	0	0	0	0	1	0	0	0	0
AJ0154_C	0	0	0	0	0	0	0	0	0	0	0	0	1	0	0	0	0

Duplication type 13

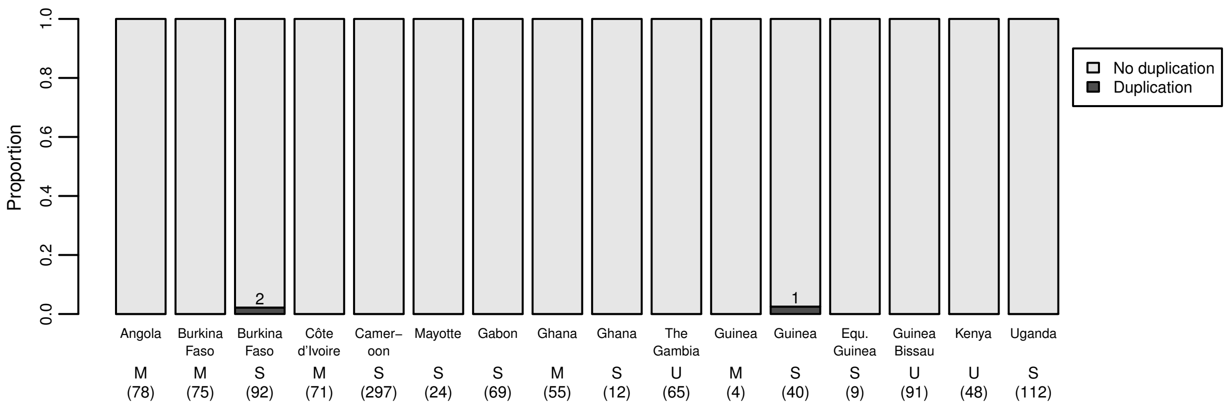

Fig. 9K1_S26: Barplot showing the proportion of samples that carry the Cyp9k1_Dup13 duplication in each of the Phase 2 populations. Numbers above the dark grey bars indicate the absolute number of samples carrying the duplication. S = *Anopheles gambiae*, M = *Anopheles coluzzi*, U = species undetermined. Numbers in brackets indicate the total number of samples from that population.

Cyp9k1_Dup13 was supported by face-away read pairs whose forward-facing read mapped in the interval 15240100 - 15240400 and whose reverse-facing read mapped in the interval 15250250 - 15250550 (Fig. **9K1_S27**). Cyp9k1_Dup13 was also supported by reads soft-clipped at the breakpoints (positions 15240067 and 15250575), with the clipped bases at each breakpoint aligning at the other breakpoint.

Cyp9k1_Dup13 breakpoint:

```
TTAGACATGGTAAAATCC GA GTGTAAGGCAATTTTAA
  end of the dup ^    ^ start of the dup
 position 15250572    position 15240070
```

The `GA` could sit on either side of the breakpoint.

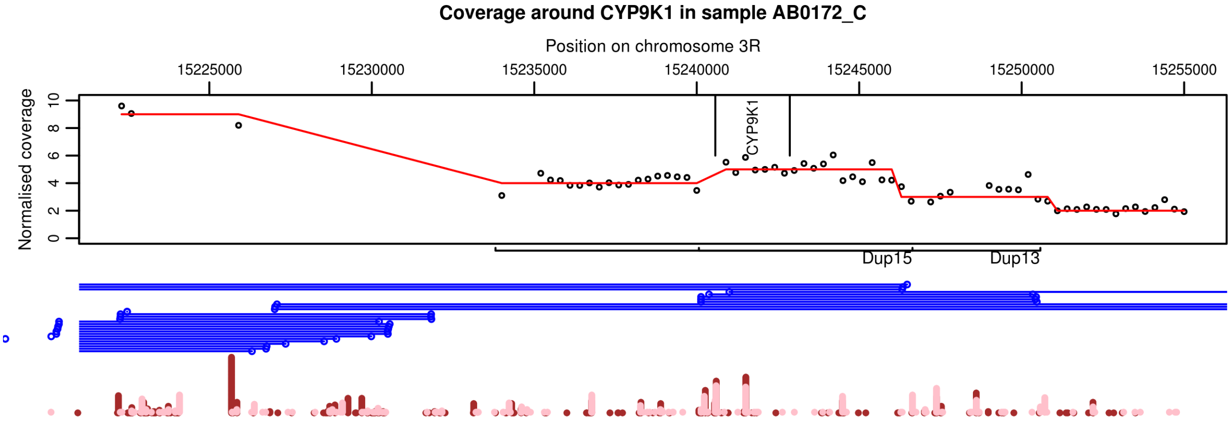

Fig. 9K1_S27: Example coverage in an individual carrying the Cyp9k1_Dup13 duplication. Open black circles indicate coverage at each position. The red line shows the HMM estimation of the coverage state at each position. Black vertical lines represent the positions of the CYP9K1 gene. Pairs of blue points connected by lines indicate pairs of face-away reads. Reads soft-clipped before the alignment start point (dark brown points) and after the alignment end point (light brown points) are present at the start and end points of the duplication (clipped at positions 15240067 and 15250575 respectively). In each case, the clipped bases align to the other end of the duplication, as expected.

The three samples that carry Cyp9k1_Dup13 are all females (Tables 9K1_S13.1 & 13.2). Estimates of copy number indicate that two of these samples have a copy number of 2 and one has a copy number of 1. However, visual inspection of these samples suggests that the estimates of 2 are errors caused by a small duplication at the end of the range of Cyp9k1_Dup13 (which does not overlap with CYP9K1).

Table 9K1_13.1: Coverage calls for Cyp9k1_Dup13. NAs were produced if coverage was too variable or if the duplication completely overlapped with another duplication whose coverage could also not be called.

copy number	AO col	BF col	BF gam	CI col	CM gam	FR gam	GA gam	GH col	GH gam	GM	GN col	GN gam	GQ gam	GW	KE	UG gam
0	78	75	90	71	297	24	69	55	12	65	4	39	9	91	48	112
1	0	0	1	0	0	0	0	0	0	0	0	0	0	0	0	0
2	0	0	1	0	0	0	0	0	0	0	0	1	0	0	0	0

Table 9K1_13.2: Coverage calls for all duplications in individuals that carry Cyp9k1_Dup13. Male samples are indicated in red.

	Dup 0	Dup 1	Dup 2	Dup 3	Dup 4	Dup 5	Dup 6	Dup 7	Dup 8	Dup 9	Dup 10	Dup 11	Dup 12	Dup 13	Dup 14	Dup 15	Dup 16
AB0172_C	0	0	0	0	0	0	0	0	0	0	0	0	0	1	0	2	0
AB0255_C	0	0	0	0	0	0	0	0	0	0	0	4	0	2	0	0	0
AV0029_C	0	0	0	0	0	0	0	0	0	0	0	0	0	2	0	2	0

Duplication type 14

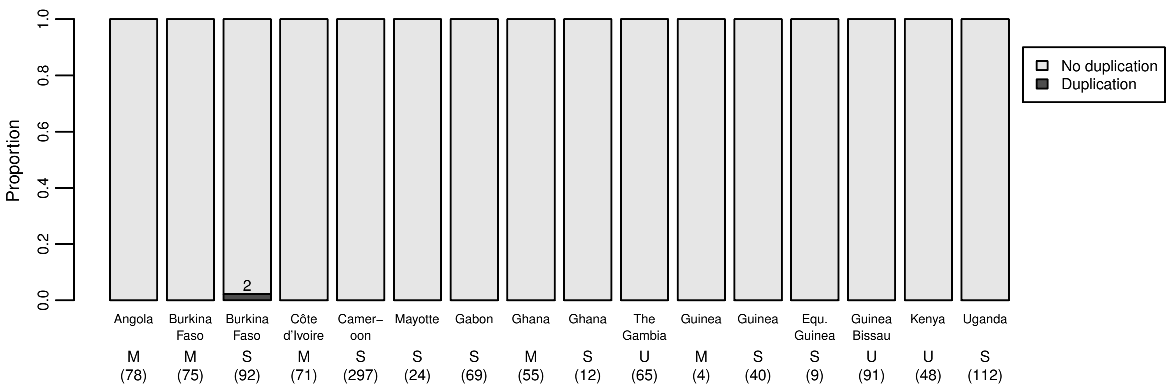

Fig. 9K1_S28: Barplot showing the proportion of samples that carry the Cyp9k1_Dup14 duplication in each of the Phase 2 populations. Numbers above the dark grey bars indicate the absolute number of samples carrying the duplication. S = *Anopheles gambiae*, M = *Anopheles coluzzi*, U = species undetermined. Numbers in brackets indicate the total number of samples from that population.

Cyp9k1_Dup14 was supported by face-away read pairs whose forward-facing read mapped in the interval 15244200 - 15244500 and whose reverse-facing read mapped in the interval 9676400 - 9676700 (Fig. **9K1_S29**). These face-away reads are unlikely to indicate the true extent of the duplication, as they span 5.5Mbp. Reads were found that were soft-clipped after the end of the duplication (position 15244936), with the clipped bases mapping to two positions in the same region as the face-away reads: either position 9676277 or 9676356 (these two positions are reverse complements of each other). Interestingly, these clipped bases are the same as those found in Cyp9k1_Dup15.

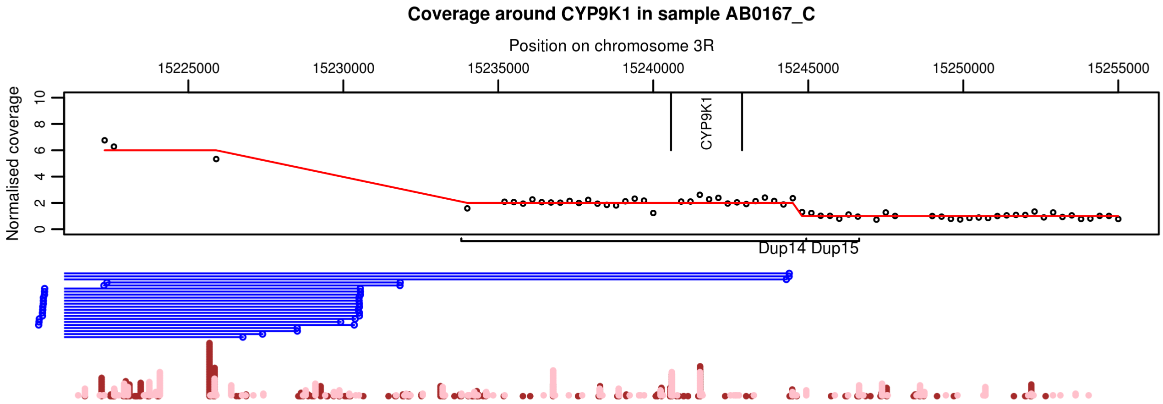

Fig. 9K1_S29: Example of coverage in an individual carrying the dup15 duplication. Open black circles indicate coverage at each position. The red line shows the HMM estimation of the coverage state at each position. Black vertical lines represent the positions of the CYP9K1 gene. Pairs of blue points connected by lines indicate pairs of face-away reads. Reads soft-clipped after the alignment end point (light brown points) are present at the end point of the duplication (clipped at position 15244936). Dark brown points indicate reads soft-clipped before the alignment start point. The alignment start point could not be determined accurately and the extent of the duplication represented here is not necessarily correct.

Cyp9k1_Dup14 was found in only two samples, one of which was male and the other female (Table 9K1_S14.1 & 14.2). Estimates of copy number in these samples indicated that the male had a copy number of 1 and the female had a copy number of 2 (Table 9K1_S14.2). Both of these samples also carried Cyp9k1_Dup15, which overlaps with Cyp9k1_Dup14 for a large part of its range, making the respective copy numbers of these two CNVs difficult to call (Table 9K1_S14.2).

Table 9K1_14.1: Coverage calls for Cyp9k1_Dup14. NAs were produced if coverage was too variable or if the duplication completely overlapped with another duplication whose coverage could also not be called.

copy number	AO col	BF col	BF gam	CI col	CM gam	FR gam	GA gam	GH col	GH gam	GM	GN col	GN gam	GQ gam	GW	KE	UG gam
0	78	75	90	71	297	24	69	55	12	65	4	40	9	91	48	112
1	0	0	1	0	0	0	0	0	0	0	0	0	0	0	0	0
2	0	0	1	0	0	0	0	0	0	0	0	0	0	0	0	0

Table 9K1_14.2: Coverage calls for all duplications in individuals that carry Cyp9k1_Dup14. Male samples are indicated in red.

	Dup 0	Dup 1	Dup 2	Dup 3	Dup 4	Dup 5	Dup 6	Dup 7	Dup 8	Dup 9	Dup 10	Dup 11	Dup 12	Dup 13	Dup 14	Dup 15	Dup 16
AB0167_C	0	0	0	0	0	0	0	0	0	0	0	0	0	0	1	0	0
AB0206_C	0	0	0	0	0	0	0	0	0	0	0	0	0	0	2	3	0

Duplication type 15

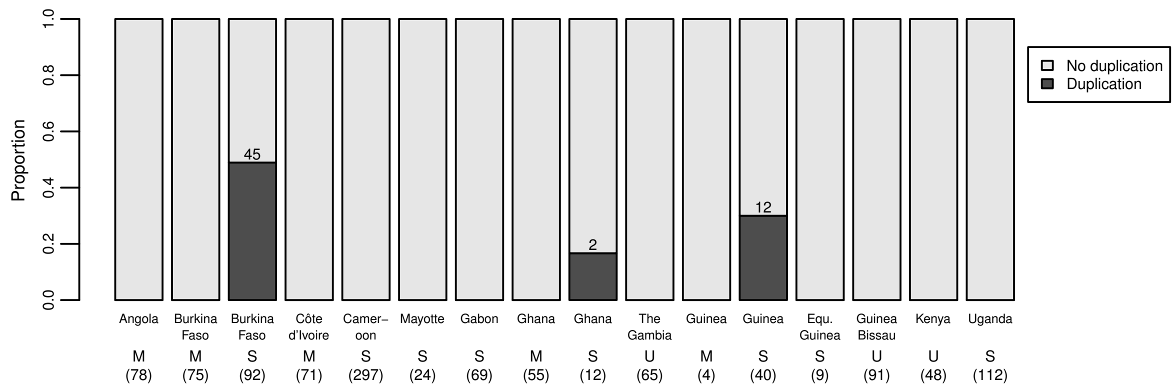

Fig. 9K1_S30: Barplot showing the proportion of samples that carry the Cyp9k1_Dup15 duplication in each of the Phase 2 populations. Numbers above the dark grey bars indicate the absolute number of samples carrying the duplication. S = *Anopheles gambiae*, M = *Anopheles coluzzi*, U = species undetermined. Numbers in brackets indicate the total number of samples from that population.

Cyp9k1_Dup15 was supported by face-away read pairs whose forward-facing read mapped in the interval 15246250 - 15246550 and whose reverse-facing read mapped in the interval 9676400 - 9676700 (**Fig. 9K1_S31**). These face-away reads are unlikely to indicate the true extent of the duplication, as they span 5.5Mbp. Reads were found that were soft-clipped after the end of the duplication (position 15246640), with the clipped bases mapping to two positions in the same region as the face-away reads: either position 9676277 or 9676537 (these two positions are reverse complements of each other).

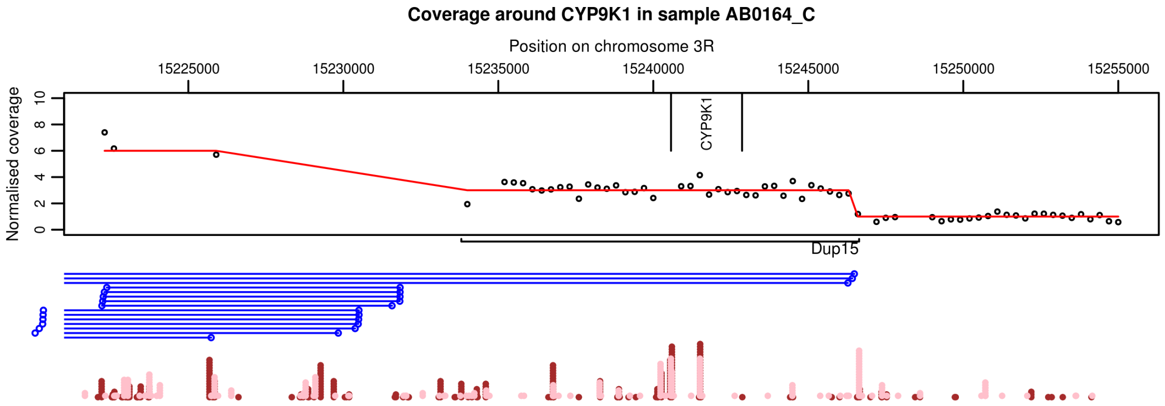

Fig. 9K1_S31: Example of coverage in an individual carrying the Cyp9k1_Dup15 duplication. Open black circles indicate coverage at each position. The red line shows the HMM estimation of the coverage state at each position. Black vertical lines represent the positions of the CYP9K1 gene. Pairs of blue points connected by lines indicate pairs of face-away reads. Reads soft-clipped after the alignment end point (light brown points) are present at the end point of the duplication (clipped at position 15246640). Dark brown points indicate reads soft-clipped before the alignment start point. The alignment start point could not be determined accurately and the extent of the duplication represented here is not necessarily correct.

Cyp9k1_Dup15 was found *An. gambiae* from Burkina Faso, Ghana and Guinea (Table 9K1_S15.1). Estimates of copy number were as high as 6 in females and 2 in males (Table 9K1_S15.2), indicating that both simple duplications and higher-level CNVs exist for Cyp9k1_Dup15. It was therefore impossible to make confident genotype calls for Cyp9k1_Dup15.

Table 9K1_15.1: Coverage calls for Cyp9k1_Dup15. NAs were produced if coverage was too variable or if the duplication completely overlapped with another duplication whose coverage could also not be called.

copy number	AO col	BF col	BF gam	CI col	CM gam	FR gam	GA gam	GH col	GH gam	GM	GN col	GN gam	GQ gam	GW	KE	UG gam
NA	0	0	2	0	0	0	0	0	0	0	0	0	0	0	0	0
0	78	75	48	71	297	24	69	55	10	65	4	28	9	91	48	112
1	0	0	5	0	0	0	0	0	1	0	0	2	0	0	0	0
2	0	0	21	0	0	0	0	0	1	0	0	5	0	0	0	0
3	0	0	5	0	0	0	0	0	0	0	0	4	0	0	0	0
4	0	0	5	0	0	0	0	0	0	0	0	0	0	0	0	0
5	0	0	3	0	0	0	0	0	0	0	0	1	0	0	0	0
6	0	0	3	0	0	0	0	0	0	0	0	0	0	0	0	0

Table 9K1_15.2: Coverage calls for all duplications in individuals that carry Cyp9k1_Dup15. Male samples are indicated in red.

	Dup 0	Dup 1	Dup 2	Dup 3	Dup 4	Dup 5	Dup 6	Dup 7	Dup 8	Dup 9	Dup 10	Dup 11	Dup 12	Dup 13	Dup 14	Dup 15	Dup 16
AA0048_C	0	0	0	0	0	0	0	0	0	0	0	0	0	0	0	1	0
AA0133_C	0	0	0	0	0	0	0	0	0	0	0	2	0	0	0	2	0
AB0085_C	0	0	0	0	0	0	0	0	0	0	0	4	0	0	0	2	0
AB0104_C	0	0	0	0	0	0	0	0	0	0	0	2	0	0	0	1	0
AB0118_C	0	0	0	0	0	0	0	0	0	0	0	0	0	0	0	2	0
AB0135_C	0	0	0	0	0	0	0	0	0	0	0	3	0	0	0	2	0
AB0146_C	0	0	0	0	0	0	0	0	0	0	0	3	0	0	0	2	0
AB0148_C	0	0	0	0	0	0	0	0	0	0	0	0	0	0	0	3	0
AB0160_C	0	0	0	0	0	0	0	0	0	0	0	0	0	0	0	1	0
AB0164_C	0	0	0	0	0	0	0	0	0	0	0	0	0	0	0	2	0
AB0165_C	0	0	0	0	0	0	0	0	0	0	0	0	0	0	0	2	0
AB0166_C	0	0	0	0	0	0	0	0	0	0	0	0	0	0	0	2	0
AB0167_C	0	0	0	0	0	0	0	0	0	0	0	0	0	0	1	0	0
AB0171_C	0	0	0	0	0	0	0	0	0	0	0	2	0	0	0	2	0
AB0172_C	0	0	0	0	0	0	0	0	0	0	0	0	0	1	0	2	0
AB0173_C	0	0	0	0	0	0	0	0	0	0	0	NA	0	0	0	2	0
AB0175_C	0	0	0	0	0	0	0	0	0	0	0	0	0	0	0	6	0
AB0176_C	0	0	0	0	0	0	0	0	0	0	0	0	0	0	0	4	0
AB0177_C	0	0	0	0	0	0	0	0	0	0	0	2	0	0	0	1	0
AB0178_C	0	0	0	0	0	0	0	0	0	0	0	2	0	0	0	2	0
AB0179_C	0	0	0	0	0	0	0	0	0	0	0	2	0	0	0	2	0
AB0197_C	0	0	0	0	0	0	0	NA	0	0	0	0	0	0	0	NA	0
AB0198_C	0	0	0	0	0	0	0	0	0	0	0	4	0	0	0	4	0
AB0199_C	0	0	0	0	0	0	0	0	0	0	0	2	0	0	0	2	0
AB0201_C	0	0	0	0	0	0	0	0	0	0	0	0	0	0	0	4	0
AB0202_C	0	0	0	0	0	0	0	0	0	0	0	2	0	0	0	2	0
AB0203_C	0	0	0	0	0	0	0	0	0	0	0	4	0	0	0	4	0
AB0205_C	0	0	0	0	0	0	0	0	0	0	0	2	0	0	0	5	0
AB0206_C	0	0	0	0	0	0	0	0	0	0	0	0	0	0	2	3	0
AB0211_C	0	0	0	0	0	0	0	0	0	0	0	2	0	0	0	2	0
AB0228_C	0	0	0	0	0	0	0	0	0	0	0	2	0	0	0	2	0
AB0231_C	0	0	0	0	0	0	0	0	0	0	0	NA	0	0	0	5	0
AB0239_C	0	0	0	0	0	0	0	0	0	0	0	2	0	0	0	2	0
AB0244_C	0	0	0	0	0	0	0	0	0	0	0	2	0	0	0	1	0
AB0256_C	0	0	0	0	1	0	0	0	0	0	0	0	0	0	0	4	0
AB0260_C	0	0	0	0	0	0	0	NA	0	0	0	NA	0	0	0	3	0
AB0261_C	0	0	0	0	0	0	0	0	0	0	0	4	0	0	0	2	0
AB0264_C	0	0	0	0	0	0	0	0	0	0	0	2	0	0	0	3	0
AB0265_C	0	0	0	0	0	0	0	0	0	0	0	1	0	0	0	6	0
AB0270_C	0	0	0	0	1	0	0	0	0	0	0	0	0	0	0	2	0
AB0273_C	0	0	0	0	0	0	0	NA	0	0	0	NA	0	0	0	5	0
AB0274_C	0	0	0	0	0	0	0	0	0	0	0	1	0	0	0	1	0
AB0275_C	0	0	0	0	NA	0	0	0	0	0	0	0	0	0	0	NA	0
AB0280_C	0	0	0	0	1	0	0	0	0	0	0	0	0	0	0	2	0
AB0281_C	0	0	0	0	0	0	0	0	0	0	0	0	0	0	0	6	0
AB0283_C	0	0	0	0	0	0	0	0	0	0	0	1	0	0	0	3	0
AB0284_C	0	0	0	0	0	0	0	0	0	0	0	2	0	0	0	2	0
AV0005_C	0	0	0	0	0	0	0	0	0	0	0	0	0	0	0	3	0
AV0008_C	0	0	0	0	0	0	0	0	0	0	0	0	0	0	0	3	0
AV0009_C	0	0	0	0	0	0	0	0	0	0	0	0	0	0	0	5	0
AV0013_C	0	0	0	0	0	0	0	0	0	0	0	0	0	0	0	2	0
AV0020_C	0	0	0	0	0	0	0	0	0	0	0	0	0	0	0	2	0
AV0022_C	0	0	0	0	0	0	0	0	0	0	0	0	0	0	0	2	0
AV0026_C	0	0	0	0	0	0	0	0	0	0	0	0	0	0	0	1	0
AV0029_C	0	0	0	0	0	0	0	0	0	0	0	0	0	2	0	2	0
AV0036_C	0	0	0	0	1	0	0	0	0	0	0	0	0	0	0	2	0
AV0041_C	0	0	0	0	0	0	0	0	0	0	0	0	0	0	0	3	0
AV0044_C	0	0	0	0	0	0	0	0	0	0	0	2	0	0	0	1	0
AV0045_C	0	0	0	0	0	0	0	0	0	0	0	0	0	0	0	3	0

Duplication type 16

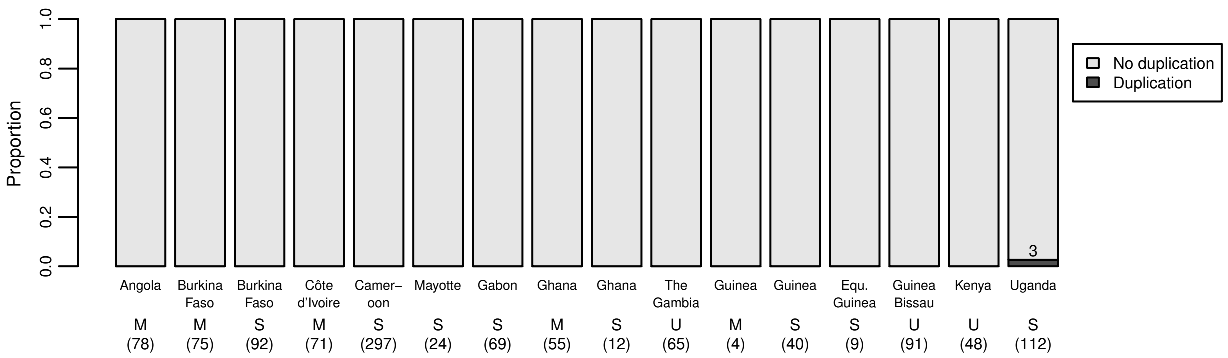

Fig. 9K1_S32: Barplot showing the proportion of samples that carry the Cyp9k1_Dup16 duplication in each of the Phase 2 populations. Numbers above the dark grey bars indicate the absolute number of samples carrying the duplication. S = *Anopheles gambiae*, M = *Anopheles coluzzi*, U = species undetermined. Numbers in brackets indicate the total number of samples from that population.

Cyp9k1_Dup16 was supported by face-away read pairs whose forward-facing read mapped in the interval 15222700 - 15223000 and whose reverse-facing read mapped in the interval 15244450 - 15244750 (Fig. **9K1_S33**). Cyp9k1_Dup16 was also supported by reads soft-clipped at the end of the duplication (position 15244755), with the clipped bases aligning at 15222810. No reads were found that were soft-clipped at position 15222810, but this may be because the reads that spanned the breakpoint mapped more easily at the end than at the beginning.

Cyp9k1_Dup16 breakpoint:

```
ACGCCCGTACCGGCTGGGCAGTA   AAGTAAAGTAAA   GTAAAGTATGGATGTAGTTTTTAA
        end of the dup ^  inserted seq   ^ start of the dup
      position 15244754                  position 15222810
```

The sequence AAGTAAAGTAAA is inserted between the sequences on either side of the breakpoint.

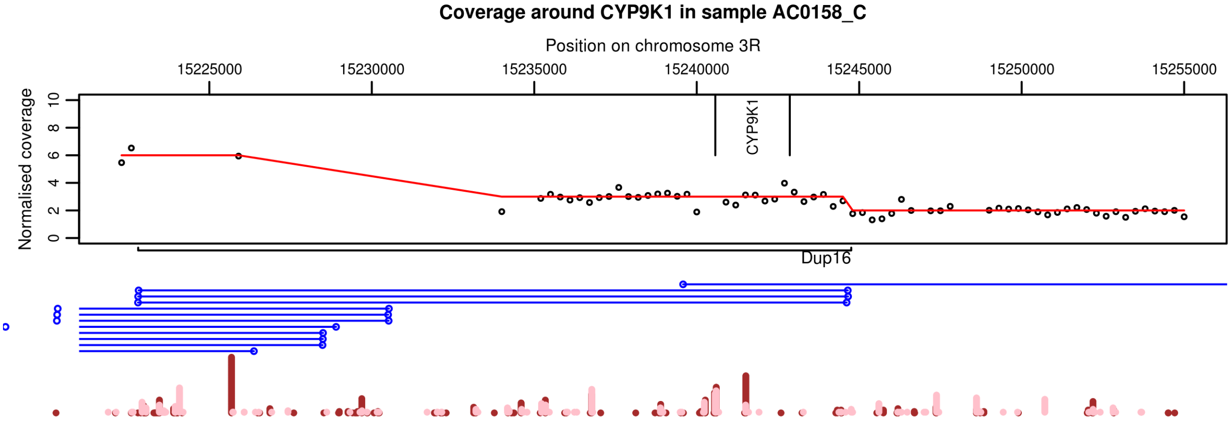

Fig. 9K1_S33: Example of coverage in an individual carrying the Cyp9k1_Dup16 duplication. Open black circles indicate coverage at each position. The red line shows the HMM estimation of the coverage state at each position. Black vertical lines represent the positions of the CYP9K1 gene. Pairs of blue points connected by lines indicate pairs of face-away reads. Reads soft-clipped after the alignment end point (light brown points) are present at the end point of the duplication (clipped at position 15244755). Dark brown points indicate reads soft-clipped before the alignment start point.

The three samples that carry Cyp9k1_Dup16 all have a copy number of 1 (Tables 9K1_S16.1 & 16.2). Cyp9k1_Dup16 is therefore a single copy duplication and all three samples are heterozygous.

Table 9K1_16.1: Coverage calls for Cyp9k1_Dup16. NAs were produced if coverage was too variable or if the duplication completely overlapped with another duplication whose coverage could also not be called.

copy number	AO col	BF col	BF gam	CI col	CM gam	FR gam	GA gam	GH col	GH gam	GM	GN col	GN gam	GQ gam	GW	KE	UG gam
0	78	75	92	71	297	24	69	55	12	65	4	40	9	91	48	109
1	0	0	0	0	0	0	0	0	0	0	0	0	0	0	0	3

Table 9K1_16.2: Coverage calls for all duplications in individuals that carry Cyp9k1_Dup16. Male samples are indicated in red.

	Dup 0	Dup 1	Dup 2	Dup 3	Dup 4	Dup 5	Dup 6	Dup 7	Dup 8	Dup 9	Dup 10	Dup 11	Dup 12	Dup 13	Dup 14	Dup 15	Dup 16
AC0100_C	0	0	0	0	0	0	0	0	0	0	0	0	0	0	0	0	1
AC0127_C	0	0	0	0	0	0	0	0	0	0	0	0	0	0	0	0	1
AC0158_C	0	0	0	0	0	0	0	0	0	0	0	0	0	0	0	0	1

Table 9K1_S17: Coverage calls for all duplications around Cyp9k1 in all individuals. Male samples are indicated in red.

	Dup 0	Dup 1	Dup 2	Dup 3	Dup 4	Dup 5	Dup 6	Dup 7	Dup 8	Dup 9	Dup 10	Dup 11	Dup 12	Dup 13	Dup 14	Dup 15	Dup 16
AA0040_C	0	0	0	0	0	0	0	0	0	0	0	0	0	0	0	0	0
AA0041_C	0	0	0	0	0	0	0	0	0	0	0	0	0	0	0	0	0
AA0042_C	0	0	0	0	0	0	0	0	0	0	0	0	0	0	0	0	0
AA0043_C	0	0	0	0	0	0	0	0	0	0	0	0	0	0	0	0	0
AA0044_C	0	0	0	0	0	0	0	0	0	0	0	0	0	0	0	0	0
AA0048_C	0	0	0	0	0	0	0	0	0	0	0	0	0	0	0	1	0
AA0049_C	0	0	0	0	0	0	0	0	0	0	0	0	0	0	0	0	0
AA0050_C	0	0	0	0	0	0	0	0	0	0	0	0	0	0	0	0	0
AA0051_C	0	0	0	0	0	0	0	0	0	0	0	0	0	0	0	0	0
AA0052_C	0	0	0	0	0	0	0	0	0	0	0	0	0	0	0	0	0
AA0053_C	0	0	0	0	0	0	0	0	0	0	0	0	0	0	0	0	0
AA0054_C	0	0	0	0	0	0	0	0	0	0	0	0	0	0	0	0	0
AA0055_C	0	0	0	0	0	0	0	0	0	0	0	0	0	0	0	0	0
AA0056_C	0	0	0	0	0	0	0	0	0	0	0	0	0	0	0	0	0
AA0060_C	0	0	0	0	1	0	0	0	0	0	0	0	0	0	0	0	0
AA0061_C	0	0	0	0	0	0	0	0	0	0	0	0	0	0	0	0	0
AA0063_C	0	0	0	0	0	0	0	0	0	0	0	0	0	0	0	0	0
AA0064_C	0	0	0	0	0	0	0	0	0	0	0	0	0	0	0	0	0
AA0066_C	0	0	0	0	0	0	0	0	0	0	0	0	0	0	0	0	0
AA0067_C	0	0	0	0	0	0	0	0	0	0	0	0	0	0	0	0	0
AA0068_C	0	0	0	0	0	0	0	0	0	0	0	0	0	0	0	0	0
AA0072_C	0	0	0	0	0	0	0	0	0	0	0	2	0	0	0	0	0
AA0073_C	0	0	0	0	0	0	0	0	0	0	0	0	0	0	0	0	0
AA0074_C	0	0	0	0	0	0	0	0	0	0	1	0	0	0	0	0	0
AA0075_C	0	0	0	0	0	0	0	0	0	0	0	0	0	0	0	0	0
AA0076_C	0	0	0	0	0	0	0	0	0	0	0	0	0	0	0	0	0
AA0077_C	0	0	0	0	0	0	0	0	0	0	0	0	0	0	0	0	0
AA0080_C	0	0	0	0	0	0	0	0	0	0	0	0	0	0	0	0	0
AA0084_C	0	0	0	0	0	0	0	0	0	0	0	0	0	0	0	0	0
AA0085_C	0	0	0	0	0	0	0	0	0	0	0	2	0	0	0	0	0
AA0086_C	0	0	0	0	0	0	0	0	0	0	0	0	0	0	0	0	0
AA0087_C	0	0	0	0	0	0	0	0	0	0	0	0	0	0	0	0	0
AA0088_C	0	0	0	0	0	0	0	0	0	0	0	0	0	0	0	0	0
AA0089_C	0	0	0	0	0	0	0	0	0	0	0	0	0	0	0	0	0
AA0090_C	0	0	0	0	0	0	0	0	0	0	0	0	0	0	0	0	0
AA0091_C	0	0	0	0	0	0	0	0	0	0	0	0	0	0	0	0	0
AA0096_C	0	0	0	0	0	0	0	0	0	0	0	0	0	0	0	0	0
AA0097_C	0	0	0	0	0	0	0	0	0	0	0	0	0	0	0	0	0
AA0098_C	0	0	0	0	0	0	0	0	0	0	0	0	0	0	0	0	0
AA0099_C	0	0	0	0	0	0	0	0	0	0	0	0	0	0	0	0	0
AA0100_C	0	0	0	0	0	0	0	0	0	0	0	0	0	0	0	0	0
AA0101_C	0	0	0	0	0	0	0	0	0	0	0	0	0	0	0	0	0
AA0102_C	0	0	0	0	0	0	0	0	0	0	0	0	0	0	0	0	0
AA0103_C	0	0	0	0	0	0	0	0	0	0	0	0	0	0	0	0	0
AA0104_C	0	0	0	0	0	0	0	0	0	0	0	0	0	0	0	0	0
AA0107_C	0	0	0	0	0	0	0	0	0	0	0	0	0	0	0	0	0
AA0108_C	0	0	0	0	0	0	0	0	0	0	0	0	0	0	0	0	0
AA0109_C	0	0	0	0	0	0	0	0	0	0	0	0	0	0	0	0	0
AA0110_C	0	0	0	0	0	0	0	0	0	0	0	0	0	0	0	0	0
AA0111_C	0	0	0	0	0	0	0	0	0	0	0	0	0	0	0	0	0
AA0113_C	0	0	0	0	0	0	0	0	0	0	0	0	0	0	0	0	0
AA0114_C	0	0	0	0	0	0	0	0	0	0	0	0	0	0	0	0	0
AA0115_C	0	0	0	0	0	0	0	0	0	0	0	0	0	0	0	0	0
AA0116_C	0	0	0	0	0	0	0	0	0	0	0	0	0	0	0	0	0
AA0122_C	0	0	0	0	1	0	0	0	0	0	0	0	0	0	0	0	0
AA0123_C	0	0	0	0	0	0	0	0	0	0	0	0	0	0	0	0	0
AA0124_C	0	0	0	0	0	0	0	0	0	0	0	0	0	0	0	0	0
AA0125_C	0	0	0	0	0	0	0	0	0	0	0	0	0	0	0	0	0
AA0127_C	0	0	0	0	0	0	0	0	0	0	0	0	0	0	0	0	0
AA0132_C	0	0	0	0	0	0	0	0	0	0	1	0	0	0	0	0	0
AA0133_C	0	0	0	0	0	0	0	0	0	0	0	2	0	0	0	2	0
AA0134_C	0	0	0	0	0	0	0	0	0	0	1	0	0	0	0	0	0
AA0135_C	0	0	0	0	0	0	0	0	0	0	0	0	0	0	0	0	0
AA0136_C	0	0	0	0	0	0	0	0	0	0	0	0	0	0	0	0	0
AA0139_C	0	0	0	0	0	0	0	0	0	0	0	0	0	0	0	0	0

	Dup 0	Dup 1	Dup 2	Dup 3	Dup 4	Dup 5	Dup 6	Dup 7	Dup 8	Dup 9	Dup 10	Dup 11	Dup 12	Dup 13	Dup 14	Dup 15	Dup 16
AA0140_C	0	0	0	0	0	0	0	0	0	0	0	0	0	0	0	0	0
AA0141_C	0	0	0	0	0	0	0	0	0	0	0	0	0	0	0	0	0
AB0085_C	0	0	0	0	0	0	0	0	0	0	0	4	0	0	0	2	0
AB0087_C	0	0	0	0	0	0	0	0	0	0	2	0	0	0	0	0	0
AB0088_C	0	0	0	0	0	0	0	0	0	0	0	0	0	0	0	0	0
AB0089_C	0	0	0	0	0	0	0	0	0	0	0	0	0	0	0	0	0
AB0090_C	0	0	0	0	0	0	0	0	0	0	0	0	0	0	0	0	0
AB0091_C	0	0	0	0	0	0	0	0	0	0	0	0	0	0	0	0	0
AB0092_C	0	0	0	0	0	0	0	0	0	0	0	0	0	0	0	0	0
AB0094_C	0	0	0	0	0	0	0	0	0	0	0	0	0	0	0	0	0
AB0095_C	0	0	0	0	0	0	0	0	0	0	0	0	0	0	0	0	0
AB0097_C	0	0	0	0	0	0	0	0	0	0	0	0	0	0	0	0	0
AB0098_C	0	0	0	0	0	0	0	0	0	0	0	0	0	0	0	0	0
AB0099_C	0	0	0	0	0	0	0	0	0	0	0	0	0	0	0	0	0
AB0100_C	0	0	0	0	0	0	0	0	0	0	0	0	0	0	0	0	0
AB0101_C	0	0	0	0	0	0	0	0	0	0	0	0	0	0	0	0	0
AB0103_C	0	0	0	0	0	0	0	0	0	0	0	4	0	0	0	0	0
AB0104_C	0	0	0	0	0	0	0	0	0	0	0	2	0	0	0	1	0
AB0108_C	0	0	0	0	0	0	0	0	0	0	0	2	0	0	0	0	0
AB0109_C	0	0	0	0	0	0	0	0	0	0	0	0	0	0	0	0	0
AB0110_C	0	0	0	0	0	0	0	0	0	0	0	0	0	0	0	0	0
AB0111_C	0	0	0	0	0	0	0	0	0	0	0	0	0	0	0	0	0
AB0112_C	0	0	0	0	0	0	0	0	0	0	0	0	0	0	0	0	0
AB0113_C	0	0	0	0	0	0	0	0	0	0	0	0	0	0	0	0	0
AB0114_C	0	0	0	0	0	0	0	0	0	0	0	0	0	0	0	0	0
AB0115_C	0	0	0	0	0	0	0	0	0	0	0	0	0	0	0	0	0
AB0117_C	0	0	0	0	0	0	0	0	0	0	0	2	0	0	0	0	0
AB0118_C	0	0	0	0	0	0	0	0	0	0	0	0	0	0	0	2	0
AB0119_C	0	0	0	0	0	0	0	0	0	0	0	2	0	0	0	0	0
AB0122_C	0	0	0	0	0	0	0	0	0	0	0	0	0	0	0	0	0
AB0123_C	0	0	0	0	0	0	0	0	0	0	0	0	0	0	0	0	0
AB0124_C	0	0	0	0	0	0	0	0	0	0	0	0	0	0	0	0	0
AB0126_C	0	0	0	0	0	0	0	0	0	0	0	0	0	0	0	0	0
AB0127_C	0	0	0	0	1	0	0	0	0	0	0	0	0	0	0	0	0
AB0128_C	0	0	0	0	0	0	0	0	0	0	0	3	0	0	0	0	0
AB0129_C	0	0	0	0	0	0	0	0	0	0	0	0	0	0	0	0	0
AB0130_C	0	0	0	0	0	0	0	0	0	0	0	0	0	0	0	0	0
AB0133_C	0	0	0	0	1	0	0	0	0	0	0	0	0	0	0	0	0
AB0134_C	0	0	0	0	1	0	0	0	0	0	0	2	0	0	0	0	0
AB0135_C	0	0	0	0	0	0	0	0	0	0	0	3	0	0	0	2	0
AB0136_C	0	0	0	0	0	0	0	0	0	0	0	4	0	0	0	0	0
AB0137_C	0	0	0	0	0	0	0	0	0	0	0	0	0	0	0	0	0
AB0138_C	0	0	0	0	0	0	0	0	0	0	0	0	0	0	0	0	0
AB0139_C	0	0	0	0	0	0	0	0	0	0	0	0	0	0	0	0	0
AB0140_C	0	0	0	0	0	0	0	0	0	0	0	0	0	0	0	0	0
AB0142_C	0	0	0	0	0	0	0	0	0	0	0	0	0	0	0	0	0
AB0143_C	0	0	0	0	0	0	0	2	0	0	0	0	0	0	0	0	0
AB0145_C	0	0	0	0	0	0	0	0	0	0	0	2	0	0	0	0	0
AB0146_C	0	0	0	0	0	0	0	0	0	0	0	3	0	0	0	2	0
AB0147_C	0	0	0	0	1	0	0	0	0	0	0	2	0	0	0	0	0
AB0148_C	0	0	0	0	0	0	0	0	0	0	0	0	0	0	0	3	0
AB0150_C	0	0	0	0	0	0	0	0	0	0	0	0	0	0	0	0	0
AB0151_C	0	0	0	0	0	0	0	0	0	0	0	2	0	0	0	0	0
AB0153_C	0	0	0	0	0	0	0	0	0	0	0	1	0	0	0	0	0
AB0155_C	0	0	0	0	0	0	0	0	0	0	0	0	0	0	0	0	0
AB0157_C	0	0	0	0	1	0	0	0	0	0	0	0	0	0	0	0	0
AB0158_C	0	0	0	0	0	0	0	2	0	0	0	0	0	0	0	0	0
AB0159_C	0	0	0	0	0	0	0	0	0	0	0	2	0	0	0	0	0
AB0160_C	0	0	0	0	0	0	0	0	0	0	0	0	0	0	0	1	0
AB0161_C	0	0	0	0	1	0	0	0	0	0	0	0	0	0	0	0	0
AB0162_C	0	0	0	0	0	0	0	0	0	0	0	2	0	0	0	0	0
AB0164_C	0	0	0	0	0	0	0	0	0	0	0	0	0	0	0	2	0
AB0165_C	0	0	0	0	0	0	0	0	0	0	0	0	0	0	0	2	0
AB0166_C	0	0	0	0	0	0	0	0	0	0	0	0	0	0	0	2	0
AB0167_C	0	0	0	0	0	0	0	0	0	0	0	0	0	0	1	0	0
AB0169_C	0	0	0	0	0	0	0	0	0	0	0	0	0	0	0	0	0
AB0170_C	0	0	0	0	0	0	0	0	0	0	0	0	0	0	0	0	0
AB0171_C	0	0	0	0	0	0	0	0	0	0	0	2	0	0	0	2	0

	Dup 0	Dup 1	Dup 2	Dup 3	Dup 4	Dup 5	Dup 6	Dup 7	Dup 8	Dup 9	Dup 10	Dup 11	Dup 12	Dup 13	Dup 14	Dup 15	Dup 16
AB0172_C	0	0	0	0	0	0	0	0	0	0	0	0	0	1	0	2	0
AB0173_C	0	0	0	0	0	0	0	0	0	0	0	NA	0	0	0	2	0
AB0174_C	0	0	0	0	0	0	0	0	0	0	0	3	0	0	0	0	0
AB0175_C	0	0	0	0	0	0	0	0	0	0	0	0	0	0	0	6	0
AB0176_C	0	0	0	0	0	0	0	0	0	0	0	0	0	0	0	4	0
AB0177_C	0	0	0	0	0	0	0	0	0	0	0	2	0	0	0	1	0
AB0178_C	0	0	0	0	0	0	0	0	0	0	0	2	0	0	0	2	0
AB0179_C	0	0	0	0	0	0	0	0	0	0	0	2	0	0	0	2	0
AB0181_C	0	0	0	0	0	0	0	0	0	0	0	0	0	0	0	0	0
AB0182_C	0	0	0	0	0	0	0	0	0	0	0	0	0	0	0	0	0
AB0183_C	0	0	0	0	0	0	0	0	0	0	0	0	0	0	0	0	0
AB0184_C	0	0	0	0	0	0	0	0	0	0	0	0	0	0	0	0	0
AB0185_C	0	0	0	0	0	0	0	0	0	0	0	0	0	0	0	0	0
AB0186_C	0	0	0	0	0	0	0	0	0	0	0	0	0	0	0	0	0
AB0187_C	0	0	0	0	0	0	0	0	0	0	0	0	0	0	0	0	0
AB0188_C	0	0	0	0	0	0	0	0	0	0	0	0	0	0	0	0	0
AB0189_C	0	0	0	0	0	0	0	0	0	0	0	0	0	0	0	0	0
AB0190_C	0	0	0	0	0	0	0	0	0	0	0	0	0	0	0	0	0
AB0191_C	0	0	0	0	0	0	0	0	0	0	0	0	0	0	0	0	0
AB0192_C	0	0	0	0	0	0	0	0	0	0	0	0	0	0	0	0	0
AB0195_C	0	0	0	0	0	0	0	0	0	0	0	0	0	0	0	0	0
AB0196_C	0	0	0	0	0	0	0	0	0	0	0	0	0	0	0	0	0
AB0197_C	0	0	0	0	0	0	0	NA	0	0	0	0	0	0	0	NA	0
AB0198_C	0	0	0	0	0	0	0	0	0	0	0	4	0	0	0	4	0
AB0199_C	0	0	0	0	0	0	0	0	0	0	0	2	0	0	0	2	0
AB0200_C	0	0	0	0	1	0	0	0	0	0	0	1	0	0	0	0	0
AB0201_C	0	0	0	0	0	0	0	0	0	0	0	0	0	0	0	4	0
AB0202_C	0	0	0	0	0	0	0	0	0	0	0	2	0	0	0	2	0
AB0203_C	0	0	0	0	0	0	0	0	0	0	0	4	0	0	0	4	0
AB0204_C	0	0	0	0	0	0	0	0	0	0	0	0	0	0	0	0	0
AB0205_C	0	0	0	0	0	0	0	0	0	0	0	2	0	0	0	5	0
AB0206_C	0	0	0	0	0	0	0	0	0	0	0	0	0	0	2	3	0
AB0207_C	0	0	0	0	0	0	0	0	0	0	0	2	0	0	0	0	0
AB0208_C	0	0	0	0	0	0	0	0	0	0	0	5	0	0	0	0	0
AB0209_C	0	0	0	0	0	0	0	0	0	0	0	0	0	0	0	0	0
AB0210_C	0	0	0	0	0	0	0	0	0	0	0	0	0	0	0	0	0
AB0211_C	0	0	0	0	0	0	0	0	0	0	0	2	0	0	0	2	0
AB0212_C	0	0	0	0	0	0	0	0	0	0	0	0	0	0	0	0	0
AB0213_C	0	0	0	0	0	0	0	0	0	0	0	0	0	0	0	0	0
AB0215_C	0	0	0	0	0	0	0	0	0	0	0	0	0	0	0	0	0
AB0217_C	0	0	0	0	0	0	0	0	0	0	0	4	0	0	0	0	0
AB0218_C	0	0	0	0	0	0	0	0	0	0	0	3	0	0	0	0	0
AB0219_C	0	0	0	0	0	0	0	0	0	0	0	0	0	0	0	0	0
AB0221_C	0	0	0	0	0	0	0	0	0	0	0	0	0	0	0	0	0
AB0222_C	0	0	0	0	0	0	0	0	0	0	0	0	0	0	0	0	0
AB0223_C	0	0	0	0	0	0	0	0	0	0	0	0	0	0	0	0	0
AB0224_C	0	0	0	0	0	0	0	0	0	0	0	0	0	0	0	0	0
AB0226_C	0	0	0	0	0	0	0	0	0	0	0	0	0	0	0	0	0
AB0227_C	0	0	0	0	0	0	0	0	0	0	0	0	0	0	0	0	0
AB0228_C	0	0	0	0	0	0	0	0	0	0	0	2	0	0	0	2	0
AB0229_C	0	0	0	0	0	0	0	0	0	0	0	0	0	0	0	0	0
AB0231_C	0	0	0	0	0	0	0	0	0	0	0	NA	0	0	0	5	0
AB0232_C	0	0	0	0	1	0	0	0	0	0	0	2	0	0	0	0	0
AB0233_C	0	0	0	0	0	0	0	0	0	0	0	3	0	0	0	0	0
AB0234_C	0	0	0	0	0	0	0	0	0	0	0	0	0	0	0	0	0
AB0235_C	0	0	0	0	1	0	0	0	0	0	0	0	0	0	0	0	0
AB0236_C	0	0	0	0	0	0	0	0	0	0	0	1	0	0	0	0	0
AB0237_C	0	0	0	0	0	0	0	0	0	0	0	0	0	0	0	0	0
AB0238_C	0	0	0	0	0	0	0	NA	0	0	0	0	0	0	0	0	0
AB0239_C	0	0	0	0	0	0	0	0	0	0	0	2	0	0	0	2	0
AB0240_C	0	0	0	0	0	0	0	0	0	0	0	0	0	0	0	0	0
AB0241_C	0	0	0	0	0	0	0	0	0	0	0	4	0	0	0	0	0
AB0242_C	0	0	0	0	0	0	0	0	0	0	0	0	0	0	0	0	0
AB0243_C	0	0	0	0	0	0	0	0	0	0	0	0	0	0	0	0	0
AB0244_C	0	0	0	0	0	0	0	0	0	0	0	2	0	0	0	1	0
AB0246_C	0	0	0	0	0	0	0	0	0	0	0	0	0	0	0	0	0
AB0247_C	0	0	0	0	0	0	0	0	0	0	0	0	0	0	0	0	0
AB0248_C	0	0	0	0	0	0	0	0	0	0	0	0	0	0	0	0	0

	Dup 0	Dup 1	Dup 2	Dup 3	Dup 4	Dup 5	Dup 6	Dup 7	Dup 8	Dup 9	Dup 10	Dup 11	Dup 12	Dup 13	Dup 14	Dup 15	Dup 16
AB0249_C	0	0	0	0	0	0	0	0	0	0	0	0	0	0	0	0	0
AB0250_C	0	0	0	0	0	0	0	0	0	0	0	0	0	0	0	0	0
AB0251_C	0	0	0	0	0	0	0	0	0	0	0	2	0	0	0	0	0
AB0252_C	0	0	0	0	0	0	0	0	0	0	0	2	0	0	0	0	0
AB0253_C	0	0	0	0	0	0	0	0	0	0	0	4	0	0	0	0	0
AB0255_C	0	0	0	0	0	0	0	0	0	0	0	4	0	2	0	0	0
AB0256_C	0	0	0	0	1	0	0	0	0	0	0	0	0	0	0	4	0
AB0257_C	0	0	0	0	0	0	0	0	0	0	0	0	0	0	0	0	0
AB0258_C	0	0	0	0	0	0	0	0	0	0	0	0	0	0	0	0	0
AB0260_C	0	0	0	0	0	0	0	NA	0	0	0	NA	0	0	0	3	0
AB0261_C	0	0	0	0	0	0	0	0	0	0	0	4	0	0	0	2	0
AB0262_C	0	0	0	0	0	0	0	0	0	0	0	0	0	0	0	0	0
AB0263_C	0	0	0	0	0	0	0	0	0	0	0	0	0	0	0	0	0
AB0264_C	0	0	0	0	0	0	0	0	0	0	0	2	0	0	0	3	0
AB0265_C	0	0	0	0	0	0	0	0	0	0	0	1	0	0	0	6	0
AB0266_C	0	0	0	0	0	0	0	0	0	0	0	0	0	0	0	0	0
AB0267_C	0	0	0	0	0	0	0	0	0	0	0	0	0	0	0	0	0
AB0268_C	0	0	0	0	1	0	0	0	0	0	0	2	0	0	0	0	0
AB0270_C	0	0	0	0	1	0	0	0	0	0	0	0	0	0	0	2	0
AB0271_C	0	0	0	0	0	0	0	0	0	0	0	4	0	0	0	0	0
AB0272_C	0	0	0	0	0	0	0	0	0	0	0	4	0	0	0	0	0
AB0273_C	0	0	0	0	0	0	0	NA	0	0	0	NA	0	0	0	5	0
AB0274_C	0	0	0	0	0	0	0	0	0	0	0	1	0	0	0	1	0
AB0275_C	0	0	0	0	NA	0	0	0	0	0	0	0	0	0	0	NA	0
AB0276_C	0	0	0	0	0	0	0	0	0	0	0	0	0	0	0	0	0
AB0277_C	0	0	0	0	0	0	0	0	0	0	0	3	0	0	0	0	0
AB0278_C	0	0	0	0	1	0	0	0	0	0	0	2	0	0	0	0	0
AB0279_C	0	0	0	0	0	0	0	0	0	0	0	0	0	0	0	0	0
AB0280_C	0	0	0	0	1	0	0	0	0	0	0	0	0	0	0	2	0
AB0281_C	0	0	0	0	0	0	0	0	0	0	0	0	0	0	0	6	0
AB0282_C	0	0	0	0	0	0	0	0	0	0	0	0	0	0	0	0	0
AB0283_C	0	0	0	0	0	0	0	0	0	0	0	1	0	0	0	3	0
AB0284_C	0	0	0	0	0	0	0	0	0	0	0	2	0	0	0	2	0
AC0089_C	0	0	0	0	0	0	0	0	0	0	0	0	0	0	0	0	0
AC0090_C	0	0	0	0	0	0	0	0	1	0	0	0	0	0	0	0	0
AC0091_C	0	0	0	0	0	0	0	0	0	0	0	0	0	0	0	0	0
AC0092_C	0	0	0	0	0	0	0	0	0	0	0	0	0	0	0	0	0
AC0093_C	0	0	0	0	0	0	0	0	0	0	0	0	0	0	0	0	0
AC0094_C	0	0	0	0	0	0	0	0	1	0	0	0	0	0	0	0	0
AC0095_C	0	0	0	0	0	0	0	0	0	0	0	0	0	0	0	0	0
AC0096_C	0	0	0	0	0	0	0	0	0	0	0	0	0	0	0	0	0
AC0097_C	0	0	0	0	0	0	0	0	1	0	0	0	0	0	0	0	0
AC0098_C	0	0	0	0	0	0	0	0	0	0	0	0	0	0	0	0	0
AC0099_C	0	0	0	0	0	0	0	0	0	0	0	0	0	0	0	0	0
AC0100_C	0	0	0	0	0	0	0	0	0	0	0	0	0	0	0	0	1
AC0101_C	0	0	0	0	0	0	0	0	1	0	0	0	0	0	0	0	0
AC0102_C	0	0	0	0	0	0	0	0	1	0	0	0	0	0	0	0	0
AC0103_C	0	0	0	0	0	0	0	0	0	0	0	0	0	0	0	0	0
AC0104_C	0	0	0	0	0	0	0	0	0	0	0	0	0	0	0	0	0
AC0105_C	0	0	0	0	0	0	0	0	0	0	0	0	0	0	0	0	0
AC0106_C	0	0	0	0	0	0	0	0	1	0	0	0	0	0	0	0	0
AC0107_C	0	0	0	0	0	0	0	0	2	0	0	0	0	0	0	0	0
AC0108_C	0	0	0	0	0	0	0	0	1	0	0	0	0	0	0	0	0
AC0109_C	0	0	0	0	0	0	0	0	0	0	0	0	0	0	0	0	0
AC0110_C	0	0	0	0	0	0	0	0	0	0	0	0	0	0	0	0	0
AC0111_C	0	0	0	0	0	0	0	0	0	0	0	0	0	0	0	0	0
AC0112_C	0	0	0	0	0	0	0	0	0	0	0	0	0	0	0	0	0
AC0113_C	0	0	0	0	0	0	0	0	0	0	0	0	0	0	0	0	0
AC0114_C	0	0	0	0	0	0	0	0	0	0	0	0	0	0	0	0	0
AC0115_C	0	0	0	0	0	0	0	0	0	0	0	0	0	0	0	0	0
AC0116_C	0	0	0	0	0	0	0	0	0	0	0	0	0	0	0	0	0
AC0117_C	0	0	0	0	0	0	0	0	1	0	0	0	0	0	0	0	0
AC0118_C	0	0	0	0	0	0	0	0	0	0	0	0	0	0	0	0	0
AC0119_C	0	0	0	0	0	0	0	0	0	0	0	0	0	0	0	0	0
AC0120_C	0	0	0	0	0	0	0	0	0	0	0	0	0	0	0	0	0
AC0121_C	0	0	0	0	0	1	0	0	0	0	0	0	0	0	0	0	0
AC0122_C	0	0	0	0	0	0	0	0	1	0	0	0	0	0	0	0	0
AC0123_C	0	0	0	0	0	0	0	0	0	0	0	0	0	0	0	0	0

	Dup 0	Dup 1	Dup 2	Dup 3	Dup 4	Dup 5	Dup 6	Dup 7	Dup 8	Dup 9	Dup 10	Dup 11	Dup 12	Dup 13	Dup 14	Dup 15	Dup 16
AC0124_C	0	0	0	0	0	0	0	0	1	0	0	0	0	0	0	0	0
AC0125_C	0	0	0	0	0	0	0	0	0	0	0	0	0	0	0	0	0
AC0126_C	0	0	0	0	0	0	0	0	0	0	0	0	0	0	0	0	0
AC0127_C	0	0	0	0	0	0	0	0	0	0	0	0	0	0	0	0	1
AC0128_C	0	0	0	0	0	0	0	0	1	0	0	0	0	0	0	0	0
AC0129_C	0	0	0	0	0	0	0	0	0	0	0	0	0	0	0	0	0
AC0130_C	0	0	0	0	0	0	0	0	0	0	0	0	0	0	0	0	0
AC0131_C	0	0	0	0	0	0	0	0	0	0	0	0	0	0	0	0	0
AC0132_C	0	0	0	0	0	0	0	0	2	0	0	0	0	0	0	0	0
AC0133_C	0	0	0	0	0	0	0	0	0	0	0	0	0	0	0	0	0
AC0134_C	0	0	0	0	0	0	0	0	0	0	0	0	0	0	0	0	0
AC0135_C	0	0	0	0	0	0	0	0	0	0	0	0	0	0	0	0	0
AC0136_C	0	0	0	0	0	0	0	0	0	0	0	0	0	0	0	0	0
AC0137_C	0	0	0	0	0	0	0	0	1	0	0	0	0	0	0	0	0
AC0138_C	0	0	0	0	0	0	0	0	1	0	0	0	0	0	0	0	0
AC0139_C	0	0	0	0	0	0	0	0	1	0	0	0	0	0	0	0	0
AC0140_C	0	0	0	0	0	0	0	0	0	0	0	0	0	0	0	0	0
AC0141_C	0	0	0	0	0	0	0	0	1	0	0	0	0	0	0	0	0
AC0142_C	0	0	0	0	0	0	0	0	0	0	0	0	0	0	0	0	0
AC0143_C	0	0	0	0	0	0	0	0	0	0	0	0	0	0	0	0	0
AC0144_C	0	0	0	0	0	0	0	0	0	0	0	0	0	0	0	0	0
AC0145_C	0	0	0	0	0	0	0	0	0	0	0	0	0	0	0	0	0
AC0146_C	0	0	0	0	0	0	0	0	0	0	0	0	0	0	0	0	0
AC0147_C	0	0	0	0	0	0	0	0	0	0	0	0	0	0	0	0	0
AC0148_C	0	0	0	0	0	0	0	0	0	0	0	0	0	0	0	0	0
AC0149_C	0	0	0	0	0	1	0	0	0	0	0	0	0	0	0	0	0
AC0150_C	0	0	0	0	0	0	0	0	0	0	0	0	0	0	0	0	0
AC0151_C	0	0	0	0	0	0	0	0	0	0	0	0	0	0	0	0	0
AC0152_C	0	0	0	0	0	0	0	0	0	0	0	0	0	0	0	0	0
AC0153_C	0	0	0	0	0	0	0	0	1	0	0	0	0	0	0	0	0
AC0154_C	0	0	0	0	0	0	0	0	0	0	0	0	0	0	0	0	0
AC0155_C	0	0	0	0	0	0	0	0	1	0	0	0	0	0	0	0	0
AC0156_C	0	0	0	0	0	0	0	0	0	0	0	0	0	0	0	0	0
AC0157_C	0	0	0	0	0	0	0	0	0	0	0	0	0	0	0	0	0
AC0158_C	0	0	0	0	0	0	0	0	0	0	0	0	0	0	0	0	1
AC0159_C	0	0	0	0	0	0	0	0	1	0	0	0	0	0	0	0	0
AC0160_C	0	0	0	0	0	0	0	0	1	0	0	0	0	0	0	0	0
AC0161_C	0	0	0	0	0	0	0	0	0	0	0	0	0	0	0	0	0
AC0162_C	0	0	0	0	0	0	0	0	0	0	0	0	0	0	0	0	0
AC0163_C	0	0	0	0	0	0	0	0	0	0	0	0	0	0	0	0	0
AC0164_C	0	0	0	0	0	0	0	0	1	0	0	0	0	0	0	0	0
AC0166_C	0	0	0	0	0	0	0	0	1	0	0	0	0	0	0	0	0
AC0167_C	0	0	0	0	0	0	0	0	0	0	0	0	0	0	0	0	0
AC0168_C	0	0	0	0	0	0	0	0	1	0	0	0	0	0	0	0	0
AC0169_C	0	0	0	0	0	0	0	0	0	0	0	0	0	0	0	0	0
AC0170_C	0	0	0	0	0	0	0	0	1	0	0	0	0	0	0	0	0
AC0171_C	0	0	0	0	0	0	0	0	0	0	0	0	0	0	0	0	0
AC0172_C	0	0	0	0	0	0	0	0	0	0	0	0	0	0	0	0	0
AC0173_C	0	0	0	0	0	0	0	0	0	0	0	0	0	0	0	0	0
AC0174_C	0	0	0	0	0	0	0	0	0	0	0	0	0	0	0	0	0
AC0176_C	0	0	0	0	0	0	0	0	0	0	0	0	0	0	0	0	0
AC0177_C	0	0	0	0	0	0	0	0	0	0	0	0	0	0	0	0	0
AC0178_C	0	0	0	0	0	0	0	0	0	0	0	0	0	0	0	0	0
AC0179_C	0	0	0	0	0	0	0	0	0	0	0	0	0	0	0	0	0
AC0180_C	0	0	0	0	0	0	0	0	1	0	0	0	0	0	0	0	0
AC0181_C	0	0	0	0	0	0	0	0	0	0	0	0	0	0	0	0	0
AC0182_C	0	0	0	0	0	0	0	0	0	0	0	0	0	0	0	0	0
AC0183_C	0	0	0	0	0	0	0	0	0	0	0	0	0	0	0	0	0
AC0184_C	0	0	0	0	0	0	0	0	0	0	0	0	0	0	0	0	0
AC0185_C	0	0	0	0	0	0	0	0	0	0	0	0	0	0	0	0	0
AC0186_C	0	0	0	0	0	0	0	0	0	0	0	0	0	0	0	0	0
AC0187_C	0	0	0	0	0	0	0	0	2	0	0	0	0	0	0	0	0
AC0188_C	0	0	0	0	0	0	0	0	0	0	0	0	0	0	0	0	0
AC0189_C	0	0	0	0	0	0	0	0	1	0	0	0	0	0	0	0	0
AC0190_C	0	0	0	0	0	0	0	0	0	0	0	0	0	0	0	0	0
AC0191_C	0	0	0	0	0	0	0	0	0	0	0	0	0	0	0	0	0
AC0192_C	0	0	0	0	0	0	0	0	0	0	0	0	0	0	0	0	0
AC0193_C	0	0	0	0	0	0	0	0	0	0	0	0	0	0	0	0	0

	Dup 0	Dup 1	Dup 2	Dup 3	Dup 4	Dup 5	Dup 6	Dup 7	Dup 8	Dup 9	Dup 10	Dup 11	Dup 12	Dup 13	Dup 14	Dup 15	Dup 16
AC0194_C	0	0	0	0	0	0	0	0	0	0	0	0	0	0	0	0	0
AC0195_C	0	0	0	0	0	0	0	0	0	0	0	0	0	0	0	0	0
AC0196_C	0	0	0	0	0	0	0	0	0	0	0	0	0	0	0	0	0
AC0197_C	0	0	0	0	0	0	0	0	0	0	0	0	0	0	0	0	0
AC0199_C	0	0	0	0	0	NA	0	0	NA	0	0	0	0	0	0	0	0
AC0200_C	0	0	0	0	0	0	0	0	1	0	0	0	0	0	0	0	0
AC0201_C	0	0	0	0	0	0	0	0	0	0	0	0	0	0	0	0	0
AC0202_C	0	0	0	0	0	0	0	0	1	0	0	0	0	0	0	0	0
AC0203_C	0	0	0	0	0	0	0	0	1	0	0	0	0	0	0	0	0
AG0082_C	0	0	0	0	0	0	0	0	0	0	0	0	2	0	0	0	0
AG0085_C	0	0	0	0	0	0	0	0	0	0	0	0	1	0	0	0	0
AG0089_C	0	0	0	0	0	0	0	0	0	0	0	0	2	0	0	0	0
AG0096_C	0	0	0	0	0	0	0	0	0	0	0	0	0	0	0	0	0
AG0097_C	0	0	0	0	0	0	0	0	0	0	0	0	0	0	0	0	0
AG0098_C	0	0	0	0	0	0	0	0	0	0	0	0	0	0	0	0	0
AG0100_C	0	0	0	0	0	0	0	0	0	0	0	0	1	0	0	0	0
AG0102_C	0	0	0	0	0	0	0	0	0	0	0	0	1	0	0	0	0
AG0104_C	0	0	0	0	0	0	0	0	0	0	0	0	0	0	0	0	0
AG0106_C	0	0	0	0	0	0	0	0	0	0	0	0	0	0	0	0	0
AG0108_C	0	0	0	0	0	0	0	0	0	0	0	0	1	0	0	0	0
AG0109_C	0	0	0	0	0	0	0	0	0	0	0	0	0	0	0	0	0
AG0111_C	0	0	0	0	0	0	0	0	0	0	0	0	0	0	0	0	0
AG0118_C	0	0	0	0	0	0	0	0	0	0	0	0	0	0	0	0	0
AG0120_C	0	0	0	0	0	0	0	0	0	0	0	0	1	0	0	0	0
AG0121_C	0	0	0	0	0	0	0	0	0	0	0	0	0	0	0	0	0
AG0123_C	0	0	0	0	0	0	0	0	0	0	0	0	1	0	0	0	0
AG0125_C	0	0	0	0	0	0	0	0	0	0	0	0	1	0	0	0	0
AG0126_C	0	0	0	0	0	0	0	0	0	0	0	0	1	0	0	0	0
AG0127_C	0	0	0	0	0	0	0	0	0	0	0	0	1	0	0	0	0
AG0128_C	0	0	0	0	0	0	0	0	0	0	0	0	0	0	0	0	0
AG0129_C	0	0	0	0	0	0	0	0	0	0	0	0	1	0	0	0	0
AG0133_C	0	0	0	0	0	0	0	0	0	0	0	0	0	0	0	0	0
AG0134_C	0	0	0	0	0	0	0	0	0	0	0	0	1	0	0	0	0
AG0136_C	0	0	0	0	0	0	0	0	0	0	0	0	0	0	0	0	0
AG0137_C	0	0	0	0	0	0	0	0	0	0	0	0	1	0	0	0	0
AG0138_C	0	0	0	0	0	0	0	0	0	0	0	0	1	0	0	0	0
AG0139_C	0	0	0	0	0	0	0	0	0	0	0	0	1	0	0	0	0
AG0141_C	0	0	0	0	0	0	0	0	0	0	0	0	0	0	0	0	0
AG0142_C	0	0	0	0	0	0	0	0	0	0	0	0	0	0	0	0	0
AG0143_C	0	0	0	0	0	0	0	0	0	0	0	0	1	0	0	0	0
AG0144_C	0	0	0	0	0	0	0	0	0	0	0	0	2	0	0	0	0
AG0145_C	0	0	0	0	0	0	0	0	0	0	0	0	0	0	0	0	0
AG0146_C	0	0	0	0	0	0	0	0	0	0	0	0	0	0	0	0	0
AG0147_C	0	0	0	0	0	0	0	0	0	0	0	0	0	0	0	0	0
AG0148_C	0	0	0	0	0	0	0	0	0	0	0	0	1	0	0	0	0
AG0152_C	0	0	0	0	0	0	0	0	0	0	0	0	1	0	0	0	0
AG0153_C	0	0	0	0	0	0	0	0	0	0	0	0	1	0	0	0	0
AG0156_C	0	0	0	0	0	0	0	0	0	0	0	0	1	0	0	0	0
AG0159_C	0	0	0	0	0	0	0	0	0	0	0	0	0	0	0	0	0
AG0162_C	0	0	0	0	0	0	0	0	0	0	0	0	2	0	0	0	0
AG0163_C	0	0	0	0	0	0	0	0	0	0	0	0	0	0	0	0	0
AG0169_C	0	0	0	0	0	0	0	0	0	0	0	0	1	0	0	0	0
AG0170_C	0	0	0	0	0	0	0	0	0	0	0	0	1	0	0	0	0
AG0172_C	0	0	0	0	0	0	0	0	0	0	0	0	0	0	0	0	0
AG0178_C	0	0	0	0	0	0	0	0	0	0	0	0	1	0	0	0	0
AG0179_C	0	0	0	0	0	0	0	0	0	0	0	0	0	0	0	0	0
AG0181_C	0	0	0	0	0	0	0	0	0	0	0	0	0	0	0	0	0
AG0183_C	0	0	0	0	0	0	0	0	0	0	0	0	0	0	0	0	0
AG0195_C	0	0	0	0	0	0	0	0	0	0	0	0	0	0	0	0	0
AG0197_C	0	0	0	0	0	0	0	0	0	0	0	0	1	0	0	0	0
AG0202_C	0	0	0	0	0	0	0	0	0	0	0	0	1	0	0	0	0
AG0203_C	0	0	0	0	0	0	0	0	0	0	0	0	0	0	0	0	0
AG0204_C	0	0	0	0	0	0	0	0	0	0	0	0	1	0	0	0	0
AG0206_C	0	0	0	0	0	0	0	0	0	0	0	0	1	0	0	0	0
AG0208_C	0	0	0	0	0	0	0	0	0	0	0	0	1	0	0	0	0
AG0214_C	0	0	0	0	0	0	0	0	0	0	0	0	2	0	0	0	0
AG0221_C	0	0	0	0	0	0	0	0	0	0	0	0	2	0	0	0	0
AG0223_C	0	0	0	0	0	0	0	0	0	0	0	0	2	0	0	0	0

	Dup 0	Dup 1	Dup 2	Dup 3	Dup 4	Dup 5	Dup 6	Dup 7	Dup 8	Dup 9	Dup 10	Dup 11	Dup 12	Dup 13	Dup 14	Dup 15	Dup 16
AG0227_C	0	0	0	0	0	0	0	0	0	0	0	0	2	0	0	0	0
AG0229_C	0	0	0	0	0	0	0	0	0	0	0	0	0	0	0	0	0
AG0230_C	0	0	0	0	0	0	0	0	0	0	0	0	1	0	0	0	0
AG0231_C	0	0	0	0	0	0	0	0	0	0	0	0	1	0	0	0	0
AG0232_C	0	0	0	0	0	0	0	0	0	0	0	0	2	0	0	0	0
AG0263_C	0	0	0	0	0	0	0	0	0	0	0	0	0	0	0	0	0
AJ0023_C	0	0	0	0	0	0	0	0	0	0	0	0	0	0	0	0	0
AJ0024_C	0	0	0	0	0	0	0	0	0	0	0	0	0	0	0	0	0
AJ0028_C	0	0	0	0	0	0	0	0	0	0	0	0	0	0	0	0	0
AJ0032_C	0	0	0	0	0	0	0	0	0	0	0	0	0	0	0	0	0
AJ0035_C	0	0	0	0	0	0	0	0	0	0	0	0	0	0	0	0	0
AJ0036_C	0	0	0	0	0	0	0	0	0	0	0	0	0	0	0	0	0
AJ0037_C	0	0	0	0	0	0	0	0	0	0	0	0	0	0	0	0	0
AJ0038_C	0	0	0	0	0	0	0	0	0	0	0	0	0	0	0	0	0
AJ0039_C	0	0	0	0	0	0	0	0	0	0	0	0	0	0	0	0	0
AJ0043_C	0	0	0	0	0	0	0	0	0	0	0	0	0	0	0	0	0
AJ0044_C	0	0	0	0	0	0	0	0	0	0	0	0	0	0	0	0	0
AJ0045_C	0	0	0	0	0	0	0	0	0	0	0	0	0	0	0	0	0
AJ0047_C	0	0	0	0	0	0	0	0	0	0	0	0	0	0	0	0	0
AJ0051_C	0	0	0	0	0	0	0	0	0	0	0	0	0	0	0	0	0
AJ0052_C	0	0	0	0	0	0	0	0	0	0	0	0	0	0	0	0	0
AJ0056_C	0	0	0	0	0	0	0	0	0	0	0	0	0	0	0	0	0
AJ0059_C	0	0	0	1	0	0	0	0	0	0	0	0	0	0	0	0	0
AJ0060_C	0	0	0	0	0	0	0	0	0	0	0	0	0	0	0	0	0
AJ0061_C	0	0	0	0	0	0	0	0	0	0	0	0	0	0	0	0	0
AJ0063_C	0	0	0	0	0	0	0	0	0	0	0	0	0	0	0	0	0
AJ0064_C	0	0	0	0	0	0	0	0	0	0	0	0	0	0	0	0	0
AJ0066_C	0	0	0	0	0	0	0	0	0	0	0	0	0	0	0	0	0
AJ0068_C	0	0	0	0	0	0	0	0	0	0	0	0	0	0	0	0	0
AJ0070_C	0	0	0	0	0	0	0	0	0	0	0	0	0	0	0	0	0
AJ0071_C	0	0	0	0	0	0	0	0	0	0	0	0	0	0	0	0	0
AJ0072_C	0	0	0	0	0	0	0	0	0	0	0	0	0	0	0	0	0
AJ0074_C	0	0	0	0	0	0	0	0	0	0	0	0	1	0	0	0	0
AJ0075_C	0	0	0	0	0	0	0	0	0	0	0	0	0	0	0	0	0
AJ0076_C	0	0	0	0	0	0	0	0	0	0	0	0	0	0	0	0	0
AJ0077_C	0	0	0	0	0	0	0	0	0	0	0	0	0	0	0	0	0
AJ0078_C	0	0	0	0	0	0	0	0	0	0	0	0	0	0	0	0	0
AJ0080_C	0	0	0	0	0	0	0	0	0	0	0	0	0	0	0	0	0
AJ0081_C	0	0	0	0	0	0	0	0	0	0	0	0	0	0	0	0	0
AJ0084_C	0	0	0	1	0	0	0	0	0	0	0	0	0	0	0	0	0
AJ0085_C	0	0	0	0	0	0	0	0	0	0	0	0	0	0	0	0	0
AJ0086_C	0	0	0	0	0	0	0	0	0	0	0	0	1	0	0	0	0
AJ0087_C	0	0	0	0	0	0	0	0	0	0	0	0	1	0	0	0	0
AJ0088_C	0	0	0	0	0	0	0	0	0	0	0	0	1	0	0	0	0
AJ0090_C	0	0	0	0	0	0	0	0	0	0	0	0	0	0	0	0	0
AJ0092_C	0	0	0	0	0	0	0	0	0	0	0	0	1	0	0	0	0
AJ0093_C	0	0	0	0	0	0	0	0	0	0	0	0	0	0	0	0	0
AJ0095_C	0	0	0	0	0	0	0	0	0	0	0	0	0	0	0	0	0
AJ0096_C	0	0	0	0	0	0	0	0	0	0	0	0	0	0	0	0	0
AJ0097_C	0	0	0	0	0	0	0	0	0	0	0	0	0	0	0	0	0
AJ0098_C	0	0	0	0	0	0	0	0	0	0	0	0	0	0	0	0	0
AJ0099_C	0	0	0	0	0	0	0	0	0	0	0	0	0	0	0	0	0
AJ0100_C	0	0	0	0	0	0	0	0	0	0	0	0	0	0	0	0	0
AJ0101_C	0	0	0	0	0	0	0	0	0	0	0	0	0	0	0	0	0
AJ0102_C	0	0	0	0	0	0	0	0	0	0	0	0	0	0	0	0	0
AJ0103_C	0	0	0	0	0	0	0	0	0	0	0	0	0	0	0	0	0
AJ0105_C	0	0	0	0	0	0	0	0	0	0	0	0	0	0	0	0	0
AJ0107_C	0	0	0	0	0	0	0	0	0	0	0	0	0	0	0	0	0
AJ0109_C	0	0	0	0	0	0	0	0	0	0	0	0	0	0	0	0	0
AJ0113_C	0	0	0	0	0	0	0	0	0	0	0	0	0	0	0	0	0
AJ0115_C	0	0	0	0	0	0	0	0	0	0	0	0	0	0	0	0	0
AJ0116_C	0	0	0	0	0	0	0	0	0	0	0	0	0	0	0	0	0
AJ0117_C	0	0	0	0	0	0	0	0	0	0	0	0	0	0	0	0	0
AJ0119_C	0	0	0	0	0	0	0	0	0	0	0	0	0	0	0	0	0
AJ0128_C	0	0	0	0	0	0	0	0	0	0	0	0	0	0	0	0	0
AJ0129_C	0	0	0	0	0	0	0	0	0	0	0	0	0	0	0	0	0
AJ0130_C	0	0	0	0	0	0	0	0	0	0	0	0	0	0	0	0	0
AJ0131_C	0	0	0	0	0	0	0	0	0	0	0	0	0	0	0	0	0

	Dup 0	Dup 1	Dup 2	Dup 3	Dup 4	Dup 5	Dup 6	Dup 7	Dup 8	Dup 9	Dup 10	Dup 11	Dup 12	Dup 13	Dup 14	Dup 15	Dup 16
AJ0132_C	0	0	0	0	0	0	0	0	0	0	0	0	0	0	0	0	0
AJ0133_C	0	0	0	0	0	0	0	0	0	0	0	0	0	0	0	0	0
AJ0134_C	0	0	0	0	0	0	0	0	0	0	0	0	0	0	0	0	0
AJ0135_C	0	0	0	0	0	0	0	0	0	0	0	0	0	0	0	0	0
AJ0136_C	0	0	0	0	0	0	0	0	0	0	0	0	0	0	0	0	0
AJ0137_C	0	0	0	0	0	0	0	0	0	0	0	0	0	0	0	0	0
AJ0138_C	0	0	0	0	0	0	0	0	0	0	0	0	0	0	0	0	0
AJ0139_C	0	0	0	0	0	0	0	0	0	0	0	0	1	0	0	0	0
AJ0140_C	0	0	0	0	0	0	0	0	0	0	0	0	1	0	0	0	0
AJ0141_C	0	0	0	0	0	0	0	0	0	0	0	0	0	0	0	0	0
AJ0142_C	0	0	0	0	0	0	0	0	0	0	0	0	0	0	0	0	0
AJ0143_C	0	0	0	0	0	0	0	0	0	0	0	0	0	0	0	0	0
AJ0144_C	0	0	0	0	0	0	0	0	0	0	0	0	0	0	0	0	0
AJ0145_C	0	0	0	0	0	0	0	0	0	0	0	0	0	0	0	0	0
AJ0146_C	0	0	0	0	0	0	0	0	0	0	0	0	0	0	0	0	0
AJ0147_C	0	0	0	0	0	0	0	0	0	0	0	0	0	0	0	0	0
AJ0148_C	0	0	0	0	0	0	0	0	0	0	0	0	0	0	0	0	0
AJ0149_C	0	0	0	0	0	0	0	0	0	0	0	0	0	0	0	0	0
AJ0150_C	0	0	0	0	0	0	0	0	0	0	0	0	0	0	0	0	0
AJ0151_C	0	0	0	0	0	0	0	0	0	0	0	0	0	0	0	0	0
AJ0152_C	0	0	0	0	0	0	0	0	0	0	0	0	0	0	0	0	0
AJ0153_C	0	0	0	0	0	0	0	0	0	0	0	0	0	0	0	0	0
AJ0154_C	0	0	0	0	0	0	0	0	0	0	0	0	1	0	0	0	0
AJ0155_C	0	0	0	0	0	0	0	0	0	0	0	0	0	0	0	0	0
AJ0156_C	0	0	0	0	0	0	0	0	0	0	0	0	0	0	0	0	0
AJ0157_C	0	0	0	0	0	0	0	0	0	0	0	0	0	0	0	0	0
AJ0158_C	0	0	0	0	0	0	0	0	0	0	0	0	0	0	0	0	0
AJ0159_C	0	0	0	0	0	0	0	0	0	0	0	0	0	0	0	0	0
AJ0161_C	0	0	0	0	0	0	0	0	0	0	0	0	0	0	0	0	0
AK0060_C	0	0	0	0	0	0	0	0	0	0	0	0	0	0	0	0	0
AK0062_C	0	0	0	0	0	0	0	0	0	0	0	0	0	0	0	0	0
AK0065_C	0	0	0	0	0	0	0	0	0	0	0	0	0	0	0	0	0
AK0066_C	0	0	0	0	0	0	0	0	0	0	0	0	0	0	0	0	0
AK0067_C	0	0	0	0	0	0	0	0	0	0	0	0	0	0	0	0	0
AK0068_C	0	0	0	0	0	0	0	0	0	0	0	0	0	0	0	0	0
AK0069_C	0	0	0	0	0	0	0	0	0	0	0	0	0	0	0	0	0
AK0070_C	0	0	0	0	0	0	0	0	0	0	0	0	0	0	0	0	0
AK0072_C	0	0	0	0	0	0	0	0	0	0	0	0	0	0	0	0	0
AK0073_C	0	0	0	0	0	0	0	0	0	0	0	0	0	0	0	0	0
AK0074_C	0	0	0	0	0	0	0	0	0	0	0	0	0	0	0	0	0
AK0075_C	0	0	0	0	0	0	0	0	0	0	0	0	0	0	0	0	0
AK0076_C	0	0	0	0	0	0	0	0	0	0	0	0	0	0	0	0	0
AK0077_C	0	0	0	0	0	0	0	0	0	0	0	0	0	0	0	0	0
AK0078_C	0	0	0	0	0	0	0	0	0	0	0	0	0	0	0	0	0
AK0079_C	0	0	0	0	0	0	0	0	0	0	0	0	0	0	0	0	0
AK0080_C	0	0	0	0	0	0	0	0	0	0	0	0	0	0	0	0	0
AK0081_C	0	0	0	0	0	0	0	0	0	0	0	0	0	0	0	0	0
AK0082_C	0	0	0	0	0	0	0	0	0	0	0	0	0	0	0	0	0
AK0085_C	0	0	0	0	0	0	0	0	0	0	0	0	0	0	0	0	0
AK0086_C	0	0	0	0	0	0	0	0	0	0	0	0	0	0	0	0	0
AK0087_C	0	0	0	0	0	0	0	0	0	0	0	0	0	0	0	0	0
AK0088_C	0	0	0	0	0	0	0	0	0	0	0	0	0	0	0	0	0
AK0089_C	0	0	0	0	0	0	0	0	0	0	0	0	0	0	0	0	0
AK0090_C	0	0	0	0	0	0	0	0	0	0	0	0	0	0	0	0	0
AK0091_C	0	0	0	0	0	0	0	0	0	0	0	0	0	0	0	0	0
AK0092_C	0	0	0	0	0	0	0	0	0	0	0	0	0	0	0	0	0
AK0093_C	0	0	0	0	0	0	0	0	0	0	0	0	0	0	0	0	0
AK0094_C	0	0	0	0	0	0	0	0	0	0	0	0	0	0	0	0	0
AK0095_C	0	0	0	0	0	0	0	0	0	0	0	0	0	0	0	0	0
AK0096_C	0	0	0	0	0	0	0	0	0	0	0	0	0	0	0	0	0
AK0098_C	0	0	0	0	0	0	0	0	0	0	0	0	0	0	0	0	0
AK0099_C	0	0	0	0	0	0	0	0	0	0	0	0	0	0	0	0	0
AK0100_C	0	0	0	0	0	0	0	0	0	0	0	0	0	0	0	0	0
AK0101_C	0	0	0	0	0	0	0	0	0	0	0	0	0	0	0	0	0
AK0102_C	0	0	0	0	0	0	0	0	0	0	0	0	0	0	0	0	0
AK0103_C	0	0	0	0	0	0	0	0	0	0	0	0	0	0	0	0	0
AK0104_C	0	0	0	0	0	0	0	0	0	0	0	0	0	0	0	0	0
AK0105_C	0	0	0	0	0	0	0	0	0	0	0	0	0	0	0	0	0

	Dup 0	Dup 1	Dup 2	Dup 3	Dup 4	Dup 5	Dup 6	Dup 7	Dup 8	Dup 9	Dup 10	Dup 11	Dup 12	Dup 13	Dup 14	Dup 15	Dup 16
AK0106_C	0	0	0	0	0	0	0	0	0	0	0	0	0	0	0	0	0
AK0107_C	0	0	0	0	0	0	0	0	0	0	0	0	0	0	0	0	0
AK0108_C	0	0	0	0	0	0	0	0	0	0	0	0	0	0	0	0	0
AK0109_C	0	0	0	0	0	0	0	0	0	0	0	0	0	0	0	0	0
AK0110_C	0	0	0	0	0	0	0	0	0	0	0	0	0	0	0	0	0
AK0116_C	0	0	0	0	0	0	0	0	0	0	0	0	0	0	0	0	0
AK0117_C	0	0	0	0	0	0	0	0	0	0	0	0	0	0	0	0	0
AK0119_C	0	0	0	0	0	0	0	0	0	0	0	0	0	0	0	0	0
AK0127_C	0	0	0	0	0	0	0	0	0	0	0	0	0	0	0	0	0
AN0007_C	0	0	0	0	0	0	0	0	0	0	0	0	0	0	0	0	0
AN0008_C	0	0	0	0	0	0	0	0	0	0	0	0	0	0	0	0	0
AN0009_C	0	0	0	0	0	0	0	0	0	0	0	0	0	0	0	0	0
AN0010_C	0	1	0	0	0	0	0	0	0	0	0	0	0	0	0	0	0
AN0011_C	0	0	0	0	0	0	0	0	0	0	0	0	0	0	0	0	0
AN0012_C	0	0	0	0	0	0	0	0	0	0	0	0	0	0	0	0	0
AN0013_C	0	0	0	0	0	0	0	0	0	0	0	0	0	0	0	0	0
AN0014_C	0	0	0	0	0	0	0	0	0	0	0	0	0	0	0	0	0
AN0015_C	0	0	0	0	0	0	0	0	0	0	0	0	0	0	0	0	0
AN0016_C	0	0	0	0	0	0	0	0	0	0	0	0	0	0	0	0	0
AN0017_C	0	0	0	0	0	0	0	0	0	0	0	0	0	0	0	0	0
AN0018_C	0	0	0	0	0	0	0	0	0	0	0	0	0	0	0	0	0
AN0019_C	0	0	0	0	0	0	0	0	0	0	0	0	0	0	0	0	0
AN0020_C	0	0	0	0	0	0	0	0	0	0	0	0	0	0	0	0	0
AN0022_C	0	0	0	0	0	0	0	0	0	0	0	0	0	0	0	0	0
AN0023_C	0	0	0	0	0	0	0	0	0	0	0	0	0	0	0	0	0
AN0024_C	0	0	0	0	0	0	0	0	0	0	0	0	0	0	0	0	0
AN0025_C	0	0	0	0	0	0	0	0	0	0	0	0	0	0	0	0	0
AN0026_C	0	0	0	0	0	0	0	0	0	0	0	0	0	0	0	0	0
AN0027_C	0	0	0	0	0	0	0	0	0	0	0	1	0	0	0	0	0
AN0028_C	0	0	0	0	0	0	0	0	0	0	0	0	0	0	0	0	0
AN0029_C	0	0	0	0	0	0	0	0	0	0	0	0	0	0	0	0	0
AN0030_C	0	0	0	0	0	0	0	0	0	0	0	0	0	0	0	0	0
AN0031_C	0	0	0	0	0	0	0	0	0	0	0	0	0	0	0	0	0
AN0032_C	0	0	0	0	0	0	0	0	0	0	0	0	0	0	0	0	0
AN0033_C	0	0	0	0	0	0	0	0	0	0	0	2	0	0	0	0	0
AN0034_C	0	0	0	0	0	0	0	0	0	0	0	0	0	0	0	0	0
AN0035_C	0	0	0	0	0	0	0	0	0	0	0	0	0	0	0	0	0
AN0036_C	0	0	0	0	0	0	0	0	0	0	0	0	0	0	0	0	0
AN0037_C	0	0	0	0	0	0	0	0	0	0	0	0	0	0	0	0	0
AN0038_C	0	0	0	0	0	0	0	0	0	0	0	0	0	0	0	0	0
AN0039_C	0	0	0	0	0	0	0	0	0	0	0	0	0	0	0	0	0
AN0040_C	0	0	0	0	0	0	0	0	0	0	0	0	0	0	0	0	0
AN0041_C	0	0	0	0	0	0	0	0	0	0	0	0	0	0	0	0	0
AN0042_C	0	0	0	0	0	0	0	0	0	0	0	0	0	0	0	0	0
AN0043_C	0	0	0	0	0	0	0	0	0	0	0	0	0	0	0	0	0
AN0044_C	0	0	0	0	0	0	0	0	0	0	0	0	0	0	0	0	0
AN0045_C	0	0	0	0	0	0	0	0	0	0	0	0	0	0	0	0	0
AN0046_C	0	0	0	0	0	0	0	0	0	0	0	0	0	0	0	0	0
AN0047_C	0	0	0	0	0	0	0	0	0	0	0	0	0	0	0	0	0
AN0048_C	0	0	0	0	0	0	0	0	0	0	0	0	0	0	0	0	0
AN0049_C	0	0	0	0	0	0	0	0	0	0	0	0	0	0	0	0	0
AN0050_C	0	0	0	0	0	0	0	0	0	1	0	0	0	0	0	0	0
AN0051_C	0	0	0	0	0	0	0	0	0	0	0	0	0	0	0	0	0
AN0053_C	0	0	0	0	0	0	0	0	0	0	0	0	0	0	0	0	0
AN0054_C	0	0	0	0	0	0	0	0	0	0	0	0	0	0	0	0	0
AN0055_C	0	0	0	0	0	0	0	0	0	0	0	0	0	0	0	0	0
AN0056_C	0	0	0	0	0	0	0	0	0	0	0	0	0	0	0	0	0
AN0057_C	0	0	0	0	0	0	0	0	0	0	0	0	0	0	0	0	0
AN0058_C	0	0	0	0	1	0	0	0	0	0	0	0	0	0	0	0	0
AN0059_C	0	0	0	0	0	0	0	0	0	1	0	0	0	0	0	0	0
AN0060_C	0	0	0	0	0	0	0	0	0	0	0	0	0	0	0	0	0
AN0061_C	0	0	0	0	0	0	0	0	0	0	0	0	0	0	0	0	0
AN0062_C	0	0	0	0	0	0	0	0	0	0	0	0	0	0	0	0	0
AN0063_C	0	0	0	0	0	0	0	0	0	0	0	0	0	0	0	0	0
AN0064_C	0	0	0	0	0	0	0	0	0	0	0	0	0	0	0	0	0
AN0065_C	0	0	0	0	0	0	0	0	0	0	0	0	0	0	0	0	0
AN0066_C	0	0	0	0	0	0	0	0	0	0	0	0	0	0	0	0	0
AN0067_C	0	0	0	0	0	0	0	0	0	0	0	0	0	0	0	0	0

	Dup 0	Dup 1	Dup 2	Dup 3	Dup 4	Dup 5	Dup 6	Dup 7	Dup 8	Dup 9	Dup 10	Dup 11	Dup 12	Dup 13	Dup 14	Dup 15	Dup 16
AN0068_C	0	0	0	0	0	0	0	0	0	0	0	0	0	0	0	0	0
AN0069_C	0	0	0	0	0	0	0	0	0	0	0	0	0	0	0	0	0
AN0070_C	0	0	0	0	0	0	0	0	0	0	0	0	0	0	0	0	0
AN0071_C	0	0	0	0	0	0	0	0	0	0	0	0	0	0	0	0	0
AN0072_C	0	0	0	0	0	0	0	0	0	0	0	0	0	0	0	0	0
AN0073_C	0	0	0	0	0	0	0	0	0	0	0	0	0	0	0	0	0
AN0074_C	0	0	0	0	0	0	0	0	0	0	0	0	0	0	0	0	0
AN0075_C	0	0	0	0	0	0	0	0	0	0	0	0	0	0	0	0	0
AN0076_C	0	0	0	0	0	0	0	0	0	0	0	0	0	0	0	0	0
AN0077_C	0	0	0	0	0	0	0	0	0	0	0	0	0	0	0	0	0
AN0079_C	0	0	0	0	0	0	0	0	0	0	0	0	0	0	0	0	0
AN0080_C	0	0	0	0	1	0	0	0	0	0	0	0	0	0	0	0	0
AN0081_C	0	0	0	0	0	0	0	0	0	0	0	0	0	0	0	0	0
AN0082_C	0	0	0	0	0	0	0	0	0	0	0	0	0	0	0	0	0
AN0083_C	0	0	0	0	0	0	0	0	0	0	0	0	0	0	0	0	0
AN0084_C	0	0	0	0	0	0	0	0	0	0	0	0	0	0	0	0	0
AN0085_C	0	0	0	0	0	0	0	0	0	0	0	0	0	0	0	0	0
AN0086_C	0	0	0	0	0	0	0	0	0	0	0	0	0	0	0	0	0
AN0087_C	0	0	0	0	0	0	0	0	0	0	0	0	0	0	0	0	0
AN0088_C	0	0	0	0	0	0	0	0	0	0	0	0	0	0	0	0	0
AN0089_C	0	0	0	0	0	0	0	0	0	0	0	0	0	0	0	0	0
AN0090_C	0	0	0	0	0	0	0	0	0	0	0	0	0	0	0	0	0
AN0091_C	0	0	0	0	0	0	0	0	0	0	0	0	0	0	0	0	0
AN0092_C	0	0	0	0	0	0	0	0	0	0	0	0	0	0	0	0	0
AN0093_C	0	0	0	0	0	0	0	0	0	0	0	0	0	0	0	0	0
AN0094_C	0	0	0	0	0	0	0	0	0	0	0	0	0	0	0	0	0
AN0095_C	0	0	0	0	0	0	0	0	0	0	0	0	0	0	0	0	0
AN0096_C	0	0	0	0	0	0	0	0	0	0	0	0	0	0	0	0	0
AN0097_C	0	0	0	0	0	0	0	0	0	0	0	0	0	0	0	0	0
AN0098_C	0	0	0	0	0	0	0	0	0	0	0	0	0	0	0	0	0
AN0099_C	0	0	0	0	0	0	0	0	0	0	0	0	0	0	0	0	0
AN0100_C	0	0	0	0	0	0	0	0	0	0	0	0	0	0	0	0	0
AN0101_C	0	0	0	0	0	0	0	0	0	0	0	0	0	0	0	0	0
AN0102_C	0	0	0	0	0	0	0	0	0	0	0	0	0	0	0	0	0
AN0103_C	0	0	0	0	0	0	0	0	0	0	0	0	0	0	0	0	0
AN0104_C	0	0	0	0	0	0	0	0	0	0	0	0	0	0	0	0	0
AN0105_C	0	0	0	0	0	0	0	0	0	1	0	0	0	0	0	0	0
AN0106_C	0	0	0	0	0	0	0	0	0	0	0	0	0	0	0	0	0
AN0107_C	0	0	0	0	0	0	0	0	0	0	0	0	0	0	0	0	0
AN0108_C	0	0	0	0	0	0	0	0	0	0	0	0	0	0	0	0	0
AN0109_C	0	0	0	0	0	0	0	0	0	0	0	0	0	0	0	0	0
AN0111_C	0	0	0	0	0	0	0	0	0	0	0	0	0	0	0	0	0
AN0112_C	0	0	0	0	0	0	0	0	0	0	0	0	0	0	0	0	0
AN0113_C	0	0	0	0	0	0	0	0	0	0	0	0	0	0	0	0	0
AN0114_C	0	0	0	0	0	0	0	0	0	0	0	0	0	0	0	0	0
AN0115_C	0	0	0	0	0	0	0	0	0	0	0	0	0	0	0	0	0
AN0117_C	0	0	0	0	0	0	0	0	0	0	0	0	0	0	0	0	0
AN0118_C	0	0	0	0	0	0	0	0	0	0	0	0	0	0	0	0	0
AN0119_C	0	0	0	0	0	0	0	0	0	0	0	0	0	0	0	0	0
AN0120_C	0	0	0	0	0	0	0	0	0	0	0	0	0	0	0	0	0
AN0121_C	0	0	0	0	0	0	0	0	0	0	0	0	0	0	0	0	0
AN0122_C	0	0	0	0	0	0	0	0	0	0	0	0	0	0	0	0	0
AN0123_C	0	0	0	0	0	0	0	0	0	0	0	0	0	0	0	0	0
AN0124_C	0	0	0	0	0	0	0	0	0	0	0	0	0	0	0	0	0
AN0125_C	0	0	0	0	0	0	0	0	0	0	0	0	0	0	0	0	0
AN0126_C	0	0	0	0	0	0	0	0	0	0	0	0	0	0	0	0	0
AN0127_C	0	0	0	0	0	0	0	0	0	0	0	0	0	0	0	0	0
AN0128_C	0	0	0	0	0	0	0	0	0	0	0	0	0	0	0	0	0
AN0129_C	0	0	0	0	0	0	0	0	0	0	0	0	0	0	0	0	0
AN0130_C	0	0	0	0	0	0	0	0	0	0	0	0	0	0	0	0	0
AN0131_C	0	0	0	0	0	0	0	0	0	0	0	0	0	0	0	0	0
AN0132_C	0	0	0	0	0	0	0	0	0	0	0	0	0	0	0	0	0
AN0134_C	0	0	0	0	0	0	0	0	0	0	0	0	0	0	0	0	0
AN0135_C	0	0	0	0	0	0	0	0	0	0	0	0	0	0	0	0	0
AN0136_C	0	0	0	0	0	0	0	0	0	0	0	0	0	0	0	0	0
AN0137_C	0	0	0	0	0	0	0	0	0	0	0	0	0	0	0	0	0
AN0138_C	0	0	0	0	0	0	0	0	0	0	0	0	0	0	0	0	0
AN0139_C	0	0	0	0	0	0	0	0	0	0	0	0	0	0	0	0	0

	Dup 0	Dup 1	Dup 2	Dup 3	Dup 4	Dup 5	Dup 6	Dup 7	Dup 8	Dup 9	Dup 10	Dup 11	Dup 12	Dup 13	Dup 14	Dup 15	Dup 16
AN0140_C	0	0	0	0	0	0	0	0	0	0	0	0	0	0	0	0	0
AN0141_C	0	0	0	0	0	0	0	0	0	0	0	0	0	0	0	0	0
AN0142_C	0	0	0	0	0	0	0	0	0	0	0	0	0	0	0	0	0
AN0143_C	0	0	0	0	0	0	0	0	0	0	0	0	0	0	0	0	0
AN0144_C	0	0	0	0	0	0	0	0	0	0	0	0	0	0	0	0	0
AN0147_C	0	0	0	0	0	0	0	0	0	0	0	0	0	0	0	0	0
AN0149_C	0	0	0	0	0	0	0	0	0	0	0	0	0	0	0	0	0
AN0151_C	0	0	0	0	0	0	0	0	0	0	0	0	0	0	0	0	0
AN0152_C	0	0	0	0	0	0	0	0	0	0	0	0	0	0	0	0	0
AN0153_C	0	0	0	0	0	0	0	0	0	0	0	0	0	0	0	0	0
AN0154_C	0	0	0	0	0	0	0	0	0	0	0	0	0	0	0	0	0
AN0155_C	0	0	0	0	0	0	0	0	0	0	0	0	0	0	0	0	0
AN0156_C	0	0	0	0	0	0	0	0	0	0	0	0	0	0	0	0	0
AN0157_C	0	0	0	0	0	0	0	0	0	0	0	0	0	0	0	0	0
AN0158_C	0	0	0	0	0	0	0	0	0	0	0	0	0	0	0	0	0
AN0159_C	0	0	0	0	0	0	0	0	0	0	0	0	0	0	0	0	0
AN0160_C	0	0	0	0	0	0	0	0	0	0	0	0	0	0	0	0	0
AN0162_C	0	0	0	0	0	0	0	0	0	0	0	0	0	0	0	0	0
AN0163_C	0	0	0	0	0	0	0	0	0	0	0	0	0	0	0	0	0
AN0164_C	0	0	0	0	0	0	0	0	0	0	0	0	0	0	0	0	0
AN0165_C	0	0	0	0	0	0	0	0	0	0	0	0	0	0	0	0	0
AN0166_C	0	0	0	0	0	0	0	0	0	0	0	0	0	0	0	0	0
AN0167_C	0	0	0	0	0	0	0	0	0	0	0	0	0	0	0	0	0
AN0168_C	0	0	0	0	0	0	0	0	0	0	0	0	0	0	0	0	0
AN0169_C	0	0	0	0	0	0	0	0	0	0	0	0	0	0	0	0	0
AN0170_C	0	0	0	0	0	0	0	0	0	0	0	0	0	0	0	0	0
AN0171_C	0	0	0	0	0	0	0	0	0	0	0	0	0	0	0	0	0
AN0172_C	0	0	0	0	0	0	0	2	0	0	0	0	0	0	0	0	0
AN0173_C	0	0	0	0	0	0	0	0	0	0	0	0	0	0	0	0	0
AN0174_C	0	0	0	0	0	0	0	0	0	0	0	0	0	0	0	0	0
AN0175_C	0	0	0	0	0	0	0	0	0	0	0	0	0	0	0	0	0
AN0176_C	0	0	0	0	0	0	0	0	0	0	0	0	0	0	0	0	0
AN0177_C	0	0	0	0	0	0	0	0	0	0	0	0	0	0	0	0	0
AN0178_C	0	0	0	0	0	0	0	0	0	0	0	0	0	0	0	0	0
AN0179_C	0	0	0	0	0	0	0	0	0	0	0	0	0	0	0	0	0
AN0180_C	0	0	0	0	0	0	0	0	0	0	0	0	0	0	0	0	0
AN0181_C	0	0	0	0	0	0	0	0	0	0	0	0	0	0	0	0	0
AN0182_C	0	0	0	0	0	0	0	0	0	0	0	0	0	0	0	0	0
AN0183_C	0	0	0	0	0	0	0	0	0	0	0	0	0	0	0	0	0
AN0184_C	0	0	0	0	0	0	0	0	0	0	0	0	0	0	0	0	0
AN0185_C	0	0	0	0	0	0	0	0	0	0	0	0	0	0	0	0	0
AN0186_C	0	0	0	0	0	0	0	0	0	0	0	0	0	0	0	0	0
AN0187_C	0	0	0	0	0	0	0	0	0	0	0	0	0	0	0	0	0
AN0188_C	0	0	0	0	0	0	0	0	0	0	0	0	0	0	0	0	0
AN0189_C	0	0	0	0	0	0	0	0	0	0	0	0	0	0	0	0	0
AN0190_C	0	0	0	0	0	0	0	0	0	0	0	0	0	0	0	0	0
AN0191_C	0	0	0	0	0	0	0	0	0	0	0	0	0	0	0	0	0
AN0192_C	0	0	0	0	0	0	0	0	0	0	0	0	0	0	0	0	0
AN0193_C	0	0	0	0	0	0	0	0	0	0	0	0	0	0	0	0	0
AN0194_C	0	0	0	0	0	0	0	0	0	0	0	0	0	0	0	0	0
AN0196_C	0	0	0	0	0	0	0	0	0	0	0	0	0	0	0	0	0
AN0197_C	0	0	0	0	0	0	0	0	0	0	0	0	0	0	0	0	0
AN0198_C	0	0	0	0	0	0	0	0	0	0	0	1	0	0	0	0	0
AN0199_C	0	0	0	0	0	0	0	0	0	0	0	0	0	0	0	0	0
AN0200_C	0	0	0	0	0	0	0	0	0	0	0	0	0	0	0	0	0
AN0201_C	0	0	0	0	0	0	0	0	0	0	0	0	0	0	0	0	0
AN0202_C	0	0	0	0	0	0	0	0	0	0	0	0	0	0	0	0	0
AN0203_C	0	0	0	0	0	0	0	0	0	0	0	0	0	0	0	0	0
AN0204_C	0	0	0	0	0	0	0	0	0	0	0	0	0	0	0	0	0
AN0205_C	0	0	0	0	0	0	0	0	0	0	0	0	0	0	0	0	0
AN0206_C	0	0	0	0	0	0	0	0	0	0	0	0	0	0	0	0	0
AN0207_C	0	0	0	0	0	0	0	0	0	0	0	0	0	0	0	0	0
AN0208_C	0	0	0	0	0	0	0	0	0	0	0	0	0	0	0	0	0
AN0209_C	0	0	0	0	0	0	0	0	0	0	0	0	0	0	0	0	0
AN0210_C	0	0	0	0	0	0	0	0	0	0	0	0	0	0	0	0	0
AN0212_C	0	0	0	0	0	0	0	0	0	0	0	0	0	0	0	0	0
AN0213_C	0	0	0	0	0	0	0	0	0	0	0	0	0	0	0	0	0
AN0214_C	0	0	0	0	0	0	0	0	0	0	0	0	0	0	0	0	0

	Dup 0	Dup 1	Dup 2	Dup 3	Dup 4	Dup 5	Dup 6	Dup 7	Dup 8	Dup 9	Dup 10	Dup 11	Dup 12	Dup 13	Dup 14	Dup 15	Dup 16
AN0215_C	0	0	0	0	0	0	0	0	0	0	0	0	0	0	0	0	0
AN0217_C	0	0	0	0	0	0	0	0	0	0	0	0	0	0	0	0	0
AN0218_C	0	0	0	0	0	0	0	0	0	0	0	2	0	0	0	0	0
AN0219_C	0	0	0	0	0	0	0	0	0	0	0	0	0	0	0	0	0
AN0220_C	0	0	0	0	0	0	0	0	0	0	0	0	0	0	0	0	0
AN0221_C	0	0	0	0	0	0	0	0	0	0	0	0	0	0	0	0	0
AN0222_C	0	0	0	0	0	0	0	0	0	0	0	0	0	0	0	0	0
AN0223_C	0	0	0	0	0	0	0	0	0	0	0	0	0	0	0	0	0
AN0224_C	0	0	0	0	0	0	0	0	0	0	0	0	0	0	0	0	0
AN0225_C	0	0	0	0	0	0	0	0	0	0	0	0	0	0	0	0	0
AN0226_C	0	0	0	0	0	0	0	0	0	0	0	0	0	0	0	0	0
AN0227_C	0	0	0	0	0	0	0	0	0	0	0	0	0	0	0	0	0
AN0228_C	0	0	0	0	0	0	0	0	0	0	0	0	0	0	0	0	0
AN0229_C	0	0	0	0	0	0	0	0	0	0	0	0	0	0	0	0	0
AN0230_C	0	0	0	0	0	0	0	0	0	0	0	0	0	0	0	0	0
AN0231_C	0	0	0	0	0	0	0	0	0	0	0	0	0	0	0	0	0
AN0232_C	0	0	0	0	0	0	0	0	0	0	0	0	0	0	0	0	0
AN0233_C	0	0	0	0	0	0	0	0	0	0	0	0	0	0	0	0	0
AN0234_C	0	0	0	0	0	0	0	0	0	0	0	0	0	0	0	0	0
AN0235_C	0	0	0	0	0	0	0	0	0	0	0	0	0	0	0	0	0
AN0236_C	0	0	0	0	0	0	0	0	0	1	0	0	0	0	0	0	0
AN0237_C	0	0	0	0	0	0	0	0	0	0	0	0	0	0	0	0	0
AN0238_C	0	0	0	0	0	0	0	0	0	0	0	0	0	0	0	0	0
AN0239_C	0	0	0	0	0	0	0	0	0	0	0	0	0	0	0	0	0
AN0240_C	0	0	0	0	0	0	0	0	0	0	0	0	0	0	0	0	0
AN0241_C	0	0	0	0	0	0	0	0	0	0	0	0	0	0	0	0	0
AN0242_C	0	0	0	0	0	0	0	0	0	0	0	0	0	0	0	0	0
AN0243_C	0	0	0	0	0	0	0	0	0	0	0	0	0	0	0	0	0
AN0244_C	0	0	0	0	0	0	0	0	0	0	0	0	0	0	0	0	0
AN0245_C	0	0	0	0	0	0	0	0	0	0	0	0	0	0	0	0	0
AN0246_C	0	0	0	0	0	0	0	0	0	0	0	0	0	0	0	0	0
AN0247_C	0	0	0	0	0	0	0	0	0	0	0	0	0	0	0	0	0
AN0248_C	0	0	0	0	0	0	0	0	0	0	0	0	0	0	0	0	0
AN0250_C	0	0	1	0	0	0	0	0	0	0	0	0	0	0	0	0	0
AN0251_C	0	0	0	0	0	0	0	0	0	0	0	0	0	0	0	0	0
AN0252_C	0	0	0	0	0	0	0	0	0	0	0	0	0	0	0	0	0
AN0253_C	0	0	0	0	0	0	0	0	0	0	0	0	0	0	0	0	0
AN0254_C	0	0	0	0	0	0	0	0	0	0	0	0	0	0	0	0	0
AN0255_C	0	0	0	0	0	0	0	0	0	0	0	0	0	0	0	0	0
AN0256_C	0	0	0	0	0	0	0	0	0	0	0	0	0	0	0	0	0
AN0258_C	0	0	0	0	0	0	0	0	0	0	0	0	0	0	0	0	0
AN0259_C	0	0	0	0	0	0	0	0	0	0	0	0	0	0	0	0	0
AN0260_C	0	0	0	0	0	0	0	0	0	0	0	0	0	0	0	0	0
AN0261_C	0	0	0	0	0	0	0	0	0	0	0	0	0	0	0	0	0
AN0262_C	0	0	0	0	0	0	0	0	0	0	0	0	0	0	0	0	0
AN0263_C	0	0	0	0	0	0	0	0	0	0	0	0	0	0	0	0	0
AN0264_C	0	0	0	0	0	0	0	0	0	0	0	0	0	0	0	0	0
AN0265_C	0	0	0	0	1	0	0	0	0	0	0	0	0	0	0	0	0
AN0266_C	0	0	0	0	1	0	0	0	0	0	0	0	0	0	0	0	0
AN0267_C	0	0	0	0	0	0	0	0	0	0	0	0	0	0	0	0	0
AN0268_C	0	0	0	0	0	0	0	0	0	0	0	0	0	0	0	0	0
AN0269_C	0	0	0	0	0	0	0	0	0	0	0	0	0	0	0	0	0
AN0270_C	0	0	0	0	0	0	0	0	0	0	0	0	0	0	0	0	0
AN0271_C	0	0	0	0	0	0	0	0	0	0	0	0	0	0	0	0	0
AN0272_C	0	0	0	0	0	0	0	0	0	0	0	0	0	0	0	0	0
AN0275_C	0	0	0	0	0	0	0	0	0	0	0	0	0	0	0	0	0
AN0276_C	0	0	0	0	0	0	0	0	0	0	0	0	0	0	0	0	0
AN0277_C	0	0	0	0	0	0	0	0	0	0	0	0	0	0	0	0	0
AN0278_C	0	0	0	0	0	0	0	0	0	0	0	0	0	0	0	0	0
AN0279_C	0	0	0	0	0	0	0	0	0	0	0	0	0	0	0	0	0
AN0280_C	0	0	1	0	0	0	0	0	0	0	0	0	0	0	0	0	0
AN0281_C	0	0	0	0	0	0	0	0	0	0	0	0	0	0	0	0	0
AN0282_C	0	0	0	0	0	0	0	0	0	0	0	0	0	0	0	0	0
AN0283_C	0	1	0	0	0	0	0	0	0	0	0	0	0	0	0	0	0
AN0284_C	0	0	0	0	0	0	0	0	0	0	0	0	0	0	0	0	0
AN0285_C	0	0	0	0	0	0	0	0	0	0	0	0	0	0	0	0	0
AN0286_C	0	0	0	0	0	0	0	0	0	0	0	0	0	0	0	0	0
AN0287_C	0	0	0	0	0	0	0	0	0	0	0	0	0	0	0	0	0

	Dup 0	Dup 1	Dup 2	Dup 3	Dup 4	Dup 5	Dup 6	Dup 7	Dup 8	Dup 9	Dup 10	Dup 11	Dup 12	Dup 13	Dup 14	Dup 15	Dup 16
AN0288_C	0	0	0	0	0	0	0	0	0	0	0	0	0	0	0	0	0
AN0289_C	0	0	0	0	0	0	0	0	0	0	0	0	0	0	0	0	0
AN0290_C	0	0	0	0	0	0	0	0	0	0	0	0	0	0	0	0	0
AN0291_C	0	0	0	0	0	0	0	0	0	0	0	0	0	0	0	0	0
AN0292_C	0	0	0	0	0	0	0	0	0	0	0	0	0	0	0	0	0
AN0293_C	0	0	0	0	0	0	0	0	0	0	0	0	0	0	0	0	0
AN0294_C	0	0	0	0	0	0	0	0	0	0	0	0	0	0	0	0	0
AN0295_C	0	0	0	0	0	0	0	0	0	0	0	0	0	0	0	0	0
AN0296_C	0	0	0	0	0	0	0	0	0	0	0	0	0	0	0	0	0
AN0297_C	0	0	0	0	0	0	0	0	0	0	0	0	0	0	0	0	0
AN0298_C	0	0	0	0	0	0	0	0	0	0	0	0	0	0	0	0	0
AN0299_C	0	0	0	0	0	0	0	0	0	1	0	0	0	0	0	0	0
AN0300_C	0	0	0	0	0	0	0	0	0	0	0	0	0	0	0	0	0
AN0301_C	0	0	0	0	0	0	0	0	0	0	0	0	0	0	0	0	0
AN0302_C	0	0	0	0	0	0	0	0	0	0	0	0	0	0	0	0	0
AN0303_C	0	0	0	0	0	0	0	0	0	0	0	0	0	0	0	0	0
AN0304_C	0	0	0	0	0	0	0	0	0	0	0	0	0	0	0	0	0
AN0305_C	0	0	0	0	0	0	0	0	0	0	0	0	0	0	0	0	0
AN0306_C	0	0	0	0	0	0	0	0	0	0	0	0	0	0	0	0	0
AN0307_C	0	0	0	0	0	0	0	0	0	0	0	0	0	0	0	0	0
AN0308_C	0	0	0	0	0	0	0	0	0	0	0	0	0	0	0	0	0
AN0309_C	0	0	0	0	0	0	0	0	0	0	0	0	0	0	0	0	0
AN0310_C	0	0	0	0	0	0	0	0	0	0	0	0	0	0	0	0	0
AN0311_C	0	0	0	0	0	0	0	0	0	0	0	0	0	0	0	0	0
AN0312_C	0	0	0	0	0	0	0	0	0	0	0	0	0	0	0	0	0
AN0313_C	0	0	0	0	0	0	0	0	0	0	0	0	0	0	0	0	0
AN0314_C	0	0	0	0	0	0	0	0	0	0	0	0	0	0	0	0	0
AN0315_C	0	0	0	0	0	0	0	0	0	0	0	0	0	0	0	0	0
AN0316_C	0	0	0	0	0	0	0	0	0	0	0	0	0	0	0	0	0
AN0317_C	0	0	0	0	0	0	0	0	0	0	0	0	0	0	0	0	0
AN0318_C	0	0	0	0	0	0	0	0	0	0	0	0	0	0	0	0	0
AN0319_C	0	0	0	0	0	0	0	0	0	0	0	0	0	0	0	0	0
AN0320_C	0	0	0	0	0	0	0	0	0	0	0	0	0	0	0	0	0
AN0321_C	0	0	0	0	0	0	0	0	0	0	0	0	0	0	0	0	0
AP0002_C	0	0	0	0	0	0	0	0	0	0	0	0	0	0	0	0	0
AP0005_C	0	0	0	0	0	0	0	0	0	0	0	0	0	0	0	0	0
AP0006_C	0	0	0	0	0	0	0	0	0	0	0	0	0	0	0	0	0
AP0007_C	0	0	0	0	0	0	0	0	0	0	0	0	0	0	0	0	0
AP0008_C	0	0	0	0	0	0	0	0	0	0	0	0	0	0	0	0	0
AP0009_C	0	0	0	0	0	0	0	0	0	0	0	0	0	0	0	0	0
AP0010_C	0	0	0	0	0	0	0	0	0	0	0	0	0	0	0	0	0
AP0011_C	0	0	0	0	0	0	0	0	0	0	0	0	0	0	0	0	0
AP0014_C	0	0	0	0	0	0	0	0	0	0	0	0	0	0	0	0	0
AP0017_C	0	0	0	0	0	0	0	0	0	0	0	0	0	0	0	0	0
AP0018_C	0	0	0	0	0	0	0	0	0	0	0	0	0	0	0	0	0
AP0019_C	0	0	0	0	0	0	0	0	0	0	0	0	0	0	0	0	0
AP0020_C	0	0	0	0	0	0	0	0	0	0	0	0	0	0	0	0	0
AP0021_C	0	0	0	0	0	0	0	0	0	0	0	0	0	0	0	0	0
AP0022_C	0	0	0	0	0	0	0	0	0	0	0	0	0	0	0	0	0
AP0023_C	0	0	0	0	0	0	0	0	0	0	0	0	0	0	0	0	0
AP0024_C	0	0	0	0	0	0	0	0	0	0	0	0	0	0	0	0	0
AP0025_C	0	0	0	0	0	0	0	0	0	0	0	0	0	0	0	0	0
AP0030_C	0	0	0	0	0	0	0	0	0	0	0	0	0	0	0	0	0
AP0031_C	0	0	0	0	0	0	0	0	0	0	0	0	0	0	0	0	0
AP0032_C	0	0	0	0	0	0	0	0	0	0	0	0	0	0	0	0	0
AP0033_C	0	0	0	0	0	0	0	0	0	0	0	0	0	0	0	0	0
AP0034_C	0	0	0	0	0	0	0	0	0	0	0	0	0	0	0	0	0
AP0035_C	0	0	0	0	0	0	0	0	0	0	0	0	0	0	0	0	0
AQ0001_C	0	0	0	0	0	0	0	0	0	0	0	0	0	0	0	0	0
AQ0002_C	0	0	0	0	0	0	0	0	0	0	0	0	0	0	0	0	0
AQ0004_C	0	0	0	0	0	0	0	0	0	0	0	0	0	0	0	0	0
AQ0005_C	0	0	0	0	0	0	0	0	0	0	0	0	0	0	0	0	0
AQ0011_C	0	0	0	0	0	0	0	0	0	0	0	0	0	0	0	0	0
AQ0012_C	0	0	0	0	0	0	0	0	0	0	0	0	0	0	0	0	0
AQ0013_C	0	0	0	0	0	0	0	0	0	0	0	0	0	0	0	0	0
AQ0014_C	0	0	0	0	0	0	0	0	0	0	0	0	0	0	0	0	0
AQ0015_C	0	0	0	0	0	0	0	0	0	0	0	0	0	0	0	0	0
AR0001_C	0	0	0	0	0	0	0	0	0	0	0	0	0	0	0	0	0

	Dup 0	Dup 1	Dup 2	Dup 3	Dup 4	Dup 5	Dup 6	Dup 7	Dup 8	Dup 9	Dup 10	Dup 11	Dup 12	Dup 13	Dup 14	Dup 15	Dup 16
AR0007_C	0	0	0	0	0	0	0	0	0	0	0	0	0	0	0	0	0
AR0008_C	0	0	0	0	0	0	0	0	0	0	0	0	0	0	0	0	0
AR0009_C	0	0	0	0	0	0	0	0	0	0	0	0	0	0	0	0	0
AR0010_C	0	0	0	0	0	0	0	0	0	0	0	0	0	0	0	0	0
AR0011_C	0	0	0	0	0	0	0	0	0	0	0	0	0	0	0	0	0
AR0012_C	0	0	0	0	0	0	0	0	0	0	0	0	0	0	0	0	0
AR0013_C	0	0	0	0	0	0	1	0	0	0	0	0	0	0	0	0	0
AR0014_C	0	0	0	0	0	0	0	0	0	0	0	0	0	0	0	0	0
AR0015_C	0	0	0	0	0	0	0	0	0	0	0	0	0	0	0	0	0
AR0016_C	0	0	0	0	0	0	0	0	0	0	0	0	0	0	0	0	0
AR0017_C	0	0	0	0	0	0	0	0	0	0	0	0	0	0	0	0	0
AR0018_C	0	0	0	0	0	0	0	0	0	0	0	0	0	0	0	0	0
AR0019_C	0	0	0	0	0	0	0	0	0	0	0	0	0	0	0	0	0
AR0020_C	0	0	0	0	0	0	0	0	0	0	0	0	0	0	0	0	0
AR0021_C	0	0	0	0	0	0	0	0	0	0	0	0	0	0	0	0	0
AR0022_C	0	0	0	0	0	0	0	0	0	0	0	0	0	0	0	0	0
AR0023_C	0	0	0	0	0	0	0	0	0	0	0	0	0	0	0	0	0
AR0024_C	0	0	0	0	0	0	1	0	0	0	0	0	0	0	0	0	0
AR0026_C	0	0	0	0	0	0	0	0	0	0	0	0	0	0	0	0	0
AR0027_C	0	0	0	0	0	0	0	0	0	0	0	0	0	0	0	0	0
AR0034_C	0	0	0	0	0	0	0	0	0	0	0	0	0	0	0	0	0
AR0035_C	0	0	0	0	0	0	1	0	0	0	0	0	0	0	0	0	0
AR0036_C	0	0	0	0	0	0	2	0	0	0	0	0	0	0	0	0	0
AR0038_C	0	0	0	0	0	0	1	0	0	0	0	0	0	0	0	0	0
AR0040_C	0	0	0	0	0	0	0	0	0	0	0	0	0	0	0	0	0
AR0042_C	0	0	0	0	0	0	0	0	0	0	0	0	0	0	0	0	0
AR0043_C	0	0	0	0	0	0	0	0	0	0	0	0	0	0	0	0	0
AR0044_C	0	0	0	0	0	0	0	0	0	0	0	0	0	0	0	0	0
AR0045_C	0	0	0	0	0	0	0	0	0	0	0	0	0	0	0	0	0
AR0046_C	0	0	0	0	0	0	0	0	0	0	0	0	0	0	0	0	0
AR0047_C	0	0	0	0	0	0	0	0	0	0	0	0	0	0	0	0	0
AR0048_C	0	0	0	0	0	0	0	0	0	0	0	0	0	0	0	0	0
AR0049_C	0	0	0	0	0	0	1	0	0	0	0	0	0	0	0	0	0
AR0050_C	0	0	0	0	0	0	0	0	0	0	0	0	0	0	0	0	0
AR0051_C	0	0	0	0	0	0	0	0	0	0	0	0	0	0	0	0	0
AR0052_C	0	0	0	0	0	0	0	0	0	0	0	0	0	0	0	0	0
AR0053_C	0	0	0	0	0	0	0	0	0	0	0	0	0	0	0	0	0
AR0054_C	0	0	0	0	0	0	0	0	0	0	0	0	0	0	0	0	0
AR0057_C	0	0	0	0	0	0	0	0	0	0	0	0	0	0	0	0	0
AR0059_C	0	0	0	0	0	0	0	0	0	0	0	0	0	0	0	0	0
AR0060_C	0	0	0	0	0	0	0	0	0	0	0	0	0	0	0	0	0
AR0061_C	0	0	0	0	0	0	0	0	0	0	0	0	0	0	0	0	0
AR0062_C	0	0	0	0	0	0	0	0	0	0	0	0	0	0	0	0	0
AR0063_C	0	0	0	0	0	0	0	0	0	0	0	0	0	0	0	0	0
AR0064_C	0	0	0	0	0	0	1	0	0	0	0	0	0	0	0	0	0
AR0065_C	0	0	0	0	0	0	1	0	0	0	0	0	0	0	0	0	0
AR0066_C	0	0	0	0	0	0	0	0	0	0	0	0	0	0	0	0	0
AR0069_C	0	0	0	0	0	0	0	0	0	0	0	0	0	0	0	0	0
AR0070_C	0	0	0	0	0	0	1	0	0	0	0	0	0	0	0	0	0
AR0071_C	0	0	0	0	0	0	0	0	0	0	0	0	0	0	0	0	0
AR0072_C	0	0	0	0	0	0	0	0	0	0	0	0	0	0	0	0	0
AR0073_C	0	0	0	0	0	0	0	0	0	0	0	0	0	0	0	0	0
AR0074_C	0	0	0	0	0	0	0	0	0	0	0	0	0	0	0	0	0
AR0075_C	0	0	0	0	0	0	0	0	0	0	0	0	0	0	0	0	0
AR0076_C	0	0	0	0	0	0	0	0	0	0	0	0	0	0	0	0	0
AR0077_C	0	0	0	0	0	0	0	0	0	0	0	0	0	0	0	0	0
AR0078_C	0	0	0	0	0	0	0	0	0	0	0	0	0	0	0	0	0
AR0079_C	0	0	0	0	0	0	1	0	0	0	0	0	0	0	0	0	0
AR0080_C	0	0	0	0	0	0	0	0	0	0	0	0	0	0	0	0	0
AR0081_C	0	0	0	0	0	0	0	0	0	0	0	0	0	0	0	0	0
AR0082_C	0	0	0	0	0	0	0	0	0	0	0	0	0	0	0	0	0
AR0083_C	0	0	0	0	0	0	0	0	0	0	0	0	0	0	0	0	0
AR0084_C	0	0	0	0	0	0	0	0	0	0	0	0	0	0	0	0	0
AR0085_C	0	0	0	0	0	0	0	0	0	0	0	0	0	0	0	0	0
AR0086_C	0	0	0	0	0	0	0	0	0	0	0	0	0	0	0	0	0
AR0087_C	0	0	0	0	0	0	0	0	0	0	0	0	0	0	0	0	0
AR0088_C	0	0	0	0	0	0	0	0	0	0	0	0	0	0	0	0	0
AR0089_C	0	0	0	0	0	0	0	0	0	0	0	0	0	0	0	0	0

	Dup 0	Dup 1	Dup 2	Dup 3	Dup 4	Dup 5	Dup 6	Dup 7	Dup 8	Dup 9	Dup 10	Dup 11	Dup 12	Dup 13	Dup 14	Dup 15	Dup 16
AR0090_C	0	0	0	0	0	0	0	0	0	0	0	0	0	0	0	0	0
AR0092_C	0	0	0	0	0	0	0	0	0	0	0	0	0	0	0	0	0
AR0093_C	0	0	0	0	0	0	0	0	0	0	0	0	0	0	0	0	0
AR0095_C	0	0	0	0	0	0	0	0	0	0	0	0	0	0	0	0	0
AR0096_C	0	0	0	0	0	0	1	0	0	0	0	0	0	0	0	0	0
AR0097_C	0	0	0	0	0	0	0	0	0	0	0	0	0	0	0	0	0
AR0098_C	0	0	0	0	0	0	1	0	0	0	0	0	0	0	0	0	0
AR0099_C	0	0	0	0	0	0	0	0	0	0	0	0	0	0	0	0	0
AR0100_C	0	0	0	0	0	0	0	0	0	0	0	0	0	0	0	0	0
AS0001_C	0	0	0	0	0	0	0	0	0	0	0	0	0	0	0	0	0
AS0002_C	0	0	0	0	0	0	0	0	0	0	0	0	0	0	0	0	0
AS0003_C	0	0	0	0	0	0	0	0	0	0	0	0	0	0	0	0	0
AS0004_C	0	0	0	0	0	0	0	0	0	0	0	0	0	0	0	0	0
AS0005_C	0	0	0	0	0	0	0	0	0	0	0	0	0	0	0	0	0
AS0006_C	0	0	0	0	0	0	0	0	0	0	0	0	0	0	0	0	0
AS0007_C	0	0	0	0	0	0	0	0	0	0	0	0	0	0	0	0	0
AS0008_C	0	0	0	0	0	0	0	0	0	0	0	0	0	0	0	0	0
AS0009_C	0	0	0	0	0	0	0	0	0	0	0	0	0	0	0	0	0
AS0010_C	0	0	0	0	0	0	0	0	0	0	0	0	0	0	0	0	0
AS0011_C	0	0	0	0	0	0	0	0	0	0	0	0	0	0	0	0	0
AS0012_C	0	0	0	0	0	0	0	0	0	0	0	0	0	0	0	0	0
AS0013_C	0	0	0	0	0	0	0	0	0	0	0	0	0	0	0	0	0
AS0014_C	0	0	0	0	0	0	0	0	0	0	0	0	0	0	0	0	0
AS0015_C	0	0	0	0	0	0	0	0	0	0	0	0	0	0	0	0	0
AS0016_C	0	0	0	0	0	0	0	0	0	0	0	0	0	0	0	0	0
AS0017_C	0	0	0	0	0	0	0	0	0	0	0	0	0	0	0	0	0
AS0018_C	0	0	0	0	0	0	0	0	0	0	0	0	0	0	0	0	0
AS0019_C	0	0	0	0	0	0	0	0	0	0	0	0	0	0	0	0	0
AS0020_C	0	0	0	0	0	0	0	0	0	0	0	0	0	0	0	0	0
AS0021_C	0	0	0	0	0	0	0	0	0	0	0	0	0	0	0	0	0
AS0022_C	0	0	0	0	0	0	0	0	0	0	0	0	0	0	0	0	0
AS0024_C	0	0	0	0	0	0	0	0	0	0	0	0	0	0	0	0	0
AS0025_C	0	0	0	0	0	0	0	0	0	0	0	0	0	0	0	0	0
AS0026_C	0	0	0	0	0	0	0	0	0	0	0	0	0	0	0	0	0
AS0027_C	0	0	0	0	0	0	0	0	0	0	0	0	0	0	0	0	0
AS0028_C	0	0	0	0	0	0	0	0	0	0	0	0	0	0	0	0	0
AS0029_C	0	0	0	0	0	0	0	0	0	0	0	0	0	0	0	0	0
AS0030_C	0	0	0	0	0	0	0	0	0	0	0	0	0	0	0	0	0
AS0032_C	0	0	0	0	0	0	0	0	0	0	0	0	0	0	0	0	0
AS0033_C	0	0	0	0	0	0	0	0	0	0	0	0	0	0	0	0	0
AS0034_C	0	0	0	0	0	0	0	0	0	0	0	0	0	0	0	0	0
AS0035_C	0	0	0	0	0	0	0	0	0	0	0	0	0	0	0	0	0
AS0036_C	0	0	0	0	0	0	0	0	0	0	0	0	0	0	0	0	0
AS0037_C	0	0	0	0	0	0	0	0	0	0	0	0	0	0	0	0	0
AS0039_C	0	0	0	0	0	0	0	0	0	0	0	0	0	0	0	0	0
AS0040_C	0	0	0	0	0	0	0	0	0	0	0	0	0	0	0	0	0
AS0041_C	0	0	0	0	0	0	0	0	0	0	0	0	0	0	0	0	0
AS0042_C	0	0	0	0	0	0	0	0	0	0	0	0	0	0	0	0	0
AS0044_C	0	0	0	0	0	0	0	0	0	0	0	0	0	0	0	0	0
AS0045_C	0	0	0	0	0	0	0	0	0	0	0	0	0	0	0	0	0
AS0046_C	0	0	0	0	0	0	0	0	0	0	0	0	0	0	0	0	0
AS0047_C	0	0	0	0	0	0	0	0	0	0	0	0	0	0	0	0	0
AS0048_C	0	0	0	0	0	0	0	0	0	0	0	0	0	0	0	0	0
AS0049_C	0	0	0	0	0	0	0	0	0	0	0	0	0	0	0	0	0
AS0051_C	0	0	0	0	0	0	0	0	0	0	0	0	0	0	0	0	0
AS0052_C	0	0	0	0	0	0	0	0	0	0	0	0	0	0	0	0	0
AS0053_C	0	0	0	0	0	0	0	0	0	0	0	0	0	0	0	0	0
AS0054_C	0	0	0	0	0	0	0	0	0	0	0	0	0	0	0	0	0
AS0055_C	0	0	0	0	0	0	0	0	0	0	0	0	0	0	0	0	0
AS0056_C	0	0	0	0	0	0	0	0	0	0	0	0	0	0	0	0	0
AS0057_C	0	0	0	0	0	0	0	0	0	0	0	0	0	0	0	0	0
AS0058_C	0	0	0	0	0	0	0	0	0	0	0	0	0	0	0	0	0
AS0059_C	0	0	0	0	0	0	0	0	0	0	0	0	0	0	0	0	0
AS0060_C	0	0	0	0	0	0	0	0	0	0	0	0	0	0	0	0	0
AS0062_C	0	0	0	0	0	0	0	0	0	0	0	0	0	0	0	0	0
AS0064_C	0	0	0	0	0	0	0	0	0	0	0	0	0	0	0	0	0
AS0065_C	0	0	0	0	0	0	0	0	0	0	0	0	0	0	0	0	0
AS0066_C	0	0	0	0	0	0	0	0	0	0	0	0	0	0	0	0	0

	Dup 0	Dup 1	Dup 2	Dup 3	Dup 4	Dup 5	Dup 6	Dup 7	Dup 8	Dup 9	Dup 10	Dup 11	Dup 12	Dup 13	Dup 14	Dup 15	Dup 16
AS0068_C	0	0	0	0	0	0	0	0	0	0	0	0	0	0	0	0	0
AS0069_C	0	0	0	0	0	0	0	0	0	0	0	0	0	0	0	0	0
AS0070_C	0	0	0	0	0	0	0	0	0	0	0	0	0	0	0	0	0
AS0071_C	0	0	0	0	0	0	0	0	0	0	0	0	0	0	0	0	0
AS0072_C	0	0	0	0	0	0	0	0	0	0	0	0	0	0	0	0	0
AS0073_C	0	0	0	0	0	0	0	0	0	0	0	0	0	0	0	0	0
AS0074_C	0	0	0	0	0	0	0	0	0	0	0	0	0	0	0	0	0
AS0076_C	0	0	0	0	0	0	0	0	0	0	0	0	0	0	0	0	0
AS0077_C	0	0	0	0	0	0	0	0	0	0	0	0	0	0	0	0	0
AS0078_C	0	0	0	0	0	0	0	0	0	0	0	0	0	0	0	0	0
AV0001_C	0	0	0	0	0	0	0	0	0	0	0	3	0	0	0	0	0
AV0002_C	0	0	0	0	0	0	0	0	0	0	0	3	0	0	0	0	0
AV0003_C	0	0	0	0	0	0	0	0	0	0	0	2	0	0	0	0	0
AV0004_C	0	0	0	0	0	0	0	0	0	0	0	0	0	0	0	0	0
AV0005_C	0	0	0	0	0	0	0	0	0	0	0	0	0	0	0	3	0
AV0006_C	0	0	0	0	0	0	0	0	0	0	0	2	0	0	0	0	0
AV0007_C	0	0	0	0	0	0	0	0	0	0	0	0	0	0	0	0	0
AV0008_C	0	0	0	0	0	0	0	0	0	0	0	0	0	0	0	3	0
AV0009_C	0	0	0	0	0	0	0	0	0	0	0	0	0	0	0	5	0
AV0010_C	0	0	0	0	0	0	0	0	0	0	0	2	0	0	0	0	0
AV0011_C	0	0	0	0	0	0	0	0	0	0	0	2	0	0	0	0	0
AV0012_C	0	0	0	0	0	0	0	0	0	0	0	2	0	0	0	0	0
AV0013_C	0	0	0	0	0	0	0	0	0	0	0	0	0	0	0	2	0
AV0014_C	0	0	0	0	0	0	0	0	0	0	0	1	0	0	0	0	0
AV0015_C	0	0	0	0	0	0	0	0	0	0	0	5	0	0	0	0	0
AV0017_C	0	0	0	0	0	0	0	0	0	0	0	2	0	0	0	0	0
AV0018_C	0	0	0	0	0	0	0	0	0	0	0	0	0	0	0	0	0
AV0020_C	0	0	0	0	0	0	0	0	0	0	0	0	0	0	0	2	0
AV0021_C	0	0	0	0	0	0	0	0	0	0	0	2	0	0	0	0	0
AV0022_C	0	0	0	0	0	0	0	0	0	0	0	0	0	0	0	2	0
AV0024_C	0	0	0	0	0	0	0	0	0	0	0	0	0	0	0	0	0
AV0025_C	0	0	0	0	0	0	0	0	0	0	0	2	0	0	0	0	0
AV0026_C	0	0	0	0	0	0	0	0	0	0	0	0	0	0	0	1	0
AV0027_C	0	0	0	0	0	0	0	0	0	0	0	0	0	0	0	0	0
AV0028_C	0	0	0	0	0	0	0	0	0	0	0	2	0	0	0	0	0
AV0029_C	0	0	0	0	0	0	0	0	0	0	0	0	0	2	0	2	0
AV0030_C	0	0	0	0	0	0	0	0	0	0	0	0	0	0	0	0	0
AV0031_C	0	0	0	0	0	0	0	0	0	0	0	2	0	0	0	0	0
AV0032_C	0	0	0	0	0	0	0	0	0	0	0	2	0	0	0	0	0
AV0033_C	0	0	0	0	0	0	0	0	0	0	0	3	0	0	0	0	0
AV0034_C	0	0	0	0	0	0	0	0	0	0	0	4	0	0	0	0	0
AV0035_C	0	0	0	0	0	0	0	0	0	0	0	2	0	0	0	0	0
AV0036_C	0	0	0	0	1	0	0	0	0	0	0	0	0	0	0	2	0
AV0037_C	0	0	0	0	0	0	0	0	0	0	0	2	0	0	0	0	0
AV0038_C	0	0	0	0	0	0	0	0	0	0	0	0	0	0	0	0	0
AV0039_C	0	0	0	0	2	0	0	0	0	0	0	0	0	0	0	0	0
AV0040_C	0	0	0	0	0	0	0	0	0	0	0	0	0	0	0	0	0
AV0041_C	0	0	0	0	0	0	0	0	0	0	0	0	0	0	0	3	0
AV0042_C	0	0	0	0	0	0	0	0	0	0	0	0	0	0	0	0	0
AV0043_C	0	0	0	0	0	0	0	0	0	0	0	2	0	0	0	0	0
AV0044_C	0	0	0	0	0	0	0	0	0	0	0	2	0	0	0	1	0
AV0045_C	0	0	0	0	0	0	0	0	0	0	0	0	0	0	0	3	0
AV0046_C	0	0	0	0	0	0	0	0	0	0	0	0	0	0	0	0	0
AV0047_C	0	0	0	0	0	0	0	0	0	0	0	0	0	0	0	0	0
AY0006_C	0	0	0	0	0	0	0	0	0	0	0	0	0	0	0	0	0
AY0007_C	0	0	0	0	0	0	0	0	0	0	0	0	0	0	0	0	0
AY0010_C	0	0	0	0	0	0	0	0	0	0	0	0	0	0	0	0	0
AY0011_C	0	0	0	0	0	0	0	0	0	0	0	0	0	0	0	0	0
AY0012_C	0	0	0	0	0	0	0	0	0	0	0	0	0	0	0	0	0
AY0013_C	0	0	0	0	0	0	0	0	0	0	0	0	0	0	0	0	0
AY0015_C	0	0	0	0	0	0	0	0	0	0	0	0	0	0	0	0	0
AY0016_C	0	0	0	0	0	0	0	0	0	0	0	0	0	0	0	0	0
AY0017_C	0	0	0	0	0	0	0	0	0	0	0	0	0	0	0	0	0
AY0018_C	0	0	0	0	0	0	0	0	0	0	0	0	0	0	0	0	0
AY0019_C	0	0	0	0	0	0	0	0	0	0	0	0	0	0	0	0	0
AY0020_C	0	0	0	0	0	0	0	0	0	0	0	0	0	0	0	0	0
AY0021_C	0	0	0	0	0	0	0	0	0	0	0	0	0	0	0	0	0
AY0023_C	0	0	0	0	0	0	0	0	0	0	0	0	0	0	0	0	0

	Dup 0	Dup 1	Dup 2	Dup 3	Dup 4	Dup 5	Dup 6	Dup 7	Dup 8	Dup 9	Dup 10	Dup 11	Dup 12	Dup 13	Dup 14	Dup 15	Dup 16
AY0024_C	0	0	0	0	0	0	0	0	0	0	0	0	0	0	0	0	0
AY0025_C	0	0	0	0	0	0	0	0	0	0	0	0	0	0	0	0	0
AY0026_C	0	0	0	0	0	0	0	0	0	0	0	0	0	0	0	0	0
AY0027_C	0	0	0	0	0	0	0	0	0	0	0	0	0	0	0	0	0
AY0029_C	0	0	0	0	0	0	0	0	0	0	0	0	0	0	0	0	0
AY0031_C	0	0	0	0	0	0	0	0	0	0	0	0	0	0	0	0	0
AY0032_C	0	0	0	0	0	0	0	0	0	0	0	0	0	0	0	0	0
AY0033_C	0	0	0	0	0	0	0	0	0	0	0	0	0	0	0	0	0
AY0034_C	0	0	0	0	0	0	0	0	0	0	0	0	0	0	0	0	0
AY0035_C	0	0	0	0	0	0	0	0	0	0	0	0	0	0	0	0	0
AY0036_C	0	0	0	0	0	0	0	0	0	0	0	0	0	0	0	0	0
AY0038_C	0	0	0	0	0	0	0	0	0	0	0	0	0	0	0	0	0
AY0039_C	0	0	0	0	0	0	0	0	0	0	0	0	0	0	0	0	0
AY0040_C	0	0	0	0	0	0	0	0	0	0	0	0	0	0	0	0	0
AY0041_C	0	0	0	0	0	0	0	0	0	0	0	0	0	0	0	0	0
AY0042_C	0	0	0	0	0	0	0	0	0	0	0	0	0	0	0	0	0
AY0043_C	0	0	0	0	0	0	0	0	0	0	0	0	0	0	0	0	0
AY0045_C	0	0	0	0	0	0	0	0	0	0	0	0	0	0	0	0	0
AY0046_C	0	0	0	0	0	0	0	0	0	0	0	0	0	0	0	0	0
AY0047_C	0	0	0	0	0	0	0	0	0	0	0	0	0	0	0	0	0
AY0048_C	0	0	0	0	0	0	0	0	0	0	0	0	0	0	0	0	0
AY0049_C	0	0	0	0	0	0	0	0	0	0	0	0	0	0	0	0	0
AY0050_C	0	0	0	0	0	0	0	0	0	0	0	0	0	0	0	0	0
AY0052_C	0	0	0	0	0	0	0	0	0	0	0	0	0	0	0	0	0
AY0053_C	0	0	0	0	0	0	0	0	0	0	0	0	0	0	0	0	0
AY0054_C	0	0	0	0	0	0	0	0	0	0	0	0	0	0	0	0	0
AY0055_C	0	0	0	0	0	0	0	0	0	0	0	0	0	0	0	0	0
AY0056_C	0	0	0	0	0	0	0	0	0	0	0	0	0	0	0	0	0
AY0057_C	0	0	0	0	0	0	0	0	0	0	0	0	0	0	0	0	0
AY0058_C	0	0	0	0	0	0	0	0	0	0	0	0	0	0	0	0	0
AY0059_C	0	0	0	0	0	0	0	0	0	0	0	0	0	0	0	0	0
AY0060_C	0	0	0	0	0	0	0	0	0	0	0	0	0	0	0	0	0
AY0061_C	0	0	0	0	0	0	0	0	0	0	0	0	0	0	0	0	0
AY0062_C	0	0	0	0	0	0	0	0	0	0	0	0	0	0	0	0	0
AY0063_C	0	0	0	0	0	0	0	0	0	0	0	0	0	0	0	0	0
AY0064_C	0	0	0	0	0	0	0	0	0	0	0	0	0	0	0	0	0
AY0065_C	0	0	0	0	0	0	0	0	0	0	0	0	0	0	0	0	0
AY0066_C	0	0	0	0	0	0	0	0	0	0	0	0	0	0	0	0	0
AY0067_C	0	0	0	0	0	0	0	0	0	0	0	0	0	0	0	0	0
AY0068_C	0	0	0	0	0	0	0	0	0	0	0	0	0	0	0	0	0
AY0069_C	0	0	0	0	0	0	0	0	0	0	0	0	0	0	0	0	0
AY0070_C	0	0	0	0	0	0	0	0	0	0	0	0	0	0	0	0	0
AY0072_C	0	0	0	0	0	0	0	0	0	0	0	0	0	0	0	0	0
AY0074_C	0	0	0	0	0	0	0	0	0	0	0	0	0	0	0	0	0
AY0076_C	0	0	0	0	0	0	0	0	0	0	0	0	0	0	0	0	0
AY0077_C	0	0	0	0	0	0	0	0	0	0	0	0	0	0	0	0	0
AY0078_C	0	0	0	0	0	0	0	0	0	0	0	0	0	0	0	0	0
AY0079_C	0	0	0	0	0	0	0	0	0	0	0	0	0	0	0	0	0
AY0080_C	0	0	0	0	0	0	0	0	0	0	0	0	0	0	0	0	0
AY0082_C	0	0	0	0	0	0	0	0	0	0	0	0	0	0	0	0	0
AY0083_C	0	0	0	0	0	0	0	0	0	0	0	0	0	0	0	0	0
AY0085_C	0	0	0	0	0	0	0	0	0	0	0	0	0	0	0	0	0
AY0087_C	0	0	0	0	0	0	0	0	0	0	0	0	0	0	0	0	0
AY0088_C	0	0	0	0	0	0	0	0	0	0	0	0	0	0	0	0	0
AY0089_C	0	0	0	0	0	0	0	0	0	0	0	0	0	0	0	0	0
AY0090_C	0	0	0	0	0	0	0	0	0	0	0	0	0	0	0	0	0
AY0091_C	0	0	0	0	0	0	0	0	0	0	0	0	0	0	0	0	0
