# Supplementary material for: Whole-genome sequencing reveals high complexity of copy number variation at insecticide resistance loci in malaria mosquitoes
Source: Genome Res. 2019 Aug;29(8):1250–61. doi: 10.1101/gr.245795.118 (PMC6673711; doi:10.1101/gr.245795.118)
Supplement: Supplemental Material [file supp_gr.245795.118_Supplementary_Data_S8.pdf]

Whole genome sequencing reveals high complexity of copy number variation at insecticide resistance loci in malaria mosquitoes

Eric R. Lucas, Alistair Miles, Nicholas J. Harding, Chris S. Clarkson, Mara K. N. Lawniczak, Dominic P. Kwiatkowski, David Weetman, Martin J. Donnelly and The *Anopheles gambiae* 1000 Genomes Consortium

Electronic Supplementary Material

Supplementary Data S8. Haplotype background analysis of CNV alleles.

To explore the possibility that some different CNV alleles represent the same original CNV event followed by a mutation disrupting the breakpoint, we built haplotype clustering dendrograms of the CNVs alleles. This required phased CNV data, and thus could not be performed for all of the CNV alleles (see main text section ...). To build the haplotype dendrograms, we used the first 1000 SNPs upstream and downstream of the region over which CNVs were found in any samples (the region containing CNVs were excluded as the presence of CNVs could disrupt the genotype calls and subsequent phasing). This distance between the SNPs used for creation of the dendrogram and the CNVs themselves increases the possibility of recombination between the haplotypes and the CNV alleles, leading to greater uncertainty for the haplotypes in the dendrogram.

A CNV alleles arising as a result of the disruption of another CNV's breakpoint would be reflected in the cluster representing by the former CNV being nested within the cluster representing the latter. Overall, most CNVs did not follow this pattern, but there were a few examples where this might be the case.

In the *Gstu* - *Gste* cluster, Gstue_Dup10 is nested within the cluster representing Cyp6aap_Dup3 (Fig. **DataS8.1**), suggesting that Cyp6aap_Dup10 resulted as a mutation of Cyp6aap_Dup3. However, there is no overlap between the genomic regions covered by Dup3 (*Gstu4*) and Dup10 (the *Gste* cluster). Is is therefore unlikely that either duplication occured as a mutation of the other. All other duplication form single clusters.

In *Cyp9k1*, with the exception of two individual haplotypes of Dup4 and Dup9 that occur

apart from the rest of their respective clusters, all CNVs form single clusters and thus there is no evidence of multiple CNVs sharing the some origin event (Fig. **DataS8.2**).

In the *Cyp6aa - Cyp6p* cluster, Cyp6aap_Dup10 is nested within the cluster representing Cyp6aap_Dup15 (Fig. **DataS8.3**), suggesting that Cyp6aap_Dup10 may have resulted as a mutation of Cyp6aap_Dup15. Similarly, Cyp6aap_Dup3 and Cyp6aap_Dup5 may have resulted as a mutation of Cyp6aap_Dup1. Cyp6aap_Dup2, Cyp6aap_Dup6, Cyp6aap_Dup8, Cyp6aap_Dup9 and Cyp6aap_Dup12 are also nested within the cluster representing Cyp6aap_Dup1, but are unlikely to be mutations of Cyp6aap_Dup1 (Dup2 has no overlap with Dup1, while Dup8, Dup9 and Dup12 all extend beyond Dup1 both downstream and upstream, and would thus have required indepedent extensions of the duplication in two directions.

Cyp6aap_Dup7 and Cyp6aap_Dup14 together form a mixed cluster (Fig. **DataS8.3**). However, Dup7 is a tandem inversion, while Dup14 is a tandem duplication (Supplementary Data S4), making it unlikely that a deletion around the breakpoint could have created one from the other. Furthermore, neither duplication forms a single cluster emerging from the other, as would be expected if a mutation event caused the creation of Dup7 from Dup14, or vice-versa. A more likely explanation for this pattern is recombination of the haplotypes of these two CNVs. Phasing of the CNVs revealed that Dup7 and Dup14 were often found alongside each-other on the same haplotype, indicating either that one of these CNVs occured on the background of the other, or that recombination combined the two CNVs. The resulting shared haplotype background would be compatible with the shared pattern that is observed. Recombination of two overlapping CNVs onto the same chromosome has previously been documented in *Plasmodium falciparum* (Miles et al. 2016).

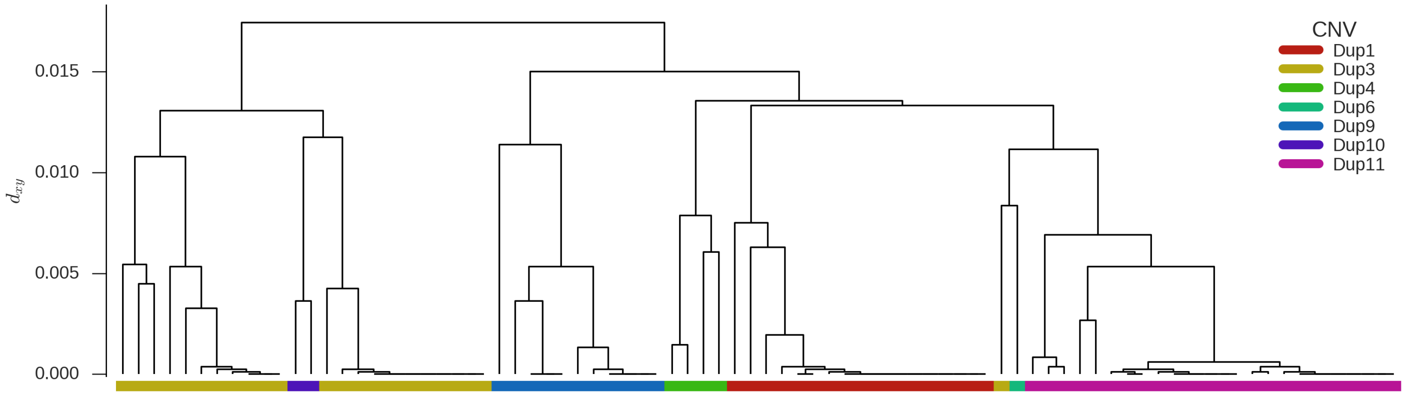

Fig. DataS8.1: Haplotype clustering dendrogram for CNVs found in the *Gstu-Gste* cluster.

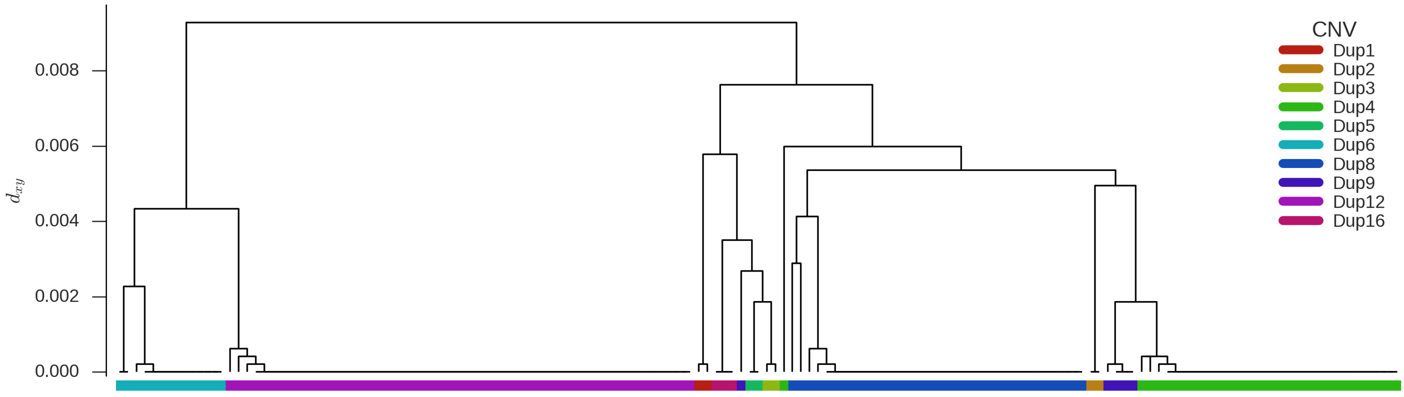

Fig. DataS8.2: Haplotype clustering dendrogram for CNVs found around *Cyp9k1*.

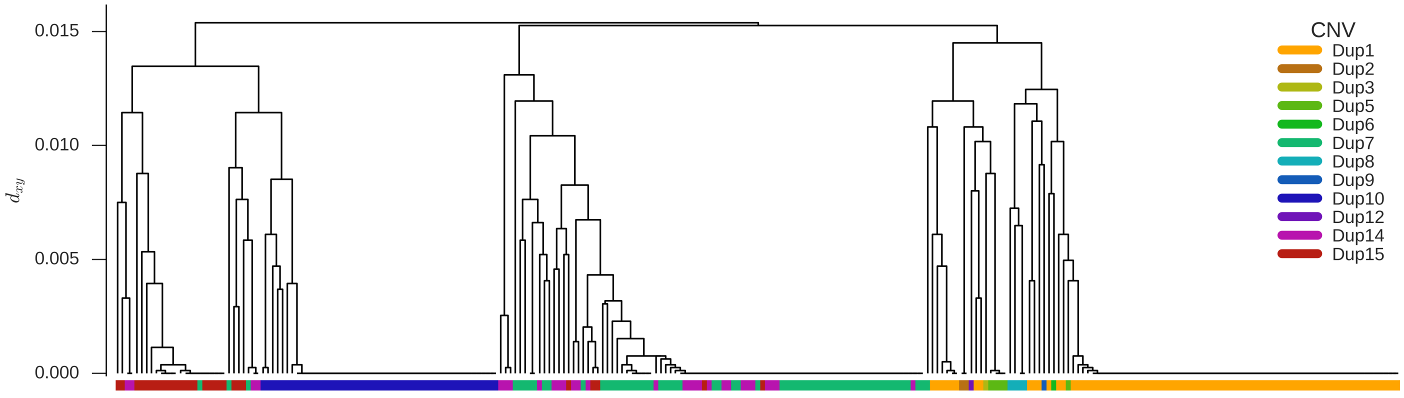

Fig. DataS8.3: Haplotype clustering dendrogram for CNVs found in the *Cyp6aa-Cyp6p* cluster.

References

Miles, A. et al. (2016). Indels, structural variation, and recombination drive genomic diversity in *Plasmodium falciparum*. *Genome research* 26(9):1288–1299.
