# Supplementary material for: Whole-genome sequencing reveals high complexity of copy number variation at insecticide resistance loci in malaria mosquitoes
Source: Genome Res. 2019 Aug;29(8):1250–61. doi: 10.1101/gr.245795.118 (PMC6673711; doi:10.1101/gr.245795.118)
Supplement: Supplemental Material [file supp_gr.245795.118_Supplementary_Data_S11.pdf]

Whole genome sequencing reveals high complexity of copy number variation at insecticide resistance loci in malaria mosquitoes

Eric R. Lucas, Alistair Miles, Nicholas J. Harding, Chris S. Clarkson, Mara K. N. Lawniczak, Dominic P. Kwiatkowski, David Weetman, Martin J. Donnelly and The *Anopheles gambiae* 1000 Genomes Consortium

Electronic Supplementary Material
Supplementary Data S11. Validation of CNV detection method.

Estimation of method specificity

To estimate the number of CNVs that could be detected by random chance using our method, we performed 100 simulations in which the coverage for every 300 bp window on chromosome 2R was randomly shuffled. We restricted our randomisations to a single chromosome in order to reduce computing load, and chose chromosome 2R as it is the largest chromsome arm in the *An. gambiae* genome. One of the filtering steps in our detection method removes CNVs that are not present in a sufficiently high proportion of samples in the population, thus requiring the same CNV to be detected in multiple individuals. Within each simulation, we therefore applied the same order of shuffling to all 1142 samples, thus maximising the chances that any randomly generated series of high coverage windows would be repeated across multiple samples.

In all 100 simulations, significantly fewer CNVs were found than in the real data. In the real data, the number of CNVs found in an individual sample ranged from 15 to 113, with a median of 25 and a mean of 26.3. Accross the 100 simulation, the median number of CNVs found per individual was always 0 (ie: more than half of the individuals had no CNVs), and the mean ranged from 0.04 to 0.23. The most CNVs found in any

individual in any of the simulations was 7, fewer than the minimum observed in the real data. Comparing the mean number of CNVs found per sample across all 100 simulations (0.089) with that observed in the real data (26.3), suggests a false discovery rate of 0.003 for individual CNVs found in a sample.

In the real data on chromosome 2R, we found 271 different CNVs after removing those that were not found at sufficiently high frequency within a population. Out of the 100 simulations, this value ranged from 0 to 9, with a mean of 3.6. This indicates a false discovery rate of 0.013 for CNVs identified in the dataset.

Estimation of method sensitivity

To estimate the sensitivity of our method for detecting real CNVs, we repeated the simulations above while adding randomly generated CNVs. In each population in the dataset, we created 10 CNVs of 5 consecutive windows (the minimum size detected by our method), 10 CNVs of 10 consecutive windows, 10 CNVs of 15 consecutive windows and 10 CNVs of 20 consecutive windows. Each CNV was allocated to 20% of the individuals in the population (rounded up), chosen at random. The position of the CNV on chromosome 2R was chosen at random. For each individual to which the CNV was allocated, the observed coverage in the affected windows was multiplied by 1.5, thus simulating a heterozygote for the CNV (single extra copy of the gene compared to the normal copy number of 2).

We considered that a CNV was correctly recovered if at least 50% of the windows that comprised it overlaped with a detected CNV. Across 100 simulations, the proportion of CNVs that were recovered had a mean of 31.4% (range 24.3% to 0.37.3%) for 5-window CNVs; 85% (range 80.1% to 89.1%) for 10-window CNVs; 94.2% (range 91.3% to 96.2%) for 15-window CNVs and 97.9% (range 96.6% to 99.1%) for 20-window CNVs.

We also calculated the number of CNVs that were identified at the population-level after filtering CNVs that were not found at sufficiently high frequency (5% of samples in the population, or 3 samples in populations smaller than 40), as done on the real data. For this calculation, we excluded two populations where the sample size was smaller than 11 (*An. coluzzii* from Guinea and *An. gambiae* from Equatorial Guinea), because the number of samples to which each artificial CNV was allocated (20%) was smaller than the minimum of 3 required to be pass our filtering process). Across 100 simulations, the proportion of CNVs that were recovered at the population level had a mean of 42.2% (range 31.4% to 50.7%) for 5-window CNVs; 83% (range 77.1% to 88.6%) for 10-window CNVs; 86% (range 82.1% to 90.7%) for 15-window CNVs and 86.4% (range 82.1% to 92.1%) for 20-window CNVs.
