# Supplementary material for: Whole-genome sequencing reveals high complexity of copy number variation at insecticide resistance loci in malaria mosquitoes
Source: Genome Res. 2019 Aug;29(8):1250–61. doi: 10.1101/gr.245795.118 (PMC6673711; doi:10.1101/gr.245795.118)
Supplement: Supplemental Material [file supp_gr.245795.118_Supplementary_Figures_and_tables.pdf]

Whole genome sequencing reveals high complexity of copy number variation at insecticide resistance loci in malaria mosquitoes

Eric R. Lucas, Alistair Miles, Nicholas J. Harding, Chris S. Clarkson, Mara K. N. Lawniczak, Dominic P. Kwiatkowski, David Weetman, Martin J. Donnelly and The *Anopheles gambiae* 1000 Genomes Consortium

Electronic Supplementary Material

Supplementary figures and tables

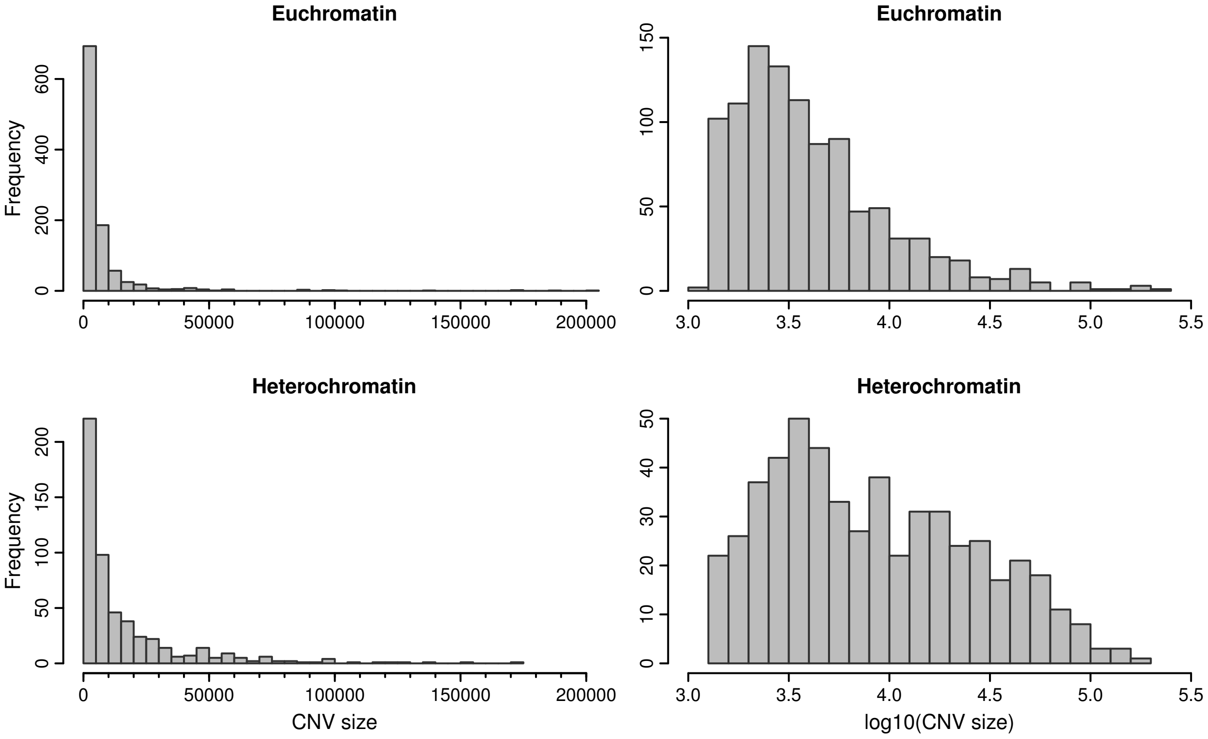

Fig. S1: Heterochromatic CNVs are significantly larger than euchromatic CNVs (Wilcoxon test: $W = 377190, P < 0.0001$).

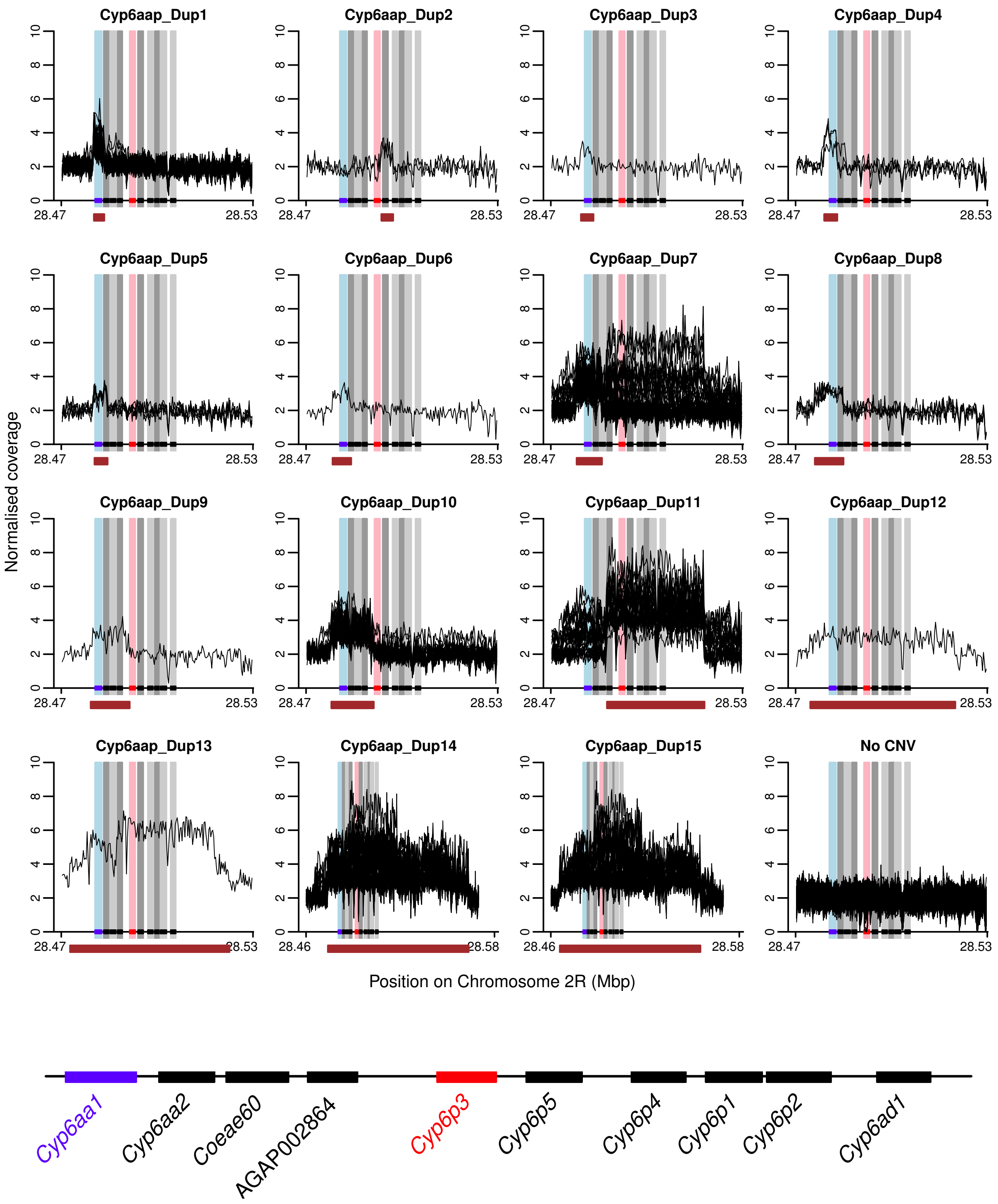

Fig. S2: CNV alleles detected around *Cyp6p3* and *Cyp6aa1*. Plots show coverage traces for samples identified as carrying each of the 15 CNV alleles using discordant reads and breakpoint reads. Bead plots and vertical grey bars show the position of the genes in the cluster, with *Cyp6p3* and *Cyp6aa1* highlighted in red and blue respectively. The magnified bead plot at the bottom shows the position of each gene along the chromosome. Brown horizontal bars show the extent of each CNV, as determined by its breakpoints (no starting breakpoint could be determined for Dup15, and so the starting point is estimated from coverage). There was substantial overlap in samples carrying Dup7, Dup11, Dup14 and Dup15, thus the coverage traces for each of these CNVs often include signals from the other three. Details on these CNVs can be found in Suppelementary Data S4.

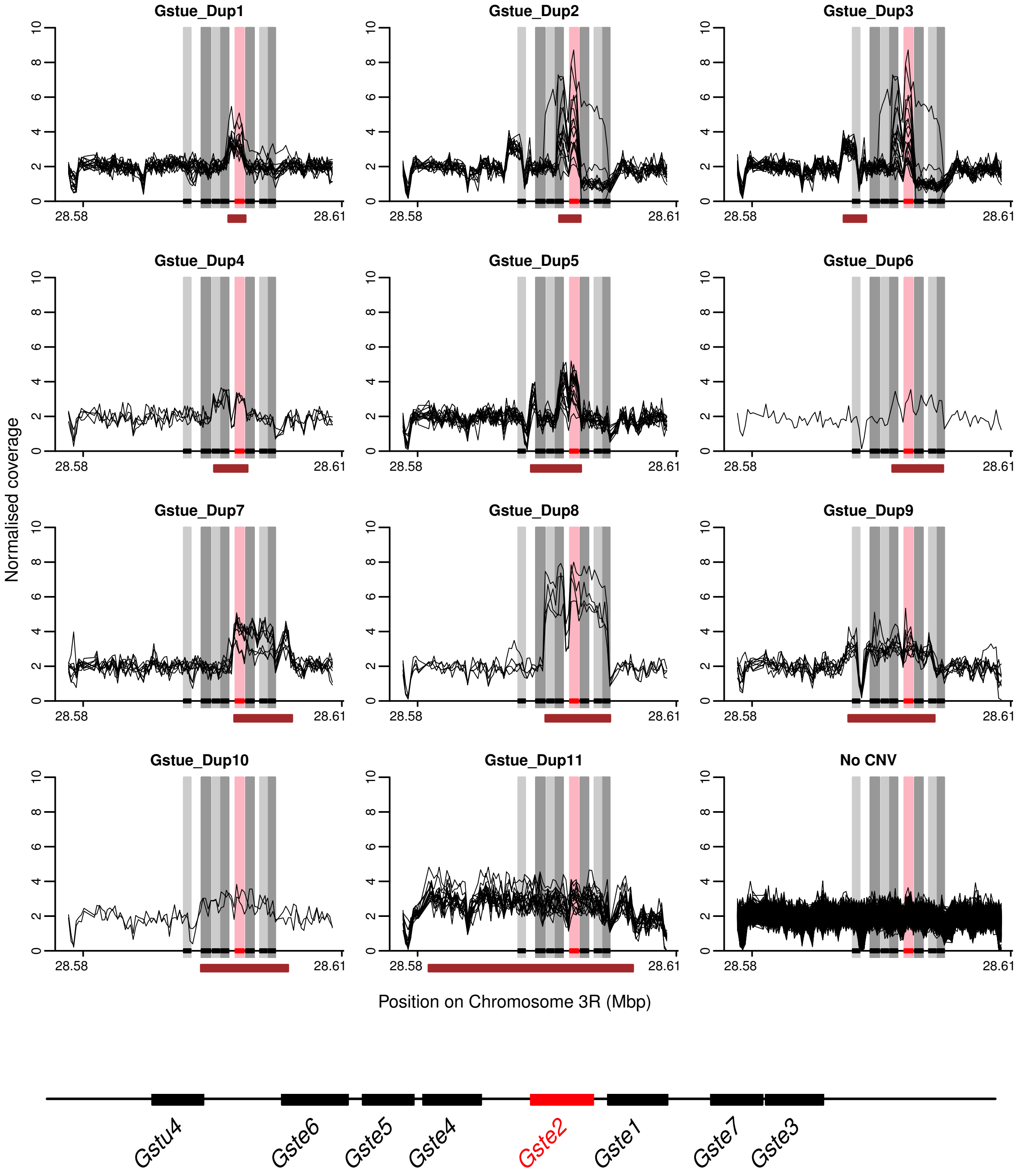

Fig. S3: CNV alleles detected around *Gste2*. Plots show coverage traces for samples identified as carrying each of the eleven CNV alleles using discordant reads and breakpoint reads. Bead plots and vertical grey bars show the position of the *Gst* genes, with *Gste2* highlighted in red. The magnified bead plot at the bottom shows the position of each gene along the chromosome. Brown horizontal bars show the extent of each CNV, as determined by its breakpoints. Traces for Dup2 and Dup3 are similar because the former always occurs on the background of the latter (ie: all samples that carry Dup2 also carry Dup3). Details on these CNVs can be found in Supplementary Data S5.

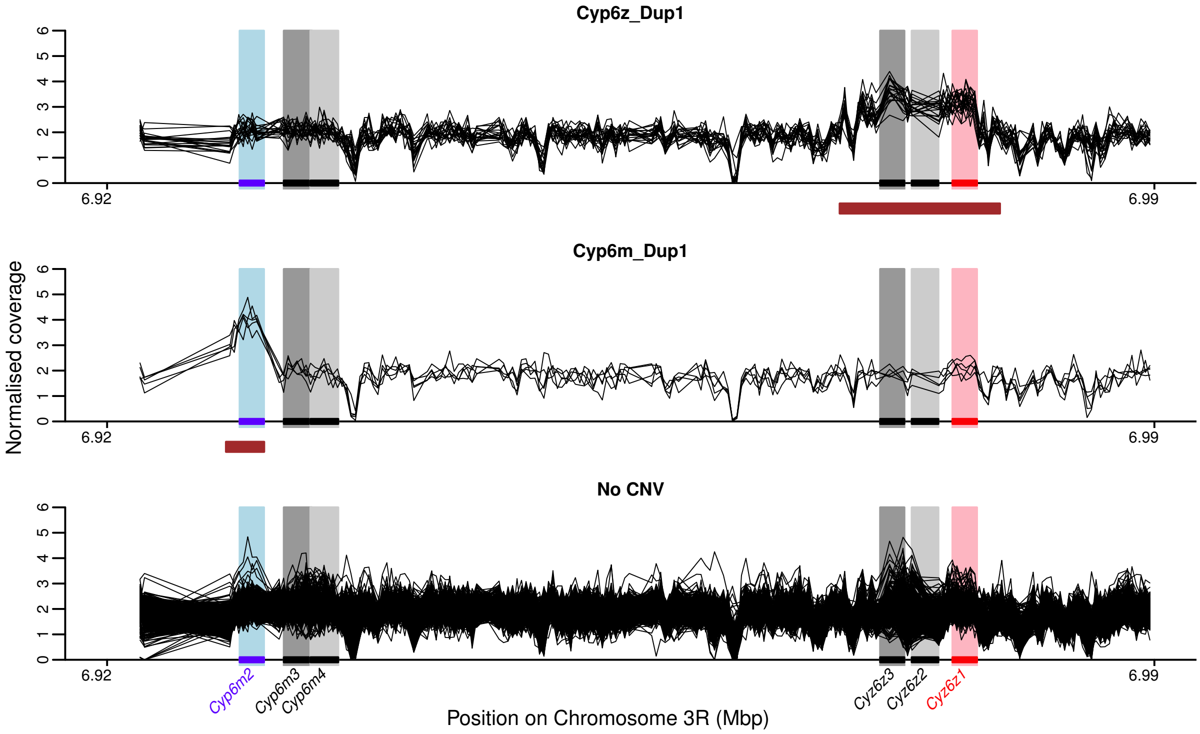

Fig. S4: CNV alleles detected around *Cyp6m2* and *Cyp6z1*. Plots show coverage traces for samples identified as carrying each of the two CNV alleles using discordant reads and breakpoint reads. The region to the left of *Cyp6m2* has very low accessibility. Bead plots and vertical grey bars show the position of the genes in the clusters, with *Cyp6m2* and *Cyp6z1* highlighted in blue and red respectively. Brown horizontal bars show the extent of each CNV, as determined by its breakpoints (no end breakpoint could be determined for Cyp6m_Dup1, so this breakpoint was estimated from coverage). Details on these CNVs can be found in Supplementary Data S6.

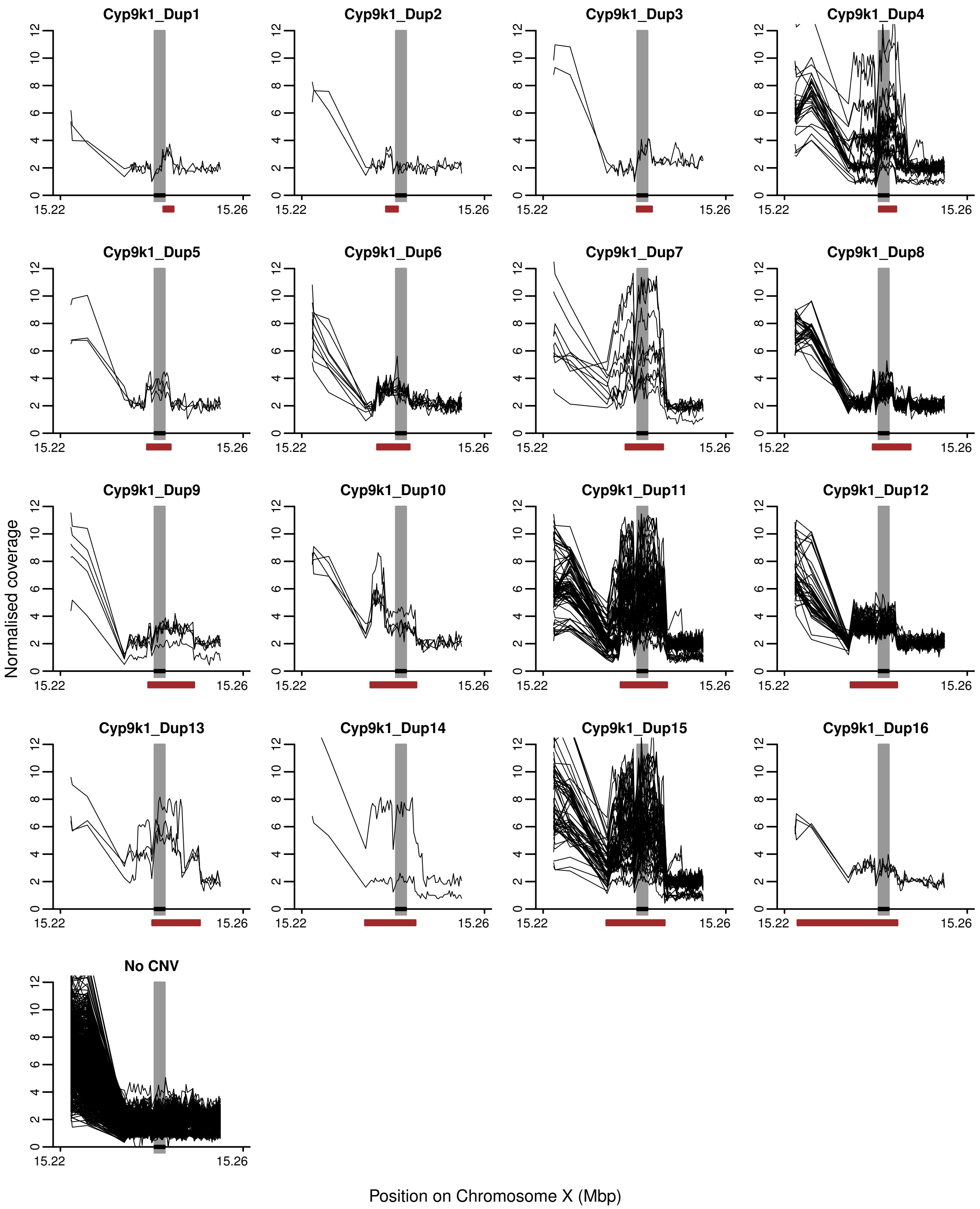

Fig. S5: CNV alleles detected around *Cyp9k1*. Plots show coverage traces for samples identified as carrying each of the 16 CNV alleles using discordant reads and breakpoint reads. The region to the left of the plot has very low accessibility and erratic coverage. Traces with a background coverage of 1 are males. Bead plots and vertical grey bars show the position of *Cyp9k1*. Brown horizontal bars show the extent of each CNV, as determined by its breakpoints (no breakpoints could be determined for Dup7, and only end breakpoints could be determined for Dup3, Dup14, Dup15 and Dup16, so these breakpoints are estimated from coverage or face-away reads). There was substantial overlap in samples carrying Dup4, Dup11, Dup15, thus the coverage traces for each of these CNVs often include signals from the other two. Details on these CNVs can be found in Supplementary Data S7.

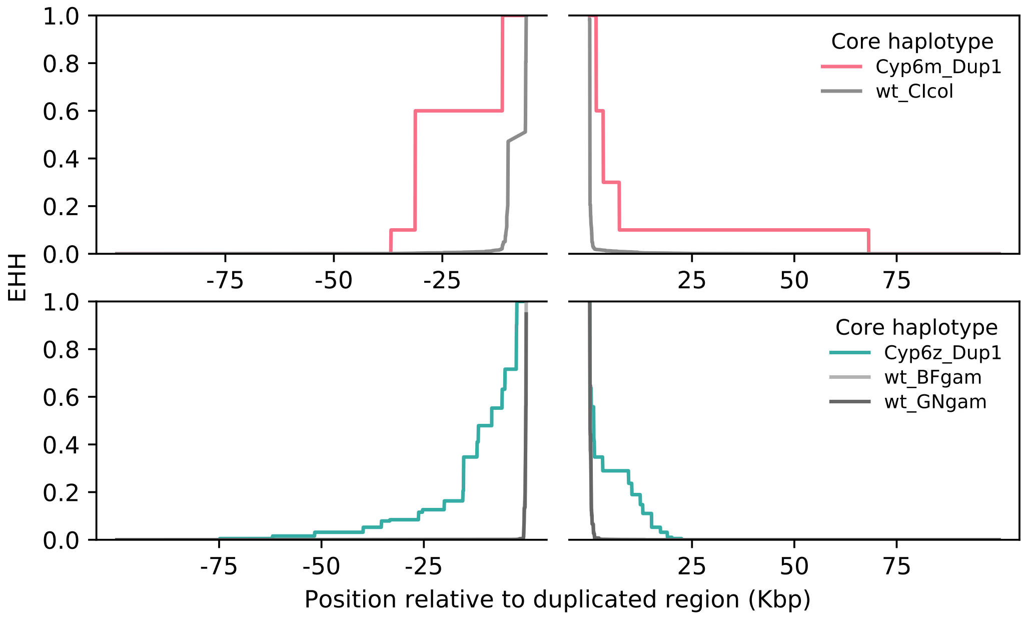

Fig. S6: Evidence for prolonged linkage disequilibrium around CNVs in the *Cyp6m* and *Cyp6z* gene clusters. Extended Haplotype Heterozygosity (EHH) decay was calculated around CNV and non-CNV (wt) haplotypes using SNPs from outside the region containing CNVs (break in the x axis). BF = Burkina Faso, CI = Côte d'Ivoire, GN = Guinea, col = *An. coluzzii*, gam = *An. gambiae*.

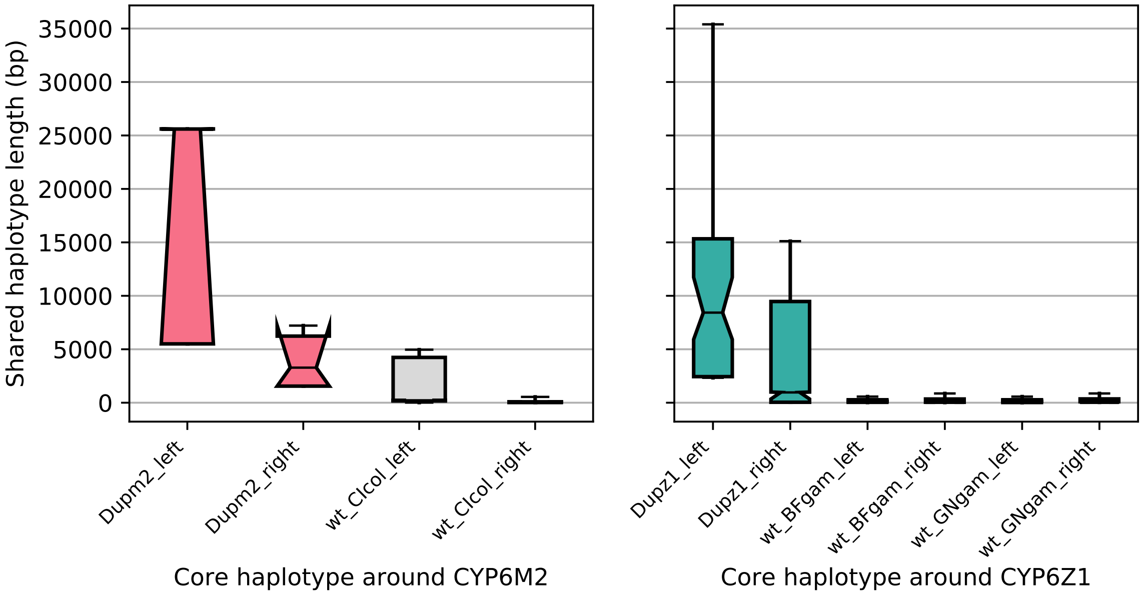

Fig. S7: Lengths of pairwise shared haplotypes are greater between samples sharing a CNV allele than between wild-type samples. Shared haplotype lengths were calculated on either side of the CNV-containing region of the *Cyp6m* and *Cyp6z* gene clusters. Non-CNV (wt) samples were taken from the same populations as the focal CNV alleles. Bars show the distribution of shared haplotype lengths between all haplotype pairs with the same core haplotype. Bar limits show the inter-quartile range, fliers show the 5th and 95th percentiles, horizontal black lines show the median, notches in the bars show the bootstrapped 95% confidence interval for the median. BF = Burkina Faso, CI = Côte d'Ivoire, GN = Guinea, col = *An. coluzzii*, gam = *An. gambiae*.

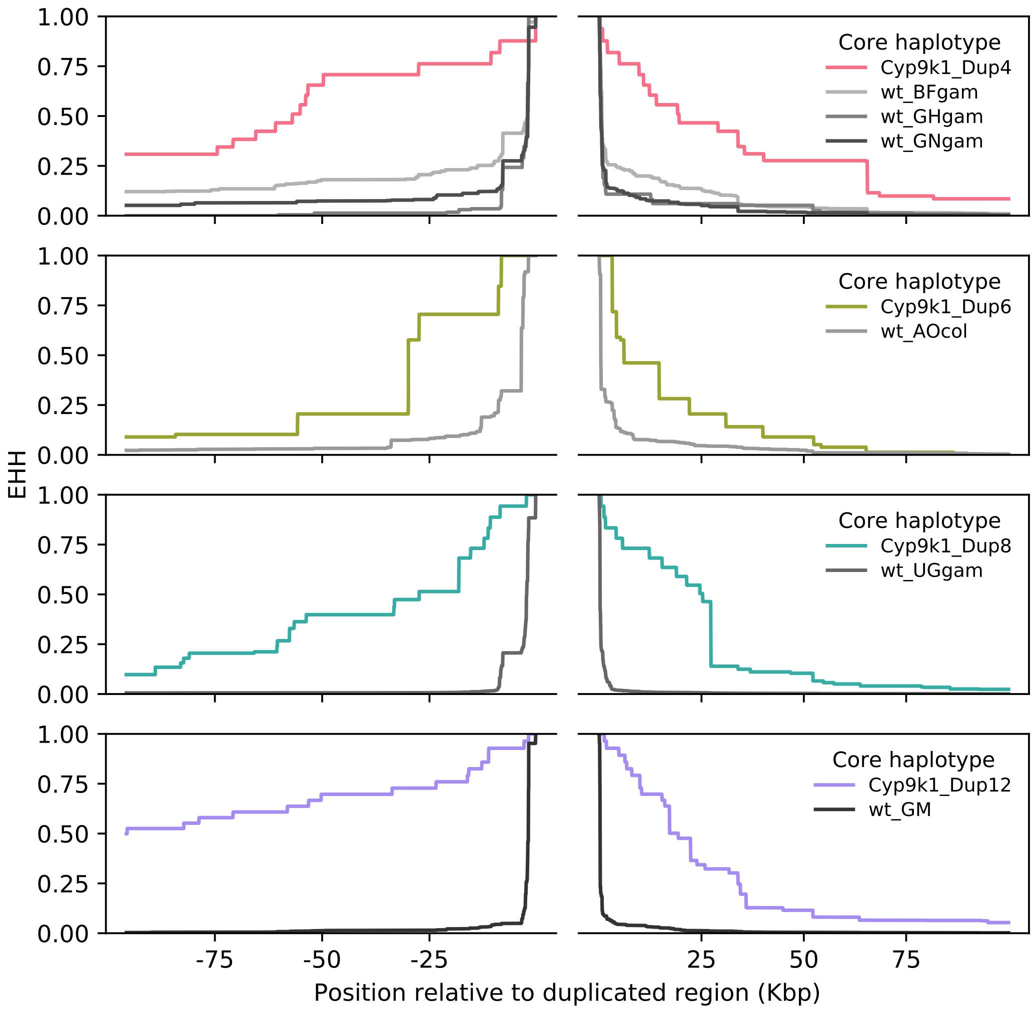

Fig. S8: Evidence for prolonged linkage disequilibrium around CNVs in *Cyp9k1*. Extended Haplotype Heterozygosity (EHH) decay was calculated around CNV and non-CNV (wt) haplotypes using SNPs from outside the region containing CNVs (break in the x axis). AO = Angola, BF = Burkina Faso, GH = Ghana, GM = The Gambiae, GN = Guinea, UG = Uganda, col = *An. coluzzii*, gam = *An. gambiae*.

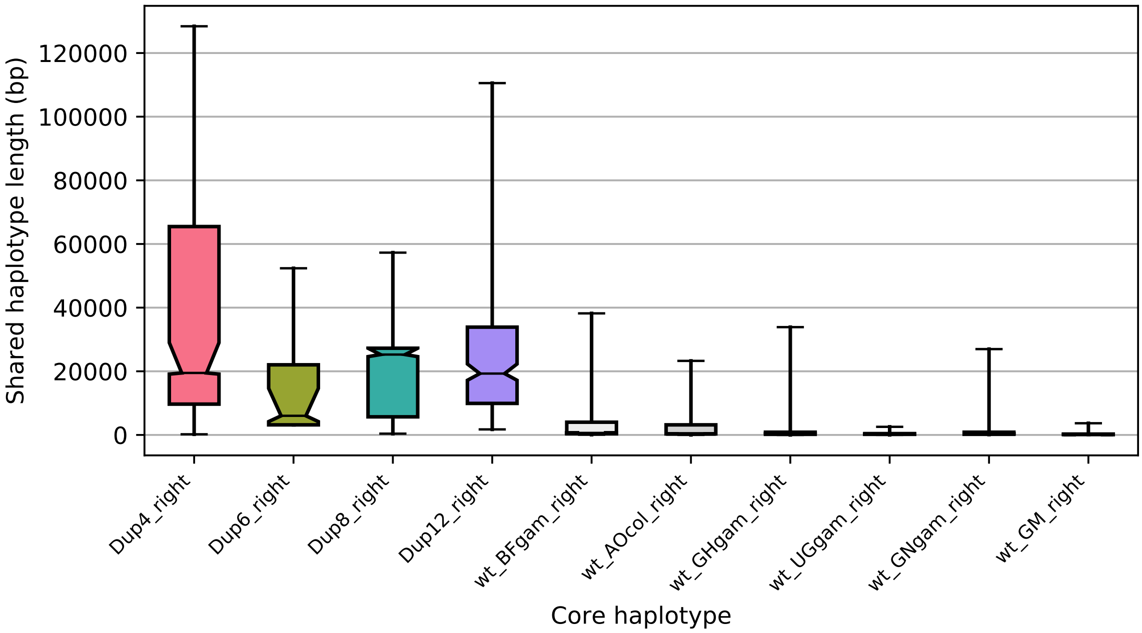

Fig. S9: Lengths of pairwise shared haplotypes are greater between samples sharing a CNV allele than between wild-type samples. Shared haplotype lengths were calculated on either side of the CNV-containing region of *Cyp9k1*. Non-CNV (wt) samples were taken from the same populations as the focal CNV alleles. Bars show the distribution of shared haplotype lengths between all haplotype pairs with the same core haplotype. Bar limits show the inter-quartile range, fliers show the 5th and 95th percentiles, horizontal black lines show the median, notches in the bars show the bootstrapped 95% confidence interval for the median. AO = Angola, BF = Burkina Faso, GH = Ghana, GM = The Gambiae, GN = Guinea, UG = Uganda, col = *An. coluzzii*, gam = *An. gambiae*.

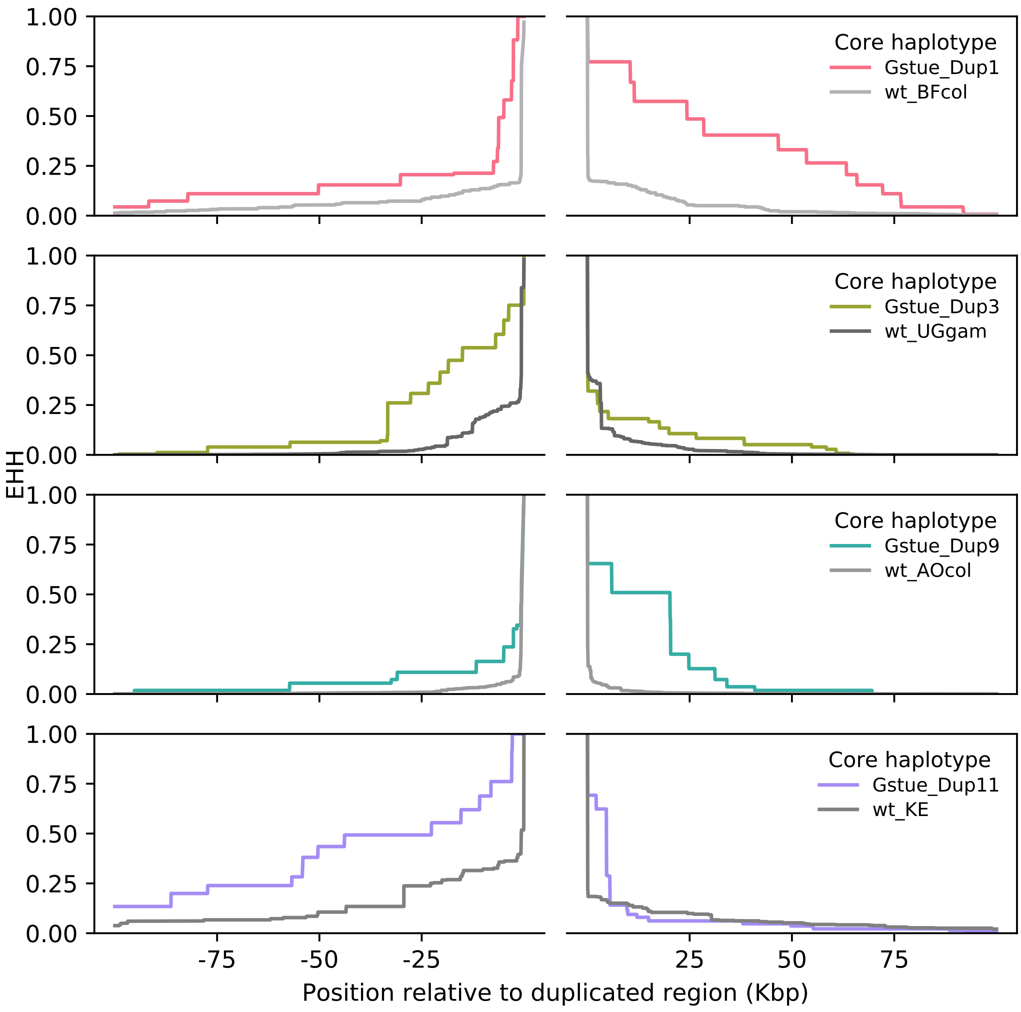

Fig. S10: Evidence for prolonged linkage disequilibrium around CNVs in the *Gstu4 - Gste3* gene cluster. Extended Haplotype Heterozygosity (EHH) decay was calculated around CNV and non-CNV (wt) haplotypes using SNPs from outside the region containing CNVs (break in the x axis). AO = Angola, BF = Burkina Faso, KE = Kenya, UG = Uganda, col = *An. coluzzii*, gam = *An. gambiae*.

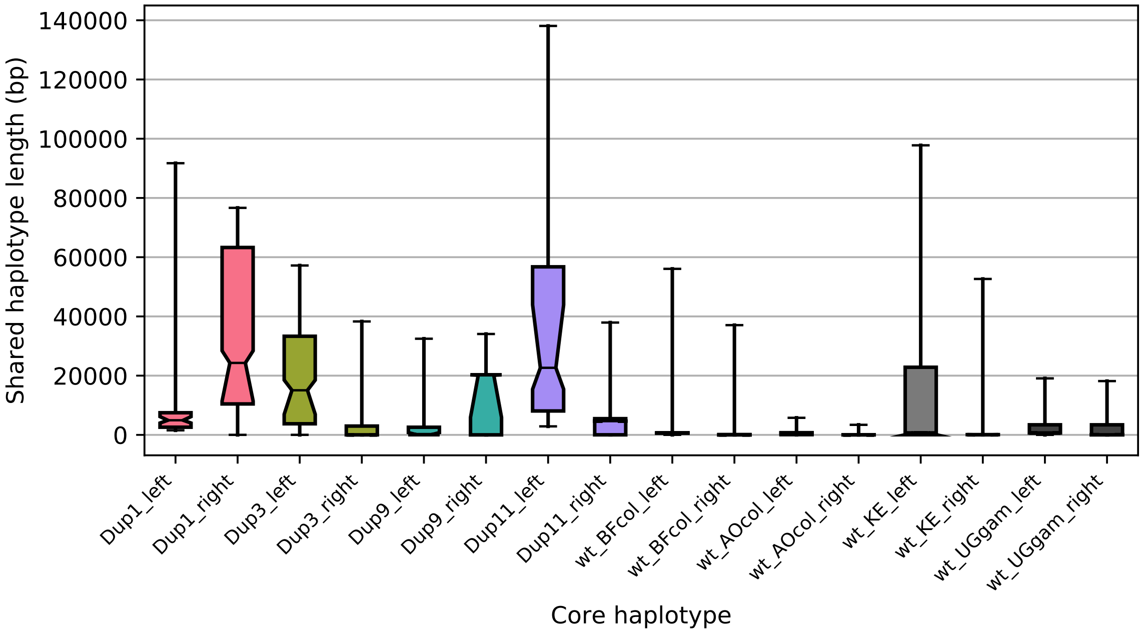

Fig. S11: Lengths of pairwise shared haplotypes are greater between samples sharing a CNV allele than between wild-type samples. Shared haplotype lengths were calculated on either side of the CNV-containing region of the *Gstu4* - *Gste3* gene cluster. Non-CNV (wt) samples were taken from the same populations as the focal CNV alleles. Bar limits show the inter-quartile range, fliers show the 5th and 95th percentiles, horizontal black lines show the median, notches in the bars show the bootstrapped 95% confidence interval for the median. AO = Angola, BF = Burkina Faso, KE = Kenya, UG = Uganda, col = *An. coluzzii*, gam = *An. gambiae*.

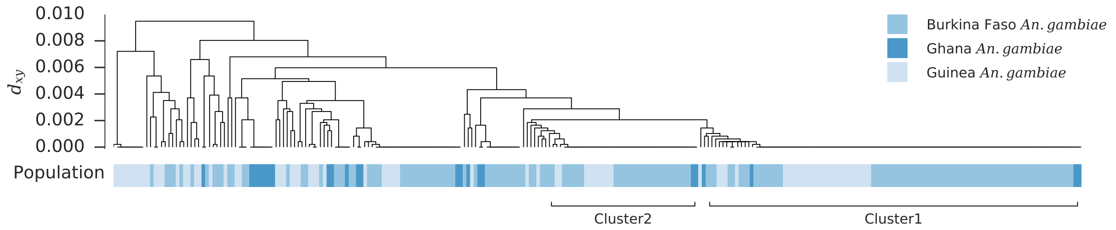

Fig. S12: Hierarchical clustering of haplotypes of *An. gambiae* from Burkina Faso, Guinea and Ghana, highlighting the two largest haplotype clusters.

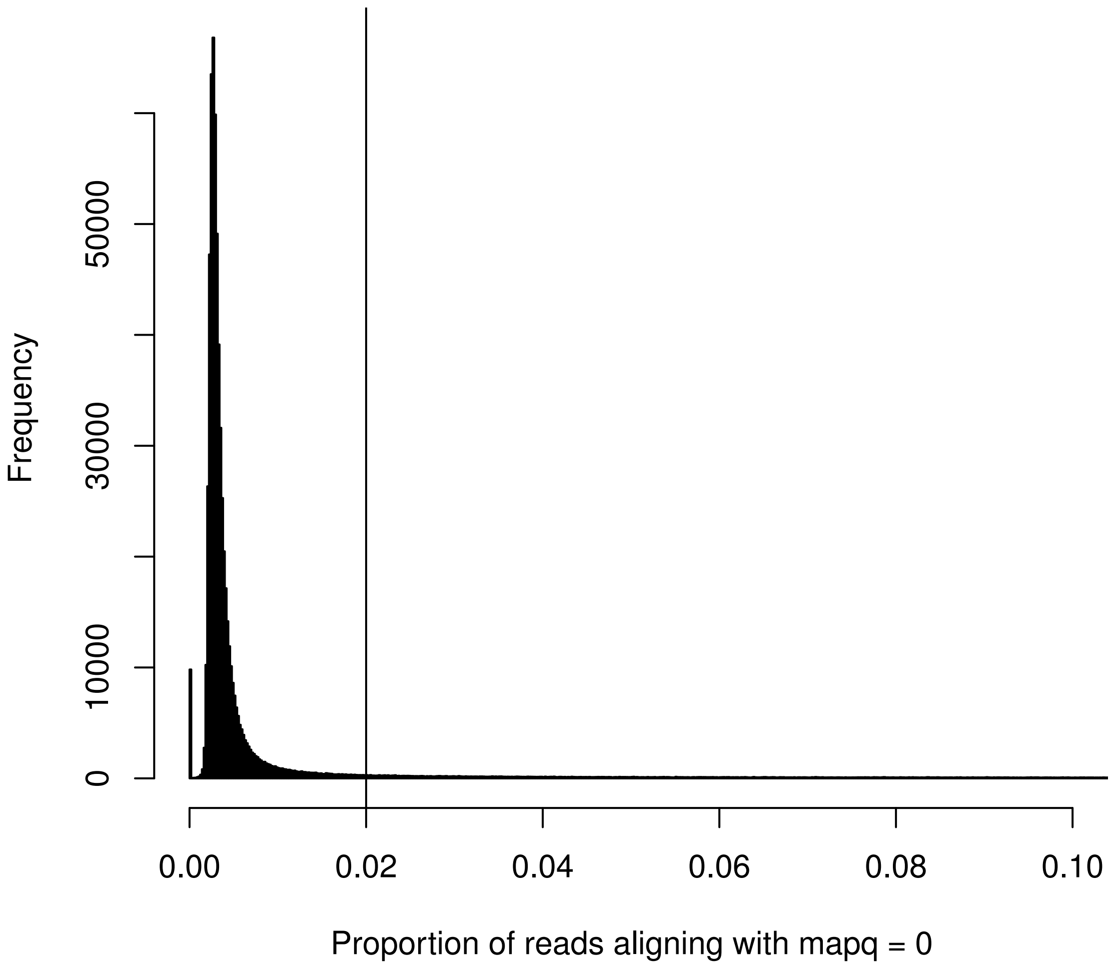

Fig. S13: Histogram of the proportion of Ag1000G phase 2 reads aligning with mapping quality 0 (mapq0) in all 300bp windows in the *An. gambiae* genome. Samples with mapq0 greater than 0.02 (dashed line) were excluded from further analysis.

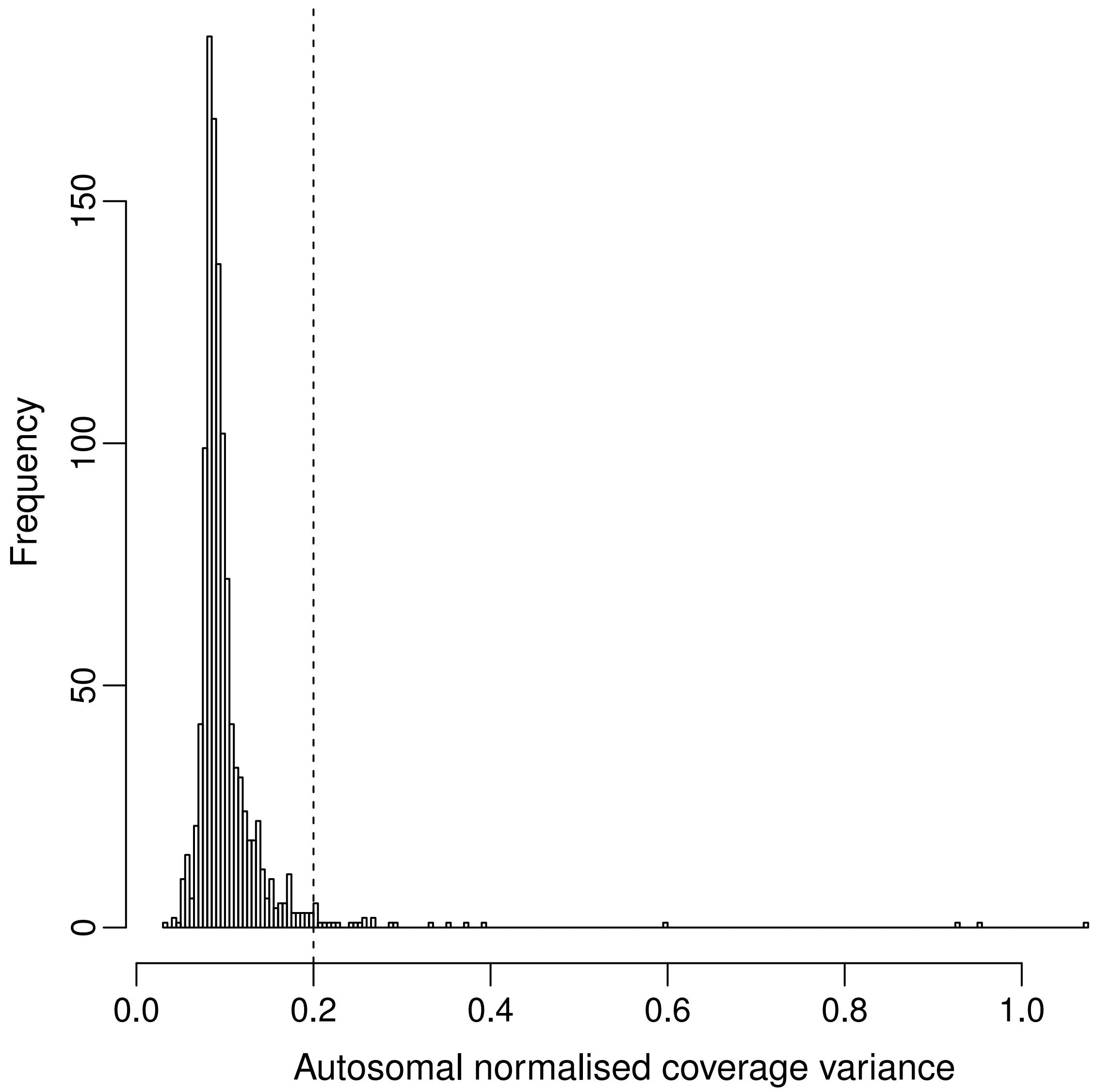

Fig. S14: Histogram of autosomal normalised coverage variance in all 1142 samples from Ag1000G phase 2. Samples with variance greater than 0.2 (dashed line) were excluded from the CNV detection analysis.

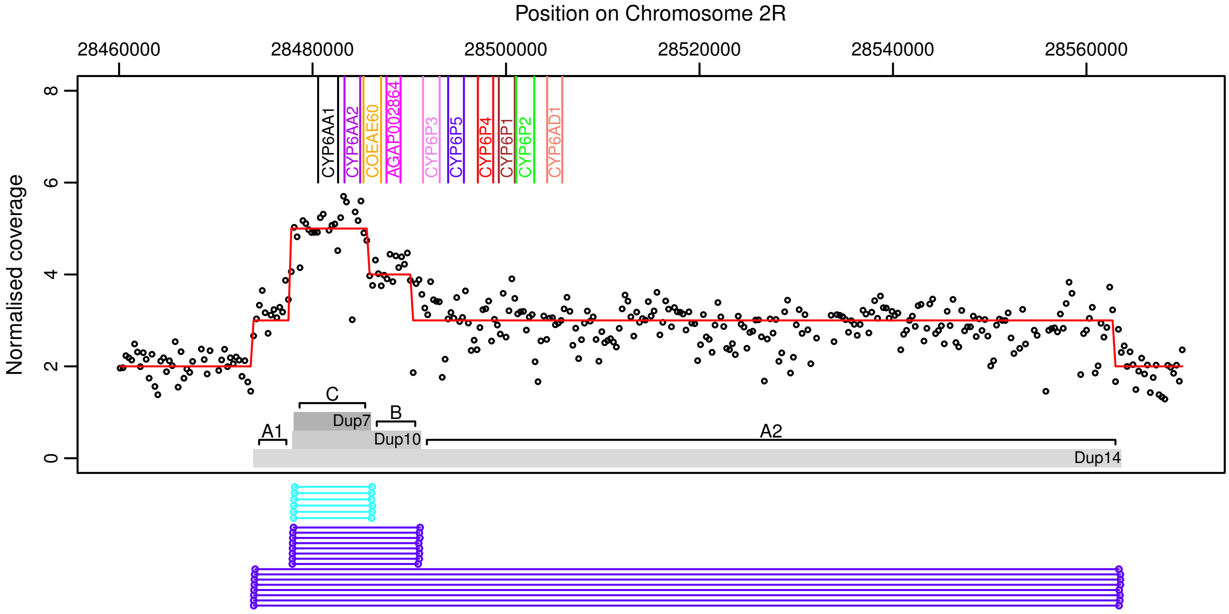

Fig. S15: Illustration of the process for determining CNV allele-specific copy numbers in a sample of *An. coluzzii* from Burkina Faso. Black circles represent coverage in each 300 bp window and the red line shows the copy number state predicted by the HMM. Colored vertical bars at the top indicate the position of the cytochrome P450 genes in this region. Using the face-away read pairs (dark blue circles joined by lines) and same-strand read pairs (cyan circles joined by lines) we determine that Dup7, Dup10 and Dup14 are present in this sample (Supplementary Information S1). The genomic regions covered by each of these three CNV alleles is shown in the grey rectangles. Dup7 and Dup10 are both completely overlapped by Dup14 and thus their copy number cannot be initially calculated. Dup14 has regions A1 and A2, which are not overlapped by any other duplication. We therefore use the HMM output (red line) in these regions to determine that the copy number of Dup14 in this individual is 1 (3 minus the background diploid coverage of 2). Region B is included in Dup10 and not Dup7. Now that we know the copy number of Dup14, we use the red line in region B to determine that the copy number of Dup10 is 1 (4 minus the background diploid coverage of 2 and minus the Dup14 copy number of 1). We now use the red line in region C to determine that the copy number of Dup7 is 1 (5 minus the background diploid coverage of 2, minus the Dup14 copy number of 1 and minus the Dup10 copy number of 1). If the red line had been too variable in regions A1 and A2 (fewer than 70% of windows sharing the same copy-number state), then the copy-number estimation would have failed for Dup14, causing the estimation to also fail for Dup10 and Dup7.

Table **S1**: Results of simulations that randomised the positions of CNVs found in the euchromatin, divided by size category of the CNVs. Shown are the number of CNVs of that size category, the number that contained genes in the real data, and the mean and range of the number of CNVs that contained genes across the 10,000 simulations, and the P value calculated by comparing the observed data to the null distribution obtained from the simulations.

CNV size	total CNVs	CNVs with genes	CNVs with genes in simulations mean (range)	P value
all sizes	1023	226	92 (59 - 127)	< 0.0002
< 6000bp	758	130	42 (21 - 68)	< 0.0002
$6000 - 15000$ bp	176	59	25 (10 - 43)	< 0.0002
≥ 15000 bp	89	37	25 (11 - 38)	0.0012

Table **S2**: Results of simulations randomising genes covered by gene-containing CNVs found in the euchromatin, divided by CNV size category. Shown are the number of CNVs of that category that contained genes in the real data, the mean and range of the number of CNVs containing detox genes across the 10,000 simulations, and the *P* value calculated by comparing the observed data to the null distribution obtained from the simulations.

CNV size	CNVs with genes	CNVs with detox genes	CNVs with detox genes in simulations mean (range)	*P* value
all sizes	226	25	4 (0 - 13)	< 0.0002
< 6000bp	130	6	2 (0 - 9)	0.018
$6000 - 15000$ bp	59	9	1 (0 - 6)	< 0.0002
≥ 15000 bp	37	10	1 (0 - 7)	< 0.0002

Table **S3**: Perfect association between the presence of of Cyp9k1_Dup11 and membership of haplotype Cluster 1 in male samples of *An. gambiae* from Burkina Faso, Ghana and Guinea. Males are haploid on chromosome X, thus excluding the existence of heterozygotes.

	Cyp9k1_Dup11 copy number			
	0	1	2	3
Haplotype not in Cluster 1	17	0	0	0
Haplotype in Cluster 1	0	1	6	1

Table **S4**: Copy number of Cyp9k1_Dup15 and membership of haplotype Cluster 2 in male samples of *An. gambiae* from Burkina Faso, Ghana and Guinea.

	Cyp9k1_Dup15 copy number		
	0	1	2
Haplotype not in Cluster 2	17	0	2
Haplotype in Cluster 2	2	1	3

Table **S5**: Number of reads aligned to the I114 amino acid position in *Gste2* and supporting either a wile-type ("I114") or mutant ("114T") allele in each of the 16 samples carrying the Gstue_Dup1 CNV. The last column shows the number of copies of Gstue_Dup1 in the sample. All of these samples have 114T reads, indicating that the mutation is present on the duplicated haplotype. Furthermore, all of the samples have more 114T than I114 reads, and many samples, including the one sample homozygous for Gstue_Dup1 (AB0123-C), are homozygote for the mutant 114T allele. This indicates that both copies of *Gste2* on Gstue_Dup1 haplotype carry the 114T mutation. Sample AB0139-C does not have a copy number because its coverage was too variable.

Sample name	I114	114T	Gstue_Dup1
AB0088-C	0	48	1
AB0095-C	0	60	1
AB0097-C	18	32	1
AB0101-C	12	30	1
AB0123-C	0	104	2
AB0138-C	28	48	1
AB0139-C	19	41	?
AB0182-C	1	34	1
AB0188-C	21	34	1
AB0204-C	0	58	1
AB0215-C	22	58	1
AB0246-C	0	57	1
AB0248-C	0	50	1
AB0250-C	12	40	1
AB0263-C	0	53	1
AB0279-C	0	74	1
