# Supplementary material for: Whole-genome sequencing reveals high complexity of copy number variation at insecticide resistance loci in malaria mosquitoes
Source: Genome Res. 2019 Aug;29(8):1250–61. doi: 10.1101/gr.245795.118 (PMC6673711; doi:10.1101/gr.245795.118)
Supplement: Supplemental Material [file supp_gr.245795.118_Supplementary_Methods.pdf]

Whole genome sequencing reveals high complexity of copy number variation at insecticide resistance loci in malaria mosquitoes

Eric R. Lucas, Alistair Miles, Nicholas J. Harding, Chris S. Clarkson, Mara K. N. Lawniczak, Dominic P. Kwiatkowski, David Weetman, Martin J. Donnelly and The *Anopheles gambiae* 1000 Genomes Consortium

Electronic Supplementary Material

Supplementary methods

SM1 Novel Phase 2 natural populations

This section describes the collection methods for the populations in Ag1000G that are novel to Phase 2. Collection methods for other sites have been previously published (The Anopheles gambiae 1000 Genomes Consortium 2017). Unless otherwise stated, the DNA extraction method used for the collections described below was Qiagen DNeasy Blood and Tissue Kit (Qiagen Science, MD, USA).

Côte d'Ivoire (CIcol): Tiassalé (-4.82293, 5.89839) is located in the evergreen forest zone of southern Côte d'Ivoire. The primary agricultural activity is rice cultivation in irrigated fields. High malaria transmission occurs during the rainy seasons, between May and November. Samples were collected as larvae from irrigated rice fields by dipping between May and Septermber 2012. All larvae were reared to adults and females preserved over silica for DNA extraction. Specimens from this site were all *An. coluzzii*, determined by PCR assay (Santolamazza et al. 2008)

Bioko Island - Equatorial Guinea (GQgam): Collections were performed during the rainy season in September, 2002 by overnight CDC light traps in Sacriba of Bioko island (8.7, 3.7). Specimens were stored dry on silica gel before DNA extraction. Specimens contributed from this site were *An. gambiae* females, genotype determined by two assays (Scott, Brogdon, and Collins 1993; Santolamazza, Torre, and Caccone 2004). All specimens had the $2L^{+a}/2L^{+a}$ karyotype as determined by the molecular PCR diagnostics (White et al. 2007). These mosquitoes represent a population that inhabited Bioko Island before a comprehensive malaria control intervention initiated in February 2004 (Sharp et al. 2007). After the intervention *An. gambiae* was declining, and more recently almost only *An. coluzzii* can be found (Overgaard et al. 2012).

Mayotte Island - France (FRgam): Samples were collected as larvae during March-April 2011 in temporary pools by dipping, in Bouyouni (-12.737813, 45.141696), M'Tsamboro Forest Reserve (-12.70271, 45.081091), Combani (-12.778704, 45.142913), Mtsanga Charifou (-12.990662, 45.155673), Karihani Lake forest reserve (-12.796525, 45.121722), and Sada (-12.852147, 45.103891) in Mayotte island. Larvae were stored in 80% ethanol prior to DNA extraction. All specimens contributed to Ag1000G phase 2 were *An. gambiae* (Santolamazza, Torre, and Caccone 2004) with the standard $2L^{+a}/2L^{+a}$ or inverted $2L^{a}/2L^{a}$ karyotype as determined by the molecular PCR diagnostics (White et al. 2007). The samples were identified as males or females by the sequencing read coverage of the X chromosome using LookSeq (Manske and Kwiatkowski 2009).

The Gambia (GM): Indoor resting female mosquitoes were collected by pyrethrum spray catch from four hamlets around Njabakunda (-15.9, 13.55), North Bank Region, The Gambia between August and October 2011. The four hamlets were Maria Samba Nyado, Sare Illo Buya, Kerr Birom Kardo, and Kerr Sama Kuma; all are within 1 km of

each other. This is an area of unusually high hybridization rates between *An. gambiae s.s.* and *An. coluzzii* (Caputo et al. 2008; Nwakanma et al. 2013). Njabakunda village is approximately 30 km to the west of Farafenni town and 4 km away from the Gambia River. The vegetation is a mix of open savannah woodland and farmland. With apparent high gene-flow in the region, it is problematic to assign species to these samples.

Ghana (GHcol/GHgam): Twifo Praso (5.60858, -1.54926) a peri-urban community located in semi-deciduous forest in the Central Region of Ghana. It is an extensive agricultural area characterised by small-scale (vegetable growing) and large-scale commercial farms such as oil palm and cocoa plantations. Mosquito samples were collected as larvae from puddles near farms between September and October, 2012. Madina (5.66849 -0.21928) is suburb of Accra within a coastal savanna zone of Ghana. It is an urban community characterised by myriad vegetable-growing areas. The vegetation consists of mainly grassland interspersed with dense short thickets often less than 5 m high with a few trees. Specimens were sampled from puddles near roadsides and farms between October and December 2012. Takoradi (4.91217, -1.77397) is the capital city of Western Region of Ghana. It is an urban community located in the coastal savanna zone. Mosquito samples were collected from puddles near road construction and farms between August and September 2012. Koforidua (6.09449, -0.26093) is a capital city of Eastern Region of Ghana and is located in semi-deciduous forest. It is an urban community characterized by numerous small-scale vegetable farms. Samples were collected from puddles near road construction and farms between August and September 2012. Larvae from all collection sites were reared to adults and females preserved over silica for DNA extraction. Both *An. gambiae* and *An. coluzzii* were collected from these sites, determined by PCR assay (Santolamazza et al. 2008).

Guinea-Bissau (GW): Mosquitoes were collected in October 2010 using indoor CDC light traps, in the village of Safim (11.956889, -15.649222), ca. 11 km north of Bissau city, the capital of the country. Malaria is hyperendemic in the region and transmitted by members of the Anopheles gambiae complex (Vicente et al., 2017). *Anopheles arabiensis, An. melas, A. coluzzii* and *A. gambiae*, as well as hybrids between the latter two species, are known to occur in the region (Gordicho et al. 2014; Vicente et al. 2017). Mosquitoes were preserved individually on 0.5ml micro-tubes filled with silica gel and cotton. DNA extraction was performed by a phenol-chloroform protocol (Donnelly et al. 1999). Guinea-Bissau is another region where defining species is problematic (Vicente), so no species has been assigned here.

SM2 CNV discovery using hidden markov models

To detect the most likely copy-number state (CNS) at each window in each individual, we applied a gaussian hidden markov model (HMM) to the individual's normalised windowed coverage data. The HMM was implemented using the GaussianHMM function from the hmmlearn software package. The HMM contained 13 hidden states (c), representing CNS from 0 to 12 in increments of 1, allowing the detection of up to 6-fold amplification of a genetic region (the normal diploid complement of two copies of a genetic region is represented by a CNS of 2, a single duplication on one chromosome is represented by 3, and so on). The Gaussian emission probability distribution for each copy number state n had a mean c_n ($c_n = n$), with variance $v_n = 0.01 + a_n c_n$, where a_n is the variance in normalised coverage for all windows with at least 90% accessible sites in the sample (The Anopheles gambiae 1000 Genomes Consortium 2017). We determined the variance empirically for each individual because variance in coverage can differ between individuals (Supplementary Figure S14), presumably due to stochastic variation in library preparation and/or sequencing runs, and we also found evidence for this among the Ag1000G data (data not shown). We calibrated the HMM transition probability (t) by fitting the HMM in a genomic region genomic region on chromosome arm 3R spanning a cluster of glutathione S-transferase epsilon genes (*Gste*), where visual inspection of the normalised coverage data from Ag1000G phase 2 showed clear evidence for a small gene duplication encompassing *Gste2* in multiple individuals (named Gstue-Dup1 in our subsequent nomenclature). No studies have previously identified specific copy number variants in *Gste2*, however this gene has been found to be over-expressed in mosquitoes resistant to insecticides (Ding et al. 2003; David et al. 2005). Larger values of t tend to increase sensitivity to detect small amplifications, but also tend to falsely break up larger amplifications into multiple blocks due to occasional windows with lower coverage. We tested values ranging from $t = 10^{-13}$ to $t = 10^{-2}$ and found that the smallest value of t that was able to detect all instances of the duplication was $t = 0.00001$, which was then used for subsequent analysis. After parameter calibration, we fitted a Gaussian HMM to normalised windowed coverage data for each individual, to obtain a predicted copy number state within each window.

SM3 Calculating the likelihood ratio of CNVs against the null model

Likelihoods were calculated as the product of the values of the Gaussian probability density function for the observed coverage at each window multiplied by the transition probabilities between the states at each window. The same values of variance and transition probability were used as in the HMM model. For the null model, the mean of the

Gaussian was 2 at every window. For the model based on the HMM prediction, the mean was taken as the predicted CNV state at each window.

SM4 Using discordant reads to identify and detect CNV alleles

Four types of discordant read pairs were obtained from alignment files. Read pairs that mapped facing away from each other on the same chromosome indicate a tandem duplication and were recorded as Face-Away (FA) reads (main text Figure 3). Read pairs that mapped facing in the same direction on the same chromosome indicate a tandem inversion and were recorded as Same Strand (SS) reads (main text Figure 3b). Read pairs that mapped facing each other on the same chromosome, but more than 1000 base pairs apart indicate a deletion and were recorded as Far-Mapped (FM) reads (main text Figure 3c). Read pairs that mapped to different chromosomes indicate a more complex duplication, possibly associated with a transposable element, and were recorded as cross-chromosome (XC) reads. Discordant pairs were only recorded if the minimum mapping quality (mapq) of the pair was at least 10. Breakpoint reads were obtained by searching for soft-clipped reads with mapq ≥ 10 (main text Figure 3d).

SM5 Estimating copy numbers of CNV alleles

First, for each sample, the CNV alleles present were determined using diagnostic discordant and breakpoint reads. Next, where only one CNV allele was present, its copy number was calculated as the most frequent CNS over the allele's range, as long as that CNS had a frequency of at least 70%. This threshold allowed for small variations in HMM output over the range of the duplication. If the most frequent CNS was less frequent than the threshold, no coverage call was recorded for that duplication in that sample.

Complex CNV calls occurred where multiple overlapping CNV alleles were present in the same sample, confounding the estimation of copy number for each allele. In these cases, we attributed values of copy number to each CNV allele using the part of its range that did not overlap with any other CNV alleles in that sample, as long as this provided at least three windows of sequence. If fewer than three non-overlapping windows existed for a given CNV allele but copy number could be obtained for the alleles that it overlapped with, we calculated that CNV allele's copy number by subtracting the copy number attributed to the overlapping alleles (Figure S15).

References

Caputo, B. et al. (2008). *Anopheles gambiae* complex along The Gambia river, with particular reference to the molecular forms of An. gambiae ss. *Malaria Journal* 7(1):182.

David, J. et al. (2005). The *Anopheles gambiae* detoxification chip: a highly specific microarray to study metabolic-based insecticide resistance in malaria vectors. *Proceedings of the National Academy of Sciences of the United States of America* 102(11):4080–4084.

Ding, Y. et al. (2003). The *Anopheles gambiae* glutathione transferase supergene family: annotation, phylogeny and expression profiles. *BMC genomics* 4(1):35.

Donnelly, M. et al. (1999). Population structure in the malaria vector, *Anopheles arabiensis* Patton, in East Africa. *Heredity* 83(4):408.

Gordicho, V. et al. (2014). First report of an exophilic *Anopheles arabiensis* population in Bissau City, Guinea-Bissau: recent introduction or sampling bias? *Malaria Journal* 13(1):423.

Manske, H. M. and D. P. Kwiatkowski (2009). LookSeq: a browser-based viewer for deep sequencing data. *Genome Research* 19(11):2125–2132.

Nwakanma, D. C. et al. (2013). Breakdown in the process of incipient speciation in *Anopheles gambiae*. *Genetics*:1221–1231.

Overgaard, H. J. et al. (2012). Malaria transmission after five years of vector control on Bioko Island, Equatorial Guinea. *Parasites & Vectors* 5(1):253.

Santolamazza, F., A. della Torre, and A. Caccone (2004). A new polymerase chain reaction-restriction fragment length polymorphism method to identify *Anopheles arabiensis* from *An. gambiae* and its two molecular forms from degraded DNA templates or museum samples. *The American Journal of Tropical Medicine and Hygiene* 70(6):604–606.

Santolamazza, F. et al. (2008). Insertion polymorphisms of SINE200 retrotransposons within speciation islands of *Anopheles gambiae* molecular forms. *Malaria Journal* 7(1):163.

Scott, J. A., W. G. Brogdon, and F. H. Collins (1993). Identification of single specimens of the *Anopheles gambiae* complex by the polymerase chain reaction. *The American Journal of Tropical Medicine and Hygiene* 49(4):520–529.

Sharp, B. L. et al. (2007). Malaria vector control by indoor residual insecticide spraying on the tropical island of Bioko, Equatorial Guinea. *Malaria Journal* 6(1):52.

The Anopheles gambiae 1000 Genomes Consortium (2017). Natural diversity of the malaria vector *Anopheles gambiae*. *Nature* 552:96–100.

Vicente, J. L. et al. (2017). Massive introgression drives species radiation at the range limit of *Anopheles gambiae*. *Scientific Reports* 7:46451.

White, B. J. et al. (2007). Molecular karyotyping of the 2La inversion in *Anopheles gambiae*. *The American Journal of Tropical Medicine and Hygiene* 76(2):334–339.
